# Comprehensive analysis of IGF2BP3 with expression features, prognosis, immune modulation and stemness in hepatocellular carcinoma and pan-cancer

**DOI:** 10.7150/jca.92768

**Published:** 2024-03-25

**Authors:** Sha Qin, Haoer Jin, Yan Li, Xue Chen, Jiang He, Juxiong Xiao, Yan Qin, Chuyi Liu, Yitao Mao, Luqing Zhao

**Affiliations:** 1 Department of Pathology, Xiangya Hospital, Central South University, Changsha, Hunan, China; and Department of Pathology, School of Basic Medical Science, Xiangya School of Medicine, Central South University, Changsha, Hunan, China.; 2 Early Clinical Trial Center, Hunan Cancer Hospital and The Affiliated Cancer Hospital of Xiangya School of Medicine, Central South University, Changsha, Hunan, China.; 3 Center for Molecular Medicine, Xiangya Hospital, Central South University, Changsha, Hunan, China.; 4 Department of Radiology, Xiangya Hospital, Central South University, Changsha, Hunan, China.; 5 College of Bioscience and Biotechnology, Hunan Agricultural University, Changsha, Hunan, China.; 6 National Clinical Research Center for Geriatric Disorders, Xiangya Hospital, Central South University, Changsha, Hunan, China.

**Keywords:** IGF2BP3, expression features, prognosis, immune modulation, hepatocellular carcinoma (HCC), pan-cancer analysis.

## Abstract

Insulin like growth factor 2 mRNA binding protein 3 (IGF2BP3) is a critical m6A reader. It encodes proteins that contain several KH domains, which are important in RNA binding, RNA synthesis and metabolism. Lots of researches have studied the malignant potential of m6A readers in tumors. However, the biological functional analysis of IGF2BP3 in hepatocellular carcinoma (HCC) and pan-cancer is not comprehensive. In this study, we used a bioinformatics approach to comprehensively analyze the significance of IGF2BP3 in HCC through analyzing its expression, mutation, prognosis, protein-protein interaction (PPI) network, functional enrichment, and the correlation with ferroptosis, stemness as well as immune modulation in HCC. IGF2BP3 presented a negative correlation with the ferroptosis molecule NFE2L2, and a positive correlation with the ferroptosis molecule SLC1A5 as well as the immune checkpoint HAVCR2. In addition, we also analyzed IGF2BP3 expression, prognosis and immune modulation in pan-cancer, revealing the prognostic value of IGF2BP3 in a variety of tumors. Finally, we verified the biological functions of IGF2BP3 in HCC through various experiments. The data showed that IGF2BP3 may enhance the proliferation, colony formation and invasion capacities of HCC cells, and IGF2BP3 is mainly positively correlated with the expression level of stemness marker SOX2. In conclusion, IGF2BP3 had a potential to be a new perspective biomarker in forecasting the immune response, ferroptosis, stemness and prognosis of HCC or even pan-cancer.

## Introduction

Cancers are the common cause of death and a significant obstacle to improving life expectancy in countries worldwide 1. Based on the global cancer statistics 2020, primary liver cancer was the sixth commonest cancer and the third most frequent cause of mortality due to cancer worldwide. In addition, hepatocellular carcinoma (HCC) was the utmost frequent primary liver cancer, accounting for about 75% to 85% of cases 2. The current therapeutic modes for HCC include drug therapy (such as sorafenib, lenvatinib, and regorafenib), percutaneous ablation, trans-arterial chemoembolization (TACE), chimeric antigen receptor engineered T-cell immunotherapy (CAR-T), HCC resection, and liver transplantation 3. However, the mortality rate of HCC patients remains high. Therefore, it is urgent to find new therapeutic directions.

Cancer stem cells (CSCs) are a unique subset of undifferentiated cells with stem cell-like properties and have been considered to be the driving force for tumor growth, metastasis and therapeutic resistance 4. Tumor stemness refers to the stem cell-like phenotype of tumor cells, which plays an important role in different aspects of HCC 5. The stemness characteristics of CSCs depend on a variety of molecular targets, involving genetic and epigenetic factors of various signaling pathways, tumor microenvironment, apoptotic pathways, microRNA, stem cell differentiation and drug resistance. Inhibition of these stem cell molecular targets is one of the effective treatment strategies to eliminate tumor stemness 6.

N6-methyladenosine (m6A) modifications, as prevalent modifications in RNA, are involved in the regulation of RNA destiny, including the stability, splicing, degradation, translation, as well as exportation of RNA. M6A regulators include “writers”, “readers”, and “erasers”, which increase, recognize and remove m6A modifications on RNA and thus play an important role in tumors 7. The aberrant m6A modification could alter the tumor immune response in various cancers by regulating immune cell infiltration, pro-tumor inflammation, immune suppression, immune surveillance and anti-tumor immune response 8, 9. m6A modifications were found to alters HCC progression by affecting immune cell recruitment and pro-tumor inflammation 10. In addition, m6A modifications altered the proliferation and metastasis of gastric cancer by participating in the infiltration of immune cells and activation of immune pathways 11.

Insulin like growth factor 2 mRNA binding protein 3 (IGF2BP3) is a critical m6A “reader” and a growing amount of research suggests that IGF2BP3 has an essential role in the development of tumors 12, 13. IGF2BP3, as a protein coding gene, encodes proteins that contain several KH domains, which are important in RNA binding, RNA synthesis and metabolism 14. Although IGF2BP3 has been discovered to exert cancer-promoting functions in HCC 15, 16, comprehensive bioinformatics analysis of IGF2BP3 in HCC and pan-cancer is scarce. The purpose of this study is to provide more new ideas on the pathogenic mechanisms and more therapeutic possibilities for the treatment of HCC and pan-cancer.

In this paper, a comprehensive bioinformatics analysis of IGF2BP3 in HCC was performed by using the tumor and normal tissue data from TCGA and GETx databases. The expression pattern, prognostic value, mutation status, protein-protein interaction, function enrichment analysis, and pertinent pathways of IGF2BP3 in HCC were conducted. Moreover, the correlation between IGF2BP3 and ferroptosis as well as immune modulation in HCC were also dissected and a series of potential targets for IGF2BP3 have been identified, which might give some new possibilities for a targeted treatment of HCC. We analyzed the prognosis value of IGF2BP3 as well as the relationship between IGF2BP3 and immune checkpoints, immune cells, TMB, MSI, immunoinhibitors, immunostimulators, and MHCs in pan-cancer. Finally, we verified the differential expression and biological functions of IGF2BP3 in HCC, and the association between IGF2BP3 and HCC stemness through various experiments.

## Materials and methods

### Data collection

RNAseq data as well as clinical information of HCC patients were derived from The Cancer Genome Atlas (TCGA) database 17 (https://portal.gdc.com) and International Cancer Genome Consortium (ICGC) database 18 (https://dcc.icgc.org/releases/current/Projects). The RNA-seq data of pan-cancer samples including 33 kinds of cancer were also obtained from TCGA dataset. 226 samples of normal tissues were obtained from the GTEx database 19 (http://commonfund.nih.gov/GTEx). The IGF2BP3 protein expression level in HCC and normal tissue was derived from the Human Protein Atlas (HPA) database 20 (https://www.proteinatlas.org/). Ferroptosis-related genes were obtained from previous research by Ze-Xian Liu et al 21. And the m6A-related genes were obtained from the research by Juan Xu et al 22.

### Gene expression analysis

Limma package of R software (version:3.40.2) were used to get the differential mRNA expression level. Adjusted *P* value < 0.05 and log2 (fold change) > 1 or log2 (fold change) < -1 was selected as mRNA differential expression threshold. “ggplot2 package” was applied to depict boxplot; and “pheatmap package” was applied to depict heatmap.

### Functional enrichment analysis

Functional enrichment analysis of differentially expressed genes was performed to further confirm the function of the target molecules. The ClusterProfiler package (version: 3.18.0) in R was used to identify promising mRNAs for GO and KEGG analysis.

### Survival analysis

Log-rank test and univariate cox proportional hazards regression were used to calculate p-values and hazard ratios (HR) with 95% confidence intervals (CI) using the R packages “survivor” and “survminer”. R software package “ggalluvial” was used to draw Sankey diagram. In addition, the "pROC package" was used for ROC analysis and the "ggplot2 package" was used for ROC visualization. *P* < 0.05 were regarded as statistical significance.

Univariate and multivariate cox regression analyses were used in this study, and forest plots were drawn by the "forestplot" package to show each variable (*P*, HR and 95% CI). In addition, the cBioPortal (https://www.cbioportal.org/) database 23 was applied to acquire genomic alteration of IGF2BP3 in LIHC from TCGA.

### Protein-protein interaction analysis

The IGF2BP3 co-expression genes were downloaded from cBioPortal. We used STRING (https://string-db.org/) database 24 to get protein-protein interaction. Furthermore, we used Cytoscape software (version 3.9.0) to integrate co-expression genes of IGF2BP3 co-expression genes that obtained from cBioPortal. The co-expression genes all showed high spearman's correlation with IGF2BP3 (>0.4). In addition, we selected hub genes through cytoHubba plug.

### Correlation analysis between genes and pathways

We collected the gene sets in the relevant pathways from the literature 25, and calculated the enrichment scores for each sample on each pathway according to the ssGSEA algorithm to obtain the association between HCC samples and pathways. Analysis was performed using the R software GSVA package with the parameter selected as method='ssgsea'. Correlations between gene and pathway scores were analyzed by Spearman's correlation method.

### Pan-cancer correlation analysis of IGF2BP3 expression, prognosis, immune modulation, TMB and MSI

TMB data obtained from previous research by Vesteinn Thorsson et al. 26; And MSI data obtained from Russell Bonneville et al. 27. We used immuneeconv R software package to assess the immune score evaluation. TISIDB database (http://cis.hku.hk/TISIDB/index.php) 28 was used to get spearman correlations between the expression level of IGF2BP3 and immunoinhibitors, immunostimulators, immune subtypes as well as MHCs among human cancers. We obtained the expression data of IGF2BP3 before and after cytokine treatment in various of cancer cell lines through TISMO database (http://tismo.cistrome.org/) 29. Moreover, the distribution of immune cells after immunotherapy and their expression data of IGF2BP3 were obtained from the TISCH2 database (http://tisch.comp-genomics.org/) 30. R software v4.0.3. were used for statistical analyses.

### Cell culture, lentiviral transduction, and RT-PCR

PLC, Hep3B, HepG2 cell lines were cultured in MEM with 10% FBS. THLE-3 and Huh-7 cell lines were cultured in DMEM with 10% FBS. The materials and methods are as before 31. And primer sequences were as follows:

IGF2BP3-F: 5′-ACGAAATATCCCGCCTCATTTAC-3′,

IGF2BP3-R: 5′-GCAGTTTCCGAGTCAGTGTTCA-3′ (reverse);

SLC1A5-F: TCCTCTTCACCCGCAAAAACCC,

SLC1A5-R: CCACGCCATTATTCTCCTCCAC;

PDCD1-F: AGCCCCAGCAACCAGAC,

PDCD1-R: GCCCCACAGAGGTAGGTG;

ACSL4-F: GGAATGACAGGCCAGTGTGA,

ACSL4-R: TAGCACATGAGCCAAAGGCA;

HAVCR2-F: AGGAGCCTGTCCTGTGTTTG,

HAVCR2-R: GGACACATCTCCTTTGCGGA;

CD274-F: CTGGCATTTGCTGAACGCAT,

CD274-R: AGTGCAGCCAGGTCTAATTGT;

SLC7A11-F: TCCTGCTTTGGCTCCATGAACG,

SLC7A11-R: AGAGGAGTGTGCTTGCGGACAT;

NFE2L2-F: CACATCCAGTCAGAAACCAGTGG,

NFE2L2-R: GGAATGTCTGCGCCAAAAGCTG;

TFRC-F: GCTGCCAGCTTTACTGGAGA,

TFRC-R: CGTCACCAGAGAGGGCATTT.

SOX2-F: ACGCTCATGAAGAAGGATAAGT,

SOX2-R: GAGCTGGTCATGGAGTTGTAC;

Nanog-F: CCTATGCCTGTGATTTGTGG,

Nanog-R: GATCCATGGAGGAAGGAAGA;

Epcam-F: AATCGTCAATGCCAGTGTACTT,

Epcam-R: TCTCATCGCAGTCAGGATCATAA;

OCT4-F: GAGAAGGATGTGGTCCGAGT,

OCT4-R: GTGCATAGTCGCTGCTTGAT;

ALDH1A1-F: TAGCTGATGCCGACTTGGAC,

ALDH1A1-R: AACACTGTGGGCTGGACAAA;

The shRNA sequences are as follows:

shIGF2BP3#1: 5'-GCAGGAATTGACGCTGTAT-3',

shIGF2BP3#2: 5'-TAATCCAGGAATTAAATGTGC-3',

shNC: 5'-TTCTCCGAACGTGTCACGT-3'.

### Western blotting

The total protein of cells was extracted with RIPA lysate (Beyotime, China). The concentration was determined by BSA standard protein and separated by SDS-PAGE. anti-IGF2BP3(14642-1-AP), anti-α-tublin (66031-1-Ig) and anti-SOX2 (66411-1-Ig) were purchased from Proteintech (Wuhan, China).

### Cell proliferation

Cell proliferation assay Cell Counting Kit-8 kit was used and operated according to the instructions. The cells were seeded in 96-well plates (3000 cells / well) and cultured at 37 °C. The absorbance at 450 nm was measured at 0,24,48 and 72h respectively.

### Colony formation assay

1000 cells were inoculated into 6-well plates and cultured for two weeks. The number of colonies with > 50 cells was observed and counted under an optical microscope.

### Transwell invasion assay

A total of 100μl of Matrigel (Corning, USA) with a concentration of 200μg/ml was evenly spread to the bottom of the Transwell chamber. The chamber was placed in a 24-well plate and incubated in a 37 ℃ incubator for 1 hour to form a gel. The cells were cultured to logarithmic growth phase, digested, suspended in serum-free medium, counted, and adjusted to a concentration of 2 × 10^5^/ml. Add 800μl complete medium containing 20% fetal bovine serum to the lower chamber, and add 200μl cell suspension to the upper chamber of the Transwell chamber. Put them in the incubator for 40hrs. The cells were fixed and stained, and observed under an optical microscope. Five high-power fields (×20) were selected and photographed.

### Statistical analysis

Statistical differences between the two groups were tested by Wilcox test. Spearman correlation analysis was used to assess the correlation between gene expression levels and checkpoint-associated genes. Univariate as well as multivariate cox regression analyses were applied to construct nomograms. We used *P* < 0.05 as statistical significance.

## Results

### Prognostic value of IGF2BP3 in HCC patients

In order to acquire a more comprehensive understanding about the role of m6A methylation, ferroptosis and immune modulation in HCC, we selected an m6A “reader” called IGF2BP3 as an example for the next study. First, we divided the LIHC data in the TCGA database into IGF2BP3^high^ and IGF2BP3^low^ group according to the IGF2BP3 median expression levels.

**Figure 1:**
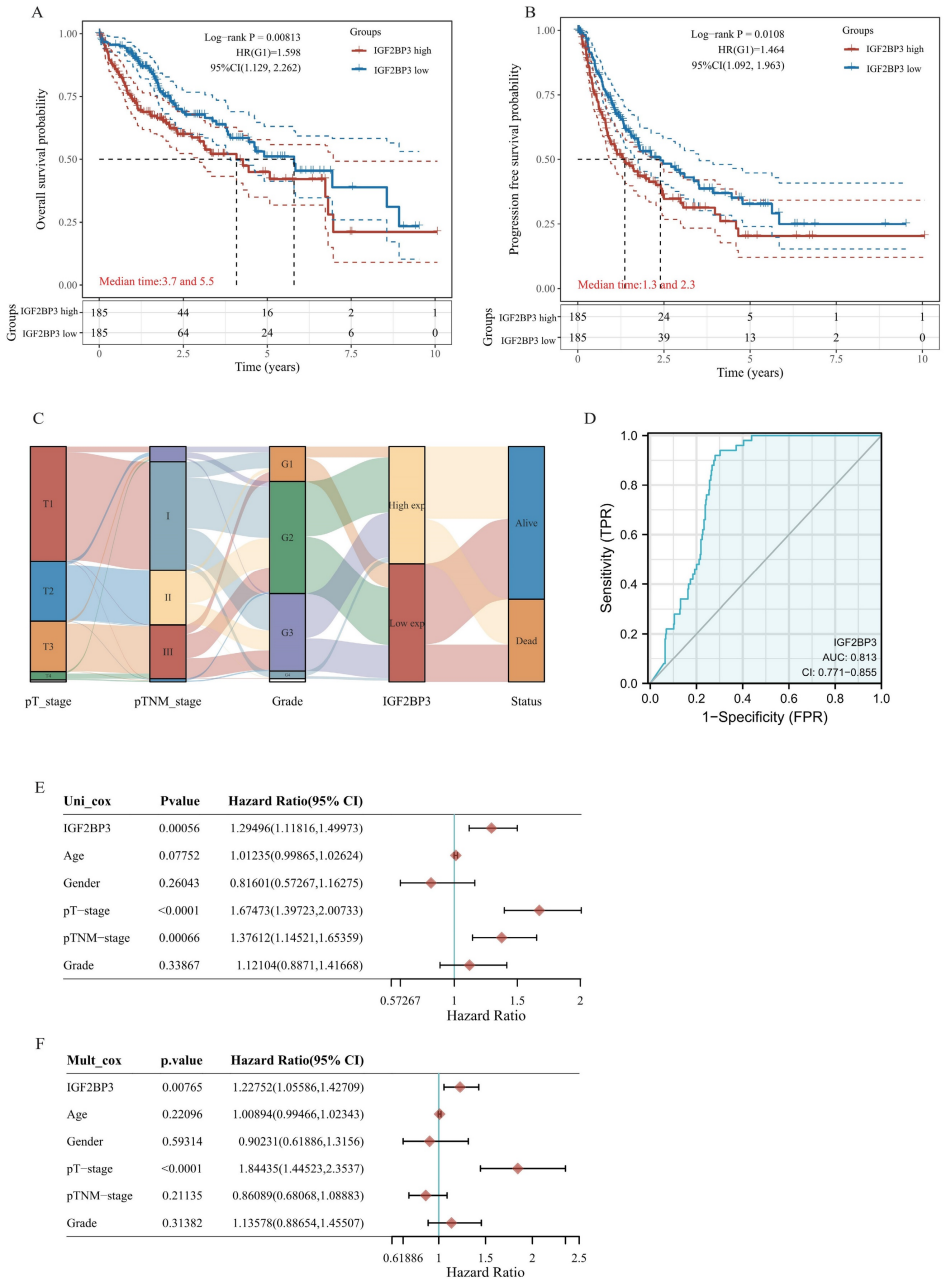
** The up-regulation of IGF2BP3 is correlated with poor clinical outcomes of HCC patients.** (A) The Kaplan-Meier analysis on the overall survival of HCC patients with high and low IGF2BP3 expression level in the TCGA cohort. (B) The Kaplan-Meier analysis on the progression free survival of HCC patients with high and low IGF2BP3 expression level in the TCGA cohort. HR represents the risk ratio of the high expression group relative to the low expression group, and 95% CI represents the HR confidence interval. (C) The Sankey diagram showed the connection degree among the IGF2BP3 expression level and TNM stage, grade and the survival in HCC patients. (D) ROC curves showed the predictive efficiency of the risk signature IGF2BP3 for HCC survival. (E) Univariate (left) and multivariate (right) Cox analysis of IGF2BP3 expression and other factors in HCC patients.

Then, we further evaluated the prognostic value of IGF2BP3^high^ expression group and IGF2BP3^low^ expression group in HCC. Patients with HCC expressing higher IGF2BP3 showed worser overall survival (OS) as well as disease-free survival (DFS) than those expressing lower IGF2BP3 group (**Figure 1A** and **Figure 1B**). In addition, Sanberry plots demonstrated that both the high and low IGF2BP3 expression groups were associated with TNM stage, grade, and survival status of HCC patients (**Figure 1C**). Furthermore, we used ROC curves to assess the prognostic value of IGF2BP3 in HCC. The area under the ROC curve in this study was 0.813, which suggests that IGF2BP3 could be a candidate factor for the diagnosis of HCC patients (**Figure 1D**). Next, we examined the correlation between IGF2BP3 and OS in HCC by Cox analysis. Univariate analysis showed that IGF2BP3 expression (HR = 1.29496, p = 0.00056), pT-stage (HR = 1.67473, p < 0.0001) and TNM stage (HR = 1.37612, p=0.00066) were associated with OS in HCC. However, age (HR = 1.01235, p=0.07752), gender (HR = 0.81601, p=0.26043), and grade (HR = 1.12104, p=0.33867) seems no statistically relationship with OS in HCC (**Figure 1E**). Multivariate analysis demonstrated that IGF2BP3 expression was an independent predictor of progression in HCC (HR = 1.22752, p = 0.00765 (**Figure 1F**).

### Genetic alteration and protein-protein interaction analysis of IGF2BP3 in HCC patients

**Figure 2:**
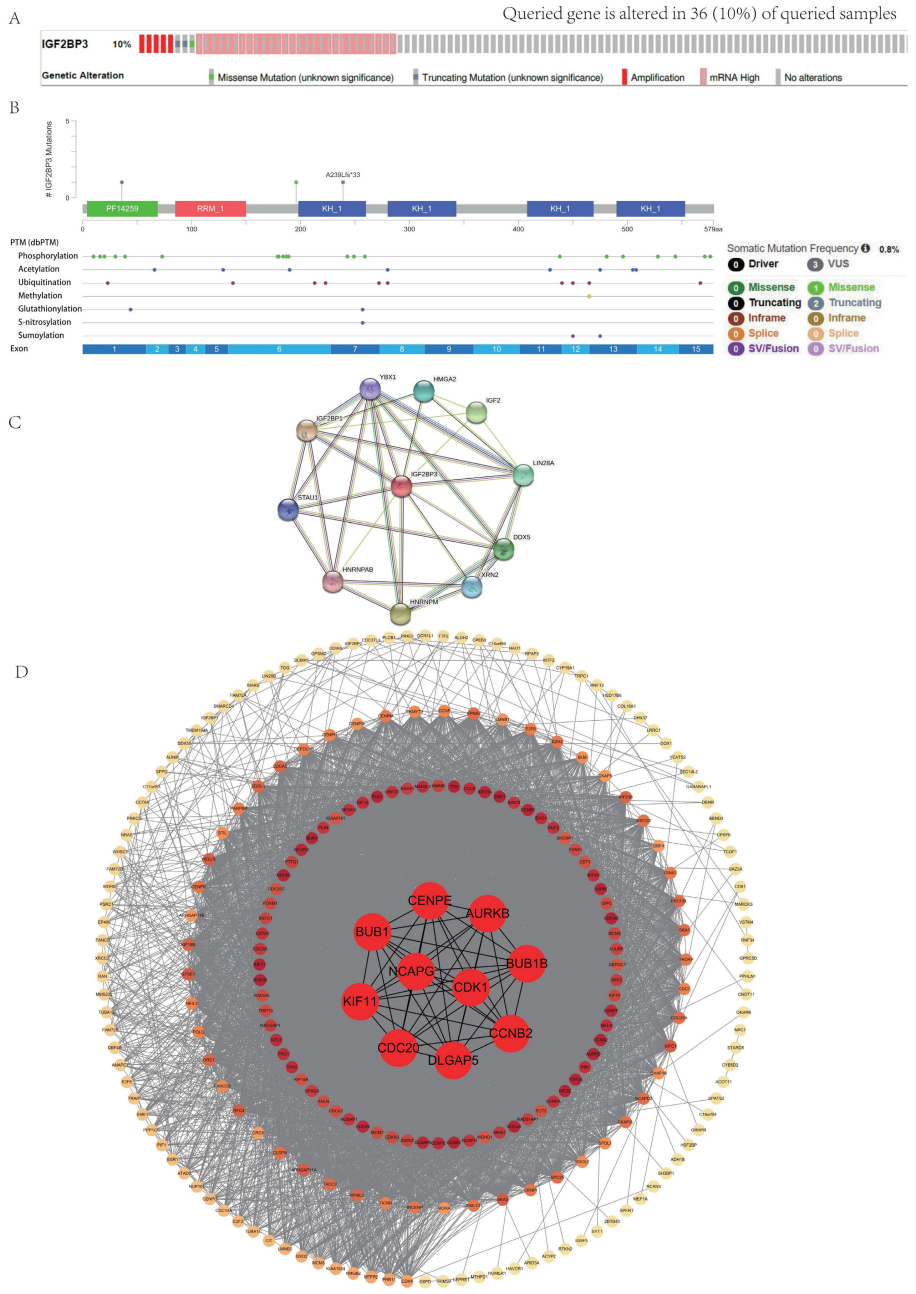
** Mutation and protein-protein interaction analysis of the IGF2BP3 co-expression genes in HCC patients.** (A) Mutation types and mutation frequency of IGF2BP3 in HCC. (B) Mutation diagram of IGF2BP3 in HCC across protein domains, and the protein post-translational modification (PTM) sites. (C) Protein-protein interaction (PPI) network that interacts with IGF2BP3 from cBioPortal database. (D) PPI network used co-expression genes of IGF2BP3 from cBioPortal database. The network was arranged according to the degree of protein interactions, with the innermost circle being top 10 hub genes. Other three circles were arranged from the outermost circle: 0-50, middle circle: 51-100, and inner circle: 101-150.

The cBioPortal database was applied to analyze genetic alterations of IGF2BP3 in HCC. As is depicted in **Fig. 2A**, IGF2BP3 was altered in 36 (10%) of HCC samples, which include “missense mutation”, “truncating mutation”, “amplification”, and “mRNA high”. As is showed, “mRNA high” and “amplification” were the most common types. Next, we investigated the mutational profile of IGF2BP3 that across protein domains in HCC and detected three mutant sites which located between 0 and 579aa. In addition, the protein post-translational modification (PTM), including phosphorylation, acetylation, ubiquitination, methylation, glutathionylation, S-nitrosylation, and sumoylation sites were also showed in **Figure 2B**.

To better understand the function of IGF2BP3, we depicted a PPI network. The result showed that IGF2BP3 has intricate interactions with multiple proteins, including YBX1, IGF2BP1, STAU1, HNRNPAB, HNRNPM, XRN2, DDX5, LIN2BA, IGF2, and HMGA2 (**Figure 2C**).

Furthermore, we obtained the co-expression molecules of IGF2BP3 gene through the cBioPortal database. The network was arranged according to the degree of protein interactions, with the innermost circle being top 10 hub genes, including NCAPG, CDK1, CCNB2, BUB1B, AURKB, CENPE, BUB1, KIF11, CDC20, and DLGAP5. These data were analyzed by cytoHubba plug in Cytoscape software. Other three circles were arranged from the outermost circle: 0-50, middle circle: 51-100, and inner circle: 101-150 (**Figure 2D**).

### Expression and functional enrichment analysis of IGF2BP3 in HCC patients

**Figure 3:**
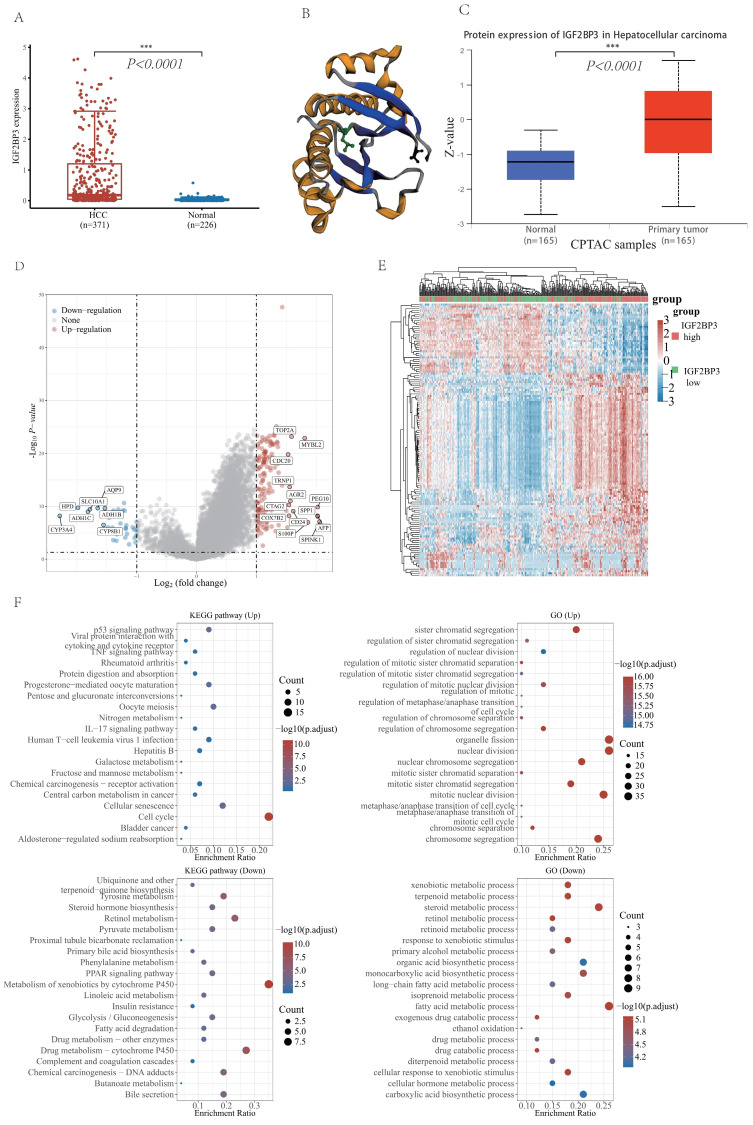
** The differential expression analysis of IGF2BP3 and functional enrichment analysis in HCC and normal tissues.** (A) The mRNA expression level of IGF2BP3 in HCC and normal tissues. (B) The spatial structure of IGF2BP3 protein. (C) The protein expression level of IGF2BP3 in HCC and normal tissues. (D) Volcano plot of differential genes in IGF2BP3^high^ and IGF2BP3^low^ expression groups (divided by the medium expression of IGF2BP3). There are 148 differentially upregulated genes and 34 significantly downregulated genes. (E) Heat map of differential genes in IGF2BP3^high^ and IGF2BP3^low^ expression groups. The top 50 up-regulated genes and top 50 down-regulated genes are displayed here separately. (F) The graphs show KEGG pathway enrichment results and GO term enrichment results for differentially up-regulated genes and down-regulated genes, respectively.

With the analysis of IGF2BP3 mRNA expression level from TCGA LIHC data and GETx database, IGF2BP3 was highly expressed in HCC tissues (**Figure 3A**). To understand the protein expression of IGF2BP3, we visualized the protein structure of IGF2BP3 using the cBioportol database (**Figure 3B**). In addition, the protein expression level of IGF2BP3 was higher in HCC based on the UALCAN database (**Figure 3C**).

To validate the role of IGF2BP3 involved in HCC, we separated HCC patients into two sub-groups based on the expression of IGF2BP3, namely, the IGF2BP3^high^ group and the IGF2BP3^low^ group. The differential expression analysis of these two groups identified 148 up-regulated genes (e.g. TOP2A, MYBL2, CDC20, TRNP1, AGR2, PEG10, BPP1, CTAG2, SPP1, COX7B2, CD24, AFP, S100P, and SPINK1) and 34 down-regulated genes (e.g. CYP3A4, HPD, ADH1C, SLC10A1, AQP9, ADH1B, and CYP8B1). We used volcano plot (**Figure 3D**) and heat maps (**Figure 3E**) for visualizing these results. Next, GO and KEGG enrichment analysis of the differential genes demonstrated that upregulated genes were enriched in cell cycle related pathways. Furthermore, they also enriched in organelle fission and nuclear division function. However, downregulated genes were enriched in the metabolism of xenobiotics by cytochrome P450 related pathways. In addition, they also enriched in fatty acid metabolic process (**Figure 3F**).

### The correlation of IGF2BP3 with different functional pathways (or functional gene sets) in HCC

**Figure 4:**
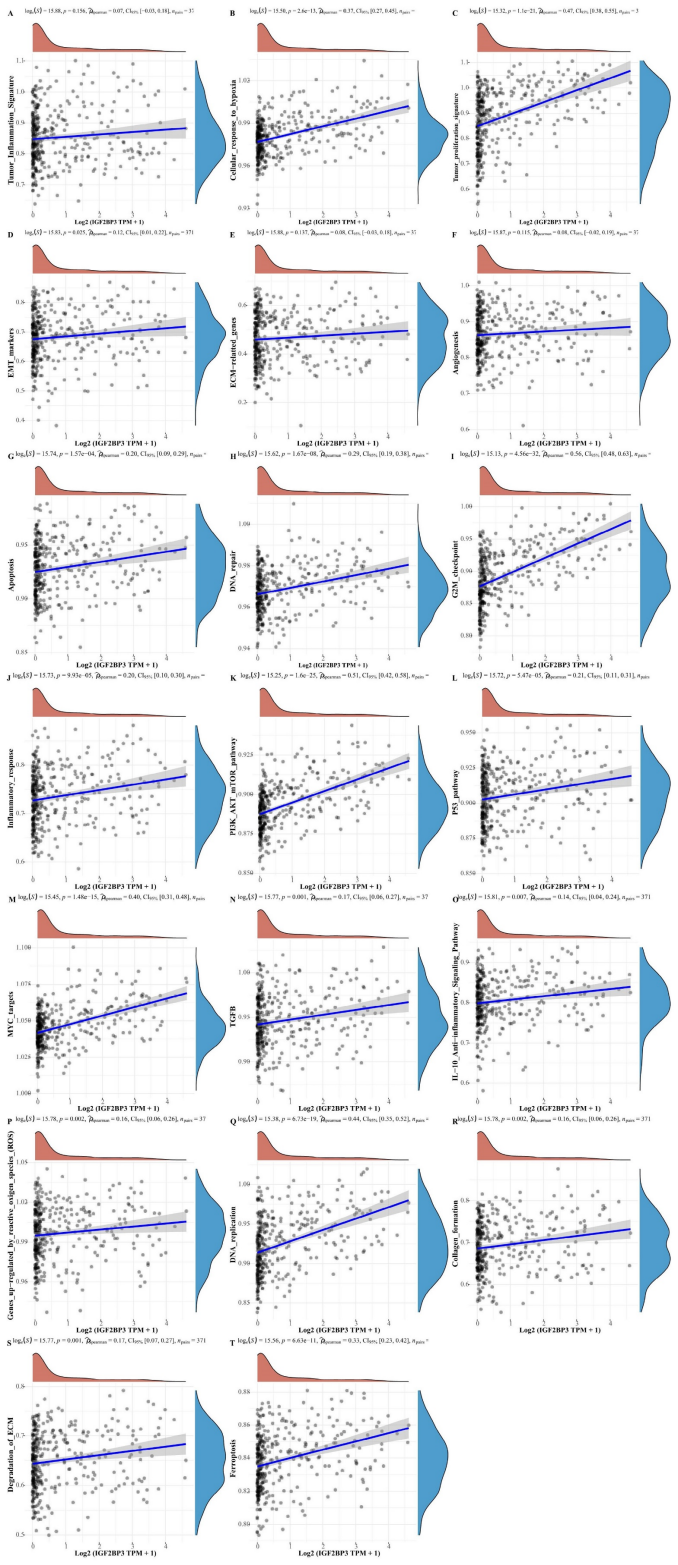
** The correlations between IGF2BP3 and functional pathways (or functional gene sets) in HCC.** The correlations between IGF2BP3 and pathway enrichment score was analyzed with Spearman. The X-axis represents the gene expression, the Y-axis represents the pathway score, and the density curve on the right side represents the distribution trend of the pathway score; the upper side density curve is the distribution trend of the gene expression. And we selected *P*<0.05 as statistical significance.

In addition, to further understand the pathways of IGF2BP3 in HCC, we collected genes in 20 common functional pathways (or functional gene sets) and analyzed the correlation between IGF2BP3 and these pathways. These functional pathways (or functional gene sets) include tumor inflammation signature, tumor proliferation signature, cellular response to hypoxia, EMT markers, angiogenesis, apoptosis, ECM related genes, DNA repair, inflammatory response, G2M checkpoint, PI3K_AKT_mTOR_pathway, MYC targets, P53_pathway, TGFβ, genes upregulated by reactive oxygen species (ROS), IL-10 anti-inflammatory signaling pathway, DNA replication, degradation of ECM, collagen formation, and ferroptosis related pathways. The enrichment scores of each sample on each pathway were calculated sequentially according to the ssGSEA algorithm to obtain the association between samples and pathways. The results demonstrated that IGF2BP3 was involved in all signaling pathways except the tumor inflammation signature, ECM related genes, and angiogenesis related pathways. More importantly, IGF2BP3 was closely associated with the following five signaling pathways (r > 0.4), that were tumor proliferation signature, G2M checkpoint, PI3K_AKT_mTOR_pathway, MYC targets, and DNA repair related pathways (**Figure 4**). Chen X et al. found that in LUAD, overexpression of IGF2BP3 can activate PI3K/AKT signal transduction, while silencing IGF2BP3 reduces the activity of this pathway 32. IGF2BP3 promotes the stability and storage of its target gene MYC mRNA in a m6A-dependent manner under normal and stress conditions, thereby affecting gene expression output and promoting the occurrence and development of cancer, such as neuroblastoma and nasopharyngeal carcinoma 33-35.

### IGF2BP3 participated in ferroptosis and immune modulation processes in HCC patients

**Figure 5:**
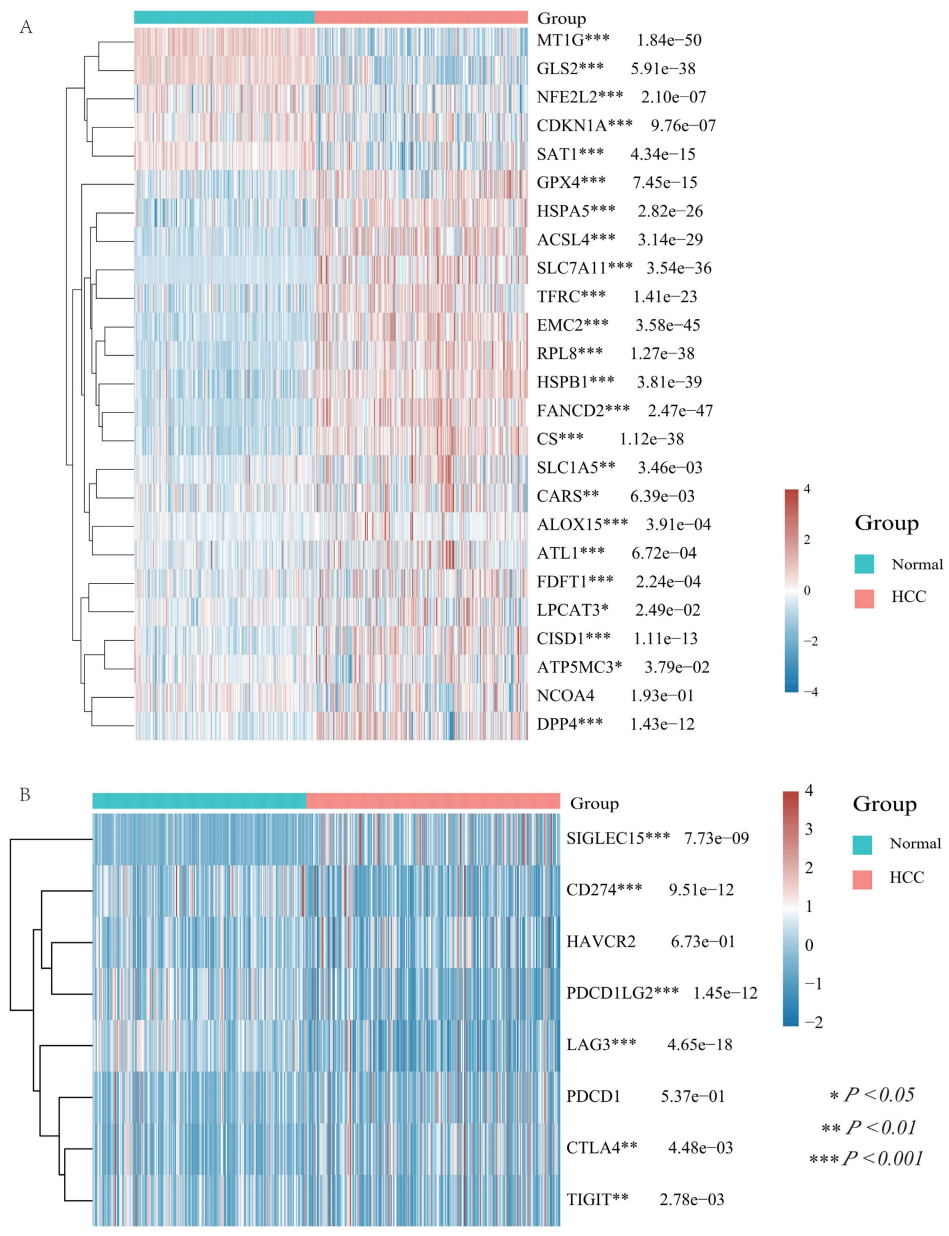
** Differential expression of ferroptosis related molecules and immune checkpoints in HCC and normal tissues.** (A) The heatmap of ferroptosis related genes expression. The different colors represent the trend of gene expression in different samples. (B) The heatmap of immune checkpoints expression. The different colors represent the trend of gene expression in different samples. Red for HCC tissue, blue for normal tissue. The statistical difference of two groups was compared through the Wilcox test, significance difference of three groups was tested with Kruskal Wallis test. *P < 0.05, **P < 0.01, ***P < 0.001.

In recent years, ferroptosis and immune modulation have been demonstrated a critical role in tumorigenesis and progression. Ferroptosis took part in chemoresistance in colorectal cancer (CRC), and studies revealed that adipose-derived exosomes promoted resistance to oxaliplatin by decreasing susceptibility to ferroptosis 36. Tu et al. analyzed the therapeutic benefits of cancer immunotherapy for personalized therapies by studying cancer immunotherapy and single cell resistance 37. To obtain a more comprehensive and complete information, we analyzed 25 ferroptosis related genes (including MT1G, GLS2, NFE2L2, CDKN1A, SAT1, GPX4, HSPA5, ACSL4, SLC7A11, TFRC, EMC2, RPL8, HSPB1, FANCD2, CS, SLC1A5, CARS, ALOX15, ATL1, FDFT1, LPCAT3, CISD1, ATP5MC3, NCOA4, and DPP4) and the differential analysis showed that except for NCOA4, all of these ferroptosis associated genes were significantly differentially expressed in HCC and normal tissues (**Figure 5A**).

In addition, we examined the differential expression of immune checkpoints in HCC and normal tissues with a total of eight immune checkpoints (including SIGLEC15, HAVCR2, CD274, PDCD1LG2, PDCD1, LAG3, CTLA4, and TIGIT) and the results displayed that all immune checkpoints, except HAVCR2 and PDCD1, were differential expressed in HCC and normal tissues (**Figure 5B**).

**Figure 6:**
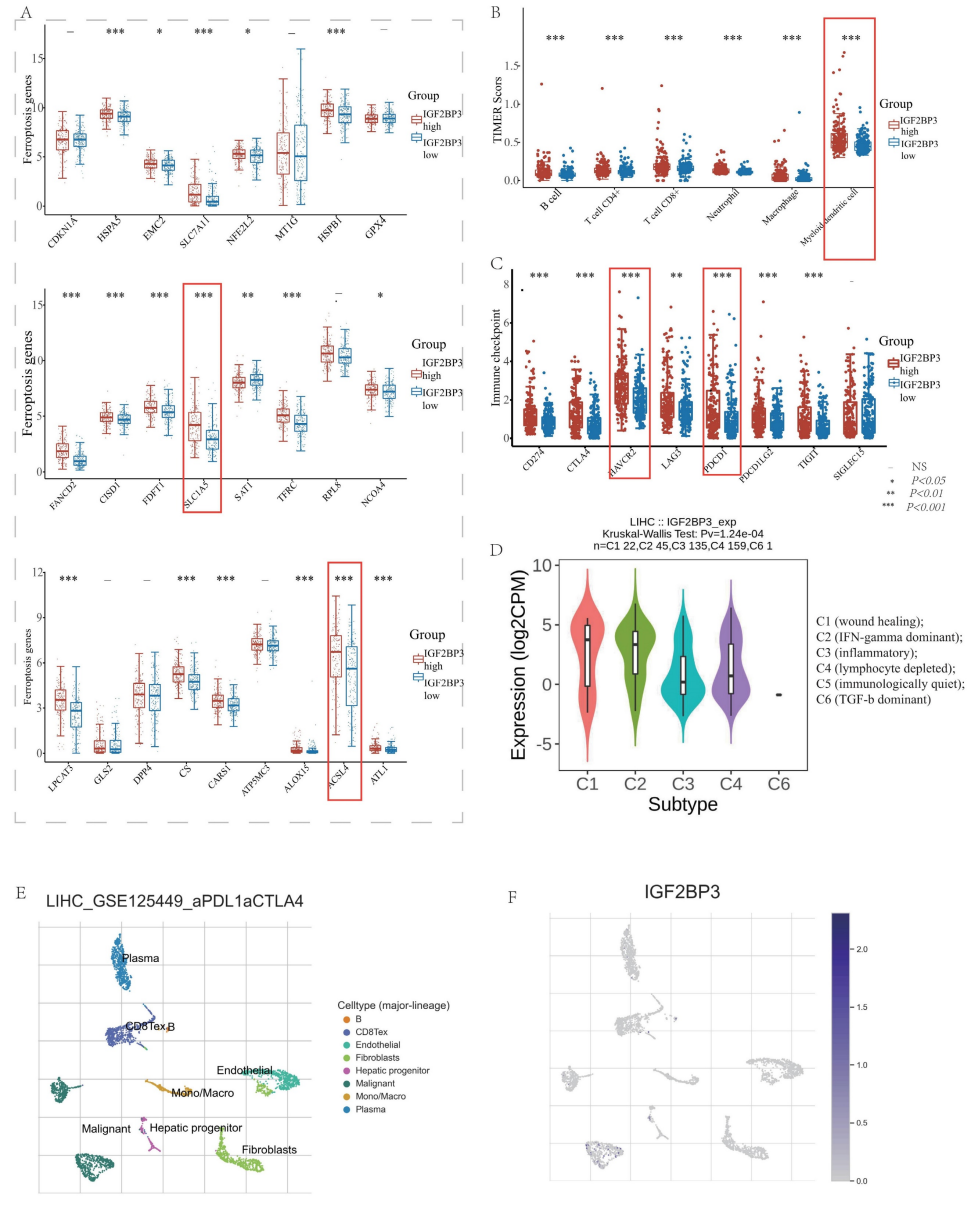
** Differential expression of ferroptosis related molecules and immune modulation in IGF2BP3^high^ and IGF2BP3^low^ expression group.** (A) The expression distribution of ferroptosis related genes in IGF2BP3^high^ and IGF2BP3^low^ expression group. (B) The expression distribution of immune checkpoints in IGF2BP3^high^ and IGF2BP3^low^ expression group. (C) The expression distribution of immune cells in IGF2BP3^high^ and IGF2BP3^low^ expression group. The statistical difference of two groups was compared through the Wilcox test, significance difference of three groups was tested with Kruskal Wallis test. *P < 0.05, **P < 0.01, ***P < 0.001. (D) IGF2BP3 was highly expressed in HCC immune subtypes C1 (wound healing), and C2(IFN-gamma dominant) via TISIDB. (E) The distribution of immune cells after immunotherapy through the TISCH2 database. (F) After PDL1-CTLA4 treatment, IGF2BP3 was mainly expressed in HCC cells, while CD8Tex cells, B cells, hepatocytes and plasma were also a little.

Recent researches pointed out that IGF2BP3 might participate in ferroptosis and immune modulation processes. For instance, Lu Z et al. verified in vivo and in vitro experiments that IGF2BP3 knockdown can significantly enhance SF-induced ferroptosis in HCC cells. IGF2BP3 can stabilize NRF2 mRNA by binding to the m6A site of NRF2 mRNA. The final conclusion was that the IGF2BP3-NRF2 axis could be used as an important mechanism for regulating ferroptosis during SF treatment in HCC 16. CircARID1A acted as a scaffold to promote the interplay of IGF2BP3 and SLC7A5 mRNA, which ultimately increased the stability of SLC7A5 mRNA and thus promoted gastric cancer cell proliferation 38. In addition, METTL3 can promote PD-L1 mRNA stabilization by upregulating PD-L1 expression at the post-transcriptional level in an IGF2BP3-dependent manner, which is important for new and effective therapeutic strategies in tumor immunotherapy 39. However, the bioinformation analysis between IGF2BP3 and ferroptosis as well as immune modulation molecules was absent. So, we analyzed the expression level of ferroptosis molecules, immune checkpoints, and immune cell infiltration in HCC patients with high and low IGF2BP3 expression groups. In detail, we found that 17 ferroptosis related genes (HSPA5, SLC1A5, EMC2, NFE2L2, HSPB1, FANCD2, SLC7A11, CISD1, FDPT1, TFRC, CARS1, NCOA4, LPCAT3, CS, ALOX15, ACSL4 and ATL1) were highly expressed in the IGF2BP3^high^ expression group. In contrast, SAT1 expression was low in the IGF2BP3^high^ expression group. However, the ferroptosis associated regulators, CDKN1A, MT1G, GPX4, RPL8, GLS2, DPP4, and ATP5MC3, displayed no statistically differences in the high and low IGF2BP3 expression groups (**Figure 6A**). In addition, we explored the TIMER database and discovered that immune cell infiltration (e.g. B cell, CD4+ T cell, CD8+ T cell, Neutrophil, Myeloid dendritic cell and Macrophage) was differentially expressed in the high and low IGF2BP3 expression groups (**Figure 6B**). In addition, we found that 7 immune checkpoints (TIGIT, PDCD1LG2, PDCD1, HAVCR2, CTLA4, CD274) were highly expressed in HCC high IGF2BP3 expression group, while SIGLEC15 showed no statistical difference (**Figure 6C**).

We further explored the relationship between IGF2BP3 and immune modulation, and found that IGF2BP3 was highly expressed in HCC immune subtypes C1 (wound healing), and C2(IFN-gamma dominant) via TISIDB (**Figure 6D**). Moreover, we analyzed the distribution of immune cells after immunotherapy (**Figure 6E**) and their expression of IGF2BP3 (**Figure 6F**) through the TISCH2 database. After PDL1-CTLA4 treatment, we found that IGF2BP3 was mainly expressed in HCC cells, while CD8T cells, B cells, hepatocytes and plasma were also a little.

**Figure 7:**
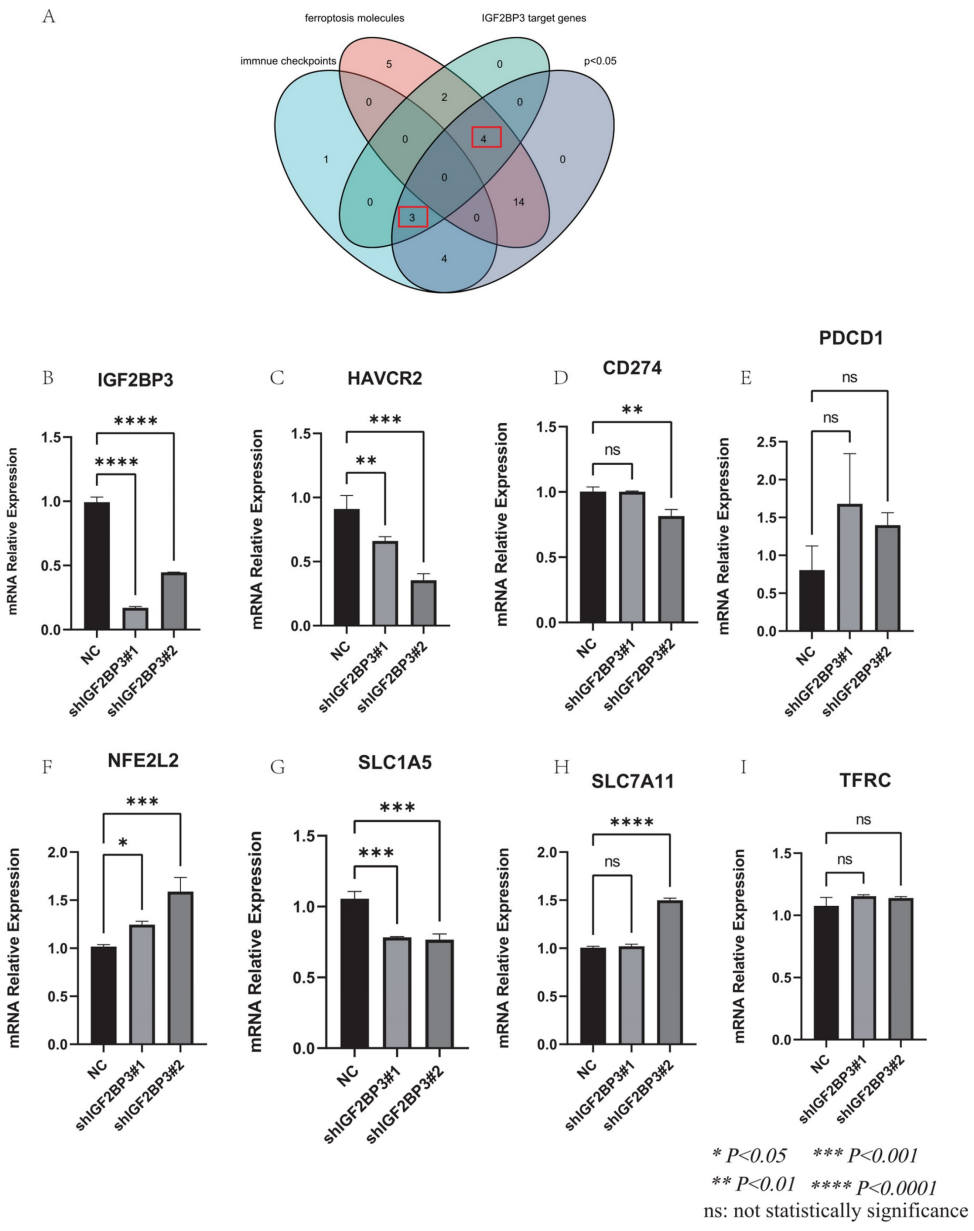
** Validation of the correlation between IGF2BP3 and ferroptosis molecules as well as immune checkpoints.** (A) Venn diagram of ferroptosis molecules, immune checkpoints, potential targets of IGF2BP3, and the differential molecules in IGF2BP3^high^ and IGF2BP3^low^ expression groups. (B) Validation of IGF2BP3 knockdown efficiency by RT-PCR. (C-E) Relationship between ferroptosis molecules and IGF2BP3. (F-I) Relationship between immune checkpoints and IGF2BP3. *P < 0.05, **P < 0.01, ***P < 0.001, ****P < 0.0001, ns: not statistically significance.

In addition, we found the target genes of IGF2BP3 through the M6AREG database (http://m6areg.idrblab.net/). And interestingly, 6 important ferroptosis genes (CDKN1A, SLC7A11, GLS2, SLC1A5, NFE2L2, TFRC) and 3 immune checkpoints (HAVCR2, PDCD1, CD274) were shown to be targets of IGF2BP3, which further indicates that IGF2BP3 was highly potential to perform important functions in ferroptosis and immune modulation (**Supplementary Table 1**). In addition, we also found a number of novel potential targets of IGF2BP3 (**Supplementary Table 2**), which can provide us with more help to study the function of IGF2BP3. Among them, the most promising positive regulatory target of IGF2BP3 is LRP6 and the most promising negative regulatory target is MTA1. However, according to **Figure 6**, we found that the expression of CDKN1A and GLS2 were not statistically different in the IGF2BP3^high^ and IGF2BP3^low^ expression groups. Moreover, through a Venn diagram, three immune checkpoint (PDCD1, HAVCR2, and CD274) and four ferroptosis molecules (SLC7A11, SLC1A5, NFE2L2, and TFRC), which are targets of IGF2BP3 and differentially expressed in the high and low IGF2BP3 expression groups, were identified (**Figure 7A**). Next, we examined the correlation between this four ferroptosis molecules as well as three immune checkpoints (that can serve as potential targets of IGF2BP3) and IGF2BP3 by RT-PCR (**Figure 7B-I**). The results showed that IGF2BP3 presented a negative correlation with the ferroptosis molecule NFE2L2, and a positive correlation with the ferroptosis molecule SLC1A5 as well as the immune checkpoint HAVCR2. However, in our data, no significant correlation has been found between other ferroptosis molecules as well as immune checkpoints and IGF2BP3, suggesting that the regulatory relationship between IGF2BP3 and ferroptosis as well as immune checkpoints may be related to post-transcriptional levels.

### Comprehensive analysis of IGF2BP3 in pan-cancer

**Figure 8:**
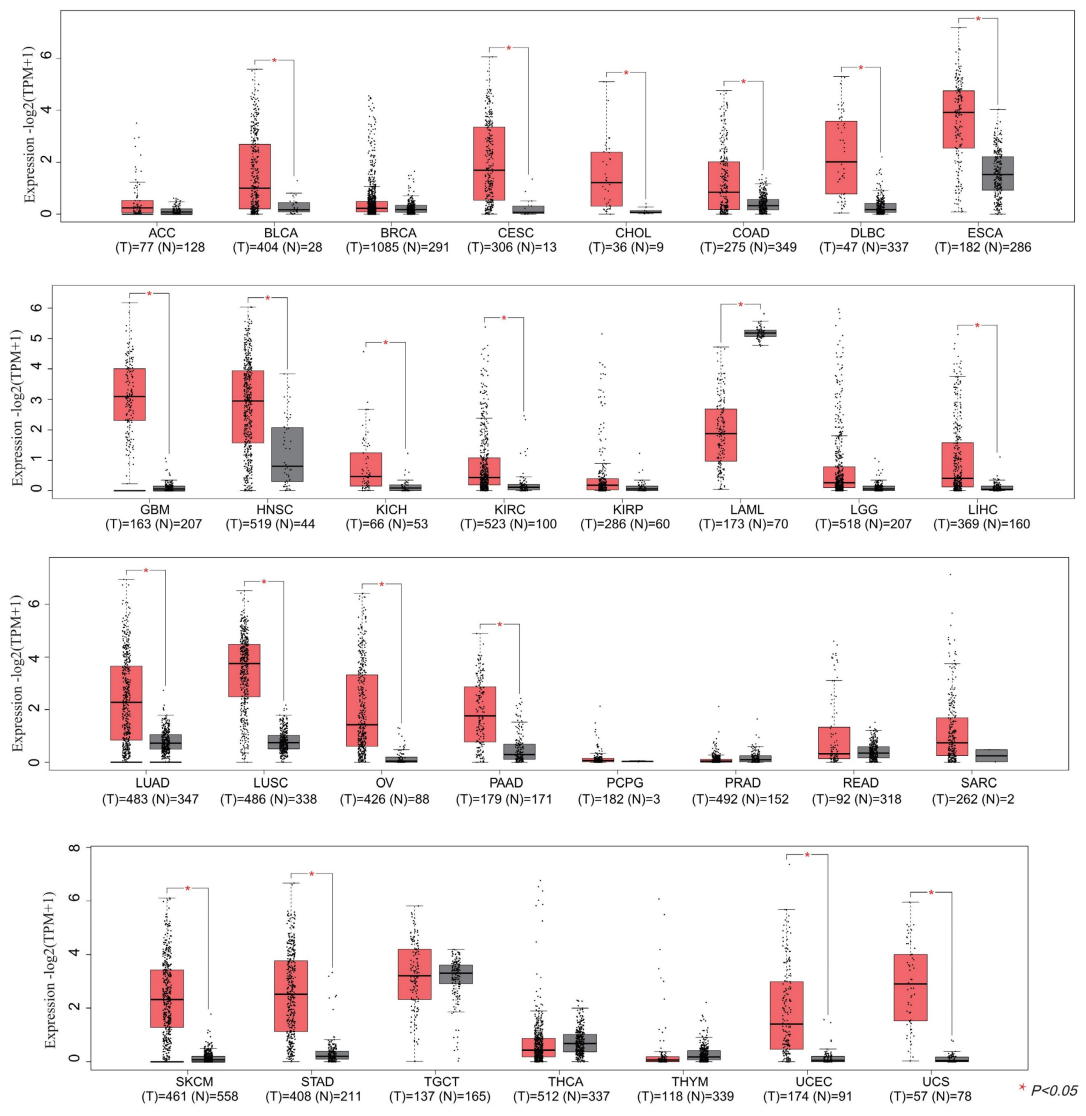
** Pan-cancer analysis of IGF2BP3 expression.** The mRNA expression level of IGF2BP3 in 31 types of tumors. The mRNA levels of IGF2BP3 were found to be upregulated in 20 different tumor tissues, including BLCA, CESE, CHOL, COAD, DLBC, ESCA, GBM, HNSC, KICH, KIRC, LAML, LIHC, LUAD, LUSC, OV, PAAD, SKCM, STAD, UCEC, and UCS.

**Figure 9:**
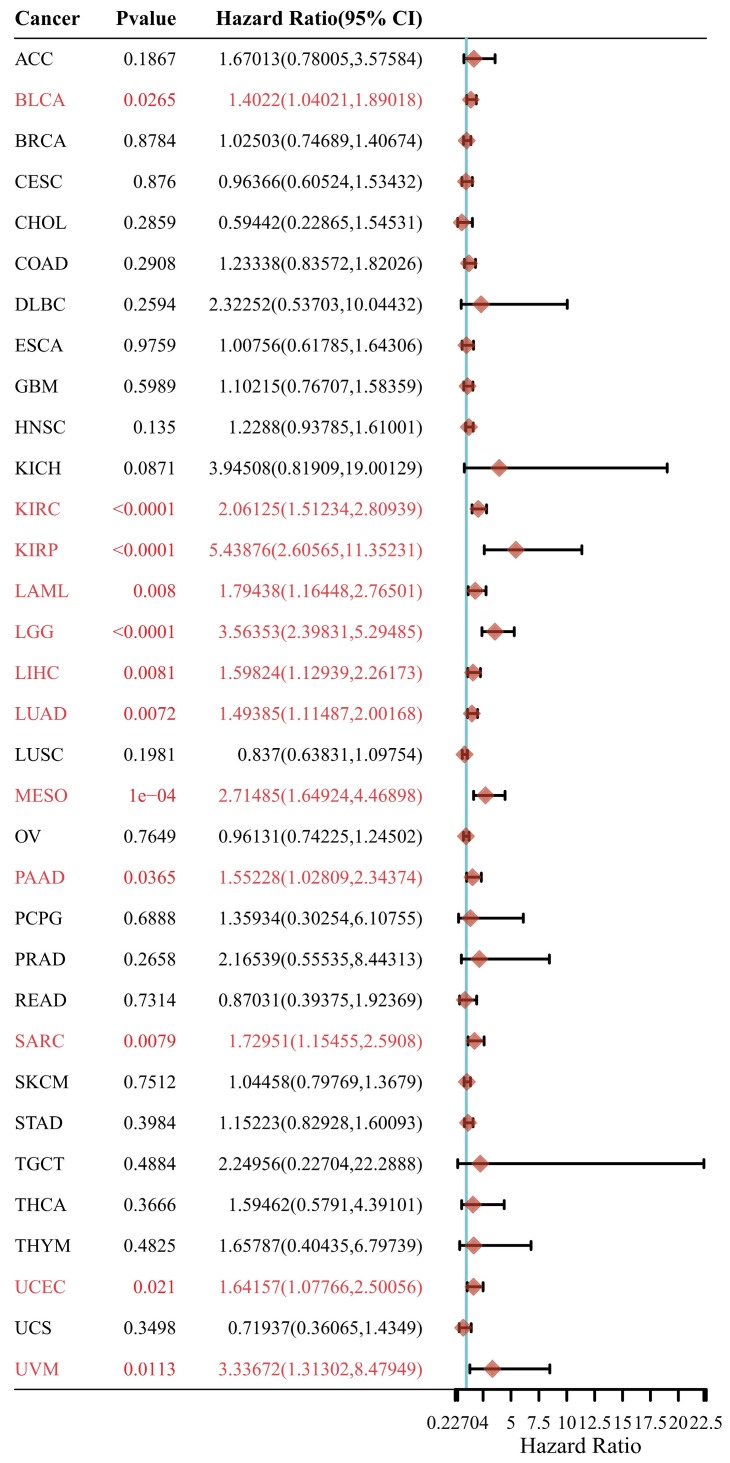
** Prognostic analysis of IGF2BP3 in pan-cancer.** Prognostic value of IGF2BP3 in pan-cancer on overall survival by forest plot. The p value, hazard ratio (HR) and confidence interval were analyzed by univariate cox regression. IGF2BP3 is a risk factor in BLCA, KIRC, KIRP, LAML, LGG, LIHC, LUAD, MESO, PAAD, SARC, UCEC, and UVM.

In this section, we wonder whether IGF2BP3 plays an important role in pan-cancer. Firstly, we analyzed the expression level of IGF2BP3 in virous types of tumors and normal tissues based on TCGA and GTEx databases. In **Figure 8**, IGF2BP3 were upregulated in 19 kinds of tumor, including BLCA, CESE, DLBC, ESCA, CHOL, COAD, GBM, HNSC, LIHC, KICH, KIRC, LUAD, LUSC, STAD, UCEC, OV, PAAD, SKCM, and UCS. The expression level of IGF2BP3 in adjacent tissues of LAML was higher. Next, we evaluated the prognostic value of IGF2BP3 in pan-cancer. Take overall survival as an example, the HR values of IGF2BP3 in 12 cancers (BLCA, KIRC, LIHC, LUAD, KIRP, LAML, PAAD, SARC, LGG, MESO, UCEC, and UVM) were all greater than 1, implying that IGF2BP3 is a risk factor in these cancers (**Figure 9**).

Subsequently, we investigated the role of IGF2BP3 in the immune infiltration and immune checkpoints of the tumor microenvironment in pan-cancer.

**Figure 10:**
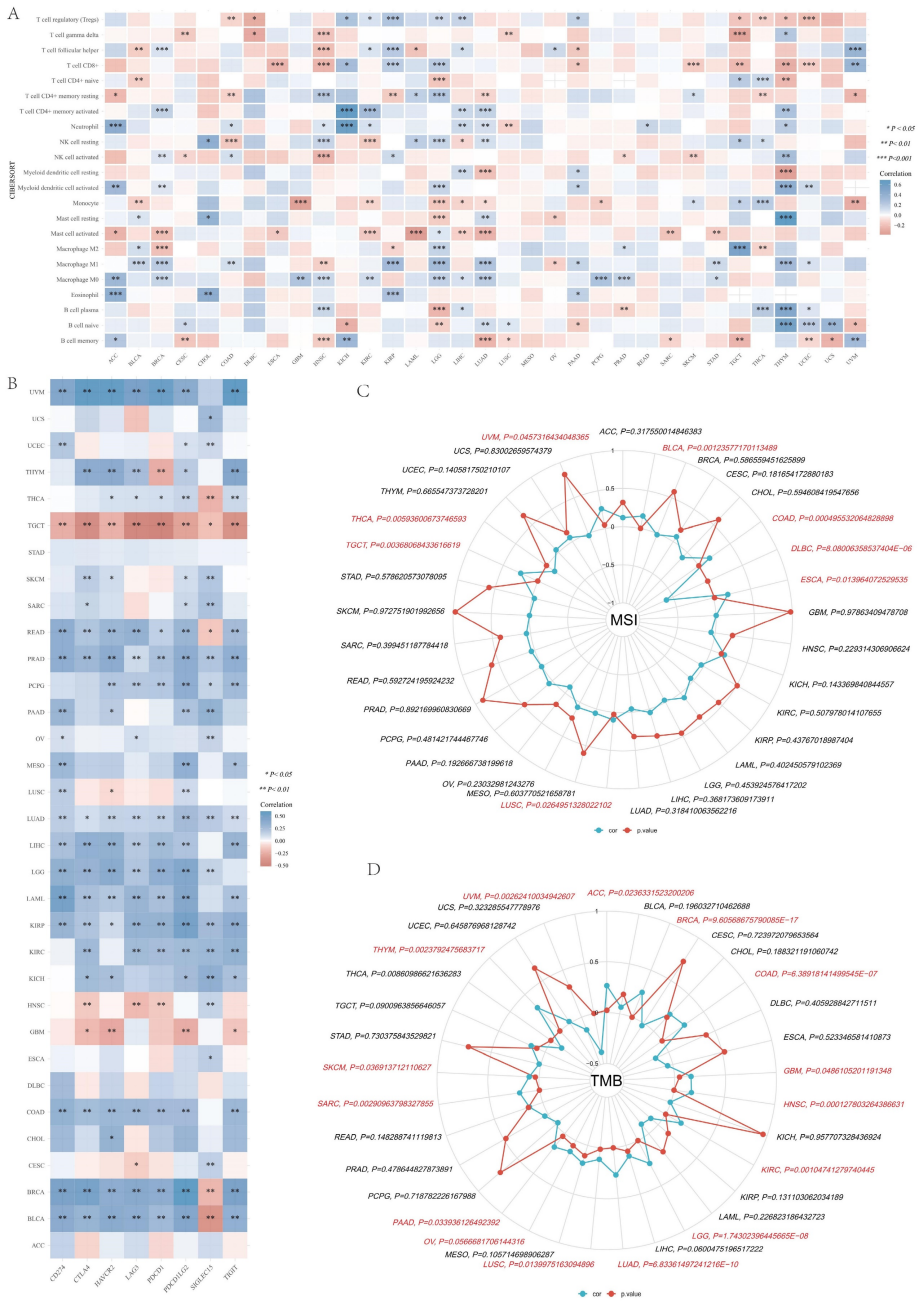
** Pan-cancer correlation analysis of IGF2BP3 and immune cell infiltration, immune checkpoints, TMB and MSI.** (A) Pan-cancer analysis of the correlation between IGF2BP3 expression and immune cell infiltration. (B) Pan-cancer analysis of the correlation between IGF2BP3 expression and immune checkpoints. (C-D) Pan-cancer analysis of the correlation between IGF2BP3 expression and TMB as well as MSI. *P < 0.05, **P < 0.01, ***P < 0.001.

CIBERSORT algorithm was applied to determine the correlation in IGF2BP3 and immune cells, including T cell regulatory (Tregs), T cell CD4+ memory resting, T cell CD4+ memory activated, T cell gamma delta, T cell CD8+, T cell CD4+ naive, T cell follicular helper, Neutrophil, Myeloid dendritic cell resting, Myeloid dendritic cell activated, NK cell resting, NK cell activated, Monocyte, Macrophage M2, Macrophage M1, Macrophage M0, Eosinophil, Mast cell resting, Mast cell activated, B cell plasma, B cell naive, and B cell memory. The results showed that IGF2BP3 maintains a close relationship with these immune cells in pan-cancer tissues, except for MESO. In particular, IGF2BP3 was closely associated with more than 10 immune cells in HNSN, LGG, LIHC, LUAD, and THYM (**Figure 10A**). In addition, the correlation between IGF2BP3 and typical immune checkpoints (including PDCD1, SIGLEC15, CD274, TIGIT, CTLA4, LAG3, HAVCR2 and PDCD1LG2) were explored. The results showed that IGF2BP3 expression levels in most tumors (except STAD, DLBC,) were closely associated with immune checkpoints, especially in TGCT, READ, PRAD, LUAD, KIRP, BRCA and BLCAB (**Figure 10B**). By reviewing the literature, we learned that inhibiting IGF2BP3 in breast cancer cells can enhance anti-tumor immunity through PD-L1-mediated T cell activation, exhaustion, and infiltration 39. In non-small cell lung cancer, IGF2BP3 inhibits CD8 + T cell response by promoting the deubiquitination of PD-L1, thereby promoting tumor immune escape 40.

Furthermore, we found that IGF2BP3 was positively correlated with microsatellite instability (MSI) of ESCA, COAD, BLCA, UVM, TGCT, LUSC, and negatively correlated with MSI of DLBC and THCA (**Figure 10C**). Additionally, IGF2BP3 was positively related to tumor mutational burden (TMB) of LUAD, LGG, KIRC, HNSC, GBM, COAD, BRCA, ACC, THYM, SKCM, SARC, PAAD, OV, and LUSC, while negatively correlated with TMB of UVM (**Figure 10D**).

**Figure 11:**
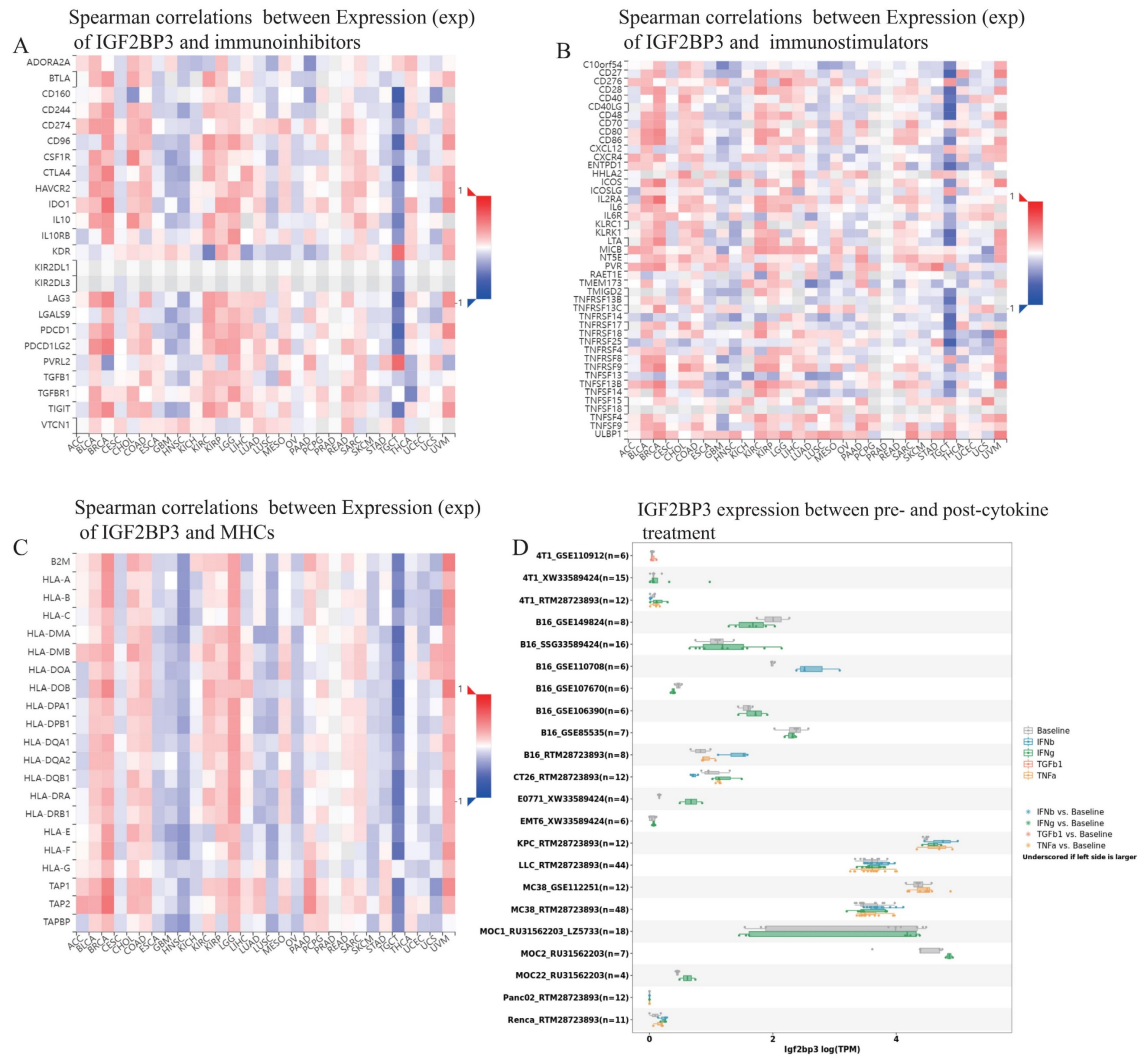
** Pan-cancer correlation analysis of IGF2BP3 and immunoinhibitors, immunostimulators, MHCs, and cytokines.** (A) IGF2BP3 had a positively strong correlation with immunoinhibitors in BLCA, BRCA, KIRC, and UVM. However, they exhibited negatively strong correlation in GBM, HNSN, and TGCT. (B) IGF2BP3 was closely and positively related to immunostimulators in BLCA, BRCA, KIRC, and UVM, while negatively in GBM, HNSN and TGCT. (C) The correlations between IGF2BP3 expression and MHC appeared obviously positive in BLCA, BRCA, LGG, and UVM, while obviously negative in HNSC, LUSC, and TGCT. (D) IGF2BP3 was differentially expressed before and after IFN-gamma treatment, especially in B16 cells, MOC2 cells and E0771 cells. In B16 cells, IGF2BP3 expression was significantly down regulated after IFN-beta treatment.

Additionally, TISIDB database demonstrated that spearman correlations between the expression level of IGF2BP3 and immunoinhibitors, immunostimulators, as well as MHCs among human cancers. IGF2BP3 had a positively strong correlation with immunoinhibitors in BLCA, BRCA, KIRC, and UVM. However, they exhibited negatively strong correlation in GBM, HNSN, and TGCT (**Figure 11A**). Moreover, IGF2BP3 was closely and positively related to immunostimulators in BLCA, BRCA, KIRC, and UVM, while negatively in GBM, HNSN and TGCT (**Figure 11B**). In addition, the correlations between IGF2BP3 expression and MHC were also displayed. They appeared obviously positive correlations in BLCA, BRCA, LGG, and UVM, while obviously negative correlations in HNSC, LUSC, and TGCT (**Figure 11C**). We obtained the expression data of IGF2BP3 before and after cytokine treatment in various of cancer cell lines through TISMO database. IGF2BP3 was differentially expressed before and after IFN-gamma treatment, especially in B16 cells, MOC2 cells and E0771 cells. It is also worth noting that in B16 cells, IGF2BP3 expression was significantly down regulated after IFN-beta treatment (**Fig. 11D**). In conclusion, IGF2BP3 participated in the immune modulation of pan-cancer and may have potential to be a new immunotherapeutic target in the tumor therapy.

### IGF2BP3 enhanced the proliferation, colony formation and invasion abilities of HCC cells

**Figure 12:**
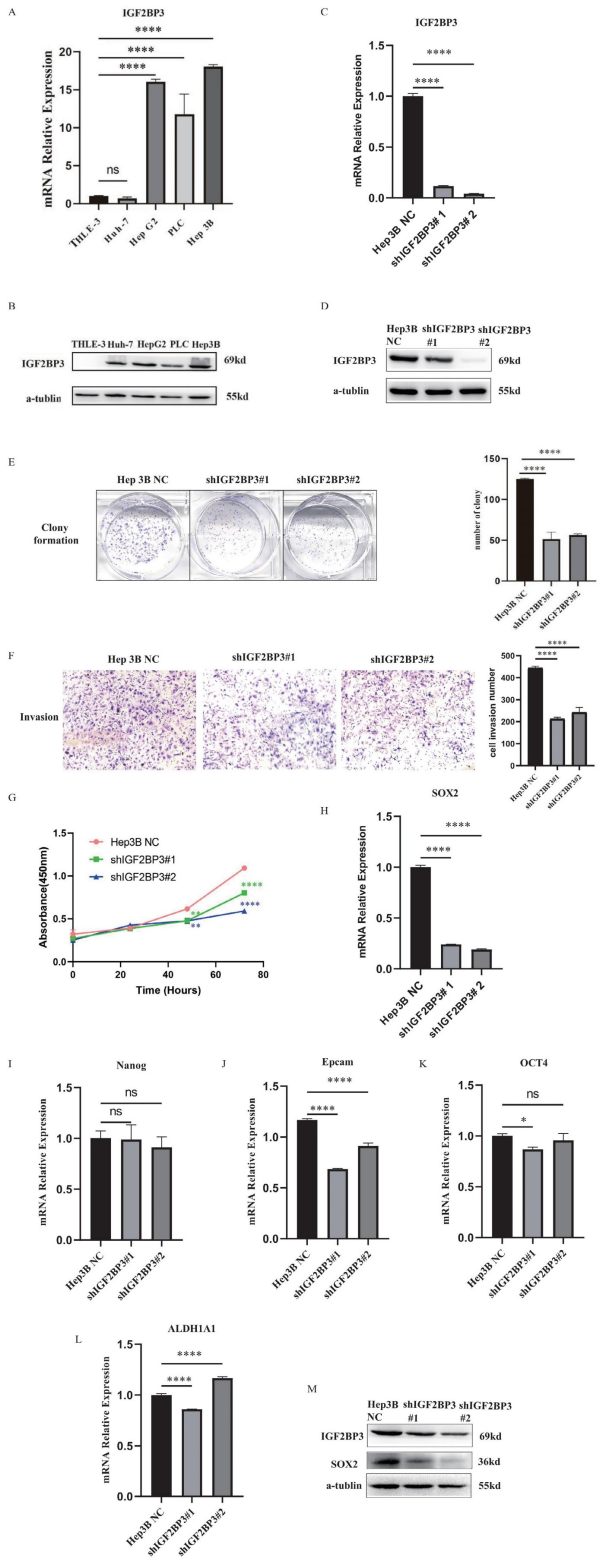
** IGF2BP3 was correlated with cell proliferation, colony formation, invasion and cancer stemness of HCC.** (A) The mRNA expression levels of IGF2BP3 were up-regulated in HCC cell lines. (B) The protein expression levels of IGF2BP3 were up-regulated in HCC cell lines. (C) The knockdown efficiency of IGF2BP3 in Hep3B was detected by RT-PCR. (D) The knockdown efficiency of IGF2BP3 in Hep3B was detected by WB. (E) The colony formation assay of IGF2BP3 KD and NC group in Hep3B cells. (F) The cell invasion assay of IGF2BP3 KD and NC group in Hep3B cells. (G) The cell proliferation assay of IGF2BP3 KD and NC group in Hep3B cells. (H-L) The expression levels of stemness markers including SOX2, Nanog, Epcam, OCT4, and ALDH1A1 in IGF2BP3 KD Hep3B cells were detected by RT-PCR. (M) The expression levels of SOX2 in IGF2BP3 KD Hep3B cells were detected by WB.

The expressions of IGF2BP3 were up-regulated in HCC cell lines (**Fig. 12A and Fig. 12B**). In order to study the biological functions of IGF2BP3 in HCC, we used the strategy of knockdown (KD) of IGF2BP3 gene. IGF2BP3-stable KD cell line was established in Hep3B cells using lentiviral shRNA method and the knockdown efficiency was detected by WB (**Fig. 12C, D**). Compared with the negative control (NC) group, the proliferation and colony formation rate of IGF2BP3 KD Hep3B cells were slower. In addition, the invasion rate of IGF2BP3 KD Hep3B cells was significantly reduced (**Fig. 12E-G**). These results suggested that IGF2BP3 enhanced the proliferation, colony formation and invasion abilities of HCC cells.

### The correlation between IGF2BP3 and HCC stemness markers

In Figure 2D, the top 10 genes with the most significant correlation with IGF2BP3 were NCAPG, CDK1, CCNB2, BUB1B, AURKB, CENPE, BUB1, KIF11, CDC20 and DLGAP5. Through literature search, we found that these key genes are considered to be associated with HCC stemness 41, 42 . It was found that NCAPG could promote the stemness and glycolysis activity of LUAD cells. Further experiments showed that 2-DG (glycolysis inhibitor) could reverse the stimulation effect of NCAPG overexpression on the stemness and glycolysis activity of LUAD cells 43. CDK1 can interact with the stemness marker SOX2 protein and positively regulate the stemness of lung cancer cells 44. In order to further explore the regulatory mechanism of IGF2BP3 on HCC stemness, we detected the expressions of stemness markers in IGF2BP3 KD cells, including SOX2, Nanog, Epcam, OCT4, ALDH1A1, and so on. The results showed that SOX2 was positively correlated with the expression of IGF2BP3 (**Fig. 12H-L**). Next, we further verified the correlation between IGF2BP3 and SOX2 at the protein level by WB (**Fig. 12M**). The results showed that the expression levels of IGF2BP3 and stemness marker SOX2 were positively correlated.

## Discussion

In this research, we mainly conducted a comprehensive analysis of IGF2BP3 in HCC and pan-cancer. Based on survival analysis, COX regression analysis, and ROC curve analysis, we concluded that IGF2BP3 was closely involved in the bad prognosis in HCC patients and was an individual risk element. More importantly, epigenetic regulation was proved a fundamental biological process involved in cancer 45. In this paper, we found that IGF2BP3 genetic and protein mutations were present in HCC patients. And the gene mutations were mainly in the form of gene amplification, while the protein level mutations involved various protein modification sites, including phosphorylation, ubiquitination, acetylation, methylation, glutathionylation, S-nitrosylation, and sumoylation sites. This indicates that epigenetic modifications may also conduct an important function in HCC. Subsequently, we constructed a protein-protein interaction network centered on IGF2BP3 protein, and analyzed it to obtain the top 10 hub genes, including NCAPG, CDK1, CCNB2, BUB1B, AURKB, CENPE, BUB1, KIF11, CDC20, and DLGAP5. Studies demonstrated that these hub genes are thought to be associated with cancer stemness 42, 46, and cancer immunity 47, 48. Altogether, IGF2BP3 is an extremely important molecule involved in a very complex tumorigenesis process, which still has large research potential in the future.

Ras-selective oncogenic small molecule, the erastin, provokes a new form of iron-dependent non-apoptotic cell death known as ferroptosis. The erastin inhibits cyanine/glutamate antitoxicants (System X(C) (-)) that inhibit cystine uptake, creating gaps in the cell's antioxidant defenses and eventually resulting in iron-dependent oxidative death. This kind of non-apoptotic form of cell death might promote the specific clearance of certain cancer cells, or become activated in certain specific states of pathology 49. Our results revealed that IGF2BP3 might participate in ferroptosis processes through regulating ferroptosis molecules (especially ACSL4, SLC1A5). This might be a novel way for IGF2BP3 to regulate HCC, as ferroptosis has been shown to play an important function in tumors. Research showed that SHARPIN promoted cholangiocarcinoma cell proliferation and inhibited ferroptosis via p53/SLC7A11/GPX4 signaling 50. In addition, ferroptosis related elements was identified as potential predictive markers for a variety of cancers, such as liver cancer 51 and breast cancer 52, 53.

Immunotherapies are now targeting more than just the CTLA-4 or PD-1 related pathways. A lot of other immunomodulators, including both irritants and inhibitors, have also been explored for cancer immunotherapy as possible targets, and these immunotherapies may become important changes in the treatment of cancer 54. For instance, discoidin domain receptor 1 (DDR1) is a passive immune modulator of colorectal cancer. It participates in CD4+ and CD8+ T cell hypo-infiltration through inhibiting the synthesis of IL-18, thereby promoting colorectal tumor growth in vivo 55. In addition, the study found that CD8 score might be a potential index to identify immune checkpoint inhibitors. And that HCC patients with high CD8 scores exhibit better tumor prognosis, which may be associated with immune-mediated tumor cell attack 56. Importantly, this study declaimed that IGF2BP3 might participate in immune modulation processes, by interacting with immune infiltration (especially myeloid dendritic cell) and immune checkpoints (PDCD1 and HAVCR2) in HCC patients. We also revealed the distribution of IGF2BP3 in the tumor microenvironment, some of IGF2BP3 existed in CD8Tex cells, which provided some new evidence for immune modulation. Altogether, our study provided integrated potential mechanisms of IGF2BP3 in the immune regulation of HCC, which is worthy of further exploration.

In addition, we validated the correlation of IGF2BP3 with ferroptosis molecules (NFE2L2, SLC1A5) and immune checkpoint (HAVCR2) through experiments. However, more extensive experiments are needed to validate the findings of this study.

## Conclusions

In conclusion, we used a bioinformatics approach to comprehensively analyze the significance of IGF2BP3 in HCC through analyzing its expression, mutation, prognosis, functional enrichment, and the correlation with ferroptosis, stemness as well as immune modulation in HCC. This helps us to explain the occurrence and progression of HCC from multiple perspectives and thus find more effective therapeutic approaches. In addition, we also analyzed IGF2BP3 expression, prognosis and immune modulation in pan-cancer, revealing the prognostic value of IGF2BP3 in a variety of tumors. Finally, we verified the biological functions of IGF2BP3 in HCC through various experiments. According to our results, IGF2BP3 can be used as a biomarker for clinical detection of HCC, which is of positive significance for the treatment and prognosis of HCC patients.

## Supplementary Material

Supplementary tables.

## Abbreviations

ACC: Adrenocortical carcinoma
BLCA: Bladder urothelial carcinoma
BRCA: Breast invasive carcinoma
CAR-T: chimeric antigen receptor engineered T-cell immunotherapy
CESC: Cervical squamous cell carcinoma and endocervical adenocarcinoma
CHOL: Cholangiocarcinoma
CI: confidence intervals
COAD: Colon adenocarcinoma
CRC: colorectal cancer
DDR1: discoidin domain receptor 1
DFS: disease-free survival
DLBC: Lymphoid neoplasm diffuse large B-cell lymphoma
ESCA: Esophageal carcinoma
GBM: Glioblastoma
HCC: hepatocellular carcinoma
HNSCC: Head and neck squamous cell carcinomas
HPA: Human Protein Atlas
ICGC: International Cancer Genome Consortium
KICH: Kidney chromophobe
KIRC: Kidney renal clear cell carcinoma
KIRP: Kidney renal papillary cell carcinoma
LAML: Acute myeloid leukemia
LGG: Low grade glioma
LIHC: Liver hepatocellular carcinoma
LUAD: Lung adenocarcinoma
LUSC: Lung squamous cell carcinoma
MSI: microsatellite instability
OS: overall survival
OV: Ovarian Cancer
PAAD: Pancreatic adenocarcinoma
PCPG: Pheochromocytoma, and paraganglioma
PPI: Protein-Protein interactions
PRAD: Prostate adenocarcinoma
PTM: post-translational modification
READ: Rectum adenocarcinoma
ROS: reactive oxygen species
SARC: Sarcoma
SKCM: Skin cutaneous melanoma
STAD: Stomach adenocarcinoma
TACE: trans-arterial chemoembolization
TCGA: the Cancer Genome Atlas
TGCT: Testicular germ cell tumors
THCA: Thyroid carcinoma
THYM: Thymoma
TMB: tumor mutational burden
TNBC: triple negative breast cancer
Tregs: T cell regulatory
UCEC: Uterine corpus endometrial carcinoma
UCS: Uterine carcinosarcoma
