# Supplementary material for: Comprehensive analysis of IGF2BP3 with expression features, prognosis, immune modulation and stemness in hepatocellular carcinoma and pan-cancer
Source: J Cancer. 2024 Mar 25;15(9):2845–65. doi: 10.7150/jca.92768 (PMC10988304; doi:10.7150/jca.92768)
Supplement: Supplementary file 1 — Supplementary tables. [file jcav15p2845s1.zip › Supplementary tables.pdf]

Supplementary table 1. General information of IGF2BP3 target genes

Target_ID	Gene ID	Gene_name	Target Type	Chromosomal location
M6ATAR00318	10128	LRPPRC	Protein coding	2p21
M6ATAR00587	6194	S6	Protein coding	9p22.1
M6ATAR00160	5226	PGD	Protein coding	1p36.22
M6ATAR00334	4313	MMP2	Protein coding	16q12.2
M6ATAR00546	56999	ADAMTS9	Protein coding	3p14.1
M6ATAR00701	259266	ASPM	Protein coding	1q31.3
M6ATAR00685	.	AC008	LncRNA	.
M6ATAR00794	105373748	AC010894.3	LncRNA	2q31.1
M6ATAR00101	100874306	ACAP2-IT1	LncRNA	3q29
M6ATAR00104	107985353	ACBD3-AS1	LncRNA	1q42.12
M6ATAR00168	31	ACACA	Protein coding	17q12
M6ATAR00170	59	ACTA2	Protein coding	10q23.31
M6ATAR00750	324	APC	Protein coding	5q22.2
M6ATAR00275	84681	HINT2	Protein coding	9p13.3
M6ATAR00303	205	AK4	Protein coding	1p31.3
M6ATAR00539	221079	ARL5B	Protein coding	10p12.31
M6ATAR00172	27125	AFF4	Protein coding	5q31.1
M6ATAR00177	216	ALDH1A1	Protein coding	9q21.13
M6ATAR00174	1645	AKR1C1	Protein coding	10p15.1
M6ATAR00520	2023	ENO1	Protein coding	1p36.23
M6ATAR00163	5562	PRKAA1	Protein coding	5p13.1
M6ATAR00479	.	AR	Protein coding	.
M6ATAR00644	7010	TEK	Protein coding	9p21.2
M6ATAR00182	51676	ASB2	Protein coding	14q32.12
M6ATAR00660	10551	AGR2	Protein coding	7p21.1
M6ATAR00582	348	APOE	Protein coding	19q13.32
M6ATAR00195	581	BAX	Protein coding	19q13.33
M6ATAR00196	596	BCL2	Protein coding	18q21.33
M6ATAR00083	103021294	ABALON	LncRNA	20q11.21
M6ATAR00533	22985	ACIN1	Protein coding	14q11.2
M6ATAR00527	91947	ARRDC4	Protein coding	15q26.2
M6ATAR00729	1615	DARS	Protein coding	2q21.3
M6ATAR00183	29028	ATAD2	Protein coding	8q24.13
M6ATAR00184	55210	ATAD3A	Protein coding	1p36.33
M6ATAR00676	89845	ABCC10	Protein coding	6p21.1
M6ATAR00165	10060	ABCC9	Protein coding	12p12.1
M6ATAR00166	215	ABCD1	Protein coding	Xq28
M6ATAR00169	47	ACLY	Protein coding	17q21.2
M6ATAR00226	1654	DDX3X	Protein coding	Xp11.4
M6ATAR00323	5243	ABCB1	Protein coding	7q21.12
M6ATAR00193	9212	AURKB	Protein coding	17p13.1
M6ATAR00189	9474	ATG5	Protein coding	6q21
M6ATAR00191	60673	ATG101	Protein coding	12q13.13
M6ATAR00161	55054	ATG16L1	Protein coding	2q37.1
M6ATAR00187	23130	ATG2A	Protein coding	11q13.1
M6ATAR00594	8312	AXIN1	Protein coding	16p13.3
M6ATAR00741	116071	BATF2	Protein coding	11q13.1
M6ATAR00198	7009	TMBIM6	Protein coding	12q13.12

M6ATAR00201	664	BNIP3	Protein coding	10q26.3
M6ATAR00556	90427	BMF	Protein coding	15q15.1
M6ATAR00194	22863	ATG14	Protein coding	14q22.3
M6ATAR00197	8678	BECN1	Protein coding	17q21.31
M6ATAR00386	9711	RUBCN	Protein coding	3q29
M6ATAR00614	56998	CTNNBIP1	Protein coding	1p36.22
M6ATAR00756	25825	BACE2	Protein coding	21q22.2-q22.3
M6ATAR00200	650	BMP2	Protein coding	20p12.3
M6ATAR00199	406	ARNTL	Protein coding	11p15.3
M6ATAR00707	672	BRCA1	Protein coding	17q21.31
M6ATAR00472	.	ABCG2	Protein coding	.
M6ATAR00145	253962	CACNA1G-AS1	LncRNA	17q21.33
M6ATAR00696	999	CDH1	Protein coding	16q22.1
M6ATAR00202	1000	CDH2	Protein coding	18q12.1
M6ATAR00309	10645	CAMKK2	Protein coding	12q24.31
M6ATAR00087	101805492	CASC9	LncRNA	8q21.13
M6ATAR00220	1375	CPT1B	Protein coding	22q13.33
M6ATAR00221	1459	CSNK2A2	Protein coding	16q21
M6ATAR00203	836	CASP3	Protein coding	4q35.1
M6ATAR00204	837	CASP4	Protein coding	11q22.3
M6ATAR00222	1499	CTNNB1	Protein coding	3p22.1
M6ATAR00679	857	CAV1	Protein coding	7q31.2
M6ATAR00747	.	CBSLR	LncRNA	.
M6ATAR00215	1050	CEBPA	Protein coding	19q13.11
M6ATAR00652	29883	CNOT7	Protein coding	8p22
M6ATAR00574	960	CD44	Protein coding	11p13
M6ATAR00086	103611090	CDR1-AS	LncRNA	Xq27.1
M6ATAR00216	63924	CIDEC	Protein coding	3p25.3
M6ATAR00356	7157	TP53	Protein coding	17p13.1
M6ATAR00699	64105	CENPK	Protein coding	5q12.3
M6ATAR00728	25932	CLIC4	Protein coding	1p36.11
M6ATAR00759	57332	CBX8	Protein coding	17q25.3
M6ATAR00006	.	Circ_104075	circRNA	.
M6ATAR00036	.	Circ_1662	circRNA	.
M6ATAR00575	.	Circ_ASK1	circRNA	.
M6ATAR00517	.	Circ_DLC1	circRNA	.
M6ATAR00008	.	Circ_E7	circRNA	.
M6ATAR00038	.	Circ_GFR-Alpha-1	circRNA	.
M6ATAR00528	.	Circ_IGF2BP3	circRNA	.
M6ATAR00586	.	Circ_MAP3K4	circRNA	.
M6ATAR00616	.	Circ_ORC5	circRNA	.
M6ATAR00548	.	Circ_PTPRA	circRNA	.
M6ATAR00016	.	Circ_SLC7A5	circRNA	.
M6ATAR00053	.	Circ_YTHDC2	circRNA	.
M6ATAR00459	9575	CLOCK	Protein coding	.
M6ATAR00757	79365	BHLHE41	Protein coding	12p12.1
M6ATAR00597	1281	COL3A1	Protein coding	2q32.2
M6ATAR00333	4322	MMP13	Protein coding	11q22.2
M6ATAR00092	100507056	CCAT1	LncRNA	8q24.21
M6ATAR00091	101805488	CCAT2	LncRNA	8q24.21

M6ATAR00346	4708	NDUFB2	Protein coding	7q34
M6ATAR00715	4846	eNOS	Protein coding	7q36.1
M6ATAR00219	57699	CPNE5	Protein coding	6p21.2
M6ATAR00051	.	CTD-3184A7.4	LncRNA	.
M6ATAR00569	64866	CDCP1	Protein coding	3p21.31
M6ATAR00223	7852	CXCR4	Protein coding	2q22.1
M6ATAR00523	3627	Cxcl10	Protein coding	4q21.1
M6ATAR00516	6373	CXCL11	Protein coding	4q21.1
M6ATAR00522	4283	Cxcl9	Protein coding	4q21.1
M6ATAR00185	468	ATF4	Protein coding	22q13.1
M6ATAR00755	467	ATF3	Protein coding	1q32.3
M6ATAR00205	890	CCNA2	Protein coding	4q27
M6ATAR00209	983	CDK1	Protein coding	10q21.2
M6ATAR00210	1017	CDK2	Protein coding	12q13.2
M6ATAR00211	1019	CDK4	Protein coding	12q14.1
M6ATAR00212	1021	CDK6	Protein coding	7q21.2
M6ATAR00213	1026	CDKN1A	Protein coding	6p21.2
M6ATAR00214	1027	CDKN1B	Protein coding	12p13.1
M6ATAR00500	1029	CDKN2A	Protein coding	9p21.3
M6ATAR00748	875	CBS	Protein coding	21q22.3
M6ATAR00484	55083	SLC7A11	Protein coding	.
M6ATAR00217	1545	CYP1B1	Protein coding	2p22.2
M6ATAR00218	1558	CYP2C8	Protein coding	10q23.33
M6ATAR00765	10912	GADD45-Gamma	Protein coding	9q22.2
M6ATAR00693	51339	DACT1	Protein coding	14q23.1
M6ATAR00711	138948	Lnc_DBH-AS1	LncRNA	9q34.2
M6ATAR00622	23604	DAPK2	Protein coding	15q22.31
M6ATAR00690	1613	DAPK3	Protein coding	19p13.3
M6ATAR00151	8847	DLEU2	LncRNA	13q14.2
M6ATAR00231	1823	DSC1	Protein coding	18q12.1
M6ATAR00230	4733	DRG1	Protein coding	22q12.2
M6ATAR00643	51655	RASD1	Protein coding	17p11.2
M6ATAR00227	84649	DGAT2	Protein coding	11q13.5
M6ATAR00627	100505658	DIAPH1-AS1	LncRNA	5q31.3
M6ATAR00721	22943	DKK1	Protein coding	10q21.1
M6ATAR00122	649446	DLGAP1-AS1	LncRNA	18p11.31
M6ATAR00027	.	DMDRMR	LncRNA	.
M6ATAR00658	1789	DNMT3B	Protein coding	20q11.21
M6ATAR00681	1643	DDB2	Protein coding	11p11.2
M6ATAR00470	9575	DDIT3	Protein coding	.
M6ATAR00225	54541	DDIT4	Protein coding	10q22.1
M6ATAR00246	2067	ERCC1	Protein coding	19q13.32
M6ATAR00481	26574	MSH2	Protein coding	.
M6ATAR00482	2956	MSH6	Protein coding	.
M6ATAR00371	51426	POLK	Protein coding	5q13.3
M6ATAR00713	5888	RAD51	Protein coding	15q15.1
M6ATAR00499	4173	MCM4	Protein coding	8q11.21
M6ATAR00321	4174	MCM5	Protein coding	22q12.3
M6ATAR00322	4175	MCM6	Protein coding	2q21.3
M6ATAR00158	4350	MPG	Protein coding	16p13.3

M6ATAR00285	3398	ID2	Protein coding	2p25.1
M6ATAR00463	26574	MRE11	Protein coding	.
M6ATAR00232	1844	DUSP2	Protein coding	2q11.2
M6ATAR00740	1847	DUSP5	Protein coding	10q25.2
M6ATAR00233	1848	DUSP6	Protein coding	12q21.33
M6ATAR00238	1959	EGR2	Protein coding	10q21.3
M6ATAR00382	5903	RANBP2	Protein coding	2q13
M6ATAR00502	9810	RNF40	Protein coding	16p11.2
M6ATAR00270	79872	CBLL1	Protein coding	7q22.3
M6ATAR00579	4193	Mdm2	Protein coding	12q15
M6ATAR00347	23327	NEDD4L	Protein coding	18q21.31
M6ATAR00392	6477	SIAH1	Protein coding	16q12.1
M6ATAR00398	57154	SMURF1	Protein coding	7q22.1
M6ATAR00466	5395	TRIM11	Protein coding	.
M6ATAR00550	81786	TRIM7	Protein coding	5q35.3
M6ATAR00159	1978	EIF4EBP1	Protein coding	8p11.23
M6ATAR00241	1994	ELAVL1	Protein coding	19p13.2
M6ATAR00719	1917	EEF1A2	Protein coding	20q13.33
M6ATAR00545	3309	Grp78	Protein coding	9q33.3
M6ATAR00243	2034	EPAS1	Protein coding	2p21
M6ATAR00544	9844	ELMO1	Protein coding	7p14.2-p14.1
M6ATAR00716	1969	EphA2	Protein coding	1p36.13
M6ATAR00244	2048	EPHB2	Protein coding	1p36.12
M6ATAR00504	2049	EPHB3	Protein coding	3q27.1
M6ATAR00237	1956	EGFR	Protein coding	7p11.2
M6ATAR00242	2014	EMP3	Protein coding	19q13.33
M6ATAR00589	80004	ESRP2	Protein coding	16q22.1
M6ATAR00723	51170	HSD17B11	Protein coding	4q22.1
M6ATAR00247	2104	ESRRG	Protein coding	1q41
M6ATAR00287	1965	EIF2S1	Protein coding	14q23.3
M6ATAR00239	8661	EIF3A	Protein coding	10q26.11
M6ATAR00240	8663	EIF3C	Protein coding	16p11.2
M6ATAR00288	1981	EIF4G1	Protein coding	3q27.1
M6ATAR00630	55959	Sulf2	Protein coding	20q13.12
M6ATAR00143	286333	FAM225A	LncRNA	9q32
M6ATAR00262	8880	FUBP1	Protein coding	1p31.1
M6ATAR00261	6624	FSCN1	Protein coding	7p22.1
M6ATAR00250	2194	FASN	Protein coding	17q25.3
M6ATAR00583	26234	FBXL5	Protein coding	4p15.32
M6ATAR00251	200933	FBXO45	Protein coding	3q29
M6ATAR00252	55294	FBXW7	Protein coding	4q31.3
M6ATAR00103	154860	FEZF1-AS1	LncRNA	7q31.32
M6ATAR00468	9575	FGA	Protein coding	.
M6ATAR00526	2264	FGFR4	Protein coding	5q35.2
M6ATAR00649	2237	FEN1	Protein coding	11q12.2
M6ATAR00254	2319	FLOT2	Protein coding	17q11.2
M6ATAR00554	5747	Fak	Protein coding	8q24.3
M6ATAR00256	2303	FOXC2	Protein coding	16q24.1
M6ATAR00257	2297	FOXD1	Protein coding	5q13.2
M6ATAR00258	2305	FOXM1	Protein coding	12p13.33

M6ATAR00259	2308	FOXO1	Protein coding	13q14.11
M6ATAR00260	2309	FOXO3	Protein coding	6q21
M6ATAR00094	84793	FOXD2-AS1	LncRNA	1p33
M6ATAR00096	400550	FENDRR	LncRNA	16q24.1
M6ATAR00642	11211	FZD10	Protein coding	12q24.33
M6ATAR00718	8324	FZD7	Protein coding	2q33.1
M6ATAR00206	595	CCND1	Protein coding	11q13.3
M6ATAR00207	894	CCND2	Protein coding	12p13.32
M6ATAR00208	898	CCNE1	Protein coding	19q12
M6ATAR00752	9133	CCNB2	Protein coding	15q22.2
M6ATAR00798	11337	GABARAP	Protein coding	17p13.1
M6ATAR00573	3965	LGALS9	Protein coding	17q11.2
M6ATAR00727	2697	GJA1	Protein coding	6q22.31
M6ATAR00095	100506046	GAS5-AS1	LncRNA	1q25.1
M6ATAR00726	81849	ST6GALNAC5	Protein coding	1p31.1
M6ATAR00266	5270	SERPINE2	Protein coding	2q36.1
M6ATAR00269	6513	SLC2A1	Protein coding	1p34.2
M6ATAR00263	2538	G6PC1	Protein coding	17q21.31
M6ATAR00764	2539	G6PD	Protein coding	Xq28
M6ATAR00349	2902	GRIN1	Protein coding	9q34.3
M6ATAR00268	2932	GSK3B	Protein coding	3q13.33
M6ATAR00148	60674	GAS5	LncRNA	1q25.1
M6ATAR00379	3845	KRAS	Protein coding	12p12.1
M6ATAR00767	2778	GNAS	Protein coding	20q13.32
M6ATAR00229	1736	DKC1	Protein coding	Xq28
M6ATAR00156	283120	H19	LncRNA	11p15.5
M6ATAR00279	3303; 3304	HSPA1A	Protein coding	6p21.33
M6ATAR00593	3297	HSF1	Protein coding	8q24.3
M6ATAR00280	3320	HSP90AA1	Protein coding	14q32.31
M6ATAR00508	10855	HPSE	Protein coding	4q21.23
M6ATAR00469	3014	HAVCR1	Protein coding	.
M6ATAR00577	84868	HAVCR2	Protein coding	5q33.3
M6ATAR00325	4233	MET	Protein coding	7q31
M6ATAR00278	6927	HNF1A	Protein coding	12q24.31
M6ATAR00255	3171	FOXA3	Protein coding	19q13.32
M6ATAR00271	3068	HDGF	Protein coding	1q23.1
M6ATAR00283	3099	HK2	Protein coding	2p12
M6ATAR00277	3146	HMGB1	Protein coding	13q12.3
M6ATAR00476	26574	HMGA1	Protein coding	.
M6ATAR00276	8091	HMGA2	Protein coding	12q14.3
M6ATAR00768	9759	HDAC4	Protein coding	2q37.3
M6ATAR00688	10014	HDAC5	Protein coding	17q21.31
M6ATAR00460	3014	H2AX	Protein coding	.
M6ATAR00669	10919	G9a	Protein coding	6p21.33
M6ATAR00249	2146	EZH2	Protein coding	7q36.1
M6ATAR00390	80854	SETD7	Protein coding	4q31.1
M6ATAR00147	493812	HCG11	LncRNA	6p22.2
M6ATAR00603	3198	HOXA1	Protein coding	7p15.2
M6ATAR00491	10481	HOXB13	Protein coding	17q21.32
M6ATAR00282	3214	HOXB4	Protein coding	17q21.32

M6ATAR00344	79923	NANOG	Protein coding	12p13.31
M6ATAR00625	4824	NKX3-1	Protein coding	8p21.2
M6ATAR00631	79618	HMBOX1	Protein coding	8p21.1-p12
M6ATAR00473	28996	HIPK2	Protein coding	.
M6ATAR00098	100874366	HOXC13-AS	LncRNA	12q13.13
M6ATAR00097	401022	HAGLR	LncRNA	2q31.1
M6ATAR00538	9448	MAP4K4	Protein coding	2q11.2
M6ATAR00064	.	hsa_circ_0000231	circRNA	.
M6ATAR00787	.	circCPSF6	circRNA	.
M6ATAR00793	.	hsa_circ_0000677	circRNA	.
M6ATAR00751	.	hsa_circ_0004287	circRNA	.
M6ATAR00057	.	hsa_circ_0004771	circRNA	.
M6ATAR00065	.	hsa_circ_0005630	circRNA	.
M6ATAR00067	.	hsa_circ_0008399	circRNA	.
M6ATAR00495	.	hsa_circ_0008542	circRNA	.
M6ATAR00058	.	hsa_circ_0021427	circRNA	.
M6ATAR00068	.	hsa_circ_0029589	circRNA	.
M6ATAR00069	.	hsa_circ_0058493	circRNA	.
M6ATAR00059	.	hsa_circ_0066779	circRNA	.
M6ATAR00061	.	hsa_circ_0077837	circRNA	.
M6ATAR00070	.	hsa_circ_0081609	circRNA	.
M6ATAR00071	.	hsa_circ_0087293	circRNA	.
M6ATAR00062	.	hsa_circ_0089552	circRNA	.
M6ATAR00072	.	hsa_circ_0092493	circRNA	.
M6ATAR00025	.	hsa-miR-126-5p	microRNA	.
M6ATAR00012	.	hsa-miR-1266-5p	microRNA	.
M6ATAR00013	.	hsa-miR-1268a	microRNA	.
M6ATAR00034	.	hsa-miR-129-5p	microRNA	.
M6ATAR00039	.	hsa-miR-133a-3p	microRNA	.
M6ATAR00055	.	hsa-miR-139-3p	microRNA	.
M6ATAR00056	.	hsa-miR-140-3p	microRNA	.
M6ATAR00017	.	hsa-miR-143-3p	microRNA	.
M6ATAR00050	.	hsa-miR-143-3p	microRNA	.
M6ATAR00063	.	hsa-miR-146a-5p	microRNA	.
M6ATAR00035	.	hsa-miR-150-5p	microRNA	.
M6ATAR00030	406955	hsa-mir-181b-1	microRNA	1q32.1
M6ATAR00540	.	hsa-miR-181b-3p	microRNA	.
M6ATAR00666	.	hsa-miR-181d-5p	microRNA	.
M6ATAR00032	.	hsa-miR-183-3p	microRNA	.
M6ATAR00033	.	hsa-miR-186-5p	microRNA	.
M6ATAR00465	.	hsa-miR-1914-3p	microRNA	.
M6ATAR00641	.	hsa-miR-1915-3p	microRNA	.
M6ATAR00599	574455	hsa-miR-193b	microRNA	16p13.12
M6ATAR00040	.	hsa-miR-199a-5p	microRNA	.
M6ATAR00024	.	hsa-miR-19a-3p	microRNA	.
M6ATAR00049	.	hsa-miR-21-5p	microRNA	.
M6ATAR00028	.	hsa-miR-221-3p	microRNA	.
M6ATAR00733	.	hsa-miR-222-3p	microRNA	.
M6ATAR00021	.	hsa-miR-25-3p	microRNA	.
M6ATAR00653	407017	hsa-miR-26b	microRNA	2q35

M6ATAR00010	.	hsa-miR-29a-3p	microRNA	.
M6ATAR00011	.	hsa-miR-29b-3p	microRNA	.
M6ATAR00534	.	hsa-miR-30c-1-3p	microRNA	.
M6ATAR00617	.	hsa-miR-30c-2-3p	microRNA	.
M6ATAR00743	407033	hsa-miR-30d	microRNA	8q24.22
M6ATAR00037	.	hsa-miR-31-5p	microRNA	.
M6ATAR00672	.	hsa-miR-320a-3p	microRNA	.
M6ATAR00047	.	hsa-miR-320b	microRNA	.
M6ATAR00045	.	hsa-miR-320c	microRNA	.
M6ATAR00046	.	hsa-miR-320d	microRNA	.
M6ATAR00651	407040	hsa-miR-34a	microRNA	1p36.22
M6ATAR00783	.	hsa-miR-375-3p	microRNA	.
M6ATAR00791	.	hsa-miR-380-3p	microRNA	.
M6ATAR00026	.	hsa-miR-422a	microRNA	.
M6ATAR00739	.	hsa-miR-5581-3p	microRNA	.
M6ATAR00730	.	hsa-miR-5586-5p	microRNA	.
M6ATAR00493	.	hsa-miR-582-3p	microRNA	.
M6ATAR00735	.	hsa-miR-589-5p	microRNA	.
M6ATAR00014	.	hsa-miR-671-3p	microRNA	.
M6ATAR00613	.	hsa-miR-671-5p	microRNA	.
M6ATAR00043	.	hsa-miR-766-5p	microRNA	.
M6ATAR00009	.	hsa-miR-873-5p	microRNA	.
M6ATAR00507	.	hsa-miR-99a-5p	microRNA	.
M6ATAR00274	3091	HIF1A	Protein coding	14q23.2
M6ATAR00292	3551	IKBKB	Protein coding	8p11.21
M6ATAR00558	3643	INSR	Protein coding	19p13.2
M6ATAR00559	3667	IRS1	Protein coding	2q36.3
M6ATAR00497	3480	IGF1R	Protein coding	15q26.3
M6ATAR00291	3479	IGF1	Protein coding	12q23.2
M6ATAR00297	3655	ITGA6	Protein coding	2q31.1
M6ATAR00298	3688	ITGB1	Protein coding	10p11.22
M6ATAR00650	3691	ITGB4	Protein coding	17q25.1
M6ATAR00155	3690	ITGB3	Protein coding	17q21.32
M6ATAR00284	3383	ICAM1	Protein coding	19p13.2
M6ATAR00290	3456	IFNB1	Protein coding	9p21.3
M6ATAR00521	3458	IFN-gamma	Protein coding	12q15
M6ATAR00753	3459	IFNGR1	Protein coding	6q23.3
M6ATAR00525	3659	Irf1	Protein coding	5q31.1
M6ATAR00296	3661	IRF3	Protein coding	19q13.33
M6ATAR00289	3433	IFIT2	Protein coding	10q23.31
M6ATAR00536	103	ADAR1	Protein coding	1q21.3
M6ATAR00295	3609	ILF3	Protein coding	19p13.2
M6ATAR00294	3553	IL1B	Protein coding	2q14.1
M6ATAR00293	3589	IL11	Protein coding	19q13.42
M6ATAR00529	3569	IL-6	Protein coding	7p15.3
M6ATAR00458	26574	ITSN2	Protein coding	.
M6ATAR00744	4312	MMP1	Protein coding	11q22.2
M6ATAR00584	3658	IRP2	Protein coding	15q25.1
M6ATAR00286	3417	IDH1	Protein coding	2q34
M6ATAR00031	.	KB-1980E6.3	LncRNA	.

M6ATAR00082	106144538	KCNK15-AS1	LncRNA	20q13.12
M6ATAR00078	104797538	KCNMB2-AS1	LncRNA	3q26.32
M6ATAR00154	10984	KCNQ1OT1	LncRNA	11p15.5
M6ATAR00306	9817	KEAP1	Protein coding	19p13.2
M6ATAR00769	3855	KRT7	Protein coding	12q13.13
M6ATAR00489	26574	KIF26B	Protein coding	.
M6ATAR00307	11004	KIF2C	Protein coding	1p34.1
M6ATAR00692	3797	KIF3C	Protein coding	2p23.3
M6ATAR00792	109729127	KRT7-AS	LncRNA	12q13.13
M6ATAR00510	11278	KLF12	Protein coding	13q22.1
M6ATAR00714	10365	KLF2	Protein coding	19p13.11
M6ATAR00310	9314	KLF4	Protein coding	9q31.2
M6ATAR00311	688	KLF5	Protein coding	13q22.1
M6ATAR00771	3939	LDHA	Protein coding	11p15.1
M6ATAR00146	151534	LBX2-AS1	LncRNA	2p13.1
M6ATAR00041	.	LCAT3	LncRNA	.
M6ATAR00316	11006	LILRB4	Protein coding	19q13.42
M6ATAR00492	961	CD47	Protein coding	3q13.12
M6ATAR00766	27165	GLS2	Protein coding	12q13.3
M6ATAR00099	100506495	LIFR-AS1	LncRNA	5p13.1
M6ATAR00628	3927	LASP1	Protein coding	17q12
M6ATAR00675	171022	ABHD11-AS1	LncRNA	7q11.23
M6ATAR00742	285194	TUSC7	LncRNA	3q13.31
M6ATAR00562	.	LINE-1	Protein coding	.
M6ATAR00317	54596	L1TD1	Protein coding	1p31.3
M6ATAR00601	.	Lnc_CDC5L	LncRNA	.
M6ATAR00001	.	Lnc_D63785	LncRNA	.
M6ATAR00588	.	Lnc-LSG1	LncRNA	.
M6ATAR00602	.	Lnc_STAT3	LncRNA	.
M6ATAR00080	101928687	LNCAROD	LncRNA	10q21.1
M6ATAR00788	100130776	AGAP2-AS1	LncRNA	12q14.1
M6ATAR00659	101927541	LINC01273	LncRNA	20q13.13
M6ATAR00081	104355288	LINC01320	LncRNA	2p22.3
M6ATAR00691	107985879	LINC01833	LncRNA	2p21
M6ATAR00074	112588022	LINC02598	LncRNA	12p13.2
M6ATAR00073	644794	LINC02604	LncRNA	7q11.21
M6ATAR00105	100873962	LINC00278	LncRNA	Yp11.31
M6ATAR00100	728192	LINC00460	LncRNA	13q33.2
M6ATAR00157	56651	LINC00470	LncRNA	18p11.32
M6ATAR00784	647979	LncRNA NORAD	LncRNA	20q11.23
M6ATAR00568	106660612	LINC00680	LncRNA	6p11.2
M6ATAR00093	439990	LINC00857	LncRNA	10q22.3
M6ATAR00090	100505865	LINC00920	LncRNA	16q21
M6ATAR00089	100292680	LINC00942	LncRNA	12p13.33
M6ATAR00088	100506305	LINC00958	LncRNA	11p15.3
M6ATAR00678	116372	LYPD1	Protein coding	2q21.2
M6ATAR00315	51176	LEF1	Protein coding	4q25
M6ATAR00332	4321	MMP12	Protein coding	11q22.2
M6ATAR00023	.	.	Protein coding	.
M6ATAR00005	.	.	Protein coding	.

M6ATAR00537	6416	MAP2K4	Protein coding	17p12
M6ATAR00565	2872	MNK2	Protein coding	19p13.3
M6ATAR00337	65108	MARCKSL1	Protein coding	1p35.1
M6ATAR00150	55384	MEG3	LncRNA	14q32.2
M6ATAR00687	10893	MMP24	Protein coding	20q11.22
M6ATAR00335	4318	MMP9	Protein coding	20q13.12
M6ATAR00324	284071	MEIOC	Protein coding	17q21.31
M6ATAR00702	4160	MC4R	Protein coding	18q21.32
M6ATAR00754	9500	MAGED1	Protein coding	Xp11.22
M6ATAR00171	8728	ADAM19	Protein coding	5q33.3
M6ATAR00745	7076	TIMP-1	Protein coding	Xp11.3
M6ATAR00648	7077	TIMP2	Protein coding	17q25.3
M6ATAR00431	7078	TIMP3	Protein coding	22q12.3
M6ATAR00706	261729	STEAP2	Protein coding	7q21.13
M6ATAR00141	378938	MALAT1	LncRNA	11q13.1
M6ATAR00542	9112	MTA1	Protein coding	14q32.33
M6ATAR00326	4255	MGMT	Protein coding	10q26.3
M6ATAR00637	80312	TET1	Protein coding	10q21.3
M6ATAR00228	54487	DGCR8	Protein coding	22q11.21
M6ATAR00138	406901	MIR107	microRNA	10q23.31
M6ATAR00109	100302142	MIR1246	microRNA	2q31.1
M6ATAR00137	406913	MIR126	microRNA	9q34.3
M6ATAR00110	100302270	MIR1305	microRNA	4q34.3
M6ATAR00136	406937	MIR145	microRNA	5q32
M6ATAR00785	406942	MIR150	microRNA	19q13.33
M6ATAR00135	406947	MIR155	microRNA	21q21.3
M6ATAR00134	406962	MIR186	microRNA	1p31.1
M6ATAR00132	406991	MIR21	microRNA	17q23.1
M6ATAR00131	406993	MIR211	microRNA	15q13.3
M6ATAR00130	407006	MIR221	microRNA	Xp11.3
M6ATAR00129	407007	MIR222	microRNA	Xp11.3
M6ATAR00128	407010	MIR23A	microRNA	19p13.12
M6ATAR00127	407014	MIR25	microRNA	7q22.1
M6ATAR00126	407021	MIR29A	microRNA	7q32.3
M6ATAR00121	442904	MIR335	microRNA	7q32.2
M6ATAR00119	442915	MIR370	microRNA	14q32.31
M6ATAR00118	494324	MIR375	microRNA	2q35
M6ATAR00115	693161	MIR576	microRNA	4q25
M6ATAR00114	724030	MIR660	microRNA	Xp11.23
M6ATAR00111	100033819	MIR675	microRNA	11p15.5
M6ATAR00125	407043	MIR7-1	microRNA	9q21.32
M6ATAR00124	407050	MIR93	microRNA	7q22.1
M6ATAR00485	406884	MIRLET7B	microRNA	22q13.31
M6ATAR00139	406890	MIRLET7G	microRNA	3p21.2
M6ATAR00611	4133	MAP2	Protein coding	2q34
M6ATAR00331	81631	MAP1LC3B	Protein coding	16q24.2
M6ATAR00626	4969	OGN	Protein coding	9q22.31
M6ATAR00600	399959	MIR100HG	microRNA	11q24.1
M6ATAR00552	.	MIR17-92	microRNA	.
M6ATAR00585	406958	MIR182	microRNA	7q32.2

M6ATAR00697	406970	MIR194-2	microRNA	11q13.1
M6ATAR00717	574506	MIR503	microRNA	Xq26.3
M6ATAR00483	5395	PMS2	Protein coding	.
M6ATAR00320	57506	MAVS	Protein coding	20p13
M6ATAR00327	5594	MAPK1	Protein coding	22q11.22
M6ATAR00330	1432	MAPK14	Protein coding	6p21.31
M6ATAR00328	5595	MAPK3	Protein coding	16p11.2
M6ATAR00329	5599	MAPK8	Protein coding	10q11.22
M6ATAR00018	.	mmu-miR-365-3p	microRNA	.
M6ATAR00020	.	mmu-miR-7212-5p	microRNA	.
M6ATAR00336	79817	MOB3B	Protein coding	9p21.2
M6ATAR00760	6347	Ccl2	Protein coding	17q12
M6ATAR00532	4087	SMAD2	Protein coding	18q21.1
M6ATAR00396	4088	SMAD3	Protein coding	15q22.33
M6ATAR00738	4091	SMAD6	Protein coding	15q22.31
M6ATAR00397	4092	SMAD7	Protein coding	18q21.1
M6ATAR00710	994	CDC25B	Protein coding	20p13
M6ATAR00722	4584	MUC3A	Protein coding	7q22.1
M6ATAR00772	4363	MRP1	Protein coding	16p13.11
M6ATAR00375	5728	PTEN	Protein coding	10q23.31
M6ATAR00341	4609	MYC	Protein coding	8q24.21
M6ATAR00342	4615	MYD88	Protein coding	3p22.2
M6ATAR00343	7593	MZF1	Protein coding	19q13.43
M6ATAR00079	104564225	MHRT	LncRNA	14q11.2
M6ATAR00654	4625	Myh7	Protein coding	14q11.2
M6ATAR00732	9088	PKMYT1	Protein coding	16p13.3
M6ATAR00605	114548	NLRP3	Protein coding	1q44
M6ATAR00708	22861	NLRP1	Protein coding	17p13
M6ATAR00393	23411	SIRT1	Protein coding	10q21.3
M6ATAR00394	51548	SIRT6	Protein coding	19p13.3
M6ATAR00264	4616	GADD45B	Protein coding	19p13.3
M6ATAR00634	79026	AHNAK	Protein coding	11q12.3
M6ATAR00667	83988	NCALD	Protein coding	8q22.3
M6ATAR00612	4760	NEUROD1	Protein coding	2q31.3
M6ATAR00351	4851	NOTCH1	Protein coding	9q34.3
M6ATAR00566	4853	NOTCH2	Protein coding	1p12
M6ATAR00162	6510	SLC1A5	Protein coding	19q13.32
M6ATAR00683	4792	Nfkbia	Protein coding	14q13.2
M6ATAR00144	254128	NIFK-AS1	LncRNA	2q14.3
M6ATAR00462	2956	NHEJ1	Protein coding	.
M6ATAR00557	25805	BAMBI	Protein coding	10p12.1
M6ATAR00786	751580	LINC00106	LncRNA	Xp22.33 and Yp11.32
M6ATAR00348	4780	NFE2L2	Protein coding	2q31.2
M6ATAR00054	4790	NFKB1	Protein coding	4q24
M6ATAR00140	283131	NEAT1	LncRNA	11q13.1
M6ATAR00352	7101	NR2E1	Protein coding	6q21
M6ATAR00604	8204	NRIP1	Protein coding	21q11.2-q21.1
M6ATAR00490	4924	NUCB1	Protein coding	19q13.33
M6ATAR00606	8996	ARC	Protein coding	16q22.1
M6ATAR00572	4869	NPM1	Protein coding	5q35.1

M6ATAR00541	2186	BPTF	Protein coding	17q24.2
M6ATAR00354	29789	OLA1	Protein coding	2q31.1
M6ATAR00355	29948	OSGIN1	Protein coding	16q23.3
M6ATAR00503	10062	LXRA	Protein coding	11p11.2
M6ATAR00549	9127	P2RX6	Protein coding	22q11.21
M6ATAR00646	64065	PERP	Protein coding	6q23.3
M6ATAR00535	5831	PYCR1	Protein coding	17q25.3
M6ATAR00580	5396	PRRX1	Protein coding	1q24.2
M6ATAR00547	3651	Pdx1	Protein coding	13q12.2
M6ATAR00376	5745	PTH1R	Protein coding	3p21.31
M6ATAR00363	5178	PEG3	Protein coding	19q13.43
M6ATAR00359	55010	PARPBP	Protein coding	12q23.2
M6ATAR00362	5163	PDK1	Protein coding	2q31.1
M6ATAR00364	5187	PER1	Protein coding	17p13.1
M6ATAR00236	1962	EHHADH	Protein coding	3q27.2
M6ATAR00475	5395	PPARA	Protein coding	.
M6ATAR00592	5468	PPARG	Protein coding	3p25.2
M6ATAR00746	23481	PES1	Protein coding	22q12.2
M6ATAR00695	55274	PHF10	Protein coding	6q27
M6ATAR00366	23035	PHLPP2	Protein coding	16q22.2
M6ATAR00358	5296	PIK3R2	Protein coding	19p13.11
M6ATAR00624	64077	LHPP	Protein coding	10q26.13
M6ATAR00368	5290	PIK3CA	Protein coding	3q26.32
M6ATAR00369	5291	PIK3CB	Protein coding	3q22.3
M6ATAR00608	5176	PEDF	Protein coding	17p13.3
M6ATAR00761	948	Cd36	Protein coding	7q21.11
M6ATAR00665	56034	PDGFC	Protein coding	4q32.1
M6ATAR00620	5156	PDGFRA	Protein coding	4q12
M6ATAR00571	5159	PDGFRB	Protein coding	5q32
M6ATAR00361	5154	PDGFA	Protein coding	7p22.3
M6ATAR00019	.	PncRNA-D	LncRNA	.
M6ATAR00551	142	PARP1	Protein coding	1q42.12
M6ATAR00519	8658	Tankyrase	Protein coding	8p23.1
M6ATAR00664	648	BMI1	Protein coding	10p12.2
M6ATAR00515	171023	Asxl1	Protein coding	20q11.21
M6ATAR00561	81033	KCNH6	Protein coding	17q23.3
M6ATAR00022	5460	POU5F1	Protein coding	6p21.33
M6ATAR00004	.	p-P70	Protein coding	.
M6ATAR00373	10891	PPARGC1A	Protein coding	4p15.2
M6ATAR00564	7799	PRDM2	Protein coding	1p36.21
M6ATAR00253	9589	WTAP	Protein coding	6q25.3
M6ATAR00632	.	pri-miR-10a	microRNA	.
M6ATAR00623	.	pri-miR-143	microRNA	.
M6ATAR00789	.	pri-miR-221	microRNA	.
M6ATAR00790	.	pri-miR-222	microRNA	.
M6ATAR00636	.	pri-miR-27	microRNA	.
M6ATAR00712	1950	EGF	Protein coding	4q25
M6ATAR00796	5133	PDCD1	Protein coding	2q37.3
M6ATAR00360	29126	CD274	Protein coding	9p24.1
M6ATAR00512	5036	PA2G4	Protein coding	12q13.2

M6ATAR00618	84335	AKT1S1	Protein coding	19q13.33
M6ATAR00689	8842	CD133	Protein coding	4p15.32
M6ATAR00365	5743	PTGS2	Protein coding	1q31.1
M6ATAR00496	100506696	PCAT6	LncRNA	1q32.1
M6ATAR00474	.	AATF	Protein coding	.
M6ATAR00178	3276	PRMT1	Protein coding	19q13.33
M6ATAR00684	10419	PRMT5	Protein coding	14q11.2
M6ATAR00173	26523	AGO1	Protein coding	1p34.3
M6ATAR00638	27161	AGO2	Protein coding	8q24.3
M6ATAR00758	7832	BTG2	Protein coding	1q32.1
M6ATAR00338	862	RUNX1T1	Protein coding	8q21.3
M6ATAR00248	2113	ETS1	Protein coding	11q24.3
M6ATAR00762	92359	CRB3	Protein coding	19p13.3
M6ATAR00598	.	E6	Protein coding	.
M6ATAR00531	81610	FAM83D	Protein coding	20q11.23
M6ATAR00770	5581	PRKCE	Protein coding	2p21
M6ATAR00621	5583	PKC-eta	Protein coding	14q23.1
M6ATAR00720	4753	NELL2	Protein coding	12q12
M6ATAR00563	8650	NUMB	Protein coding	14q24.3
M6ATAR00374	5727	PTCH1	Protein coding	9q22.32
M6ATAR00372	5494	PPM1A	Protein coding	14q23.1
M6ATAR00633	55920	RCC2	Protein coding	1p36.13
M6ATAR00655	60485	SAV1	Protein coding	14q22.1
M6ATAR00662	6418	SET	Protein coding	9q34.11
M6ATAR00409	10253	SPRY2	Protein coding	13q31.1
M6ATAR00448	25962	VIRMA	Protein coding	8q22.1
M6ATAR00670	7474	WNT5A	Protein coding	3p14.3
M6ATAR00680	51646	YPEL5	Protein coding	2p23.1
M6ATAR00429	7052	TGM2	Protein coding	20q11.23
M6ATAR00619	79633	FAT4	Protein coding	4q28.1
M6ATAR00700	5966	c-Rel	Protein coding	2p16.1
M6ATAR00694	7471	Wnt1	Protein coding	12q13.12
M6ATAR00509	103752588	PACERR	LncRNA	1q31.1
M6ATAR00167	403314	APOBEC4	Protein coding	1q25.3
M6ATAR00281	.	HSPA7	Protein coding	1q23.3
M6ATAR00377	.	PTTG3P	Protein coding	8q13.1
M6ATAR00181	.	ARHGAP5-AS1	Protein coding	14q12
M6ATAR00224	.	DANCR	Protein coding	4q12
M6ATAR00153	5820	PVT1	LncRNA	8q24.21
M6ATAR00555	5166	PDK4	Protein coding	7q21.3
M6ATAR00312	5315	PKM	Protein coding	15q23
M6ATAR00175	207	AKT1	Protein coding	14q32.33
M6ATAR00176	10000	AKT3	Protein coding	1q43-q44
M6ATAR00773	10635	RAD51AP1	Protein coding	12p13.32
M6ATAR00319	10542	LAMTOR5	Protein coding	1p13.3
M6ATAR00709	8826	IQGAP1	Protein coding	15q26.1
M6ATAR00380	57403	RAB22A	Protein coding	20q13.32
M6ATAR00381	9821	RB1CC1	Protein coding	8q11.23
M6ATAR00245	2064	ERBB2	Protein coding	17q12
M6ATAR00384	8767	RIPK2	Protein coding	8q21.3

M6ATAR00576	5788	CD45	Protein coding	1q31.3-q32.1
M6ATAR00378	5914	RARA	Protein coding	17q21.2
M6ATAR00797	.	PML-RAR-Alpha	Protein coding	.
M6ATAR00698	10743	RAI1	Protein coding	17p11.2
M6ATAR00661	23089	PEG10	Protein coding	7q21.3
M6ATAR00795	394	ARHGAP5	Protein coding	14q12
M6ATAR00383	10395	DLC1	Protein coding	8p22
M6ATAR00647	9181	ARHGEF2	Protein coding	1q22
M6ATAR00142	78998	RHPN1-AS1	LncRNA	8q24.3
M6ATAR00385	29102	DROSHA	Protein coding	5p13.3
M6ATAR00313	6198	RPS6KB1	Protein coding	17q23.1
M6ATAR00567	23586	RIG-I	Protein coding	9p21.1
M6ATAR00345	6023	NME1	LncRNA	9p13.3
M6ATAR00353	54888	NSUN2	Protein coding	5p15.31
M6ATAR00350	28987	NOB1	Protein coding	16q22.1
M6ATAR00052	.	RP11-108L7.15	LncRNA	.
M6ATAR00513	860	Runx2	Protein coding	6p21.1
M6ATAR00457	488	ATP2A2	Protein coding	.
M6ATAR00413	8578	SCARF1	Protein coding	17p13.3
M6ATAR00645	6423	SFRP2	Protein coding	4q31.3
M6ATAR00076	107126285	SEPTIN14P20	LncRNA	20q13.33
M6ATAR00410	8878	SQSTM1	Protein coding	5q35.3
M6ATAR00570	10772	SRSF10	Protein coding	1p36.11
M6ATAR00415	9295	SRSF11	Protein coding	1p31.1
M6ATAR00416	6428	SRSF3	Protein coding	6p21.31-p21.2
M6ATAR00417	6431	SRSF6	Protein coding	20q13.11
M6ATAR00779	6432	SRSF7	Protein coding	2p22.1
M6ATAR00734	6789	STK4	Protein coding	20q13.12
M6ATAR00314	26524	LATS2	Protein coding	13q12.11
M6ATAR00339	2475	MTOR	Protein coding	1p36.22
M6ATAR00367	65018	PINK1	Protein coding	1p36.12
M6ATAR00370	5347	PLK1	Protein coding	16p12.2
M6ATAR00420	6794	STK11	Protein coding	19p13.3
M6ATAR00444	8408	ULK1	Protein coding	12q24.33
M6ATAR00192	472	ATM	Protein coding	11q22.3
M6ATAR00461	5395	SRF	Protein coding	.
M6ATAR00801	6773	STAT2	Protein coding	12q13.3
M6ATAR00418	6774	STAT3	Protein coding	17q21.2
M6ATAR00736	6776	STAT5A	Protein coding	17q21.2
M6ATAR00395	90417	KNSTRN	Protein coding	15q15.1
M6ATAR00704	84973	SNHG7	LncRNA	9q34.3
M6ATAR00117	23642	SNHG1	LncRNA	11q12.3
M6ATAR00477	8420	SNHG3	LncRNA	1p35.3
M6ATAR00113	724102	SNHG4	LncRNA	5q31.2
M6ATAR00703	8243	SMC1A	Protein coding	Xp11.22
M6ATAR00048	.	S-mu-GLT	LncRNA	.
M6ATAR00387	6557	SLC12A1	Protein coding	15q21.1
M6ATAR00498	30061	FPN1	Protein coding	2q32.2
M6ATAR00591	.	SOX	Protein coding	.
M6ATAR00802	6712	SPTBN2	Protein coding	11q13.2

M6ATAR00629	8877	SPHK1	Protein coding	17q25.1
M6ATAR00778	56848	SPHK2	Protein coding	19q13.33
M6ATAR00494	10946	SF3A3	Protein coding	1p34.3
M6ATAR00391	6421	SFPQ	Protein coding	1p34.3
M6ATAR00464	5395	SPRED2	Protein coding	.
M6ATAR00414	6733	SRPK2	Protein coding	7q22.3
M6ATAR00400	27044	SND1	Protein coding	7q32.1
M6ATAR00388	6319	SCD	Protein coding	10q24.31
M6ATAR00411	6720	SREBF1	Protein coding	17p11.2
M6ATAR00419	340061	STING1	Protein coding	5q31.2
M6ATAR00530	4314	MMP-3	Protein coding	11q22.2
M6ATAR00800	29843	SENP1	Protein coding	12q13.11
M6ATAR00402	6648	SOD2	Protein coding	6q25.3
M6ATAR00774	8651	SOCS1	Protein coding	16p13.13
M6ATAR00401	8835	SOCS2	Protein coding	12q
M6ATAR00610	9021	SOCS3	Protein coding	17q25.3
M6ATAR00776	122809	SOCS4	Protein coding	14q22.3
M6ATAR00775	9655	SOCS5	Protein coding	2p21
M6ATAR00777	9306	SOCS6	Protein coding	18q22.2
M6ATAR00673	102724316	SVIL-AS1	LncRNA	10p11.23
M6ATAR00682	64223	MLST8	Protein coding	16p13.3
M6ATAR00595	10024	TROAP	Protein coding	12q13.12
M6ATAR00423	6886	TAL1	Protein coding	1p33
M6ATAR00075	100506797	THORLNC	LncRNA	2q14.2
M6ATAR00737	7048	TGF-Beta-R2	Protein coding	3p24.1
M6ATAR00102	439931	THAP7-AS1	LncRNA	22q11.21
M6ATAR00430	7295	TXN	Protein coding	9q31.3
M6ATAR00578	81567	TXNDC5	Protein coding	6p24.3
M6ATAR00486	5395	THBS1	Protein coding	.
M6ATAR00308	7083	TK1	Protein coding	17q25.3
M6ATAR00615	7980	TFPI-2	Protein coding	7q21.3
M6ATAR00725	7273	Titin	Protein coding	2q31.2
M6ATAR00514	7128	TNFAIP3	Protein coding	6q23.3
M6ATAR00435	7185	TRAF1	Protein coding	9q33.2
M6ATAR00674	9618	TRAF4	Protein coding	17q11.2
M6ATAR00671	7188	TRAF5	Protein coding	1q32.3
M6ATAR00596	7189	TRAF6	Protein coding	11p12
M6ATAR00007	.	Tnfrsf2	Protein coding	.
M6ATAR00265	2625	GATA3	Protein coding	10p14
M6ATAR00607	6934	TCF7L2	Protein coding	10q25.2-q25.3
M6ATAR00180	7022	TFAP2C	Protein coding	20q13.31
M6ATAR00179	7020	TFAP2A	Protein coding	6p24.3
M6ATAR00234	1869	E2F1	Protein coding	20q11.22
M6ATAR00235	1871	E2F3	Protein coding	6p22.3
M6ATAR00560	79733	E2F8	Protein coding	11p15.1
M6ATAR00657	1877	E4F1	Protein coding	16p13.3
M6ATAR00426	7942	TFEB	Protein coding	6p21.1
M6ATAR00272	3280	HES1	Protein coding	3q29
M6ATAR00273	388585	HES5	Protein coding	1p36.32
M6ATAR00505	3097	HIVEP2	Protein coding	6q24.2

M6ATAR00524	6772	Stat1	Protein coding	2q32.2
M6ATAR00301	3725	JUN	Protein coding	1p32.1
M6ATAR00302	3726	JUNB	Protein coding	19p13.13
M6ATAR00425	5970	RELA	Protein coding	11q13.1
M6ATAR00408	6688	SPI1	Protein coding	11p11.2
M6ATAR00403	6663	SOX10	Protein coding	22q13.1
M6ATAR00404	6657	SOX2	Protein coding	3q26.33
M6ATAR00405	6659	SOX4	Protein coding	6p22.3
M6ATAR00406	6667	SP1	Protein coding	12q13.13
M6ATAR00407	6668	SP2	Protein coding	17q21.32
M6ATAR00340	4602	MYB	Protein coding	6q23.3
M6ATAR00451	10413	YAP1	Protein coding	11q22.1
M6ATAR00424	8463	TEAD2	Protein coding	19q13.33
M6ATAR00635	7528	YY1	Protein coding	14q32.2
M6ATAR00427	7037	TFRC	Protein coding	3q29
M6ATAR00422	10460	TACC3	Protein coding	4p16.3
M6ATAR00488	5395	TGFB1	Protein coding	.
M6ATAR00428	7042	TGFB2	Protein coding	1q41
M6ATAR00581	6876	SM22alpha	Protein coding	11q23.3
M6ATAR00656	9697	TRAM2	Protein coding	6p12.2
M6ATAR00389	7095	SEC62	Protein coding	3q26.2
M6ATAR00478	5395	TM9SF1	Protein coding	.
M6ATAR00506	28951	TRIB2	Protein coding	2p24.3
M6ATAR00436	23650	TRIM29	Protein coding	11q23.3
M6ATAR00432	7124	TNF	Protein coding	6p21.33
M6ATAR00434	8797	TNFRSF10A	Protein coding	8p21.3
M6ATAR00433	355	FAS	Protein coding	10q23.31
M6ATAR00357	8626	TP63	Protein coding	3q28
M6ATAR00763	5610	eIF2AK2	Protein coding	2p22.2
M6ATAR00511	2534	FYN	Protein coding	6q21
M6ATAR00299	3717	JAK2	Protein coding	9p24.1
M6ATAR00300	3718	JAK3	Protein coding	19p13.11
M6ATAR00686	7075	TIE1	Protein coding	1p34.2
M6ATAR00443	558	AXL	Protein coding	19q13.2
M6ATAR00639	5777	PTPN6	Protein coding	12p13.31
M6ATAR00149	26121	PRPF31	Protein coding	19q13.42
M6ATAR00084	550112	UBA6-DT	LncRNA	4q13.2
M6ATAR00442	56893	UBQLN4	Protein coding	1q22
M6ATAR00438	7398	USP1	Protein coding	1p31.3
M6ATAR00439	9097	USP14	Protein coding	18p11.32
M6ATAR00501	23326	USP22	Protein coding	17p11.2
M6ATAR00668	7375	USP4	Protein coding	3p21.31
M6ATAR00440	84196	USP48	Protein coding	1p36.12
M6ATAR00441	7874	USP7	Protein coding	16p13.2
M6ATAR00640	1540	CYLD	Protein coding	16q12.1
M6ATAR00799	7329	UBE2I	Protein coding	16p13.3
M6ATAR00705	257000	TINCR	Protein coding	19p13.3
M6ATAR00437	11065	UBE2C	Protein coding	20q13.12
M6ATAR00590	55236	UBA6	Protein coding	4q13.2
M6ATAR00190	10533	ATG7	Protein coding	3p25.3

M6ATAR00186	9140	ATG12	Protein coding	5q22.3
M6ATAR00188	64422	ATG3	Protein coding	3q13.2
M6ATAR00731	51035	UBXN1	Protein coding	11q12.3
M6ATAR00780	7364	UGT2B7	Protein coding	4q13.2
M6ATAR00609	7371	UCK2	Protein coding	1q24.1
M6ATAR00663	5328	PLAU	Protein coding	10q22.2
M6ATAR00107	652995	UCA1	LncRNA	19p13.12
M6ATAR00449	84313	VPS25	Protein coding	17q21.31
M6ATAR00445	81839	VANGL1	Protein coding	1p13.1
M6ATAR00487	7412	VCAM1	Protein coding	.
M6ATAR00446	7422	VEGFA	Protein coding	6p21.1
M6ATAR00781	554	Avpr2	Protein coding	Xq28
M6ATAR00518	7431	vimentin	Protein coding	10p13
M6ATAR00447	7428	VHL	Protein coding	3p25.3
M6ATAR00724	7465	WEE1	Protein coding	11p15.4
M6ATAR00480	55083	WT1	Protein coding	.
M6ATAR00450	11197	WIF1	Protein coding	12q14.3
M6ATAR00152	7503	XIST	LncRNA	Xq13.2
M6ATAR00452	4904	YBX1	Protein coding	1p34.2
M6ATAR00782	7525	YES1	Protein coding	18p11.32
M6ATAR00553	57659	ZBTB4	Protein coding	17p13.1
M6ATAR00453	6935	ZEB1	Protein coding	10p11.22
M6ATAR00454	79830	ZMYM1	Protein coding	1p34.3
M6ATAR00455	23060	ZNF609	Protein coding	15q22.1
M6ATAR00456	79755	ZNF750	Protein coding	17q25.3
M6ATAR00267	2735	GLI1	Protein coding	12q13.3
M6ATAR00399	6615	SNAI1	Protein coding	20q13.13
M6ATAR00677	6591	Slug	Protein coding	8q11.21
M6ATAR00112	441951	ZFAS1	LncRNA	20q13.13

Supplementary table 2. Potential target genes of IGF2BP3

Regulator ID	target symbol	GSE	log2FoldChange	perturbation	direction	pvalue
REG00014	MTA1	GSE109604	-24.60316349	knock down	down-regulator	2.72E-07
REG00014	BAIAP3	GSE109604	-8.810442128	knock down	down-regulator	1.36E-13
REG00014	ATP5F1D	GSE109604	-7.676062196	knock down	down-regulator	1.27E-13
REG00014	MED25	GSE109604	-6.145042772	knock down	down-regulator	0.006360168
REG00014	MAN2A2	GSE109604	-6.035720845	knock down	down-regulator	0.008166452
REG00014	ANO7	GSE109604	-5.794961219	knock down	down-regulator	0.006922137
REG00014	MMADHC	GSE109604	-5.695530545	knock down	down-regulator	0.02071685
REG00014	MPST	GSE109604	-5.541729803	knock down	down-regulator	0.029988203
REG00014	TMBIM6	GSE109604	-5.393946208	knock down	down-regulator	0.000239833
REG00014	ESM1	GSE109604	-5.231923195	knock down	down-regulator	2.15E-06
REG00014	UQCRHL	GSE109604	-5.189840681	knock down	down-regulator	2.41E-08
REG00014	TMEM106C	GSE109604	-5.099161266	knock down	down-regulator	6.96E-07
REG00014	ATAD1	GSE109604	-4.961845844	knock down	down-regulator	0.000736656
REG00014	FAM114A1	GSE109604	-4.904838173	knock down	down-regulator	0.048014615
REG00014	TNIK	GSE109604	-4.901321361	knock down	down-regulator	5.82E-08
REG00014	UEVLD	GSE109604	-4.727382277	knock down	down-regulator	2.85E-06
REG00014	BIRC5	GSE109604	-4.689516712	knock down	down-regulator	3.21E-29
REG00014	ILKAP	GSE109604	-4.662427988	knock down	down-regulator	0.04398751
REG00014	FCGRT	GSE109604	-4.628955877	knock down	down-regulator	0.043841155
REG00014	H6PD	GSE109604	-4.548735245	knock down	down-regulator	0.031495264
REG00014	LZTS3	GSE109604	-4.522536353	knock down	down-regulator	0.001732652
REG00014	BCL11A	GSE109604	-4.490428335	knock down	down-regulator	2.44E-73
REG00014	TRIM47	GSE109604	-4.440537822	knock down	down-regulator	7.37E-05
REG00014	LRRC59	GSE109604	-4.331145937	knock down	down-regulator	0.010707086
REG00014	TNFRSF19	GSE109604	-4.325734685	knock down	down-regulator	1.09E-06
REG00014	TMEM245	GSE109604	-4.295540764	knock down	down-regulator	5.47E-06
REG00014	BICD1	GSE109604	-4.197184803	knock down	down-regulator	0.003859512
REG00014	CDC25C	GSE109604	-4.010000347	knock down	down-regulator	2.95E-06
REG00014	TNK2	GSE109604	-3.945559632	knock down	down-regulator	2.84E-06
REG00014	AXIN2	GSE109604	-3.633589731	knock down	down-regulator	9.35E-12
REG00014	FSTL3	GSE109604	-3.607680473	knock down	down-regulator	0.00026877
REG00014	B3GLCT	GSE109604	-3.578112667	knock down	down-regulator	0.000440855
REG00014	TMEM164	GSE109604	-3.573017895	knock down	down-regulator	2.49E-07
REG00014	TLE5	GSE109604	-3.419983563	knock down	down-regulator	1.01E-05
REG00014	TNFRSF21	GSE109604	-3.375605603	knock down	down-regulator	5.02E-06
REG00014	TNFRSF9	GSE109604	-3.320894264	knock down	down-regulator	0.000314633
REG00014	TRPM2	GSE109604	-3.30110155	knock down	down-regulator	2.62E-05
REG00014	TSR1	GSE109604	-3.264736431	knock down	down-regulator	9.15E-05
REG00014	BTD	GSE109604	-3.133665626	knock down	down-regulator	9.51E-06
REG00014	TIMM17B	GSE109604	-3.067778815	knock down	down-regulator	0.004258762
REG00014	TMEM177	GSE109604	-2.966509755	knock down	down-regulator	0.000199627
REG00014	RBL1	GSE109604	-2.96381396	knock down	down-regulator	0.007889103
REG00014	ABCB1	GSE109604	-2.959666361	knock down	down-regulator	2.00E-10
REG00014	HEG1	GSE109604	-2.952311517	knock down	down-regulator	1.04E-10
REG00014	NRIP3	GSE109604	-2.90327871	knock down	down-regulator	5.55E-05
REG00014	POLM	GSE109604	-2.813013405	knock down	down-regulator	0.007816655
REG00014	TKFC	GSE109604	-2.756209309	knock down	down-regulator	0.001166443
REG00014	TMOD2	GSE109604	-2.752114488	knock down	down-regulator	1.65E-05

REG00014	ZNF837	GSE109604	-2.711778734	knock down	down-regulator	0.000643579
REG00014	CD320	GSE109604	-2.697498454	knock down	down-regulator	0.036021875
REG00014	IGF2BP3	GSE109604	-2.694363275	knock down	down-regulator	2.99E-08
REG00014	LONP2	GSE109604	-2.688327233	knock down	down-regulator	0.009992393
REG00014	TSPOAP1	GSE109604	-2.674630273	knock down	down-regulator	2.21E-05
REG00014	CCDC15	GSE109604	-2.659834503	knock down	down-regulator	0.004661786
REG00014	ZNF773	GSE109604	-2.657769742	knock down	down-regulator	0.037264333
REG00014	USP9X	GSE109604	-2.650383551	knock down	down-regulator	0.000154489
REG00014	TUFT1	GSE109604	-2.63288787	knock down	down-regulator	1.46E-05
REG00014	TFRC	GSE109604	-2.629275367	knock down	down-regulator	0.000210068
REG00014	UBE2R2	GSE109604	-2.626293351	knock down	down-regulator	0.001589747
REG00014	STUM	GSE109604	-2.565397441	knock down	down-regulator	0.004446677
REG00014	CSAD	GSE109604	-2.547310624	knock down	down-regulator	0.002577833
REG00014	ADAMTS2	GSE109604	-2.519068078	knock down	down-regulator	0.001783108
REG00014	UNG	GSE109604	-2.504690126	knock down	down-regulator	9.59E-06
REG00014	HSPG2	GSE109604	-2.503536384	knock down	down-regulator	0.030283709
REG00014	TCAF2	GSE109604	-2.497727092	knock down	down-regulator	0.005437987
REG00014	TOMM22	GSE109604	-2.485270792	knock down	down-regulator	9.03E-06
REG00014	FN1	GSE109604	-2.479215706	knock down	down-regulator	0.020594775
REG00014	TCEAL8	GSE109604	-2.459456773	knock down	down-regulator	0.006590192
REG00014	TMEM80	GSE109604	-2.407919757	knock down	down-regulator	0.00798861
REG00014	ADAMTS12	GSE109604	-2.374761771	knock down	down-regulator	0.04651253
REG00014	PMM1	GSE109604	-2.360347035	knock down	down-regulator	0.000602289
REG00014	TRAPPC4	GSE109604	-2.352377562	knock down	down-regulator	0.000230285
REG00014	ATP6AP1	GSE109604	-2.350966505	knock down	down-regulator	0.026593885
REG00014	TRIM21	GSE109604	-2.33874363	knock down	down-regulator	0.000188572
REG00014	C14orf119	GSE109604	-2.322176703	knock down	down-regulator	0.025802811
REG00014	WDR36	GSE109604	-2.31439633	knock down	down-regulator	2.14E-28
REG00014	TMEM175	GSE109604	-2.313854199	knock down	down-regulator	0.001210679
REG00014	RTTN	GSE109604	-2.302546949	knock down	down-regulator	0.041198625
REG00014	BTBD2	GSE109604	-2.299759515	knock down	down-regulator	1.06E-08
REG00014	TMEM59L	GSE109604	-2.266436159	knock down	down-regulator	7.27E-05
REG00014	TMEM201	GSE109604	-2.253245923	knock down	down-regulator	1.10E-05
REG00014	FAM120C	GSE109604	-2.239694286	knock down	down-regulator	0.028982444
REG00014	CROT	GSE109604	-2.232837685	knock down	down-regulator	1.05E-05
REG00014	MDH1	GSE109604	-2.215082714	knock down	down-regulator	3.52E-05
REG00014	PAK4	GSE109604	-2.212712877	knock down	down-regulator	0.002004588
REG00014	C22orf39	GSE109604	-2.20250017	knock down	down-regulator	0.027612937
REG00014	C20orf204	GSE109604	-2.198733899	knock down	down-regulator	0.025067229
REG00014	TRIP6	GSE109604	-2.184178819	knock down	down-regulator	3.23E-05
REG00014	TMEM198	GSE109604	-2.155313646	knock down	down-regulator	1.69E-05
REG00014	ATXN3	GSE109604	-2.113412848	knock down	down-regulator	0.026406999
REG00014	TMX2	GSE109604	-2.094538349	knock down	down-regulator	0.000548614
REG00014	BCAR1	GSE109604	-2.086523334	knock down	down-regulator	8.48E-22
REG00014	TTLL11	GSE109604	-2.085089682	knock down	down-regulator	0.002695405
REG00014	HLX	GSE109604	-2.0752075	knock down	down-regulator	6.37E-126
REG00014	BBS5	GSE109604	-2.050816865	knock down	down-regulator	1.31E-08
REG00014	CCN3	GSE109604	-2.038939533	knock down	down-regulator	0.002021185
REG00014	PLPP4	GSE109604	-2.026655249	knock down	down-regulator	0.000212931
REG00014	PEAR1	GSE109604	-2.024402729	knock down	down-regulator	0.017147727

REG00014	DRG1	GSE109604	-2.021864947	knock down	down-regulator	6.29E-06
REG00014	PARP2	GSE109604	-2.004595312	knock down	down-regulator	0.016994948
REG00014	CLCN5	GSE109604	-1.968906323	knock down	down-regulator	0.004292528
REG00014	FUNDC1	GSE109604	-1.964533402	knock down	down-regulator	0.00182208
REG00014	FAM193B	GSE109604	-1.951125748	knock down	down-regulator	0.001476548
REG00014	ZNF148	GSE109604	-1.950836419	knock down	down-regulator	1.24E-21
REG00014	PIH1D2	GSE109604	-1.93557465	knock down	down-regulator	6.18E-05
REG00014	COQ5	GSE109604	-1.90639404	knock down	down-regulator	0.000181199
REG00014	ZDHHC8	GSE109604	-1.902195292	knock down	down-regulator	8.51E-07
REG00014	TIMP1	GSE109604	-1.889188641	knock down	down-regulator	0.00991633
REG00014	TRMT61B	GSE109604	-1.883160964	knock down	down-regulator	0.000572153
REG00014	TVP23B	GSE109604	-1.879234585	knock down	down-regulator	5.98E-07
REG00014	LCAT	GSE109604	-1.867039505	knock down	down-regulator	0.000471316
REG00014	RNF208	GSE109604	-1.866569349	knock down	down-regulator	0.00156339
REG00014	MAGEB17	GSE109604	-1.864250542	knock down	down-regulator	0.000210506
REG00014	HNRNPH2	GSE109604	-1.863486861	knock down	down-regulator	0.013011927
REG00014	BICC1	GSE109604	-1.856933096	knock down	down-regulator	8.69E-12
REG00014	TGFBR1	GSE109604	-1.841985229	knock down	down-regulator	3.56E-06
REG00014	TUBB6	GSE109604	-1.840183669	knock down	down-regulator	0.000716607
REG00014	CHRM3	GSE109604	-1.832168738	knock down	down-regulator	0.027679306
REG00014	TMSB15A	GSE109604	-1.816675424	knock down	down-regulator	0.0028108
REG00014	ATG10	GSE109604	-1.779796717	knock down	down-regulator	0.004626035
REG00014	PIP4K2C	GSE109604	-1.778372806	knock down	down-regulator	0.000315543
REG00014	PLAU	GSE109604	-1.761954274	knock down	down-regulator	0.000101124
REG00014	USP37	GSE109604	-1.759798072	knock down	down-regulator	0.000588889
REG00014	BTN3A1	GSE109604	-1.756221935	knock down	down-regulator	6.78E-09
REG00014	DNM1	GSE109604	-1.749950955	knock down	down-regulator	0.00578644
REG00014	TCEAL3	GSE109604	-1.742184885	knock down	down-regulator	0.00542031
REG00014	FAM8A1	GSE109604	-1.735696027	knock down	down-regulator	0.008209292
REG00014	CXCL2	GSE109604	-1.732172103	knock down	down-regulator	0.006885558
REG00014	GET3	GSE109604	-1.725687394	knock down	down-regulator	7.57E-13
REG00014	DEAF1	GSE109604	-1.720746099	knock down	down-regulator	0.000161376
REG00014	CCDC50	GSE109604	-1.715146618	knock down	down-regulator	0.000270162
REG00014	RHOA	GSE109604	-1.703737595	knock down	down-regulator	0.010270824
REG00014	DCTN4	GSE109604	-1.702854542	knock down	down-regulator	0.011220668
REG00014	AMH	GSE109604	-1.701443352	knock down	down-regulator	0.026853888
REG00014	FUT10	GSE109604	-1.696230335	knock down	down-regulator	0.028230319
REG00014	TTYH3	GSE109604	-1.687607111	knock down	down-regulator	0.000698104
REG00014	HSD11B1	GSE109604	-1.684342708	knock down	down-regulator	0.047754268
REG00014	CDC16	GSE109604	-1.681528507	knock down	down-regulator	1.80E-05
REG00014	B3GALNT2	GSE109604	-1.675518313	knock down	down-regulator	3.48E-13
REG00014	PNMA6A	GSE109604	-1.670015989	knock down	down-regulator	0.023561805
REG00014	ERRFI1	GSE109604	-1.662436897	knock down	down-regulator	0.000823852
REG00014	TLX1	GSE109604	-1.656473382	knock down	down-regulator	0.046829025
REG00014	CCM2	GSE109604	-1.65468225	knock down	down-regulator	0.000897378
REG00014	ATG14	GSE109604	-1.636767522	knock down	down-regulator	1.07E-21
REG00014	ATP6V1B1	GSE109604	-1.634950666	knock down	down-regulator	1.90E-05
REG00014	GLT8D2	GSE109604	-1.632426927	knock down	down-regulator	4.71E-05
REG00014	DCBLD1	GSE109604	-1.631586944	knock down	down-regulator	8.42E-15
REG00014	FZD8	GSE109604	-1.62437247	knock down	down-regulator	0.001980509

REG00014	CCDC71	GSE109604	-1.61565893	knock down	down-regulator	0.004079181
REG00014	NLRP6	GSE109604	-1.599379141	knock down	down-regulator	0.001085257
REG00014	KIF20A	GSE109604	-1.597334101	knock down	down-regulator	9.81E-28
REG00014	GORAB	GSE109604	-1.595605955	knock down	down-regulator	0.043969144
REG00014	VGF	GSE109604	-1.593129909	knock down	down-regulator	5.02E-12
REG00014	MORC4	GSE109604	-1.59288689	knock down	down-regulator	0.017725536
REG00014	ATP6V1E1	GSE109604	-1.5908099	knock down	down-regulator	0.000560268
REG00014	F3	GSE109604	-1.587980002	knock down	down-regulator	0.000687921
REG00014	ESD	GSE109604	-1.58334025	knock down	down-regulator	0.006001287
REG00014	ZNF512B	GSE109604	-1.576901341	knock down	down-regulator	3.84E-15
REG00014	POPDC3	GSE109604	-1.576690658	knock down	down-regulator	5.09E-08
REG00014	PTPRS	GSE109604	-1.573009456	knock down	down-regulator	0.000849014
REG00014	MCIDAS	GSE109604	-1.569980678	knock down	down-regulator	0.037623322
REG00014	STC1	GSE109604	-1.568958242	knock down	down-regulator	0.001481412
REG00014	TFR2	GSE109604	-1.563983108	knock down	down-regulator	0.00282894
REG00014	PAG1	GSE109604	-1.56033538	knock down	down-regulator	0.016803328
REG00014	EXOSC8	GSE109604	-1.559601576	knock down	down-regulator	0.001106739
REG00014	DIP2A	GSE109604	-1.556342651	knock down	down-regulator	0.005571691
REG00014	TRNAU1AP	GSE109604	-1.551824984	knock down	down-regulator	0.000704464
REG00014	SNX7	GSE109604	-1.55092663	knock down	down-regulator	0.00028061
REG00014	ANOS1	GSE109604	-1.545533617	knock down	down-regulator	0.03374287
REG00014	PNPLA3	GSE109604	-1.538770809	knock down	down-regulator	0.028348919
REG00014	GPR68	GSE109604	-1.537733523	knock down	down-regulator	7.49E-05
REG00014	DEXI	GSE109604	-1.535539314	knock down	down-regulator	1.98E-08
REG00014	AHDC1	GSE109604	-1.534913524	knock down	down-regulator	0.000314674
REG00014	ABCA1	GSE109604	-1.523726671	knock down	down-regulator	0.001287544
REG00014	EI24	GSE109604	-1.522120959	knock down	down-regulator	0.000292354
REG00014	OCLN	GSE109604	-1.514142301	knock down	down-regulator	0.000548793
REG00014	ZBTB38	GSE109604	-1.509984919	knock down	down-regulator	2.79E-10
REG00014	ZNF77	GSE109604	-1.509672146	knock down	down-regulator	0.041129273
REG00014	HSPB1	GSE109604	-1.498993256	knock down	down-regulator	1.29E-13
REG00014	ABCA2	GSE109604	-1.49516403	knock down	down-regulator	0.049948987
REG00014	S1PR3	GSE109604	-1.494289062	knock down	down-regulator	2.65E-05
REG00014	YJU2B	GSE109604	-1.489385227	knock down	down-regulator	2.88E-15
REG00014	MRPL54	GSE109604	-1.483793227	knock down	down-regulator	0.002605512
REG00014	GTPBP3	GSE109604	-1.480393224	knock down	down-regulator	5.54E-16
REG00014	PSCA	GSE109604	-1.479013738	knock down	down-regulator	0.000582322
REG00014	VPS41	GSE109604	-1.478599498	knock down	down-regulator	5.98E-26
REG00014	GGTLC2	GSE109604	-1.477232918	knock down	down-regulator	1.21E-14
REG00014	RNF157	GSE109604	-1.471654696	knock down	down-regulator	9.35E-07
REG00014	ERCC5	GSE109604	-1.466724583	knock down	down-regulator	0.003717637
REG00014	COPS3	GSE109604	-1.463480605	knock down	down-regulator	0.004923068
REG00014	YWHAZ	GSE109604	-1.462089052	knock down	down-regulator	0.000654658
REG00014	FOXG1	GSE109604	-1.457032268	knock down	down-regulator	0.000257192
REG00014	COQ4	GSE109604	-1.457024347	knock down	down-regulator	0.049176339
REG00014	CCDC116	GSE109604	-1.456842691	knock down	down-regulator	0.04916878
REG00014	NPDC1	GSE109604	-1.452209215	knock down	down-regulator	0.021684037
REG00014	ZNF544	GSE109604	-1.448776125	knock down	down-regulator	4.52E-06
REG00014	ATP5MJ	GSE109604	-1.441311193	knock down	down-regulator	9.26E-54
REG00014	PPP2CB	GSE109604	-1.439687905	knock down	down-regulator	0.000759738

REG00014	GSTM3	GSE109604	-1.43788708	knock down	down-regulator	5.48E-07
REG00014	VDAC1	GSE109604	-1.437001926	knock down	down-regulator	3.10E-06
REG00014	YPEL5	GSE109604	-1.434863947	knock down	down-regulator	7.20E-06
REG00014	SYTL2	GSE109604	-1.433601547	knock down	down-regulator	0.000599403
REG00014	MSMO1	GSE109604	-1.420944763	knock down	down-regulator	0.016304658
REG00014	C12orf73	GSE109604	-1.420220611	knock down	down-regulator	0.000103395
REG00014	HDHD5	GSE109604	-1.416115962	knock down	down-regulator	5.48E-13
REG00014	DNAJC22	GSE109604	-1.415258488	knock down	down-regulator	5.99E-05
REG00014	NCOA3	GSE109604	-1.405338383	knock down	down-regulator	0.001142211
REG00014	KCNAB3	GSE109604	-1.402	knock down	down-regulator	0.005981
REG00014	CCDC9	GSE109604	-1.401823375	knock down	down-regulator	0.003910737
REG00014	RBBP9	GSE109604	-1.399595965	knock down	down-regulator	0.014671598
REG00014	SLC26A11	GSE109604	-1.398788238	knock down	down-regulator	8.38E-06
REG00014	MYCBP	GSE109604	-1.393704366	knock down	down-regulator	0.032297394
REG00014	F12	GSE109604	-1.391606921	knock down	down-regulator	0.03915955
REG00014	DXO	GSE109604	-1.390376519	knock down	down-regulator	3.55E-06
REG00014	TRIM16	GSE109604	-1.382702156	knock down	down-regulator	0.010422817
REG00014	SORT1	GSE109604	-1.381249968	knock down	down-regulator	0.000519966
REG00014	TCN2	GSE109604	-1.381214581	knock down	down-regulator	0.012590343
REG00014	SPOCD1	GSE109604	-1.377378483	knock down	down-regulator	0.000437244
REG00014	TMEM229B	GSE109604	-1.376287822	knock down	down-regulator	3.22E-05
REG00014	PIN1	GSE109604	-1.375612901	knock down	down-regulator	1.37E-06
REG00014	LRIG3	GSE109604	-1.372260739	knock down	down-regulator	0.002844422
REG00014	MLLT1	GSE109604	-1.370117565	knock down	down-regulator	0.011237318
REG00014	CDV3	GSE109604	-1.367628974	knock down	down-regulator	0.022625788
REG00014	CLK4	GSE109604	-1.365536254	knock down	down-regulator	0.000154368
REG00014	B4GALT2	GSE109604	-1.364817778	knock down	down-regulator	8.64E-07
REG00014	RAB3GAP1	GSE109604	-1.362133346	knock down	down-regulator	0.00022371
REG00014	ZC3H12D	GSE109604	-1.361744406	knock down	down-regulator	4.08E-13
REG00014	TRIM9	GSE109604	-1.361212669	knock down	down-regulator	0.004934184
REG00014	ELK3	GSE109604	-1.359121553	knock down	down-regulator	0.021130552
REG00014	PCED1A	GSE109604	-1.357419131	knock down	down-regulator	0.000788992
REG00014	METTL18	GSE109604	-1.356297553	knock down	down-regulator	0.014495853
REG00014	FOXK2	GSE109604	-1.355769136	knock down	down-regulator	2.97E-07
REG00014	KRT80	GSE109604	-1.354224126	knock down	down-regulator	6.50E-27
REG00014	ZMAT5	GSE109604	-1.353460183	knock down	down-regulator	1.80E-07
REG00014	RALGPS2	GSE109604	-1.352922309	knock down	down-regulator	0.004809893
REG00014	CCDC78	GSE109604	-1.348567546	knock down	down-regulator	0.0028466
REG00014	CC2D1A	GSE109604	-1.346549393	knock down	down-regulator	0.017999625
REG00014	SCNN1D	GSE109604	-1.34620317	knock down	down-regulator	0.002315322
REG00014	SPTLC3	GSE109604	-1.342144993	knock down	down-regulator	0.003686444
REG00014	MYO1D	GSE109604	-1.341752121	knock down	down-regulator	0.034386396
REG00014	APBA3	GSE109604	-1.337923432	knock down	down-regulator	0.044807821
REG00014	PPIA	GSE109604	-1.337757445	knock down	down-regulator	4.39E-05
REG00014	GTPBP8	GSE109604	-1.33366256	knock down	down-regulator	0.042855043
REG00014	CAND1	GSE109604	-1.333278027	knock down	down-regulator	0.003315234
REG00014	ZNF451	GSE109604	-1.332549845	knock down	down-regulator	0.000169144
REG00014	TPM2	GSE109604	-1.330771842	knock down	down-regulator	0.00129011
REG00014	SNX14	GSE109604	-1.324570854	knock down	down-regulator	0.002042564
REG00014	RHOT2	GSE109604	-1.321263045	knock down	down-regulator	0.035592636

REG00014	SNAPC4	GSE109604	-1.320624452	knock down	down-regulator	0.000152023
REG00014	PRPF4	GSE109604	-1.320624452	knock down	down-regulator	0.000152023
REG00014	VPS35L	GSE109604	-1.314051092	knock down	down-regulator	0.00180822
REG00014	DAGLA	GSE109604	-1.312219348	knock down	down-regulator	8.63E-08
REG00014	SLC13A4	GSE109604	-1.309471888	knock down	down-regulator	0.00045628
REG00014	RPL23	GSE109604	-1.307815357	knock down	down-regulator	0.000205118
REG00014	PXDNL	GSE109604	-1.306880854	knock down	down-regulator	0.000465694
REG00014	KLC1	GSE109604	-1.30626651	knock down	down-regulator	5.60E-33
REG00014	PSEN1	GSE109604	-1.298599171	knock down	down-regulator	0.001025656
REG00014	PWWP3A	GSE109604	-1.294257017	knock down	down-regulator	0.00164929
REG00014	LPXN	GSE109604	-1.292708187	knock down	down-regulator	0.028658806
REG00014	BOC	GSE109604	-1.291929703	knock down	down-regulator	5.68E-05
REG00014	STEAP3	GSE109604	-1.289499876	knock down	down-regulator	0.020694325
REG00014	IFIT1	GSE109604	-1.286612025	knock down	down-regulator	2.33E-06
REG00014	TNIP2	GSE109604	-1.285994243	knock down	down-regulator	0.016117723
REG00014	CLCN2	GSE109604	-1.284014999	knock down	down-regulator	0.004021426
REG00014	PCNX3	GSE109604	-1.282144555	knock down	down-regulator	7.02E-05
REG00014	DIP2B	GSE109604	-1.274319665	knock down	down-regulator	7.37E-10
REG00014	RPL24	GSE109604	-1.267642923	knock down	down-regulator	0.000225002
REG00014	ROBO3	GSE109604	-1.267140782	knock down	down-regulator	0.001893407
REG00014	ENGASE	GSE109604	-1.257208688	knock down	down-regulator	2.28E-05
REG00014	PNMA2	GSE109604	-1.257002719	knock down	down-regulator	0.001761747
REG00014	PPP1R35	GSE109604	-1.255075709	knock down	down-regulator	0.001712579
REG00014	PREP	GSE109604	-1.250439285	knock down	down-regulator	0.000686998
REG00014	FBRSL1	GSE109604	-1.247698595	knock down	down-regulator	0.022331409
REG00014	TSC22D2	GSE109604	-1.247089572	knock down	down-regulator	0.008820956
REG00014	TNFRSF10C	GSE109604	-1.245642722	knock down	down-regulator	0.001441388
REG00014	ZSCAN26	GSE109604	-1.241885866	knock down	down-regulator	0.01183607
REG00014	PTX3	GSE109604	-1.240374269	knock down	down-regulator	1.37E-05
REG00014	SPANXA1	GSE109604	-1.238861571	knock down	down-regulator	0.004101448
REG00014	SAP30L	GSE109604	-1.234113308	knock down	down-regulator	0.00093145
REG00014	KANSL1L	GSE109604	-1.230725069	knock down	down-regulator	3.74E-40
REG00014	LRRC17	GSE109604	-1.227381123	knock down	down-regulator	0.00641821
REG00014	TMCC2	GSE109604	-1.224780492	knock down	down-regulator	0.028414173
REG00014	DNASE1	GSE109604	-1.222646879	knock down	down-regulator	4.02E-10
REG00014	MAPK14	GSE109604	-1.222030245	knock down	down-regulator	0.002975491
REG00014	SREBF1	GSE109604	-1.221745524	knock down	down-regulator	0.006531042
REG00014	RASSF1	GSE109604	-1.217609886	knock down	down-regulator	0.00831362
REG00014	F2RL1	GSE109604	-1.217313966	knock down	down-regulator	0.010169289
REG00014	CAPRIN2	GSE109604	-1.213132721	knock down	down-regulator	6.32E-06
REG00014	C15orf62	GSE109604	-1.211948189	knock down	down-regulator	0.009377398
REG00014	ST6GAL1	GSE109604	-1.21163253	knock down	down-regulator	0.039417393
REG00014	VCAM1	GSE109604	-1.210954791	knock down	down-regulator	0.001237274
REG00014	DYRK4	GSE109604	-1.210234396	knock down	down-regulator	0.004101387
REG00014	F2R	GSE109604	-1.209397736	knock down	down-regulator	0.030186934
REG00014	PTGS2	GSE109604	-1.205302367	knock down	down-regulator	0.000387816
REG00014	MAPK8IP1	GSE109604	-1.204418215	knock down	down-regulator	0.003123609
REG00014	ENDOV	GSE109604	-1.203701172	knock down	down-regulator	2.85E-06
REG00014	QTRT1	GSE109604	-1.203423993	knock down	down-regulator	0.007167941
REG00014	NSF	GSE109604	-1.202911541	knock down	down-regulator	2.72E-05

REG00014	DCLK2	GSE109604	-1.202698877	knock down	down-regulator	0.000860711
REG00014	PLAC1	GSE109604	-1.198256265	knock down	down-regulator	5.97E-06
REG00014	NUP37	GSE109604	-1.195776237	knock down	down-regulator	0.021850647
REG00014	THYN1	GSE109604	-1.195736876	knock down	down-regulator	0.000501588
REG00014	COPZ1	GSE109604	-1.193154607	knock down	down-regulator	0.01795319
REG00014	TRPV2	GSE109604	-1.190410944	knock down	down-regulator	0.002058475
REG00014	PSMG3	GSE109604	-1.187201396	knock down	down-regulator	0.001492407
REG00014	BTN3A2	GSE109604	-1.185815105	knock down	down-regulator	2.39E-07
REG00014	MRPS33	GSE109604	-1.183376786	knock down	down-regulator	0.008107272
REG00014	OXCT1	GSE109604	-1.182516487	knock down	down-regulator	7.29E-05
REG00014	FAM53B	GSE109604	-1.182350902	knock down	down-regulator	0.020102851
REG00014	PANK1	GSE109604	-1.181836533	knock down	down-regulator	0.0329188
REG00014	TGFBI	GSE109604	-1.181439109	knock down	down-regulator	0.000472698
REG00014	TENT4A	GSE109604	-1.181017297	knock down	down-regulator	0.013730869
REG00014	EAPP	GSE109604	-1.179371295	knock down	down-regulator	0.013786674
REG00014	QRSL1	GSE109604	-1.177850985	knock down	down-regulator	0.000883287
REG00014	UBE2J1	GSE109604	-1.177712545	knock down	down-regulator	0.045833827
REG00014	WTIP	GSE109604	-1.175256046	knock down	down-regulator	7.31E-15
REG00014	TSC22D1	GSE109604	-1.174352919	knock down	down-regulator	0.020941531
REG00014	ZNF219	GSE109604	-1.174135307	knock down	down-regulator	0.000856488
REG00014	UBL7	GSE109604	-1.172597093	knock down	down-regulator	0.004133397
REG00014	EZH2	GSE109604	-1.170344981	knock down	down-regulator	2.43E-06
REG00014	SPESP1	GSE109604	-1.159964552	knock down	down-regulator	0.002944341
REG00014	DUSP1	GSE109604	-1.156837601	knock down	down-regulator	0.000203289
REG00014	PYROXD2	GSE109604	-1.154692815	knock down	down-regulator	0.001645552
REG00014	BAG5	GSE109604	-1.154059754	knock down	down-regulator	0.002664156
REG00014	STX1B	GSE109604	-1.15352861	knock down	down-regulator	0.041592742
REG00014	MTIF2	GSE109604	-1.147580407	knock down	down-regulator	0.019402318
REG00014	SCPEP1	GSE109604	-1.146880458	knock down	down-regulator	2.72E-06
REG00014	RADX	GSE109604	-1.145070685	knock down	down-regulator	0.000358347
REG00014	DPP7	GSE109604	-1.144290258	knock down	down-regulator	4.25E-06
REG00014	CRY2	GSE109604	-1.141401806	knock down	down-regulator	0.011805262
REG00014	RRP36	GSE109604	-1.140108046	knock down	down-regulator	0.003294637
REG00014	SLC16A3	GSE109604	-1.139249467	knock down	down-regulator	0.003715365
REG00014	IGSF3	GSE109604	-1.135544151	knock down	down-regulator	0.025703852
REG00014	STT3A	GSE109604	-1.13295866	knock down	down-regulator	0.042751779
REG00014	EDA2R	GSE109604	-1.131682034	knock down	down-regulator	2.94E-05
REG00014	ROR1	GSE109604	-1.131590872	knock down	down-regulator	0.002948004
REG00014	SH2B1	GSE109604	-1.129950321	knock down	down-regulator	0.000386869
REG00014	SLC2A8	GSE109604	-1.129030009	knock down	down-regulator	0.000706462
REG00014	PTDSS1	GSE109604	-1.126428172	knock down	down-regulator	0.000918908
REG00014	WSB2	GSE109604	-1.12016036	knock down	down-regulator	1.03E-07
REG00014	RNF20	GSE109604	-1.119549859	knock down	down-regulator	0.000773135
REG00014	FBXO44	GSE109604	-1.118738224	knock down	down-regulator	0.037825762
REG00014	RPS27L	GSE109604	-1.116329407	knock down	down-regulator	0.00214337
REG00014	SYT5	GSE109604	-1.114790079	knock down	down-regulator	0.015805189
REG00014	PPP1R13L	GSE109604	-1.112454817	knock down	down-regulator	0.005072452
REG00014	PELP1	GSE109604	-1.111862719	knock down	down-regulator	0.00202312
REG00014	BTBD3	GSE109604	-1.111857905	knock down	down-regulator	4.20E-05
REG00014	FAM185A	GSE109604	-1.104009974	knock down	down-regulator	0.021907997

REG00014	PSMC1	GSE109604	-1.101035419	knock down	down-regulator	0.000151835
REG00014	PRMT5	GSE109604	-1.100322804	knock down	down-regulator	0.001422893
REG00014	SLC1A3	GSE109604	-1.091786145	knock down	down-regulator	0.004981895
REG00014	XRCC5	GSE109604	-1.085187006	knock down	down-regulator	1.50E-06
REG00014	P4HTM	GSE109604	-1.083198191	knock down	down-regulator	0.00169979
REG00014	FAM174B	GSE109604	-1.081971942	knock down	down-regulator	0.010945523
REG00014	MZT2A	GSE109604	-1.078755419	knock down	down-regulator	0.025670551
REG00014	DLD	GSE109604	-1.078611584	knock down	down-regulator	2.72E-05
REG00014	DPH5	GSE109604	-1.078123068	knock down	down-regulator	0.039797094
REG00014	ARAF	GSE109604	-1.076176533	knock down	down-regulator	0.04897344
REG00014	MYBL2	GSE109604	-1.073421667	knock down	down-regulator	0.009705049
REG00014	SLC66A2	GSE109604	-1.072035603	knock down	down-regulator	0.004962236
REG00014	SCAF1	GSE109604	-1.069114083	knock down	down-regulator	6.90E-05
REG00014	CYLD	GSE109604	-1.067918277	knock down	down-regulator	0.003687696
REG00014	KPNA6	GSE109604	-1.067029383	knock down	down-regulator	6.52E-28
REG00014	GPX8	GSE109604	-1.065780425	knock down	down-regulator	4.74E-05
REG00014	TMEM132B	GSE109604	-1.063628039	knock down	down-regulator	0.001161461
REG00014	SLC25A45	GSE109604	-1.06039569	knock down	down-regulator	0.003245135
REG00014	SPATC1L	GSE109604	-1.060341002	knock down	down-regulator	0.013123949
REG00014	REXO1	GSE109604	-1.056638182	knock down	down-regulator	0.000599503
REG00014	AMZ2	GSE109604	-1.055427826	knock down	down-regulator	2.08E-08
REG00014	SH3BP1	GSE109604	-1.055417249	knock down	down-regulator	0.014744681
REG00014	CENPX	GSE109604	-1.054733501	knock down	down-regulator	0.000769846
REG00014	CCDC61	GSE109604	-1.052756416	knock down	down-regulator	0.008795574
REG00014	PSMB8	GSE109604	-1.050896846	knock down	down-regulator	0.000961433
REG00014	CADPS2	GSE109604	-1.049485454	knock down	down-regulator	0.000318157
REG00014	LSM3	GSE109604	-1.047410843	knock down	down-regulator	0.00406835
REG00014	SLC45A4	GSE109604	-1.046743917	knock down	down-regulator	0.005123449
REG00014	TUBB3	GSE109604	-1.044592876	knock down	down-regulator	0.001180674
REG00014	CDIP1	GSE109604	-1.044464846	knock down	down-regulator	0.022854691
REG00014	SPANXC	GSE109604	-1.043266097	knock down	down-regulator	0.001611371
REG00014	UBE2Z	GSE109604	-1.043168652	knock down	down-regulator	0.008079201
REG00014	QSOX1	GSE109604	-1.041613352	knock down	down-regulator	0.0204348
REG00014	SLC44A4	GSE109604	-1.03720317	knock down	down-regulator	0.001924829
REG00014	NQO1	GSE109604	-1.036887989	knock down	down-regulator	0.038607121
REG00014	CMIP	GSE109604	-1.036734524	knock down	down-regulator	0.00581816
REG00014	MAPK13	GSE109604	-1.03506489	knock down	down-regulator	0.035971478
REG00014	METTL27	GSE109604	-1.034214535	knock down	down-regulator	0.046515769
REG00014	MORN3	GSE109604	-1.031807005	knock down	down-regulator	0.022227162
REG00014	PTGES2	GSE109604	-1.031561413	knock down	down-regulator	0.000547218
REG00014	VPS37C	GSE109604	-1.031407514	knock down	down-regulator	3.13E-10
REG00014	ACSS2	GSE109604	-1.03097434	knock down	down-regulator	0.006487226
REG00014	SNX25	GSE109604	-1.030653118	knock down	down-regulator	0.020813452
REG00014	ECSIT	GSE109604	-1.030128779	knock down	down-regulator	0.008054034
REG00014	ZBTB17	GSE109604	-1.028796749	knock down	down-regulator	2.37E-13
REG00014	TMPRSS9	GSE109604	-1.026859667	knock down	down-regulator	0.01704463
REG00014	P4HA2	GSE109604	-1.024524218	knock down	down-regulator	0.002576492
REG00014	PTOV1	GSE109604	-1.022517711	knock down	down-regulator	0.001818681
REG00014	MON1A	GSE109604	-1.018592079	knock down	down-regulator	0.046824387
REG00014	ENO3	GSE109604	-1.016893198	knock down	down-regulator	0.000157097

REG00014	LMAN2L	GSE109604	-1.015843164	knock down	down-regulator	7.38E-05
REG00014	RASSF4	GSE109604	-1.014546494	knock down	down-regulator	0.016828126
REG00014	CXCL1	GSE109604	-1.014051374	knock down	down-regulator	0.001481532
REG00014	ATAT1	GSE109604	-1.012189154	knock down	down-regulator	0.012820536
REG00014	CNOT10	GSE109604	-1.010315082	knock down	down-regulator	0.042272978
REG00014	SPANXA2	GSE109604	-1.010044907	knock down	down-regulator	0.004001614
REG00014	FAM131A	GSE109604	-1.009724211	knock down	down-regulator	0.045231948
REG00014	MVB12A	GSE109604	-1.009112586	knock down	down-regulator	0.001141858
REG00014	INSYN2B	GSE109604	-1.002840789	knock down	down-regulator	0.001044428
REG00014	HELB	GSE109604	-1.002196952	knock down	down-regulator	3.46E-05
REG00014	PRDX1	GSE109604	-1.000217154	knock down	down-regulator	0.002260173
REG00014	CEP170B	GSE109604	-0.999435672	knock down	down-regulator	0.044768249
REG00014	WWC1	GSE109604	-0.999336664	knock down	down-regulator	0.00619451
REG00014	PEMT	GSE109604	-0.999294666	knock down	down-regulator	0.003426896
REG00014	EIF1AX	GSE109604	-0.998458198	knock down	down-regulator	6.85E-05
REG00014	MAN2B2	GSE109604	-0.997076482	knock down	down-regulator	0.002120407
REG00014	SMTN	GSE109604	-0.99521155	knock down	down-regulator	0.001405894
REG00014	EGR1	GSE109604	-0.994009595	knock down	down-regulator	0.024485064
REG00014	COPE	GSE109604	-0.994005048	knock down	down-regulator	0.008087462
REG00014	C20orf144	GSE109604	-0.992013965	knock down	down-regulator	0.002948292
REG00014	TSEN54	GSE109604	-0.99182221	knock down	down-regulator	0.017782687
REG00014	C21orf58	GSE109604	-0.990681919	knock down	down-regulator	0.008032219
REG00014	RPL36AL	GSE109604	-0.989584265	knock down	down-regulator	2.23E-05
REG00014	WASHC1	GSE109604	-0.98945719	knock down	down-regulator	2.09E-06
REG00014	RNF182	GSE109604	-0.987670444	knock down	down-regulator	0.00026495
REG00014	PKD1	GSE109604	-0.987219284	knock down	down-regulator	0.002619733
REG00014	LRP8	GSE109604	-0.985947348	knock down	down-regulator	0.020447443
REG00014	ICAM5	GSE109604	-0.985186325	knock down	down-regulator	7.33E-06
REG00014	MRPS15	GSE109604	-0.985151687	knock down	down-regulator	0.001904209
REG00014	CCDC80	GSE109604	-0.984734286	knock down	down-regulator	0.012626987
REG00014	PGPEP1	GSE109604	-0.982330265	knock down	down-regulator	0.005078603
REG00014	ATP5MC1	GSE109604	-0.98227801	knock down	down-regulator	0.001672979
REG00014	RABL6	GSE109604	-0.980290383	knock down	down-regulator	0.004161559
REG00014	SLC30A6	GSE109604	-0.97998013	knock down	down-regulator	0.003920172
REG00014	SPOPL	GSE109604	-0.978292419	knock down	down-regulator	0.039978937
REG00014	WDR27	GSE109604	-0.977615444	knock down	down-regulator	0.00640335
REG00014	PRNP	GSE109604	-0.977165786	knock down	down-regulator	0.013877809
REG00014	ASB9	GSE109604	-0.977124232	knock down	down-regulator	0.020384025
REG00014	DUSP4	GSE109604	-0.976413762	knock down	down-regulator	0.009522209
REG00014	NPC1	GSE109604	-0.975216671	knock down	down-regulator	0.014861819
REG00014	ARHGEF1	GSE109604	-0.973946055	knock down	down-regulator	0.031674294
REG00014	RAPH1	GSE109604	-0.971728643	knock down	down-regulator	0.00333165
REG00014	SP140	GSE109604	-0.971664667	knock down	down-regulator	0.003629958
REG00014	SERINC2	GSE109604	-0.969535862	knock down	down-regulator	0.004337706
REG00014	DDR1	GSE109604	-0.967383724	knock down	down-regulator	1.20E-06
REG00014	SCAP	GSE109604	-0.964759091	knock down	down-regulator	0.049787439
REG00014	CLDN11	GSE109604	-0.964001814	knock down	down-regulator	0.009622913
REG00014	PIGZ	GSE109604	-0.962160495	knock down	down-regulator	2.19E-05
REG00014	RFC5	GSE109604	-0.961899371	knock down	down-regulator	0.000172351
REG00014	ZNF76	GSE109604	-0.961005915	knock down	down-regulator	0.000815429

REG00014	PDLIM7	GSE109604	-0.959273654	knock down	down-regulator	0.000977316
REG00014	CDPF1	GSE109604	-0.958671597	knock down	down-regulator	0.001118319
REG00014	GIGYF2	GSE109604	-0.957967007	knock down	down-regulator	1.57E-29
REG00014	AKTIP	GSE109604	-0.957360743	knock down	down-regulator	2.22E-08
REG00014	CMTM1	GSE109604	-0.956498246	knock down	down-regulator	0.037059305
REG00014	RABGGTB	GSE109604	-0.954599543	knock down	down-regulator	0.001218696
REG00014	PHPT1	GSE109604	-0.953160409	knock down	down-regulator	0.000364208
REG00014	CDK10	GSE109604	-0.952645848	knock down	down-regulator	0.017945788
REG00014	PAMR1	GSE109604	-0.951909989	knock down	down-regulator	0.001843336
REG00014	BLOC1S5	GSE109604	-0.950293591	knock down	down-regulator	2.90E-08
REG00014	EXTL2	GSE109604	-0.950151066	knock down	down-regulator	0.009385839
REG00014	NUP62	GSE109604	-0.949879525	knock down	down-regulator	0.008024951
REG00014	NPAT	GSE109604	-0.946561385	knock down	down-regulator	0.000130001
REG00014	SF3A3	GSE109604	-0.945713911	knock down	down-regulator	0.003110066
REG00014	INPP5E	GSE109604	-0.945698394	knock down	down-regulator	4.44E-05
REG00014	ZNF768	GSE109604	-0.945624037	knock down	down-regulator	3.44E-09
REG00014	CSNK1G2	GSE109604	-0.945527193	knock down	down-regulator	6.77E-06
REG00014	SNX24	GSE109604	-0.944698476	knock down	down-regulator	0.008717134
REG00014	RNF151	GSE109604	-0.944522024	knock down	down-regulator	4.45E-05
REG00014	UHRF2	GSE109604	-0.943069556	knock down	down-regulator	0.007742822
REG00014	KCNC4	GSE109604	-0.9426	knock down	down-regulator	1.01E-08
REG00014	CUEDC2	GSE109604	-0.942483304	knock down	down-regulator	0.009390211
REG00014	DCAF6	GSE109604	-0.940957151	knock down	down-regulator	0.00430643
REG00014	RAPGEFL1	GSE109604	-0.940810051	knock down	down-regulator	0.000209864
REG00014	RPP25L	GSE109604	-0.940662646	knock down	down-regulator	9.18E-06
REG00014	OCRL	GSE109604	-0.939756452	knock down	down-regulator	0.000303981
REG00014	CLTCL1	GSE109604	-0.939060796	knock down	down-regulator	0.032053905
REG00014	PALS2	GSE109604	-0.938696445	knock down	down-regulator	0.000515111
REG00014	ZNF555	GSE109604	-0.937654416	knock down	down-regulator	0.001034961
REG00014	ARC	GSE109604	-0.936503769	knock down	down-regulator	0.002249108
REG00014	GPSM1	GSE109604	-0.936479592	knock down	down-regulator	1.05E-06
REG00014	CLDN15	GSE109604	-0.935270901	knock down	down-regulator	0.001562422
REG00014	MFAP4	GSE109604	-0.935103104	knock down	down-regulator	0.002535876
REG00014	NAPG	GSE109604	-0.934576902	knock down	down-regulator	1.09E-06
REG00014	GPR63	GSE109604	-0.933715336	knock down	down-regulator	5.33E-11
REG00014	GPX1	GSE109604	-0.932624091	knock down	down-regulator	0.030219046
REG00014	RAP1GDS1	GSE109604	-0.93197248	knock down	down-regulator	0.01784317
REG00014	ST3GAL5	GSE109604	-0.928687272	knock down	down-regulator	0.037539459
REG00014	CGB5	GSE109604	-0.928275769	knock down	down-regulator	0.00288761
REG00014	PEX19	GSE109604	-0.927302044	knock down	down-regulator	0.00184461
REG00014	CTIF	GSE109604	-0.92711918	knock down	down-regulator	0.000115621
REG00014	OMA1	GSE109604	-0.926358735	knock down	down-regulator	3.18E-05
REG00014	LAMTOR4	GSE109604	-0.924118704	knock down	down-regulator	4.72E-10
REG00014	FOSL2	GSE109604	-0.923480305	knock down	down-regulator	4.02E-09
REG00014	SPNS2	GSE109604	-0.923068686	knock down	down-regulator	0.008247336
REG00014	PKN3	GSE109604	-0.921464962	knock down	down-regulator	0.003298002
REG00014	BAIAP2L2	GSE109604	-0.92117557	knock down	down-regulator	3.60E-21
REG00014	GART	GSE109604	-0.920190616	knock down	down-regulator	0.000279024
REG00014	RPS6KL1	GSE109604	-0.920065835	knock down	down-regulator	0.002539023
REG00014	RWDD2B	GSE109604	-0.91999129	knock down	down-regulator	0.004235968

REG00014	SPACA6	GSE109604	-0.919205523	knock down	down-regulator	0.006594564
REG00014	STMN1	GSE109604	-0.917604403	knock down	down-regulator	0.01053835
REG00014	GNG5	GSE109604	-0.917121986	knock down	down-regulator	0.049750954
REG00014	EML4	GSE109604	-0.916605279	knock down	down-regulator	0.003128033
REG00014	RAB40B	GSE109604	-0.915683	knock down	down-regulator	0.00468319
REG00014	CCDC105	GSE109604	-0.912738327	knock down	down-regulator	0.011346701
REG00014	CCDC92	GSE109604	-0.912567883	knock down	down-regulator	0.015835261
REG00014	RPL18	GSE109604	-0.911902401	knock down	down-regulator	2.53E-06
REG00014	PIN4	GSE109604	-0.911902401	knock down	down-regulator	2.53E-06
REG00014	GPC1	GSE109604	-0.911726213	knock down	down-regulator	3.38E-05
REG00014	ZP3	GSE109604	-0.910109208	knock down	down-regulator	0.041452851
REG00014	ARAP3	GSE109604	-0.907980339	knock down	down-regulator	0.028619159
REG00014	SLC31A1	GSE109604	-0.907368028	knock down	down-regulator	0.000843331
REG00014	CXCL3	GSE109604	-0.906610178	knock down	down-regulator	0.015352465
REG00014	WNT3	GSE109604	-0.902993123	knock down	down-regulator	0.017136573
REG00014	RMND5A	GSE109604	-0.900962191	knock down	down-regulator	0.002021286
REG00014	NUFIP1	GSE109604	-0.89903216	knock down	down-regulator	5.18E-05
REG00014	IL12A	GSE109604	-0.896774379	knock down	down-regulator	0.00045239
REG00014	QTRT2	GSE109604	-0.896512098	knock down	down-regulator	0.013068426
REG00014	SFXN3	GSE109604	-0.894894699	knock down	down-regulator	4.73E-06
REG00014	NRP1	GSE109604	-0.894894699	knock down	down-regulator	4.73E-06
REG00014	RAB6A	GSE109604	-0.894602478	knock down	down-regulator	0.007543147
REG00014	GSTZ1	GSE109604	-0.892416907	knock down	down-regulator	0.000437992
REG00014	TP63	GSE109604	-0.889089253	knock down	down-regulator	0.008439192
REG00014	PPP1R15A	GSE109604	-0.888504273	knock down	down-regulator	0.003070308
REG00014	NUTM2B	GSE109604	-0.886647102	knock down	down-regulator	0.002533692
REG00014	PLK2	GSE109604	-0.886217882	knock down	down-regulator	0.023292884
REG00014	GGT1	GSE109604	-0.884788375	knock down	down-regulator	2.18E-11
REG00014	CANT1	GSE109604	-0.883912644	knock down	down-regulator	0.000120308
REG00014	PAPLN	GSE109604	-0.882688152	knock down	down-regulator	0.000918282
REG00014	PPP1R27	GSE109604	-0.882249793	knock down	down-regulator	0.005385513
REG00014	SUPT20H	GSE109604	-0.879074025	knock down	down-regulator	0.00547778
REG00014	PTK2B	GSE109604	-0.878177702	knock down	down-regulator	0.012999572
REG00014	PPP1R3G	GSE109604	-0.876872287	knock down	down-regulator	0.006506168
REG00014	MORF4L2	GSE109604	-0.875732965	knock down	down-regulator	0.007801364
REG00014	CIC	GSE109604	-0.875269802	knock down	down-regulator	0.000773762
REG00014	NFIB	GSE109604	-0.873482823	knock down	down-regulator	8.06E-05
REG00014	SPATA2	GSE109604	-0.873178114	knock down	down-regulator	0.030362382
REG00014	SLC37A2	GSE109604	-0.872652605	knock down	down-regulator	0.0170598
REG00014	NIM1K	GSE109604	-0.869262307	knock down	down-regulator	3.50E-05
REG00014	TRAM2	GSE109604	-0.86799517	knock down	down-regulator	0.004501787
REG00014	ACTR3C	GSE109604	-0.867745619	knock down	down-regulator	0.01083994
REG00014	PAQR7	GSE109604	-0.867565876	knock down	down-regulator	0.008044405
REG00014	ADM5	GSE109604	-0.867505995	knock down	down-regulator	2.12E-09
REG00014	FAM13B	GSE109604	-0.86606171	knock down	down-regulator	0.00046037
REG00014	C19orf47	GSE109604	-0.865784195	knock down	down-regulator	0.006499632
REG00014	NGF	GSE109604	-0.863853478	knock down	down-regulator	3.01E-05
REG00014	TGM2	GSE109604	-0.863840587	knock down	down-regulator	0.010771514
REG00014	TEDC2	GSE109604	-0.862931233	knock down	down-regulator	0.015251839
REG00014	MMAB	GSE109604	-0.862746418	knock down	down-regulator	1.40E-06

REG00014	DKC1	GSE109604	-0.862261549	knock down	down-regulator	0.029275821
REG00014	DOK3	GSE109604	-0.862073333	knock down	down-regulator	0.023356395
REG00014	H2AC11	GSE109604	-0.861263048	knock down	down-regulator	4.38E-08
REG00014	PTPN6	GSE109604	-0.860180875	knock down	down-regulator	0.002928893
REG00014	NKX2-6	GSE109604	-0.858465693	knock down	down-regulator	0.000143265
REG00014	DHX9	GSE109604	-0.856285012	knock down	down-regulator	4.95E-06
REG00014	C5orf22	GSE109604	-0.856240194	knock down	down-regulator	0.036457154
REG00014	OLFML2B	GSE109604	-0.855271774	knock down	down-regulator	0.011945505
REG00014	MYH7B	GSE109604	-0.855145581	knock down	down-regulator	0.009490454
REG00014	NCF1	GSE109604	-0.853135533	knock down	down-regulator	2.84E-06
REG00014	SS18L1	GSE109604	-0.851683791	knock down	down-regulator	0.023183337
REG00014	SNX30	GSE109604	-0.850669217	knock down	down-regulator	0.000925687
REG00014	IFITM2	GSE109604	-0.850311498	knock down	down-regulator	0.00474999
REG00014	RPS3A	GSE109604	-0.847497591	knock down	down-regulator	0.002738566
REG00014	APOL2	GSE109604	-0.847432217	knock down	down-regulator	0.011538846
REG00014	DAAM2	GSE109604	-0.847047368	knock down	down-regulator	0.00234042
REG00014	PRSS23	GSE109604	-0.846011069	knock down	down-regulator	0.032003865
REG00014	KLHL35	GSE109604	-0.845537271	knock down	down-regulator	6.43E-22
REG00014	RAB1A	GSE109604	-0.84528228	knock down	down-regulator	0.007103271
REG00014	ORAI2	GSE109604	-0.84494035	knock down	down-regulator	0.000100632
REG00014	EML2	GSE109604	-0.843885793	knock down	down-regulator	0.000371008
REG00014	FAF2	GSE109604	-0.843115357	knock down	down-regulator	2.41E-23
REG00014	FAM83G	GSE109604	-0.841970406	knock down	down-regulator	0.022129056
REG00014	ELOC	GSE109604	-0.841166641	knock down	down-regulator	0.044577638
REG00014	ZCCHC17	GSE109604	-0.839647946	knock down	down-regulator	2.50E-07
REG00014	PSMD9	GSE109604	-0.837845604	knock down	down-regulator	0.002923655
REG00014	MED21	GSE109604	-0.835942221	knock down	down-regulator	0.00973764
REG00014	PDCD6	GSE109604	-0.835833622	knock down	down-regulator	0.007828069
REG00014	RALGDS	GSE109604	-0.834349408	knock down	down-regulator	0.004817459
REG00014	GLCE	GSE109604	-0.83422498	knock down	down-regulator	0.020079774
REG00014	AGO1	GSE109604	-0.833754173	knock down	down-regulator	0.025301618
REG00014	NT5C2	GSE109604	-0.831771533	knock down	down-regulator	0.002392458
REG00014	OAT	GSE109604	-0.83174285	knock down	down-regulator	0.006131815
REG00014	RPL29	GSE109604	-0.830680299	knock down	down-regulator	0.000452238
REG00014	VPS16	GSE109604	-0.830610683	knock down	down-regulator	0.00021938
REG00014	SH3BP2	GSE109604	-0.829258032	knock down	down-regulator	0.004877783
REG00014	NPIPB11	GSE109604	-0.829169045	knock down	down-regulator	0.010089305
REG00014	HIRA	GSE109604	-0.828948334	knock down	down-regulator	0.000728376
REG00014	NR3C1	GSE109604	-0.828221258	knock down	down-regulator	4.66E-05
REG00014	GALNT7	GSE109604	-0.826401496	knock down	down-regulator	4.06E-05
REG00014	RPS6	GSE109604	-0.825115803	knock down	down-regulator	4.56E-05
REG00014	ZNF106	GSE109604	-0.82478183	knock down	down-regulator	1.40E-08
REG00014	APBA2	GSE109604	-0.824749738	knock down	down-regulator	2.35E-08
REG00014	GSE1	GSE109604	-0.824339855	knock down	down-regulator	2.51E-10
REG00014	OSCP1	GSE109604	-0.823678392	knock down	down-regulator	0.00603078
REG00014	XPO4	GSE109604	-0.823045583	knock down	down-regulator	0.047760581
REG00014	MAFB	GSE109604	-0.818982208	knock down	down-regulator	0.008561678
REG00014	GPRIN1	GSE109604	-0.818636372	knock down	down-regulator	0.032718164
REG00014	RAX2	GSE109604	-0.818536263	knock down	down-regulator	0.000911893
REG00014	STMN3	GSE109604	-0.81779037	knock down	down-regulator	0.004243312

REG00014	BBLN	GSE109604	-0.817246389	knock down	down-regulator	1.51E-05
REG00014	SHANK2	GSE109604	-0.817045776	knock down	down-regulator	3.64E-05
REG00014	SLC49A4	GSE109604	-0.816969151	knock down	down-regulator	0.012038174
REG00014	KCNG1	GSE109604	-0.816889144	knock down	down-regulator	1.53E-08
REG00014	GJB2	GSE109604	-0.815996677	knock down	down-regulator	0.003169932
REG00014	PLRG1	GSE109604	-0.814368557	knock down	down-regulator	0.002192003
REG00014	RPP30	GSE109604	-0.812262681	knock down	down-regulator	0.008476529
REG00014	RAC3	GSE109604	-0.812070503	knock down	down-regulator	0.005467682
REG00014	LAMTOR2	GSE109604	-0.811154934	knock down	down-regulator	1.38E-26
REG00014	PYY	GSE109604	-0.811088797	knock down	down-regulator	0.012090268
REG00014	ID2	GSE109604	-0.810976152	knock down	down-regulator	0.000342284
REG00014	GADD45A	GSE109604	-0.810872975	knock down	down-regulator	4.78E-06
REG00014	ARNTL	GSE109604	-0.810048663	knock down	down-regulator	0.025052135
REG00014	RILPL2	GSE109604	-0.809997393	knock down	down-regulator	0.003372432
REG00014	CEPT1	GSE109604	-0.808987773	knock down	down-regulator	0.020817538
REG00014	ZNF717	GSE109604	-0.807985924	knock down	down-regulator	0.032138405
REG00014	RASSF3	GSE109604	-0.807564063	knock down	down-regulator	0.006314445
REG00014	STPG1	GSE109604	-0.805816877	knock down	down-regulator	0.012533333
REG00014	RNF146	GSE109604	-0.803240923	knock down	down-regulator	0.000285754
REG00014	MFF	GSE109604	-0.801718532	knock down	down-regulator	0.003195557
REG00014	SERPIND1	GSE109604	-0.80129453	knock down	down-regulator	0.018085456
REG00014	EIF3M	GSE109604	-0.799758777	knock down	down-regulator	0.006494918
REG00014	ARCN1	GSE109604	-0.799165055	knock down	down-regulator	9.14E-05
REG00014	EMC3	GSE109604	-0.795460241	knock down	down-regulator	0.009876649
REG00014	TBX2	GSE109604	-0.795414407	knock down	down-regulator	0.028055652
REG00014	PPP1R16A	GSE109604	-0.794825345	knock down	down-regulator	0.018520722
REG00014	CFAP410	GSE109604	-0.792518868	knock down	down-regulator	0.013744339
REG00014	RPL6	GSE109604	-0.790844485	knock down	down-regulator	0.032403155
REG00014	FAM86B2	GSE109604	-0.790392164	knock down	down-regulator	0.003822285
REG00014	PIGA	GSE109604	-0.790318701	knock down	down-regulator	0.020364453
REG00014	AIDA	GSE109604	-0.788331139	knock down	down-regulator	3.64E-19
REG00014	RABGAP1	GSE109604	-0.788115279	knock down	down-regulator	0.005599921
REG00014	ZNF668	GSE109604	-0.786559956	knock down	down-regulator	9.98E-06
REG00014	INAFM2	GSE109604	-0.786269607	knock down	down-regulator	0.019594724
REG00014	HDAC7	GSE109604	-0.785885794	knock down	down-regulator	0.00178503
REG00014	STARD7	GSE109604	-0.785127246	knock down	down-regulator	0.018061081
REG00014	RGL1	GSE109604	-0.782034485	knock down	down-regulator	0.024317786
REG00014	RTCB	GSE109604	-0.781440078	knock down	down-regulator	2.94E-05
REG00014	PHYHIP	GSE109604	-0.781034537	knock down	down-regulator	0.000731123
REG00014	FBXL14	GSE109604	-0.780983084	knock down	down-regulator	8.30E-05
REG00014	RPL27A	GSE109604	-0.78010346	knock down	down-regulator	2.64E-07
REG00014	RRAGD	GSE109604	-0.779568199	knock down	down-regulator	0.000253903
REG00014	PNPLA6	GSE109604	-0.779277483	knock down	down-regulator	0.012682248
REG00014	EPS15	GSE109604	-0.778978149	knock down	down-regulator	0.029215596
REG00014	PPP1R12C	GSE109604	-0.778926819	knock down	down-regulator	0.013961984
REG00014	PTP4A3	GSE109604	-0.778902186	knock down	down-regulator	0.001382764
REG00014	ERAP1	GSE109604	-0.778682658	knock down	down-regulator	7.52E-05
REG00014	MVK	GSE109604	-0.777194676	knock down	down-regulator	0.018486936
REG00014	ANKRD1	GSE109604	-0.777144169	knock down	down-regulator	0.001785154
REG00014	SAMD8	GSE109604	-0.776990162	knock down	down-regulator	0.007458728

REG00014	SH2B2	GSE109604	-0.776697384	knock down	down-regulator	2.19E-08
REG00014	ARL2	GSE109604	-0.776337857	knock down	down-regulator	0.003134376
REG00014	DOT1L	GSE109604	-0.775843657	knock down	down-regulator	0.017924765
REG00014	PSMB9	GSE109604	-0.774695055	knock down	down-regulator	0.006247764
REG00014	SGSH	GSE109604	-0.774654973	knock down	down-regulator	8.12E-07
REG00014	PPP1R14B	GSE109604	-0.774552196	knock down	down-regulator	0.003025678
REG00014	CHST4	GSE109604	-0.773254647	knock down	down-regulator	0.00438832
REG00014	BABAM2	GSE109604	-0.77244222	knock down	down-regulator	0.006938079
REG00014	IFT22	GSE109604	-0.772319778	knock down	down-regulator	1.88E-05
REG00014	PAQR4	GSE109604	-0.77227381	knock down	down-regulator	0.014617859
REG00014	GGCX	GSE109604	-0.771796472	knock down	down-regulator	4.73E-08
REG00014	BUD31	GSE109604	-0.77077341	knock down	down-regulator	5.64E-07
REG00014	CD109	GSE109604	-0.770224302	knock down	down-regulator	0.048918506
REG00014	FAM72D	GSE109604	-0.769565759	knock down	down-regulator	0.007018043
REG00014	FADS3	GSE109604	-0.768383314	knock down	down-regulator	0.031510622
REG00014	AKR1C3	GSE109604	-0.768354672	knock down	down-regulator	5.26E-17
REG00014	PCBP3	GSE109604	-0.768318747	knock down	down-regulator	0.034176146
REG00014	PARP4	GSE109604	-0.768224047	knock down	down-regulator	0.004102805
REG00014	CSK	GSE109604	-0.767623419	knock down	down-regulator	0.009765556
REG00014	EVA1A	GSE109604	-0.766827436	knock down	down-regulator	0.039336945
REG00014	RASD1	GSE109604	-0.766226106	knock down	down-regulator	0.041866029
REG00014	MACROD1	GSE109604	-0.766149647	knock down	down-regulator	0.01885541
REG00014	H3C4	GSE109604	-0.765356168	knock down	down-regulator	7.39E-11
REG00014	NBAS	GSE109604	-0.7651925	knock down	down-regulator	0.00655608
REG00014	STRADA	GSE109604	-0.76382117	knock down	down-regulator	0.023071218
REG00014	FAM229A	GSE109604	-0.762942959	knock down	down-regulator	0.047913283
REG00014	CPSF6	GSE109604	-0.762856848	knock down	down-regulator	0.003527953
REG00014	PUS3	GSE109604	-0.762701997	knock down	down-regulator	0.007087386
REG00014	SP9	GSE109604	-0.762626319	knock down	down-regulator	0.009686208
REG00014	PGS1	GSE109604	-0.761260848	knock down	down-regulator	0.010058749
REG00014	IQGAP1	GSE109604	-0.759957921	knock down	down-regulator	6.92E-05
REG00014	RSRC1	GSE109604	-0.759225094	knock down	down-regulator	1.80E-07
REG00014	SNAPC5	GSE109604	-0.758955997	knock down	down-regulator	0.004246557
REG00014	ZNF23	GSE109604	-0.758544437	knock down	down-regulator	0.004045657
REG00014	ZNF573	GSE109604	-0.758359125	knock down	down-regulator	2.21E-08
REG00014	SOX15	GSE109604	-0.758132675	knock down	down-regulator	0.005199507
REG00014	CCT6B	GSE109604	-0.755040848	knock down	down-regulator	0.019096391
REG00014	ACTN1	GSE109604	-0.754470587	knock down	down-regulator	0.003068467
REG00014	ZNF557	GSE109604	-0.754265037	knock down	down-regulator	5.51E-06
REG00014	TPST1	GSE109604	-0.753152912	knock down	down-regulator	0.000178016
REG00014	ONECUT3	GSE109604	-0.753058472	knock down	down-regulator	0.015026598
REG00014	ADGRF5	GSE109604	-0.75277323	knock down	down-regulator	0.000739117
REG00014	FAM86B1	GSE109604	-0.751602665	knock down	down-regulator	0.030328484
REG00014	ISYNA1	GSE109604	-0.751105106	knock down	down-regulator	0.000333409
REG00014	DARS1	GSE109604	-0.75073882	knock down	down-regulator	0.004158742
REG00014	EPS8L1	GSE109604	-0.749013295	knock down	down-regulator	0.00672561
REG00014	PDP1	GSE109604	-0.748907118	knock down	down-regulator	0.013596039
REG00014	RNPS1	GSE109604	-0.748448791	knock down	down-regulator	0.002715245
REG00014	GATA2	GSE109604	-0.747490646	knock down	down-regulator	0.000615555
REG00014	AFAP1L2	GSE109604	-0.747221282	knock down	down-regulator	5.89E-05

REG00014	RPL27	GSE109604	-0.746835903	knock down	down-regulator	0.003773251
REG00014	CYP11A1	GSE109604	-0.746275008	knock down	down-regulator	0.007665128
REG00014	NT5M	GSE109604	-0.746005419	knock down	down-regulator	0.001148641
REG00014	TTC33	GSE109604	-0.742293904	knock down	down-regulator	0.02626802
REG00014	SH3BP5L	GSE109604	-0.741972665	knock down	down-regulator	0.018586273
REG00014	PRR7	GSE109604	-0.740893793	knock down	down-regulator	0.001778327
REG00014	CLBA1	GSE109604	-0.740101522	knock down	down-regulator	0.026716933
REG00014	FCSK	GSE109604	-0.739945553	knock down	down-regulator	0.000187886
REG00014	NDUFAF7	GSE109604	-0.739719074	knock down	down-regulator	0.024141338
REG00014	AHR	GSE109604	-0.73950552	knock down	down-regulator	5.83E-06
REG00014	PARP12	GSE109604	-0.739466565	knock down	down-regulator	0.005828401
REG00014	MGRN1	GSE109604	-0.738685925	knock down	down-regulator	2.07E-05
REG00014	PRR13	GSE109604	-0.734426608	knock down	down-regulator	0.001171849
REG00014	TMEM187	GSE109604	-0.734215732	knock down	down-regulator	0.012794261
REG00014	ABHD5	GSE109604	-0.733744002	knock down	down-regulator	0.000418655
REG00014	PIGM	GSE109604	-0.7331701	knock down	down-regulator	0.017583446
REG00014	SP110	GSE109604	-0.732738125	knock down	down-regulator	0.005225153
REG00014	ABTB3	GSE109604	-0.732647257	knock down	down-regulator	0.047245044
REG00014	MEX3C	GSE109604	-0.732059362	knock down	down-regulator	0.000220877
REG00014	TSGA10IP	GSE109604	-0.73109457	knock down	down-regulator	0.029057193
REG00014	TRIM46	GSE109604	-0.730975376	knock down	down-regulator	0.045696226
REG00014	SORCS2	GSE109604	-0.730894968	knock down	down-regulator	0.003865957
REG00014	ENTPD3	GSE109604	-0.730469395	knock down	down-regulator	0.000661459
REG00014	GPR173	GSE109604	-0.729814934	knock down	down-regulator	0.013629286
REG00014	UQCRFS1	GSE109604	-0.729453207	knock down	down-regulator	0.024138131
REG00014	PSME2	GSE109604	-0.729318004	knock down	down-regulator	0.011262544
REG00014	RNF166	GSE109604	-0.728771825	knock down	down-regulator	0.003086001
REG00014	DTX2	GSE109604	-0.728413159	knock down	down-regulator	0.001451893
REG00014	PDCL3	GSE109604	-0.727480993	knock down	down-regulator	0.00045073
REG00014	WASHC2C	GSE109604	-0.725023707	knock down	down-regulator	0.001565675
REG00014	SYNGAP1	GSE109604	-0.724590385	knock down	down-regulator	0.045467096
REG00014	SKIL	GSE109604	-0.724373979	knock down	down-regulator	0.001351246
REG00014	WEE1	GSE109604	-0.723580552	knock down	down-regulator	0.002560046
REG00014	NKX6-1	GSE109604	-0.723428726	knock down	down-regulator	0.015767755
REG00014	LYN	GSE109604	-0.720501911	knock down	down-regulator	0.004361129
REG00014	EIF2S3	GSE109604	-0.720455905	knock down	down-regulator	0.021455105
REG00014	SMC5	GSE109604	-0.718733108	knock down	down-regulator	0.041377137
REG00014	RHOB	GSE109604	-0.718023605	knock down	down-regulator	0.000874376
REG00014	ESPL1	GSE109604	-0.717648662	knock down	down-regulator	0.046720786
REG00014	ZMIZ2	GSE109604	-0.717549969	knock down	down-regulator	0.025730398
REG00014	RAB27B	GSE109604	-0.716345064	knock down	down-regulator	0.018065246
REG00014	ZNF608	GSE109604	-0.716076129	knock down	down-regulator	9.17E-06
REG00014	GBP1	GSE109604	-0.716028293	knock down	down-regulator	2.79E-30
REG00014	DNAJA2	GSE109604	-0.71586747	knock down	down-regulator	0.00109067
REG00014	CLDN4	GSE109604	-0.715587218	knock down	down-regulator	0.009617474
REG00014	CREBRF	GSE109604	-0.71519378	knock down	down-regulator	0.006187067
REG00014	YEATS4	GSE109604	-0.714278711	knock down	down-regulator	2.90E-05
REG00014	GSTCD	GSE109604	-0.714148619	knock down	down-regulator	0.001863141
REG00014	HABP4	GSE109604	-0.713992467	knock down	down-regulator	0.003979312
REG00014	C11orf68	GSE109604	-0.71397579	knock down	down-regulator	0.037685801

REG00014	PER2	GSE109604	-0.713086905	knock down	down-regulator	0.001435055
REG00014	SLC1A4	GSE109604	-0.712503588	knock down	down-regulator	0.01238884
REG00014	RNF138	GSE109604	-0.711979291	knock down	down-regulator	0.000351666
REG00014	SLC6A9	GSE109604	-0.711925269	knock down	down-regulator	0.020852639
REG00014	XBP1	GSE109604	-0.711646752	knock down	down-regulator	6.46E-07
REG00014	PROB1	GSE109604	-0.711632907	knock down	down-regulator	0.017664424
REG00014	SOWAHC	GSE109604	-0.710879045	knock down	down-regulator	0.006326896
REG00014	PABPC1L	GSE109604	-0.710700887	knock down	down-regulator	0.0342603
REG00014	DNAAF8	GSE109604	-0.710373252	knock down	down-regulator	0.006311843
REG00014	SLC9A1	GSE109604	-0.710326426	knock down	down-regulator	0.00950799
REG00014	PUS1	GSE109604	-0.708399165	knock down	down-regulator	0.026348706
REG00014	FNIP1	GSE109604	-0.707964138	knock down	down-regulator	0.009848759
REG00014	ANXA7	GSE109604	-0.707821957	knock down	down-regulator	0.003610293
REG00014	PRRT3	GSE109604	-0.707104732	knock down	down-regulator	0.018389162
REG00014	EMP1	GSE109604	-0.705848325	knock down	down-regulator	0.008194708
REG00014	PGK1	GSE109604	-0.704376484	knock down	down-regulator	0.001082032
REG00014	COPS7B	GSE109604	-0.704044233	knock down	down-regulator	0.011512852
REG00014	C1orf53	GSE109604	-0.701802011	knock down	down-regulator	0.009465165
REG00014	S1PR1	GSE109604	-0.70083032	knock down	down-regulator	0.005485678
REG00014	RPL35	GSE109604	-0.700635508	knock down	down-regulator	0.005611022
REG00014	AGRN	GSE109604	-0.700474284	knock down	down-regulator	1.03E-08
REG00014	BNIP3L	GSE109604	-0.699880295	knock down	down-regulator	0.031568242
REG00014	DYNLL2	GSE109604	-0.699804551	knock down	down-regulator	0.00016042
REG00014	SLC9B2	GSE109604	-0.698429837	knock down	down-regulator	0.006433038
REG00014	PTHLH	GSE109604	-0.69779192	knock down	down-regulator	0.042968575
REG00014	CANX	GSE109604	-0.696066359	knock down	down-regulator	2.64E-05
REG00014	SIRT3	GSE109604	-0.695761861	knock down	down-regulator	0.00100155
REG00014	SLC2A10	GSE109604	-0.693776383	knock down	down-regulator	0.017901811
REG00014	PSME3	GSE109604	-0.693776383	knock down	down-regulator	0.017901811
REG00014	OPN3	GSE109604	-0.693346311	knock down	down-regulator	8.44E-05
REG00014	MAPK8IP3	GSE109604	-0.692452885	knock down	down-regulator	0.00193681
REG00014	COL3A1	GSE109604	-0.69173015	knock down	down-regulator	0.003860828
REG00014	SGPL1	GSE109604	-0.690632611	knock down	down-regulator	0.000468434
REG00014	CCDC85C	GSE109604	-0.689358989	knock down	down-regulator	0.036524142
REG00014	MAP6D1	GSE109604	-0.685874164	knock down	down-regulator	8.52E-09
REG00014	CNOT9	GSE109604	-0.6857632	knock down	down-regulator	0.029027722
REG00014	FAM131C	GSE109604	-0.685399248	knock down	down-regulator	0.025183503
REG00014	KLHL26	GSE109604	-0.683862154	knock down	down-regulator	4.42E-17
REG00014	SPECC1	GSE109604	-0.682946779	knock down	down-regulator	0.011348169
REG00014	KRT15	GSE109604	-0.682746522	knock down	down-regulator	2.18E-15
REG00014	DLEC1	GSE109604	-0.681145805	knock down	down-regulator	0.003492244
REG00014	ADGRF3	GSE109604	-0.680762395	knock down	down-regulator	4.11E-05
REG00014	PTGFRN	GSE109604	-0.680512487	knock down	down-regulator	0.011990929
REG00014	HERPUD1	GSE109604	-0.678506557	knock down	down-regulator	0.004841899
REG00014	MZF1	GSE109604	-0.677676241	knock down	down-regulator	0.040626038
REG00014	EFCAB14	GSE109604	-0.677637163	knock down	down-regulator	0.000745566
REG00014	WFIKKN1	GSE109604	-0.675667364	knock down	down-regulator	0.029091974
REG00014	PSG9	GSE109604	-0.675430257	knock down	down-regulator	0.004712008
REG00014	RARS1	GSE109604	-0.674242334	knock down	down-regulator	2.28E-05
REG00014	ANKMY2	GSE109604	-0.674238343	knock down	down-regulator	0.006866986

REG00014	CKMT1A	GSE109604	-0.673168175	knock down	down-regulator	0.002134891
REG00014	JADE3	GSE109604	-0.673045648	knock down	down-regulator	0.00066474
REG00014	MBD3	GSE109604	-0.671655366	knock down	down-regulator	0.022063536
REG00014	PPP2R3B	GSE109604	-0.670773155	knock down	down-regulator	0.025292098
REG00014	PPM1B	GSE109604	-0.670186672	knock down	down-regulator	0.010147644
REG00014	E2F2	GSE109604	-0.670159103	knock down	down-regulator	0.000405599
REG00014	UBE2L6	GSE109604	-0.669614287	knock down	down-regulator	0.030572545
REG00014	RIPK1	GSE109604	-0.669233841	knock down	down-regulator	0.001325878
REG00014	SLCO4A1	GSE109604	-0.66910356	knock down	down-regulator	0.026897599
REG00014	GINS4	GSE109604	-0.668064028	knock down	down-regulator	4.73E-14
REG00014	PTGS1	GSE109604	-0.667594193	knock down	down-regulator	0.020196364
REG00014	ADAMTS15	GSE109604	-0.666942957	knock down	down-regulator	0.007082955
REG00014	DYRK1A	GSE109604	-0.663071853	knock down	down-regulator	0.012847486
REG00014	FKBP6	GSE109604	-0.661656242	knock down	down-regulator	0.000768965
REG00014	HIPK3	GSE109604	-0.661163241	knock down	down-regulator	0.002333834
REG00014	NOTCH4	GSE109604	-0.660462117	knock down	down-regulator	0.001731212
REG00014	ZNF227	GSE109604	-0.660183217	knock down	down-regulator	0.000259153
REG00014	EPN2	GSE109604	-0.660097031	knock down	down-regulator	0.033464237
REG00014	RPS6KB2	GSE109604	-0.659862114	knock down	down-regulator	1.52E-05
REG00014	CEACAM1	GSE109604	-0.659496421	knock down	down-regulator	0.007436701
REG00014	DNER	GSE109604	-0.65882886	knock down	down-regulator	0.000617751
REG00014	PRPS1	GSE109604	-0.657656646	knock down	down-regulator	0.000501381
REG00014	RACGAP1	GSE109604	-0.657134164	knock down	down-regulator	0.03136391
REG00014	CALHM5	GSE109604	-0.656407682	knock down	down-regulator	0.033589242
REG00014	SPRYD4	GSE109604	-0.653015359	knock down	down-regulator	0.008326742
REG00014	USP24	GSE109604	-0.652963201	knock down	down-regulator	0.01534306
REG00014	NFX1	GSE109604	-0.652222069	knock down	down-regulator	0.0258611
REG00014	BRME1	GSE109604	-0.651707066	knock down	down-regulator	0.000741729
REG00014	DHX32	GSE109604	-0.650816013	knock down	down-regulator	0.026246473
REG00014	OSGIN1	GSE109604	-0.649585094	knock down	down-regulator	4.16E-05
REG00014	RARS2	GSE109604	-0.649240692	knock down	down-regulator	0.000926228
REG00014	SUCLG2	GSE109604	-0.648649203	knock down	down-regulator	0.046000681
REG00014	SERPINE1	GSE109604	-0.648642854	knock down	down-regulator	0.000372608
REG00014	OGFOD2	GSE109604	-0.64832788	knock down	down-regulator	0.001918727
REG00014	EIF2S3B	GSE109604	-0.647978146	knock down	down-regulator	0.00464393
REG00014	ARL4C	GSE109604	-0.646785118	knock down	down-regulator	3.41E-09
REG00014	ZNF45	GSE109604	-0.646543529	knock down	down-regulator	0.000100503
REG00014	ST6GALNAC6	GSE109604	-0.646095474	knock down	down-regulator	0.008678332
REG00014	PDGFRA	GSE109604	-0.645367677	knock down	down-regulator	0.016012039
REG00014	FBXL5	GSE109604	-0.644732421	knock down	down-regulator	2.48E-05
REG00014	NAA25	GSE109604	-0.644019202	knock down	down-regulator	0.000150818
REG00014	BCAN	GSE109604	-0.643899567	knock down	down-regulator	0.021883421
REG00014	CGB1	GSE109604	-0.642764487	knock down	down-regulator	0.03914618
REG00014	CPM	GSE109604	-0.642478529	knock down	down-regulator	0.001619797
REG00014	PTTG1IP	GSE109604	-0.642377382	knock down	down-regulator	0.007153079
REG00014	LPCAT2	GSE109604	-0.642072676	knock down	down-regulator	0.000429829
REG00014	SPART	GSE109604	-0.641220112	knock down	down-regulator	0.026859956
REG00014	LSS	GSE109604	-0.641034334	knock down	down-regulator	1.17E-05
REG00014	SNORC	GSE109604	-0.640627444	knock down	down-regulator	0.005977351
REG00014	NDUFA1	GSE109604	-0.639755999	knock down	down-regulator	0.001488989

REG00014	KCTD4	GSE109604	-0.639293384	knock down	down-regulator	9.49E-05
REG00014	GMEB2	GSE109604	-0.638958371	knock down	down-regulator	4.30E-103
REG00014	ZDHHC1	GSE109604	-0.638289413	knock down	down-regulator	0.002369959
REG00014	PHF1	GSE109604	-0.637996916	knock down	down-regulator	0.00096147
REG00014	DHRS3	GSE109604	-0.637860874	knock down	down-regulator	0.000698616
REG00014	SLC35F2	GSE109604	-0.637643083	knock down	down-regulator	0.025936102
REG00014	CLCN6	GSE109604	-0.637619235	knock down	down-regulator	0.003808515
REG00014	KLHL22	GSE109604	-0.63755293	knock down	down-regulator	6.48E-08
REG00014	G0S2	GSE109604	-0.637302264	knock down	down-regulator	0.000286102
REG00014	KRTAP2-4	GSE109604	-0.637268947	knock down	down-regulator	7.89E-09
REG00014	SEPTIN11	GSE109604	-0.636824771	knock down	down-regulator	0.021347683
REG00014	AHCYL2	GSE109604	-0.636333176	knock down	down-regulator	0.012704711
REG00014	MC1R	GSE109604	-0.635547391	knock down	down-regulator	0.0007855
REG00014	RAP1GAP2	GSE109604	-0.635246283	knock down	down-regulator	0.015403522
REG00014	ANO6	GSE109604	-0.634932	knock down	down-regulator	0.015229983
REG00014	NDUFS2	GSE109604	-0.63358483	knock down	down-regulator	0.047720215
REG00014	LSM7	GSE109604	-0.633465811	knock down	down-regulator	2.06E-05
REG00014	LSM10	GSE109604	-0.632878051	knock down	down-regulator	6.68E-05
REG00014	LIMD2	GSE109604	-0.632601305	knock down	down-regulator	0.047792686
REG00014	SLC2A4RG	GSE109604	-0.632250237	knock down	down-regulator	7.11E-06
REG00014	NPHP3	GSE109604	-0.631412136	knock down	down-regulator	4.98E-05
REG00014	SPANXD	GSE109604	-0.63135462	knock down	down-regulator	0.012534346
REG00014	ZNF551	GSE109604	-0.631130419	knock down	down-regulator	4.79E-07
REG00014	PPP1R11	GSE109604	-0.630786374	knock down	down-regulator	0.036423587
REG00014	FAM171A1	GSE109604	-0.630385448	knock down	down-regulator	0.038426906
REG00014	ZNF574	GSE109604	-0.630020334	knock down	down-regulator	1.87E-05
REG00014	MVD	GSE109604	-0.628516191	knock down	down-regulator	0.001416224
REG00014	KPNB1	GSE109604	-0.628345968	knock down	down-regulator	0.039517534
REG00014	NUTM2A	GSE109604	-0.627905907	knock down	down-regulator	0.000990931
REG00014	CDH5	GSE109604	-0.627503555	knock down	down-regulator	0.000279801
REG00014	YWHAE	GSE109604	-0.626549035	knock down	down-regulator	0.002621097
REG00014	NECAB3	GSE109604	-0.626524611	knock down	down-regulator	0.001148718
REG00014	ZFP36	GSE109604	-0.626204636	knock down	down-regulator	0.012280546
REG00014	ITCH	GSE109604	-0.624724768	knock down	down-regulator	2.65E-05
REG00014	RPL26L1	GSE109604	-0.624541354	knock down	down-regulator	3.37E-05
REG00014	PSME1	GSE109604	-0.624259154	knock down	down-regulator	0.022246075
REG00014	RBM33	GSE109604	-0.622821437	knock down	down-regulator	1.84E-05
REG00014	ACADM	GSE109604	-0.622648928	knock down	down-regulator	0.000170513
REG00014	PKP3	GSE109604	-0.621824316	knock down	down-regulator	0.002803523
REG00014	NGLY1	GSE109604	-0.621667768	knock down	down-regulator	0.004346553
REG00014	ADGRL1	GSE109604	-0.621156718	knock down	down-regulator	0.000140231
REG00014	DIAPH3	GSE109604	-0.621143194	knock down	down-regulator	0.007560265
REG00014	CCT2	GSE109604	-0.620907404	knock down	down-regulator	0.023799876
REG00014	PUS10	GSE109604	-0.620235337	knock down	down-regulator	0.004240774
REG00014	BMP4	GSE109604	-0.620233062	knock down	down-regulator	0.007909647
REG00014	HDAC4	GSE109604	-0.619742588	knock down	down-regulator	0.034468397
REG00014	RBMX2	GSE109604	-0.619564757	knock down	down-regulator	0.001841661
REG00014	ZNF616	GSE109604	-0.617300545	knock down	down-regulator	4.92E-05
REG00014	SPEG	GSE109604	-0.617222805	knock down	down-regulator	0.004484872
REG00014	ANXA5	GSE109604	-0.616436353	knock down	down-regulator	0.000331994

REG00014	IMPA2	GSE109604	-0.615656971	knock down	down-regulator	0.001133816
REG00014	CYFIP2	GSE109604	-0.615239989	knock down	down-regulator	0.000504607
REG00014	INTS4	GSE109604	-0.615006723	knock down	down-regulator	0.000691686
REG00014	MGST1	GSE109604	-0.614291936	knock down	down-regulator	0.041727572
REG00014	PTGR1	GSE109604	-0.613723856	knock down	down-regulator	0.026105152
REG00014	RAB3B	GSE109604	-0.613382807	knock down	down-regulator	0.022108755
REG00014	NGFR	GSE109604	-0.61305988	knock down	down-regulator	0.000583501
REG00014	ARL6IP5	GSE109604	-0.612419714	knock down	down-regulator	0.005868425
REG00014	FOXD4L3	GSE109604	-0.612286939	knock down	down-regulator	0.000284738
REG00014	PTK6	GSE109604	-0.611734937	knock down	down-regulator	0.005281996
REG00014	IL17D	GSE109604	-0.611100184	knock down	down-regulator	8.26E-05
REG00014	POLE3	GSE109604	-0.61015439	knock down	down-regulator	0.004359755
REG00014	ABCB7	GSE109604	-0.610021487	knock down	down-regulator	2.60E-12
REG00014	PTPRZ1	GSE109604	-0.609723278	knock down	down-regulator	0.011523828
REG00014	LRATD1	GSE109604	-0.608016633	knock down	down-regulator	0.016164903
REG00014	IFIT2	GSE109604	-0.607958766	knock down	down-regulator	0.000255485
REG00014	NUP98	GSE109604	-0.607760094	knock down	down-regulator	3.37E-08
REG00014	RAB11FIP1	GSE109604	-0.607271942	knock down	down-regulator	0.019408743
REG00014	WRAP73	GSE109604	-0.60649142	knock down	down-regulator	0.004584264
REG00014	SAMD4A	GSE109604	-0.605883318	knock down	down-regulator	0.004097076
REG00014	PLAT	GSE109604	-0.605421804	knock down	down-regulator	0.017607073
REG00014	PSMB10	GSE109604	-0.603931279	knock down	down-regulator	0.010085568
REG00014	RAVER1	GSE109604	-0.603314382	knock down	down-regulator	0.012282476
REG00014	PDZD11	GSE109604	-0.603314381	knock down	down-regulator	0.012282476
REG00014	HPDL	GSE109604	-0.603282904	knock down	down-regulator	0.005342158
REG00014	PTPRN2	GSE109604	-0.603114395	knock down	down-regulator	0.00755474
REG00014	PPP6C	GSE109604	-0.60230736	knock down	down-regulator	0.030132193
REG00014	SPTB	GSE109604	-0.601542158	knock down	down-regulator	0.00606544
REG00014	FKBP1A	GSE109604	-0.601540273	knock down	down-regulator	0.000755133
REG00014	KIT	GSE109604	-0.601306399	knock down	down-regulator	0.000951688
REG00014	PSMB2	GSE109604	-0.601189884	knock down	down-regulator	0.047448776
REG00014	PCDH7	GSE109604	-0.600970885	knock down	down-regulator	0.028462584
REG00014	OSBP2	GSE109604	-0.600364708	knock down	down-regulator	0.029616911
REG00014	GLIS2	GSE109604	-0.599354473	knock down	down-regulator	0.002539272
REG00014	CARMIL1	GSE109604	-0.599102433	knock down	down-regulator	0.000526328
REG00014	ZNF692	GSE109604	-0.598257805	knock down	down-regulator	0.044866194
REG00014	DGKZ	GSE109604	-0.597485704	knock down	down-regulator	0.035361304
REG00014	AK5	GSE109604	-0.597407263	knock down	down-regulator	0.001813011
REG00014	GPM6A	GSE109604	-0.596366531	knock down	down-regulator	0.001162792
REG00014	COL18A1	GSE109604	-0.59506405	knock down	down-regulator	0.012117297
REG00014	FANCI	GSE109604	-0.594627234	knock down	down-regulator	0.024323113
REG00014	M6PR	GSE109604	-0.594163381	knock down	down-regulator	0.000739671
REG00014	ADNP2	GSE109604	-0.594089451	knock down	down-regulator	5.05E-09
REG00014	MDH2	GSE109604	-0.593122388	knock down	down-regulator	0.00119753
REG00014	IFTAP	GSE109604	-0.592274022	knock down	down-regulator	3.30E-05
REG00014	NR3C2	GSE109604	-0.592258672	knock down	down-regulator	0.002419816
REG00014	HARS1	GSE109604	-0.592034948	knock down	down-regulator	0.016011796
REG00014	MICB	GSE109604	-0.591880245	knock down	down-regulator	0.044043022
REG00014	BRSK1	GSE109604	-0.591255898	knock down	down-regulator	0.013400796
REG00014	DYNC1I2	GSE109604	-0.59120048	knock down	down-regulator	0.001240604

REG00014	SLC6A6	GSE109604	-0.591182147	knock down	down-regulator	0.000416046
REG00014	ERV3-1	GSE109604	-0.590828694	knock down	down-regulator	0.003039056
REG00014	WDR90	GSE109604	-0.590303032	knock down	down-regulator	0.00026392
REG00014	SSBP4	GSE109604	-0.589762391	knock down	down-regulator	0.000817948
REG00014	CACNB1	GSE109604	-0.589502342	knock down	down-regulator	0.005798232
REG00014	HEXD	GSE109604	-0.588367211	knock down	down-regulator	0.033634923
REG00014	GEM	GSE109604	-0.587828763	knock down	down-regulator	1.61E-11
REG00014	PITPNM2	GSE109604	-0.58684398	knock down	down-regulator	2.00E-06
REG00014	MPP1	GSE109604	-0.586443493	knock down	down-regulator	0.000439876
REG00014	PTPRK	GSE109604	-0.585993008	knock down	down-regulator	0.01213018
REG00014	MTPAP	GSE109604	-0.585707707	knock down	down-regulator	0.00271089
REG00014	RECQL4	GSE109604	-0.585231714	knock down	down-regulator	0.000789576
REG00014	SIRT7	GSE109604	-0.585031467	knock down	down-regulator	0.001018224
REG00014	NDUFS1	GSE109604	0.58527256	knock down	up-regulate	0.000132675
REG00014	PIP5K1A	GSE109604	0.586394324	knock down	up-regulate	0.000230677
REG00014	BANP	GSE109604	0.586570211	knock down	up-regulate	3.70E-06
REG00014	GTF2A2	GSE109604	0.586644762	knock down	up-regulate	1.09E-06
REG00014	TAF1C	GSE109604	0.588366534	knock down	up-regulate	0.02204421
REG00014	SGSM2	GSE109604	0.588566753	knock down	up-regulate	0.023750171
REG00014	DISP2	GSE109604	0.588612449	knock down	up-regulate	0.015955095
REG00014	REEP2	GSE109604	0.589330223	knock down	up-regulate	0.000601299
REG00014	CHERP	GSE109604	0.589565281	knock down	up-regulate	0.020763749
REG00014	NME2	GSE109604	0.591823102	knock down	up-regulate	0.009463537
REG00014	DHX57	GSE109604	0.592998098	knock down	up-regulate	0.002167528
REG00014	RCN3	GSE109604	0.59347513	knock down	up-regulate	0.002785827
REG00014	H2BC4	GSE109604	0.593509347	knock down	up-regulate	1.91E-29
REG00014	DTX3	GSE109604	0.593532647	knock down	up-regulate	0.02625019
REG00014	EMILIN3	GSE109604	0.595830306	knock down	up-regulate	0.027494433
REG00014	POLR3C	GSE109604	0.596674768	knock down	up-regulate	0.008606702
REG00014	AP3B1	GSE109604	0.59712698	knock down	up-regulate	0.001493161
REG00014	NVL	GSE109604	0.597563458	knock down	up-regulate	0.000203246
REG00014	TBCCD1	GSE109604	0.597824949	knock down	up-regulate	0.017132644
REG00014	FLRT1	GSE109604	0.598972705	knock down	up-regulate	0.009504501
REG00014	ENTPD7	GSE109604	0.600412682	knock down	up-regulate	0.000835695
REG00014	SQOR	GSE109604	0.600682345	knock down	up-regulate	0.040647253
REG00014	COLEC11	GSE109604	0.601510736	knock down	up-regulate	0.002543456
REG00014	AMDHD1	GSE109604	0.602855502	knock down	up-regulate	0.00029031
REG00014	CASKIN1	GSE109604	0.60294784	knock down	up-regulate	0.019654941
REG00014	H2AC18	GSE109604	0.603576738	knock down	up-regulate	2.96E-32
REG00014	SARDH	GSE109604	0.603598976	knock down	up-regulate	0.016902354
REG00014	AK6	GSE109604	0.603957298	knock down	up-regulate	0.001905842
REG00014	ERVMER34-1	GSE109604	0.605279731	knock down	up-regulate	0.001224442
REG00014	CD93	GSE109604	0.605437639	knock down	up-regulate	0.039164898
REG00014	CST3	GSE109604	0.607062061	knock down	up-regulate	0.003354813
REG00014	ZNF460	GSE109604	0.60779652	knock down	up-regulate	0.004435405
REG00014	C17orf107	GSE109604	0.608524094	knock down	up-regulate	0.019735008
REG00014	GYPC	GSE109604	0.609389275	knock down	up-regulate	0.02428843
REG00014	KRTAP4-9	GSE109604	0.609454512	knock down	up-regulate	9.91E-13
REG00014	GNB3	GSE109604	0.610672361	knock down	up-regulate	7.29E-06
REG00014	IGFBP7	GSE109604	0.611831426	knock down	up-regulate	0.000145755

REG00014	ACTR8	GSE109604	0.613300034	knock down	up-regulate	0.03260372
REG00014	NDC1	GSE109604	0.613469001	knock down	up-regulate	0.000197854
REG00014	NANS	GSE109604	0.613543015	knock down	up-regulate	0.011099533
REG00014	IFI6	GSE109604	0.613547461	knock down	up-regulate	9.66E-05
REG00014	L3MBTL3	GSE109604	0.613556499	knock down	up-regulate	0.000868639
REG00014	DLX1	GSE109604	0.613637696	knock down	up-regulate	0.010566484
REG00014	COL1A1	GSE109604	0.61418428	knock down	up-regulate	0.017376088
REG00014	THBS1	GSE109604	0.614315423	knock down	up-regulate	0.046147065
REG00014	WDR3	GSE109604	0.615418341	knock down	up-regulate	0.00019217
REG00014	DNAJB14	GSE109604	0.617422558	knock down	up-regulate	0.003480235
REG00014	DOCK2	GSE109604	0.618155936	knock down	up-regulate	0.034950152
REG00014	HSPA2	GSE109604	0.618995386	knock down	up-regulate	0.005850212
REG00014	NEK8	GSE109604	0.619337355	knock down	up-regulate	0.00469574
REG00014	TAF6L	GSE109604	0.619569474	knock down	up-regulate	0.043443572
REG00014	GPNMB	GSE109604	0.620301396	knock down	up-regulate	0.001952394
REG00014	E2F4	GSE109604	0.621248208	knock down	up-regulate	0.04139221
REG00014	OAS1	GSE109604	0.622576076	knock down	up-regulate	0.000622045
REG00014	CTU2	GSE109604	0.623590881	knock down	up-regulate	0.000701383
REG00014	DIPK1A	GSE109604	0.623696103	knock down	up-regulate	0.029329741
REG00014	CCNDBP1	GSE109604	0.623819047	knock down	up-regulate	0.034243362
REG00014	GRAMD1A	GSE109604	0.625087562	knock down	up-regulate	0.002499821
REG00014	NGDN	GSE109604	0.626719402	knock down	up-regulate	0.005011404
REG00014	MAPK12	GSE109604	0.626774011	knock down	up-regulate	0.013121232
REG00014	RP2	GSE109604	0.628597546	knock down	up-regulate	0.013395746
REG00014	LRP5	GSE109604	0.628718353	knock down	up-regulate	0.00424393
REG00014	IL13RA1	GSE109604	0.629272487	knock down	up-regulate	0.000205757
REG00014	CPS1	GSE109604	0.629828378	knock down	up-regulate	0.016221799
REG00014	SEC23A	GSE109604	0.629884435	knock down	up-regulate	0.005496575
REG00014	CIB1	GSE109604	0.630349876	knock down	up-regulate	6.92E-06
REG00014	TAF9B	GSE109604	0.631441562	knock down	up-regulate	0.046175994
REG00014	FOXD4L1	GSE109604	0.631705384	knock down	up-regulate	6.70E-05
REG00014	SH3GL1	GSE109604	0.632401296	knock down	up-regulate	0.010579041
REG00014	HPS1	GSE109604	0.632821009	knock down	up-regulate	0.047900523
REG00014	DDO	GSE109604	0.632990236	knock down	up-regulate	0.011885168
REG00014	NTHL1	GSE109604	0.633102543	knock down	up-regulate	3.09E-05
REG00014	ATF1	GSE109604	0.633356207	knock down	up-regulate	0.009743267
REG00014	STAC	GSE109604	0.634124862	knock down	up-regulate	0.036094592
REG00014	ARHGEF25	GSE109604	0.634241315	knock down	up-regulate	0.014183849
REG00014	SLC9A3R1	GSE109604	0.634483719	knock down	up-regulate	0.004899275
REG00014	ANKRD33B	GSE109604	0.634622398	knock down	up-regulate	1.47E-05
REG00014	ANXA1	GSE109604	0.635009774	knock down	up-regulate	0.032703555
REG00014	NMT2	GSE109604	0.635426978	knock down	up-regulate	0.036477912
REG00014	LIFR	GSE109604	0.636061982	knock down	up-regulate	0.025905613
REG00014	MB21D2	GSE109604	0.637176618	knock down	up-regulate	0.001137637
REG00014	DPYSL3	GSE109604	0.638587261	knock down	up-regulate	0.002497753
REG00014	ARMCX2	GSE109604	0.639281246	knock down	up-regulate	0.004768021
REG00014	SMAD7	GSE109604	0.640992356	knock down	up-regulate	0.033430825
REG00014	GLA	GSE109604	0.64135671	knock down	up-regulate	0.000235824
REG00014	DNAJC4	GSE109604	0.642162141	knock down	up-regulate	0.022385727
REG00014	ATF2	GSE109604	0.643526415	knock down	up-regulate	0.013447358

REG00014	SFI1	GSE109604	0.644508866	knock down	up-regulate	0.000294598
REG00014	KRBA1	GSE109604	0.64503478	knock down	up-regulate	0.000527875
REG00014	KDM4B	GSE109604	0.645216979	knock down	up-regulate	0.00092087
REG00014	KYAT1	GSE109604	0.647067661	knock down	up-regulate	2.37E-16
REG00014	CPT1A	GSE109604	0.648102699	knock down	up-regulate	0.001008856
REG00014	AZIN2	GSE109604	0.648740729	knock down	up-regulate	0.043052462
REG00014	FRS3	GSE109604	0.649227631	knock down	up-regulate	0.002907905
REG00014	NPHP4	GSE109604	0.649590035	knock down	up-regulate	0.002713198
REG00014	YJEFN3	GSE109604	0.649591347	knock down	up-regulate	1.06E-05
REG00014	STAT2	GSE109604	0.650800024	knock down	up-regulate	0.000676786
REG00014	TSPAN15	GSE109604	0.650977678	knock down	up-regulate	0.013014505
REG00014	NFXL1	GSE109604	0.651643901	knock down	up-regulate	5.40E-07
REG00014	PRDM15	GSE109604	0.6522	knock down	up-regulate	0.03916
REG00014	OPTN	GSE109604	0.65291771	knock down	up-regulate	0.012356355
REG00014	ADGRB2	GSE109604	0.653018019	knock down	up-regulate	1.47E-05
REG00014	MEX3D	GSE109604	0.653143285	knock down	up-regulate	0.018543461
REG00014	SH2D5	GSE109604	0.654002798	knock down	up-regulate	0.00297586
REG00014	ZFP91	GSE109604	0.654076297	knock down	up-regulate	1.14E-05
REG00014	NDUFAF1	GSE109604	0.654120391	knock down	up-regulate	0.000798457
REG00014	CDK20	GSE109604	0.65516992	knock down	up-regulate	0.022755706
REG00014	UNC13D	GSE109604	0.655175247	knock down	up-regulate	0.017391088
REG00014	GLUD1	GSE109604	0.656712638	knock down	up-regulate	0.006021706
REG00014	NAA16	GSE109604	0.658421681	knock down	up-regulate	0.002713003
REG00014	OGDH	GSE109604	0.660619763	knock down	up-regulate	0.042116168
REG00014	PLPP5	GSE109604	0.661776486	knock down	up-regulate	0.003484343
REG00014	RAVER2	GSE109604	0.662113665	knock down	up-regulate	0.02385327
REG00014	CCNL2	GSE109604	0.662969873	knock down	up-regulate	0.001528486
REG00014	C3AR1	GSE109604	0.667405387	knock down	up-regulate	0.029034522
REG00014	CNIH3	GSE109604	0.668545389	knock down	up-regulate	0.001318821
REG00014	GAB1	GSE109604	0.671667849	knock down	up-regulate	6.60E-65
REG00014	LY96	GSE109604	0.671680985	knock down	up-regulate	0.003887293
REG00014	IP6K2	GSE109604	0.672052512	knock down	up-regulate	0.000119656
REG00014	SKIC3	GSE109604	0.673812685	knock down	up-regulate	0.002479639
REG00014	CAPRIN1	GSE109604	0.674051182	knock down	up-regulate	0.049677989
REG00014	RNASEH1	GSE109604	0.674504954	knock down	up-regulate	0.004009156
REG00014	DERL2	GSE109604	0.674585052	knock down	up-regulate	0.000944743
REG00014	MFAP1	GSE109604	0.675262063	knock down	up-regulate	0.009809071
REG00014	DHRS12	GSE109604	0.675846708	knock down	up-regulate	0.013855384
REG00014	ZC3H10	GSE109604	0.675892041	knock down	up-regulate	0.001191631
REG00014	CCDC57	GSE109604	0.676432801	knock down	up-regulate	0.036381604
REG00014	SFXN2	GSE109604	0.676583882	knock down	up-regulate	0.007931348
REG00014	ABHD16A	GSE109604	0.677526972	knock down	up-regulate	0.048317852
REG00014	DUT	GSE109604	0.679061939	knock down	up-regulate	0.00044568
REG00014	FBXW9	GSE109604	0.679507411	knock down	up-regulate	0.009538763
REG00014	PEX2	GSE109604	0.67978541	knock down	up-regulate	0.022573811
REG00014	ATP2C1	GSE109604	0.681823584	knock down	up-regulate	2.84E-07
REG00014	MTRF1L	GSE109604	0.68223974	knock down	up-regulate	0.007149317
REG00014	PPFIBP1	GSE109604	0.6826	knock down	up-regulate	0.001285
REG00014	CARMIL2	GSE109604	0.683765781	knock down	up-regulate	0.029063683
REG00014	GRTP1	GSE109604	0.684686293	knock down	up-regulate	3.70E-07

REG00014	KIAA0586	GSE109604	0.686297039	knock down	up-regulate	0.010687988
REG00014	FDXACB1	GSE109604	0.687484913	knock down	up-regulate	0.023984794
REG00014	KLF16	GSE109604	0.687958968	knock down	up-regulate	3.55E-29
REG00014	DBNDD2	GSE109604	0.687971828	knock down	up-regulate	0.003655296
REG00014	CELSR3	GSE109604	0.688872133	knock down	up-regulate	0.007536888
REG00014	FBXW4	GSE109604	0.68958968	knock down	up-regulate	0.021409724
REG00014	CLMP	GSE109604	0.690109472	knock down	up-regulate	0.002386957
REG00014	LEPROT	GSE109604	0.690658216	knock down	up-regulate	0.048936209
REG00014	MROH6	GSE109604	0.692099612	knock down	up-regulate	7.23E-06
REG00014	COL17A1	GSE109604	0.69258595	knock down	up-regulate	0.003681437
REG00014	MILR1	GSE109604	0.692735587	knock down	up-regulate	1.96E-05
REG00014	TAF4	GSE109604	0.692753071	knock down	up-regulate	0.006766804
REG00014	PLAGL1	GSE109604	0.694055405	knock down	up-regulate	0.003467828
REG00014	VDAC3	GSE109604	0.694502222	knock down	up-regulate	6.43E-07
REG00014	ID3	GSE109604	0.694953414	knock down	up-regulate	0.000238378
REG00014	MYO9B	GSE109604	0.698474535	knock down	up-regulate	0.035253427
REG00014	S100A10	GSE109604	0.700252165	knock down	up-regulate	0.000545992
REG00014	PARP3	GSE109604	0.700252165	knock down	up-regulate	0.000545992
REG00014	SERPINE2	GSE109604	0.701336411	knock down	up-regulate	9.96E-05
REG00014	SEMA6C	GSE109604	0.701393761	knock down	up-regulate	0.029368862
REG00014	DNAH17	GSE109604	0.702412457	knock down	up-regulate	0.011056434
REG00014	PHF10	GSE109604	0.704462628	knock down	up-regulate	0.000194576
REG00014	SMIM30	GSE109604	0.704675558	knock down	up-regulate	0.000872427
REG00014	CELSR2	GSE109604	0.70476413	knock down	up-regulate	0.001702188
REG00014	GSTT2B	GSE109604	0.706189093	knock down	up-regulate	0.000337946
REG00014	TARBP1	GSE109604	0.707094747	knock down	up-regulate	0.008328369
REG00014	DLL3	GSE109604	0.707271815	knock down	up-regulate	0.000613545
REG00014	SDK1	GSE109604	0.708234985	knock down	up-regulate	0.023219889
REG00014	GLIPR1	GSE109604	0.709074115	knock down	up-regulate	0.003975024
REG00014	IARS1	GSE109604	0.710360678	knock down	up-regulate	0.000431737
REG00014	EBPL	GSE109604	0.71053312	knock down	up-regulate	0.02494292
REG00014	LYRM1	GSE109604	0.711589784	knock down	up-regulate	2.10E-05
REG00014	RPRD1A	GSE109604	0.711774847	knock down	up-regulate	0.000144611
REG00014	TES	GSE109604	0.712997094	knock down	up-regulate	0.008463117
REG00014	FADS2	GSE109604	0.713077143	knock down	up-regulate	0.01914578
REG00014	CHP1	GSE109604	0.713101217	knock down	up-regulate	0.004380225
REG00014	EXOSC5	GSE109604	0.715791571	knock down	up-regulate	0.004512599
REG00014	REXO2	GSE109604	0.715896449	knock down	up-regulate	0.00303629
REG00014	NDUFAF4	GSE109604	0.716134375	knock down	up-regulate	0.011944128
REG00014	EML3	GSE109604	0.716745378	knock down	up-regulate	0.019084415
REG00014	SYP	GSE109604	0.717261959	knock down	up-regulate	0.003696034
REG00014	INSIG1	GSE109604	0.718219934	knock down	up-regulate	0.001954017
REG00014	IL11	GSE109604	0.718917267	knock down	up-regulate	5.08E-05
REG00014	SASH1	GSE109604	0.719782401	knock down	up-regulate	0.012296034
REG00014	FTSJ1	GSE109604	0.719961315	knock down	up-regulate	0.034074031
REG00014	C1orf159	GSE109604	0.720087218	knock down	up-regulate	8.06E-07
REG00014	MMP2	GSE109604	0.720160166	knock down	up-regulate	6.55E-05
REG00014	RPS24	GSE109604	0.720473363	knock down	up-regulate	0.000326089
REG00014	TXN2	GSE109604	0.72107191	knock down	up-regulate	0.005381619
REG00014	IQCG	GSE109604	0.721338472	knock down	up-regulate	0.000305565

REG00014	RHBDD2	GSE109604	0.722229919	knock down	up-regulate	0.000709947
REG00014	LAMA5	GSE109604	0.722823559	knock down	up-regulate	6.30E-07
REG00014	NTN4	GSE109604	0.722844325	knock down	up-regulate	0.012788122
REG00014	UBE2C	GSE109604	0.723480072	knock down	up-regulate	0.007893079
REG00014	HOXA11	GSE109604	0.72516729	knock down	up-regulate	0.000112422
REG00014	ATP6V1A	GSE109604	0.725274244	knock down	up-regulate	0.002578132
REG00014	MAPK7	GSE109604	0.725588529	knock down	up-regulate	0.00460045
REG00014	GMFG	GSE109604	0.728359208	knock down	up-regulate	0.005761688
REG00014	LOXL2	GSE109604	0.728741161	knock down	up-regulate	0.000205694
REG00014	TMEM171	GSE109604	0.729313814	knock down	up-regulate	0.016126346
REG00014	CKAP4	GSE109604	0.729994506	knock down	up-regulate	0.013060929
REG00014	VDAC2	GSE109604	0.73022657	knock down	up-regulate	0.004822332
REG00014	DZANK1	GSE109604	0.730757187	knock down	up-regulate	0.005010825
REG00014	RFX1	GSE109604	0.731795441	knock down	up-regulate	0.007790591
REG00014	RNF126	GSE109604	0.732455713	knock down	up-regulate	3.01E-05
REG00014	GFPT2	GSE109604	0.732473186	knock down	up-regulate	6.91E-05
REG00014	CCDC86	GSE109604	0.734336518	knock down	up-regulate	0.003347496
REG00014	FANCD2	GSE109604	0.73470459	knock down	up-regulate	0.019823168
REG00014	ATF6	GSE109604	0.73500782	knock down	up-regulate	0.009542749
REG00014	FGF5	GSE109604	0.736097538	knock down	up-regulate	0.015936958
REG00014	NAXD	GSE109604	0.736487184	knock down	up-regulate	0.042352666
REG00014	GCNT1	GSE109604	0.736646146	knock down	up-regulate	2.72E-10
REG00014	NR2F1	GSE109604	0.737619585	knock down	up-regulate	0.000396726
REG00014	FBXO32	GSE109604	0.737724721	knock down	up-regulate	0.008985851
REG00014	IFI35	GSE109604	0.740349149	knock down	up-regulate	0.001083003
REG00014	PRDM1	GSE109604	0.7409	knock down	up-regulate	1.11E-05
REG00014	KCNV2	GSE109604	0.7435	knock down	up-regulate	0.0102
REG00014	CDH20	GSE109604	0.744007628	knock down	up-regulate	0.008134222
REG00014	SMURF2	GSE109604	0.744574696	knock down	up-regulate	0.011207065
REG00014	LLGL2	GSE109604	0.745304819	knock down	up-regulate	0.044144481
REG00014	TBX6	GSE109604	0.745538953	knock down	up-regulate	0.012146886
REG00014	NSMAF	GSE109604	0.746527763	knock down	up-regulate	0.014810876
REG00014	SHC4	GSE109604	0.747786902	knock down	up-regulate	0.018104368
REG00014	CCDC32	GSE109604	0.748932175	knock down	up-regulate	0.042043845
REG00014	VWA7	GSE109604	0.749287496	knock down	up-regulate	1.39E-06
REG00014	CDHR3	GSE109604	0.74938806	knock down	up-regulate	3.76E-05
REG00014	PCIF1	GSE109604	0.753730525	knock down	up-regulate	0.000826437
REG00014	POLR2G	GSE109604	0.753730525	knock down	up-regulate	0.000826437
REG00014	STKLD1	GSE109604	0.755541934	knock down	up-regulate	0.001896846
REG00014	MGAT5B	GSE109604	0.75636279	knock down	up-regulate	7.71E-09
REG00014	INVS	GSE109604	0.756578	knock down	up-regulate	0.001833467
REG00014	ELFN2	GSE109604	0.757191168	knock down	up-regulate	0.008491322
REG00014	LENG8	GSE109604	0.757757824	knock down	up-regulate	0.002478147
REG00014	BMPER	GSE109604	0.75797571	knock down	up-regulate	0.017326264
REG00014	APC2	GSE109604	0.759016666	knock down	up-regulate	0.029401829
REG00014	DONSON	GSE109604	0.763949139	knock down	up-regulate	6.11E-05
REG00014	RSKR	GSE109604	0.763975725	knock down	up-regulate	0.010578863
REG00014	FKBP11	GSE109604	0.764110229	knock down	up-regulate	1.06E-05
REG00014	DOCK9	GSE109604	0.764305782	knock down	up-regulate	0.001737591
REG00014	SIX5	GSE109604	0.764595816	knock down	up-regulate	0.000134121

REG00014	GSTP1	GSE109604	0.766791253	knock down	up-regulate	1.64E-05
REG00014	KCNQ5	GSE109604	0.7668	knock down	up-regulate	0.01479
REG00014	ARHGAP33	GSE109604	0.76765083	knock down	up-regulate	0.029405361
REG00014	CALCOCO2	GSE109604	0.768023443	knock down	up-regulate	0.018285254
REG00014	CLIC2	GSE109604	0.769718668	knock down	up-regulate	0.01371182
REG00014	CDC42EP3	GSE109604	0.769942502	knock down	up-regulate	0.001699536
REG00014	RPL37A	GSE109604	0.770315425	knock down	up-regulate	0.005161356
REG00014	RPS27A	GSE109604	0.770717811	knock down	up-regulate	3.34E-06
REG00014	HMGB3	GSE109604	0.771915771	knock down	up-regulate	0.037633353
REG00014	SRSF2	GSE109604	0.772119028	knock down	up-regulate	0.037272751
REG00014	PARP14	GSE109604	0.772786385	knock down	up-regulate	0.020213447
REG00014	PGAP1	GSE109604	0.773476559	knock down	up-regulate	0.000111286
REG00014	FASTKD5	GSE109604	0.773613522	knock down	up-regulate	0.014760023
REG00014	FBXL15	GSE109604	0.77423787	knock down	up-regulate	1.37E-87
REG00014	CCNJ	GSE109604	0.775920632	knock down	up-regulate	0.011367683
REG00014	ITGB5	GSE109604	0.777386287	knock down	up-regulate	0.006451563
REG00014	IFT46	GSE109604	0.778021603	knock down	up-regulate	4.80E-05
REG00014	AK1	GSE109604	0.778398013	knock down	up-regulate	3.17E-05
REG00014	SCRIB	GSE109604	0.779223761	knock down	up-regulate	0.000293618
REG00014	ZNF417	GSE109604	0.779527293	knock down	up-regulate	0.000368325
REG00014	NINL	GSE109604	0.780107362	knock down	up-regulate	0.000239812
REG00014	KDELR2	GSE109604	0.780577678	knock down	up-regulate	2.65E-21
REG00014	SHISA3	GSE109604	0.783408245	knock down	up-regulate	0.014079085
REG00014	GLB1L	GSE109604	0.783998969	knock down	up-regulate	0.00168444
REG00014	TAGLN3	GSE109604	0.784481578	knock down	up-regulate	0.004444269
REG00014	GM2A	GSE109604	0.786158062	knock down	up-regulate	2.01E-07
REG00014	FHIP2B	GSE109604	0.786259646	knock down	up-regulate	0.02521682
REG00014	TMEM167B	GSE109604	0.786476565	knock down	up-regulate	0.004445331
REG00014	FOXO3B	GSE109604	0.786702892	knock down	up-regulate	5.47E-05
REG00014	PER1	GSE109604	0.788475893	knock down	up-regulate	0.000496173
REG00014	STAG3	GSE109604	0.791549536	knock down	up-regulate	0.000581614
REG00014	CASD1	GSE109604	0.791628795	knock down	up-regulate	0.000295708
REG00014	ZDHHC18	GSE109604	0.792886877	knock down	up-regulate	0.045073916
REG00014	MRPL4	GSE109604	0.792955457	knock down	up-regulate	0.000629041
REG00014	GOLGA5	GSE109604	0.793252934	knock down	up-regulate	3.06E-05
REG00014	DCXR	GSE109604	0.796962985	knock down	up-regulate	0.000689665
REG00014	C16orf46	GSE109604	0.79735636	knock down	up-regulate	0.046956015
REG00014	AEBP1	GSE109604	0.797855406	knock down	up-regulate	1.11E-07
REG00014	TTC39C	GSE109604	0.798467571	knock down	up-regulate	0.013698888
REG00014	GOLGA8A	GSE109604	0.799128474	knock down	up-regulate	0.045048318
REG00014	TIMP3	GSE109604	0.799728674	knock down	up-regulate	0.03874894
REG00014	UBL4A	GSE109604	0.800375221	knock down	up-regulate	0.001911092
REG00014	ZBTB1	GSE109604	0.801281146	knock down	up-regulate	1.40E-05
REG00014	GSN	GSE109604	0.801373198	knock down	up-regulate	9.84E-10
REG00014	SUSD2	GSE109604	0.803145165	knock down	up-regulate	0.00440375
REG00014	BTRC	GSE109604	0.80338488	knock down	up-regulate	3.47E-06
REG00014	ACADS	GSE109604	0.807720549	knock down	up-regulate	0.001286275
REG00014	EXOSC9	GSE109604	0.809277723	knock down	up-regulate	0.008908954
REG00014	CA11	GSE109604	0.810723153	knock down	up-regulate	0.014815704
REG00014	ACSL5	GSE109604	0.811590911	knock down	up-regulate	0.003733107

REG00014	CSF3	GSE109604	0.81252994	knock down	up-regulate	0.0007453
REG00014	MAT2B	GSE109604	0.813652275	knock down	up-regulate	0.047675797
REG00014	ASMTL	GSE109604	0.816818174	knock down	up-regulate	0.018773519
REG00014	TINAGL1	GSE109604	0.817491085	knock down	up-regulate	0.00143739
REG00014	CAMKK1	GSE109604	0.820635389	knock down	up-regulate	0.036160708
REG00014	DLX2	GSE109604	0.820678118	knock down	up-regulate	0.007183215
REG00014	HDGF	GSE109604	0.822213125	knock down	up-regulate	0.024239285
REG00014	STK25	GSE109604	0.825161228	knock down	up-regulate	0.015592131
REG00014	VPS9D1	GSE109604	0.825211601	knock down	up-regulate	1.28E-06
REG00014	PIF1	GSE109604	0.825495018	knock down	up-regulate	0.02992935
REG00014	CALM2	GSE109604	0.825633571	knock down	up-regulate	0.012516149
REG00014	MAPKBP1	GSE109604	0.827828187	knock down	up-regulate	0.012912434
REG00014	ACTL8	GSE109604	0.828173567	knock down	up-regulate	0.001792938
REG00014	MED7	GSE109604	0.831288054	knock down	up-regulate	9.92E-11
REG00014	ECPAS	GSE109604	0.832111752	knock down	up-regulate	0.04541185
REG00014	ANKRD44	GSE109604	0.833125075	knock down	up-regulate	8.53E-05
REG00014	GDPD5	GSE109604	0.835529847	knock down	up-regulate	6.54E-05
REG00014	CRHR2	GSE109604	0.836779604	knock down	up-regulate	0.013765495
REG00014	NIF3L1	GSE109604	0.837905058	knock down	up-regulate	0.000683779
REG00014	STK16	GSE109604	0.838402086	knock down	up-regulate	0.001478938
REG00014	HIBADH	GSE109604	0.838621179	knock down	up-regulate	0.031488331
REG00014	RC3H2	GSE109604	0.839882683	knock down	up-regulate	0.000125441
REG00014	NPRL3	GSE109604	0.840718999	knock down	up-regulate	0.025915652
REG00014	CAMK1G	GSE109604	0.841874415	knock down	up-regulate	0.000651401
REG00014	MFSD14B	GSE109604	0.841964887	knock down	up-regulate	4.10E-05
REG00014	NPC1L1	GSE109604	0.842057412	knock down	up-regulate	0.036525402
REG00014	FOS	GSE109604	0.842327697	knock down	up-regulate	0.000577871
REG00014	ORMDL2	GSE109604	0.842484772	knock down	up-regulate	0.017504796
REG00014	ZFYVE27	GSE109604	0.84390631	knock down	up-regulate	0.020642563
REG00014	FERMT3	GSE109604	0.84544925	knock down	up-regulate	0.045376154
REG00014	PDE5A	GSE109604	0.848696319	knock down	up-regulate	0.004825085
REG00014	TNC	GSE109604	0.850428963	knock down	up-regulate	0.032218973
REG00014	RCOR1	GSE109604	0.850564852	knock down	up-regulate	0.00176265
REG00014	AP1G2	GSE109604	0.850930897	knock down	up-regulate	0.025234697
REG00014	MXI1	GSE109604	0.851203829	knock down	up-regulate	6.46E-06
REG00014	CCDC77	GSE109604	0.854261941	knock down	up-regulate	0.04288871
REG00014	LDAH	GSE109604	0.854983724	knock down	up-regulate	0.026501017
REG00014	C1GALT1	GSE109604	0.855235492	knock down	up-regulate	0.002184963
REG00014	MSL1	GSE109604	0.857771125	knock down	up-regulate	0.007750136
REG00014	TAPT1	GSE109604	0.85848722	knock down	up-regulate	0.008360606
REG00014	FN3KRP	GSE109604	0.858951204	knock down	up-regulate	0.00968288
REG00014	HLA-B	GSE109604	0.859611241	knock down	up-regulate	0.000657139
REG00014	NLGN2	GSE109604	0.860112521	knock down	up-regulate	0.020902221
REG00014	SVIL	GSE109604	0.861328356	knock down	up-regulate	0.040089024
REG00014	CENPN	GSE109604	0.863817149	knock down	up-regulate	0.048244914
REG00014	ITGA2	GSE109604	0.865585946	knock down	up-regulate	6.26E-05
REG00014	EHD1	GSE109604	0.866659106	knock down	up-regulate	0.036720085
REG00014	PPCDC	GSE109604	0.868003836	knock down	up-regulate	0.005686043
REG00014	C16orf74	GSE109604	0.871363028	knock down	up-regulate	3.97E-06
REG00014	KDM5B	GSE109604	0.872885682	knock down	up-regulate	2.72E-05

REG00014	FRMD5	GSE109604	0.873312647	knock down	up-regulate	0.007627559
REG00014	SOD1	GSE109604	0.876489538	knock down	up-regulate	0.04002362
REG00014	PPT1	GSE109604	0.876801356	knock down	up-regulate	0.00038673
REG00014	ILDR2	GSE109604	0.879135183	knock down	up-regulate	0.022137172
REG00014	SERINC3	GSE109604	0.879377503	knock down	up-regulate	0.043271175
REG00014	KLHDC10	GSE109604	0.879607686	knock down	up-regulate	1.97E-20
REG00014	PLEKHD1	GSE109604	0.883300303	knock down	up-regulate	0.005106543
REG00014	DNM2	GSE109604	0.88351361	knock down	up-regulate	0.007554848
REG00014	FBXO25	GSE109604	0.883788923	knock down	up-regulate	3.69E-06
REG00014	B3GAT3	GSE109604	0.885093057	knock down	up-regulate	3.49E-05
REG00014	DOCK11	GSE109604	0.890044343	knock down	up-regulate	0.001299587
REG00014	FBXL8	GSE109604	0.890097215	knock down	up-regulate	1.72E-286
REG00014	ASB1	GSE109604	0.891701112	knock down	up-regulate	0.000173304
REG00014	GALNT18	GSE109604	0.894033664	knock down	up-regulate	5.55E-05
REG00014	CARD9	GSE109604	0.896358973	knock down	up-regulate	0.024271462
REG00014	ZNF428	GSE109604	0.897428392	knock down	up-regulate	1.51E-06
REG00014	ARHGEF39	GSE109604	0.897896158	knock down	up-regulate	0.045956531
REG00014	C11orf1	GSE109604	0.90041337	knock down	up-regulate	0.008445617
REG00014	HMGN3	GSE109604	0.900789682	knock down	up-regulate	0.033291661
REG00014	TOR2A	GSE109604	0.905204373	knock down	up-regulate	0.001008808
REG00014	PPRC1	GSE109604	0.9079	knock down	up-regulate	0.0001494
REG00014	KLHL2	GSE109604	0.908447102	knock down	up-regulate	0.049636126
REG00014	MAML1	GSE109604	0.909825844	knock down	up-regulate	1.38E-09
REG00014	CCT3	GSE109604	0.910498489	knock down	up-regulate	0.037172535
REG00014	NECTIN1	GSE109604	0.911301486	knock down	up-regulate	0.005811293
REG00014	CASP6	GSE109604	0.912405373	knock down	up-regulate	0.003110745
REG00014	NOG	GSE109604	0.914872438	knock down	up-regulate	0.013062042
REG00014	POLR2C	GSE109604	0.915935126	knock down	up-regulate	0.000350363
REG00014	CD9	GSE109604	0.916117892	knock down	up-regulate	0.018319768
REG00014	CALB2	GSE109604	0.917048009	knock down	up-regulate	0.007113071
REG00014	D2HGDH	GSE109604	0.917133966	knock down	up-regulate	0.008866141
REG00014	OSBPL7	GSE109604	0.918328311	knock down	up-regulate	0.016525377
REG00014	AAK1	GSE109604	0.919809441	knock down	up-regulate	0.003528617
REG00014	ARL6IP6	GSE109604	0.922137956	knock down	up-regulate	5.29E-05
REG00014	SHC3	GSE109604	0.92281043	knock down	up-regulate	0.000750042
REG00014	CD63	GSE109604	0.924679704	knock down	up-regulate	0.007373506
REG00014	KLHL4	GSE109604	0.924890656	knock down	up-regulate	0.000174751
REG00014	NGEF	GSE109604	0.926205615	knock down	up-regulate	0.037267696
REG00014	RHOBTB3	GSE109604	0.927786385	knock down	up-regulate	0.009863687
REG00014	HLA-DPA1	GSE109604	0.92794624	knock down	up-regulate	0.000378649
REG00014	XAB2	GSE109604	0.930369418	knock down	up-regulate	2.49E-09
REG00014	C1RL	GSE109604	0.932596867	knock down	up-regulate	0.00090554
REG00014	ERCC2	GSE109604	0.934085078	knock down	up-regulate	0.026457149
REG00014	MIPEP	GSE109604	0.934151081	knock down	up-regulate	0.000669516
REG00014	RIPPLY2	GSE109604	0.935414586	knock down	up-regulate	0.000131147
REG00014	SMIM29	GSE109604	0.937160975	knock down	up-regulate	0.030133671
REG00014	ECH1	GSE109604	0.938744948	knock down	up-regulate	0.000279831
REG00014	CDH22	GSE109604	0.939412061	knock down	up-regulate	0.000123401
REG00014	CPEB1	GSE109604	0.9400167	knock down	up-regulate	1.98E-05
REG00014	FBXL17	GSE109604	0.94133918	knock down	up-regulate	1.89E-08

REG00014	KPNA2	GSE109604	0.94439931	knock down	up-regulate	0.000957801
REG00014	DPYSL2	GSE109604	0.944712003	knock down	up-regulate	7.59E-05
REG00014	LBH	GSE109604	0.946125622	knock down	up-regulate	4.88E-15
REG00014	MIB1	GSE109604	0.946737832	knock down	up-regulate	0.004787828
REG00014	SMKR1	GSE109604	0.95035664	knock down	up-regulate	0.001019298
REG00014	MRPL47	GSE109604	0.950801799	knock down	up-regulate	0.005515789
REG00014	MYOF	GSE109604	0.954430646	knock down	up-regulate	0.006855585
REG00014	JRK	GSE109604	0.959449095	knock down	up-regulate	3.30E-06
REG00014	MTO1	GSE109604	0.960871649	knock down	up-regulate	0.011225535
REG00014	RPN1	GSE109604	0.963391969	knock down	up-regulate	0.002669097
REG00014	RUNX1T1	GSE109604	0.967655934	knock down	up-regulate	0.005903704
REG00014	PPA2	GSE109604	0.969410993	knock down	up-regulate	0.000149672
REG00014	CCDC122	GSE109604	0.9696301	knock down	up-regulate	0.002962262
REG00014	KLHL20	GSE109604	0.970837144	knock down	up-regulate	2.06E-21
REG00014	ARHGDIA	GSE109604	0.972263613	knock down	up-regulate	0.019378858
REG00014	F8	GSE109604	0.973705118	knock down	up-regulate	5.49E-05
REG00014	CLIP4	GSE109604	0.977523769	knock down	up-regulate	0.00012247
REG00014	ABHD16B	GSE109604	0.979384691	knock down	up-regulate	0.008376981
REG00014	ZSWIM7	GSE109604	0.979938519	knock down	up-regulate	0.029509835
REG00014	TPGS1	GSE109604	0.981897809	knock down	up-regulate	0.010457732
REG00014	TOP3B	GSE109604	0.983835356	knock down	up-regulate	0.005351236
REG00014	DAB2	GSE109604	0.984841143	knock down	up-regulate	0.004501416
REG00014	ARHGEF19	GSE109604	0.985873915	knock down	up-regulate	0.023944701
REG00014	KIF27	GSE109604	0.987278173	knock down	up-regulate	5.25E-20
REG00014	FASN	GSE109604	0.988767275	knock down	up-regulate	6.23E-06
REG00014	OXLD1	GSE109604	0.991422346	knock down	up-regulate	0.020824227
REG00014	HMGCR	GSE109604	0.993665676	knock down	up-regulate	0.003127284
REG00014	GNL3	GSE109604	0.994040951	knock down	up-regulate	6.66E-11
REG00014	CTSW	GSE109604	0.994127764	knock down	up-regulate	0.006246211
REG00014	APOL6	GSE109604	0.995805159	knock down	up-regulate	0.000223997
REG00014	MLEC	GSE109604	0.996042649	knock down	up-regulate	0.000107492
REG00014	ADAMTS1	GSE109604	0.99679917	knock down	up-regulate	0.015274572
REG00014	MACROD1	GSE90639	1.00E+00	.	.	.
REG00014	GALNT1	GSE90639	1.00E+00	.	.	.
REG00014	NUCKS1	GSE109604	1.000421914	knock down	up-regulate	7.98E-06
REG00014	GOLGA7	GSE109604	1.003111979	knock down	up-regulate	0.001260895
REG00014	ZNF695	GSE109604	1.004835929	knock down	up-regulate	0.022117545
REG00014	PILRB	GSE109604	1.005285109	knock down	up-regulate	0.014291277
REG00014	ELP5	GSE109604	1.006723014	knock down	up-regulate	2.78E-05
REG00014	TM4SF1	GSE109604	1.006911592	knock down	up-regulate	0.004325877
REG00014	STAG1	GSE90639	1.01E+00	.	.	.
REG00014	PRELID1	GSE90639	1.01E+00	.	.	.
REG00014	PPIA	GSE90639	1.01E+00	.	.	.
REG00014	RFC3	GSE90639	1.01E+00	.	.	.
REG00014	DUS3L	GSE109604	1.010406658	knock down	up-regulate	2.54E-08
REG00014	NLRP1	GSE109604	1.013102728	knock down	up-regulate	0.001567347
REG00014	RNF44	GSE109604	1.014642233	knock down	up-regulate	0.000220055
REG00014	NUDT17	GSE109604	1.016852434	knock down	up-regulate	0.002599765
REG00014	RPL14	GSE90639	1.02E+00	.	.	.
REG00014	CNBP	GSE90639	1.02E+00	.	.	.

REG00014	NHP2	GSE90639	1.02E+00	.	.	.
REG00014	SF3B6	GSE90639	1.02E+00	.	.	.
REG00014	RAD21	GSE90639	1.02E+00	.	.	.
REG00014	FAM43A	GSE109604	1.02216918	knock down	up-regulate	0.043914397
REG00014	TCTN3	GSE109604	1.022871	knock down	up-regulate	0.000216182
REG00014	TYW1	GSE109604	1.025217005	knock down	up-regulate	0.000540507
REG00014	AADAT	GSE109604	1.028969019	knock down	up-regulate	0.031586918
REG00014	NCAPH	GSE90639	1.03E+00	.	.	.
REG00014	CYCS	GSE90639	1.03E+00	.	.	.
REG00014	VTA1	GSE90639	1.03E+00	.	.	.
REG00014	EIF5	GSE90639	1.03E+00	.	.	.
REG00014	ERH	GSE90639	1.03E+00	.	.	.
REG00014	FUCA2	GSE90639	1.03E+00	.	.	.
REG00014	CENPT	GSE109604	1.031157039	knock down	up-regulate	0.005137671
REG00014	HSD17B4	GSE109604	1.0315011	knock down	up-regulate	0.018546902
REG00014	TTLL12	GSE109604	1.032459424	knock down	up-regulate	0.00619861
REG00014	ECD	GSE109604	1.032667606	knock down	up-regulate	0.018178302
REG00014	TEAD4	GSE109604	1.033402671	knock down	up-regulate	0.045398433
REG00014	MTG2	GSE109604	1.033449253	knock down	up-regulate	6.23E-06
REG00014	GNG12	GSE109604	1.036864893	knock down	up-regulate	0.001585661
REG00014	IL17RE	GSE109604	1.036953131	knock down	up-regulate	0.000728907
REG00014	PARVB	GSE109604	1.037312091	knock down	up-regulate	0.01678861
REG00014	GTF3C3	GSE109604	1.038500922	knock down	up-regulate	2.19E-05
REG00014	U2SURP	GSE90639	1.04E+00	.	.	.
REG00014	NCAM1	GSE109604	1.040545046	knock down	up-regulate	0.000469187
REG00014	IL6R	GSE109604	1.041939923	knock down	up-regulate	6.26E-05
REG00014	ARHGAP17	GSE109604	1.046841639	knock down	up-regulate	0.008872035
REG00014	ANKRD10	GSE109604	1.049469114	knock down	up-regulate	0.014487258
REG00014	DHPS	GSE109604	1.049786567	knock down	up-regulate	4.91E-06
REG00014	GCK	GSE109604	1.049924479	knock down	up-regulate	0.000850898
REG00014	TRA2A	GSE90639	1.05E+00	.	.	.
REG00014	TMEM14C	GSE90639	1.05E+00	.	.	.
REG00014	RTCB	GSE90639	1.05E+00	.	.	.
REG00014	CCDC86	GSE90639	1.05E+00	.	.	.
REG00014	HNRNPH1	GSE90639	1.05E+00	.	.	.
REG00014	HSPA4	GSE90639	1.05E+00	.	.	.
REG00014	TERB1	GSE109604	1.051772412	knock down	up-regulate	0.046871797
REG00014	EIF4A2	GSE109604	1.053581816	knock down	up-regulate	0.006721047
REG00014	ATXN7L2	GSE109604	1.057748071	knock down	up-regulate	0.028415561
REG00014	SMN2	GSE90639	1.06E+00	.	.	.
REG00014	GOLIM4	GSE90639	1.06E+00	.	.	.
REG00014	AATF	GSE90639	1.06E+00	.	.	.
REG00014	CSNK1A1	GSE90639	1.06E+00	.	.	.
REG00014	DHFR	GSE90639	1.06E+00	.	.	.
REG00014	EDEM1	GSE109604	1.061977873	knock down	up-regulate	0.02390129
REG00014	ARHGAP39	GSE109604	1.062092987	knock down	up-regulate	0.025689581
REG00014	TYMS	GSE90639	1.07E+00	.	.	.
REG00014	C2orf47	GSE90639	1.07E+00	.	.	.
REG00014	FEM1B	GSE90639	1.07E+00	.	.	.
REG00014	POLR2I	GSE90639	1.07E+00	.	.	.

REG00014	RANBP6	GSE90639	1.07E+00	.	.	.
REG00014	ALKBH2	GSE109604	1.072777213	knock down	up-regulate	3.61E-05
REG00014	ITGA1	GSE109604	1.074493744	knock down	up-regulate	0.000141507
REG00014	JAG1	GSE109604	1.075358368	knock down	up-regulate	0.000284808
REG00014	SIPA1L3	GSE109604	1.076019914	knock down	up-regulate	0.001720506
REG00014	NOL10	GSE90639	1.08E+00	.	.	.
REG00014	FCHSD2	GSE90639	1.08E+00	.	.	.
REG00014	CCBL2	GSE90639	1.08E+00	.	.	.
REG00014	MAT2A	GSE90639	1.08E+00	.	.	.
REG00014	PIGK	GSE90639	1.08E+00	.	.	.
REG00014	HAUS1	GSE109604	1.084909872	knock down	up-regulate	1.35E-30
REG00014	SEMA5A	GSE109604	1.08492239	knock down	up-regulate	0.021192144
REG00014	PIDD1	GSE109604	1.08492239	knock down	up-regulate	0.021192144
REG00014	MET	GSE109604	1.087080243	knock down	up-regulate	0.016075312
REG00014	HLA-C	GSE90639	1.09E+00	.	.	.
REG00014	PRPS1	GSE90639	1.09E+00	.	.	.
REG00014	SEMA6D	GSE109604	1.093362411	knock down	up-regulate	0.015402095
REG00014	MICALL1	GSE109604	1.093785088	knock down	up-regulate	0.001646976
REG00014	NBEAL2	GSE109604	1.093884189	knock down	up-regulate	0.002200572
REG00014	JAM3	GSE109604	1.094627643	knock down	up-regulate	7.73E-39
REG00014	KLHL29	GSE109604	1.099927625	knock down	up-regulate	0.032223454
REG00014	RPS23	GSE90639	1.10E+00	.	.	.
REG00014	C1QBP	GSE90639	1.10E+00	.	.	.
REG00014	VWA8	GSE90639	1.10E+00	.	.	.
REG00014	MCM3	GSE90639	1.10E+00	.	.	.
REG00014	NDUFB6	GSE90639	1.10E+00	.	.	.
REG00014	ALG8	GSE109604	1.101929685	knock down	up-regulate	0.009145916
REG00014	CFTR	GSE109604	1.102491211	knock down	up-regulate	0.005483982
REG00014	TTC32	GSE109604	1.102805733	knock down	up-regulate	0.006442303
REG00014	ARMCX5	GSE109604	1.103516174	knock down	up-regulate	0.000359519
REG00014	NID1	GSE109604	1.106891716	knock down	up-regulate	0.037866945
REG00014	COMMD3	GSE109604	1.107431363	knock down	up-regulate	0.00193006
REG00014	RPL13A	GSE90639	1.11E+00	.	.	.
REG00014	SRM	GSE90639	1.11E+00	.	.	.
REG00014	TOP2B	GSE90639	1.11E+00	.	.	.
REG00014	UCHL1	GSE90639	1.11E+00	.	.	.
REG00014	HIST1H2BF	GSE90639	1.11E+00	.	.	.
REG00014	SPRN	GSE109604	1.111023233	knock down	up-regulate	0.006020464
REG00014	PHC1	GSE109604	1.111554759	knock down	up-regulate	0.008102842
REG00014	PAPPA	GSE109604	1.111655363	knock down	up-regulate	0.036497713
REG00014	CALML4	GSE109604	1.118020973	knock down	up-regulate	0.001094254
REG00014	SMC4	GSE90639	1.12E+00	.	.	.
REG00014	CNOT6	GSE90639	1.12E+00	.	.	.
REG00014	SPEN	GSE90639	1.12E+00	.	.	.
REG00014	MIER3	GSE90639	1.12E+00	.	.	.
REG00014	NACA	GSE90639	1.12E+00	.	.	.
REG00014	PLS3	GSE90639	1.12E+00	.	.	.
REG00014	DKK2	GSE109604	1.122590363	knock down	up-regulate	5.73E-06
REG00014	EBLN2	GSE109604	1.124409719	knock down	up-regulate	0.003183179
REG00014	ATP6V0E1	GSE109604	1.127584824	knock down	up-regulate	4.96E-55

REG00014	SRP14	GSE90639	1.13E+00	.	.	.
REG00014	HSP90B1	GSE90639	1.13E+00	.	.	.
REG00014	DHX29	GSE90639	1.13E+00	.	.	.
REG00014	HMG20B	GSE109604	1.131297183	knock down	up-regulate	0.016260938
REG00014	CDK19	GSE109604	1.133634237	knock down	up-regulate	0.000311776
REG00014	NSG1	GSE109604	1.134191143	knock down	up-regulate	0.025490242
REG00014	EXTL3	GSE109604	1.135484078	knock down	up-regulate	3.31E-07
REG00014	C16orf86	GSE109604	1.135828626	knock down	up-regulate	0.033529887
REG00014	ASGR1	GSE109604	1.136428943	knock down	up-regulate	0.031852687
REG00014	MYL12B	GSE109604	1.137346615	knock down	up-regulate	0.031966432
REG00014	ZNF587B	GSE109604	1.139137375	knock down	up-regulate	0.005521107
REG00014	PRDX6	GSE109604	1.139891109	knock down	up-regulate	6.11E-06
REG00014	NOP14	GSE90639	1.14E+00	.	.	.
REG00014	HMGB1	GSE90639	1.14E+00	.	.	.
REG00014	PPP6C	GSE90639	1.14E+00	.	.	.
REG00014	MTFMT	GSE109604	1.140976681	knock down	up-regulate	0.002702893
REG00014	DECR1	GSE109604	1.143539145	knock down	up-regulate	0.010069331
REG00014	NKRF	GSE109604	1.146040602	knock down	up-regulate	8.65E-05
REG00014	EVC	GSE109604	1.147490766	knock down	up-regulate	0.028496683
REG00014	TFAM	GSE109604	1.147509863	knock down	up-regulate	0.002778972
REG00014	TAF11	GSE90639	1.15E+00	.	.	.
REG00014	APMAP	GSE90639	1.15E+00	.	.	.
REG00014	KIF15	GSE90639	1.15E+00	.	.	.
REG00014	COX7C	GSE90639	1.15E+00	.	.	.
REG00014	HSPA5	GSE90639	1.15E+00	.	.	.
REG00014	CPEB3	GSE109604	1.150140435	knock down	up-regulate	0.000845649
REG00014	TERT	GSE109604	1.150650099	knock down	up-regulate	0.030116861
REG00014	ZNF419	GSE109604	1.153007234	knock down	up-regulate	4.06E-14
REG00014	BLZF1	GSE109604	1.158631563	knock down	up-regulate	6.81E-14
REG00014	SMARCA5	GSE90639	1.16E+00	.	.	.
REG00014	ZIC2	GSE90639	1.16E+00	.	.	.
REG00014	DDX5	GSE90639	1.16E+00	.	.	.
REG00014	CCDC85B	GSE109604	1.164183556	knock down	up-regulate	0.002317374
REG00014	CYP2J2	GSE109604	1.168126521	knock down	up-regulate	0.000204792
REG00014	IRX4	GSE109604	1.169332864	knock down	up-regulate	5.48E-05
REG00014	CBS	GSE90639	1.17E+00	.	.	.
REG00014	C4orf46	GSE90639	1.17E+00	.	.	.
REG00014	ID2	GSE90639	1.17E+00	.	.	.
REG00014	PRDX5	GSE90639	1.17E+00	.	.	.
REG00014	LTBP4	GSE109604	1.172429136	knock down	up-regulate	9.72E-13
REG00014	CD4	GSE109604	1.17740146	knock down	up-regulate	0.046642727
REG00014	MAP2K3	GSE109604	1.1783507	knock down	up-regulate	0.005357939
REG00014	NUDT11	GSE109604	1.178554656	knock down	up-regulate	0.010554161
REG00014	RPL23A	GSE109604	1.179908362	knock down	up-regulate	6.72E-05
REG00014	RPS9	GSE90639	1.18E+00	.	.	.
REG00014	SNRNP70	GSE90639	1.18E+00	.	.	.
REG00014	BIRC6	GSE90639	1.18E+00	.	.	.
REG00014	CCT3	GSE90639	1.18E+00	.	.	.
REG00014	UQCRQ	GSE90639	1.18E+00	.	.	.
REG00014	EEF1A2	GSE90639	1.18E+00	.	.	.

REG00014	EIF3E	GSE90639	1.18E+00	.	.	.
REG00014	ATPIF1	GSE90639	1.18E+00	.	.	.
REG00014	SLAIN1	GSE109604	1.181549798	knock down	up-regulate	6.47E-06
REG00014	CSNK1E	GSE109604	1.183908373	knock down	up-regulate	0.000341423
REG00014	CYB561A3	GSE109604	1.184590402	knock down	up-regulate	0.000243786
REG00014	JAGN1	GSE109604	1.184890587	knock down	up-regulate	0.000351212
REG00014	GPT	GSE109604	1.187054094	knock down	up-regulate	0.007205648
REG00014	FAM104B	GSE109604	1.187077259	knock down	up-regulate	0.041116167
REG00014	EIF3L	GSE90639	1.19E+00	.	.	.
REG00014	IGF2R	GSE90639	1.19E+00	.	.	.
REG00014	ONECUT2	GSE90639	1.19E+00	.	.	.
REG00014	ZNF304	GSE109604	1.19838701	knock down	up-regulate	1.27E-08
REG00014	CCNA2	GSE90639	1.20E+00	.	.	.
REG00014	TIMM23	GSE90639	1.20E+00	.	.	.
REG00014	TXNRD3	GSE90639	1.20E+00	.	.	.
REG00014	EIF1	GSE90639	1.20E+00	.	.	.
REG00014	H3F3B	GSE90639	1.20E+00	.	.	.
REG00014	PSMC1	GSE90639	1.20E+00	.	.	.
REG00014	SCARF2	GSE109604	1.201737053	knock down	up-regulate	0.005751101
REG00014	PHLDB2	GSE109604	1.204215942	knock down	up-regulate	6.56E-05
REG00014	MAP3K10	GSE109604	1.205460778	knock down	up-regulate	0.021072011
REG00014	RPL37	GSE90639	1.21E+00	.	.	.
REG00014	ACTB	GSE90639	1.21E+00	.	.	.
REG00014	MRPL14	GSE90639	1.21E+00	.	.	.
REG00014	PRPF39	GSE90639	1.21E+00	.	.	.
REG00014	NDUFAF4	GSE90639	1.21E+00	.	.	.
REG00014	OFD1	GSE90639	1.21E+00	.	.	.
REG00014	PTPMT1	GSE90639	1.21E+00	.	.	.
REG00014	HMGN2	GSE90639	1.21E+00	.	.	.
REG00014	UTP15	GSE109604	1.21774469	knock down	up-regulate	0.002647913
REG00014	CAV1	GSE109604	1.218234295	knock down	up-regulate	2.75E-05
REG00014	RAET1E	GSE109604	1.21877406	knock down	up-regulate	0.000435927
REG00014	SLC25A5	GSE90639	1.22E+00	.	.	.
REG00014	TFAM	GSE90639	1.22E+00	.	.	.
REG00014	EIF3M	GSE90639	1.22E+00	.	.	.
REG00014	PRRC2C	GSE90639	1.22E+00	.	.	.
REG00014	PGAP1	GSE90639	1.22E+00	.	.	.
REG00014	HSPD1	GSE90639	1.22E+00	.	.	.
REG00014	JAK2	GSE90639	1.22E+00	.	.	.
REG00014	ATP5G3	GSE90639	1.22E+00	.	.	.
REG00014	UBE2T	GSE109604	1.228862297	knock down	up-regulate	0.00257795
REG00014	KIF13B	GSE109604	1.22892306	knock down	up-regulate	0.004765011
REG00014	MRPL12	GSE90639	1.23E+00	.	.	.
REG00014	C17orf58	GSE90639	1.23E+00	.	.	.
REG00014	GLUD1	GSE90639	1.23E+00	.	.	.
REG00014	MTHFD2	GSE90639	1.23E+00	.	.	.
REG00014	BDH1	GSE109604	1.232403472	knock down	up-regulate	0.003926579
REG00014	FMN2	GSE109604	1.234346643	knock down	up-regulate	6.43E-05
REG00014	MAST2	GSE109604	1.237602985	knock down	up-regulate	0.000817612
REG00014	MIB1	GSE90639	1.24E+00	.	.	.

REG00014	BOLA2	GSE90639	1.24E+00	.	.	.
REG00014	HSF2	GSE90639	1.24E+00	.	.	.
REG00014	GRPR	GSE109604	1.246576546	knock down	up-regulate	2.08E-16
REG00014	TBCA	GSE90639	1.25E+00	.	.	.
REG00014	CLIC4	GSE90639	1.25E+00	.	.	.
REG00014	CAMK1	GSE90639	1.25E+00	.	.	.
REG00014	FAM35A	GSE90639	1.25E+00	.	.	.
REG00014	CAPN15	GSE109604	1.250893257	knock down	up-regulate	0.00274795
REG00014	BBC3	GSE109604	1.256544824	knock down	up-regulate	0.000248386
REG00014	AGO2	GSE109604	1.256838408	knock down	up-regulate	2.20E-05
REG00014	DARS2	GSE109604	1.258852095	knock down	up-regulate	7.54E-06
REG00014	ATG16L2	GSE109604	1.259630392	knock down	up-regulate	7.28E-18
REG00014	SMS	GSE90639	1.26E+00	.	.	.
REG00014	PDIA4	GSE90639	1.26E+00	.	.	.
REG00014	DZIP3	GSE90639	1.26E+00	.	.	.
REG00014	IRAK1	GSE90639	1.26E+00	.	.	.
REG00014	ABI3BP	GSE109604	1.262156526	knock down	up-regulate	0.00134422
REG00014	JPT2	GSE109604	1.262426466	knock down	up-regulate	0.00050354
REG00014	ABHD2	GSE109604	1.265207515	knock down	up-regulate	0.000686673
REG00014	G3BP1	GSE109604	1.267003646	knock down	up-regulate	6.21E-24
REG00014	ST13	GSE90639	1.27E+00	.	.	.
REG00014	TRIT1	GSE90639	1.27E+00	.	.	.
REG00014	HIST2H2BF	GSE90639	1.27E+00	.	.	.
REG00014	CWC22	GSE90639	1.27E+00	.	.	.
REG00014	MPG	GSE109604	1.278096933	knock down	up-regulate	0.001779927
REG00014	TANK	GSE109604	1.278590737	knock down	up-regulate	0.001651521
REG00014	CCT8	GSE90639	1.28E+00	.	.	.
REG00014	LARP1	GSE90639	1.28E+00	.	.	.
REG00014	SMIM32	GSE109604	1.281813887	knock down	up-regulate	6.95E-05
REG00014	TBCD	GSE109604	1.282248681	knock down	up-regulate	0.002499143
REG00014	NUPR1	GSE109604	1.282299305	knock down	up-regulate	5.50E-05
REG00014	CSPG4	GSE109604	1.284479941	knock down	up-regulate	0.049038707
REG00014	RPS27A	GSE90639	1.29E+00	.	.	.
REG00014	XRCC5	GSE90639	1.29E+00	.	.	.
REG00014	HNRNPD	GSE90639	1.29E+00	.	.	.
REG00014	CYBRD1	GSE109604	1.293921203	knock down	up-regulate	0.000153485
REG00014	NUTM2D	GSE109604	1.293939954	knock down	up-regulate	0.005262481
REG00014	ACBD3	GSE109604	1.295735403	knock down	up-regulate	0.003997804
REG00014	CHEK1	GSE109604	1.296530112	knock down	up-regulate	0.019732652
REG00014	RPLP2	GSE90639	1.30E+00	.	.	.
REG00014	GHITM	GSE90639	1.30E+00	.	.	.
REG00014	CMAS	GSE90639	1.30E+00	.	.	.
REG00014	HNRNPA3	GSE90639	1.30E+00	.	.	.
REG00014	DGUOK	GSE90639	1.30E+00	.	.	.
REG00014	JTB	GSE90639	1.30E+00	.	.	.
REG00014	NDUFV2	GSE90639	1.30E+00	.	.	.
REG00014	ABHD17A	GSE109604	1.302750814	knock down	up-regulate	0.000140051
REG00014	C1QBP	GSE109604	1.307611014	knock down	up-regulate	4.66E-05
REG00014	CHCHD10	GSE90639	1.31E+00	.	.	.
REG00014	DCP2	GSE90639	1.31E+00	.	.	.

REG00014	PGLS	GSE90639	1.31E+00	.	.	.
REG00014	RAD23B	GSE90639	1.31E+00	.	.	.
REG00014	PRIM2	GSE109604	1.318224262	knock down	up-regulate	0.000473288
REG00014	RPS13	GSE90639	1.32E+00	.	.	.
REG00014	SNRPD2	GSE90639	1.32E+00	.	.	.
REG00014	SRP72	GSE90639	1.32E+00	.	.	.
REG00014	SMC2	GSE90639	1.32E+00	.	.	.
REG00014	CLTC	GSE90639	1.32E+00	.	.	.
REG00014	CEBPZ	GSE90639	1.32E+00	.	.	.
REG00014	LGI3	GSE109604	1.320399106	knock down	up-regulate	0.027548756
REG00014	GPRASP2	GSE109604	1.325562538	knock down	up-regulate	9.78E-22
REG00014	ARMC6	GSE109604	1.327130239	knock down	up-regulate	7.77E-08
REG00014	FBLN7	GSE109604	1.331412411	knock down	up-regulate	1.72E-12
REG00014	YIPF1	GSE109604	1.33425615	knock down	up-regulate	6.50E-15
REG00014	HSD17B11	GSE109604	1.335268136	knock down	up-regulate	1.50E-05
REG00014	RFT1	GSE109604	1.339618748	knock down	up-regulate	0.026280454
REG00014	DDX21	GSE90639	1.34E+00	.	.	.
REG00014	LRRC40	GSE109604	1.343354814	knock down	up-regulate	7.88E-05
REG00014	RPL9	GSE90639	1.35E+00	.	.	.
REG00014	GOPC	GSE90639	1.35E+00	.	.	.
REG00014	DDX50	GSE90639	1.35E+00	.	.	.
REG00014	DNAAF2	GSE109604	1.350187946	knock down	up-regulate	0.002166931
REG00014	ZNF185	GSE109604	1.351254368	knock down	up-regulate	0.042657928
REG00014	ADORA1	GSE109604	1.351870857	knock down	up-regulate	0.003750386
REG00014	DAB2IP	GSE109604	1.358251378	knock down	up-regulate	0.001487598
REG00014	RPL35A	GSE90639	1.36E+00	.	.	.
REG00014	RPL36	GSE90639	1.36E+00	.	.	.
REG00014	TPM1	GSE109604	1.361749918	knock down	up-regulate	0.000409488
REG00014	TGFBR3L	GSE109604	1.362598955	knock down	up-regulate	0.001814935
REG00014	CYB5D2	GSE109604	1.368932836	knock down	up-regulate	0.000404584
REG00014	PSPC1	GSE90639	1.37E+00	.	.	.
REG00014	TOMM20	GSE90639	1.37E+00	.	.	.
REG00014	C9orf40	GSE90639	1.37E+00	.	.	.
REG00014	HMGB2	GSE90639	1.37E+00	.	.	.
REG00014	ZW10	GSE109604	1.371094986	knock down	up-regulate	0.006753127
REG00014	CKAP5	GSE109604	1.372721557	knock down	up-regulate	0.001086454
REG00014	LRFN4	GSE109604	1.378756817	knock down	up-regulate	0.016309087
REG00014	SMC1A	GSE90639	1.38E+00	.	.	.
REG00014	NDUFB3	GSE90639	1.38E+00	.	.	.
REG00014	GTF2B	GSE109604	1.3800664	knock down	up-regulate	0.049126929
REG00014	DCST1	GSE109604	1.380253368	knock down	up-regulate	5.89E-05
REG00014	FAM118B	GSE109604	1.384284966	knock down	up-regulate	0.022322802
REG00014	ARMC7	GSE109604	1.387282923	knock down	up-regulate	0.001503932
REG00014	SLK	GSE90639	1.39E+00	.	.	.
REG00014	MORF4L2	GSE90639	1.39E+00	.	.	.
REG00014	USP41	GSE90639	1.39E+00	.	.	.
REG00014	MAP3K2	GSE90639	1.39E+00	.	.	.
REG00014	PGK1	GSE90639	1.39E+00	.	.	.
REG00014	RAD54L	GSE90639	1.39E+00	.	.	.
REG00014	LYSMD2	GSE109604	1.393901639	knock down	up-regulate	7.46E-06

REG00014	ERBB2	GSE109604	1.395071798	knock down	up-regulate	0.030066939
REG00014	TCP1	GSE90639	1.40E+00	.	.	.
REG00014	HNRNPH3	GSE90639	1.40E+00	.	.	.
REG00014	HMGN2	GSE109604	1.401839056	knock down	up-regulate	0.023952104
REG00014	TREX1	GSE109604	1.407720461	knock down	up-regulate	0.031557619
REG00014	MRPL15	GSE90639	1.41E+00	.	.	.
REG00014	ANKRD10	GSE90639	1.41E+00	.	.	.
REG00014	PARN	GSE109604	1.411161831	knock down	up-regulate	0.009199013
REG00014	JRKL	GSE109604	1.412131033	knock down	up-regulate	4.91E-05
REG00014	DECR2	GSE109604	1.414706061	knock down	up-regulate	0.000162948
REG00014	EPN1	GSE109604	1.417649267	knock down	up-regulate	0.013802901
REG00014	NUP35	GSE109604	1.419517622	knock down	up-regulate	0.002987144
REG00014	ZDHHC2	GSE90639	1.42E+00	.	.	.
REG00014	CUL1	GSE90639	1.42E+00	.	.	.
REG00014	IARS2	GSE90639	1.42E+00	.	.	.
REG00014	GARS	GSE90639	1.42E+00	.	.	.
REG00014	KRTAP4-7	GSE109604	1.420342017	knock down	up-regulate	5.21E-08
REG00014	CKMT1B	GSE109604	1.422110805	knock down	up-regulate	0.000257639
REG00014	PDLIM1	GSE109604	1.423507087	knock down	up-regulate	0.000170379
REG00014	CUTC	GSE109604	1.42399778	knock down	up-regulate	0.000122215
REG00014	ITPRIP	GSE109604	1.424354573	knock down	up-regulate	1.34E-05
REG00014	LPCAT4	GSE109604	1.4268711	knock down	up-regulate	0.003106614
REG00014	RPL28	GSE90639	1.43E+00	.	.	.
REG00014	RPS8	GSE90639	1.43E+00	.	.	.
REG00014	MRPL53	GSE90639	1.43E+00	.	.	.
REG00014	GSPT1	GSE90639	1.43E+00	.	.	.
REG00014	RHOA	GSE90639	1.43E+00	.	.	.
REG00014	BPNT1	GSE109604	1.431604078	knock down	up-regulate	0.001326877
REG00014	CHD1L	GSE109604	1.433150695	knock down	up-regulate	0.011647905
REG00014	MTLN	GSE109604	1.433206713	knock down	up-regulate	0.007010891
REG00014	KLF2	GSE109604	1.437121902	knock down	up-regulate	1.09E-36
REG00014	ALYREF	GSE90639	1.44E+00	.	.	.
REG00014	MTDH	GSE90639	1.44E+00	.	.	.
REG00014	NUP153	GSE90639	1.44E+00	.	.	.
REG00014	ANKMY1	GSE109604	1.448991436	knock down	up-regulate	0.000621721
REG00014	ANP32B	GSE90639	1.45E+00	.	.	.
REG00014	HNRNPC	GSE90639	1.45E+00	.	.	.
REG00014	CYB561	GSE109604	1.455849986	knock down	up-regulate	8.49E-05
REG00014	RPL15	GSE90639	1.46E+00	.	.	.
REG00014	RPL17	GSE90639	1.46E+00	.	.	.
REG00014	CEP290	GSE90639	1.46E+00	.	.	.
REG00014	G3BP1	GSE90639	1.46E+00	.	.	.
REG00014	ITM2B	GSE90639	1.46E+00	.	.	.
REG00014	SF3B1	GSE90639	1.47E+00	.	.	.
REG00014	DICER1	GSE90639	1.47E+00	.	.	.
REG00014	DCXR	GSE90639	1.47E+00	.	.	.
REG00014	NDUFB10	GSE90639	1.47E+00	.	.	.
REG00014	CYC1	GSE109604	1.470977134	knock down	up-regulate	0.011754072
REG00014	ASPSCR1	GSE109604	1.473824342	knock down	up-regulate	0.000128461
REG00014	CGB8	GSE109604	1.479188329	knock down	up-regulate	0.012660772

REG00014	CDT1	GSE90639	1.48E+00	.	.	.
REG00014	DNAJA1	GSE90639	1.48E+00	.	.	.
REG00014	TWIST2	GSE109604	1.483366438	knock down	up-regulate	0.000949506
REG00014	NOP56	GSE90639	1.49E+00	.	.	.
REG00014	CCNG1	GSE90639	1.49E+00	.	.	.
REG00014	TGS1	GSE90639	1.49E+00	.	.	.
REG00014	BOD1	GSE90639	1.49E+00	.	.	.
REG00014	C10orf88	GSE90639	1.49E+00	.	.	.
REG00014	GPX4	GSE90639	1.49E+00	.	.	.
REG00014	HNRNPU	GSE90639	1.49E+00	.	.	.
REG00014	TOP3B	GSE90639	1.50E+00	.	.	.
REG00014	IMPDH2	GSE90639	1.50E+00	.	.	.
REG00014	NDUFA1	GSE90639	1.50E+00	.	.	.
REG00014	C12orf57	GSE109604	1.500273598	knock down	up-regulate	0.007526428
REG00014	ARF1	GSE109604	1.512438811	knock down	up-regulate	5.38E-05
REG00014	ARRB2	GSE109604	1.518006719	knock down	up-regulate	4.98E-06
REG00014	PLXNA2	GSE109604	1.519355378	knock down	up-regulate	1.68E-05
REG00014	UBC	GSE90639	1.52E+00	.	.	.
REG00014	MZT1	GSE90639	1.52E+00	.	.	.
REG00014	UBE2K	GSE90639	1.52E+00	.	.	.
REG00014	RAP2A	GSE90639	1.52E+00	.	.	.
REG00014	ACCS	GSE109604	1.522259725	knock down	up-regulate	0.004855921
REG00014	CCNB1	GSE109604	1.526725875	knock down	up-regulate	0.001274949
REG00014	RPL11	GSE90639	1.53E+00	.	.	.
REG00014	KDELR2	GSE90639	1.53E+00	.	.	.
REG00014	ZFYVE28	GSE109604	1.53067887	knock down	up-regulate	0.003243512
REG00014	CDKN2C	GSE109604	1.535885574	knock down	up-regulate	0.003534637
REG00014	PTMA	GSE90639	1.54E+00	.	.	.
REG00014	DNHD1	GSE109604	1.543776163	knock down	up-regulate	0.002362511
REG00014	HHIP	GSE109604	1.544254907	knock down	up-regulate	5.66E-98
REG00014	DLGAP5	GSE90639	1.55E+00	.	.	.
REG00014	EIF4G2	GSE90639	1.55E+00	.	.	.
REG00014	ZC3H3	GSE109604	1.555309499	knock down	up-regulate	0.044287714
REG00014	RPN2	GSE90639	1.56E+00	.	.	.
REG00014	BRD2	GSE90639	1.56E+00	.	.	.
REG00014	PIEZO1	GSE109604	1.569561262	knock down	up-regulate	4.26E-07
REG00014	POLR3H	GSE109604	1.569561263	knock down	up-regulate	4.26E-07
REG00014	TMEM57	GSE90639	1.57E+00	.	.	.
REG00014	EID2	GSE90639	1.57E+00	.	.	.
REG00014	UXT	GSE90639	1.58E+00	.	.	.
REG00014	EEF1B2	GSE90639	1.58E+00	.	.	.
REG00014	CCT6A	GSE90639	1.59E+00	.	.	.
REG00014	BCAS2	GSE90639	1.60E+00	.	.	.
REG00014	GALNT6	GSE109604	1.60401905	knock down	up-regulate	9.27E-07
REG00014	CDK5R1	GSE109604	1.607766911	knock down	up-regulate	0.001491187
REG00014	ZNF460	GSE90639	1.61E+00	.	.	.
REG00014	GPR171	GSE90639	1.61E+00	.	.	.
REG00014	TXNDC5	GSE109604	1.618895111	knock down	up-regulate	0.000161898
REG00014	RPS24	GSE90639	1.62E+00	.	.	.
REG00014	KHDRBS1	GSE90639	1.62E+00	.	.	.

REG00014	AHCTF1	GSE90639	1.62E+00	.	.	.
REG00014	EIF4A1	GSE90639	1.62E+00	.	.	.
REG00014	EPRS	GSE90639	1.62E+00	.	.	.
REG00014	HSPA8	GSE90639	1.62E+00	.	.	.
REG00014	HTATSF1	GSE90639	1.62E+00	.	.	.
REG00014	MAZ	GSE90639	1.62E+00	.	.	.
REG00014	ATIC	GSE90639	1.62E+00	.	.	.
REG00014	SHISAL2A	GSE109604	1.626367454	knock down	up-regulate	1.02E-05
REG00014	SRSF2	GSE90639	1.63E+00	.	.	.
REG00014	EIF4B	GSE90639	1.63E+00	.	.	.
REG00014	DCUN1D3	GSE109604	1.63971627	knock down	up-regulate	1.78E-06
REG00014	UQCR10	GSE90639	1.64E+00	.	.	.
REG00014	API5	GSE90639	1.64E+00	.	.	.
REG00014	PMAIP1	GSE90639	1.64E+00	.	.	.
REG00014	ASTN2	GSE109604	1.64103356	knock down	up-regulate	0.016384301
REG00014	DCBLD2	GSE109604	1.647683456	knock down	up-regulate	3.99E-05
REG00014	NUDT4P1	GSE90639	1.65E+00	.	.	.
REG00014	SMC3	GSE90639	1.65E+00	.	.	.
REG00014	RAN	GSE90639	1.65E+00	.	.	.
REG00014	FAM104A	GSE109604	1.652326517	knock down	up-regulate	0.04938769
REG00014	RMC1	GSE109604	1.659153086	knock down	up-regulate	5.40E-06
REG00014	RPL34	GSE90639	1.66E+00	.	.	.
REG00014	RPL41	GSE90639	1.66E+00	.	.	.
REG00014	PRPF40A	GSE90639	1.66E+00	.	.	.
REG00014	EIF4A2	GSE90639	1.66E+00	.	.	.
REG00014	PLEKHG5	GSE109604	1.668342965	knock down	up-regulate	0.047014134
REG00014	DDX39B	GSE90639	1.67E+00	.	.	.
REG00014	COX5A	GSE90639	1.67E+00	.	.	.
REG00014	SASS6	GSE90639	1.67E+00	.	.	.
REG00014	CHAC1	GSE90639	1.67E+00	.	.	.
REG00014	TMEM151B	GSE109604	1.676485379	knock down	up-regulate	0.000383563
REG00014	CDCA2	GSE90639	1.68E+00	.	.	.
REG00014	PDCD5	GSE90639	1.68E+00	.	.	.
REG00014	GTF2H3	GSE109604	1.686425663	knock down	up-regulate	1.23E-20
REG00014	LYRM9	GSE109604	1.687699664	knock down	up-regulate	0.000116399
REG00014	VIM	GSE109604	1.688559451	knock down	up-regulate	0.006823533
REG00014	RPS12	GSE90639	1.69E+00	.	.	.
REG00014	SRSF3	GSE90639	1.69E+00	.	.	.
REG00014	EID1	GSE90639	1.69E+00	.	.	.
REG00014	C14orf166	GSE90639	1.69E+00	.	.	.
REG00014	ARL6IP1	GSE90639	1.69E+00	.	.	.
REG00014	ATP5L	GSE90639	1.70E+00	.	.	.
REG00014	CCNH	GSE90639	1.70E+00	.	.	.
REG00014	PGRMC1	GSE90639	1.70E+00	.	.	.
REG00014	ZNF695	GSE90639	1.70E+00	.	.	.
REG00014	EML1	GSE109604	1.701899463	knock down	up-regulate	0.000136794
REG00014	USP35	GSE109604	1.716664052	knock down	up-regulate	0.001471305
REG00014	ASPM	GSE90639	1.72E+00	.	.	.
REG00014	HIST3H2A	GSE90639	1.72E+00	.	.	.
REG00014	PNISR	GSE90639	1.72E+00	.	.	.

REG00014	FOXF1	GSE109604	1.726970952	knock down	up-regulate	0.01334093
REG00014	RPL3	GSE90639	1.73E+00	.	.	.
REG00014	NBAS	GSE90639	1.73E+00	.	.	.
REG00014	CSE1L	GSE90639	1.73E+00	.	.	.
REG00014	EIF3A	GSE90639	1.73E+00	.	.	.
REG00014	NNT	GSE90639	1.73E+00	.	.	.
REG00014	LAT2	GSE109604	1.734219742	knock down	up-regulate	0.012086562
REG00014	WIPI1	GSE109604	1.734474473	knock down	up-regulate	3.49E-26
REG00014	ATXN10	GSE90639	1.74E+00	.	.	.
REG00014	GNB2L1	GSE90639	1.74E+00	.	.	.
REG00014	MARF1	GSE109604	1.741021306	knock down	up-regulate	0.018267602
REG00014	TMEM116	GSE109604	1.746178371	knock down	up-regulate	0.000114915
REG00014	TCF12	GSE109604	1.748379041	knock down	up-regulate	0.000816922
REG00014	RPS5	GSE90639	1.75E+00	.	.	.
REG00014	MAP2	GSE109604	1.7520519	knock down	up-regulate	0.005912203
REG00014	CHCHD2	GSE90639	1.76E+00	.	.	.
REG00014	DCUN1D5	GSE90639	1.76E+00	.	.	.
REG00014	MYH10	GSE90639	1.76E+00	.	.	.
REG00014	KSR1	GSE109604	1.761450576	knock down	up-regulate	5.20E-11
REG00014	ATP2A1	GSE109604	1.764812615	knock down	up-regulate	5.44E-54
REG00014	STOML2	GSE90639	1.77E+00	.	.	.
REG00014	GLO1	GSE90639	1.77E+00	.	.	.
REG00014	OAZ1	GSE90639	1.77E+00	.	.	.
REG00014	RPL38	GSE90639	1.78E+00	.	.	.
REG00014	RPLP0	GSE90639	1.78E+00	.	.	.
REG00014	RPS14	GSE90639	1.78E+00	.	.	.
REG00014	ZNF703	GSE90639	1.78E+00	.	.	.
REG00014	CTDSPL	GSE109604	1.782781368	knock down	up-regulate	0.000300771
REG00014	VPS51	GSE109604	1.786664505	knock down	up-regulate	3.18E-15
REG00014	HIST2H2AB	GSE90639	1.80E+00	.	.	.
REG00014	AHCYL1	GSE109604	1.809054814	knock down	up-regulate	0.004163122
REG00014	LUC7L3	GSE90639	1.81E+00	.	.	.
REG00014	NDUFA8	GSE90639	1.81E+00	.	.	.
REG00014	CENPH	GSE109604	1.81326509	knock down	up-regulate	0.002543602
REG00014	SCML1	GSE90639	1.82E+00	.	.	.
REG00014	NT5DC2	GSE109604	1.822554452	knock down	up-regulate	0.000153828
REG00014	FOSB	GSE109604	1.829480094	knock down	up-regulate	0.000800755
REG00014	RPL27A	GSE90639	1.84E+00	.	.	.
REG00014	SNRPB	GSE90639	1.84E+00	.	.	.
REG00014	ATP11C	GSE90639	1.84E+00	.	.	.
REG00014	HSD11B1L	GSE109604	1.842461503	knock down	up-regulate	0.009989548
REG00014	DAD1	GSE90639	1.85E+00	.	.	.
REG00014	ZFPM1	GSE109604	1.851145936	knock down	up-regulate	0.005926608
REG00014	H2BC13	GSE109604	1.857007624	knock down	up-regulate	0.000161415
REG00014	SFPQ	GSE90639	1.86E+00	.	.	.
REG00014	PPIH	GSE90639	1.86E+00	.	.	.
REG00014	TMEM141	GSE90639	1.86E+00	.	.	.
REG00014	NUP107	GSE90639	1.86E+00	.	.	.
REG00014	SOCS3	GSE109604	1.864898024	knock down	up-regulate	0.032287703
REG00014	EP400	GSE109604	1.866606995	knock down	up-regulate	0.005348487

REG00014	STUB1	GSE90639	1.87E+00	.	.	.
REG00014	MRPL47	GSE90639	1.87E+00	.	.	.
REG00014	CPE	GSE90639	1.87E+00	.	.	.
REG00014	ARHGAP42	GSE90639	1.87E+00	.	.	.
REG00014	TEPP	GSE109604	1.876785336	knock down	up-regulate	0.006268115
REG00014	RPS3A	GSE90639	1.88E+00	.	.	.
REG00014	TPX2	GSE90639	1.88E+00	.	.	.
REG00014	DKK1	GSE109604	1.88384898	knock down	up-regulate	7.27E-21
REG00014	EXOC3L4	GSE109604	1.889552894	knock down	up-regulate	0.028166723
REG00014	RPL24	GSE90639	1.89E+00	.	.	.
REG00014	SACS	GSE90639	1.89E+00	.	.	.
REG00014	NFIL3	GSE109604	1.890854585	knock down	up-regulate	5.86E-06
REG00014	MXD3	GSE109604	1.894538535	knock down	up-regulate	0.000501373
REG00014	TUBB	GSE90639	1.90E+00	.	.	.
REG00014	H2AFX	GSE90639	1.90E+00	.	.	.
REG00014	DTL	GSE90639	1.91E+00	.	.	.
REG00014	ADAM11	GSE109604	1.91018896	knock down	up-regulate	0.001095024
REG00014	PCNP	GSE90639	1.92E+00	.	.	.
REG00014	NKPD1	GSE109604	1.926935801	knock down	up-regulate	4.64E-06
REG00014	DCAKD	GSE109604	1.928350689	knock down	up-regulate	1.32E-15
REG00014	RPL31	GSE90639	1.93E+00	.	.	.
REG00014	CASD1	GSE90639	1.93E+00	.	.	.
REG00014	MCM6	GSE90639	1.93E+00	.	.	.
REG00014	BTN1A1	GSE109604	1.935042521	knock down	up-regulate	1.25E-07
REG00014	RPL30	GSE90639	1.94E+00	.	.	.
REG00014	UBE2T	GSE90639	1.94E+00	.	.	.
REG00014	HIST1H2AG	GSE90639	1.94E+00	.	.	.
REG00014	RPSA	GSE90639	1.94E+00	.	.	.
REG00014	PABPC1	GSE90639	1.94E+00	.	.	.
REG00014	WDR19	GSE109604	1.942280724	knock down	up-regulate	3.39E-34
REG00014	LMO4	GSE109604	1.950327422	knock down	up-regulate	0.013181198
REG00014	RPL23	GSE90639	1.96E+00	.	.	.
REG00014	RPL35	GSE90639	1.96E+00	.	.	.
REG00014	SERBP1	GSE90639	1.96E+00	.	.	.
REG00014	ATG5	GSE90639	1.96E+00	.	.	.
REG00014	FBXW11	GSE109604	1.964821142	knock down	up-regulate	9.07E-07
REG00014	SDAD1	GSE90639	1.97E+00	.	.	.
REG00014	MRFAP1L1	GSE90639	1.97E+00	.	.	.
REG00014	BCLAF1	GSE90639	1.98E+00	.	.	.
REG00014	RPL32	GSE90639	1.99E+00	.	.	.
REG00014	ESD	GSE90639	1.99E+00	.	.	.
REG00014	HMGN1	GSE90639	2.00E+00	.	.	.
REG00014	MAPRE1	GSE90639	2.00E+00	.	.	.
REG00014	RFX3	GSE90639	2.00E+00	.	.	.
REG00014	MTX2	GSE90639	2.01E+00	.	.	.
REG00014	AHI1	GSE109604	2.010137078	knock down	up-regulate	0.000941419
REG00014	INPP5B	GSE109604	2.0167438	knock down	up-regulate	1.64E-05
REG00014	KLK1	GSE109604	2.023343656	knock down	up-regulate	6.03E-17
REG00014	CD2AP	GSE90639	2.03E+00	.	.	.
REG00014	JAK1	GSE109604	2.039534019	knock down	up-regulate	2.24E-06

REG00014	RPS20	GSE90639	2.05E+00	.	.	.
REG00014	RPL23A	GSE90639	2.06E+00	.	.	.
REG00014	SRSF1	GSE90639	2.06E+00	.	.	.
REG00014	NOP58	GSE90639	2.06E+00	.	.	.
REG00014	EEF1A1	GSE90639	2.06E+00	.	.	.
REG00014	RPS4X	GSE90639	2.07E+00	.	.	.
REG00014	UBA52	GSE90639	2.08E+00	.	.	.
REG00014	ACOX1	GSE109604	2.084655308	knock down	up-regulate	0.000698058
REG00014	RPS27	GSE90639	2.09E+00	.	.	.
REG00014	RPS6	GSE90639	2.09E+00	.	.	.
REG00014	SNRPC	GSE90639	2.09E+00	.	.	.
REG00014	SSB	GSE90639	2.09E+00	.	.	.
REG00014	XRN2	GSE90639	2.09E+00	.	.	.
REG00014	NUCKS1	GSE90639	2.09E+00	.	.	.
REG00014	HIST1H2AC	GSE90639	2.09E+00	.	.	.
REG00014	TOP1MT	GSE109604	2.095251865	knock down	up-regulate	5.80E-12
REG00014	TOP1	GSE90639	2.10E+00	.	.	.
REG00014	MRPS9	GSE90639	2.11E+00	.	.	.
REG00014	H1FX	GSE90639	2.11E+00	.	.	.
REG00014	NAA80	GSE109604	2.116423573	knock down	up-regulate	3.13E-05
REG00014	RPL12	GSE90639	2.12E+00	.	.	.
REG00014	RPS17	GSE90639	2.13E+00	.	.	.
REG00014	RPL22L1	GSE90639	2.13E+00	.	.	.
REG00014	IL6ST	GSE90639	2.13E+00	.	.	.
REG00014	EXD3	GSE109604	2.136019652	knock down	up-regulate	0.013066533
REG00014	SLC18B1	GSE90639	2.14E+00	.	.	.
REG00014	KIAA0319L	GSE109604	2.149960884	knock down	up-regulate	4.52E-06
REG00014	RPS21	GSE90639	2.15E+00	.	.	.
REG00014	RPS28	GSE90639	2.15E+00	.	.	.
REG00014	SNRPE	GSE90639	2.15E+00	.	.	.
REG00014	MYBL2	GSE90639	2.15E+00	.	.	.
REG00014	MRPL22	GSE90639	2.16E+00	.	.	.
REG00014	HNRNPK	GSE90639	2.16E+00	.	.	.
REG00014	TRAPPC14	GSE109604	2.163153808	knock down	up-regulate	0.005766234
REG00014	CCT7	GSE90639	2.17E+00	.	.	.
REG00014	GOLT1B	GSE90639	2.17E+00	.	.	.
REG00014	NDUFB11	GSE90639	2.17E+00	.	.	.
REG00014	HNRNPA1L2	GSE109604	2.170444613	knock down	up-regulate	0.030555775
REG00014	STYXL1	GSE90639	2.18E+00	.	.	.
REG00014	GAPDH	GSE90639	2.19E+00	.	.	.
REG00014	HNRNPDL	GSE90639	2.19E+00	.	.	.
REG00014	TRABD	GSE109604	2.19115356	knock down	up-regulate	5.06E-05
REG00014	LRRC29	GSE109604	2.197783792	knock down	up-regulate	0.008554932
REG00014	NONO	GSE90639	2.20E+00	.	.	.
REG00014	ARVCF	GSE109604	2.204553131	knock down	up-regulate	0.027139713
REG00014	RPS3	GSE90639	2.21E+00	.	.	.
REG00014	DUT	GSE90639	2.21E+00	.	.	.
REG00014	RANBP2	GSE90639	2.21E+00	.	.	.
REG00014	CYP27B1	GSE109604	2.214193558	knock down	up-regulate	6.27E-05
REG00014	RPS19	GSE90639	2.22E+00	.	.	.

REG00014	YTHDC1	GSE109604	2.220285147	knock down	up-regulate	5.79E-08
REG00014	ENO1	GSE90639	2.23E+00	.	.	.
REG00014	H2BFS	GSE90639	2.23E+00	.	.	.
REG00014	ATE1	GSE90639	2.23E+00	.	.	.
REG00014	NPM1	GSE90639	2.23E+00	.	.	.
REG00014	HES4	GSE90639	2.24E+00	.	.	.
REG00014	RPS11	GSE90639	2.25E+00	.	.	.
REG00014	NAT8L	GSE109604	2.255443936	knock down	up-regulate	0.000112925
REG00014	RPL27	GSE90639	2.26E+00	.	.	.
REG00014	PCNA	GSE90639	2.26E+00	.	.	.
REG00014	SH2D6	GSE109604	2.265848591	knock down	up-regulate	4.15E-05
REG00014	RPL10	GSE90639	2.27E+00	.	.	.
REG00014	RPL7	GSE90639	2.27E+00	.	.	.
REG00014	RPLP1	GSE90639	2.27E+00	.	.	.
REG00014	COX7A2	GSE90639	2.27E+00	.	.	.
REG00014	ZYX	GSE109604	2.270450169	knock down	up-regulate	0.005786486
REG00014	DCLRE1A	GSE109604	2.279281339	knock down	up-regulate	2.52E-11
REG00014	RPL36AL	GSE90639	2.28E+00	.	.	.
REG00014	RPS7	GSE90639	2.28E+00	.	.	.
REG00014	WLS	GSE109604	2.291346286	knock down	up-regulate	1.90E-30
REG00014	WDR70	GSE109604	2.297881295	knock down	up-regulate	1.23E-06
REG00014	RPL39	GSE90639	2.30E+00	.	.	.
REG00014	RAD51AP1	GSE90639	2.30E+00	.	.	.
REG00014	CCNB1	GSE90639	2.31E+00	.	.	.
REG00014	HAGHL	GSE109604	2.31389199	knock down	up-regulate	0.027143278
REG00014	TOP2A	GSE90639	2.33E+00	.	.	.
REG00014	AGTPBP1	GSE90639	2.33E+00	.	.	.
REG00014	NBN	GSE90639	2.33E+00	.	.	.
REG00014	TRPS1	GSE109604	2.343144199	knock down	up-regulate	0.000292628
REG00014	RPL18	GSE90639	2.35E+00	.	.	.
REG00014	SSRP1	GSE90639	2.35E+00	.	.	.
REG00014	CDK1	GSE90639	2.36E+00	.	.	.
REG00014	NCL	GSE90639	2.36E+00	.	.	.
REG00014	TTC37	GSE90639	2.37E+00	.	.	.
REG00014	ATP5B	GSE90639	2.37E+00	.	.	.
REG00014	DEK	GSE90639	2.38E+00	.	.	.
REG00014	MRPS26	GSE90639	2.40E+00	.	.	.
REG00014	EIF4EBP1	GSE90639	2.41E+00	.	.	.
REG00014	MEGF8	GSE109604	2.419522586	knock down	up-regulate	0.000499123
REG00014	RPL19	GSE90639	2.42E+00	.	.	.
REG00014	FAU	GSE90639	2.42E+00	.	.	.
REG00014	KPNA1	GSE109604	2.429340907	knock down	up-regulate	5.59E-05
REG00014	RPL5	GSE90639	2.43E+00	.	.	.
REG00014	DARS2	GSE90639	2.43E+00	.	.	.
REG00014	HIST1H3F	GSE90639	2.43E+00	.	.	.
REG00014	PARD3	GSE109604	2.436804767	knock down	up-regulate	0.029694477
REG00014	RPS16	GSE90639	2.45E+00	.	.	.
REG00014	CD70	GSE109604	2.45487341	knock down	up-regulate	0.000125926
REG00014	TP53I13	GSE109604	2.459658665	knock down	up-regulate	0.00016128
REG00014	RPL7A	GSE90639	2.46E+00	.	.	.

REG00014	RPS10	GSE90639	2.46E+00	.	.	.
REG00014	AIMP1	GSE90639	2.46E+00	.	.	.
REG00014	SPN	GSE109604	2.465696376	knock down	up-regulate	0.00042247
REG00014	HIST2H2AA3	GSE90639	2.48E+00	.	.	.
REG00014	BOD1	GSE109604	2.480894729	knock down	up-regulate	2.50E-36
REG00014	TIGD1	GSE109604	2.489940154	knock down	up-regulate	0.000130192
REG00014	RPL4	GSE90639	2.49E+00	.	.	.
REG00014	ACTG1	GSE90639	2.49E+00	.	.	.
REG00014	HNRNPA1	GSE90639	2.49E+00	.	.	.
REG00014	COPG2	GSE90639	2.51E+00	.	.	.
REG00014	TRIM6	GSE109604	2.518811526	knock down	up-regulate	0.000270183
REG00014	HIST2H4B	GSE90639	2.52E+00	.	.	.
REG00014	PPT1	GSE90639	2.54E+00	.	.	.
REG00014	ATF3	GSE109604	2.541472016	knock down	up-regulate	4.93E-05
REG00014	RPL10A	GSE90639	2.55E+00	.	.	.
REG00014	HEATR5B	GSE109604	2.561037198	knock down	up-regulate	0.011655421
REG00014	RPS18	GSE90639	2.57E+00	.	.	.
REG00014	CDKN2AIP	GSE90639	2.57E+00	.	.	.
REG00014	EPS8	GSE109604	2.583813681	knock down	up-regulate	0.005829074
REG00014	MAP1S	GSE109604	2.592036295	knock down	up-regulate	0.00118948
REG00014	CKB	GSE90639	2.60E+00	.	.	.
REG00014	HIST1H2AM	GSE90639	2.60E+00	.	.	.
REG00014	ZNF385D	GSE109604	2.618662645	knock down	up-regulate	0.000807621
REG00014	HSP90AB1	GSE90639	2.63E+00	.	.	.
REG00014	FAM111A	GSE109604	2.631642854	knock down	up-regulate	0.012933085
REG00014	CA2	GSE90639	2.64E+00	.	.	.
REG00014	HIST1H2AL	GSE90639	2.65E+00	.	.	.
REG00014	HACL1	GSE109604	2.650582965	knock down	up-regulate	9.84E-06
REG00014	UVRAG	GSE109604	2.65796449	knock down	up-regulate	0.003683496
REG00014	ATP9A	GSE109604	2.662028251	knock down	up-regulate	4.01E-18
REG00014	VBP1	GSE90639	2.67E+00	.	.	.
REG00014	RPL26	GSE90639	2.68E+00	.	.	.
REG00014	HNRNPR	GSE90639	2.68E+00	.	.	.
REG00014	LRRN4CL	GSE109604	2.683872996	knock down	up-regulate	8.75E-05
REG00014	H2AFZ	GSE90639	2.69E+00	.	.	.
REG00014	RPL37A	GSE90639	2.71E+00	.	.	.
REG00014	RPL8	GSE90639	2.73E+00	.	.	.
REG00014	KIF5B	GSE90639	2.74E+00	.	.	.
REG00014	EIF4E1B	GSE109604	2.74445683	knock down	up-regulate	0.000525229
REG00014	ZCCHC2	GSE109604	2.748463719	knock down	up-regulate	0.001620199
REG00014	VAMP4	GSE109604	2.762082343	knock down	up-regulate	3.35E-05
REG00014	RPL22	GSE90639	2.77E+00	.	.	.
REG00014	RPL29	GSE90639	2.77E+00	.	.	.
REG00014	WDR55	GSE109604	2.77577276	knock down	up-regulate	0.027155934
REG00014	EEF1G	GSE90639	2.82E+00	.	.	.
REG00014	ADAMTSL1	GSE109604	2.85483478	knock down	up-regulate	6.16E-05
REG00014	ADPRM	GSE109604	2.890288422	knock down	up-regulate	0.011709885
REG00014	ALKBH4	GSE109604	2.893278971	knock down	up-regulate	1.32E-07
REG00014	TMTC2	GSE109604	2.899243217	knock down	up-regulate	0.000243495
REG00014	RPS15	GSE90639	2.90E+00	.	.	.

REG00014	HCCS	GSE90639	2.90E+00	.	.	.
REG00014	QPCT	GSE109604	2.905852976	knock down	up-regulate	0.047503983
REG00014	ZNF792	GSE109604	2.90977988	knock down	up-regulate	0.036483561
REG00014	EEF2	GSE90639	2.93E+00	.	.	.
REG00014	ATF4	GSE90639	2.93E+00	.	.	.
REG00014	IRS4	GSE90639	2.97E+00	.	.	.
REG00014	TTLL7	GSE109604	2.984091955	knock down	up-regulate	1.76E-06
REG00014	NDUFS5	GSE90639	2.99E+00	.	.	.
REG00014	HIST1H1D	GSE90639	3.04E+00	.	.	.
REG00014	HIST1H4H	GSE90639	3.09E+00	.	.	.
REG00014	TRIM2	GSE109604	3.109905358	knock down	up-regulate	0.000121787
REG00014	OSBPL5	GSE109604	3.159566862	knock down	up-regulate	0.000993331
REG00014	HNRNPAB	GSE90639	3.16E+00	.	.	.
REG00014	CCT4	GSE90639	3.17E+00	.	.	.
REG00014	TRAK2	GSE109604	3.182034488	knock down	up-regulate	9.51E-06
REG00014	ZNF862	GSE109604	3.206592944	knock down	up-regulate	0.012274605
REG00014	LDHB	GSE90639	3.21E+00	.	.	.
REG00014	TMEM65	GSE109604	3.228033881	knock down	up-regulate	0.000324908
REG00014	CEP72	GSE109604	3.234492992	knock down	up-regulate	0.048409412
REG00014	RPL6	GSE90639	3.25E+00	.	.	.
REG00014	ATP10D	GSE109604	3.290601623	knock down	up-regulate	4.67E-22
REG00014	TUBA1B	GSE90639	3.30E+00	.	.	.
REG00014	LAPTM4A	GSE109604	3.304552685	knock down	up-regulate	5.38E-08
REG00014	HIST2H3C	GSE90639	3.32E+00	.	.	.
REG00014	HIST2H3A	GSE90639	3.32E+00	.	.	.
REG00014	ACY1	GSE109604	3.326591228	knock down	up-regulate	0.031818436
REG00014	MRPS34	GSE90639	3.33E+00	.	.	.
REG00014	TMEM222	GSE109604	3.338373269	knock down	up-regulate	0.000462217
REG00014	STRAP	GSE90639	3.38E+00	.	.	.
REG00014	TRIM68	GSE109604	3.380159094	knock down	up-regulate	2.98E-06
REG00014	UBB	GSE90639	3.39E+00	.	.	.
REG00014	UBE2Q1	GSE109604	3.395113447	knock down	up-regulate	5.91E-06
REG00014	CD200	GSE109604	3.400084909	knock down	up-regulate	0.001244598
REG00014	UNKL	GSE109604	3.405959751	knock down	up-regulate	0.000857912
REG00014	PSAT1	GSE90639	3.41E+00	.	.	.
REG00014	RPS2	GSE90639	3.42E+00	.	.	.
REG00014	XRCC6	GSE90639	3.44E+00	.	.	.
REG00014	CD59	GSE109604	3.467579887	knock down	up-regulate	0.017019534
REG00014	TGFB2	GSE109604	3.493634905	knock down	up-regulate	9.76E-06
REG00014	PSPC1	GSE109604	3.541059453	knock down	up-regulate	1.79E-05
REG00014	TMEM39A	GSE109604	3.541584016	knock down	up-regulate	0.000348278
REG00014	GINS3	GSE109604	3.574060341	knock down	up-regulate	6.62E-43
REG00014	HIST1H4B	GSE90639	3.68E+00	.	.	.
REG00014	TMEM203	GSE109604	3.68151341	knock down	up-regulate	1.06E-05
REG00014	HIST1H2BC	GSE90639	3.76E+00	.	.	.
REG00014	NDUFB4	GSE90639	3.76E+00	.	.	.
REG00014	HIST2H3D	GSE90639	3.83E+00	.	.	.
REG00014	HMGB3	GSE90639	3.86E+00	.	.	.
REG00014	ZFP41	GSE109604	3.863463663	knock down	up-regulate	1.04E-15
REG00014	HIST1H3H	GSE90639	3.90E+00	.	.	.

REG00014	HIST1H4E	GSE90639	3.90E+00	.	.	.
REG00014	HIST2H2AC	GSE90639	3.95E+00	.	.	.
REG00014	AMMECR1	GSE109604	3.989907646	knock down	up-regulate	0.005152202
REG00014	TRIM37	GSE109604	4.007090437	knock down	up-regulate	0.000117263
REG00014	UQCRH	GSE109604	4.01070252	knock down	up-regulate	0.000150641
REG00014	HIST1H2BJ	GSE90639	4.08E+00	.	.	.
REG00014	FAM126A	GSE109604	4.096500123	knock down	up-regulate	0.043541763
REG00014	MIF	GSE90639	4.11E+00	.	.	.
REG00014	HNRNPA2B1	GSE90639	4.15E+00	.	.	.
REG00014	FAM111B	GSE109604	4.254718055	knock down	up-regulate	0.032802446
REG00014	HIST1H2BO	GSE90639	4.33E+00	.	.	.
REG00014	FJX1	GSE109604	4.396751791	knock down	up-regulate	0.000836766
REG00014	HIST1H4D	GSE90639	4.42E+00	.	.	.
REG00014	APLN	GSE109604	4.513603658	knock down	up-regulate	0.002642123
REG00014	IDUA	GSE109604	4.51539441	knock down	up-regulate	0.026584485
REG00014	HIST1H2AH	GSE90639	4.57E+00	.	.	.
REG00014	EXOC3	GSE90639	4.59E+00	.	.	.
REG00014	ZNF865	GSE109604	4.628260668	knock down	up-regulate	0.047090884
REG00014	FZR1	GSE109604	4.639780834	knock down	up-regulate	2.91E-07
REG00014	HIST1H4J	GSE90639	4.66E+00	.	.	.
REG00014	HIST1H2AI	GSE90639	4.67E+00	.	.	.
REG00014	AURKA	GSE109604	4.678448133	knock down	up-regulate	0.003430597
REG00014	HIST1H2BD	GSE90639	4.76E+00	.	.	.
REG00014	HIST2H4A	GSE90639	4.82E+00	.	.	.
REG00014	ABTB1	GSE109604	4.88402028	knock down	up-regulate	0.023458458
REG00014	UCKL1	GSE109604	4.93161684	knock down	up-regulate	0.000986105
REG00014	NCAPG	GSE90639	5.09E+00	.	.	.
REG00014	HIST2H2BE	GSE90639	5.09E+00	.	.	.
REG00014	GAA	GSE109604	5.143311187	knock down	up-regulate	0.003414005
REG00014	ANKRD28	GSE109604	5.361094624	knock down	up-regulate	0.003229527
REG00014	HIST1H2BK	GSE90639	5.59E+00	.	.	.
REG00014	WDR24	GSE109604	5.594097717	knock down	up-regulate	5.73E-59
REG00014	ZMAT2	GSE109604	5.619612042	knock down	up-regulate	1.50E-18
REG00014	MANF	GSE109604	6.147743511	knock down	up-regulate	0.005859822
REG00014	HIST1H3B	GSE90639	6.47E+00	.	.	.
REG00014	GPR153	GSE109604	6.511519454	knock down	up-regulate	9.87E-05
REG00014	AFTPH	GSE109604	6.903746022	knock down	up-regulate	0.000897303
REG00014	HIST1H1E	GSE90639	7.74E+00	.	.	.
REG00014	LRP6	GSE109604	9.897537073	knock down	up-regulate	2.25E-11
REG00014	RHBDL1	GSE92220	.	.	.	.
REG00014	BCL6	GSE92220	.	.	.	.
REG00014	RHEB	GSE92220	.	.	.	.
REG00014	RING1	GSE92220	.	.	.	.
REG00014	RIPK1	GSE92220	.	.	.	.
REG00014	RIPK2	GSE92220	.	.	.	.
REG00014	RIT1	GSE92220	.	.	.	.
REG00014	RLBP1	GSE92220	.	.	.	.
REG00014	BCL7A	GSE92220	.	.	.	.
REG00014	RNASE4	GSE92220	.	.	.	.
REG00014	RNF10	GSE92220	.	.	.	.

REG00014	RNF11	GSE92220	.	.	.	.
REG00014	RNF13	GSE92220	.	.	.	.
REG00014	TRIM3	GSE92220	.	.	.	.
REG00014	TRIM39	GSE92220	.	.	.	.
REG00014	PCGF3	GSE92220	.	.	.	.
REG00014	RNF4	GSE92220	.	.	.	.
REG00014	RNF7	GSE92220	.	.	.	.
REG00014	RNGTT	GSE92220	.	.	.	.
REG00014	RNH1	GSE92220	.	.	.	.
REG00014	RNMT	GSE92220	.	.	.	.
REG00014	RNPEPL1	GSE92220	.	.	.	.
REG00014	RNPS1	GSE92220	.	.	.	.
REG00014	ASIC3	GSE92220	.	.	.	.
REG00014	OPN1SW	GSE92220	.	.	.	.
REG00014	BCR	GSE92220	.	.	.	.
REG00014	BCS1L	GSE92220	.	.	.	.
REG00014	ROBO1	GSE92220	.	.	.	.
REG00014	ROCK1	GSE92220	.	.	.	.
REG00014	ROCK2	GSE92220	.	.	.	.
REG00014	PTBP3	GSE92220	.	.	.	.
REG00014	ROM1	GSE92220	.	.	.	.
REG00014	ROR2	GSE92220	.	.	.	.
REG00014	RORA	GSE92220	.	.	.	.
REG00014	BDH1	GSE92220	.	.	.	.
REG00014	RPA1	GSE92220	.	.	.	.
REG00014	BDKRB1	GSE92220	.	.	.	.
REG00014	RPE	GSE92220	.	.	.	.
REG00014	RPIA	GSE92220	.	.	.	.
REG00014	RPL10	GSE92220	.	.	.	.
REG00014	RPL10A	GSE92220	.	.	.	.
REG00014	CNNM2	GSE92220	.	.	.	.
REG00014	RPL11	GSE92220	.	.	.	.
REG00014	RPL12	GSE92220	.	.	.	.
REG00014	RPL13	GSE92220	.	.	.	.
REG00014	RPL13A	GSE92220	.	.	.	.
REG00014	RPL14	GSE92220	.	.	.	.
REG00014	RPL15	GSE92220	.	.	.	.
REG00014	RPL18A	GSE92220	.	.	.	.
REG00014	RPL19	GSE92220	.	.	.	.
REG00014	RPL21	GSE92220	.	.	.	.
REG00014	RPL22	GSE92220	.	.	.	.
REG00014	RPL23	GSE92220	.	.	.	.
REG00014	RPL23A	GSE92220	.	.	.	.
REG00014	RPL24	GSE92220	.	.	.	.
REG00014	RPL26	GSE92220	.	.	.	.
REG00014	RPL27	GSE92220	.	.	.	.
REG00014	RPL27A	GSE92220	.	.	.	.
REG00014	RPL28	GSE92220	.	.	.	.
REG00014	RPL29	GSE92220	.	.	.	.
REG00014	RPL30	GSE92220	.	.	.	.

REG00014	RPL31	GSE92220	.	.	.	.
REG00014	RPL32	GSE92220	.	.	.	.
REG00014	BECN1	GSE92220	.	.	.	.
REG00014	RPL34	GSE92220	.	.	.	.
REG00014	RPL35	GSE92220	.	.	.	.
REG00014	RPL35A	GSE92220	.	.	.	.
REG00014	RPL36AL	GSE92220	.	.	.	.
REG00014	RPL37	GSE92220	.	.	.	.
REG00014	RPL37A	GSE92220	.	.	.	.
REG00014	RPL38	GSE92220	.	.	.	.
REG00014	RPL39	GSE92220	.	.	.	.
REG00014	RPL41	GSE92220	.	.	.	.
REG00014	RPL36A	GSE92220	.	.	.	.
REG00014	RPL5	GSE92220	.	.	.	.
REG00014	RPL6	GSE92220	.	.	.	.
REG00014	RPL7	GSE92220	.	.	.	.
REG00014	RPL7A	GSE92220	.	.	.	.
REG00014	RPL9	GSE92220	.	.	.	.
REG00014	CFB	GSE92220	.	.	.	.
REG00014	RPLP0	GSE92220	.	.	.	.
REG00014	RPLP1	GSE92220	.	.	.	.
REG00014	RPLP2	GSE92220	.	.	.	.
REG00014	MRPL12	GSE92220	.	.	.	.
REG00014	MRPL3	GSE92220	.	.	.	.
REG00014	MRPS12	GSE92220	.	.	.	.
REG00014	RPN1	GSE92220	.	.	.	.
REG00014	RPS10	GSE92220	.	.	.	.
REG00014	RPS11	GSE92220	.	.	.	.
REG00014	RPS12	GSE92220	.	.	.	.
REG00014	RPS13	GSE92220	.	.	.	.
REG00014	RPS14	GSE92220	.	.	.	.
REG00014	RPS15	GSE92220	.	.	.	.
REG00014	RPS15A	GSE92220	.	.	.	.
REG00014	RPS16	GSE92220	.	.	.	.
REG00014	RPS17	GSE92220	.	.	.	.
REG00014	CNNM3	GSE92220	.	.	.	.
REG00014	RPS18	GSE92220	.	.	.	.
REG00014	RPS19	GSE92220	.	.	.	.
REG00014	RPS2	GSE92220	.	.	.	.
REG00014	RPS20	GSE92220	.	.	.	.
REG00014	RPS21	GSE92220	.	.	.	.
REG00014	RPS23	GSE92220	.	.	.	.
REG00014	RPS24	GSE92220	.	.	.	.
REG00014	RPS25	GSE92220	.	.	.	.
REG00014	RPS26	GSE92220	.	.	.	.
REG00014	RPS27	GSE92220	.	.	.	.
REG00014	RPS27A	GSE92220	.	.	.	.
REG00014	RPS28	GSE92220	.	.	.	.
REG00014	RPS29	GSE92220	.	.	.	.
REG00014	RPS3	GSE92220	.	.	.	.

REG00014	RPS3A	GSE92220	.	.	.	.
REG00014	RPS4X	GSE92220	.	.	.	.
REG00014	RPS4Y1	GSE92220	.	.	.	.
REG00014	RPS5	GSE92220	.	.	.	.
REG00014	RPS6	GSE92220	.	.	.	.
REG00014	BGLAP	GSE92220	.	.	.	.
REG00014	RPS6KA3	GSE92220	.	.	.	.
REG00014	RPS6KB1	GSE92220	.	.	.	.
REG00014	RPS6KB2	GSE92220	.	.	.	.
REG00014	RPS6KC1	GSE92220	.	.	.	.
REG00014	RPS7	GSE92220	.	.	.	.
REG00014	RPS8	GSE92220	.	.	.	.
REG00014	RPS9	GSE92220	.	.	.	.
REG00014	CNOT9	GSE92220	.	.	.	.
REG00014	RRBP1	GSE92220	.	.	.	.
REG00014	RREB1	GSE92220	.	.	.	.
REG00014	RRM1	GSE92220	.	.	.	.
REG00014	C2orf48	GSE92220	.	.	.	.
REG00014	RSC1A1	GSE92220	.	.	.	.
REG00014	CLIP1	GSE92220	.	.	.	.
REG00014	RSU1	GSE92220	.	.	.	.
REG00014	RTKN	GSE92220	.	.	.	.
REG00014	RTN3	GSE92220	.	.	.	.
REG00014	RUNX1	GSE92220	.	.	.	.
REG00014	RUVBL1	GSE92220	.	.	.	.
REG00014	RXRA	GSE92220	.	.	.	.
REG00014	RXRB	GSE92220	.	.	.	.
REG00014	BHMT2	GSE92220	.	.	.	.
REG00014	RYBP	GSE92220	.	.	.	.
REG00014	RYK	GSE92220	.	.	.	.
REG00014	S100A1	GSE92220	.	.	.	.
REG00014	S100A11	GSE92220	.	.	.	.
REG00014	S100A13	GSE92220	.	.	.	.
REG00014	S100A6	GSE92220	.	.	.	.
REG00014	BID	GSE92220	.	.	.	.
REG00014	S100B	GSE92220	.	.	.	.
REG00014	S100P	GSE92220	.	.	.	.
REG00014	SAA4	GSE92220	.	.	.	.
REG00014	VPS52	GSE92220	.	.	.	.
REG00014	SACS	GSE92220	.	.	.	.
REG00014	SAFB	GSE92220	.	.	.	.
REG00014	SALL1	GSE92220	.	.	.	.
REG00014	SAP18	GSE92220	.	.	.	.
REG00014	SAR1A	GSE92220	.	.	.	.
REG00014	SAR1B	GSE92220	.	.	.	.
REG00014	SARDH	GSE92220	.	.	.	.
REG00014	SARS	GSE92220	.	.	.	.
REG00014	SART1	GSE92220	.	.	.	.
REG00014	SAT1	GSE92220	.	.	.	.
REG00014	SBF1	GSE92220	.	.	.	.

REG00014	MSMO1	GSE92220	.	.	.	.
REG00014	SC5D	GSE92220	.	.	.	.
REG00014	ATXN1	GSE92220	.	.	.	.
REG00014	ATXN10	GSE92220	.	.	.	.
REG00014	BLCAP	GSE92220	.	.	.	.
REG00014	ATXN2	GSE92220	.	.	.	.
REG00014	ATXN7	GSE92220	.	.	.	.
REG00014	SCAMP2	GSE92220	.	.	.	.
REG00014	SCAMP3	GSE92220	.	.	.	.
REG00014	SCAND1	GSE92220	.	.	.	.
REG00014	SCD	GSE92220	.	.	.	.
REG00014	SCML1	GSE92220	.	.	.	.
REG00014	SCN1A	GSE92220	.	.	.	.
REG00014	SCN1B	GSE92220	.	.	.	.
REG00014	BLMH	GSE92220	.	.	.	.
REG00014	SCN9A	GSE92220	.	.	.	.
REG00014	SCNN1A	GSE92220	.	.	.	.
REG00014	CXCR5	GSE92220	.	.	.	.
REG00014	SCNN1D	GSE92220	.	.	.	.
REG00014	SCO1	GSE92220	.	.	.	.
REG00014	SCO2	GSE92220	.	.	.	.
REG00014	CCL14	GSE92220	.	.	.	.
REG00014	CCL15	GSE92220	.	.	.	.
REG00014	CCL16	GSE92220	.	.	.	.
REG00014	CCL20	GSE92220	.	.	.	.
REG00014	CX3CL1	GSE92220	.	.	.	.
REG00014	AIMP1	GSE92220	.	.	.	.
REG00014	SDC1	GSE92220	.	.	.	.
REG00014	SDC2	GSE92220	.	.	.	.
REG00014	BMI1	GSE92220	.	.	.	.
REG00014	SDC3	GSE92220	.	.	.	.
REG00014	SDCBP	GSE92220	.	.	.	.
REG00014	NEMF	GSE92220	.	.	.	.
REG00014	CWC27	GSE92220	.	.	.	.
REG00014	UTP14A	GSE92220	.	.	.	.
REG00014	SDCCAG3	GSE92220	.	.	.	.
REG00014	NOXA1	GSE92220	.	.	.	.
REG00014	TSHZ1	GSE92220	.	.	.	.
REG00014	BMP1	GSE92220	.	.	.	.
REG00014	SDF2	GSE92220	.	.	.	.
REG00014	SDF2L1	GSE92220	.	.	.	.
REG00014	SDHA	GSE92220	.	.	.	.
REG00014	SDHD	GSE92220	.	.	.	.
REG00014	SDPR	GSE92220	.	.	.	.
REG00014	SDS	GSE92220	.	.	.	.
REG00014	EXOC5	GSE92220	.	.	.	.
REG00014	SEC13	GSE92220	.	.	.	.
REG00014	SEC14L1	GSE92220	.	.	.	.
REG00014	SEC22B	GSE92220	.	.	.	.
REG00014	SEC23A	GSE92220	.	.	.	.

REG00014	SEC23B	GSE92220	.	.	.	.
REG00014	SEC24A	GSE92220	.	.	.	.
REG00014	SEC24C	GSE92220	.	.	.	.
REG00014	SEC24D	GSE92220	.	.	.	.
REG00014	BMP4	GSE92220	.	.	.	.
REG00014	SEL1L	GSE92220	.	.	.	.
REG00014	SELENBP1	GSE92220	.	.	.	.
REG00014	SEMA3B	GSE92220	.	.	.	.
REG00014	SEMA3F	GSE92220	.	.	.	.
REG00014	SEMA4A	GSE92220	.	.	.	.
REG00014	SEMA4C	GSE92220	.	.	.	.
REG00014	SEMA4F	GSE92220	.	.	.	.
REG00014	SEMA4G	GSE92220	.	.	.	.
REG00014	SEMA6A	GSE92220	.	.	.	.
REG00014	SEMA6B	GSE92220	.	.	.	.
REG00014	SEMA6C	GSE92220	.	.	.	.
REG00014	SEMA7A	GSE92220	.	.	.	.
REG00014	SELENOP	GSE92220	.	.	.	.
REG00014	SERF2	GSE92220	.	.	.	.
REG00014	SERP1	GSE92220	.	.	.	.
REG00014	BMPR1A	GSE92220	.	.	.	.
REG00014	SET	GSE92220	.	.	.	.
REG00014	SETDB1	GSE92220	.	.	.	.
REG00014	SF3A1	GSE92220	.	.	.	.
REG00014	SF3A3	GSE92220	.	.	.	.
REG00014	SF3B1	GSE92220	.	.	.	.
REG00014	SF3B2	GSE92220	.	.	.	.
REG00014	SF3B3	GSE92220	.	.	.	.
REG00014	SF3B4	GSE92220	.	.	.	.
REG00014	SFN	GSE92220	.	.	.	.
REG00014	SFPQ	GSE92220	.	.	.	.
REG00014	SFRP5	GSE92220	.	.	.	.
REG00014	BMPR2	GSE92220	.	.	.	.
REG00014	SRSF1	GSE92220	.	.	.	.
REG00014	TRA2B	GSE92220	.	.	.	.
REG00014	SRSF11	GSE92220	.	.	.	.
REG00014	SCAF11	GSE92220	.	.	.	.
REG00014	SRSF3	GSE92220	.	.	.	.
REG00014	SRSF4	GSE92220	.	.	.	.
REG00014	SRSF5	GSE92220	.	.	.	.
REG00014	SRSF6	GSE92220	.	.	.	.
REG00014	SRSF7	GSE92220	.	.	.	.
REG00014	SFSWAP	GSE92220	.	.	.	.
REG00014	SRSF9	GSE92220	.	.	.	.
REG00014	POLR3D	GSE92220	.	.	.	.
REG00014	SFTPB	GSE92220	.	.	.	.
REG00014	SGCE	GSE92220	.	.	.	.
REG00014	SGK1	GSE92220	.	.	.	.
REG00014	SGSH	GSE92220	.	.	.	.
REG00014	SGTA	GSE92220	.	.	.	.

REG00014	SH3BP1	GSE92220	.	.	.	.
REG00014	SH3BP2	GSE92220	.	.	.	.
REG00014	SH3BP4	GSE92220	.	.	.	.
REG00014	SH3GL1	GSE92220	.	.	.	.
REG00014	SH3GLB1	GSE92220	.	.	.	.
REG00014	SH3GLB2	GSE92220	.	.	.	.
REG00014	SHB	GSE92220	.	.	.	.
REG00014	SHBG	GSE92220	.	.	.	.
REG00014	BNIP3	GSE92220	.	.	.	.
REG00014	SHC1	GSE92220	.	.	.	.
REG00014	SEM1	GSE92220	.	.	.	.
REG00014	FBXW4	GSE92220	.	.	.	.
REG00014	SHH	GSE92220	.	.	.	.
REG00014	BNIP3L	GSE92220	.	.	.	.
REG00014	SHMT2	GSE92220	.	.	.	.
REG00014	SIAH1	GSE92220	.	.	.	.
REG00014	SIAH2	GSE92220	.	.	.	.
REG00014	ST6GAL1	GSE92220	.	.	.	.
REG00014	ST3GAL1	GSE92220	.	.	.	.
REG00014	ST3GAL2	GSE92220	.	.	.	.
REG00014	ST3GAL4	GSE92220	.	.	.	.
REG00014	STIL	GSE92220	.	.	.	.
REG00014	SIM1	GSE92220	.	.	.	.
REG00014	SIM2	GSE92220	.	.	.	.
REG00014	SIX5	GSE92220	.	.	.	.
REG00014	PRMT5	GSE92220	.	.	.	.
REG00014	SKI	GSE92220	.	.	.	.
REG00014	SKP1	GSE92220	.	.	.	.
REG00014	DST	GSE92220	.	.	.	.
REG00014	SKP2	GSE92220	.	.	.	.
REG00014	SLBP	GSE92220	.	.	.	.
REG00014	SLC11A2	GSE92220	.	.	.	.
REG00014	SLC40A1	GSE92220	.	.	.	.
REG00014	SLC12A2	GSE92220	.	.	.	.
REG00014	SLC12A4	GSE92220	.	.	.	.
REG00014	SLC12A7	GSE92220	.	.	.	.
REG00014	SLC16A3	GSE92220	.	.	.	.
REG00014	SLC17A2	GSE92220	.	.	.	.
REG00014	SLC17A5	GSE92220	.	.	.	.
REG00014	SLC18A2	GSE92220	.	.	.	.
REG00014	SLC19A1	GSE92220	.	.	.	.
REG00014	SLC19A2	GSE92220	.	.	.	.
REG00014	BPHL	GSE92220	.	.	.	.
REG00014	SLC1A4	GSE92220	.	.	.	.
REG00014	SLC1A5	GSE92220	.	.	.	.
REG00014	SLC20A1	GSE92220	.	.	.	.
REG00014	SLC20A2	GSE92220	.	.	.	.
REG00014	SLCO4A1	GSE92220	.	.	.	.
REG00014	SLCO2B1	GSE92220	.	.	.	.
REG00014	SLC22A3	GSE92220	.	.	.	.

REG00014	BRAF	GSE92220	.	.	.	.
REG00014	SLC23A2	GSE92220	.	.	.	.
REG00014	SLC23A1	GSE92220	.	.	.	.
REG00014	SLC24A1	GSE92220	.	.	.	.
REG00014	SLC25A1	GSE92220	.	.	.	.
REG00014	SLC25A10	GSE92220	.	.	.	.
REG00014	SLC25A11	GSE92220	.	.	.	.
REG00014	SLC25A12	GSE92220	.	.	.	.
REG00014	SLC25A15	GSE92220	.	.	.	.
REG00014	SLC25A16	GSE92220	.	.	.	.
REG00014	SLC25A17	GSE92220	.	.	.	.
REG00014	SLC25A3	GSE92220	.	.	.	.
REG00014	SLC25A4	GSE92220	.	.	.	.
REG00014	SLC25A5	GSE92220	.	.	.	.
REG00014	SLC25A6	GSE92220	.	.	.	.
REG00014	SLC26A1	GSE92220	.	.	.	.
REG00014	SLC27A1	GSE92220	.	.	.	.
REG00014	SLC27A3	GSE92220	.	.	.	.
REG00014	SLC27A4	GSE92220	.	.	.	.
REG00014	SLC29A1	GSE92220	.	.	.	.
REG00014	SLC29A2	GSE92220	.	.	.	.
REG00014	SLC2A2	GSE92220	.	.	.	.
REG00014	SLC2A3	GSE92220	.	.	.	.
REG00014	SLC2A4	GSE92220	.	.	.	.
REG00014	SLC2A5	GSE92220	.	.	.	.
REG00014	SLC2A6	GSE92220	.	.	.	.
REG00014	SLC30A1	GSE92220	.	.	.	.
REG00014	SLC30A3	GSE92220	.	.	.	.
REG00014	SLC31A2	GSE92220	.	.	.	.
REG00014	BRD1	GSE92220	.	.	.	.
REG00014	SLC35A1	GSE92220	.	.	.	.
REG00014	SLC35A2	GSE92220	.	.	.	.
REG00014	SLC3A2	GSE92220	.	.	.	.
REG00014	SLC4A2	GSE92220	.	.	.	.
REG00014	BRD2	GSE92220	.	.	.	.
REG00014	SLC4A7	GSE92220	.	.	.	.
REG00014	SLC5A2	GSE92220	.	.	.	.
REG00014	BRD3	GSE92220	.	.	.	.
REG00014	SLC6A1	GSE92220	.	.	.	.
REG00014	SLC6A11	GSE92220	.	.	.	.
REG00014	SLC6A13	GSE92220	.	.	.	.
REG00014	SLC6A3	GSE92220	.	.	.	.
REG00014	SLC6A4	GSE92220	.	.	.	.
REG00014	SLC6A6	GSE92220	.	.	.	.
REG00014	SLC6A8	GSE92220	.	.	.	.
REG00014	SLC6A9	GSE92220	.	.	.	.
REG00014	SLC7A11	GSE92220	.	.	.	.
REG00014	SLC7A2	GSE92220	.	.	.	.
REG00014	SLC7A5	GSE92220	.	.	.	.
REG00014	SLC7A6	GSE92220	.	.	.	.

REG00014	ZFP36L1	GSE92220	.	.	.	.
REG00014	SLC8A3	GSE92220	.	.	.	.
REG00014	SLC9A1	GSE92220	.	.	.	.
REG00014	SLC9A3R2	GSE92220	.	.	.	.
REG00014	SLC9A5	GSE92220	.	.	.	.
REG00014	ZFP36L2	GSE92220	.	.	.	.
REG00014	BRI3	GSE92220	.	.	.	.
REG00014	SMARCA1	GSE92220	.	.	.	.
REG00014	SMARCA2	GSE92220	.	.	.	.
REG00014	SMARCA4	GSE92220	.	.	.	.
REG00014	SMARCA5	GSE92220	.	.	.	.
REG00014	SMARCAL1	GSE92220	.	.	.	.
REG00014	SMARCB1	GSE92220	.	.	.	.
REG00014	SMARCC1	GSE92220	.	.	.	.
REG00014	SMARCC2	GSE92220	.	.	.	.
REG00014	SMARCD1	GSE92220	.	.	.	.
REG00014	SMARCD2	GSE92220	.	.	.	.
REG00014	SMARCE1	GSE92220	.	.	.	.
REG00014	ARID1A	GSE92220	.	.	.	.
REG00014	KDM5C	GSE92220	.	.	.	.
REG00014	KDM5D	GSE92220	.	.	.	.
REG00014	SMN1	GSE92220	.	.	.	.
REG00014	SMN2	GSE92220	.	.	.	.
REG00014	SMO	GSE92220	.	.	.	.
REG00014	SMPD1	GSE92220	.	.	.	.
REG00014	SUMO2	GSE92220	.	.	.	.
REG00014	SMTN	GSE92220	.	.	.	.
REG00014	SNAP29	GSE92220	.	.	.	.
REG00014	SNAPC2	GSE92220	.	.	.	.
REG00014	SNAPC3	GSE92220	.	.	.	.
REG00014	SNCG	GSE92220	.	.	.	.
REG00014	SNN	GSE92220	.	.	.	.
REG00014	SNRPA	GSE92220	.	.	.	.
REG00014	SNRPA1	GSE92220	.	.	.	.
REG00014	SNRPB	GSE92220	.	.	.	.
REG00014	SNRPB2	GSE92220	.	.	.	.
REG00014	SNRPC	GSE92220	.	.	.	.
REG00014	SNRPD1	GSE92220	.	.	.	.
REG00014	SNRPD2	GSE92220	.	.	.	.
REG00014	BSG	GSE92220	.	.	.	.
REG00014	SNRPE	GSE92220	.	.	.	.
REG00014	SNRPF	GSE92220	.	.	.	.
REG00014	SNRPG	GSE92220	.	.	.	.
REG00014	SNTB1	GSE92220	.	.	.	.
REG00014	SNTB2	GSE92220	.	.	.	.
REG00014	SNX1	GSE92220	.	.	.	.
REG00014	SOAT2	GSE92220	.	.	.	.
REG00014	SOD1	GSE92220	.	.	.	.
REG00014	SOD2	GSE92220	.	.	.	.
REG00014	SON	GSE92220	.	.	.	.

REG00014	SORD	GSE92220	.	.	.	.
REG00014	SORL1	GSE92220	.	.	.	.
REG00014	SORT1	GSE92220	.	.	.	.
REG00014	SOS1	GSE92220	.	.	.	.
REG00014	SOS2	GSE92220	.	.	.	.
REG00014	BST2	GSE92220	.	.	.	.
REG00014	SOX13	GSE92220	.	.	.	.
REG00014	SOX18	GSE92220	.	.	.	.
REG00014	SOX2	GSE92220	.	.	.	.
REG00014	SOX15	GSE92220	.	.	.	.
REG00014	SOX12	GSE92220	.	.	.	.
REG00014	SOX4	GSE92220	.	.	.	.
REG00014	SOX5	GSE92220	.	.	.	.
REG00014	SOX9	GSE92220	.	.	.	.
REG00014	SP1	GSE92220	.	.	.	.
REG00014	SP100	GSE92220	.	.	.	.
REG00014	SP2	GSE92220	.	.	.	.
REG00014	SP3	GSE92220	.	.	.	.
REG00014	SPAG4	GSE92220	.	.	.	.
REG00014	SPAG7	GSE92220	.	.	.	.
REG00014	BTD	GSE92220	.	.	.	.
REG00014	SPG11	GSE92220	.	.	.	.
REG00014	KLF9	GSE92220	.	.	.	.
REG00014	SPAST	GSE92220	.	.	.	.
REG00014	SPG7	GSE92220	.	.	.	.
REG00014	SPHK1	GSE92220	.	.	.	.
REG00014	BTF3	GSE92220	.	.	.	.
REG00014	SPON2	GSE92220	.	.	.	.
REG00014	SPP1	GSE92220	.	.	.	.
REG00014	SPR	GSE92220	.	.	.	.
REG00014	SPRY1	GSE92220	.	.	.	.
REG00014	SPTAN1	GSE92220	.	.	.	.
REG00014	SPTBN1	GSE92220	.	.	.	.
REG00014	SPTLC1	GSE92220	.	.	.	.
REG00014	SPTLC2	GSE92220	.	.	.	.
REG00014	SQLE	GSE92220	.	.	.	.
REG00014	SQSTM1	GSE92220	.	.	.	.
REG00014	SRC	GSE92220	.	.	.	.
REG00014	SRD5A1	GSE92220	.	.	.	.
REG00014	SREBF1	GSE92220	.	.	.	.
REG00014	SREBF2	GSE92220	.	.	.	.
REG00014	SRF	GSE92220	.	.	.	.
REG00014	SRM	GSE92220	.	.	.	.
REG00014	SRP14	GSE92220	.	.	.	.
REG00014	BTG1	GSE92220	.	.	.	.
REG00014	SRP19	GSE92220	.	.	.	.
REG00014	SRP54	GSE92220	.	.	.	.
REG00014	SRP68	GSE92220	.	.	.	.
REG00014	SRP72	GSE92220	.	.	.	.
REG00014	SRPK1	GSE92220	.	.	.	.

REG00014	SRPK2	GSE92220	.	.	.	.
REG00014	SRPRA	GSE92220	.	.	.	.
REG00014	BTG2	GSE92220	.	.	.	.
REG00014	SSB	GSE92220	.	.	.	.
REG00014	SSBP1	GSE92220	.	.	.	.
REG00014	SSFA2	GSE92220	.	.	.	.
REG00014	BTG3	GSE92220	.	.	.	.
REG00014	SSPN	GSE92220	.	.	.	.
REG00014	SSR1	GSE92220	.	.	.	.
REG00014	SSR2	GSE92220	.	.	.	.
REG00014	SSR3	GSE92220	.	.	.	.
REG00014	SSR4	GSE92220	.	.	.	.
REG00014	SSRP1	GSE92220	.	.	.	.
REG00014	SSTR5	GSE92220	.	.	.	.
REG00014	SS18	GSE92220	.	.	.	.
REG00014	ST13	GSE92220	.	.	.	.
REG00014	ST14	GSE92220	.	.	.	.
REG00014	STAG1	GSE92220	.	.	.	.
REG00014	STAG2	GSE92220	.	.	.	.
REG00014	STAM	GSE92220	.	.	.	.
REG00014	STAR	GSE92220	.	.	.	.
REG00014	BTN2A1	GSE92220	.	.	.	.
REG00014	STAT1	GSE92220	.	.	.	.
REG00014	STAT2	GSE92220	.	.	.	.
REG00014	STAT3	GSE92220	.	.	.	.
REG00014	STAT5A	GSE92220	.	.	.	.
REG00014	STAT5B	GSE92220	.	.	.	.
REG00014	STAT6	GSE92220	.	.	.	.
REG00014	BTN2A2	GSE92220	.	.	.	.
REG00014	STAU1	GSE92220	.	.	.	.
REG00014	STAU2	GSE92220	.	.	.	.
REG00014	STC2	GSE92220	.	.	.	.
REG00014	HSPA13	GSE92220	.	.	.	.
REG00014	BTN3A1	GSE92220	.	.	.	.
REG00014	STIP1	GSE92220	.	.	.	.
REG00014	STK11	GSE92220	.	.	.	.
REG00014	AURKB	GSE92220	.	.	.	.
REG00014	AURKC	GSE92220	.	.	.	.
REG00014	AURKA	GSE92220	.	.	.	.
REG00014	STK16	GSE92220	.	.	.	.
REG00014	STK17A	GSE92220	.	.	.	.
REG00014	PLK4	GSE92220	.	.	.	.
REG00014	STK19	GSE92220	.	.	.	.
REG00014	STK24	GSE92220	.	.	.	.
REG00014	STK25	GSE92220	.	.	.	.
REG00014	STK4	GSE92220	.	.	.	.
REG00014	CENPX	GSE92220	.	.	.	.
REG00014	STRN	GSE92220	.	.	.	.
REG00014	STS	GSE92220	.	.	.	.
REG00014	STUB1	GSE92220	.	.	.	.

REG00014	STX10	GSE92220	.	.	.	.
REG00014	STX16	GSE92220	.	.	.	.
REG00014	STX17	GSE92220	.	.	.	.
REG00014	STX4	GSE92220	.	.	.	.
REG00014	STX6	GSE92220	.	.	.	.
REG00014	STX7	GSE92220	.	.	.	.
REG00014	STXBP1	GSE92220	.	.	.	.
REG00014	STXBP2	GSE92220	.	.	.	.
REG00014	STXBP3	GSE92220	.	.	.	.
REG00014	STYX	GSE92220	.	.	.	.
REG00014	SUCLG1	GSE92220	.	.	.	.
REG00014	SUCLG2	GSE92220	.	.	.	.
REG00014	RIOK3	GSE92220	.	.	.	.
REG00014	SULT1A1	GSE92220	.	.	.	.
REG00014	SULT1A2	GSE92220	.	.	.	.
REG00014	SULT2A1	GSE92220	.	.	.	.
REG00014	SUOX	GSE92220	.	.	.	.
REG00014	SUPT16H	GSE92220	.	.	.	.
REG00014	SUPT3H	GSE92220	.	.	.	.
REG00014	SUPT4H1	GSE92220	.	.	.	.
REG00014	SUPT5H	GSE92220	.	.	.	.
REG00014	SUPT6H	GSE92220	.	.	.	.
REG00014	SUPV3L1	GSE92220	.	.	.	.
REG00014	SURF2	GSE92220	.	.	.	.
REG00014	SURF4	GSE92220	.	.	.	.
REG00014	SURF6	GSE92220	.	.	.	.
REG00014	SYN3	GSE92220	.	.	.	.
REG00014	SYNGR2	GSE92220	.	.	.	.
REG00014	ACLY	GSE92220	.	.	.	.
REG00014	SYNGR3	GSE92220	.	.	.	.
REG00014	SYNJ2	GSE92220	.	.	.	.
REG00014	SYPL1	GSE92220	.	.	.	.
REG00014	BUB3	GSE92220	.	.	.	.
REG00014	SYT2	GSE92220	.	.	.	.
REG00014	SYT5	GSE92220	.	.	.	.
REG00014	BVES	GSE92220	.	.	.	.
REG00014	TACC1	GSE92220	.	.	.	.
REG00014	TACC2	GSE92220	.	.	.	.
REG00014	TACC3	GSE92220	.	.	.	.
REG00014	EPCAM	GSE92220	.	.	.	.
REG00014	TAF1C	GSE92220	.	.	.	.
REG00014	TAF1	GSE92220	.	.	.	.
REG00014	TAF4	GSE92220	.	.	.	.
REG00014	TAF7	GSE92220	.	.	.	.
REG00014	TAF10	GSE92220	.	.	.	.
REG00014	TAF11	GSE92220	.	.	.	.
REG00014	TAF12	GSE92220	.	.	.	.
REG00014	TAF13	GSE92220	.	.	.	.
REG00014	TAF15	GSE92220	.	.	.	.
REG00014	BRF1	GSE92220	.	.	.	.

REG00014	TAGLN	GSE92220	.	.	.	.
REG00014	TAGLN2	GSE92220	.	.	.	.
REG00014	TAL2	GSE92220	.	.	.	.
REG00014	TALDO1	GSE92220	.	.	.	.
REG00014	TANK	GSE92220	.	.	.	.
REG00014	TARBP1	GSE92220	.	.	.	.
REG00014	TARBP2	GSE92220	.	.	.	.
REG00014	BYSL	GSE92220	.	.	.	.
REG00014	TARDBP	GSE92220	.	.	.	.
REG00014	TARS	GSE92220	.	.	.	.
REG00014	TAX1BP1	GSE92220	.	.	.	.
REG00014	TBC1D1	GSE92220	.	.	.	.
REG00014	TBCA	GSE92220	.	.	.	.
REG00014	TSPO	GSE92220	.	.	.	.
REG00014	TBCC	GSE92220	.	.	.	.
REG00014	TBCD	GSE92220	.	.	.	.
REG00014	SERPINA7	GSE92220	.	.	.	.
REG00014	TBK1	GSE92220	.	.	.	.
REG00014	TBL1X	GSE92220	.	.	.	.
REG00014	TBL2	GSE92220	.	.	.	.
REG00014	TBP	GSE92220	.	.	.	.
REG00014	TBR1	GSE92220	.	.	.	.
REG00014	TBX2	GSE92220	.	.	.	.
REG00014	TWNK	GSE92220	.	.	.	.
REG00014	TBX3	GSE92220	.	.	.	.
REG00014	TBX6	GSE92220	.	.	.	.
REG00014	TCAP	GSE92220	.	.	.	.
REG00014	TCEA1	GSE92220	.	.	.	.
REG00014	TCEA2	GSE92220	.	.	.	.
REG00014	TCEA3	GSE92220	.	.	.	.
REG00014	ELOC	GSE92220	.	.	.	.
REG00014	ELOB	GSE92220	.	.	.	.
REG00014	ELOA	GSE92220	.	.	.	.
REG00014	HNF1A	GSE92220	.	.	.	.
REG00014	TCF12	GSE92220	.	.	.	.
REG00014	TCF20	GSE92220	.	.	.	.
REG00014	TCF3	GSE92220	.	.	.	.
REG00014	TCF7	GSE92220	.	.	.	.
REG00014	TCF7L1	GSE92220	.	.	.	.
REG00014	TCF7L2	GSE92220	.	.	.	.
REG00014	ZEB1	GSE92220	.	.	.	.
REG00014	MLX	GSE92220	.	.	.	.
REG00014	TCFL5	GSE92220	.	.	.	.
REG00014	TCIRG1	GSE92220	.	.	.	.
REG00014	DAGLA	GSE92220	.	.	.	.
REG00014	TCOF1	GSE92220	.	.	.	.
REG00014	TCP1	GSE92220	.	.	.	.
REG00014	TCP11	GSE92220	.	.	.	.
REG00014	TCTA	GSE92220	.	.	.	.
REG00014	TCTE1	GSE92220	.	.	.	.

REG00014	DYNLT1	GSE92220	.	.	.	.
REG00014	SERINC3	GSE92220	.	.	.	.
REG00014	ACO1	GSE92220	.	.	.	.
REG00014	TEAD1	GSE92220	.	.	.	.
REG00014	TEAD2	GSE92220	.	.	.	.
REG00014	TEAD3	GSE92220	.	.	.	.
REG00014	TEAD4	GSE92220	.	.	.	.
REG00014	TEF	GSE92220	.	.	.	.
REG00014	TEP1	GSE92220	.	.	.	.
REG00014	TERF2	GSE92220	.	.	.	.
REG00014	PPP6R3	GSE92220	.	.	.	.
REG00014	TERT	GSE92220	.	.	.	.
REG00014	C11orf24	GSE92220	.	.	.	.
REG00014	TF	GSE92220	.	.	.	.
REG00014	TFAM	GSE92220	.	.	.	.
REG00014	TFAP4	GSE92220	.	.	.	.
REG00014	TFDP1	GSE92220	.	.	.	.
REG00014	TFDP2	GSE92220	.	.	.	.
REG00014	TFE3	GSE92220	.	.	.	.
REG00014	TFEB	GSE92220	.	.	.	.
REG00014	TFG	GSE92220	.	.	.	.
REG00014	MRPL49	GSE92220	.	.	.	.
REG00014	TFPI	GSE92220	.	.	.	.
REG00014	TFR2	GSE92220	.	.	.	.
REG00014	TFRC	GSE92220	.	.	.	.
REG00014	TGFB1	GSE92220	.	.	.	.
REG00014	TGFB2	GSE92220	.	.	.	.
REG00014	ZNHIT2	GSE92220	.	.	.	.
REG00014	TGFBI	GSE92220	.	.	.	.
REG00014	TGFBR1	GSE92220	.	.	.	.
REG00014	TGFBR2	GSE92220	.	.	.	.
REG00014	TGFBR3	GSE92220	.	.	.	.
REG00014	TGIF1	GSE92220	.	.	.	.
REG00014	TGM1	GSE92220	.	.	.	.
REG00014	TGM2	GSE92220	.	.	.	.
REG00014	THBS1	GSE92220	.	.	.	.
REG00014	THOP1	GSE92220	.	.	.	.
REG00014	THPO	GSE92220	.	.	.	.
REG00014	THRB	GSE92220	.	.	.	.
REG00014	THRSP	GSE92220	.	.	.	.
REG00014	TIA1	GSE92220	.	.	.	.
REG00014	TIAL1	GSE92220	.	.	.	.
REG00014	DNAJA3	GSE92220	.	.	.	.
REG00014	MYRF	GSE92220	.	.	.	.
REG00014	KLF10	GSE92220	.	.	.	.
REG00014	KLF11	GSE92220	.	.	.	.
REG00014	TRIM24	GSE92220	.	.	.	.
REG00014	TIMELESS	GSE92220	.	.	.	.
REG00014	TIMM13	GSE92220	.	.	.	.
REG00014	TIMM8B	GSE92220	.	.	.	.

REG00014	TIMM9	GSE92220	.	.	.	.
REG00014	TIMP2	GSE92220	.	.	.	.
REG00014	TIMP3	GSE92220	.	.	.	.
REG00014	TINF2	GSE92220	.	.	.	.
REG00014	TJP1	GSE92220	.	.	.	.
REG00014	TJP2	GSE92220	.	.	.	.
REG00014	TKT	GSE92220	.	.	.	.
REG00014	TLE1	GSE92220	.	.	.	.
REG00014	TLE2	GSE92220	.	.	.	.
REG00014	TLE3	GSE92220	.	.	.	.
REG00014	TLK1	GSE92220	.	.	.	.
REG00014	TLK2	GSE92220	.	.	.	.
REG00014	TLL2	GSE92220	.	.	.	.
REG00014	SEC62	GSE92220	.	.	.	.
REG00014	TSPAN7	GSE92220	.	.	.	.
REG00014	TM4SF4	GSE92220	.	.	.	.
REG00014	TM4SF5	GSE92220	.	.	.	.
REG00014	TSPAN6	GSE92220	.	.	.	.
REG00014	TSPAN4	GSE92220	.	.	.	.
REG00014	TM6SF2	GSE92220	.	.	.	.
REG00014	TM7SF2	GSE92220	.	.	.	.
REG00014	TM9SF1	GSE92220	.	.	.	.
REG00014	TM9SF2	GSE92220	.	.	.	.
REG00014	TMEFF1	GSE92220	.	.	.	.
REG00014	TRAPPC10	GSE92220	.	.	.	.
REG00014	TMEM2	GSE92220	.	.	.	.
REG00014	C14orf1	GSE92220	.	.	.	.
REG00014	TMF1	GSE92220	.	.	.	.
REG00014	TMOD4	GSE92220	.	.	.	.
REG00014	TMSB10	GSE92220	.	.	.	.
REG00014	C14orf2	GSE92220	.	.	.	.
REG00014	AHSA1	GSE92220	.	.	.	.
REG00014	CLEC3B	GSE92220	.	.	.	.
REG00014	TNFAIP1	GSE92220	.	.	.	.
REG00014	TNFRSF10B	GSE92220	.	.	.	.
REG00014	TNFRSF10D	GSE92220	.	.	.	.
REG00014	TNFRSF25	GSE92220	.	.	.	.
REG00014	TNFRSF19	GSE92220	.	.	.	.
REG00014	TNFRSF1A	GSE92220	.	.	.	.
REG00014	TNFRSF4	GSE92220	.	.	.	.
REG00014	FAS	GSE92220	.	.	.	.
REG00014	TNFRSF6B	GSE92220	.	.	.	.
REG00014	TNFSF10	GSE92220	.	.	.	.
REG00014	TNFSF14	GSE92220	.	.	.	.
REG00014	FASLG	GSE92220	.	.	.	.
REG00014	TNKS	GSE92220	.	.	.	.
REG00014	TNNT2	GSE92220	.	.	.	.
REG00014	MED12	GSE92220	.	.	.	.
REG00014	EP400	GSE92220	.	.	.	.
REG00014	GIGYF2	GSE92220	.	.	.	.

REG00014	TNRC18	GSE92220	.	.	.	.
REG00014	CELF3	GSE92220	.	.	.	.
REG00014	CNPY3	GSE92220	.	.	.	.
REG00014	TNRC6A	GSE92220	.	.	.	.
REG00014	TOX3	GSE92220	.	.	.	.
REG00014	TNXB	GSE92220	.	.	.	.
REG00014	TOB1	GSE92220	.	.	.	.
REG00014	TOB2	GSE92220	.	.	.	.
REG00014	TOM1	GSE92220	.	.	.	.
REG00014	TOM1L1	GSE92220	.	.	.	.
REG00014	TOP1	GSE92220	.	.	.	.
REG00014	TOP2A	GSE92220	.	.	.	.
REG00014	TOP2B	GSE92220	.	.	.	.
REG00014	TOR1B	GSE92220	.	.	.	.
REG00014	TOR3A	GSE92220	.	.	.	.
REG00014	TP53	GSE92220	.	.	.	.
REG00014	ACOX2	GSE92220	.	.	.	.
REG00014	TPD52	GSE92220	.	.	.	.
REG00014	TPD52L2	GSE92220	.	.	.	.
REG00014	TPH1	GSE92220	.	.	.	.
REG00014	TPI1	GSE92220	.	.	.	.
REG00014	TPM1	GSE92220	.	.	.	.
REG00014	TPM2	GSE92220	.	.	.	.
REG00014	TPM3	GSE92220	.	.	.	.
REG00014	TPM4	GSE92220	.	.	.	.
REG00014	TPP2	GSE92220	.	.	.	.
REG00014	TPR	GSE92220	.	.	.	.
REG00014	TPST1	GSE92220	.	.	.	.
REG00014	TPT1	GSE92220	.	.	.	.
REG00014	TRADD	GSE92220	.	.	.	.
REG00014	TRAF1	GSE92220	.	.	.	.
REG00014	TRAF3	GSE92220	.	.	.	.
REG00014	TRAF4	GSE92220	.	.	.	.
REG00014	TRAF5	GSE92220	.	.	.	.
REG00014	ACOX3	GSE92220	.	.	.	.
REG00014	LDLRAD4	GSE92220	.	.	.	.
REG00014	GIPC1	GSE92220	.	.	.	.
REG00014	TREX1	GSE92220	.	.	.	.
REG00014	SERPING1	GSE92220	.	.	.	.
REG00014	TRIO	GSE92220	.	.	.	.
REG00014	TRIP10	GSE92220	.	.	.	.
REG00014	TRIP11	GSE92220	.	.	.	.
REG00014	TRIP12	GSE92220	.	.	.	.
REG00014	ZNHIT3	GSE92220	.	.	.	.
REG00014	TRIP4	GSE92220	.	.	.	.
REG00014	TRIP6	GSE92220	.	.	.	.
REG00014	JMJD1C	GSE92220	.	.	.	.
REG00014	EGLN1	GSE92220	.	.	.	.
REG00014	TRO	GSE92220	.	.	.	.
REG00014	FAM189B	GSE92220	.	.	.	.

REG00014	TRPC1	GSE92220	.	.	.	.
REG00014	TRRAP	GSE92220	.	.	.	.
REG00014	TSC2	GSE92220	.	.	.	.
REG00014	TSFM	GSE92220	.	.	.	.
REG00014	UBQLN4	GSE92220	.	.	.	.
REG00014	TSN	GSE92220	.	.	.	.
REG00014	TSSC1	GSE92220	.	.	.	.
REG00014	PHLDA2	GSE92220	.	.	.	.
REG00014	TSSC4	GSE92220	.	.	.	.
REG00014	TST	GSE92220	.	.	.	.
REG00014	TMEM59	GSE92220	.	.	.	.
REG00014	TSTA3	GSE92220	.	.	.	.
REG00014	DNAJC7	GSE92220	.	.	.	.
REG00014	TTC3	GSE92220	.	.	.	.
REG00014	SUCO	GSE92220	.	.	.	.
REG00014	TTR	GSE92220	.	.	.	.
REG00014	TUB	GSE92220	.	.	.	.
REG00014	TUBA8	GSE92220	.	.	.	.
REG00014	TUBB2A	GSE92220	.	.	.	.
REG00014	TUBG1	GSE92220	.	.	.	.
REG00014	TUBG2	GSE92220	.	.	.	.
REG00014	TUFM	GSE92220	.	.	.	.
REG00014	TULP1	GSE92220	.	.	.	.
REG00014	TULP3	GSE92220	.	.	.	.
REG00014	TWSG1	GSE92220	.	.	.	.
REG00014	C1QBP	GSE92220	.	.	.	.
REG00014	TXN	GSE92220	.	.	.	.
REG00014	TXNL1	GSE92220	.	.	.	.
REG00014	TXNRD1	GSE92220	.	.	.	.
REG00014	TYK2	GSE92220	.	.	.	.
REG00014	TYMS	GSE92220	.	.	.	.
REG00014	TYRO3	GSE92220	.	.	.	.
REG00014	UAP1	GSE92220	.	.	.	.
REG00014	UBA52	GSE92220	.	.	.	.
REG00014	C1R	GSE92220	.	.	.	.
REG00014	UBAP1	GSE92220	.	.	.	.
REG00014	UBB	GSE92220	.	.	.	.
REG00014	UBC	GSE92220	.	.	.	.
REG00014	UBA1	GSE92220	.	.	.	.
REG00014	C1S	GSE92220	.	.	.	.
REG00014	UBA3	GSE92220	.	.	.	.
REG00014	UBE2A	GSE92220	.	.	.	.
REG00014	UBE2B	GSE92220	.	.	.	.
REG00014	UBE2D2	GSE92220	.	.	.	.
REG00014	UBE2D3	GSE92220	.	.	.	.
REG00014	UBE2E1	GSE92220	.	.	.	.
REG00014	UBE2E3	GSE92220	.	.	.	.
REG00014	C2	GSE92220	.	.	.	.
REG00014	UBE2F	GSE92220	.	.	.	.
REG00014	UBE2G1	GSE92220	.	.	.	.

REG00014	UBE2G2	GSE92220	.	.	.	.
REG00014	UBE2H	GSE92220	.	.	.	.
REG00014	UBE2I	GSE92220	.	.	.	.
REG00014	UBE2M	GSE92220	.	.	.	.
REG00014	UBE2N	GSE92220	.	.	.	.
REG00014	UBE2V1	GSE92220	.	.	.	.
REG00014	UBE2V2	GSE92220	.	.	.	.
REG00014	UBE3A	GSE92220	.	.	.	.
REG00014	UBE4B	GSE92220	.	.	.	.
REG00014	SUMO1	GSE92220	.	.	.	.
REG00014	UBL3	GSE92220	.	.	.	.
REG00014	UBL4A	GSE92220	.	.	.	.
REG00014	UBN1	GSE92220	.	.	.	.
REG00014	UBP1	GSE92220	.	.	.	.
REG00014	UBQLN2	GSE92220	.	.	.	.
REG00014	UCN	GSE92220	.	.	.	.
REG00014	UCP1	GSE92220	.	.	.	.
REG00014	UGDH	GSE92220	.	.	.	.
REG00014	UGP2	GSE92220	.	.	.	.
REG00014	FAM3B	GSE92220	.	.	.	.
REG00014	UGT2B10	GSE92220	.	.	.	.
REG00014	UGT2B7	GSE92220	.	.	.	.
REG00014	ULK1	GSE92220	.	.	.	.
REG00014	UCK2	GSE92220	.	.	.	.
REG00014	UMPS	GSE92220	.	.	.	.
REG00014	UNC119	GSE92220	.	.	.	.
REG00014	UNG	GSE92220	.	.	.	.
REG00014	UPK1A	GSE92220	.	.	.	.
REG00014	SETD4	GSE92220	.	.	.	.
REG00014	UQCRB	GSE92220	.	.	.	.
REG00014	UQCRC1	GSE92220	.	.	.	.
REG00014	UQCRC2	GSE92220	.	.	.	.
REG00014	UQCRFS1	GSE92220	.	.	.	.
REG00014	UQCRH	GSE92220	.	.	.	.
REG00014	UROD	GSE92220	.	.	.	.
REG00014	USF2	GSE92220	.	.	.	.
REG00014	C21orf2	GSE92220	.	.	.	.
REG00014	USP1	GSE92220	.	.	.	.
REG00014	USP10	GSE92220	.	.	.	.
REG00014	USP11	GSE92220	.	.	.	.
REG00014	USP13	GSE92220	.	.	.	.
REG00014	USP14	GSE92220	.	.	.	.
REG00014	USP19	GSE92220	.	.	.	.
REG00014	USP2	GSE92220	.	.	.	.
REG00014	USP21	GSE92220	.	.	.	.
REG00014	USP22	GSE92220	.	.	.	.
REG00014	USP24	GSE92220	.	.	.	.
REG00014	USP25	GSE92220	.	.	.	.
REG00014	USP3	GSE92220	.	.	.	.
REG00014	USP4	GSE92220	.	.	.	.

REG00014	USP7	GSE92220	.	.	.	.
REG00014	USP9X	GSE92220	.	.	.	.
REG00014	UTRN	GSE92220	.	.	.	.
REG00014	KDM6A	GSE92220	.	.	.	.
REG00014	VAMP1	GSE92220	.	.	.	.
REG00014	VAMP2	GSE92220	.	.	.	.
REG00014	VAMP3	GSE92220	.	.	.	.
REG00014	VAMP5	GSE92220	.	.	.	.
REG00014	VAPA	GSE92220	.	.	.	.
REG00014	VAPB	GSE92220	.	.	.	.
REG00014	VARS	GSE92220	.	.	.	.
REG00014	VASP	GSE92220	.	.	.	.
REG00014	VAV2	GSE92220	.	.	.	.
REG00014	VAV3	GSE92220	.	.	.	.
REG00014	C2CD2	GSE92220	.	.	.	.
REG00014	VAX2	GSE92220	.	.	.	.
REG00014	VBP1	GSE92220	.	.	.	.
REG00014	VCP	GSE92220	.	.	.	.
REG00014	VDAC1	GSE92220	.	.	.	.
REG00014	VDAC2	GSE92220	.	.	.	.
REG00014	VDAC3	GSE92220	.	.	.	.
REG00014	VEGFB	GSE92220	.	.	.	.
REG00014	VHL	GSE92220	.	.	.	.
REG00014	VIL1	GSE92220	.	.	.	.
REG00014	VLDLR	GSE92220	.	.	.	.
REG00014	VPS26A	GSE92220	.	.	.	.
REG00014	TRPV1	GSE92220	.	.	.	.
REG00014	VSNL1	GSE92220	.	.	.	.
REG00014	VTN	GSE92220	.	.	.	.
REG00014	WARS	GSE92220	.	.	.	.
REG00014	C21orf33	GSE92220	.	.	.	.
REG00014	WARS2	GSE92220	.	.	.	.
REG00014	WASF1	GSE92220	.	.	.	.
REG00014	WASF2	GSE92220	.	.	.	.
REG00014	WASF3	GSE92220	.	.	.	.
REG00014	WASL	GSE92220	.	.	.	.
REG00014	WBP1	GSE92220	.	.	.	.
REG00014	WBP4	GSE92220	.	.	.	.
REG00014	EIF4H	GSE92220	.	.	.	.
REG00014	MLXIPL	GSE92220	.	.	.	.
REG00014	WDR1	GSE92220	.	.	.	.
REG00014	WDR4	GSE92220	.	.	.	.
REG00014	WDR5	GSE92220	.	.	.	.
REG00014	WDR6	GSE92220	.	.	.	.
REG00014	BRWD1	GSE92220	.	.	.	.
REG00014	WEE1	GSE92220	.	.	.	.
REG00014	WFS1	GSE92220	.	.	.	.
REG00014	NSD2	GSE92220	.	.	.	.
REG00014	NSD3	GSE92220	.	.	.	.
REG00014	NELFA	GSE92220	.	.	.	.

REG00014	WISP1	GSE92220	.	.	.	.
REG00014	WNT11	GSE92220	.	.	.	.
REG00014	WNT3	GSE92220	.	.	.	.
REG00014	WRB	GSE92220	.	.	.	.
REG00014	WWOX	GSE92220	.	.	.	.
REG00014	TMEM50B	GSE92220	.	.	.	.
REG00014	XBP1	GSE92220	.	.	.	.
REG00014	XG	GSE92220	.	.	.	.
REG00014	XPA	GSE92220	.	.	.	.
REG00014	XPC	GSE92220	.	.	.	.
REG00014	REXO4	GSE92220	.	.	.	.
REG00014	XPO1	GSE92220	.	.	.	.
REG00014	XPOT	GSE92220	.	.	.	.
REG00014	XPR1	GSE92220	.	.	.	.
REG00014	XRCC3	GSE92220	.	.	.	.
REG00014	XRCC4	GSE92220	.	.	.	.
REG00014	XRCC5	GSE92220	.	.	.	.
REG00014	XRN2	GSE92220	.	.	.	.
REG00014	XYLB	GSE92220	.	.	.	.
REG00014	YES1	GSE92220	.	.	.	.
REG00014	YME1L1	GSE92220	.	.	.	.
REG00014	YPEL1	GSE92220	.	.	.	.
REG00014	YWHAB	GSE92220	.	.	.	.
REG00014	YWHAE	GSE92220	.	.	.	.
REG00014	YWHAG	GSE92220	.	.	.	.
REG00014	YWHAH	GSE92220	.	.	.	.
REG00014	YWHAQ	GSE92220	.	.	.	.
REG00014	YWHAZ	GSE92220	.	.	.	.
REG00014	YY1	GSE92220	.	.	.	.
REG00014	ZAP70	GSE92220	.	.	.	.
REG00014	RNF103	GSE92220	.	.	.	.
REG00014	ZBTB14	GSE92220	.	.	.	.
REG00014	ZFP36	GSE92220	.	.	.	.
REG00014	ZFPL1	GSE92220	.	.	.	.
REG00014	ZFX	GSE92220	.	.	.	.
REG00014	ZNF101	GSE92220	.	.	.	.
REG00014	ZNF106	GSE92220	.	.	.	.
REG00014	ZNF117	GSE92220	.	.	.	.
REG00014	ACTA1	GSE92220	.	.	.	.
REG00014	ZNF12	GSE92220	.	.	.	.
REG00014	ZNF121	GSE92220	.	.	.	.
REG00014	ZNF124	GSE92220	.	.	.	.
REG00014	DOPEY2	GSE92220	.	.	.	.
REG00014	ZNF133	GSE92220	.	.	.	.
REG00014	ZNF142	GSE92220	.	.	.	.
REG00014	PCGF2	GSE92220	.	.	.	.
REG00014	ZNF148	GSE92220	.	.	.	.
REG00014	ZBTB17	GSE92220	.	.	.	.
REG00014	ZNF154	GSE92220	.	.	.	.
REG00014	VEZF1	GSE92220	.	.	.	.

REG00014	SF1	GSE92220	.	.	.	.
REG00014	TRIM26	GSE92220	.	.	.	.
REG00014	DNAJC28	GSE92220	.	.	.	.
REG00014	SPATC1L	GSE92220	.	.	.	.
REG00014	ZNF189	GSE92220	.	.	.	.
REG00014	ZMYM2	GSE92220	.	.	.	.
REG00014	ZNF202	GSE92220	.	.	.	.
REG00014	ZNF205	GSE92220	.	.	.	.
REG00014	ZNF207	GSE92220	.	.	.	.
REG00014	ZNF212	GSE92220	.	.	.	.
REG00014	ZFAND5	GSE92220	.	.	.	.
REG00014	ZNF217	GSE92220	.	.	.	.
REG00014	C21orf59	GSE92220	.	.	.	.
REG00014	ZNF219	GSE92220	.	.	.	.
REG00014	ZNF234	GSE92220	.	.	.	.
REG00014	ZNF236	GSE92220	.	.	.	.
REG00014	ZNF24	GSE92220	.	.	.	.
REG00014	ZNF251	GSE92220	.	.	.	.
REG00014	ZNF254	GSE92220	.	.	.	.
REG00014	ZPR1	GSE92220	.	.	.	.
REG00014	ZMYM3	GSE92220	.	.	.	.
REG00014	ZMYM4	GSE92220	.	.	.	.
REG00014	ZNF263	GSE92220	.	.	.	.
REG00014	ZRANB2	GSE92220	.	.	.	.
REG00014	ZNF274	GSE92220	.	.	.	.
REG00014	ZNF277	GSE92220	.	.	.	.
REG00014	PATZ1	GSE92220	.	.	.	.
REG00014	ZNF281	GSE92220	.	.	.	.
REG00014	ZNF282	GSE92220	.	.	.	.
REG00014	RHBDD3	GSE92220	.	.	.	.
REG00014	SCAPER	GSE92220	.	.	.	.
REG00014	ZBTB22	GSE92220	.	.	.	.
REG00014	ZNF3	GSE92220	.	.	.	.
REG00014	TBC1D22A	GSE92220	.	.	.	.
REG00014	RNF114	GSE92220	.	.	.	.
REG00014	TMEM184B	GSE92220	.	.	.	.
REG00014	ZKSCAN1	GSE92220	.	.	.	.
REG00014	ZNF37A	GSE92220	.	.	.	.
REG00014	ZSCAN21	GSE92220	.	.	.	.
REG00014	MZF1	GSE92220	.	.	.	.
REG00014	ZNF44	GSE92220	.	.	.	.
REG00014	ZBTB25	GSE92220	.	.	.	.
REG00014	ZNF48	GSE92220	.	.	.	.
REG00014	FAM118A	GSE92220	.	.	.	.
REG00014	ZNF69	GSE92220	.	.	.	.
REG00014	KIAA0930	GSE92220	.	.	.	.
REG00014	ZNF76	GSE92220	.	.	.	.
REG00014	ZNF77	GSE92220	.	.	.	.
REG00014	ZNF83	GSE92220	.	.	.	.
REG00014	EML4	GSE92220	.	.	.	.

REG00014	CNBP	GSE92220	.	.	.	.
REG00014	ZNF91	GSE92220	.	.	.	.
REG00014	C3	GSE92220	.	.	.	.
REG00014	ZNRD1	GSE92220	.	.	.	.
REG00014	ZP3	GSE92220	.	.	.	.
REG00014	DNAJC2	GSE92220	.	.	.	.
REG00014	ZXDB	GSE92220	.	.	.	.
REG00014	ACTB	GSE92220	.	.	.	.
REG00014	STRADB	GSE92220	.	.	.	.
REG00014	ANKRA2	GSE92220	.	.	.	.
REG00014	TIMMDC1	GSE92220	.	.	.	.
REG00014	ATP2C1	GSE92220	.	.	.	.
REG00014	ATP5L2	GSE92220	.	.	.	.
REG00014	CLDND1	GSE92220	.	.	.	.
REG00014	ANP32A	GSE92220	.	.	.	.
REG00014	URI1	GSE92220	.	.	.	.
REG00014	TMEM59L	GSE92220	.	.	.	.
REG00014	APMAP	GSE92220	.	.	.	.
REG00014	LMBR1	GSE92220	.	.	.	.
REG00014	NOM1	GSE92220	.	.	.	.
REG00014	FAM120A	GSE92220	.	.	.	.
REG00014	CAPN12	GSE92220	.	.	.	.
REG00014	C4BPA	GSE92220	.	.	.	.
REG00014	FTSJ1	GSE92220	.	.	.	.
REG00014	CYP4F11	GSE92220	.	.	.	.
REG00014	DDX24	GSE92220	.	.	.	.
REG00014	DEXI	GSE92220	.	.	.	.
REG00014	DUOX2	GSE92220	.	.	.	.
REG00014	C4BPB	GSE92220	.	.	.	.
REG00014	ERO1A	GSE92220	.	.	.	.
REG00014	FIGN	GSE92220	.	.	.	.
REG00014	SLC30A9	GSE92220	.	.	.	.
REG00014	GABARAPL2	GSE92220	.	.	.	.
REG00014	LGR4	GSE92220	.	.	.	.
REG00014	GPR62	GSE92220	.	.	.	.
REG00014	GPRC5C	GSE92220	.	.	.	.
REG00014	C5	GSE92220	.	.	.	.
REG00014	GSTO1	GSE92220	.	.	.	.
REG00014	LXN	GSE92220	.	.	.	.
REG00014	FAM13B	GSE92220	.	.	.	.
REG00014	MAP1LC3B	GSE92220	.	.	.	.
REG00014	MCOLN1	GSE92220	.	.	.	.
REG00014	KDM3B	GSE92220	.	.	.	.
REG00014	NIF3L1	GSE92220	.	.	.	.
REG00014	C6orf1	GSE92220	.	.	.	.
REG00014	PDCD11	GSE92220	.	.	.	.
REG00014	PHTF2	GSE92220	.	.	.	.
REG00014	PPP2R3B	GSE92220	.	.	.	.
REG00014	RLIM	GSE92220	.	.	.	.
REG00014	TRAF3IP2	GSE92220	.	.	.	.

REG00014	RNF19A	GSE92220	.	.	.	.
REG00014	RSPH14	GSE92220	.	.	.	.
REG00014	SLC2A10	GSE92220	.	.	.	.
REG00014	SLC2A9	GSE92220	.	.	.	.
REG00014	SLC38A1	GSE92220	.	.	.	.
REG00014	SLC38A2	GSE92220	.	.	.	.
REG00014	BANP	GSE92220	.	.	.	.
REG00014	SERINC1	GSE92220	.	.	.	.
REG00014	TNFRSF21	GSE92220	.	.	.	.
REG00014	TTYH1	GSE92220	.	.	.	.
REG00014	UBE3B	GSE92220	.	.	.	.
REG00014	ULK2	GSE92220	.	.	.	.
REG00014	UNC93B1	GSE92220	.	.	.	.
REG00014	VPS35	GSE92220	.	.	.	.
REG00014	VPS4A	GSE92220	.	.	.	.
REG00014	ZBTB20	GSE92220	.	.	.	.
REG00014	ARFGAP2	GSE92220	.	.	.	.
REG00014	ZNF317	GSE92220	.	.	.	.
REG00014	BIRC6	GSE92220	.	.	.	.
REG00014	CLIC4	GSE92220	.	.	.	.
REG00014	MESDC1	GSE92220	.	.	.	.
REG00014	C8A	GSE92220	.	.	.	.
REG00014	MESDC2	GSE92220	.	.	.	.
REG00014	PTTG1IP	GSE92220	.	.	.	.
REG00014	VPS9D1	GSE92220	.	.	.	.
REG00014	ATP6V1D	GSE92220	.	.	.	.
REG00014	B3GNT3	GSE92220	.	.	.	.
REG00014	CNPY2	GSE92220	.	.	.	.
REG00014	ATP8A2	GSE92220	.	.	.	.
REG00014	ATP8B2	GSE92220	.	.	.	.
REG00014	MARK4	GSE92220	.	.	.	.
REG00014	AGTRAP	GSE92220	.	.	.	.
REG00014	ATP9A	GSE92220	.	.	.	.
REG00014	ATP9B	GSE92220	.	.	.	.
REG00014	CHRAC1	GSE92220	.	.	.	.
REG00014	OSGIN2	GSE92220	.	.	.	.
REG00014	ATP11A	GSE92220	.	.	.	.
REG00014	SPOCK2	GSE92220	.	.	.	.
REG00014	C8orf4	GSE92220	.	.	.	.
REG00014	BRD4	GSE92220	.	.	.	.
REG00014	ZNF318	GSE92220	.	.	.	.
REG00014	PAXBP1	GSE92220	.	.	.	.
REG00014	C9	GSE92220	.	.	.	.
REG00014	FBXO4	GSE92220	.	.	.	.
REG00014	FBXO5	GSE92220	.	.	.	.
REG00014	FBXO6	GSE92220	.	.	.	.
REG00014	FBXO7	GSE92220	.	.	.	.
REG00014	FBXO9	GSE92220	.	.	.	.
REG00014	FBXO11	GSE92220	.	.	.	.
REG00014	FBXO21	GSE92220	.	.	.	.

REG00014	FBXO22	GSE92220	.	.	.	.
REG00014	TSPAN17	GSE92220	.	.	.	.
REG00014	FBXO25	GSE92220	.	.	.	.
REG00014	FBXL3	GSE92220	.	.	.	.
REG00014	FBXL4	GSE92220	.	.	.	.
REG00014	FBXL5	GSE92220	.	.	.	.
REG00014	FBXL6	GSE92220	.	.	.	.
REG00014	KDM2A	GSE92220	.	.	.	.
REG00014	FBXW11	GSE92220	.	.	.	.
REG00014	FBXW2	GSE92220	.	.	.	.
REG00014	FBXL12	GSE92220	.	.	.	.
REG00014	FBXW5	GSE92220	.	.	.	.
REG00014	FRRS1L	GSE92220	.	.	.	.
REG00014	FBXO18	GSE92220	.	.	.	.
REG00014	TMEM245	GSE92220	.	.	.	.
REG00014	TFPT	GSE92220	.	.	.	.
REG00014	RPL36	GSE92220	.	.	.	.
REG00014	MAML1	GSE92220	.	.	.	.
REG00014	MAL2	GSE92220	.	.	.	.
REG00014	FAM206A	GSE92220	.	.	.	.
REG00014	LAPTM4B	GSE92220	.	.	.	.
REG00014	CACFD1	GSE92220	.	.	.	.
REG00014	ADAMTS13	GSE92220	.	.	.	.
REG00014	KIAA0754	GSE92220	.	.	.	.
REG00014	MACF1	GSE92220	.	.	.	.
REG00014	AAAS	GSE92220	.	.	.	.
REG00014	AP1M1	GSE92220	.	.	.	.
REG00014	SPACA9	GSE92220	.	.	.	.
REG00014	KLF13	GSE92220	.	.	.	.
REG00014	IL22RA1	GSE92220	.	.	.	.
REG00014	MAK16	GSE92220	.	.	.	.
REG00014	GPR182	GSE92220	.	.	.	.
REG00014	DEGS1	GSE92220	.	.	.	.
REG00014	FOSL1	GSE92220	.	.	.	.
REG00014	RCE1	GSE92220	.	.	.	.
REG00014	NUTF2	GSE92220	.	.	.	.
REG00014	CTCF	GSE92220	.	.	.	.
REG00014	KMT2C	GSE92220	.	.	.	.
REG00014	RANBP9	GSE92220	.	.	.	.
REG00014	ADGRG3	GSE92220	.	.	.	.
REG00014	MAEA	GSE92220	.	.	.	.
REG00014	CDH23	GSE92220	.	.	.	.
REG00014	UBL5	GSE92220	.	.	.	.
REG00014	HERPUD1	GSE92220	.	.	.	.
REG00014	FAM3A	GSE92220	.	.	.	.
REG00014	FSD1L	GSE92220	.	.	.	.
REG00014	CYFIP1	GSE92220	.	.	.	.
REG00014	RELT	GSE92220	.	.	.	.
REG00014	CA5A	GSE92220	.	.	.	.
REG00014	IGDCC4	GSE92220	.	.	.	.

REG00014	SOST	GSE92220	.	.	.	.
REG00014	RNF24	GSE92220	.	.	.	.
REG00014	MRS2	GSE92220	.	.	.	.
REG00014	BCL2L12	GSE92220	.	.	.	.
REG00014	ERP29	GSE92220	.	.	.	.
REG00014	CHRNA10	GSE92220	.	.	.	.
REG00014	SLC2A8	GSE92220	.	.	.	.
REG00014	SLC4A1AP	GSE92220	.	.	.	.
REG00014	SLC12A5	GSE92220	.	.	.	.
REG00014	GFRA4	GSE92220	.	.	.	.
REG00014	ASPSCR1	GSE92220	.	.	.	.
REG00014	ADGRA3	GSE92220	.	.	.	.
REG00014	ADGRG6	GSE92220	.	.	.	.
REG00014	ZNF316	GSE92220	.	.	.	.
REG00014	SH3KBP1	GSE92220	.	.	.	.
REG00014	NUDT21	GSE92220	.	.	.	.
REG00014	CPSF6	GSE92220	.	.	.	.
REG00014	ABCG5	GSE92220	.	.	.	.
REG00014	ABCG8	GSE92220	.	.	.	.
REG00014	ITCH	GSE92220	.	.	.	.
REG00014	SGK2	GSE92220	.	.	.	.
REG00014	MXD4	GSE92220	.	.	.	.
REG00014	DMRTA2	GSE92220	.	.	.	.
REG00014	APOM	GSE92220	.	.	.	.
REG00014	BAG6	GSE92220	.	.	.	.
REG00014	GPANK1	GSE92220	.	.	.	.
REG00014	CCHCR1	GSE92220	.	.	.	.
REG00014	LY6G5B	GSE92220	.	.	.	.
REG00014	LY6G5C	GSE92220	.	.	.	.
REG00014	SAPCD1	GSE92220	.	.	.	.
REG00014	VWA7	GSE92220	.	.	.	.
REG00014	CACNA1G	GSE92220	.	.	.	.
REG00014	LSM2	GSE92220	.	.	.	.
REG00014	SLC44A4	GSE92220	.	.	.	.
REG00014	EGFL8	GSE92220	.	.	.	.
REG00014	CACNA1H	GSE92220	.	.	.	.
REG00014	NELFE	GSE92220	.	.	.	.
REG00014	PDLIM2	GSE92220	.	.	.	.
REG00014	PRDM11	GSE92220	.	.	.	.
REG00014	PRDM15	GSE92220	.	.	.	.
REG00014	PRDM16	GSE92220	.	.	.	.
REG00014	PRDM14	GSE92220	.	.	.	.
REG00014	CDK2AP1	GSE92220	.	.	.	.
REG00014	PEG10	GSE92220	.	.	.	.
REG00014	OPN3	GSE92220	.	.	.	.
REG00014	MXD3	GSE92220	.	.	.	.
REG00014	MGA	GSE92220	.	.	.	.
REG00014	SMC2	GSE92220	.	.	.	.
REG00014	SMC4	GSE92220	.	.	.	.
REG00014	MEMO1	GSE92220	.	.	.	.

REG00014	CACNB3	GSE92220	.	.	.	.
REG00014	MRPL38	GSE92220	.	.	.	.
REG00014	MRPL37	GSE92220	.	.	.	.
REG00014	MRPL30	GSE92220	.	.	.	.
REG00014	MRPL24	GSE92220	.	.	.	.
REG00014	CACNB4	GSE92220	.	.	.	.
REG00014	MRPL11	GSE92220	.	.	.	.
REG00014	MRPS21	GSE92220	.	.	.	.
REG00014	MRPS16	GSE92220	.	.	.	.
REG00014	MRPS14	GSE92220	.	.	.	.
REG00014	CACNG1	GSE92220	.	.	.	.
REG00014	MRPS6	GSE92220	.	.	.	.
REG00014	MRPL19	GSE92220	.	.	.	.
REG00014	MRPL17	GSE92220	.	.	.	.
REG00014	MRPL15	GSE92220	.	.	.	.
REG00014	MRPL10	GSE92220	.	.	.	.
REG00014	CELF5	GSE92220	.	.	.	.
REG00014	CELF6	GSE92220	.	.	.	.
REG00014	REV1	GSE92220	.	.	.	.
REG00014	HDAC4	GSE92220	.	.	.	.
REG00014	HDAC6	GSE92220	.	.	.	.
REG00014	HDAC5	GSE92220	.	.	.	.
REG00014	CERS2	GSE92220	.	.	.	.
REG00014	NGB	GSE92220	.	.	.	.
REG00014	MYO15B	GSE92220	.	.	.	.
REG00014	RTN4	GSE92220	.	.	.	.
REG00014	ZSCAN31	GSE92220	.	.	.	.
REG00014	CNOT6	GSE92220	.	.	.	.
REG00014	CNOT7	GSE92220	.	.	.	.
REG00014	GLOD4	GSE92220	.	.	.	.
REG00014	NPRL3	GSE92220	.	.	.	.
REG00014	ARHGAP9	GSE92220	.	.	.	.
REG00014	MSRB1	GSE92220	.	.	.	.
REG00014	TPSG1	GSE92220	.	.	.	.
REG00014	PIGQ	GSE92220	.	.	.	.
REG00014	HN1L	GSE92220	.	.	.	.
REG00014	PRR35	GSE92220	.	.	.	.
REG00014	METTL26	GSE92220	.	.	.	.
REG00014	MCRIP2	GSE92220	.	.	.	.
REG00014	JMJD8	GSE92220	.	.	.	.
REG00014	METRN	GSE92220	.	.	.	.
REG00014	FAM173A	GSE92220	.	.	.	.
REG00014	CCDC78	GSE92220	.	.	.	.
REG00014	LMF1	GSE92220	.	.	.	.
REG00014	TMEM204	GSE92220	.	.	.	.
REG00014	FAHD1	GSE92220	.	.	.	.
REG00014	TSR3	GSE92220	.	.	.	.
REG00014	HAGHL	GSE92220	.	.	.	.
REG00014	NARFL	GSE92220	.	.	.	.
REG00014	PDIA2	GSE92220	.	.	.	.

REG00014	UNKL	GSE92220	.	.	.	.
REG00014	UBAP2	GSE92220	.	.	.	.
REG00014	OVOL3	GSE92220	.	.	.	.
REG00014	AVIL	GSE92220	.	.	.	.
REG00014	ARHGEF12	GSE92220	.	.	.	.
REG00014	ELAC2	GSE92220	.	.	.	.
REG00014	SUN2	GSE92220	.	.	.	.
REG00014	CIC	GSE92220	.	.	.	.
REG00014	TRIM55	GSE92220	.	.	.	.
REG00014	RBM14	GSE92220	.	.	.	.
REG00014	DERL3	GSE92220	.	.	.	.
REG00014	GUCD1	GSE92220	.	.	.	.
REG00014	CAD	GSE92220	.	.	.	.
REG00014	RSPH6A	GSE92220	.	.	.	.
REG00014	RAB18	GSE92220	.	.	.	.
REG00014	ATP5L	GSE92220	.	.	.	.
REG00014	MED15	GSE92220	.	.	.	.
REG00014	BRI3BP	GSE92220	.	.	.	.
REG00014	CERS1	GSE92220	.	.	.	.
REG00014	BRPF3	GSE92220	.	.	.	.
REG00014	CD2AP	GSE92220	.	.	.	.
REG00014	RAB23	GSE92220	.	.	.	.
REG00014	GAR1	GSE92220	.	.	.	.
REG00014	CDH24	GSE92220	.	.	.	.
REG00014	RASSF3	GSE92220	.	.	.	.
REG00014	MRPL4	GSE92220	.	.	.	.
REG00014	MRPL13	GSE92220	.	.	.	.
REG00014	MRPL14	GSE92220	.	.	.	.
REG00014	CIAO1	GSE92220	.	.	.	.
REG00014	IRF2BPL	GSE92220	.	.	.	.
REG00014	NLGN3	GSE92220	.	.	.	.
REG00014	NLGN2	GSE92220	.	.	.	.
REG00014	SHANK3	GSE92220	.	.	.	.
REG00014	SHANK2	GSE92220	.	.	.	.
REG00014	ACTC1	GSE92220	.	.	.	.
REG00014	LRRC1	GSE92220	.	.	.	.
REG00014	BRD7	GSE92220	.	.	.	.
REG00014	CRIPT	GSE92220	.	.	.	.
REG00014	C1QTNF1	GSE92220	.	.	.	.
REG00014	MKL1	GSE92220	.	.	.	.
REG00014	PLEKHA1	GSE92220	.	.	.	.
REG00014	PLEKHA2	GSE92220	.	.	.	.
REG00014	PLEKHA3	GSE92220	.	.	.	.
REG00014	PLEKHA4	GSE92220	.	.	.	.
REG00014	CALB1	GSE92220	.	.	.	.
REG00014	VPS29	GSE92220	.	.	.	.
REG00014	C1QTNF4	GSE92220	.	.	.	.
REG00014	HTRA2	GSE92220	.	.	.	.
REG00014	SEPTIN10	GSE92220	.	.	.	.
REG00014	ARID3B	GSE92220	.	.	.	.

REG00014	WDR13	GSE92220	.	.	.	.
REG00014	IRX3	GSE92220	.	.	.	.
REG00014	SCYL1	GSE92220	.	.	.	.
REG00014	GLMN	GSE92220	.	.	.	.
REG00014	PPP1R15A	GSE92220	.	.	.	.
REG00014	NHP2	GSE92220	.	.	.	.
REG00014	NOP10	GSE92220	.	.	.	.
REG00014	CDRT4	GSE92220	.	.	.	.
REG00014	GP6	GSE92220	.	.	.	.
REG00014	CDRT15	GSE92220	.	.	.	.
REG00014	CPVL	GSE92220	.	.	.	.
REG00014	ACTG1	GSE92220	.	.	.	.
REG00014	HIPK2	GSE92220	.	.	.	.
REG00014	KIF13B	GSE92220	.	.	.	.
REG00014	PTPN23	GSE92220	.	.	.	.
REG00014	SLC25A19	GSE92220	.	.	.	.
REG00014	CALD1	GSE92220	.	.	.	.
REG00014	DHX36	GSE92220	.	.	.	.
REG00014	CALM1	GSE92220	.	.	.	.
REG00014	SLC13A3	GSE92220	.	.	.	.
REG00014	RAPH1	GSE92220	.	.	.	.
REG00014	ALS2CR12	GSE92220	.	.	.	.
REG00014	FAM117B	GSE92220	.	.	.	.
REG00014	ICA1L	GSE92220	.	.	.	.
REG00014	CALM2	GSE92220	.	.	.	.
REG00014	LPIN2	GSE92220	.	.	.	.
REG00014	LPIN3	GSE92220	.	.	.	.
REG00014	ANAPC11	GSE92220	.	.	.	.
REG00014	H2AFY2	GSE92220	.	.	.	.
REG00014	H2AFJ	GSE92220	.	.	.	.
REG00014	GAB2	GSE92220	.	.	.	.
REG00014	NDRG2	GSE92220	.	.	.	.
REG00014	SLC39A11	GSE92220	.	.	.	.
REG00014	SLC26A11	GSE92220	.	.	.	.
REG00014	SLC26A6	GSE92220	.	.	.	.
REG00014	MRPL16	GSE92220	.	.	.	.
REG00014	MRPL20	GSE92220	.	.	.	.
REG00014	MRPL21	GSE92220	.	.	.	.
REG00014	MRPL27	GSE92220	.	.	.	.
REG00014	MRPL28	GSE92220	.	.	.	.
REG00014	MRPL33	GSE92220	.	.	.	.
REG00014	MRPL34	GSE92220	.	.	.	.
REG00014	MRPL35	GSE92220	.	.	.	.
REG00014	CALM3	GSE92220	.	.	.	.
REG00014	MRPL36	GSE92220	.	.	.	.
REG00014	MRPL41	GSE92220	.	.	.	.
REG00014	MRPL42	GSE92220	.	.	.	.
REG00014	MRPS2	GSE92220	.	.	.	.
REG00014	IMP3	GSE92220	.	.	.	.
REG00014	MRPS5	GSE92220	.	.	.	.

REG00014	MRPS7	GSE92220	.	.	.	.
REG00014	ACTG2	GSE92220	.	.	.	.
REG00014	MRPS9	GSE92220	.	.	.	.
REG00014	MRPS10	GSE92220	.	.	.	.
REG00014	MRPS15	GSE92220	.	.	.	.
REG00014	MRPS22	GSE92220	.	.	.	.
REG00014	MRPS23	GSE92220	.	.	.	.
REG00014	MRPS24	GSE92220	.	.	.	.
REG00014	MRPS25	GSE92220	.	.	.	.
REG00014	MRPS28	GSE92220	.	.	.	.
REG00014	MRPS18B	GSE92220	.	.	.	.
REG00014	MRPL43	GSE92220	.	.	.	.
REG00014	MIOX	GSE92220	.	.	.	.
REG00014	SPAG9	GSE92220	.	.	.	.
REG00014	SP5	GSE92220	.	.	.	.
REG00014	KLF15	GSE92220	.	.	.	.
REG00014	NPC2	GSE92220	.	.	.	.
REG00014	RNF213	GSE92220	.	.	.	.
REG00014	WNK1	GSE92220	.	.	.	.
REG00014	WNK2	GSE92220	.	.	.	.
REG00014	WNK4	GSE92220	.	.	.	.
REG00014	CALR	GSE92220	.	.	.	.
REG00014	IKBKE	GSE92220	.	.	.	.
REG00014	LRP10	GSE92220	.	.	.	.
REG00014	STOML2	GSE92220	.	.	.	.
REG00014	BET1	GSE92220	.	.	.	.
REG00014	SORBS1	GSE92220	.	.	.	.
REG00014	KIF13A	GSE92220	.	.	.	.
REG00014	STK33	GSE92220	.	.	.	.
REG00014	HN1	GSE92220	.	.	.	.
REG00014	RHBG	GSE92220	.	.	.	.
REG00014	ENTPD4	GSE92220	.	.	.	.
REG00014	MCF2L	GSE92220	.	.	.	.
REG00014	VPS16	GSE92220	.	.	.	.
REG00014	CAMK1	GSE92220	.	.	.	.
REG00014	NET1	GSE92220	.	.	.	.
REG00014	IMMP2L	GSE92220	.	.	.	.
REG00014	TINAG	GSE92220	.	.	.	.
REG00014	RGS20	GSE92220	.	.	.	.
REG00014	ECI2	GSE92220	.	.	.	.
REG00014	DMTF1	GSE92220	.	.	.	.
REG00014	AMN	GSE92220	.	.	.	.
REG00014	TINCR	GSE92220	.	.	.	.
REG00014	CAMK2B	GSE92220	.	.	.	.
REG00014	CAMK2D	GSE92220	.	.	.	.
REG00014	CDCA4	GSE92220	.	.	.	.
REG00014	CDCA5	GSE92220	.	.	.	.
REG00014	CDCA7	GSE92220	.	.	.	.
REG00014	CRELD1	GSE92220	.	.	.	.
REG00014	PDAP1	GSE92220	.	.	.	.

REG00014	IPPK	GSE92220	.	.	.	.
REG00014	RNF26	GSE92220	.	.	.	.
REG00014	LETM2	GSE92220	.	.	.	.
REG00014	PPIG	GSE92220	.	.	.	.
REG00014	PPIH	GSE92220	.	.	.	.
REG00014	PARVB	GSE92220	.	.	.	.
REG00014	EGLN2	GSE92220	.	.	.	.
REG00014	MMEL1	GSE92220	.	.	.	.
REG00014	SLC38A4	GSE92220	.	.	.	.
REG00014	HDGFRP2	GSE92220	.	.	.	.
REG00014	SPATA2	GSE92220	.	.	.	.
REG00014	SPATA1	GSE92220	.	.	.	.
REG00014	POFUT2	GSE92220	.	.	.	.
REG00014	F11R	GSE92220	.	.	.	.
REG00014	CAMKK2	GSE92220	.	.	.	.
REG00014	CAMLG	GSE92220	.	.	.	.
REG00014	CANX	GSE92220	.	.	.	.
REG00014	CAPN1	GSE92220	.	.	.	.
REG00014	CAPN10	GSE92220	.	.	.	.
REG00014	CAPN11	GSE92220	.	.	.	.
REG00014	CAPN2	GSE92220	.	.	.	.
REG00014	CAPN3	GSE92220	.	.	.	.
REG00014	CAPNS1	GSE92220	.	.	.	.
REG00014	CAPN7	GSE92220	.	.	.	.
REG00014	UCK1	GSE92220	.	.	.	.
REG00014	UBXN4	GSE92220	.	.	.	.
REG00014	APOL5	GSE92220	.	.	.	.
REG00014	CAPS	GSE92220	.	.	.	.
REG00014	APOL6	GSE92220	.	.	.	.
REG00014	INTS6	GSE92220	.	.	.	.
REG00014	CAPZA1	GSE92220	.	.	.	.
REG00014	GTPBP3	GSE92220	.	.	.	.
REG00014	DNAJA2	GSE92220	.	.	.	.
REG00014	DNAJB4	GSE92220	.	.	.	.
REG00014	DNAJB6	GSE92220	.	.	.	.
REG00014	DNAJB11	GSE92220	.	.	.	.
REG00014	DNAJB12	GSE92220	.	.	.	.
REG00014	DPP7	GSE92220	.	.	.	.
REG00014	CAPZA2	GSE92220	.	.	.	.
REG00014	CAPZB	GSE92220	.	.	.	.
REG00014	TAS2R3	GSE92220	.	.	.	.
REG00014	SHPK	GSE92220	.	.	.	.
REG00014	TSGA10	GSE92220	.	.	.	.
REG00014	UBXN6	GSE92220	.	.	.	.
REG00014	SIRT1	GSE92220	.	.	.	.
REG00014	CARS	GSE92220	.	.	.	.
REG00014	SIRT5	GSE92220	.	.	.	.
REG00014	SIRT6	GSE92220	.	.	.	.
REG00014	SIRT7	GSE92220	.	.	.	.
REG00014	PIGS	GSE92220	.	.	.	.

REG00014	PIGT	GSE92220	.	.	.	.
REG00014	PPP1R1C	GSE92220	.	.	.	.
REG00014	PPP1R3E	GSE92220	.	.	.	.
REG00014	PPP1R3F	GSE92220	.	.	.	.
REG00014	PPP1R3G	GSE92220	.	.	.	.
REG00014	PPP1R9A	GSE92220	.	.	.	.
REG00014	PPP1R12C	GSE92220	.	.	.	.
REG00014	RCC1L	GSE92220	.	.	.	.
REG00014	PPP1R15B	GSE92220	.	.	.	.
REG00014	PUM1	GSE92220	.	.	.	.
REG00014	PUM2	GSE92220	.	.	.	.
REG00014	RBM15	GSE92220	.	.	.	.
REG00014	SYT13	GSE92220	.	.	.	.
REG00014	LRRC3	GSE92220	.	.	.	.
REG00014	SNX5	GSE92220	.	.	.	.
REG00014	SNX6	GSE92220	.	.	.	.
REG00014	SNX7	GSE92220	.	.	.	.
REG00014	SNX9	GSE92220	.	.	.	.
REG00014	SNX11	GSE92220	.	.	.	.
REG00014	SNX12	GSE92220	.	.	.	.
REG00014	SNX14	GSE92220	.	.	.	.
REG00014	SNX15	GSE92220	.	.	.	.
REG00014	PAPOLA	GSE92220	.	.	.	.
REG00014	ZFP91	GSE92220	.	.	.	.
REG00014	LUZP1	GSE92220	.	.	.	.
REG00014	ASAP3	GSE92220	.	.	.	.
REG00014	CASP10	GSE92220	.	.	.	.
REG00014	CASP3	GSE92220	.	.	.	.
REG00014	CASP4	GSE92220	.	.	.	.
REG00014	OR2AE1	GSE92220	.	.	.	.
REG00014	CASP8	GSE92220	.	.	.	.
REG00014	CAST	GSE92220	.	.	.	.
REG00014	CAT	GSE92220	.	.	.	.
REG00014	CBFA2T2	GSE92220	.	.	.	.
REG00014	CBFB	GSE92220	.	.	.	.
REG00014	SERPINA6	GSE92220	.	.	.	.
REG00014	CBL	GSE92220	.	.	.	.
REG00014	GOLIM4	GSE92220	.	.	.	.
REG00014	TGOLN2	GSE92220	.	.	.	.
REG00014	GOLPH3	GSE92220	.	.	.	.
REG00014	ACBD3	GSE92220	.	.	.	.
REG00014	MBTPS1	GSE92220	.	.	.	.
REG00014	P2RX2	GSE92220	.	.	.	.
REG00014	SERPINH1	GSE92220	.	.	.	.
REG00014	MANF	GSE92220	.	.	.	.
REG00014	PTDSS2	GSE92220	.	.	.	.
REG00014	GPHN	GSE92220	.	.	.	.
REG00014	DYNLRB1	GSE92220	.	.	.	.
REG00014	DNAJC6	GSE92220	.	.	.	.
REG00014	DNAJC8	GSE92220	.	.	.	.

REG00014	ALDH1A2	GSE92220	.	.	.	.
REG00014	TSSK3	GSE92220	.	.	.	.
REG00014	PRSS27	GSE92220	.	.	.	.
REG00014	DYNLL1	GSE92220	.	.	.	.
REG00014	ADAM32	GSE92220	.	.	.	.
REG00014	NCKIPSD	GSE92220	.	.	.	.
REG00014	TMX1	GSE92220	.	.	.	.
REG00014	IL23A	GSE92220	.	.	.	.
REG00014	ZNF331	GSE92220	.	.	.	.
REG00014	ANKH	GSE92220	.	.	.	.
REG00014	CBS	GSE92220	.	.	.	.
REG00014	LENG8	GSE92220	.	.	.	.
REG00014	LENG1	GSE92220	.	.	.	.
REG00014	BTBD2	GSE92220	.	.	.	.
REG00014	MBOAT7	GSE92220	.	.	.	.
REG00014	TSEN34	GSE92220	.	.	.	.
REG00014	PUS1	GSE92220	.	.	.	.
REG00014	PICALM	GSE92220	.	.	.	.
REG00014	ARHGEF16	GSE92220	.	.	.	.
REG00014	XYLT2	GSE92220	.	.	.	.
REG00014	CBX2	GSE92220	.	.	.	.
REG00014	LPAR6	GSE92220	.	.	.	.
REG00014	KCNIP2	GSE92220	.	.	.	.
REG00014	LBX2	GSE92220	.	.	.	.
REG00014	CBX3	GSE92220	.	.	.	.
REG00014	TULP4	GSE92220	.	.	.	.
REG00014	EBNA1BP2	GSE92220	.	.	.	.
REG00014	SPRY4	GSE92220	.	.	.	.
REG00014	CBX4	GSE92220	.	.	.	.
REG00014	ARID4B	GSE92220	.	.	.	.
REG00014	CHCHD10	GSE92220	.	.	.	.
REG00014	CBX6	GSE92220	.	.	.	.
REG00014	ILKAP	GSE92220	.	.	.	.
REG00014	SH3BGRL2	GSE92220	.	.	.	.
REG00014	SH3BGRL3	GSE92220	.	.	.	.
REG00014	CABYR	GSE92220	.	.	.	.
REG00014	RB1CC1	GSE92220	.	.	.	.
REG00014	MYO3B	GSE92220	.	.	.	.
REG00014	EPPK1	GSE92220	.	.	.	.
REG00014	REPS1	GSE92220	.	.	.	.
REG00014	TRIM8	GSE92220	.	.	.	.
REG00014	SYTL1	GSE92220	.	.	.	.
REG00014	LRRC4	GSE92220	.	.	.	.
REG00014	SS18L1	GSE92220	.	.	.	.
REG00014	SS18L2	GSE92220	.	.	.	.
REG00014	SLC12A8	GSE92220	.	.	.	.
REG00014	HPS3	GSE92220	.	.	.	.
REG00014	HAMP	GSE92220	.	.	.	.
REG00014	FBXO30	GSE92220	.	.	.	.
REG00014	LAMTOR3	GSE92220	.	.	.	.

REG00014	ARHGEF7	GSE92220	.	.	.	.
REG00014	NOLC1	GSE92220	.	.	.	.
REG00014	NBAS	GSE92220	.	.	.	.
REG00014	B3GNT2	GSE92220	.	.	.	.
REG00014	TCERG1	GSE92220	.	.	.	.
REG00014	TLR9	GSE92220	.	.	.	.
REG00014	TAS1R3	GSE92220	.	.	.	.
REG00014	UGGT1	GSE92220	.	.	.	.
REG00014	UGGT2	GSE92220	.	.	.	.
REG00014	MTPN	GSE92220	.	.	.	.
REG00014	ALG9	GSE92220	.	.	.	.
REG00014	PHIP	GSE92220	.	.	.	.
REG00014	SSBP3	GSE92220	.	.	.	.
REG00014	SSBP4	GSE92220	.	.	.	.
REG00014	TNKS2	GSE92220	.	.	.	.
REG00014	PHC3	GSE92220	.	.	.	.
REG00014	B3GNT4	GSE92220	.	.	.	.
REG00014	CCK	GSE92220	.	.	.	.
REG00014	UBE2Q1	GSE92220	.	.	.	.
REG00014	CCKAR	GSE92220	.	.	.	.
REG00014	PPIL4	GSE92220	.	.	.	.
REG00014	RHOF	GSE92220	.	.	.	.
REG00014	PAQR8	GSE92220	.	.	.	.
REG00014	ZASP	GSE92220	.	.	.	.
REG00014	ANAPC5	GSE92220	.	.	.	.
REG00014	LRPPRC	GSE92220	.	.	.	.
REG00014	MAP1S	GSE92220	.	.	.	.
REG00014	HEBP2	GSE92220	.	.	.	.
REG00014	APCDD1	GSE92220	.	.	.	.
REG00014	STRN3	GSE92220	.	.	.	.
REG00014	STRN4	GSE92220	.	.	.	.
REG00014	KRIT1	GSE92220	.	.	.	.
REG00014	HID1	GSE92220	.	.	.	.
REG00014	DSTN	GSE92220	.	.	.	.
REG00014	NAPB	GSE92220	.	.	.	.
REG00014	MYL9	GSE92220	.	.	.	.
REG00014	SDCBP2	GSE92220	.	.	.	.
REG00014	ADRM1	GSE92220	.	.	.	.
REG00014	OSBPL2	GSE92220	.	.	.	.
REG00014	CRNKL1	GSE92220	.	.	.	.
REG00014	POLR3F	GSE92220	.	.	.	.
REG00014	TGIF2	GSE92220	.	.	.	.
REG00014	ADNP	GSE92220	.	.	.	.
REG00014	OGFR	GSE92220	.	.	.	.
REG00014	CPXM1	GSE92220	.	.	.	.
REG00014	ARFGEF1	GSE92220	.	.	.	.
REG00014	METAP1	GSE92220	.	.	.	.
REG00014	CCNB1	GSE92220	.	.	.	.
REG00014	PABPC1L	GSE92220	.	.	.	.
REG00014	MANBAL	GSE92220	.	.	.	.

REG00014	CCNB2	GSE92220	.	.	.	.
REG00014	GCNA	GSE92220	.	.	.	.
REG00014	ZNF335	GSE92220	.	.	.	.
REG00014	ZNF337	GSE92220	.	.	.	.
REG00014	FAM207A	GSE92220	.	.	.	.
REG00014	ACSS2	GSE92220	.	.	.	.
REG00014	RBM38	GSE92220	.	.	.	.
REG00014	CCND1	GSE92220	.	.	.	.
REG00014	RASD1	GSE92220	.	.	.	.
REG00014	ELOVL6	GSE92220	.	.	.	.
REG00014	BSCL2	GSE92220	.	.	.	.
REG00014	KMT2B	GSE92220	.	.	.	.
REG00014	ERBIN	GSE92220	.	.	.	.
REG00014	HPS4	GSE92220	.	.	.	.
REG00014	RBP5	GSE92220	.	.	.	.
REG00014	CCND3	GSE92220	.	.	.	.
REG00014	NECAB3	GSE92220	.	.	.	.
REG00014	ARFGAP1	GSE92220	.	.	.	.
REG00014	ARFGEF2	GSE92220	.	.	.	.
REG00014	BTBD3	GSE92220	.	.	.	.
REG00014	TP53TG5	GSE92220	.	.	.	.
REG00014	GID8	GSE92220	.	.	.	.
REG00014	TASP1	GSE92220	.	.	.	.
REG00014	PRPF6	GSE92220	.	.	.	.
REG00014	DHX35	GSE92220	.	.	.	.
REG00014	SMOX	GSE92220	.	.	.	.
REG00014	RBCK1	GSE92220	.	.	.	.
REG00014	KIZ	GSE92220	.	.	.	.
REG00014	MRGBP	GSE92220	.	.	.	.
REG00014	YTHDF1	GSE92220	.	.	.	.
REG00014	ABHD12	GSE92220	.	.	.	.
REG00014	KIF16B	GSE92220	.	.	.	.
REG00014	CCNDBP1	GSE92220	.	.	.	.
REG00014	C20orf24	GSE92220	.	.	.	.
REG00014	C20orf27	GSE92220	.	.	.	.
REG00014	SPEF1	GSE92220	.	.	.	.
REG00014	AP5S1	GSE92220	.	.	.	.
REG00014	TMEM230	GSE92220	.	.	.	.
REG00014	CASS4	GSE92220	.	.	.	.
REG00014	CDK5RAP1	GSE92220	.	.	.	.
REG00014	PCMTD2	GSE92220	.	.	.	.
REG00014	AAR2	GSE92220	.	.	.	.
REG00014	LSM14B	GSE92220	.	.	.	.
REG00014	CCNE1	GSE92220	.	.	.	.
REG00014	UQCC1	GSE92220	.	.	.	.
REG00014	TMEM74B	GSE92220	.	.	.	.
REG00014	PANK2	GSE92220	.	.	.	.
REG00014	ESF1	GSE92220	.	.	.	.
REG00014	CCNE2	GSE92220	.	.	.	.
REG00014	IFT52	GSE92220	.	.	.	.

REG00014	L3MBTL1	GSE92220	.	.	.	.
REG00014	NAA20	GSE92220	.	.	.	.
REG00014	NCOA5	GSE92220	.	.	.	.
REG00014	NFS1	GSE92220	.	.	.	.
REG00014	NSFL1C	GSE92220	.	.	.	.
REG00014	PLCB1	GSE92220	.	.	.	.
REG00014	CCNG1	GSE92220	.	.	.	.
REG00014	RALY	GSE92220	.	.	.	.
REG00014	RBM39	GSE92220	.	.	.	.
REG00014	STMN3	GSE92220	.	.	.	.
REG00014	ERGIC3	GSE92220	.	.	.	.
REG00014	CCNG2	GSE92220	.	.	.	.
REG00014	SLC2A4RG	GSE92220	.	.	.	.
REG00014	NELFCD	GSE92220	.	.	.	.
REG00014	NCOA6	GSE92220	.	.	.	.
REG00014	UCKL1	GSE92220	.	.	.	.
REG00014	CCNH	GSE92220	.	.	.	.
REG00014	ZFP64	GSE92220	.	.	.	.
REG00014	STX18	GSE92220	.	.	.	.
REG00014	ZGPAT	GSE92220	.	.	.	.
REG00014	CCNI	GSE92220	.	.	.	.
REG00014	CCNK	GSE92220	.	.	.	.
REG00014	CBLC	GSE92220	.	.	.	.
REG00014	CBX8	GSE92220	.	.	.	.
REG00014	TSG101	GSE92220	.	.	.	.
REG00014	SASH3	GSE92220	.	.	.	.
REG00014	ZNF296	GSE92220	.	.	.	.
REG00014	CUL9	GSE92220	.	.	.	.
REG00014	GLRX3	GSE92220	.	.	.	.
REG00014	SERPINA10	GSE92220	.	.	.	.
REG00014	SELENON	GSE92220	.	.	.	.
REG00014	SERPINA3	GSE92220	.	.	.	.
REG00014	CCNT2	GSE92220	.	.	.	.
REG00014	MPLKIP	GSE92220	.	.	.	.
REG00014	ASB1	GSE92220	.	.	.	.
REG00014	ASB3	GSE92220	.	.	.	.
REG00014	CASD1	GSE92220	.	.	.	.
REG00014	MOB1A	GSE92220	.	.	.	.
REG00014	CADPS2	GSE92220	.	.	.	.
REG00014	RNF31	GSE92220	.	.	.	.
REG00014	ORMDL1	GSE92220	.	.	.	.
REG00014	ORMDL2	GSE92220	.	.	.	.
REG00014	ORMDL3	GSE92220	.	.	.	.
REG00014	ANGPTL4	GSE92220	.	.	.	.
REG00014	UNC50	GSE92220	.	.	.	.
REG00014	PRADC1	GSE92220	.	.	.	.
REG00014	FRAT2	GSE92220	.	.	.	.
REG00014	PTGES3	GSE92220	.	.	.	.
REG00014	CCR4	GSE92220	.	.	.	.
REG00014	PARD3	GSE92220	.	.	.	.

REG00014	ZNF444	GSE92220	.	.	.	.
REG00014	SLC46A2	GSE92220	.	.	.	.
REG00014	PAK4	GSE92220	.	.	.	.
REG00014	CRTC1	GSE92220	.	.	.	.
REG00014	MLLT10	GSE92220	.	.	.	.
REG00014	GGA2	GSE92220	.	.	.	.
REG00014	GLRX2	GSE92220	.	.	.	.
REG00014	CCR6	GSE92220	.	.	.	.
REG00014	SFXN5	GSE92220	.	.	.	.
REG00014	OCIAD1	GSE92220	.	.	.	.
REG00014	RAB33B	GSE92220	.	.	.	.
REG00014	DUSP22	GSE92220	.	.	.	.
REG00014	SFXN1	GSE92220	.	.	.	.
REG00014	SFXN2	GSE92220	.	.	.	.
REG00014	SFXN3	GSE92220	.	.	.	.
REG00014	SFXN4	GSE92220	.	.	.	.
REG00014	PGRMC2	GSE92220	.	.	.	.
REG00014	PGRMC1	GSE92220	.	.	.	.
REG00014	ACSS1	GSE92220	.	.	.	.
REG00014	ZBTB46	GSE92220	.	.	.	.
REG00014	PHF20	GSE92220	.	.	.	.
REG00014	ACTL7A	GSE92220	.	.	.	.
REG00014	FAM209A	GSE92220	.	.	.	.
REG00014	FAM210B	GSE92220	.	.	.	.
REG00014	TP53INP2	GSE92220	.	.	.	.
REG00014	OSER1	GSE92220	.	.	.	.
REG00014	NOL4L	GSE92220	.	.	.	.
REG00014	ACKR4	GSE92220	.	.	.	.
REG00014	DDRGK1	GSE92220	.	.	.	.
REG00014	SOGA1	GSE92220	.	.	.	.
REG00014	TLDC2	GSE92220	.	.	.	.
REG00014	TTPAL	GSE92220	.	.	.	.
REG00014	OCSTAMP	GSE92220	.	.	.	.
REG00014	PDRG1	GSE92220	.	.	.	.
REG00014	FAM83C	GSE92220	.	.	.	.
REG00014	EMILIN3	GSE92220	.	.	.	.
REG00014	MROH8	GSE92220	.	.	.	.
REG00014	ACTL10	GSE92220	.	.	.	.
REG00014	SAMD10	GSE92220	.	.	.	.
REG00014	CCS	GSE92220	.	.	.	.
REG00014	SRXN1	GSE92220	.	.	.	.
REG00014	TBC1D20	GSE92220	.	.	.	.
REG00014	FITM2	GSE92220	.	.	.	.
REG00014	C20orf144	GSE92220	.	.	.	.
REG00014	NANP	GSE92220	.	.	.	.
REG00014	PPDPF	GSE92220	.	.	.	.
REG00014	CABLES2	GSE92220	.	.	.	.
REG00014	MCM8	GSE92220	.	.	.	.
REG00014	CRLS1	GSE92220	.	.	.	.
REG00014	CCT2	GSE92220	.	.	.	.

REG00014	SNX21	GSE92220	.	.	.	.
REG00014	NEURL2	GSE92220	.	.	.	.
REG00014	ZSWIM3	GSE92220	.	.	.	.
REG00014	SPATA25	GSE92220	.	.	.	.
REG00014	CCT3	GSE92220	.	.	.	.
REG00014	DNTTIP1	GSE92220	.	.	.	.
REG00014	DSN1	GSE92220	.	.	.	.
REG00014	C20orf173	GSE92220	.	.	.	.
REG00014	CCT4	GSE92220	.	.	.	.
REG00014	FAM217B	GSE92220	.	.	.	.
REG00014	CHMP4B	GSE92220	.	.	.	.
REG00014	BPIFB2	GSE92220	.	.	.	.
REG00014	CCT5	GSE92220	.	.	.	.
REG00014	TRPC4AP	GSE92220	.	.	.	.
REG00014	SLC17A9	GSE92220	.	.	.	.
REG00014	TP53RK	GSE92220	.	.	.	.
REG00014	GTSF1L	GSE92220	.	.	.	.
REG00014	CCT6A	GSE92220	.	.	.	.
REG00014	PCIF1	GSE92220	.	.	.	.
REG00014	RALGAPA2	GSE92220	.	.	.	.
REG00014	LRRN4	GSE92220	.	.	.	.
REG00014	RPRD1B	GSE92220	.	.	.	.
REG00014	ISM1	GSE92220	.	.	.	.
REG00014	CCT7	GSE92220	.	.	.	.
REG00014	COMMD7	GSE92220	.	.	.	.
REG00014	C20orf96	GSE92220	.	.	.	.
REG00014	TRIB3	GSE92220	.	.	.	.
REG00014	NRSN2	GSE92220	.	.	.	.
REG00014	CCT8	GSE92220	.	.	.	.
REG00014	ZCCHC3	GSE92220	.	.	.	.
REG00014	COX4I2	GSE92220	.	.	.	.
REG00014	DNAJC5	GSE92220	.	.	.	.
REG00014	MTG2	GSE92220	.	.	.	.
REG00014	MYLK2	GSE92220	.	.	.	.
REG00014	NPEPL1	GSE92220	.	.	.	.
REG00014	PARD6B	GSE92220	.	.	.	.
REG00014	STK35	GSE92220	.	.	.	.
REG00014	YAP1	GSE92220	.	.	.	.
REG00014	CDC42EP2	GSE92220	.	.	.	.
REG00014	TRAP1	GSE92220	.	.	.	.
REG00014	WNT16	GSE92220	.	.	.	.
REG00014	PNPLA6	GSE92220	.	.	.	.
REG00014	SLC16A8	GSE92220	.	.	.	.
REG00014	SYAP1	GSE92220	.	.	.	.
REG00014	TRIM4	GSE92220	.	.	.	.
REG00014	TRIM5	GSE92220	.	.	.	.
REG00014	TRIM11	GSE92220	.	.	.	.
REG00014	TRIM14	GSE92220	.	.	.	.
REG00014	TRIM15	GSE92220	.	.	.	.
REG00014	TRIM35	GSE92220	.	.	.	.

REG00014	NFU1	GSE92220	.	.	.	.
REG00014	TRIM33	GSE92220	.	.	.	.
REG00014	MIDN	GSE92220	.	.	.	.
REG00014	ACTN1	GSE92220	.	.	.	.
REG00014	CD151	GSE92220	.	.	.	.
REG00014	LENG9	GSE92220	.	.	.	.
REG00014	SNX22	GSE92220	.	.	.	.
REG00014	CD164	GSE92220	.	.	.	.
REG00014	ARHGAP12	GSE92220	.	.	.	.
REG00014	PARK7	GSE92220	.	.	.	.
REG00014	OSBPL3	GSE92220	.	.	.	.
REG00014	CAPNS2	GSE92220	.	.	.	.
REG00014	FAM8A1	GSE92220	.	.	.	.
REG00014	PPM1L	GSE92220	.	.	.	.
REG00014	TRIM28	GSE92220	.	.	.	.
REG00014	NSUN5	GSE92220	.	.	.	.
REG00014	OSBPL9	GSE92220	.	.	.	.
REG00014	OSBPL7	GSE92220	.	.	.	.
REG00014	CARD9	GSE92220	.	.	.	.
REG00014	ZNF346	GSE92220	.	.	.	.
REG00014	IL17F	GSE92220	.	.	.	.
REG00014	WBSCR22	GSE92220	.	.	.	.
REG00014	EFHC1	GSE92220	.	.	.	.
REG00014	NLRC4	GSE92220	.	.	.	.
REG00014	CARD10	GSE92220	.	.	.	.
REG00014	AZIN1	GSE92220	.	.	.	.
REG00014	HM13	GSE92220	.	.	.	.
REG00014	SLC4A11	GSE92220	.	.	.	.
REG00014	DHRS1	GSE92220	.	.	.	.
REG00014	CD24	GSE92220	.	.	.	.
REG00014	FARP2	GSE92220	.	.	.	.
REG00014	WBP11	GSE92220	.	.	.	.
REG00014	STRBP	GSE92220	.	.	.	.
REG00014	PRPF40A	GSE92220	.	.	.	.
REG00014	SLC45A2	GSE92220	.	.	.	.
REG00014	TOLLIP	GSE92220	.	.	.	.
REG00014	ADAP1	GSE92220	.	.	.	.
REG00014	UNC119B	GSE92220	.	.	.	.
REG00014	ATG4A	GSE92220	.	.	.	.
REG00014	PIDD1	GSE92220	.	.	.	.
REG00014	FAM160B2	GSE92220	.	.	.	.
REG00014	ZMIZ1	GSE92220	.	.	.	.
REG00014	PLSCR3	GSE92220	.	.	.	.
REG00014	PLSCR4	GSE92220	.	.	.	.
REG00014	BPIFB6	GSE92220	.	.	.	.
REG00014	CYGB	GSE92220	.	.	.	.
REG00014	FBXO31	GSE92220	.	.	.	.
REG00014	SEPTIN8	GSE92220	.	.	.	.
REG00014	KLF3	GSE92220	.	.	.	.
REG00014	LARGE2	GSE92220	.	.	.	.

REG00014	RAB17	GSE92220	.	.	.	.
REG00014	RAB14	GSE92220	.	.	.	.
REG00014	ACSL5	GSE92220	.	.	.	.
REG00014	CD2BP2	GSE92220	.	.	.	.
REG00014	ACTN4	GSE92220	.	.	.	.
REG00014	MRPS36	GSE92220	.	.	.	.
REG00014	MRPS18C	GSE92220	.	.	.	.
REG00014	KIF1B	GSE92220	.	.	.	.
REG00014	SRRM1	GSE92220	.	.	.	.
REG00014	SCARB1	GSE92220	.	.	.	.
REG00014	CXCL16	GSE92220	.	.	.	.
REG00014	SLMAP	GSE92220	.	.	.	.
REG00014	TRA2A	GSE92220	.	.	.	.
REG00014	CXCR6	GSE92220	.	.	.	.
REG00014	MRPL44	GSE92220	.	.	.	.
REG00014	MRPL45	GSE92220	.	.	.	.
REG00014	MRPL52	GSE92220	.	.	.	.
REG00014	BCL2L14	GSE92220	.	.	.	.
REG00014	CAPN13	GSE92220	.	.	.	.
REG00014	TMEM30A	GSE92220	.	.	.	.
REG00014	C19orf33	GSE92220	.	.	.	.
REG00014	METAP2	GSE92220	.	.	.	.
REG00014	ANP32E	GSE92220	.	.	.	.
REG00014	ANP32B	GSE92220	.	.	.	.
REG00014	ZBTB33	GSE92220	.	.	.	.
REG00014	OTUD7B	GSE92220	.	.	.	.
REG00014	MRPL53	GSE92220	.	.	.	.
REG00014	EIF4ENIF1	GSE92220	.	.	.	.
REG00014	YIF1A	GSE92220	.	.	.	.
REG00014	TUBGCP4	GSE92220	.	.	.	.
REG00014	CD320	GSE92220	.	.	.	.
REG00014	BCAP31	GSE92220	.	.	.	.
REG00014	MYL12A	GSE92220	.	.	.	.
REG00014	PAPD7	GSE92220	.	.	.	.
REG00014	FAM89B	GSE92220	.	.	.	.
REG00014	CALCRL	GSE92220	.	.	.	.
REG00014	AKTIP	GSE92220	.	.	.	.
REG00014	FBXW7	GSE92220	.	.	.	.
REG00014	SRSF10	GSE92220	.	.	.	.
REG00014	DDX31	GSE92220	.	.	.	.
REG00014	DHX33	GSE92220	.	.	.	.
REG00014	DHX34	GSE92220	.	.	.	.
REG00014	MCEE	GSE92220	.	.	.	.
REG00014	MEX3D	GSE92220	.	.	.	.
REG00014	TMEM189	GSE92220	.	.	.	.
REG00014	ZBTB11	GSE92220	.	.	.	.
REG00014	CIZ1	GSE92220	.	.	.	.
REG00014	MBNL2	GSE92220	.	.	.	.
REG00014	PRDX6	GSE92220	.	.	.	.
REG00014	ACAP3	GSE92220	.	.	.	.

REG00014	COPS7A	GSE92220	.	.	.	.
REG00014	ZNF544	GSE92220	.	.	.	.
REG00014	COPS7B	GSE92220	.	.	.	.
REG00014	GRIN3B	GSE92220	.	.	.	.
REG00014	CD247	GSE92220	.	.	.	.
REG00014	CDC73	GSE92220	.	.	.	.
REG00014	EDEM3	GSE92220	.	.	.	.
REG00014	COLGALT2	GSE92220	.	.	.	.
REG00014	TSEN15	GSE92220	.	.	.	.
REG00014	SMG7	GSE92220	.	.	.	.
REG00014	ACTR1B	GSE92220	.	.	.	.
REG00014	UBE3C	GSE92220	.	.	.	.
REG00014	WWP2	GSE92220	.	.	.	.
REG00014	UBR5	GSE92220	.	.	.	.
REG00014	SMURF1	GSE92220	.	.	.	.
REG00014	SMURF2	GSE92220	.	.	.	.
REG00014	CD44	GSE92220	.	.	.	.
REG00014	COQ8A	GSE92220	.	.	.	.
REG00014	PPP1R27	GSE92220	.	.	.	.
REG00014	CHD5	GSE92220	.	.	.	.
REG00014	NOP14	GSE92220	.	.	.	.
REG00014	FAM193A	GSE92220	.	.	.	.
REG00014	TMEM11	GSE92220	.	.	.	.
REG00014	TSC22D1	GSE92220	.	.	.	.
REG00014	CDC123	GSE92220	.	.	.	.
REG00014	SEC22C	GSE92220	.	.	.	.
REG00014	RRP9	GSE92220	.	.	.	.
REG00014	IL32	GSE92220	.	.	.	.
REG00014	ATP6V1F	GSE92220	.	.	.	.
REG00014	SOCS6	GSE92220	.	.	.	.
REG00014	NREP	GSE92220	.	.	.	.
REG00014	TAOK2	GSE92220	.	.	.	.
REG00014	ZNF358	GSE92220	.	.	.	.
REG00014	LITAF	GSE92220	.	.	.	.
REG00014	WTAP	GSE92220	.	.	.	.
REG00014	MORF4L2	GSE92220	.	.	.	.
REG00014	CD5	GSE92220	.	.	.	.
REG00014	SOCS5	GSE92220	.	.	.	.
REG00014	RAPGEF2	GSE92220	.	.	.	.
REG00014	TRAM2	GSE92220	.	.	.	.
REG00014	KLF16	GSE92220	.	.	.	.
REG00014	USP6NL	GSE92220	.	.	.	.
REG00014	NOS1AP	GSE92220	.	.	.	.
REG00014	SART3	GSE92220	.	.	.	.
REG00014	RAPGEF5	GSE92220	.	.	.	.
REG00014	BCLAF1	GSE92220	.	.	.	.
REG00014	PHYHIP	GSE92220	.	.	.	.
REG00014	RNF40	GSE92220	.	.	.	.
REG00014	SPRN	GSE92220	.	.	.	.
REG00014	MRC2	GSE92220	.	.	.	.

REG00014	MFN2	GSE92220	.	.	.	.
REG00014	HELZ	GSE92220	.	.	.	.
REG00014	REC8	GSE92220	.	.	.	.
REG00014	AAED1	GSE92220	.	.	.	.
REG00014	HCN4	GSE92220	.	.	.	.
REG00014	PSMD14	GSE92220	.	.	.	.
REG00014	CD59	GSE92220	.	.	.	.
REG00014	CTDSPL	GSE92220	.	.	.	.
REG00014	TRIB1	GSE92220	.	.	.	.
REG00014	MFSD10	GSE92220	.	.	.	.
REG00014	RABEPK	GSE92220	.	.	.	.
REG00014	ACTR2	GSE92220	.	.	.	.
REG00014	SMNDC1	GSE92220	.	.	.	.
REG00014	BCKDK	GSE92220	.	.	.	.
REG00014	TNIP1	GSE92220	.	.	.	.
REG00014	GSTK1	GSE92220	.	.	.	.
REG00014	CPQ	GSE92220	.	.	.	.
REG00014	EMG1	GSE92220	.	.	.	.
REG00014	CTNNBIP1	GSE92220	.	.	.	.
REG00014	HAX1	GSE92220	.	.	.	.
REG00014	LRRC41	GSE92220	.	.	.	.
REG00014	SYNCRIP	GSE92220	.	.	.	.
REG00014	VAT1	GSE92220	.	.	.	.
REG00014	CD63	GSE92220	.	.	.	.
REG00014	CIB1	GSE92220	.	.	.	.
REG00014	AGAP1	GSE92220	.	.	.	.
REG00014	AGAP3	GSE92220	.	.	.	.
REG00014	ARAP1	GSE92220	.	.	.	.
REG00014	CD68	GSE92220	.	.	.	.
REG00014	CHERP	GSE92220	.	.	.	.
REG00014	HYOU1	GSE92220	.	.	.	.
REG00014	FEM1C	GSE92220	.	.	.	.
REG00014	FEM1A	GSE92220	.	.	.	.
REG00014	DGAT2	GSE92220	.	.	.	.
REG00014	RBM17	GSE92220	.	.	.	.
REG00014	PAIP1	GSE92220	.	.	.	.
REG00014	P3H4	GSE92220	.	.	.	.
REG00014	ERLIN1	GSE92220	.	.	.	.
REG00014	MYDGF	GSE92220	.	.	.	.
REG00014	FAM120C	GSE92220	.	.	.	.
REG00014	CD7	GSE92220	.	.	.	.
REG00014	STAMBP	GSE92220	.	.	.	.
REG00014	TXNIP	GSE92220	.	.	.	.
REG00014	GAS2L1	GSE92220	.	.	.	.
REG00014	YKT6	GSE92220	.	.	.	.
REG00014	CD226	GSE92220	.	.	.	.
REG00014	RRAGA	GSE92220	.	.	.	.
REG00014	ZMYND11	GSE92220	.	.	.	.
REG00014	ARPP19	GSE92220	.	.	.	.
REG00014	HSPH1	GSE92220	.	.	.	.

REG00014	CD74	GSE92220	.	.	.	.
REG00014	FRS3	GSE92220	.	.	.	.
REG00014	SRCAP	GSE92220	.	.	.	.
REG00014	BNIPL	GSE92220	.	.	.	.
REG00014	DHRS4	GSE92220	.	.	.	.
REG00014	LMAN2	GSE92220	.	.	.	.
REG00014	SUGT1	GSE92220	.	.	.	.
REG00014	SRSF8	GSE92220	.	.	.	.
REG00014	MORF4L1	GSE92220	.	.	.	.
REG00014	C11orf58	GSE92220	.	.	.	.
REG00014	CKAP4	GSE92220	.	.	.	.
REG00014	LRRC8D	GSE92220	.	.	.	.
REG00014	SEC61B	GSE92220	.	.	.	.
REG00014	OS9	GSE92220	.	.	.	.
REG00014	TMED2	GSE92220	.	.	.	.
REG00014	TMED10	GSE92220	.	.	.	.
REG00014	ACTR3	GSE92220	.	.	.	.
REG00014	PIAS4	GSE92220	.	.	.	.
REG00014	WWP1	GSE92220	.	.	.	.
REG00014	CNMD	GSE92220	.	.	.	.
REG00014	DUSP14	GSE92220	.	.	.	.
REG00014	TOPBP1	GSE92220	.	.	.	.
REG00014	TRIOBP	GSE92220	.	.	.	.
REG00014	HNRNPUL1	GSE92220	.	.	.	.
REG00014	FGFR1OP	GSE92220	.	.	.	.
REG00014	CD160	GSE92220	.	.	.	.
REG00014	CDC42EP1	GSE92220	.	.	.	.
REG00014	LSM6	GSE92220	.	.	.	.
REG00014	SEC23IP	GSE92220	.	.	.	.
REG00014	PRICKLE1	GSE92220	.	.	.	.
REG00014	RNF139	GSE92220	.	.	.	.
REG00014	PHF11	GSE92220	.	.	.	.
REG00014	COL21A1	GSE92220	.	.	.	.
REG00014	SLC16A10	GSE92220	.	.	.	.
REG00014	SNF8	GSE92220	.	.	.	.
REG00014	GPN1	GSE92220	.	.	.	.
REG00014	TUSC2	GSE92220	.	.	.	.
REG00014	EXOSC8	GSE92220	.	.	.	.
REG00014	TFB1M	GSE92220	.	.	.	.
REG00014	TMEM259	GSE92220	.	.	.	.
REG00014	CASC3	GSE92220	.	.	.	.
REG00014	PUF60	GSE92220	.	.	.	.
REG00014	NIPA1	GSE92220	.	.	.	.
REG00014	NIPA2	GSE92220	.	.	.	.
REG00014	NKD1	GSE92220	.	.	.	.
REG00014	RPL26L1	GSE92220	.	.	.	.
REG00014	SEC31A	GSE92220	.	.	.	.
REG00014	INPP5F	GSE92220	.	.	.	.
REG00014	MLXIP	GSE92220	.	.	.	.
REG00014	RPH3A	GSE92220	.	.	.	.

REG00014	SACM1L	GSE92220	.	.	.	.
REG00014	CD8A	GSE92220	.	.	.	.
REG00014	HABP4	GSE92220	.	.	.	.
REG00014	RAB3GAP1	GSE92220	.	.	.	.
REG00014	KDM4C	GSE92220	.	.	.	.
REG00014	ERC1	GSE92220	.	.	.	.
REG00014	TAB2	GSE92220	.	.	.	.
REG00014	GGA3	GSE92220	.	.	.	.
REG00014	EXOC6B	GSE92220	.	.	.	.
REG00014	CLASP1	GSE92220	.	.	.	.
REG00014	ARHGEF18	GSE92220	.	.	.	.
REG00014	LARS2	GSE92220	.	.	.	.
REG00014	EXOSC2	GSE92220	.	.	.	.
REG00014	DICER1	GSE92220	.	.	.	.
REG00014	ACVR1	GSE92220	.	.	.	.
REG00014	SUZ12	GSE92220	.	.	.	.
REG00014	ARHGAP45	GSE92220	.	.	.	.
REG00014	CDON	GSE92220	.	.	.	.
REG00014	HIF1AN	GSE92220	.	.	.	.
REG00014	SLC35C2	GSE92220	.	.	.	.
REG00014	RNF32	GSE92220	.	.	.	.
REG00014	SLC39A3	GSE92220	.	.	.	.
REG00014	SLC39A4	GSE92220	.	.	.	.
REG00014	CDAN1	GSE92220	.	.	.	.
REG00014	FTSJ3	GSE92220	.	.	.	.
REG00014	OPTN	GSE92220	.	.	.	.
REG00014	NT5C	GSE92220	.	.	.	.
REG00014	SNAPIN	GSE92220	.	.	.	.
REG00014	ARL2BP	GSE92220	.	.	.	.
REG00014	CDC42EP4	GSE92220	.	.	.	.
REG00014	SMUG1	GSE92220	.	.	.	.
REG00014	CARHSP1	GSE92220	.	.	.	.
REG00014	ACOT9	GSE92220	.	.	.	.
REG00014	RUSC1	GSE92220	.	.	.	.
REG00014	EDC4	GSE92220	.	.	.	.
REG00014	ARFIP2	GSE92220	.	.	.	.
REG00014	BCL2L13	GSE92220	.	.	.	.
REG00014	RAB3GAP2	GSE92220	.	.	.	.
REG00014	PRDX4	GSE92220	.	.	.	.
REG00014	TRUB2	GSE92220	.	.	.	.
REG00014	LOXL4	GSE92220	.	.	.	.
REG00014	NAGK	GSE92220	.	.	.	.
REG00014	HEBP1	GSE92220	.	.	.	.
REG00014	ASB6	GSE92220	.	.	.	.
REG00014	ASB7	GSE92220	.	.	.	.
REG00014	OLFM2	GSE92220	.	.	.	.
REG00014	CDC14B	GSE92220	.	.	.	.
REG00014	TIRAP	GSE92220	.	.	.	.
REG00014	NDUFA13	GSE92220	.	.	.	.
REG00014	ZNF354B	GSE92220	.	.	.	.

REG00014	CHSY1	GSE92220	.	.	.	.
REG00014	CDC16	GSE92220	.	.	.	.
REG00014	TMEM8A	GSE92220	.	.	.	.
REG00014	TMEM242	GSE92220	.	.	.	.
REG00014	BICD2	GSE92220	.	.	.	.
REG00014	DIP2A	GSE92220	.	.	.	.
REG00014	RAB11FIP3	GSE92220	.	.	.	.
REG00014	RAD54B	GSE92220	.	.	.	.
REG00014	CDC20	GSE92220	.	.	.	.
REG00014	ELMO2	GSE92220	.	.	.	.
REG00014	ACTR3B	GSE92220	.	.	.	.
REG00014	SPDEF	GSE92220	.	.	.	.
REG00014	LSM4	GSE92220	.	.	.	.
REG00014	CDC25B	GSE92220	.	.	.	.
REG00014	MOB4	GSE92220	.	.	.	.
REG00014	BRMS1	GSE92220	.	.	.	.
REG00014	RNF113B	GSE92220	.	.	.	.
REG00014	CDC25C	GSE92220	.	.	.	.
REG00014	PASK	GSE92220	.	.	.	.
REG00014	RRAS2	GSE92220	.	.	.	.
REG00014	ERI3	GSE92220	.	.	.	.
REG00014	ZFR	GSE92220	.	.	.	.
REG00014	PNRC1	GSE92220	.	.	.	.
REG00014	CDC27	GSE92220	.	.	.	.
REG00014	GHITM	GSE92220	.	.	.	.
REG00014	EXOSC1	GSE92220	.	.	.	.
REG00014	APOA5	GSE92220	.	.	.	.
REG00014	ELMO3	GSE92220	.	.	.	.
REG00014	CDK11B	GSE92220	.	.	.	.
REG00014	TMED1	GSE92220	.	.	.	.
REG00014	PRKD2	GSE92220	.	.	.	.
REG00014	DAB2IP	GSE92220	.	.	.	.
REG00014	RRM2B	GSE92220	.	.	.	.
REG00014	BRF2	GSE92220	.	.	.	.
REG00014	ACVR2A	GSE92220	.	.	.	.
REG00014	CDK11A	GSE92220	.	.	.	.
REG00014	TAF8	GSE92220	.	.	.	.
REG00014	PCSK1N	GSE92220	.	.	.	.
REG00014	NEGR1	GSE92220	.	.	.	.
REG00014	TAF3	GSE92220	.	.	.	.
REG00014	TAF5L	GSE92220	.	.	.	.
REG00014	TAF6L	GSE92220	.	.	.	.
REG00014	TAF9B	GSE92220	.	.	.	.
REG00014	BTAF1	GSE92220	.	.	.	.
REG00014	IP6K2	GSE92220	.	.	.	.
REG00014	TIMM17A	GSE92220	.	.	.	.
REG00014	TIMM44	GSE92220	.	.	.	.
REG00014	TIMM22	GSE92220	.	.	.	.
REG00014	PRODH2	GSE92220	.	.	.	.
REG00014	WAC	GSE92220	.	.	.	.

REG00014	DTNBP1	GSE92220	.	.	.	.
REG00014	SLA2	GSE92220	.	.	.	.
REG00014	CDK13	GSE92220	.	.	.	.
REG00014	CDC34	GSE92220	.	.	.	.
REG00014	PRPF8	GSE92220	.	.	.	.
REG00014	BRWD3	GSE92220	.	.	.	.
REG00014	PRPF4B	GSE92220	.	.	.	.
REG00014	PRPF3	GSE92220	.	.	.	.
REG00014	APOBEC3D	GSE92220	.	.	.	.
REG00014	NUP54	GSE92220	.	.	.	.
REG00014	CDC42	GSE92220	.	.	.	.
REG00014	LRIG1	GSE92220	.	.	.	.
REG00014	ARID5B	GSE92220	.	.	.	.
REG00014	YAF2	GSE92220	.	.	.	.
REG00014	IRAK1BP1	GSE92220	.	.	.	.
REG00014	ABT1	GSE92220	.	.	.	.
REG00014	CDC42BPA	GSE92220	.	.	.	.
REG00014	FHL5	GSE92220	.	.	.	.
REG00014	AK3	GSE92220	.	.	.	.
REG00014	CDC42BPB	GSE92220	.	.	.	.
REG00014	ANAPC7	GSE92220	.	.	.	.
REG00014	SH2B2	GSE92220	.	.	.	.
REG00014	SMPDL3A	GSE92220	.	.	.	.
REG00014	CDC45	GSE92220	.	.	.	.
REG00014	MARCHF7	GSE92220	.	.	.	.
REG00014	BANF1	GSE92220	.	.	.	.
REG00014	ARHGAP32	GSE92220	.	.	.	.
REG00014	ACVR2B	GSE92220	.	.	.	.
REG00014	CXorf40B	GSE92220	.	.	.	.
REG00014	LRBA	GSE92220	.	.	.	.
REG00014	CHST12	GSE92220	.	.	.	.
REG00014	NPFFR1	GSE92220	.	.	.	.
REG00014	RAPGEFL1	GSE92220	.	.	.	.
REG00014	CHP1	GSE92220	.	.	.	.
REG00014	SLC12A9	GSE92220	.	.	.	.
REG00014	CDC6	GSE92220	.	.	.	.
REG00014	RFWD2	GSE92220	.	.	.	.
REG00014	RCOR1	GSE92220	.	.	.	.
REG00014	TBRG4	GSE92220	.	.	.	.
REG00014	CLSTN1	GSE92220	.	.	.	.
REG00014	CDC7	GSE92220	.	.	.	.
REG00014	AS3MT	GSE92220	.	.	.	.
REG00014	PDLIM5	GSE92220	.	.	.	.
REG00014	PJA2	GSE92220	.	.	.	.
REG00014	GMNN	GSE92220	.	.	.	.
REG00014	GJC2	GSE92220	.	.	.	.
REG00014	SERGEF	GSE92220	.	.	.	.
REG00014	GORASP2	GSE92220	.	.	.	.
REG00014	HOMER1	GSE92220	.	.	.	.
REG00014	HOMER3	GSE92220	.	.	.	.

REG00014	INSM2	GSE92220	.	.	.	.
REG00014	JDP2	GSE92220	.	.	.	.
REG00014	CDH16	GSE92220	.	.	.	.
REG00014	KREMEN1	GSE92220	.	.	.	.
REG00014	MED16	GSE92220	.	.	.	.
REG00014	LCMT1	GSE92220	.	.	.	.
REG00014	LSM10	GSE92220	.	.	.	.
REG00014	METTL3	GSE92220	.	.	.	.
REG00014	MINK1	GSE92220	.	.	.	.
REG00014	EPDR1	GSE92220	.	.	.	.
REG00014	ALPK3	GSE92220	.	.	.	.
REG00014	SPEN	GSE92220	.	.	.	.
REG00014	STARD3	GSE92220	.	.	.	.
REG00014	MTCH2	GSE92220	.	.	.	.
REG00014	ORAOV1	GSE92220	.	.	.	.
REG00014	CDH2	GSE92220	.	.	.	.
REG00014	C12orf10	GSE92220	.	.	.	.
REG00014	CLDN23	GSE92220	.	.	.	.
REG00014	NDFIP1	GSE92220	.	.	.	.
REG00014	NCDN	GSE92220	.	.	.	.
REG00014	BFAR	GSE92220	.	.	.	.
REG00014	NDE1	GSE92220	.	.	.	.
REG00014	NDEL1	GSE92220	.	.	.	.
REG00014	POLR1E	GSE92220	.	.	.	.
REG00014	NUFIP2	GSE92220	.	.	.	.
REG00014	PERP	GSE92220	.	.	.	.
REG00014	GOPC	GSE92220	.	.	.	.
REG00014	NGLY1	GSE92220	.	.	.	.
REG00014	POPDC3	GSE92220	.	.	.	.
REG00014	NECTIN3	GSE92220	.	.	.	.
REG00014	XPO5	GSE92220	.	.	.	.
REG00014	RABGEF1	GSE92220	.	.	.	.
REG00014	RABEP1	GSE92220	.	.	.	.
REG00014	RBAK	GSE92220	.	.	.	.
REG00014	ARMC1	GSE92220	.	.	.	.
REG00014	RIC1	GSE92220	.	.	.	.
REG00014	RCL1	GSE92220	.	.	.	.
REG00014	POP5	GSE92220	.	.	.	.
REG00014	CDIPT	GSE92220	.	.	.	.
REG00014	DHRS3	GSE92220	.	.	.	.
REG00014	ACY1	GSE92220	.	.	.	.
REG00014	SELENOF	GSE92220	.	.	.	.
REG00014	SIVA1	GSE92220	.	.	.	.
REG00014	STK39	GSE92220	.	.	.	.
REG00014	SEC11A	GSE92220	.	.	.	.
REG00014	CDC42SE1	GSE92220	.	.	.	.
REG00014	CDK3	GSE92220	.	.	.	.
REG00014	SGPP1	GSE92220	.	.	.	.
REG00014	C20orf194	GSE92220	.	.	.	.
REG00014	SPRED2	GSE92220	.	.	.	.

REG00014	UXS1	GSE92220	.	.	.	.
REG00014	CLASRP	GSE92220	.	.	.	.
REG00014	TMEM63B	GSE92220	.	.	.	.
REG00014	CDK5R1	GSE92220	.	.	.	.
REG00014	TSPAN3	GSE92220	.	.	.	.
REG00014	EDA2R	GSE92220	.	.	.	.
REG00014	PDSS1	GSE92220	.	.	.	.
REG00014	RNF138	GSE92220	.	.	.	.
REG00014	TDP2	GSE92220	.	.	.	.
REG00014	CDK6	GSE92220	.	.	.	.
REG00014	RALGAPA1	GSE92220	.	.	.	.
REG00014	TXN2	GSE92220	.	.	.	.
REG00014	UBOX5	GSE92220	.	.	.	.
REG00014	CDK7	GSE92220	.	.	.	.
REG00014	LIN7A	GSE92220	.	.	.	.
REG00014	LIN7B	GSE92220	.	.	.	.
REG00014	LIN7C	GSE92220	.	.	.	.
REG00014	CDK8	GSE92220	.	.	.	.
REG00014	TBC1D8	GSE92220	.	.	.	.
REG00014	VTI1A	GSE92220	.	.	.	.
REG00014	VTI1B	GSE92220	.	.	.	.
REG00014	RHOU	GSE92220	.	.	.	.
REG00014	SAV1	GSE92220	.	.	.	.
REG00014	YLPM1	GSE92220	.	.	.	.
REG00014	CDK9	GSE92220	.	.	.	.
REG00014	FARSB	GSE92220	.	.	.	.
REG00014	PIPOX	GSE92220	.	.	.	.
REG00014	CYHR1	GSE92220	.	.	.	.
REG00014	ZC3H4	GSE92220	.	.	.	.
REG00014	SMG6	GSE92220	.	.	.	.
REG00014	CDKL1	GSE92220	.	.	.	.
REG00014	AMOTL2	GSE92220	.	.	.	.
REG00014	IGSF8	GSE92220	.	.	.	.
REG00014	SLF2	GSE92220	.	.	.	.
REG00014	CDKL2	GSE92220	.	.	.	.
REG00014	NT5C3A	GSE92220	.	.	.	.
REG00014	DDX39A	GSE92220	.	.	.	.
REG00014	PTGES2	GSE92220	.	.	.	.
REG00014	C9orf16	GSE92220	.	.	.	.
REG00014	PLA2G16	GSE92220	.	.	.	.
REG00014	ST8SIA5	GSE92220	.	.	.	.
REG00014	MDH1B	GSE92220	.	.	.	.
REG00014	NIFK	GSE92220	.	.	.	.
REG00014	CDC26	GSE92220	.	.	.	.
REG00014	CDKN1A	GSE92220	.	.	.	.
REG00014	CHCHD5	GSE92220	.	.	.	.
REG00014	GGA1	GSE92220	.	.	.	.
REG00014	KLHL36	GSE92220	.	.	.	.
REG00014	ST6GALNAC4	GSE92220	.	.	.	.
REG00014	CDKN1B	GSE92220	.	.	.	.

REG00014	HINFP	GSE92220	.	.	.	.
REG00014	REXO2	GSE92220	.	.	.	.
REG00014	CHPT1	GSE92220	.	.	.	.
REG00014	IBTK	GSE92220	.	.	.	.
REG00014	UPF2	GSE92220	.	.	.	.
REG00014	GPSM1	GSE92220	.	.	.	.
REG00014	NUP188	GSE92220	.	.	.	.
REG00014	SERBP1	GSE92220	.	.	.	.
REG00014	TRAF3IP1	GSE92220	.	.	.	.
REG00014	NPTN	GSE92220	.	.	.	.
REG00014	BBC3	GSE92220	.	.	.	.
REG00014	AFF4	GSE92220	.	.	.	.
REG00014	ARMT1	GSE92220	.	.	.	.
REG00014	NMNAT1	GSE92220	.	.	.	.
REG00014	LMTK2	GSE92220	.	.	.	.
REG00014	NRSN1	GSE92220	.	.	.	.
REG00014	SREK1	GSE92220	.	.	.	.
REG00014	CRCP	GSE92220	.	.	.	.
REG00014	APEX2	GSE92220	.	.	.	.
REG00014	PGAP2	GSE92220	.	.	.	.
REG00014	ZNF638	GSE92220	.	.	.	.
REG00014	PRPF19	GSE92220	.	.	.	.
REG00014	NKIRAS2	GSE92220	.	.	.	.
REG00014	COMMD5	GSE92220	.	.	.	.
REG00014	MED4	GSE92220	.	.	.	.
REG00014	DROSHA	GSE92220	.	.	.	.
REG00014	DUSP16	GSE92220	.	.	.	.
REG00014	CDKN3	GSE92220	.	.	.	.
REG00014	MIEF2	GSE92220	.	.	.	.
REG00014	SMCR8	GSE92220	.	.	.	.
REG00014	REPIN1	GSE92220	.	.	.	.
REG00014	TFCP2L1	GSE92220	.	.	.	.
REG00014	PSMC3IP	GSE92220	.	.	.	.
REG00014	PURG	GSE92220	.	.	.	.
REG00014	SERTAD3	GSE92220	.	.	.	.
REG00014	KAT8	GSE92220	.	.	.	.
REG00014	PODXL2	GSE92220	.	.	.	.
REG00014	SLC45A1	GSE92220	.	.	.	.
REG00014	DERL2	GSE92220	.	.	.	.
REG00014	EXOSC3	GSE92220	.	.	.	.
REG00014	PRLH	GSE92220	.	.	.	.
REG00014	NOSIP	GSE92220	.	.	.	.
REG00014	TJAP1	GSE92220	.	.	.	.
REG00014	WDR18	GSE92220	.	.	.	.
REG00014	SAMD1	GSE92220	.	.	.	.
REG00014	RDH11	GSE92220	.	.	.	.
REG00014	PAIP2	GSE92220	.	.	.	.
REG00014	C6orf203	GSE92220	.	.	.	.
REG00014	REEP2	GSE92220	.	.	.	.
REG00014	CDR2	GSE92220	.	.	.	.

REG00014	TRPM7	GSE92220	.	.	.	.
REG00014	NAGS	GSE92220	.	.	.	.
REG00014	FKRP	GSE92220	.	.	.	.
REG00014	PKN3	GSE92220	.	.	.	.
REG00014	AAMP	GSE92220	.	.	.	.
REG00014	ACYP2	GSE92220	.	.	.	.
REG00014	CDS1	GSE92220	.	.	.	.
REG00014	PHF5A	GSE92220	.	.	.	.
REG00014	TOMM40	GSE92220	.	.	.	.
REG00014	TOMM22	GSE92220	.	.	.	.
REG00014	NXN	GSE92220	.	.	.	.
REG00014	CDS2	GSE92220	.	.	.	.
REG00014	GDAP2	GSE92220	.	.	.	.
REG00014	IL17RB	GSE92220	.	.	.	.
REG00014	NUP133	GSE92220	.	.	.	.
REG00014	NUP160	GSE92220	.	.	.	.
REG00014	ZFAND3	GSE92220	.	.	.	.
REG00014	TMC6	GSE92220	.	.	.	.
REG00014	TP53INP1	GSE92220	.	.	.	.
REG00014	EMID1	GSE92220	.	.	.	.
REG00014	ARID2	GSE92220	.	.	.	.
REG00014	ARID1B	GSE92220	.	.	.	.
REG00014	CNOT6L	GSE92220	.	.	.	.
REG00014	SLC38A3	GSE92220	.	.	.	.
REG00014	ACSM1	GSE92220	.	.	.	.
REG00014	RNF38	GSE92220	.	.	.	.
REG00014	PKD1L1	GSE92220	.	.	.	.
REG00014	GPHB5	GSE92220	.	.	.	.
REG00014	EBPL	GSE92220	.	.	.	.
REG00014	GPT2	GSE92220	.	.	.	.
REG00014	NAT8	GSE92220	.	.	.	.
REG00014	SLC38A5	GSE92220	.	.	.	.
REG00014	EMSY	GSE92220	.	.	.	.
REG00014	ARL6IP4	GSE92220	.	.	.	.
REG00014	ZBTB7A	GSE92220	.	.	.	.
REG00014	ZNF362	GSE92220	.	.	.	.
REG00014	ST3GAL6	GSE92220	.	.	.	.
REG00014	TRPV4	GSE92220	.	.	.	.
REG00014	TMEM199	GSE92220	.	.	.	.
REG00014	CDYL	GSE92220	.	.	.	.
REG00014	CCDC50	GSE92220	.	.	.	.
REG00014	KHDRBS1	GSE92220	.	.	.	.
REG00014	RSF1	GSE92220	.	.	.	.
REG00014	SPATA5	GSE92220	.	.	.	.
REG00014	ZNRF3	GSE92220	.	.	.	.
REG00014	TUBGCP6	GSE92220	.	.	.	.
REG00014	HDAC10	GSE92220	.	.	.	.
REG00014	GHRL	GSE92220	.	.	.	.
REG00014	SYCP3	GSE92220	.	.	.	.
REG00014	SELENOT	GSE92220	.	.	.	.

REG00014	CHST15	GSE92220	.	.	.	.
REG00014	EIF3L	GSE92220	.	.	.	.
REG00014	CEACAM1	GSE92220	.	.	.	.
REG00014	DCDC2	GSE92220	.	.	.	.
REG00014	DAAM1	GSE92220	.	.	.	.
REG00014	MYO18B	GSE92220	.	.	.	.
REG00014	TNFRSF12A	GSE92220	.	.	.	.
REG00014	TXNRD2	GSE92220	.	.	.	.
REG00014	TAB1	GSE92220	.	.	.	.
REG00014	SCLY	GSE92220	.	.	.	.
REG00014	TRIM73	GSE92220	.	.	.	.
REG00014	FKBP10	GSE92220	.	.	.	.
REG00014	CMPK1	GSE92220	.	.	.	.
REG00014	ERAP1	GSE92220	.	.	.	.
REG00014	STK26	GSE92220	.	.	.	.
REG00014	COQ3	GSE92220	.	.	.	.
REG00014	VPS28	GSE92220	.	.	.	.
REG00014	VPS33A	GSE92220	.	.	.	.
REG00014	TPCN1	GSE92220	.	.	.	.
REG00014	DCUN1D1	GSE92220	.	.	.	.
REG00014	ERRFI1	GSE92220	.	.	.	.
REG00014	TMCO1	GSE92220	.	.	.	.
REG00014	EXOSC4	GSE92220	.	.	.	.
REG00014	MTMR12	GSE92220	.	.	.	.
REG00014	DDX56	GSE92220	.	.	.	.
REG00014	TMEM37	GSE92220	.	.	.	.
REG00014	ZRANB1	GSE92220	.	.	.	.
REG00014	CDHR2	GSE92220	.	.	.	.
REG00014	MOCOS	GSE92220	.	.	.	.
REG00014	ARHGAP17	GSE92220	.	.	.	.
REG00014	RCBTB1	GSE92220	.	.	.	.
REG00014	SMU1	GSE92220	.	.	.	.
REG00014	ELP2	GSE92220	.	.	.	.
REG00014	PHF10	GSE92220	.	.	.	.
REG00014	SELENOH	GSE92220	.	.	.	.
REG00014	PARL	GSE92220	.	.	.	.
REG00014	HES6	GSE92220	.	.	.	.
REG00014	NPLOC4	GSE92220	.	.	.	.
REG00014	RAB21	GSE92220	.	.	.	.
REG00014	FMNL2	GSE92220	.	.	.	.
REG00014	BTBD7	GSE92220	.	.	.	.
REG00014	HHAT	GSE92220	.	.	.	.
REG00014	ENAH	GSE92220	.	.	.	.
REG00014	SEC61G	GSE92220	.	.	.	.
REG00014	UTP6	GSE92220	.	.	.	.
REG00014	RNF130	GSE92220	.	.	.	.
REG00014	PECR	GSE92220	.	.	.	.
REG00014	RAB40B	GSE92220	.	.	.	.
REG00014	RAB40C	GSE92220	.	.	.	.
REG00014	CMAS	GSE92220	.	.	.	.

REG00014	DMAP1	GSE92220	.	.	.	.
REG00014	ALG1	GSE92220	.	.	.	.
REG00014	PPP4R2	GSE92220	.	.	.	.
REG00014	PILRB	GSE92220	.	.	.	.
REG00014	SLC2A14	GSE92220	.	.	.	.
REG00014	MDN1	GSE92220	.	.	.	.
REG00014	ATP6V1H	GSE92220	.	.	.	.
REG00014	COTL1	GSE92220	.	.	.	.
REG00014	ATP6AP2	GSE92220	.	.	.	.
REG00014	KCNG3	GSE92220	.	.	.	.
REG00014	ERP44	GSE92220	.	.	.	.
REG00014	PXT1	GSE92220	.	.	.	.
REG00014	ASXL1	GSE92220	.	.	.	.
REG00014	PRIMA1	GSE92220	.	.	.	.
REG00014	HSD3B7	GSE92220	.	.	.	.
REG00014	YPEL3	GSE92220	.	.	.	.
REG00014	YPEL5	GSE92220	.	.	.	.
REG00014	CEBPA	GSE92220	.	.	.	.
REG00014	TIGD6	GSE92220	.	.	.	.
REG00014	TIGD2	GSE92220	.	.	.	.
REG00014	TIGD3	GSE92220	.	.	.	.
REG00014	TIGD5	GSE92220	.	.	.	.
REG00014	CEBPB	GSE92220	.	.	.	.
REG00014	FAM46A	GSE92220	.	.	.	.
REG00014	TICAM1	GSE92220	.	.	.	.
REG00014	DHRS2	GSE92220	.	.	.	.
REG00014	CEBPD	GSE92220	.	.	.	.
REG00014	IL17RC	GSE92220	.	.	.	.
REG00014	IP6K1	GSE92220	.	.	.	.
REG00014	CEBPG	GSE92220	.	.	.	.
REG00014	RAB1B	GSE92220	.	.	.	.
REG00014	CLSTN3	GSE92220	.	.	.	.
REG00014	SESTD1	GSE92220	.	.	.	.
REG00014	BZW1	GSE92220	.	.	.	.
REG00014	SCGB3A1	GSE92220	.	.	.	.
REG00014	SFTA2	GSE92220	.	.	.	.
REG00014	SMARCAD1	GSE92220	.	.	.	.
REG00014	RNF41	GSE92220	.	.	.	.
REG00014	UBXN1	GSE92220	.	.	.	.
REG00014	AGMAT	GSE92220	.	.	.	.
REG00014	ZNF292	GSE92220	.	.	.	.
REG00014	UCN2	GSE92220	.	.	.	.
REG00014	HIP1R	GSE92220	.	.	.	.
REG00014	FICD	GSE92220	.	.	.	.
REG00014	HYPK	GSE92220	.	.	.	.
REG00014	SETD2	GSE92220	.	.	.	.
REG00014	AGO3	GSE92220	.	.	.	.
REG00014	AGO4	GSE92220	.	.	.	.
REG00014	MIA2	GSE92220	.	.	.	.
REG00014	CHTF18	GSE92220	.	.	.	.

REG00014	CECR6	GSE92220	.	.	.	.
REG00014	ST7L	GSE92220	.	.	.	.
REG00014	CALML4	GSE92220	.	.	.	.
REG00014	LAP3	GSE92220	.	.	.	.
REG00014	MCFD2	GSE92220	.	.	.	.
REG00014	ZNRF1	GSE92220	.	.	.	.
REG00014	ZDHHC7	GSE92220	.	.	.	.
REG00014	H1FOO	GSE92220	.	.	.	.
REG00014	RNASEH1	GSE92220	.	.	.	.
REG00014	ZDHHC3	GSE92220	.	.	.	.
REG00014	ZDHHC4	GSE92220	.	.	.	.
REG00014	ZDHHC5	GSE92220	.	.	.	.
REG00014	ZDHHC8	GSE92220	.	.	.	.
REG00014	ZDHHC9	GSE92220	.	.	.	.
REG00014	RPS27L	GSE92220	.	.	.	.
REG00014	MRTO4	GSE92220	.	.	.	.
REG00014	RSL24D1	GSE92220	.	.	.	.
REG00014	MRM3	GSE92220	.	.	.	.
REG00014	TBL1Y	GSE92220	.	.	.	.
REG00014	RNF43	GSE92220	.	.	.	.
REG00014	CENPA	GSE92220	.	.	.	.
REG00014	ZHX2	GSE92220	.	.	.	.
REG00014	VASN	GSE92220	.	.	.	.
REG00014	CENPB	GSE92220	.	.	.	.
REG00014	USP48	GSE92220	.	.	.	.
REG00014	NUSAP1	GSE92220	.	.	.	.
REG00014	KMT2E	GSE92220	.	.	.	.
REG00014	CDC42SE2	GSE92220	.	.	.	.
REG00014	PLLP	GSE92220	.	.	.	.
REG00014	PLA2G12A	GSE92220	.	.	.	.
REG00014	PLA2G12B	GSE92220	.	.	.	.
REG00014	CENPF	GSE92220	.	.	.	.
REG00014	HSDL2	GSE92220	.	.	.	.
REG00014	ADGRL2	GSE92220	.	.	.	.
REG00014	PPM1H	GSE92220	.	.	.	.
REG00014	MSI2	GSE92220	.	.	.	.
REG00014	SUN1	GSE92220	.	.	.	.
REG00014	C22orf23	GSE92220	.	.	.	.
REG00014	CEP250	GSE92220	.	.	.	.
REG00014	PNPLA3	GSE92220	.	.	.	.
REG00014	NEK9	GSE92220	.	.	.	.
REG00014	NEK10	GSE92220	.	.	.	.
REG00014	NEK11	GSE92220	.	.	.	.
REG00014	HIC2	GSE92220	.	.	.	.
REG00014	TUBGCP3	GSE92220	.	.	.	.
REG00014	ADA	GSE92220	.	.	.	.
REG00014	TUBGCP5	GSE92220	.	.	.	.
REG00014	SLC39A6	GSE92220	.	.	.	.
REG00014	COG3	GSE92220	.	.	.	.
REG00014	COG4	GSE92220	.	.	.	.

REG00014	COG7	GSE92220	.	.	.	.
REG00014	COG8	GSE92220	.	.	.	.
REG00014	FKBP11	GSE92220	.	.	.	.
REG00014	FKBP14	GSE92220	.	.	.	.
REG00014	STAB1	GSE92220	.	.	.	.
REG00014	CES1	GSE92220	.	.	.	.
REG00014	CES2	GSE92220	.	.	.	.
REG00014	LDLRAP1	GSE92220	.	.	.	.
REG00014	SUGP2	GSE92220	.	.	.	.
REG00014	SUGP1	GSE92220	.	.	.	.
REG00014	KLHL8	GSE92220	.	.	.	.
REG00014	HSD17B12	GSE92220	.	.	.	.
REG00014	DPP9	GSE92220	.	.	.	.
REG00014	SPATS2	GSE92220	.	.	.	.
REG00014	VPS54	GSE92220	.	.	.	.
REG00014	RIOK1	GSE92220	.	.	.	.
REG00014	TTC39A	GSE92220	.	.	.	.
REG00014	NUP205	GSE92220	.	.	.	.
REG00014	FAM3C	GSE92220	.	.	.	.
REG00014	PMPCA	GSE92220	.	.	.	.
REG00014	ZBTB7B	GSE92220	.	.	.	.
REG00014	MPP5	GSE92220	.	.	.	.
REG00014	CDK5RAP2	GSE92220	.	.	.	.
REG00014	CDK5RAP3	GSE92220	.	.	.	.
REG00014	DDX41	GSE92220	.	.	.	.
REG00014	DDX42	GSE92220	.	.	.	.
REG00014	DDX46	GSE92220	.	.	.	.
REG00014	DDX47	GSE92220	.	.	.	.
REG00014	EIF4A3	GSE92220	.	.	.	.
REG00014	DDX49	GSE92220	.	.	.	.
REG00014	HSD17B13	GSE92220	.	.	.	.
REG00014	CRB2	GSE92220	.	.	.	.
REG00014	CITED4	GSE92220	.	.	.	.
REG00014	ASCC3	GSE92220	.	.	.	.
REG00014	DDX25	GSE92220	.	.	.	.
REG00014	NAA10	GSE92220	.	.	.	.
REG00014	GRIPAP1	GSE92220	.	.	.	.
REG00014	GRASP	GSE92220	.	.	.	.
REG00014	CCNB3	GSE92220	.	.	.	.
REG00014	GLCCI1	GSE92220	.	.	.	.
REG00014	ABHD3	GSE92220	.	.	.	.
REG00014	CFDP1	GSE92220	.	.	.	.
REG00014	SKIV2L2	GSE92220	.	.	.	.
REG00014	RHOBTB1	GSE92220	.	.	.	.
REG00014	CFL1	GSE92220	.	.	.	.
REG00014	SLURP1	GSE92220	.	.	.	.
REG00014	RIN1	GSE92220	.	.	.	.
REG00014	CFL2	GSE92220	.	.	.	.
REG00014	RIN3	GSE92220	.	.	.	.
REG00014	MAPKAP1	GSE92220	.	.	.	.

REG00014	FBXO17	GSE92220	.	.	.	.
REG00014	POLE4	GSE92220	.	.	.	.
REG00014	RHOBTB2	GSE92220	.	.	.	.
REG00014	RHOBTB3	GSE92220	.	.	.	.
REG00014	CFLAR	GSE92220	.	.	.	.
REG00014	PCNX3	GSE92220	.	.	.	.
REG00014	SLC36A1	GSE92220	.	.	.	.
REG00014	RRP1	GSE92220	.	.	.	.
REG00014	FAM50A	GSE92220	.	.	.	.
REG00014	FAM50B	GSE92220	.	.	.	.
REG00014	KREMEN2	GSE92220	.	.	.	.
REG00014	SLC44A1	GSE92220	.	.	.	.
REG00014	ATP5S	GSE92220	.	.	.	.
REG00014	ADAM10	GSE92220	.	.	.	.
REG00014	POGK	GSE92220	.	.	.	.
REG00014	POGZ	GSE92220	.	.	.	.
REG00014	ATPAF1	GSE92220	.	.	.	.
REG00014	CAMTA1	GSE92220	.	.	.	.
REG00014	BZW2	GSE92220	.	.	.	.
REG00014	TUBA1B	GSE92220	.	.	.	.
REG00014	ZNF397	GSE92220	.	.	.	.
REG00014	TMEM9	GSE92220	.	.	.	.
REG00014	SERINC5	GSE92220	.	.	.	.
REG00014	NUDT13	GSE92220	.	.	.	.
REG00014	KLHL10	GSE92220	.	.	.	.
REG00014	ZNF394	GSE92220	.	.	.	.
REG00014	PPP1R13L	GSE92220	.	.	.	.
REG00014	STH	GSE92220	.	.	.	.
REG00014	CYP4F12	GSE92220	.	.	.	.
REG00014	PIGM	GSE92220	.	.	.	.
REG00014	SPHK2	GSE92220	.	.	.	.
REG00014	ASAH2	GSE92220	.	.	.	.
REG00014	KCNU1	GSE92220	.	.	.	.
REG00014	GGN	GSE92220	.	.	.	.
REG00014	POLN	GSE92220	.	.	.	.
REG00014	LAT	GSE92220	.	.	.	.
REG00014	CGGBP1	GSE92220	.	.	.	.
REG00014	TMEM185B	GSE92220	.	.	.	.
REG00014	MAGI2	GSE92220	.	.	.	.
REG00014	CSAD	GSE92220	.	.	.	.
REG00014	EDEM1	GSE92220	.	.	.	.
REG00014	AP1S3	GSE92220	.	.	.	.
REG00014	ARNTL2	GSE92220	.	.	.	.
REG00014	DCXR	GSE92220	.	.	.	.
REG00014	GBA2	GSE92220	.	.	.	.
REG00014	THTPA	GSE92220	.	.	.	.
REG00014	ADGRF3	GSE92220	.	.	.	.
REG00014	VRK3	GSE92220	.	.	.	.
REG00014	AANAT	GSE92220	.	.	.	.
REG00014	DCLK2	GSE92220	.	.	.	.

REG00014	SCMH1	GSE92220	.	.	.	.
REG00014	HIPK1	GSE92220	.	.	.	.
REG00014	KLHL11	GSE92220	.	.	.	.
REG00014	TRIM41	GSE92220	.	.	.	.
REG00014	TRIM44	GSE92220	.	.	.	.
REG00014	TRIM50	GSE92220	.	.	.	.
REG00014	TRIM47	GSE92220	.	.	.	.
REG00014	HSPA12A	GSE92220	.	.	.	.
REG00014	TRIM56	GSE92220	.	.	.	.
REG00014	C1orf35	GSE92220	.	.	.	.
REG00014	MAST2	GSE92220	.	.	.	.
REG00014	COQ8B	GSE92220	.	.	.	.
REG00014	SLCO5A1	GSE92220	.	.	.	.
REG00014	ASPM	GSE92220	.	.	.	.
REG00014	EXOSC6	GSE92220	.	.	.	.
REG00014	CHD6	GSE92220	.	.	.	.
REG00014	ZBTB12	GSE92220	.	.	.	.
REG00014	CH25H	GSE92220	.	.	.	.
REG00014	THOC1	GSE92220	.	.	.	.
REG00014	THOC3	GSE92220	.	.	.	.
REG00014	THOC2	GSE92220	.	.	.	.
REG00014	THOC5	GSE92220	.	.	.	.
REG00014	TIFA	GSE92220	.	.	.	.
REG00014	C6orf47	GSE92220	.	.	.	.
REG00014	PLEKHB1	GSE92220	.	.	.	.
REG00014	TNKS1BP1	GSE92220	.	.	.	.
REG00014	CTDNEP1	GSE92220	.	.	.	.
REG00014	ASH1L	GSE92220	.	.	.	.
REG00014	SLC30A5	GSE92220	.	.	.	.
REG00014	CHAD	GSE92220	.	.	.	.
REG00014	IL4I1	GSE92220	.	.	.	.
REG00014	GCC1	GSE92220	.	.	.	.
REG00014	PSD4	GSE92220	.	.	.	.
REG00014	RBPMS	GSE92220	.	.	.	.
REG00014	RBPMS2	GSE92220	.	.	.	.
REG00014	CHAF1A	GSE92220	.	.	.	.
REG00014	TAS2R20	GSE92220	.	.	.	.
REG00014	TAS2R30	GSE92220	.	.	.	.
REG00014	TNIP2	GSE92220	.	.	.	.
REG00014	DNAJC9	GSE92220	.	.	.	.
REG00014	FIP1L1	GSE92220	.	.	.	.
REG00014	PSAT1	GSE92220	.	.	.	.
REG00014	RCC1	GSE92220	.	.	.	.
REG00014	CD276	GSE92220	.	.	.	.
REG00014	POMGNT1	GSE92220	.	.	.	.
REG00014	USP32	GSE92220	.	.	.	.
REG00014	GJD3	GSE92220	.	.	.	.
REG00014	CHD1	GSE92220	.	.	.	.
REG00014	ZDHHC11	GSE92220	.	.	.	.
REG00014	ZDHHC12	GSE92220	.	.	.	.

REG00014	CHD1L	GSE92220	.	.	.	.
REG00014	ZDHHC6	GSE92220	.	.	.	.
REG00014	STARD9	GSE92220	.	.	.	.
REG00014	STARD13	GSE92220	.	.	.	.
REG00014	TBC1D4	GSE92220	.	.	.	.
REG00014	TINAGL1	GSE92220	.	.	.	.
REG00014	STARD3NL	GSE92220	.	.	.	.
REG00014	CHD2	GSE92220	.	.	.	.
REG00014	CMTM3	GSE92220	.	.	.	.
REG00014	CMTM4	GSE92220	.	.	.	.
REG00014	CMTM6	GSE92220	.	.	.	.
REG00014	CMTM8	GSE92220	.	.	.	.
REG00014	CHD3	GSE92220	.	.	.	.
REG00014	RNF44	GSE92220	.	.	.	.
REG00014	SASH1	GSE92220	.	.	.	.
REG00014	HCN3	GSE92220	.	.	.	.
REG00014	STK11IP	GSE92220	.	.	.	.
REG00014	FRAS1	GSE92220	.	.	.	.
REG00014	CHD4	GSE92220	.	.	.	.
REG00014	DOCK7	GSE92220	.	.	.	.
REG00014	DOCK4	GSE92220	.	.	.	.
REG00014	SERPINA11	GSE92220	.	.	.	.
REG00014	STIM2	GSE92220	.	.	.	.
REG00014	SHKBP1	GSE92220	.	.	.	.
REG00014	WSB1	GSE92220	.	.	.	.
REG00014	WSB2	GSE92220	.	.	.	.
REG00014	C4orf3	GSE92220	.	.	.	.
REG00014	FUT11	GSE92220	.	.	.	.
REG00014	AATF	GSE92220	.	.	.	.
REG00014	PLEKHB2	GSE92220	.	.	.	.
REG00014	NANS	GSE92220	.	.	.	.
REG00014	TERF2IP	GSE92220	.	.	.	.
REG00014	CHEK1	GSE92220	.	.	.	.
REG00014	PPP6R2	GSE92220	.	.	.	.
REG00014	PLAC9	GSE92220	.	.	.	.
REG00014	CERK	GSE92220	.	.	.	.
REG00014	LTB4R2	GSE92220	.	.	.	.
REG00014	MTO1	GSE92220	.	.	.	.
REG00014	PAPLN	GSE92220	.	.	.	.
REG00014	LMAN2L	GSE92220	.	.	.	.
REG00014	UBE2J2	GSE92220	.	.	.	.
REG00014	TTC5	GSE92220	.	.	.	.
REG00014	FOXN3	GSE92220	.	.	.	.
REG00014	METTL17	GSE92220	.	.	.	.
REG00014	SCYL2	GSE92220	.	.	.	.
REG00014	CDKL4	GSE92220	.	.	.	.
REG00014	CHGA	GSE92220	.	.	.	.
REG00014	LMTK3	GSE92220	.	.	.	.
REG00014	EPHA6	GSE92220	.	.	.	.
REG00014	TNK2	GSE92220	.	.	.	.

REG00014	TMEM55B	GSE92220	.	.	.	.
REG00014	ADAM15	GSE92220	.	.	.	.
REG00014	SCAF4	GSE92220	.	.	.	.
REG00014	SLC30A6	GSE92220	.	.	.	.
REG00014	SDK1	GSE92220	.	.	.	.
REG00014	KANK1	GSE92220	.	.	.	.
REG00014	PIM3	GSE92220	.	.	.	.
REG00014	EAPP	GSE92220	.	.	.	.
REG00014	P3H1	GSE92220	.	.	.	.
REG00014	NKX6-2	GSE92220	.	.	.	.
REG00014	MMAB	GSE92220	.	.	.	.
REG00014	CDK19	GSE92220	.	.	.	.
REG00014	NRBP2	GSE92220	.	.	.	.
REG00014	DENND5A	GSE92220	.	.	.	.
REG00014	BET1L	GSE92220	.	.	.	.
REG00014	KIF21A	GSE92220	.	.	.	.
REG00014	KDELC1	GSE92220	.	.	.	.
REG00014	SIN3A	GSE92220	.	.	.	.
REG00014	SIN3B	GSE92220	.	.	.	.
REG00014	JMJD6	GSE92220	.	.	.	.
REG00014	ALG12	GSE92220	.	.	.	.
REG00014	C1QL3	GSE92220	.	.	.	.
REG00014	KLHL12	GSE92220	.	.	.	.
REG00014	PANK3	GSE92220	.	.	.	.
REG00014	PANK4	GSE92220	.	.	.	.
REG00014	PPHLN1	GSE92220	.	.	.	.
REG00014	CHKA	GSE92220	.	.	.	.
REG00014	TP53I3	GSE92220	.	.	.	.
REG00014	NKRF	GSE92220	.	.	.	.
REG00014	SLC25A23	GSE92220	.	.	.	.
REG00014	SOCS1	GSE92220	.	.	.	.
REG00014	PPM1F	GSE92220	.	.	.	.
REG00014	NOXO1	GSE92220	.	.	.	.
REG00014	PGBD5	GSE92220	.	.	.	.
REG00014	CHML	GSE92220	.	.	.	.
REG00014	LRRTM3	GSE92220	.	.	.	.
REG00014	HS3ST5	GSE92220	.	.	.	.
REG00014	STOML3	GSE92220	.	.	.	.
REG00014	ING5	GSE92220	.	.	.	.
REG00014	ING4	GSE92220	.	.	.	.
REG00014	IPO4	GSE92220	.	.	.	.
REG00014	CNIH1	GSE92220	.	.	.	.
REG00014	CHN2	GSE92220	.	.	.	.
REG00014	SBDS	GSE92220	.	.	.	.
REG00014	RIOX2	GSE92220	.	.	.	.
REG00014	CHRD	GSE92220	.	.	.	.
REG00014	ADAM17	GSE92220	.	.	.	.
REG00014	CHRNB1	GSE92220	.	.	.	.
REG00014	CHRNB2	GSE92220	.	.	.	.
REG00014	CHST10	GSE92220	.	.	.	.

REG00014	SMAP1	GSE92220	.	.	.	.
REG00014	STXBP6	GSE92220	.	.	.	.
REG00014	WDR20	GSE92220	.	.	.	.
REG00014	GPBAR1	GSE92220	.	.	.	.
REG00014	UHMK1	GSE92220	.	.	.	.
REG00014	SEPHS1	GSE92220	.	.	.	.
REG00014	SEPHS2	GSE92220	.	.	.	.
REG00014	EIF2AK4	GSE92220	.	.	.	.
REG00014	NRBF2	GSE92220	.	.	.	.
REG00014	STXBP4	GSE92220	.	.	.	.
REG00014	GRPEL1	GSE92220	.	.	.	.
REG00014	POM121	GSE92220	.	.	.	.
REG00014	ULK3	GSE92220	.	.	.	.
REG00014	LDHD	GSE92220	.	.	.	.
REG00014	CHST3	GSE92220	.	.	.	.
REG00014	CANT1	GSE92220	.	.	.	.
REG00014	XPO6	GSE92220	.	.	.	.
REG00014	EPM2AIP1	GSE92220	.	.	.	.
REG00014	TNS2	GSE92220	.	.	.	.
REG00014	TTC6	GSE92220	.	.	.	.
REG00014	PCNX1	GSE92220	.	.	.	.
REG00014	POMT2	GSE92220	.	.	.	.
REG00014	VSX2	GSE92220	.	.	.	.
REG00014	FNBP4	GSE92220	.	.	.	.
REG00014	RUFY1	GSE92220	.	.	.	.
REG00014	ZFPM1	GSE92220	.	.	.	.
REG00014	ASB13	GSE92220	.	.	.	.
REG00014	ASB16	GSE92220	.	.	.	.
REG00014	CIDEB	GSE92220	.	.	.	.
REG00014	EPB41L4B	GSE92220	.	.	.	.
REG00014	EPB41L5	GSE92220	.	.	.	.
REG00014	SYF2	GSE92220	.	.	.	.
REG00014	TSSK4	GSE92220	.	.	.	.
REG00014	NOP9	GSE92220	.	.	.	.
REG00014	FAM177A1	GSE92220	.	.	.	.
REG00014	UTP4	GSE92220	.	.	.	.
REG00014	FBXO33	GSE92220	.	.	.	.
REG00014	MAPK1IP1L	GSE92220	.	.	.	.
REG00014	NAA30	GSE92220	.	.	.	.
REG00014	ELMSAN1	GSE92220	.	.	.	.
REG00014	FAM161B	GSE92220	.	.	.	.
REG00014	ISCA2	GSE92220	.	.	.	.
REG00014	SYNE3	GSE92220	.	.	.	.
REG00014	POU2F3	GSE92220	.	.	.	.
REG00014	MBTD1	GSE92220	.	.	.	.
REG00014	DOK4	GSE92220	.	.	.	.
REG00014	SCARF2	GSE92220	.	.	.	.
REG00014	GALNT10	GSE92220	.	.	.	.
REG00014	BRD8	GSE92220	.	.	.	.
REG00014	GALNT11	GSE92220	.	.	.	.

REG00014	EMILIN2	GSE92220	.	.	.	.
REG00014	HOOK1	GSE92220	.	.	.	.
REG00014	HOOK2	GSE92220	.	.	.	.
REG00014	TBCB	GSE92220	.	.	.	.
REG00014	RRAGC	GSE92220	.	.	.	.
REG00014	UBE2R2	GSE92220	.	.	.	.
REG00014	RPP25L	GSE92220	.	.	.	.
REG00014	CKB	GSE92220	.	.	.	.
REG00014	DCAF12	GSE92220	.	.	.	.
REG00014	KIAA1161	GSE92220	.	.	.	.
REG00014	C9orf24	GSE92220	.	.	.	.
REG00014	CKM	GSE92220	.	.	.	.
REG00014	TRAPPC3	GSE92220	.	.	.	.
REG00014	CAMSAP1	GSE92220	.	.	.	.
REG00014	SLC25A22	GSE92220	.	.	.	.
REG00014	KIAA0391	GSE92220	.	.	.	.
REG00014	FAM179B	GSE92220	.	.	.	.
REG00014	CKMT2	GSE92220	.	.	.	.
REG00014	KIAA0586	GSE92220	.	.	.	.
REG00014	ATG14	GSE92220	.	.	.	.
REG00014	TTLL5	GSE92220	.	.	.	.
REG00014	CCDC88C	GSE92220	.	.	.	.
REG00014	NAP1L5	GSE92220	.	.	.	.
REG00014	MED6	GSE92220	.	.	.	.
REG00014	MED8	GSE92220	.	.	.	.
REG00014	CLMN	GSE92220	.	.	.	.
REG00014	RHPN1	GSE92220	.	.	.	.
REG00014	RHPN2	GSE92220	.	.	.	.
REG00014	RDH10	GSE92220	.	.	.	.
REG00014	RDH14	GSE92220	.	.	.	.
REG00014	GNPNAT1	GSE92220	.	.	.	.
REG00014	RAB43	GSE92220	.	.	.	.
REG00014	SUB1	GSE92220	.	.	.	.
REG00014	CYCS	GSE92220	.	.	.	.
REG00014	ANAPC1	GSE92220	.	.	.	.
REG00014	GPR135	GSE92220	.	.	.	.
REG00014	OPN5	GSE92220	.	.	.	.
REG00014	TNPO2	GSE92220	.	.	.	.
REG00014	PCSK9	GSE92220	.	.	.	.
REG00014	CAP2	GSE92220	.	.	.	.
REG00014	ZNF408	GSE92220	.	.	.	.
REG00014	USP33	GSE92220	.	.	.	.
REG00014	USP31	GSE92220	.	.	.	.
REG00014	USP35	GSE92220	.	.	.	.
REG00014	USP36	GSE92220	.	.	.	.
REG00014	USP37	GSE92220	.	.	.	.
REG00014	USP44	GSE92220	.	.	.	.
REG00014	USP34	GSE92220	.	.	.	.
REG00014	USP38	GSE92220	.	.	.	.
REG00014	USP42	GSE92220	.	.	.	.

REG00014	USP40	GSE92220	.	.	.	.
REG00014	USP39	GSE92220	.	.	.	.
REG00014	SNX27	GSE92220	.	.	.	.
REG00014	PAN2	GSE92220	.	.	.	.
REG00014	USP46	GSE92220	.	.	.	.
REG00014	USP47	GSE92220	.	.	.	.
REG00014	USP49	GSE92220	.	.	.	.
REG00014	USP50	GSE92220	.	.	.	.
REG00014	DDX51	GSE92220	.	.	.	.
REG00014	DDX54	GSE92220	.	.	.	.
REG00014	DDX55	GSE92220	.	.	.	.
REG00014	AK7	GSE92220	.	.	.	.
REG00014	ADSSL1	GSE92220	.	.	.	.
REG00014	ANKRD9	GSE92220	.	.	.	.
REG00014	FAM71D	GSE92220	.	.	.	.
REG00014	DEGS2	GSE92220	.	.	.	.
REG00014	SLC25A47	GSE92220	.	.	.	.
REG00014	SLC25A29	GSE92220	.	.	.	.
REG00014	EXOC3L4	GSE92220	.	.	.	.
REG00014	AP3S1	GSE92220	.	.	.	.
REG00014	GLRX5	GSE92220	.	.	.	.
REG00014	NUDT14	GSE92220	.	.	.	.
REG00014	ZNF410	GSE92220	.	.	.	.
REG00014	GPR143	GSE92220	.	.	.	.
REG00014	PLEKHD1	GSE92220	.	.	.	.
REG00014	RAB15	GSE92220	.	.	.	.
REG00014	SLC17A8	GSE92220	.	.	.	.
REG00014	ZFHX2	GSE92220	.	.	.	.
REG00014	CHD8	GSE92220	.	.	.	.
REG00014	ABHD4	GSE92220	.	.	.	.
REG00014	HECTD1	GSE92220	.	.	.	.
REG00014	TOX4	GSE92220	.	.	.	.
REG00014	C14orf93	GSE92220	.	.	.	.
REG00014	HOMEZ	GSE92220	.	.	.	.
REG00014	KHNYN	GSE92220	.	.	.	.
REG00014	GOLT1B	GSE92220	.	.	.	.
REG00014	MYPOP	GSE92220	.	.	.	.
REG00014	JKAMP	GSE92220	.	.	.	.
REG00014	TMEM260	GSE92220	.	.	.	.
REG00014	NRDE2	GSE92220	.	.	.	.
REG00014	CLCN1	GSE92220	.	.	.	.
REG00014	AP5M1	GSE92220	.	.	.	.
REG00014	HFM1	GSE92220	.	.	.	.
REG00014	POLR1C	GSE92220	.	.	.	.
REG00014	SLC35C1	GSE92220	.	.	.	.
REG00014	CLCN2	GSE92220	.	.	.	.
REG00014	FBXO34	GSE92220	.	.	.	.
REG00014	CLCN3	GSE92220	.	.	.	.
REG00014	GPATCH2L	GSE92220	.	.	.	.
REG00014	COX16	GSE92220	.	.	.	.

REG00014	PPP4R3A	GSE92220	.	.	.	.
REG00014	CLCN4	GSE92220	.	.	.	.
REG00014	FCF1	GSE92220	.	.	.	.
REG00014	RPS6KL1	GSE92220	.	.	.	.
REG00014	DCAF5	GSE92220	.	.	.	.
REG00014	DPF1	GSE92220	.	.	.	.
REG00014	DCAF4	GSE92220	.	.	.	.
REG00014	CLCN5	GSE92220	.	.	.	.
REG00014	KLHDC2	GSE92220	.	.	.	.
REG00014	EVL	GSE92220	.	.	.	.
REG00014	CRB3	GSE92220	.	.	.	.
REG00014	FNDC5	GSE92220	.	.	.	.
REG00014	CYP2W1	GSE92220	.	.	.	.
REG00014	RAB2B	GSE92220	.	.	.	.
REG00014	MEPCE	GSE92220	.	.	.	.
REG00014	REM2	GSE92220	.	.	.	.
REG00014	SPRED1	GSE92220	.	.	.	.
REG00014	CLCN7	GSE92220	.	.	.	.
REG00014	AJUBA	GSE92220	.	.	.	.
REG00014	MGRN1	GSE92220	.	.	.	.
REG00014	DCAF11	GSE92220	.	.	.	.
REG00014	NUP58	GSE92220	.	.	.	.
REG00014	SKA3	GSE92220	.	.	.	.
REG00014	LRRC10	GSE92220	.	.	.	.
REG00014	ANKRD10	GSE92220	.	.	.	.
REG00014	ALG5	GSE92220	.	.	.	.
REG00014	NGDN	GSE92220	.	.	.	.
REG00014	CHMP4A	GSE92220	.	.	.	.
REG00014	SDR39U1	GSE92220	.	.	.	.
REG00014	HEATR5A	GSE92220	.	.	.	.
REG00014	DTD2	GSE92220	.	.	.	.
REG00014	NUBPL	GSE92220	.	.	.	.
REG00014	SIPA1L1	GSE92220	.	.	.	.
REG00014	PROSER1	GSE92220	.	.	.	.
REG00014	CAB39	GSE92220	.	.	.	.
REG00014	ABHD13	GSE92220	.	.	.	.
REG00014	FNDC3A	GSE92220	.	.	.	.
REG00014	SLC22A16	GSE92220	.	.	.	.
REG00014	LRCH1	GSE92220	.	.	.	.
REG00014	CHAMP1	GSE92220	.	.	.	.
REG00014	PRPF39	GSE92220	.	.	.	.
REG00014	TRPT1	GSE92220	.	.	.	.
REG00014	SMOC1	GSE92220	.	.	.	.
REG00014	CLDN1	GSE92220	.	.	.	.
REG00014	PSPC1	GSE92220	.	.	.	.
REG00014	UTP14C	GSE92220	.	.	.	.
REG00014	RBM26	GSE92220	.	.	.	.
REG00014	DCUN1D2	GSE92220	.	.	.	.
REG00014	TMCO3	GSE92220	.	.	.	.
REG00014	CLDN10	GSE92220	.	.	.	.

REG00014	POMP	GSE92220	.	.	.	.
REG00014	UPF3A	GSE92220	.	.	.	.
REG00014	SCOC	GSE92220	.	.	.	.
REG00014	G2E3	GSE92220	.	.	.	.
REG00014	CLDN12	GSE92220	.	.	.	.
REG00014	ZDHHC14	GSE92220	.	.	.	.
REG00014	GSKIP	GSE92220	.	.	.	.
REG00014	ZNF839	GSE92220	.	.	.	.
REG00014	PCNX4	GSE92220	.	.	.	.
REG00014	OTUB2	GSE92220	.	.	.	.
REG00014	VCPKMT	GSE92220	.	.	.	.
REG00014	ZC2HC1C	GSE92220	.	.	.	.
REG00014	GON7	GSE92220	.	.	.	.
REG00014	CLDN15	GSE92220	.	.	.	.
REG00014	SPTSSA	GSE92220	.	.	.	.
REG00014	CEP170B	GSE92220	.	.	.	.
REG00014	AREL1	GSE92220	.	.	.	.
REG00014	PLEKHG3	GSE92220	.	.	.	.
REG00014	CIPC	GSE92220	.	.	.	.
REG00014	SHISA2	GSE92220	.	.	.	.
REG00014	ZC3H13	GSE92220	.	.	.	.
REG00014	TEX29	GSE92220	.	.	.	.
REG00014	NDUFA11	GSE92220	.	.	.	.
REG00014	NDUFB11	GSE92220	.	.	.	.
REG00014	SPG21	GSE92220	.	.	.	.
REG00014	SULF1	GSE92220	.	.	.	.
REG00014	SULF2	GSE92220	.	.	.	.
REG00014	ATF7IP2	GSE92220	.	.	.	.
REG00014	CLDN19	GSE92220	.	.	.	.
REG00014	DQX1	GSE92220	.	.	.	.
REG00014	SUMF2	GSE92220	.	.	.	.
REG00014	PDS5B	GSE92220	.	.	.	.
REG00014	LRRC14	GSE92220	.	.	.	.
REG00014	CLDN20	GSE92220	.	.	.	.
REG00014	POLR1D	GSE92220	.	.	.	.
REG00014	MBIP	GSE92220	.	.	.	.
REG00014	ZAR1	GSE92220	.	.	.	.
REG00014	CLDN22	GSE92220	.	.	.	.
REG00014	S100A16	GSE92220	.	.	.	.
REG00014	FBRS	GSE92220	.	.	.	.
REG00014	MTSS1	GSE92220	.	.	.	.
REG00014	MBD6	GSE92220	.	.	.	.
REG00014	WDFY1	GSE92220	.	.	.	.
REG00014	INSIG2	GSE92220	.	.	.	.
REG00014	POLR1B	GSE92220	.	.	.	.
REG00014	CHFR	GSE92220	.	.	.	.
REG00014	TRAF7	GSE92220	.	.	.	.
REG00014	RNF144A	GSE92220	.	.	.	.
REG00014	CLDN4	GSE92220	.	.	.	.
REG00014	SMC5	GSE92220	.	.	.	.

REG00014	SMC6	GSE92220	.	.	.	.
REG00014	LSM7	GSE92220	.	.	.	.
REG00014	LSM8	GSE92220	.	.	.	.
REG00014	BRIP1	GSE92220	.	.	.	.
REG00014	WDFY2	GSE92220	.	.	.	.
REG00014	USP12	GSE92220	.	.	.	.
REG00014	L3HYPDH	GSE92220	.	.	.	.
REG00014	APOPT1	GSE92220	.	.	.	.
REG00014	SETD3	GSE92220	.	.	.	.
REG00014	SLC39A5	GSE92220	.	.	.	.
REG00014	ZC3H14	GSE92220	.	.	.	.
REG00014	CLDN9	GSE92220	.	.	.	.
REG00014	HIST4H4	GSE92220	.	.	.	.
REG00014	BRMS1L	GSE92220	.	.	.	.
REG00014	MBNL3	GSE92220	.	.	.	.
REG00014	TRAM1	GSE92220	.	.	.	.
REG00014	CCNL1	GSE92220	.	.	.	.
REG00014	CCNL2	GSE92220	.	.	.	.
REG00014	CYP20A1	GSE92220	.	.	.	.
REG00014	CYP2R1	GSE92220	.	.	.	.
REG00014	CYP26B1	GSE92220	.	.	.	.
REG00014	SLC35B4	GSE92220	.	.	.	.
REG00014	KCMF1	GSE92220	.	.	.	.
REG00014	VPS39	GSE92220	.	.	.	.
REG00014	UFM1	GSE92220	.	.	.	.
REG00014	LONP2	GSE92220	.	.	.	.
REG00014	SLC23A3	GSE92220	.	.	.	.
REG00014	AIMP2	GSE92220	.	.	.	.
REG00014	PHLPP1	GSE92220	.	.	.	.
REG00014	TDRD5	GSE92220	.	.	.	.
REG00014	MICAL1	GSE92220	.	.	.	.
REG00014	CLIC1	GSE92220	.	.	.	.
REG00014	TNFAIP8L3	GSE92220	.	.	.	.
REG00014	CHD7	GSE92220	.	.	.	.
REG00014	PSME4	GSE92220	.	.	.	.
REG00014	CLIC3	GSE92220	.	.	.	.
REG00014	ZNF417	GSE92220	.	.	.	.
REG00014	SLC37A3	GSE92220	.	.	.	.
REG00014	TXNDC15	GSE92220	.	.	.	.
REG00014	RAPGEF6	GSE92220	.	.	.	.
REG00014	C5orf15	GSE92220	.	.	.	.
REG00014	SLC25A26	GSE92220	.	.	.	.
REG00014	SLC25A25	GSE92220	.	.	.	.
REG00014	H2AFV	GSE92220	.	.	.	.
REG00014	MCCD1	GSE92220	.	.	.	.
REG00014	IQGAP3	GSE92220	.	.	.	.
REG00014	PDLIM1	GSE92220	.	.	.	.
REG00014	PHF8	GSE92220	.	.	.	.
REG00014	CNDP1	GSE92220	.	.	.	.
REG00014	ANO9	GSE92220	.	.	.	.

REG00014	CLK1	GSE92220	.	.	.	.
REG00014	CLK2	GSE92220	.	.	.	.
REG00014	ELP3	GSE92220	.	.	.	.
REG00014	APBB3	GSE92220	.	.	.	.
REG00014	SLTM	GSE92220	.	.	.	.
REG00014	CLK3	GSE92220	.	.	.	.
REG00014	ZDHHC18	GSE92220	.	.	.	.
REG00014	ZDHHC16	GSE92220	.	.	.	.
REG00014	MID1IP1	GSE92220	.	.	.	.
REG00014	KLC2	GSE92220	.	.	.	.
REG00014	KLC3	GSE92220	.	.	.	.
REG00014	ZBED4	GSE92220	.	.	.	.
REG00014	SCFD1	GSE92220	.	.	.	.
REG00014	ARMC9	GSE92220	.	.	.	.
REG00014	SYVN1	GSE92220	.	.	.	.
REG00014	IPMK	GSE92220	.	.	.	.
REG00014	CLN3	GSE92220	.	.	.	.
REG00014	SESN2	GSE92220	.	.	.	.
REG00014	FANCL	GSE92220	.	.	.	.
REG00014	ZDHHC20	GSE92220	.	.	.	.
REG00014	WDFY3	GSE92220	.	.	.	.
REG00014	PLEKHF2	GSE92220	.	.	.	.
REG00014	ZFYVE21	GSE92220	.	.	.	.
REG00014	ZFYVE26	GSE92220	.	.	.	.
REG00014	PDLIM3	GSE92220	.	.	.	.
REG00014	TUBA1C	GSE92220	.	.	.	.
REG00014	CLN6	GSE92220	.	.	.	.
REG00014	TUBB4B	GSE92220	.	.	.	.
REG00014	TUBB3	GSE92220	.	.	.	.
REG00014	TUBB6	GSE92220	.	.	.	.
REG00014	TUBB	GSE92220	.	.	.	.
REG00014	RHBDF2	GSE92220	.	.	.	.
REG00014	ATG4B	GSE92220	.	.	.	.
REG00014	SLC35A5	GSE92220	.	.	.	.
REG00014	CYBRD1	GSE92220	.	.	.	.
REG00014	SLC35B1	GSE92220	.	.	.	.
REG00014	SLC35D2	GSE92220	.	.	.	.
REG00014	ADAM30	GSE92220	.	.	.	.
REG00014	CLNS1A	GSE92220	.	.	.	.
REG00014	SLC35D1	GSE92220	.	.	.	.
REG00014	SLC35E1	GSE92220	.	.	.	.
REG00014	ZNF428	GSE92220	.	.	.	.
REG00014	ZNF436	GSE92220	.	.	.	.
REG00014	KDM3A	GSE92220	.	.	.	.
REG00014	PHF12	GSE92220	.	.	.	.
REG00014	CATSPER3	GSE92220	.	.	.	.
REG00014	TPCN2	GSE92220	.	.	.	.
REG00014	CABP7	GSE92220	.	.	.	.
REG00014	CLPP	GSE92220	.	.	.	.
REG00014	FOXP4	GSE92220	.	.	.	.

REG00014	FNBP1L	GSE92220	.	.	.	.
REG00014	WDR24	GSE92220	.	.	.	.
REG00014	RNF145	GSE92220	.	.	.	.
REG00014	THAP2	GSE92220	.	.	.	.
REG00014	THAP3	GSE92220	.	.	.	.
REG00014	THAP1	GSE92220	.	.	.	.
REG00014	SLC39A14	GSE92220	.	.	.	.
REG00014	SLC39A13	GSE92220	.	.	.	.
REG00014	SLC39A10	GSE92220	.	.	.	.
REG00014	SLC35E2	GSE92220	.	.	.	.
REG00014	SLC35E3	GSE92220	.	.	.	.
REG00014	ZBTB2	GSE92220	.	.	.	.
REG00014	CLPTM1	GSE92220	.	.	.	.
REG00014	RPF2	GSE92220	.	.	.	.
REG00014	GTF3C6	GSE92220	.	.	.	.
REG00014	WRNIP1	GSE92220	.	.	.	.
REG00014	CASKIN1	GSE92220	.	.	.	.
REG00014	PAK1IP1	GSE92220	.	.	.	.
REG00014	AGPAT5	GSE92220	.	.	.	.
REG00014	LRIG2	GSE92220	.	.	.	.
REG00014	BCOR	GSE92220	.	.	.	.
REG00014	CLTA	GSE92220	.	.	.	.
REG00014	EAF1	GSE92220	.	.	.	.
REG00014	DZIP1	GSE92220	.	.	.	.
REG00014	AFMID	GSE92220	.	.	.	.
REG00014	BLOC1S3	GSE92220	.	.	.	.
REG00014	CLTC	GSE92220	.	.	.	.
REG00014	HACD4	GSE92220	.	.	.	.
REG00014	CLTCL1	GSE92220	.	.	.	.
REG00014	SENP6	GSE92220	.	.	.	.
REG00014	TOMM20	GSE92220	.	.	.	.
REG00014	CLU	GSE92220	.	.	.	.
REG00014	FOXQ1	GSE92220	.	.	.	.
REG00014	TMEM14C	GSE92220	.	.	.	.
REG00014	VTA1	GSE92220	.	.	.	.
REG00014	SDHAF4	GSE92220	.	.	.	.
REG00014	TMEM181	GSE92220	.	.	.	.
REG00014	SCAF8	GSE92220	.	.	.	.
REG00014	ANKS1A	GSE92220	.	.	.	.
REG00014	WTIP	GSE92220	.	.	.	.
REG00014	SMYD2	GSE92220	.	.	.	.
REG00014	ANKMY1	GSE92220	.	.	.	.
REG00014	FAM184A	GSE92220	.	.	.	.
REG00014	RWDD1	GSE92220	.	.	.	.
REG00014	C6orf62	GSE92220	.	.	.	.
REG00014	AATK	GSE92220	.	.	.	.
REG00014	MSS51	GSE92220	.	.	.	.
REG00014	PLXDC2	GSE92220	.	.	.	.
REG00014	NHSL1	GSE92220	.	.	.	.
REG00014	AARS2	GSE92220	.	.	.	.

REG00014	YIPF3	GSE92220	.	.	.	.
REG00014	CUL7	GSE92220	.	.	.	.
REG00014	ARHGAP18	GSE92220	.	.	.	.
REG00014	ZNF446	GSE92220	.	.	.	.
REG00014	NOL7	GSE92220	.	.	.	.
REG00014	NUS1	GSE92220	.	.	.	.
REG00014	PITPNM2	GSE92220	.	.	.	.
REG00014	PITPNC1	GSE92220	.	.	.	.
REG00014	CDKAL1	GSE92220	.	.	.	.
REG00014	MFSD4B	GSE92220	.	.	.	.
REG00014	MTHFD1L	GSE92220	.	.	.	.
REG00014	RSPH9	GSE92220	.	.	.	.
REG00014	SERAC1	GSE92220	.	.	.	.
REG00014	TBC1D7	GSE92220	.	.	.	.
REG00014	RNF121	GSE92220	.	.	.	.
REG00014	MANEA	GSE92220	.	.	.	.
REG00014	TXNDC5	GSE92220	.	.	.	.
REG00014	GINM1	GSE92220	.	.	.	.
REG00014	CMTR1	GSE92220	.	.	.	.
REG00014	HDDC2	GSE92220	.	.	.	.
REG00014	TRMT11	GSE92220	.	.	.	.
REG00014	NCOA7	GSE92220	.	.	.	.
REG00014	SEC63	GSE92220	.	.	.	.
REG00014	FAM135A	GSE92220	.	.	.	.
REG00014	MIB1	GSE92220	.	.	.	.
REG00014	SCD5	GSE92220	.	.	.	.
REG00014	ZNF451	GSE92220	.	.	.	.
REG00014	QKI	GSE92220	.	.	.	.
REG00014	CUTA	GSE92220	.	.	.	.
REG00014	SFT2D1	GSE92220	.	.	.	.
REG00014	SLC22A23	GSE92220	.	.	.	.
REG00014	PSMG4	GSE92220	.	.	.	.
REG00014	GLTSCR1L	GSE92220	.	.	.	.
REG00014	DLK2	GSE92220	.	.	.	.
REG00014	C6orf89	GSE92220	.	.	.	.
REG00014	MTFR2	GSE92220	.	.	.	.
REG00014	ECT2L	GSE92220	.	.	.	.
REG00014	DSE	GSE92220	.	.	.	.
REG00014	RCN3	GSE92220	.	.	.	.
REG00014	ZMYND19	GSE92220	.	.	.	.
REG00014	RNF123	GSE92220	.	.	.	.
REG00014	PRR3	GSE92220	.	.	.	.
REG00014	RNF126	GSE92220	.	.	.	.
REG00014	RNF128	GSE92220	.	.	.	.
REG00014	GTF2H5	GSE92220	.	.	.	.
REG00014	RAP2C	GSE92220	.	.	.	.
REG00014	RHOT2	GSE92220	.	.	.	.
REG00014	ADAT2	GSE92220	.	.	.	.
REG00014	LTV1	GSE92220	.	.	.	.
REG00014	ZC2HC1B	GSE92220	.	.	.	.

REG00014	RMND1	GSE92220	.	.	.	.
REG00014	SAMD5	GSE92220	.	.	.	.
REG00014	SUMO4	GSE92220	.	.	.	.
REG00014	NUP43	GSE92220	.	.	.	.
REG00014	PNLDC1	GSE92220	.	.	.	.
REG00014	ATAT1	GSE92220	.	.	.	.
REG00014	FA2H	GSE92220	.	.	.	.
REG00014	HCFC1R1	GSE92220	.	.	.	.
REG00014	PBXIP1	GSE92220	.	.	.	.
REG00014	IPCEF1	GSE92220	.	.	.	.
REG00014	WDR26	GSE92220	.	.	.	.
REG00014	C6orf106	GSE92220	.	.	.	.
REG00014	UHRF1BP1	GSE92220	.	.	.	.
REG00014	DNPH1	GSE92220	.	.	.	.
REG00014	ICK	GSE92220	.	.	.	.
REG00014	PNISR	GSE92220	.	.	.	.
REG00014	UQCC2	GSE92220	.	.	.	.
REG00014	TMEM217	GSE92220	.	.	.	.
REG00014	CCDC167	GSE92220	.	.	.	.
REG00014	LEMD2	GSE92220	.	.	.	.
REG00014	PI16	GSE92220	.	.	.	.
REG00014	PNPLA1	GSE92220	.	.	.	.
REG00014	C6orf120	GSE92220	.	.	.	.
REG00014	WDR27	GSE92220	.	.	.	.
REG00014	LHFPL5	GSE92220	.	.	.	.
REG00014	OARD1	GSE92220	.	.	.	.
REG00014	CAPRIN2	GSE92220	.	.	.	.
REG00014	C1RL	GSE92220	.	.	.	.
REG00014	ANKRD13A	GSE92220	.	.	.	.
REG00014	GRWD1	GSE92220	.	.	.	.
REG00014	ADCY10	GSE92220	.	.	.	.
REG00014	KCTD2	GSE92220	.	.	.	.
REG00014	EPS8L1	GSE92220	.	.	.	.
REG00014	EPS8L2	GSE92220	.	.	.	.
REG00014	EPS8L3	GSE92220	.	.	.	.
REG00014	AACS	GSE92220	.	.	.	.
REG00014	CROCC	GSE92220	.	.	.	.
REG00014	RPP21	GSE92220	.	.	.	.
REG00014	C6orf136	GSE92220	.	.	.	.
REG00014	ADPRHL2	GSE92220	.	.	.	.
REG00014	KCTD3	GSE92220	.	.	.	.
REG00014	ELOVL5	GSE92220	.	.	.	.
REG00014	COPS9	GSE92220	.	.	.	.
REG00014	ANKRD11	GSE92220	.	.	.	.
REG00014	DYM	GSE92220	.	.	.	.
REG00014	SKOR1	GSE92220	.	.	.	.
REG00014	NEU4	GSE92220	.	.	.	.
REG00014	STK32C	GSE92220	.	.	.	.
REG00014	SNX13	GSE92220	.	.	.	.
REG00014	RNF146	GSE92220	.	.	.	.

REG00014	PTCHD4	GSE92220	.	.	.	.
REG00014	SBF2	GSE92220	.	.	.	.
REG00014	TICAM2	GSE92220	.	.	.	.
REG00014	CORO6	GSE92220	.	.	.	.
REG00014	PXDC1	GSE92220	.	.	.	.
REG00014	SNRNP48	GSE92220	.	.	.	.
REG00014	RPL7L1	GSE92220	.	.	.	.
REG00014	STK40	GSE92220	.	.	.	.
REG00014	RRP36	GSE92220	.	.	.	.
REG00014	LRRC73	GSE92220	.	.	.	.
REG00014	OOEP	GSE92220	.	.	.	.
REG00014	TMEM14B	GSE92220	.	.	.	.
REG00014	SCML4	GSE92220	.	.	.	.
REG00014	SMIM8	GSE92220	.	.	.	.
REG00014	RARS2	GSE92220	.	.	.	.
REG00014	AKIRIN2	GSE92220	.	.	.	.
REG00014	PM20D2	GSE92220	.	.	.	.
REG00014	AIFM2	GSE92220	.	.	.	.
REG00014	UPK3B	GSE92220	.	.	.	.
REG00014	RC3H2	GSE92220	.	.	.	.
REG00014	INPP5E	GSE92220	.	.	.	.
REG00014	VKORC1L1	GSE92220	.	.	.	.
REG00014	KIF12	GSE92220	.	.	.	.
REG00014	ACAD9	GSE92220	.	.	.	.
REG00014	ATG16L1	GSE92220	.	.	.	.
REG00014	ADAM8	GSE92220	.	.	.	.
REG00014	DUS4L	GSE92220	.	.	.	.
REG00014	DHRS7	GSE92220	.	.	.	.
REG00014	TM9SF3	GSE92220	.	.	.	.
REG00014	SNX24	GSE92220	.	.	.	.
REG00014	GTPBP4	GSE92220	.	.	.	.
REG00014	SYCP2L	GSE92220	.	.	.	.
REG00014	NT5DC1	GSE92220	.	.	.	.
REG00014	PPIL6	GSE92220	.	.	.	.
REG00014	TSPYL4	GSE92220	.	.	.	.
REG00014	CNN2	GSE92220	.	.	.	.
REG00014	THEMIS	GSE92220	.	.	.	.
REG00014	CNN3	GSE92220	.	.	.	.
REG00014	CNP	GSE92220	.	.	.	.
REG00014	TTL	GSE92220	.	.	.	.
REG00014	FAM19A5	GSE92220	.	.	.	.
REG00014	SESN1	GSE92220	.	.	.	.
REG00014	ADAM9	GSE92220	.	.	.	.
REG00014	SLC35B3	GSE92220	.	.	.	.
REG00014	EPN1	GSE92220	.	.	.	.
REG00014	SAFB2	GSE92220	.	.	.	.
REG00014	MPC1	GSE92220	.	.	.	.
REG00014	AIG1	GSE92220	.	.	.	.
REG00014	LUC7L2	GSE92220	.	.	.	.
REG00014	LNPK	GSE92220	.	.	.	.

REG00014	CTDSP1	GSE92220	.	.	.	.
REG00014	TNS3	GSE92220	.	.	.	.
REG00014	ZNF460	GSE92220	.	.	.	.
REG00014	SATB2	GSE92220	.	.	.	.
REG00014	TSPAN9	GSE92220	.	.	.	.
REG00014	VARS2	GSE92220	.	.	.	.
REG00014	TSPAN13	GSE92220	.	.	.	.
REG00014	CHCHD2	GSE92220	.	.	.	.
REG00014	UBE2D4	GSE92220	.	.	.	.
REG00014	BAIAP2L1	GSE92220	.	.	.	.
REG00014	BMP8A	GSE92220	.	.	.	.
REG00014	ORAI2	GSE92220	.	.	.	.
REG00014	SIK2	GSE92220	.	.	.	.
REG00014	RNASET2	GSE92220	.	.	.	.
REG00014	ZNHIT1	GSE92220	.	.	.	.
REG00014	CNTF	GSE92220	.	.	.	.
REG00014	GET4	GSE92220	.	.	.	.
REG00014	TSC22D4	GSE92220	.	.	.	.
REG00014	TMEM120A	GSE92220	.	.	.	.
REG00014	RNF216	GSE92220	.	.	.	.
REG00014	RAD9B	GSE92220	.	.	.	.
REG00014	BRAT1	GSE92220	.	.	.	.
REG00014	C7orf26	GSE92220	.	.	.	.
REG00014	LRRC61	GSE92220	.	.	.	.
REG00014	ARMC10	GSE92220	.	.	.	.
REG00014	TMEM243	GSE92220	.	.	.	.
REG00014	GPR146	GSE92220	.	.	.	.
REG00014	MALSU1	GSE92220	.	.	.	.
REG00014	ARHGEF17	GSE92220	.	.	.	.
REG00014	B3GNTL1	GSE92220	.	.	.	.
REG00014	IRF2BP1	GSE92220	.	.	.	.
REG00014	IRF2BP2	GSE92220	.	.	.	.
REG00014	ANTXR2	GSE92220	.	.	.	.
REG00014	CPEB4	GSE92220	.	.	.	.
REG00014	COPS6	GSE92220	.	.	.	.
REG00014	KBTBD2	GSE92220	.	.	.	.
REG00014	TMEM60	GSE92220	.	.	.	.
REG00014	CHST13	GSE92220	.	.	.	.
REG00014	ZNF853	GSE92220	.	.	.	.
REG00014	COCH	GSE92220	.	.	.	.
REG00014	VPS13B	GSE92220	.	.	.	.
REG00014	COL10A1	GSE92220	.	.	.	.
REG00014	WDR60	GSE92220	.	.	.	.
REG00014	COL11A2	GSE92220	.	.	.	.
REG00014	FBXL18	GSE92220	.	.	.	.
REG00014	SNX25	GSE92220	.	.	.	.
REG00014	PRKRIP1	GSE92220	.	.	.	.
REG00014	TMEM209	GSE92220	.	.	.	.
REG00014	NCAPG2	GSE92220	.	.	.	.
REG00014	CHCHD3	GSE92220	.	.	.	.

REG00014	HERPUD2	GSE92220	.	.	.	.
REG00014	PARP12	GSE92220	.	.	.	.
REG00014	COL16A1	GSE92220	.	.	.	.
REG00014	UBN2	GSE92220	.	.	.	.
REG00014	ZNF746	GSE92220	.	.	.	.
REG00014	COL18A1	GSE92220	.	.	.	.
REG00014	KCTD7	GSE92220	.	.	.	.
REG00014	ZSCAN25	GSE92220	.	.	.	.
REG00014	LRGUK	GSE92220	.	.	.	.
REG00014	CLEC2L	GSE92220	.	.	.	.
REG00014	COL2A1	GSE92220	.	.	.	.
REG00014	LSMEM1	GSE92220	.	.	.	.
REG00014	COL4A5	GSE92220	.	.	.	.
REG00014	VMA21	GSE92220	.	.	.	.
REG00014	COL6A1	GSE92220	.	.	.	.
REG00014	COL7A1	GSE92220	.	.	.	.
REG00014	FAM20C	GSE92220	.	.	.	.
REG00014	MUSTN1	GSE92220	.	.	.	.
REG00014	SLC5A9	GSE92220	.	.	.	.
REG00014	COL8A2	GSE92220	.	.	.	.
REG00014	COL9A2	GSE92220	.	.	.	.
REG00014	COL9A3	GSE92220	.	.	.	.
REG00014	SCRN1	GSE92220	.	.	.	.
REG00014	KLHDC10	GSE92220	.	.	.	.
REG00014	AP5Z1	GSE92220	.	.	.	.
REG00014	PTCD1	GSE92220	.	.	.	.
REG00014	COBL	GSE92220	.	.	.	.
REG00014	TCAF1	GSE92220	.	.	.	.
REG00014	PHF14	GSE92220	.	.	.	.
REG00014	AHCYL2	GSE92220	.	.	.	.
REG00014	DPY19L1	GSE92220	.	.	.	.
REG00014	KIAA0895	GSE92220	.	.	.	.
REG00014	NUDCD3	GSE92220	.	.	.	.
REG00014	STRIP2	GSE92220	.	.	.	.
REG00014	ATXN7L1	GSE92220	.	.	.	.
REG00014	ZNF777	GSE92220	.	.	.	.
REG00014	TECPR1	GSE92220	.	.	.	.
REG00014	KIAA1549	GSE92220	.	.	.	.
REG00014	TTYH3	GSE92220	.	.	.	.
REG00014	EEPD1	GSE92220	.	.	.	.
REG00014	KDM7A	GSE92220	.	.	.	.
REG00014	ZMIZ2	GSE92220	.	.	.	.
REG00014	AMZ1	GSE92220	.	.	.	.
REG00014	KCTD13	GSE92220	.	.	.	.
REG00014	BHLHA15	GSE92220	.	.	.	.
REG00014	COMP	GSE92220	.	.	.	.
REG00014	COMT	GSE92220	.	.	.	.
REG00014	TMED4	GSE92220	.	.	.	.
REG00014	COPB1	GSE92220	.	.	.	.
REG00014	COPB2	GSE92220	.	.	.	.

REG00014	COPE	GSE92220	.	.	.	.
REG00014	KLF6	GSE92220	.	.	.	.
REG00014	COPG1	GSE92220	.	.	.	.
REG00014	COPS3	GSE92220	.	.	.	.
REG00014	COPS5	GSE92220	.	.	.	.
REG00014	TMEM106B	GSE92220	.	.	.	.
REG00014	ATG9A	GSE92220	.	.	.	.
REG00014	C7orf50	GSE92220	.	.	.	.
REG00014	ZC3HAV1L	GSE92220	.	.	.	.
REG00014	C7orf49	GSE92220	.	.	.	.
REG00014	COQ7	GSE92220	.	.	.	.
REG00014	ANKRD61	GSE92220	.	.	.	.
REG00014	MED13	GSE92220	.	.	.	.
REG00014	ADAR	GSE92220	.	.	.	.
REG00014	CORO1B	GSE92220	.	.	.	.
REG00014	CORO1C	GSE92220	.	.	.	.
REG00014	CORT	GSE92220	.	.	.	.
REG00014	ADARB1	GSE92220	.	.	.	.
REG00014	COX4I1	GSE92220	.	.	.	.
REG00014	NOMO2	GSE92220	.	.	.	.
REG00014	COX5A	GSE92220	.	.	.	.
REG00014	COX5B	GSE92220	.	.	.	.
REG00014	COX6A1	GSE92220	.	.	.	.
REG00014	FEZF1	GSE92220	.	.	.	.
REG00014	ADAT1	GSE92220	.	.	.	.
REG00014	COX6B1	GSE92220	.	.	.	.
REG00014	IQCA1L	GSE92220	.	.	.	.
REG00014	COX6C	GSE92220	.	.	.	.
REG00014	COX7A1	GSE92220	.	.	.	.
REG00014	COX7A2	GSE92220	.	.	.	.
REG00014	COX7A2L	GSE92220	.	.	.	.
REG00014	COX7B	GSE92220	.	.	.	.
REG00014	ZCCHC2	GSE92220	.	.	.	.
REG00014	COX7C	GSE92220	.	.	.	.
REG00014	GMPPA	GSE92220	.	.	.	.
REG00014	KLHL13	GSE92220	.	.	.	.
REG00014	GMPPB	GSE92220	.	.	.	.
REG00014	KBTBD3	GSE92220	.	.	.	.
REG00014	SYMPK	GSE92220	.	.	.	.
REG00014	COX8A	GSE92220	.	.	.	.
REG00014	CP	GSE92220	.	.	.	.
REG00014	POGLUT1	GSE92220	.	.	.	.
REG00014	PDLIM7	GSE92220	.	.	.	.
REG00014	HSD17B11	GSE92220	.	.	.	.
REG00014	MED13L	GSE92220	.	.	.	.
REG00014	THRAP3	GSE92220	.	.	.	.
REG00014	PEX26	GSE92220	.	.	.	.
REG00014	KDM4A	GSE92220	.	.	.	.
REG00014	SLC10A4	GSE92220	.	.	.	.
REG00014	JADE3	GSE92220	.	.	.	.

REG00014	JADE2	GSE92220	.	.	.	.
REG00014	COL27A1	GSE92220	.	.	.	.
REG00014	TMC4	GSE92220	.	.	.	.
REG00014	ABAT	GSE92220	.	.	.	.
REG00014	CPD	GSE92220	.	.	.	.
REG00014	CYB561A3	GSE92220	.	.	.	.
REG00014	FAM20A	GSE92220	.	.	.	.
REG00014	FAM20B	GSE92220	.	.	.	.
REG00014	ZNF280B	GSE92220	.	.	.	.
REG00014	SAMD4A	GSE92220	.	.	.	.
REG00014	GNPTG	GSE92220	.	.	.	.
REG00014	CPE	GSE92220	.	.	.	.
REG00014	MED30	GSE92220	.	.	.	.
REG00014	CNKSR3	GSE92220	.	.	.	.
REG00014	BEND3	GSE92220	.	.	.	.
REG00014	NANOS1	GSE92220	.	.	.	.
REG00014	TM7SF3	GSE92220	.	.	.	.
REG00014	ARL5B	GSE92220	.	.	.	.
REG00014	RTN4RL2	GSE92220	.	.	.	.
REG00014	BRK1	GSE92220	.	.	.	.
REG00014	BCAN	GSE92220	.	.	.	.
REG00014	TRAPPC6B	GSE92220	.	.	.	.
REG00014	TRAPPC5	GSE92220	.	.	.	.
REG00014	MED29	GSE92220	.	.	.	.
REG00014	OSGEPL1	GSE92220	.	.	.	.
REG00014	OTUB1	GSE92220	.	.	.	.
REG00014	RHBDD1	GSE92220	.	.	.	.
REG00014	RHBDD2	GSE92220	.	.	.	.
REG00014	ARHGAP33	GSE92220	.	.	.	.
REG00014	SLC43A2	GSE92220	.	.	.	.
REG00014	SLC13A5	GSE92220	.	.	.	.
REG00014	CPLX1	GSE92220	.	.	.	.
REG00014	SLC15A4	GSE92220	.	.	.	.
REG00014	SLC5A11	GSE92220	.	.	.	.
REG00014	SLC29A4	GSE92220	.	.	.	.
REG00014	FGFR1OP2	GSE92220	.	.	.	.
REG00014	CPLX2	GSE92220	.	.	.	.
REG00014	ELL	GSE92220	.	.	.	.
REG00014	SENP2	GSE92220	.	.	.	.
REG00014	CPN1	GSE92220	.	.	.	.
REG00014	CPN2	GSE92220	.	.	.	.
REG00014	NAT9	GSE92220	.	.	.	.
REG00014	RNF149	GSE92220	.	.	.	.
REG00014	CPNE1	GSE92220	.	.	.	.
REG00014	MAFA	GSE92220	.	.	.	.
REG00014	UNC13D	GSE92220	.	.	.	.
REG00014	PLCXD1	GSE92220	.	.	.	.
REG00014	CPNE2	GSE92220	.	.	.	.
REG00014	FERMT3	GSE92220	.	.	.	.
REG00014	SLC5A10	GSE92220	.	.	.	.

REG00014	U2AF2	GSE92220	.	.	.	.
REG00014	PNRC2	GSE92220	.	.	.	.
REG00014	ALG2	GSE92220	.	.	.	.
REG00014	CPNE3	GSE92220	.	.	.	.
REG00014	SAT2	GSE92220	.	.	.	.
REG00014	ALG8	GSE92220	.	.	.	.
REG00014	C14orf166	GSE92220	.	.	.	.
REG00014	PODN	GSE92220	.	.	.	.
REG00014	ISM2	GSE92220	.	.	.	.
REG00014	KEAP1	GSE92220	.	.	.	.
REG00014	ZNF761	GSE92220	.	.	.	.
REG00014	CPNE5	GSE92220	.	.	.	.
REG00014	CLINT1	GSE92220	.	.	.	.
REG00014	THAP4	GSE92220	.	.	.	.
REG00014	THAP5	GSE92220	.	.	.	.
REG00014	THAP11	GSE92220	.	.	.	.
REG00014	CYP4V2	GSE92220	.	.	.	.
REG00014	HBP1	GSE92220	.	.	.	.
REG00014	RIF1	GSE92220	.	.	.	.
REG00014	CPOX	GSE92220	.	.	.	.
REG00014	PIGW	GSE92220	.	.	.	.
REG00014	EXOC7	GSE92220	.	.	.	.
REG00014	PIGO	GSE92220	.	.	.	.
REG00014	GCC2	GSE92220	.	.	.	.
REG00014	CPS1	GSE92220	.	.	.	.
REG00014	UBA5	GSE92220	.	.	.	.
REG00014	SERINC2	GSE92220	.	.	.	.
REG00014	GALNT16	GSE92220	.	.	.	.
REG00014	KCTD10	GSE92220	.	.	.	.
REG00014	HSD17B14	GSE92220	.	.	.	.
REG00014	RBM25	GSE92220	.	.	.	.
REG00014	MOGAT2	GSE92220	.	.	.	.
REG00014	MOGAT3	GSE92220	.	.	.	.
REG00014	CPSF2	GSE92220	.	.	.	.
REG00014	DGAT2L6	GSE92220	.	.	.	.
REG00014	AWAT2	GSE92220	.	.	.	.
REG00014	CPSF4	GSE92220	.	.	.	.
REG00014	CPT1A	GSE92220	.	.	.	.
REG00014	CPT1B	GSE92220	.	.	.	.
REG00014	WAPL	GSE92220	.	.	.	.
REG00014	C10orf62	GSE92220	.	.	.	.
REG00014	KCTD14	GSE92220	.	.	.	.
REG00014	GJD4	GSE92220	.	.	.	.
REG00014	KCTD15	GSE92220	.	.	.	.
REG00014	PBLD	GSE92220	.	.	.	.
REG00014	TSPAN14	GSE92220	.	.	.	.
REG00014	PRAP1	GSE92220	.	.	.	.
REG00014	SLC25A51	GSE92220	.	.	.	.
REG00014	SLC25A52	GSE92220	.	.	.	.
REG00014	ZFP90	GSE92220	.	.	.	.

REG00014	ZNF276	GSE92220	.	.	.	.
REG00014	ACBD4	GSE92220	.	.	.	.
REG00014	ACBD6	GSE92220	.	.	.	.
REG00014	CCNY	GSE92220	.	.	.	.
REG00014	C10orf10	GSE92220	.	.	.	.
REG00014	CCAR2	GSE92220	.	.	.	.
REG00014	MFSD14A	GSE92220	.	.	.	.
REG00014	ST6GALNAC6	GSE92220	.	.	.	.
REG00014	ARPC5L	GSE92220	.	.	.	.
REG00014	NTMT1	GSE92220	.	.	.	.
REG00014	GAPVD1	GSE92220	.	.	.	.
REG00014	MFSD14B	GSE92220	.	.	.	.
REG00014	MYCBP2	GSE92220	.	.	.	.
REG00014	FAM120AOS	GSE92220	.	.	.	.
REG00014	PHYHD1	GSE92220	.	.	.	.
REG00014	FREM1	GSE92220	.	.	.	.
REG00014	SEC11C	GSE92220	.	.	.	.
REG00014	SPCS1	GSE92220	.	.	.	.
REG00014	C10orf11	GSE92220	.	.	.	.
REG00014	DOLK	GSE92220	.	.	.	.
REG00014	ECHDC2	GSE92220	.	.	.	.
REG00014	TMBIM1	GSE92220	.	.	.	.
REG00014	WASHC2C	GSE92220	.	.	.	.
REG00014	KIF1BP	GSE92220	.	.	.	.
REG00014	CRAT	GSE92220	.	.	.	.
REG00014	KIAA1958	GSE92220	.	.	.	.
REG00014	FGFBP3	GSE92220	.	.	.	.
REG00014	C9orf40	GSE92220	.	.	.	.
REG00014	CCNJ	GSE92220	.	.	.	.
REG00014	FAM25A	GSE92220	.	.	.	.
REG00014	NUTM2A	GSE92220	.	.	.	.
REG00014	ZNF485	GSE92220	.	.	.	.
REG00014	NUTM2B	GSE92220	.	.	.	.
REG00014	NUTM2G	GSE92220	.	.	.	.
REG00014	CREB1	GSE92220	.	.	.	.
REG00014	ASAH2B	GSE92220	.	.	.	.
REG00014	AGAP6	GSE92220	.	.	.	.
REG00014	SLC25A28	GSE92220	.	.	.	.
REG00014	TMEM236	GSE92220	.	.	.	.
REG00014	DOCK5	GSE92220	.	.	.	.
REG00014	CREBBP	GSE92220	.	.	.	.
REG00014	FOXK1	GSE92220	.	.	.	.
REG00014	DUPD1	GSE92220	.	.	.	.
REG00014	FAM208B	GSE92220	.	.	.	.
REG00014	ATF6B	GSE92220	.	.	.	.
REG00014	FAM122A	GSE92220	.	.	.	.
REG00014	CALHM2	GSE92220	.	.	.	.
REG00014	CREBL2	GSE92220	.	.	.	.
REG00014	BMS1	GSE92220	.	.	.	.
REG00014	ADO	GSE92220	.	.	.	.

REG00014	CREG1	GSE92220	.	.	.	.
REG00014	WBP1L	GSE92220	.	.	.	.
REG00014	BEND7	GSE92220	.	.	.	.
REG00014	CHCHD1	GSE92220	.	.	.	.
REG00014	FAM171A1	GSE92220	.	.	.	.
REG00014	ZSWIM8	GSE92220	.	.	.	.
REG00014	C9orf43	GSE92220	.	.	.	.
REG00014	COBLL1	GSE92220	.	.	.	.
REG00014	MORC3	GSE92220	.	.	.	.
REG00014	MORC2	GSE92220	.	.	.	.
REG00014	ANKRD17	GSE92220	.	.	.	.
REG00014	HOOK3	GSE92220	.	.	.	.
REG00014	CRIM1	GSE92220	.	.	.	.
REG00014	ARL9	GSE92220	.	.	.	.
REG00014	VPS13C	GSE92220	.	.	.	.
REG00014	ADCY5	GSE92220	.	.	.	.
REG00014	TBC1D10A	GSE92220	.	.	.	.
REG00014	CRIP2	GSE92220	.	.	.	.
REG00014	SLC35F2	GSE92220	.	.	.	.
REG00014	SLC35F5	GSE92220	.	.	.	.
REG00014	GPR153	GSE92220	.	.	.	.
REG00014	CRK	GSE92220	.	.	.	.
REG00014	IGFLR1	GSE92220	.	.	.	.
REG00014	GPR151	GSE92220	.	.	.	.
REG00014	CRKL	GSE92220	.	.	.	.
REG00014	TTC37	GSE92220	.	.	.	.
REG00014	CRLF1	GSE92220	.	.	.	.
REG00014	NIPSNAP3B	GSE92220	.	.	.	.
REG00014	ANKRD18A	GSE92220	.	.	.	.
REG00014	CRMP1	GSE92220	.	.	.	.
REG00014	TIMM50	GSE92220	.	.	.	.
REG00014	GNE	GSE92220	.	.	.	.
REG00014	VKORC1	GSE92220	.	.	.	.
REG00014	SH3PXD2A	GSE92220	.	.	.	.
REG00014	IER5L	GSE92220	.	.	.	.
REG00014	PLPP6	GSE92220	.	.	.	.
REG00014	DCAF10	GSE92220	.	.	.	.
REG00014	GPR157	GSE92220	.	.	.	.
REG00014	GSDMB	GSE92220	.	.	.	.
REG00014	GPR160	GSE92220	.	.	.	.
REG00014	GPR161	GSE92220	.	.	.	.
REG00014	DIXDC1	GSE92220	.	.	.	.
REG00014	PHLDB1	GSE92220	.	.	.	.
REG00014	ADCY6	GSE92220	.	.	.	.
REG00014	MED14	GSE92220	.	.	.	.
REG00014	ERMP1	GSE92220	.	.	.	.
REG00014	TTC39B	GSE92220	.	.	.	.
REG00014	ZNF492	GSE92220	.	.	.	.
REG00014	REEP3	GSE92220	.	.	.	.
REG00014	ZNF496	GSE92220	.	.	.	.

REG00014	ZBTB45	GSE92220	.	.	.	.
REG00014	ZNF500	GSE92220	.	.	.	.
REG00014	MED23	GSE92220	.	.	.	.
REG00014	CREB3L2	GSE92220	.	.	.	.
REG00014	CERCAM	GSE92220	.	.	.	.
REG00014	ARHGAP19	GSE92220	.	.	.	.
REG00014	ARHGAP21	GSE92220	.	.	.	.
REG00014	FAM107B	GSE92220	.	.	.	.
REG00014	CACUL1	GSE92220	.	.	.	.
REG00014	PROSER2	GSE92220	.	.	.	.
REG00014	PPP2R2D	GSE92220	.	.	.	.
REG00014	NECAB2	GSE92220	.	.	.	.
REG00014	CERS4	GSE92220	.	.	.	.
REG00014	CERS5	GSE92220	.	.	.	.
REG00014	MED17	GSE92220	.	.	.	.
REG00014	MED26	GSE92220	.	.	.	.
REG00014	EPHX3	GSE92220	.	.	.	.
REG00014	KBTBD4	GSE92220	.	.	.	.
REG00014	POLDIP2	GSE92220	.	.	.	.
REG00014	POLDIP3	GSE92220	.	.	.	.
REG00014	ZNF507	GSE92220	.	.	.	.
REG00014	MTA3	GSE92220	.	.	.	.
REG00014	PIKFYVE	GSE92220	.	.	.	.
REG00014	PIP4K2C	GSE92220	.	.	.	.
REG00014	CINP	GSE92220	.	.	.	.
REG00014	CRTAP	GSE92220	.	.	.	.
REG00014	TRMT61A	GSE92220	.	.	.	.
REG00014	INF2	GSE92220	.	.	.	.
REG00014	PLD4	GSE92220	.	.	.	.
REG00014	PACS2	GSE92220	.	.	.	.
REG00014	QTRT1	GSE92220	.	.	.	.
REG00014	DND1	GSE92220	.	.	.	.
REG00014	ADCY7	GSE92220	.	.	.	.
REG00014	ADNP2	GSE92220	.	.	.	.
REG00014	ASXL2	GSE92220	.	.	.	.
REG00014	THUMPD1	GSE92220	.	.	.	.
REG00014	BEND4	GSE92220	.	.	.	.
REG00014	CERS6	GSE92220	.	.	.	.
REG00014	CRY1	GSE92220	.	.	.	.
REG00014	GRIP2	GSE92220	.	.	.	.
REG00014	ABTB2	GSE92220	.	.	.	.
REG00014	GMCL1	GSE92220	.	.	.	.
REG00014	BTBD11	GSE92220	.	.	.	.
REG00014	NACC2	GSE92220	.	.	.	.
REG00014	ZBTB4	GSE92220	.	.	.	.
REG00014	CTIF	GSE92220	.	.	.	.
REG00014	ITGB1BP1	GSE92220	.	.	.	.
REG00014	CRYBA1	GSE92220	.	.	.	.
REG00014	NDUFA12	GSE92220	.	.	.	.
REG00014	STAC2	GSE92220	.	.	.	.

REG00014	AASDH	GSE92220	.	.	.	.
REG00014	CRYBB3	GSE92220	.	.	.	.
REG00014	AZI2	GSE92220	.	.	.	.
REG00014	ATAD3B	GSE92220	.	.	.	.
REG00014	MIA3	GSE92220	.	.	.	.
REG00014	ABI2	GSE92220	.	.	.	.
REG00014	DNTTIP2	GSE92220	.	.	.	.
REG00014	COMMD6	GSE92220	.	.	.	.
REG00014	ACTL8	GSE92220	.	.	.	.
REG00014	KLHL17	GSE92220	.	.	.	.
REG00014	INHBE	GSE92220	.	.	.	.
REG00014	ADIPOR1	GSE92220	.	.	.	.
REG00014	ADIPOR2	GSE92220	.	.	.	.
REG00014	WWTR1	GSE92220	.	.	.	.
REG00014	ATL2	GSE92220	.	.	.	.
REG00014	ARL6IP6	GSE92220	.	.	.	.
REG00014	AEBP2	GSE92220	.	.	.	.
REG00014	TOR1AIP2	GSE92220	.	.	.	.
REG00014	GALM	GSE92220	.	.	.	.
REG00014	ARMCX3	GSE92220	.	.	.	.
REG00014	AMIGO3	GSE92220	.	.	.	.
REG00014	ANAPC10	GSE92220	.	.	.	.
REG00014	ANGPTL7	GSE92220	.	.	.	.
REG00014	APH1B	GSE92220	.	.	.	.
REG00014	MAMDC4	GSE92220	.	.	.	.
REG00014	SHROOM1	GSE92220	.	.	.	.
REG00014	SRPRB	GSE92220	.	.	.	.
REG00014	A1CF	GSE92220	.	.	.	.
REG00014	ATRAID	GSE92220	.	.	.	.
REG00014	ZBTB8OS	GSE92220	.	.	.	.
REG00014	SORBS2	GSE92220	.	.	.	.
REG00014	ARSG	GSE92220	.	.	.	.
REG00014	ASCC2	GSE92220	.	.	.	.
REG00014	AKNA	GSE92220	.	.	.	.
REG00014	TXNDC9	GSE92220	.	.	.	.
REG00014	ATP13A3	GSE92220	.	.	.	.
REG00014	AURKAIP1	GSE92220	.	.	.	.
REG00014	RNASEH2C	GSE92220	.	.	.	.
REG00014	TM2D2	GSE92220	.	.	.	.
REG00014	TM2D3	GSE92220	.	.	.	.
REG00014	FAM129C	GSE92220	.	.	.	.
REG00014	STAP1	GSE92220	.	.	.	.
REG00014	ZCCHC14	GSE92220	.	.	.	.
REG00014	B3GNT8	GSE92220	.	.	.	.
REG00014	B3GNT6	GSE92220	.	.	.	.
REG00014	WWC2	GSE92220	.	.	.	.
REG00014	HES4	GSE92220	.	.	.	.
REG00014	CEND1	GSE92220	.	.	.	.
REG00014	PHF21A	GSE92220	.	.	.	.
REG00014	HGH1	GSE92220	.	.	.	.

REG00014	TPPP	GSE92220	.	.	.	.
REG00014	HACD3	GSE92220	.	.	.	.
REG00014	CABIN1	GSE92220	.	.	.	.
REG00014	SDF4	GSE92220	.	.	.	.
REG00014	CRYZ	GSE92220	.	.	.	.
REG00014	CAMK2N1	GSE92220	.	.	.	.
REG00014	CAMK2N2	GSE92220	.	.	.	.
REG00014	OVCA2	GSE92220	.	.	.	.
REG00014	BLOC1S4	GSE92220	.	.	.	.
REG00014	CISD2	GSE92220	.	.	.	.
REG00014	CILP2	GSE92220	.	.	.	.
REG00014	ATP13A1	GSE92220	.	.	.	.
REG00014	CIR1	GSE92220	.	.	.	.
REG00014	CS	GSE92220	.	.	.	.
REG00014	CDK12	GSE92220	.	.	.	.
REG00014	CCPG1	GSE92220	.	.	.	.
REG00014	CCAR1	GSE92220	.	.	.	.
REG00014	CEP350	GSE92220	.	.	.	.
REG00014	GAL3ST1	GSE92220	.	.	.	.
REG00014	LETMD1	GSE92220	.	.	.	.
REG00014	SLC9B1	GSE92220	.	.	.	.
REG00014	DRC1	GSE92220	.	.	.	.
REG00014	YARS2	GSE92220	.	.	.	.
REG00014	NMD3	GSE92220	.	.	.	.
REG00014	TMED5	GSE92220	.	.	.	.
REG00014	FAHD2A	GSE92220	.	.	.	.
REG00014	FAM96B	GSE92220	.	.	.	.
REG00014	AMDHD2	GSE92220	.	.	.	.
REG00014	DESI2	GSE92220	.	.	.	.
REG00014	PTRH2	GSE92220	.	.	.	.
REG00014	ASCC1	GSE92220	.	.	.	.
REG00014	DERA	GSE92220	.	.	.	.
REG00014	DPH5	GSE92220	.	.	.	.
REG00014	CUTC	GSE92220	.	.	.	.
REG00014	SIDT2	GSE92220	.	.	.	.
REG00014	RRNAD1	GSE92220	.	.	.	.
REG00014	UTP18	GSE92220	.	.	.	.
REG00014	SCCPDH	GSE92220	.	.	.	.
REG00014	SAMM50	GSE92220	.	.	.	.
REG00014	ABHD17B	GSE92220	.	.	.	.
REG00014	SLC25A39	GSE92220	.	.	.	.
REG00014	YBX3	GSE92220	.	.	.	.
REG00014	OTUD6B	GSE92220	.	.	.	.
REG00014	KMT5B	GSE92220	.	.	.	.
REG00014	TRAPPC12	GSE92220	.	.	.	.
REG00014	RMDN1	GSE92220	.	.	.	.
REG00014	RRP7A	GSE92220	.	.	.	.
REG00014	CHDH	GSE92220	.	.	.	.
REG00014	CEPT1	GSE92220	.	.	.	.
REG00014	CHPF	GSE92220	.	.	.	.

REG00014	CSGALNACT2	GSE92220	.	.	.	.
REG00014	ADD1	GSE92220	.	.	.	.
REG00014	GPR137	GSE92220	.	.	.	.
REG00014	EMC7	GSE92220	.	.	.	.
REG00014	RBM15B	GSE92220	.	.	.	.
REG00014	MTMR11	GSE92220	.	.	.	.
REG00014	CLPTM1L	GSE92220	.	.	.	.
REG00014	LUC7L3	GSE92220	.	.	.	.
REG00014	CSE1L	GSE92220	.	.	.	.
REG00014	PLEKHO1	GSE92220	.	.	.	.
REG00014	CRTAM	GSE92220	.	.	.	.
REG00014	TACO1	GSE92220	.	.	.	.
REG00014	HIGD1B	GSE92220	.	.	.	.
REG00014	CMIP	GSE92220	.	.	.	.
REG00014	CSF1	GSE92220	.	.	.	.
REG00014	DENND4A	GSE92220	.	.	.	.
REG00014	CARTPT	GSE92220	.	.	.	.
REG00014	CDKN2AIP	GSE92220	.	.	.	.
REG00014	C18orf8	GSE92220	.	.	.	.
REG00014	NIP7	GSE92220	.	.	.	.
REG00014	CSF1R	GSE92220	.	.	.	.
REG00014	CPLX4	GSE92220	.	.	.	.
REG00014	MALRD1	GSE92220	.	.	.	.
REG00014	COPS8	GSE92220	.	.	.	.
REG00014	CPNE9	GSE92220	.	.	.	.
REG00014	C1GALT1	GSE92220	.	.	.	.
REG00014	CXXC1	GSE92220	.	.	.	.
REG00014	PHRF1	GSE92220	.	.	.	.
REG00014	TNS4	GSE92220	.	.	.	.
REG00014	CHTF8	GSE92220	.	.	.	.
REG00014	TSPYL2	GSE92220	.	.	.	.
REG00014	WASHC1	GSE92220	.	.	.	.
REG00014	CYB5B	GSE92220	.	.	.	.
REG00014	CSF3	GSE92220	.	.	.	.
REG00014	BOLA3	GSE92220	.	.	.	.
REG00014	CNDP2	GSE92220	.	.	.	.
REG00014	GHDC	GSE92220	.	.	.	.
REG00014	CSK	GSE92220	.	.	.	.
REG00014	HMCES	GSE92220	.	.	.	.
REG00014	CMC2	GSE92220	.	.	.	.
REG00014	OSTC	GSE92220	.	.	.	.
REG00014	ENY2	GSE92220	.	.	.	.
REG00014	DEDD2	GSE92220	.	.	.	.
REG00014	DCP2	GSE92220	.	.	.	.
REG00014	DNER	GSE92220	.	.	.	.
REG00014	DTX3	GSE92220	.	.	.	.
REG00014	EIF3M	GSE92220	.	.	.	.
REG00014	CHST14	GSE92220	.	.	.	.
REG00014	ANKRD23	GSE92220	.	.	.	.
REG00014	PSRC1	GSE92220	.	.	.	.

REG00014	PCBD2	GSE92220	.	.	.	.
REG00014	DLGAP4	GSE92220	.	.	.	.
REG00014	TTLL3	GSE92220	.	.	.	.
REG00014	LSM14A	GSE92220	.	.	.	.
REG00014	NEPRO	GSE92220	.	.	.	.
REG00014	C15orf39	GSE92220	.	.	.	.
REG00014	KIAA1429	GSE92220	.	.	.	.
REG00014	PLEKHG4	GSE92220	.	.	.	.
REG00014	CSNK1A1	GSE92220	.	.	.	.
REG00014	CHTOP	GSE92220	.	.	.	.
REG00014	TMEM268	GSE92220	.	.	.	.
REG00014	NOC2L	GSE92220	.	.	.	.
REG00014	TCTN3	GSE92220	.	.	.	.
REG00014	CSNK1D	GSE92220	.	.	.	.
REG00014	FAM98A	GSE92220	.	.	.	.
REG00014	HERC4	GSE92220	.	.	.	.
REG00014	ZZZ3	GSE92220	.	.	.	.
REG00014	PRR13	GSE92220	.	.	.	.
REG00014	TMEM98	GSE92220	.	.	.	.
REG00014	CSNK1E	GSE92220	.	.	.	.
REG00014	LOC400927-CSNK1E	GSE92220	.	.	.	.
REG00014	TMEM186	GSE92220	.	.	.	.
REG00014	RSL1D1	GSE92220	.	.	.	.
REG00014	DCAF13	GSE92220	.	.	.	.
REG00014	NECAP1	GSE92220	.	.	.	.
REG00014	CSNK1G1	GSE92220	.	.	.	.
REG00014	ANAPC13	GSE92220	.	.	.	.
REG00014	EPC2	GSE92220	.	.	.	.
REG00014	NSL1	GSE92220	.	.	.	.
REG00014	MRFAP1	GSE92220	.	.	.	.
REG00014	CSNK1G2	GSE92220	.	.	.	.
REG00014	METTL7A	GSE92220	.	.	.	.
REG00014	TKFC	GSE92220	.	.	.	.
REG00014	INTS1	GSE92220	.	.	.	.
REG00014	OLFML2B	GSE92220	.	.	.	.
REG00014	CSNK1G3	GSE92220	.	.	.	.
REG00014	CCDC9	GSE92220	.	.	.	.
REG00014	FAM32A	GSE92220	.	.	.	.
REG00014	KANSL1	GSE92220	.	.	.	.
REG00014	PHF19	GSE92220	.	.	.	.
REG00014	CSNK2A1	GSE92220	.	.	.	.
REG00014	SPATS2L	GSE92220	.	.	.	.
REG00014	GINS2	GSE92220	.	.	.	.
REG00014	DESI1	GSE92220	.	.	.	.
REG00014	DDI2	GSE92220	.	.	.	.
REG00014	DNAJC14	GSE92220	.	.	.	.
REG00014	DOK3	GSE92220	.	.	.	.
REG00014	METTL9	GSE92220	.	.	.	.
REG00014	FILIP1L	GSE92220	.	.	.	.
REG00014	CSNK2A2	GSE92220	.	.	.	.

REG00014	DPY30	GSE92220	.	.	.	.
REG00014	DCTN5	GSE92220	.	.	.	.
REG00014	DYNLL2	GSE92220	.	.	.	.
REG00014	ANKS1B	GSE92220	.	.	.	.
REG00014	EEFSEC	GSE92220	.	.	.	.
REG00014	REXO1	GSE92220	.	.	.	.
REG00014	AHCTF1	GSE92220	.	.	.	.
REG00014	ENGASE	GSE92220	.	.	.	.
REG00014	DCBLD2	GSE92220	.	.	.	.
REG00014	MED28	GSE92220	.	.	.	.
REG00014	EPS15L1	GSE92220	.	.	.	.
REG00014	PRSS48	GSE92220	.	.	.	.
REG00014	DNAJC10	GSE92220	.	.	.	.
REG00014	PRELID3A	GSE92220	.	.	.	.
REG00014	C2orf40	GSE92220	.	.	.	.
REG00014	SMG5	GSE92220	.	.	.	.
REG00014	ESCO1	GSE92220	.	.	.	.
REG00014	ETAA1	GSE92220	.	.	.	.
REG00014	ETNK1	GSE92220	.	.	.	.
REG00014	EHMT1	GSE92220	.	.	.	.
REG00014	EIF3K	GSE92220	.	.	.	.
REG00014	MAPK15	GSE92220	.	.	.	.
REG00014	CSPG5	GSE92220	.	.	.	.
REG00014	FNDC3B	GSE92220	.	.	.	.
REG00014	FLAD1	GSE92220	.	.	.	.
REG00014	FBF1	GSE92220	.	.	.	.
REG00014	FASTK	GSE92220	.	.	.	.
REG00014	TMEM150A	GSE92220	.	.	.	.
REG00014	FBXL20	GSE92220	.	.	.	.
REG00014	FLVCR1	GSE92220	.	.	.	.
REG00014	AGGF1	GSE92220	.	.	.	.
REG00014	FBLIM1	GSE92220	.	.	.	.
REG00014	CSRP1	GSE92220	.	.	.	.
REG00014	MICAL3	GSE92220	.	.	.	.
REG00014	FAM219B	GSE92220	.	.	.	.
REG00014	TTC14	GSE92220	.	.	.	.
REG00014	CSRP2	GSE92220	.	.	.	.
REG00014	RABL6	GSE92220	.	.	.	.
REG00014	LARP1B	GSE92220	.	.	.	.
REG00014	QRICH1	GSE92220	.	.	.	.
REG00014	ANKHD1	GSE92220	.	.	.	.
REG00014	TBC1D8B	GSE92220	.	.	.	.
REG00014	PTCD3	GSE92220	.	.	.	.
REG00014	ACSS3	GSE92220	.	.	.	.
REG00014	METTL4	GSE92220	.	.	.	.
REG00014	NHLRC2	GSE92220	.	.	.	.
REG00014	FUOM	GSE92220	.	.	.	.
REG00014	NKPD1	GSE92220	.	.	.	.
REG00014	LINC00094	GSE92220	.	.	.	.
REG00014	CST3	GSE92220	.	.	.	.

REG00014	RBM44	GSE92220	.	.	.	.
REG00014	TYW3	GSE92220	.	.	.	.
REG00014	C19orf54	GSE92220	.	.	.	.
REG00014	RSBN1L	GSE92220	.	.	.	.
REG00014	GOLT1A	GSE92220	.	.	.	.
REG00014	SIMC1	GSE92220	.	.	.	.
REG00014	YJEFN3	GSE92220	.	.	.	.
REG00014	ZNF621	GSE92220	.	.	.	.
REG00014	CST7	GSE92220	.	.	.	.
REG00014	RINL	GSE92220	.	.	.	.
REG00014	FAM83H	GSE92220	.	.	.	.
REG00014	PLIN2	GSE92220	.	.	.	.
REG00014	SSUH2	GSE92220	.	.	.	.
REG00014	PDIA5	GSE92220	.	.	.	.
REG00014	RABEP2	GSE92220	.	.	.	.
REG00014	FOXJ2	GSE92220	.	.	.	.
REG00014	CSTB	GSE92220	.	.	.	.
REG00014	RFFL	GSE92220	.	.	.	.
REG00014	FN3K	GSE92220	.	.	.	.
REG00014	FZR1	GSE92220	.	.	.	.
REG00014	MLST8	GSE92220	.	.	.	.
REG00014	CSTF1	GSE92220	.	.	.	.
REG00014	GPRIN1	GSE92220	.	.	.	.
REG00014	C3orf18	GSE92220	.	.	.	.
REG00014	GALP	GSE92220	.	.	.	.
REG00014	GAS2L2	GSE92220	.	.	.	.
REG00014	FBXO44	GSE92220	.	.	.	.
REG00014	CSTF3	GSE92220	.	.	.	.
REG00014	SIGLEC16	GSE92220	.	.	.	.
REG00014	GMIP	GSE92220	.	.	.	.
REG00014	CCDC91	GSE92220	.	.	.	.
REG00014	YAE1D1	GSE92220	.	.	.	.
REG00014	MFF	GSE92220	.	.	.	.
REG00014	G6PC3	GSE92220	.	.	.	.
REG00014	MOGS	GSE92220	.	.	.	.
REG00014	GPAM	GSE92220	.	.	.	.
REG00014	CEP104	GSE92220	.	.	.	.
REG00014	IRGQ	GSE92220	.	.	.	.
REG00014	GAL3ST2	GSE92220	.	.	.	.
REG00014	GLT8D1	GSE92220	.	.	.	.
REG00014	GOLGA7	GSE92220	.	.	.	.
REG00014	YIPF5	GSE92220	.	.	.	.
REG00014	TMED9	GSE92220	.	.	.	.
REG00014	GOLPH3L	GSE92220	.	.	.	.
REG00014	OXER1	GSE92220	.	.	.	.
REG00014	GREB1	GSE92220	.	.	.	.
REG00014	DCAF8	GSE92220	.	.	.	.
REG00014	CASC4	GSE92220	.	.	.	.
REG00014	PRRC2C	GSE92220	.	.	.	.
REG00014	MOB2	GSE92220	.	.	.	.

REG00014	CREBZF	GSE92220	.	.	.	.
REG00014	ASNSD1	GSE92220	.	.	.	.
REG00014	HSH2D	GSE92220	.	.	.	.
REG00014	EIF2AK1	GSE92220	.	.	.	.
REG00014	CCDC85B	GSE92220	.	.	.	.
REG00014	CHP2	GSE92220	.	.	.	.
REG00014	VPS37A	GSE92220	.	.	.	.
REG00014	PSMG2	GSE92220	.	.	.	.
REG00014	ANGPTL8	GSE92220	.	.	.	.
REG00014	CTBP1	GSE92220	.	.	.	.
REG00014	PIK3IP1	GSE92220	.	.	.	.
REG00014	DDIT4	GSE92220	.	.	.	.
REG00014	DOT1L	GSE92220	.	.	.	.
REG00014	OTUD4	GSE92220	.	.	.	.
REG00014	HEXIM1	GSE92220	.	.	.	.
REG00014	OLFML3	GSE92220	.	.	.	.
REG00014	CTBS	GSE92220	.	.	.	.
REG00014	EME1	GSE92220	.	.	.	.
REG00014	MAF1	GSE92220	.	.	.	.
REG00014	NPRL2	GSE92220	.	.	.	.
REG00014	IFFO1	GSE92220	.	.	.	.
REG00014	HP1BP3	GSE92220	.	.	.	.
REG00014	CTDP1	GSE92220	.	.	.	.
REG00014	DNAJB7	GSE92220	.	.	.	.
REG00014	NMRAL1	GSE92220	.	.	.	.
REG00014	CTF1	GSE92220	.	.	.	.
REG00014	COA3	GSE92220	.	.	.	.
REG00014	C19orf53	GSE92220	.	.	.	.
REG00014	ZNF706	GSE92220	.	.	.	.
REG00014	COMMD2	GSE92220	.	.	.	.
REG00014	INIP	GSE92220	.	.	.	.
REG00014	ARMC8	GSE92220	.	.	.	.
REG00014	CTGF	GSE92220	.	.	.	.
REG00014	ZBTB44	GSE92220	.	.	.	.
REG00014	CTH	GSE92220	.	.	.	.
REG00014	LGALSL	GSE92220	.	.	.	.
REG00014	CNIH4	GSE92220	.	.	.	.
REG00014	TMEM208	GSE92220	.	.	.	.
REG00014	SSU72	GSE92220	.	.	.	.
REG00014	ZNF581	GSE92220	.	.	.	.
REG00014	TMEM216	GSE92220	.	.	.	.
REG00014	MON1B	GSE92220	.	.	.	.
REG00014	PITHD1	GSE92220	.	.	.	.
REG00014	PLPP5	GSE92220	.	.	.	.
REG00014	PRPF40B	GSE92220	.	.	.	.
REG00014	N4BP2L1	GSE92220	.	.	.	.
REG00014	SMDT1	GSE92220	.	.	.	.
REG00014	TPGS1	GSE92220	.	.	.	.
REG00014	DMKN	GSE92220	.	.	.	.
REG00014	GATC	GSE92220	.	.	.	.

REG00014	FBXO46	GSE92220	.	.	.	.
REG00014	ACD	GSE92220	.	.	.	.
REG00014	WDR45B	GSE92220	.	.	.	.
REG00014	RBMXL1	GSE92220	.	.	.	.
REG00014	LYRM1	GSE92220	.	.	.	.
REG00014	NBPF3	GSE92220	.	.	.	.
REG00014	MOSPD3	GSE92220	.	.	.	.
REG00014	CCDC34	GSE92220	.	.	.	.
REG00014	SMAP2	GSE92220	.	.	.	.
REG00014	CTNNA1	GSE92220	.	.	.	.
REG00014	CMBL	GSE92220	.	.	.	.
REG00014	ZC3H18	GSE92220	.	.	.	.
REG00014	MTSS1L	GSE92220	.	.	.	.
REG00014	DCAF15	GSE92220	.	.	.	.
REG00014	LMF2	GSE92220	.	.	.	.
REG00014	CABLES1	GSE92220	.	.	.	.
REG00014	TP53I13	GSE92220	.	.	.	.
REG00014	AP5B1	GSE92220	.	.	.	.
REG00014	BOD1	GSE92220	.	.	.	.
REG00014	C11orf84	GSE92220	.	.	.	.
REG00014	SHF	GSE92220	.	.	.	.
REG00014	OTULIN	GSE92220	.	.	.	.
REG00014	CTNNAL1	GSE92220	.	.	.	.
REG00014	TMEM44	GSE92220	.	.	.	.
REG00014	NCBP2-AS2	GSE92220	.	.	.	.
REG00014	PKDCC	GSE92220	.	.	.	.
REG00014	HNRNPLL	GSE92220	.	.	.	.
REG00014	MARS2	GSE92220	.	.	.	.
REG00014	LRSAM1	GSE92220	.	.	.	.
REG00014	TMEM129	GSE92220	.	.	.	.
REG00014	MARCHF9	GSE92220	.	.	.	.
REG00014	CTNNB1	GSE92220	.	.	.	.
REG00014	SFT2D2	GSE92220	.	.	.	.
REG00014	SLC9B2	GSE92220	.	.	.	.
REG00014	CTNND1	GSE92220	.	.	.	.
REG00014	PWWP2B	GSE92220	.	.	.	.
REG00014	ADAT3	GSE92220	.	.	.	.
REG00014	MVB12A	GSE92220	.	.	.	.
REG00014	SMIM12	GSE92220	.	.	.	.
REG00014	MFSD3	GSE92220	.	.	.	.
REG00014	KTI12	GSE92220	.	.	.	.
REG00014	CHADL	GSE92220	.	.	.	.
REG00014	SMIM19	GSE92220	.	.	.	.
REG00014	TM4SF19	GSE92220	.	.	.	.
REG00014	CCDC124	GSE92220	.	.	.	.
REG00014	ZNF689	GSE92220	.	.	.	.
REG00014	TMEM240	GSE92220	.	.	.	.
REG00014	CTPS1	GSE92220	.	.	.	.
REG00014	ARL8A	GSE92220	.	.	.	.
REG00014	TMEM45B	GSE92220	.	.	.	.

REG00014	FAM199X	GSE92220	.	.	.	.
REG00014	ZNF653	GSE92220	.	.	.	.
REG00014	CCDC74A	GSE92220	.	.	.	.
REG00014	SLC25A46	GSE92220	.	.	.	.
REG00014	DPH7	GSE92220	.	.	.	.
REG00014	ADH4	GSE92220	.	.	.	.
REG00014	CTPS2	GSE92220	.	.	.	.
REG00014	TMEM219	GSE92220	.	.	.	.
REG00014	ZFAND2B	GSE92220	.	.	.	.
REG00014	PIH1D2	GSE92220	.	.	.	.
REG00014	FAM49B	GSE92220	.	.	.	.
REG00014	CNOT11	GSE92220	.	.	.	.
REG00014	MMADHC	GSE92220	.	.	.	.
REG00014	ARRDC2	GSE92220	.	.	.	.
REG00014	SGSM3	GSE92220	.	.	.	.
REG00014	LYRM2	GSE92220	.	.	.	.
REG00014	CTRC	GSE92220	.	.	.	.
REG00014	AHDC1	GSE92220	.	.	.	.
REG00014	YIPF1	GSE92220	.	.	.	.
REG00014	RSRP1	GSE92220	.	.	.	.
REG00014	MOSPD1	GSE92220	.	.	.	.
REG00014	TMX4	GSE92220	.	.	.	.
REG00014	CTRL	GSE92220	.	.	.	.
REG00014	ANO6	GSE92220	.	.	.	.
REG00014	ENKD1	GSE92220	.	.	.	.
REG00014	CRISPLD2	GSE92220	.	.	.	.
REG00014	IQCG	GSE92220	.	.	.	.
REG00014	GRAMD1C	GSE92220	.	.	.	.
REG00014	TMEM18	GSE92220	.	.	.	.
REG00014	C1orf167	GSE92220	.	.	.	.
REG00014	ZCCHC8	GSE92220	.	.	.	.
REG00014	CCDC74B	GSE92220	.	.	.	.
REG00014	APOLD1	GSE92220	.	.	.	.
REG00014	CTSB	GSE92220	.	.	.	.
REG00014	KLHDC4	GSE92220	.	.	.	.
REG00014	PUS7L	GSE92220	.	.	.	.
REG00014	FAM71E2	GSE92220	.	.	.	.
REG00014	CTSC	GSE92220	.	.	.	.
REG00014	ZNF543	GSE92220	.	.	.	.
REG00014	FAM129B	GSE92220	.	.	.	.
REG00014	FNDC8	GSE92220	.	.	.	.
REG00014	DRC7	GSE92220	.	.	.	.
REG00014	CTSD	GSE92220	.	.	.	.
REG00014	ZNF700	GSE92220	.	.	.	.
REG00014	ADH5	GSE92220	.	.	.	.
REG00014	FBXL19	GSE92220	.	.	.	.
REG00014	COQ9	GSE92220	.	.	.	.
REG00014	CCDC70	GSE92220	.	.	.	.
REG00014	FAM198B	GSE92220	.	.	.	.
REG00014	C3orf20	GSE92220	.	.	.	.

REG00014	QRICH2	GSE92220	.	.	.	.
REG00014	C1orf131	GSE92220	.	.	.	.
REG00014	RNF214	GSE92220	.	.	.	.
REG00014	C2orf83	GSE92220	.	.	.	.
REG00014	CTSH	GSE92220	.	.	.	.
REG00014	ZNF710	GSE92220	.	.	.	.
REG00014	SLC30A10	GSE92220	.	.	.	.
REG00014	SPRTN	GSE92220	.	.	.	.
REG00014	RNF170	GSE92220	.	.	.	.
REG00014	CTSK	GSE92220	.	.	.	.
REG00014	TMEM222	GSE92220	.	.	.	.
REG00014	HDHD2	GSE92220	.	.	.	.
REG00014	TEX35	GSE92220	.	.	.	.
REG00014	CCDC8	GSE92220	.	.	.	.
REG00014	CTSL	GSE92220	.	.	.	.
REG00014	INTS14	GSE92220	.	.	.	.
REG00014	ANKRD13C	GSE92220	.	.	.	.
REG00014	PDE12	GSE92220	.	.	.	.
REG00014	LIN54	GSE92220	.	.	.	.
REG00014	FAM200A	GSE92220	.	.	.	.
REG00014	OTUD5	GSE92220	.	.	.	.
REG00014	FLYWCH1	GSE92220	.	.	.	.
REG00014	ZNF664	GSE92220	.	.	.	.
REG00014	FYTTD1	GSE92220	.	.	.	.
REG00014	SUSD1	GSE92220	.	.	.	.
REG00014	ATXN7L3	GSE92220	.	.	.	.
REG00014	RNF208	GSE92220	.	.	.	.
REG00014	KIAA1217	GSE92220	.	.	.	.
REG00014	LRP2BP	GSE92220	.	.	.	.
REG00014	PRAG1	GSE92220	.	.	.	.
REG00014	TMEM64	GSE92220	.	.	.	.
REG00014	C19orf12	GSE92220	.	.	.	.
REG00014	TSR2	GSE92220	.	.	.	.
REG00014	MTURN	GSE92220	.	.	.	.
REG00014	MFSD11	GSE92220	.	.	.	.
REG00014	PAF1	GSE92220	.	.	.	.
REG00014	KAZALD1	GSE92220	.	.	.	.
REG00014	FRMD8	GSE92220	.	.	.	.
REG00014	ATG16L2	GSE92220	.	.	.	.
REG00014	IWS1	GSE92220	.	.	.	.
REG00014	CTSZ	GSE92220	.	.	.	.
REG00014	WIPI1	GSE92220	.	.	.	.
REG00014	SPATA6L	GSE92220	.	.	.	.
REG00014	KANSL3	GSE92220	.	.	.	.
REG00014	TMEM248	GSE92220	.	.	.	.
REG00014	MREG	GSE92220	.	.	.	.
REG00014	TRMU	GSE92220	.	.	.	.
REG00014	ARGLU1	GSE92220	.	.	.	.
REG00014	TMEM51	GSE92220	.	.	.	.
REG00014	YEATS2	GSE92220	.	.	.	.

REG00014	CELF1	GSE92220	.	.	.	.
REG00014	SAMD4B	GSE92220	.	.	.	.
REG00014	EBLN2	GSE92220	.	.	.	.
REG00014	ATP5SL	GSE92220	.	.	.	.
REG00014	ADH6	GSE92220	.	.	.	.
REG00014	BSDC1	GSE92220	.	.	.	.
REG00014	RBM22	GSE92220	.	.	.	.
REG00014	MANSC1	GSE92220	.	.	.	.
REG00014	VAC14	GSE92220	.	.	.	.
REG00014	XKR8	GSE92220	.	.	.	.
REG00014	CUL1	GSE92220	.	.	.	.
REG00014	TMEM39B	GSE92220	.	.	.	.
REG00014	PRPF38B	GSE92220	.	.	.	.
REG00014	GPN2	GSE92220	.	.	.	.
REG00014	CEP192	GSE92220	.	.	.	.
REG00014	HEATR1	GSE92220	.	.	.	.
REG00014	CUL2	GSE92220	.	.	.	.
REG00014	SRBD1	GSE92220	.	.	.	.
REG00014	WRAP53	GSE92220	.	.	.	.
REG00014	CCDC88A	GSE92220	.	.	.	.
REG00014	FAM193B	GSE92220	.	.	.	.
REG00014	NDC1	GSE92220	.	.	.	.
REG00014	ANKZF1	GSE92220	.	.	.	.
REG00014	NECAP2	GSE92220	.	.	.	.
REG00014	WDR74	GSE92220	.	.	.	.
REG00014	CUL3	GSE92220	.	.	.	.
REG00014	MTPAP	GSE92220	.	.	.	.
REG00014	DALRD3	GSE92220	.	.	.	.
REG00014	SDAD1	GSE92220	.	.	.	.
REG00014	RFWD3	GSE92220	.	.	.	.
REG00014	CUL4A	GSE92220	.	.	.	.
REG00014	ARHGEF10L	GSE92220	.	.	.	.
REG00014	TMEM33	GSE92220	.	.	.	.
REG00014	TSR1	GSE92220	.	.	.	.
REG00014	MSL2	GSE92220	.	.	.	.
REG00014	CEP72	GSE92220	.	.	.	.
REG00014	INTS10	GSE92220	.	.	.	.
REG00014	CUL4B	GSE92220	.	.	.	.
REG00014	RMDN3	GSE92220	.	.	.	.
REG00014	SMG8	GSE92220	.	.	.	.
REG00014	GNL3L	GSE92220	.	.	.	.
REG00014	SLC25A36	GSE92220	.	.	.	.
REG00014	DNAJC17	GSE92220	.	.	.	.
REG00014	PRMT7	GSE92220	.	.	.	.
REG00014	EVA1B	GSE92220	.	.	.	.
REG00014	RPRD1A	GSE92220	.	.	.	.
REG00014	PLEKHG6	GSE92220	.	.	.	.
REG00014	SETD5	GSE92220	.	.	.	.
REG00014	ATAD3A	GSE92220	.	.	.	.
REG00014	CUX1	GSE92220	.	.	.	.

REG00014	DNAJC11	GSE92220	.	.	.	.
REG00014	TMEM57	GSE92220	.	.	.	.
REG00014	NAXD	GSE92220	.	.	.	.
REG00014	RAVER2	GSE92220	.	.	.	.
REG00014	UBA6	GSE92220	.	.	.	.
REG00014	SLC38A7	GSE92220	.	.	.	.
REG00014	OGFOD1	GSE92220	.	.	.	.
REG00014	TMEM184C	GSE92220	.	.	.	.
REG00014	SLC47A1	GSE92220	.	.	.	.
REG00014	SEPTIN11	GSE92220	.	.	.	.
REG00014	OGDHL	GSE92220	.	.	.	.
REG00014	RCOR3	GSE92220	.	.	.	.
REG00014	L1TD1	GSE92220	.	.	.	.
REG00014	TTC17	GSE92220	.	.	.	.
REG00014	C1orf106	GSE92220	.	.	.	.
REG00014	TMEM39A	GSE92220	.	.	.	.
REG00014	C7orf43	GSE92220	.	.	.	.
REG00014	TMEM19	GSE92220	.	.	.	.
REG00014	VPS53	GSE92220	.	.	.	.
REG00014	FAM214A	GSE92220	.	.	.	.
REG00014	CCDC93	GSE92220	.	.	.	.
REG00014	UBE2W	GSE92220	.	.	.	.
REG00014	RBM41	GSE92220	.	.	.	.
REG00014	C4orf19	GSE92220	.	.	.	.
REG00014	PRR11	GSE92220	.	.	.	.
REG00014	DDX19A	GSE92220	.	.	.	.
REG00014	FAM105A	GSE92220	.	.	.	.
REG00014	CPPED1	GSE92220	.	.	.	.
REG00014	C5orf22	GSE92220	.	.	.	.
REG00014	DRAM1	GSE92220	.	.	.	.
REG00014	FAM63A	GSE92220	.	.	.	.
REG00014	C19orf66	GSE92220	.	.	.	.
REG00014	WDR33	GSE92220	.	.	.	.
REG00014	TCP11L1	GSE92220	.	.	.	.
REG00014	BCORL1	GSE92220	.	.	.	.
REG00014	SAP30L	GSE92220	.	.	.	.
REG00014	ZNF556	GSE92220	.	.	.	.
REG00014	CHMP6	GSE92220	.	.	.	.
REG00014	ZKSCAN2	GSE92220	.	.	.	.
REG00014	ATG101	GSE92220	.	.	.	.
REG00014	PAAF1	GSE92220	.	.	.	.
REG00014	FAM127A	GSE92220	.	.	.	.
REG00014	ANKRD53	GSE92220	.	.	.	.
REG00014	CARS2	GSE92220	.	.	.	.
REG00014	GSDMD	GSE92220	.	.	.	.
REG00014	ADK	GSE92220	.	.	.	.
REG00014	CYB5A	GSE92220	.	.	.	.
REG00014	CHD9	GSE92220	.	.	.	.
REG00014	KCTD17	GSE92220	.	.	.	.
REG00014	WDR59	GSE92220	.	.	.	.

REG00014	MIIP	GSE92220	.	.	.	.
REG00014	COA7	GSE92220	.	.	.	.
REG00014	NT5DC2	GSE92220	.	.	.	.
REG00014	LPCAT1	GSE92220	.	.	.	.
REG00014	C12orf43	GSE92220	.	.	.	.
REG00014	AEN	GSE92220	.	.	.	.
REG00014	WDR75	GSE92220	.	.	.	.
REG00014	LAS1L	GSE92220	.	.	.	.
REG00014	NKAIN1	GSE92220	.	.	.	.
REG00014	AKIRIN1	GSE92220	.	.	.	.
REG00014	ISG20L2	GSE92220	.	.	.	.
REG00014	TANGO6	GSE92220	.	.	.	.
REG00014	MAP6D1	GSE92220	.	.	.	.
REG00014	VPS37B	GSE92220	.	.	.	.
REG00014	TTC31	GSE92220	.	.	.	.
REG00014	MUL1	GSE92220	.	.	.	.
REG00014	SMG9	GSE92220	.	.	.	.
REG00014	CCDC14	GSE92220	.	.	.	.
REG00014	S100PBP	GSE92220	.	.	.	.
REG00014	KRI1	GSE92220	.	.	.	.
REG00014	CYBA	GSE92220	.	.	.	.
REG00014	WDR54	GSE92220	.	.	.	.
REG00014	RFX7	GSE92220	.	.	.	.
REG00014	MTHFSD	GSE92220	.	.	.	.
REG00014	ARMC5	GSE92220	.	.	.	.
REG00014	CENPT	GSE92220	.	.	.	.
REG00014	CYC1	GSE92220	.	.	.	.
REG00014	FASTKD5	GSE92220	.	.	.	.
REG00014	USB1	GSE92220	.	.	.	.
REG00014	PHACTR4	GSE92220	.	.	.	.
REG00014	GUF1	GSE92220	.	.	.	.
REG00014	C5orf42	GSE92220	.	.	.	.
REG00014	DNAJC22	GSE92220	.	.	.	.
REG00014	TMEM254	GSE92220	.	.	.	.
REG00014	CNTD2	GSE92220	.	.	.	.
REG00014	SLC7A6OS	GSE92220	.	.	.	.
REG00014	FAM161A	GSE92220	.	.	.	.
REG00014	CEP63	GSE92220	.	.	.	.
REG00014	EVA1A	GSE92220	.	.	.	.
REG00014	ZCCHC6	GSE92220	.	.	.	.
REG00014	BRD9	GSE92220	.	.	.	.
REG00014	COQ10B	GSE92220	.	.	.	.
REG00014	ZNF668	GSE92220	.	.	.	.
REG00014	C10orf88	GSE92220	.	.	.	.
REG00014	OGFOD2	GSE92220	.	.	.	.
REG00014	SYDE1	GSE92220	.	.	.	.
REG00014	TRAPPC13	GSE92220	.	.	.	.
REG00014	DHRS12	GSE92220	.	.	.	.
REG00014	FAM65A	GSE92220	.	.	.	.
REG00014	HHIPL2	GSE92220	.	.	.	.

REG00014	ZNF750	GSE92220	.	.	.	.
REG00014	NAA40	GSE92220	.	.	.	.
REG00014	PYCRL	GSE92220	.	.	.	.
REG00014	UBE2Z	GSE92220	.	.	.	.
REG00014	C16orf59	GSE92220	.	.	.	.
REG00014	MORN1	GSE92220	.	.	.	.
REG00014	METTL8	GSE92220	.	.	.	.
REG00014	CLIP2	GSE92220	.	.	.	.
REG00014	NOL10	GSE92220	.	.	.	.
REG00014	CXorf36	GSE92220	.	.	.	.
REG00014	ZNF696	GSE92220	.	.	.	.
REG00014	C1orf115	GSE92220	.	.	.	.
REG00014	MFSD1	GSE92220	.	.	.	.
REG00014	NAA60	GSE92220	.	.	.	.
REG00014	ZNF606	GSE92220	.	.	.	.
REG00014	C10orf95	GSE92220	.	.	.	.
REG00014	ZNF703	GSE92220	.	.	.	.
REG00014	KLHL22	GSE92220	.	.	.	.
REG00014	CCDC142	GSE92220	.	.	.	.
REG00014	CBR4	GSE92220	.	.	.	.
REG00014	ZC3H10	GSE92220	.	.	.	.
REG00014	ZNF514	GSE92220	.	.	.	.
REG00014	C1orf198	GSE92220	.	.	.	.
REG00014	AFAP1L2	GSE92220	.	.	.	.
REG00014	POMGNT2	GSE92220	.	.	.	.
REG00014	ATAD1	GSE92220	.	.	.	.
REG00014	NFATC2IP	GSE92220	.	.	.	.
REG00014	FAM136A	GSE92220	.	.	.	.
REG00014	TMEM87B	GSE92220	.	.	.	.
REG00014	STRIP1	GSE92220	.	.	.	.
REG00014	FIZ1	GSE92220	.	.	.	.
REG00014	C8orf76	GSE92220	.	.	.	.
REG00014	RITA1	GSE92220	.	.	.	.
REG00014	WDR73	GSE92220	.	.	.	.
REG00014	CYP17A1	GSE92220	.	.	.	.
REG00014	PRPF38A	GSE92220	.	.	.	.
REG00014	CGNL1	GSE92220	.	.	.	.
REG00014	MPND	GSE92220	.	.	.	.
REG00014	NSMCE4A	GSE92220	.	.	.	.
REG00014	C11orf71	GSE92220	.	.	.	.
REG00014	TET2	GSE92220	.	.	.	.
REG00014	KLHL24	GSE92220	.	.	.	.
REG00014	ZNF562	GSE92220	.	.	.	.
REG00014	AFTPH	GSE92220	.	.	.	.
REG00014	QPCTL	GSE92220	.	.	.	.
REG00014	GIN1	GSE92220	.	.	.	.
REG00014	FAM134B	GSE92220	.	.	.	.
REG00014	DEF8	GSE92220	.	.	.	.
REG00014	WDR55	GSE92220	.	.	.	.
REG00014	FAM83E	GSE92220	.	.	.	.

REG00014	ELP6	GSE92220	.	.	.	.
REG00014	MIEF1	GSE92220	.	.	.	.
REG00014	TRMT1	GSE92220	.	.	.	.
REG00014	TOR4A	GSE92220	.	.	.	.
REG00014	TMEM214	GSE92220	.	.	.	.
REG00014	PIGG	GSE92220	.	.	.	.
REG00014	TTC27	GSE92220	.	.	.	.
REG00014	DCAF16	GSE92220	.	.	.	.
REG00014	TEX10	GSE92220	.	.	.	.
REG00014	CWC25	GSE92220	.	.	.	.
REG00014	AMBRA1	GSE92220	.	.	.	.
REG00014	RETSAT	GSE92220	.	.	.	.
REG00014	ALKBH5	GSE92220	.	.	.	.
REG00014	INO80D	GSE92220	.	.	.	.
REG00014	CYP21A2	GSE92220	.	.	.	.
REG00014	PQLC2	GSE92220	.	.	.	.
REG00014	TTC19	GSE92220	.	.	.	.
REG00014	SH3TC1	GSE92220	.	.	.	.
REG00014	SPDL1	GSE92220	.	.	.	.
REG00014	LIME1	GSE92220	.	.	.	.
REG00014	IMPAD1	GSE92220	.	.	.	.
REG00014	CYP24A1	GSE92220	.	.	.	.
REG00014	TMEM161A	GSE92220	.	.	.	.
REG00014	LYAR	GSE92220	.	.	.	.
REG00014	EXD3	GSE92220	.	.	.	.
REG00014	KANSL2	GSE92220	.	.	.	.
REG00014	MARCHF5	GSE92220	.	.	.	.
REG00014	PIGV	GSE92220	.	.	.	.
REG00014	PUS7	GSE92220	.	.	.	.
REG00014	SDHAF2	GSE92220	.	.	.	.
REG00014	SLC48A1	GSE92220	.	.	.	.
REG00014	TMEM127	GSE92220	.	.	.	.
REG00014	PARP16	GSE92220	.	.	.	.
REG00014	TXNL4B	GSE92220	.	.	.	.
REG00014	TMEM160	GSE92220	.	.	.	.
REG00014	ODAM	GSE92220	.	.	.	.
REG00014	C1orf56	GSE92220	.	.	.	.
REG00014	INTS8	GSE92220	.	.	.	.
REG00014	ZNF692	GSE92220	.	.	.	.
REG00014	CYP27A1	GSE92220	.	.	.	.
REG00014	HPF1	GSE92220	.	.	.	.
REG00014	INTS11	GSE92220	.	.	.	.
REG00014	SLC35F6	GSE92220	.	.	.	.
REG00014	C2orf42	GSE92220	.	.	.	.
REG00014	LAGE3	GSE92220	.	.	.	.
REG00014	C1orf123	GSE92220	.	.	.	.
REG00014	ZNF770	GSE92220	.	.	.	.
REG00014	C1orf159	GSE92220	.	.	.	.
REG00014	TESC	GSE92220	.	.	.	.
REG00014	TUG1	GSE92220	.	.	.	.

REG00014	LAMTOR1	GSE92220	.	.	.	.
REG00014	C19orf24	GSE92220	.	.	.	.
REG00014	PIH1D1	GSE92220	.	.	.	.
REG00014	VSIG10	GSE92220	.	.	.	.
REG00014	DENND4C	GSE92220	.	.	.	.
REG00014	LINC00483	GSE92220	.	.	.	.
REG00014	TTC38	GSE92220	.	.	.	.
REG00014	PID1	GSE92220	.	.	.	.
REG00014	HEATR3	GSE92220	.	.	.	.
REG00014	NBPF1	GSE92220	.	.	.	.
REG00014	ZNHIT6	GSE92220	.	.	.	.
REG00014	C19orf60	GSE92220	.	.	.	.
REG00014	ACSF2	GSE92220	.	.	.	.
REG00014	PLEKHH3	GSE92220	.	.	.	.
REG00014	C22orf29	GSE92220	.	.	.	.
REG00014	EDC3	GSE92220	.	.	.	.
REG00014	SETD6	GSE92220	.	.	.	.
REG00014	HECTD3	GSE92220	.	.	.	.
REG00014	SIKE1	GSE92220	.	.	.	.
REG00014	SPATA20	GSE92220	.	.	.	.
REG00014	C12orf49	GSE92220	.	.	.	.
REG00014	ZNF552	GSE92220	.	.	.	.
REG00014	HMBOX1	GSE92220	.	.	.	.
REG00014	C19orf44	GSE92220	.	.	.	.
REG00014	PALB2	GSE92220	.	.	.	.
REG00014	AGBL5	GSE92220	.	.	.	.
REG00014	CRTC3	GSE92220	.	.	.	.
REG00014	SOWAHC	GSE92220	.	.	.	.
REG00014	INTS3	GSE92220	.	.	.	.
REG00014	QSER1	GSE92220	.	.	.	.
REG00014	CPED1	GSE92220	.	.	.	.
REG00014	CORO7	GSE92220	.	.	.	.
REG00014	NAA16	GSE92220	.	.	.	.
REG00014	ZNF574	GSE92220	.	.	.	.
REG00014	TMEM135	GSE92220	.	.	.	.
REG00014	FAAP100	GSE92220	.	.	.	.
REG00014	REEP4	GSE92220	.	.	.	.
REG00014	FHOD3	GSE92220	.	.	.	.
REG00014	ZNF672	GSE92220	.	.	.	.
REG00014	COLGALT1	GSE92220	.	.	.	.
REG00014	TUT1	GSE92220	.	.	.	.
REG00014	TMEM53	GSE92220	.	.	.	.
REG00014	PQLC1	GSE92220	.	.	.	.
REG00014	MTARC1	GSE92220	.	.	.	.
REG00014	MTMR14	GSE92220	.	.	.	.
REG00014	DENND2D	GSE92220	.	.	.	.
REG00014	CSPP1	GSE92220	.	.	.	.
REG00014	NUDT18	GSE92220	.	.	.	.
REG00014	MFSD13A	GSE92220	.	.	.	.
REG00014	KATNBL1	GSE92220	.	.	.	.

REG00014	MRM1	GSE92220	.	.	.	.
REG00014	BAIAP2L2	GSE92220	.	.	.	.
REG00014	TTC13	GSE92220	.	.	.	.
REG00014	PGGHG	GSE92220	.	.	.	.
REG00014	SPCS3	GSE92220	.	.	.	.
REG00014	FUZ	GSE92220	.	.	.	.
REG00014	PIF1	GSE92220	.	.	.	.
REG00014	OCEL1	GSE92220	.	.	.	.
REG00014	FAM124B	GSE92220	.	.	.	.
REG00014	TM4SF20	GSE92220	.	.	.	.
REG00014	FAM96A	GSE92220	.	.	.	.
REG00014	SH3D21	GSE92220	.	.	.	.
REG00014	DCAKD	GSE92220	.	.	.	.
REG00014	CYP2D7	GSE92220	.	.	.	.
REG00014	CYP2D6	GSE92220	.	.	.	.
REG00014	ZMYM1	GSE92220	.	.	.	.
REG00014	TTI2	GSE92220	.	.	.	.
REG00014	FOXRED2	GSE92220	.	.	.	.
REG00014	NOL9	GSE92220	.	.	.	.
REG00014	MSANTD2	GSE92220	.	.	.	.
REG00014	POMK	GSE92220	.	.	.	.
REG00014	PIEZO2	GSE92220	.	.	.	.
REG00014	ZNF768	GSE92220	.	.	.	.
REG00014	PODNL1	GSE92220	.	.	.	.
REG00014	ISOC2	GSE92220	.	.	.	.
REG00014	ZNF671	GSE92220	.	.	.	.
REG00014	CCDC82	GSE92220	.	.	.	.
REG00014	BBS10	GSE92220	.	.	.	.
REG00014	AGBL2	GSE92220	.	.	.	.
REG00014	ADORA2A	GSE92220	.	.	.	.
REG00014	LONRF1	GSE92220	.	.	.	.
REG00014	FAM91A1	GSE92220	.	.	.	.
REG00014	HEXDC	GSE92220	.	.	.	.
REG00014	CDHR3	GSE92220	.	.	.	.
REG00014	COMTD1	GSE92220	.	.	.	.
REG00014	SLFNL1	GSE92220	.	.	.	.
REG00014	SGK494	GSE92220	.	.	.	.
REG00014	B4GALNT4	GSE92220	.	.	.	.
REG00014	MYRFL	GSE92220	.	.	.	.
REG00014	ASPRV1	GSE92220	.	.	.	.
REG00014	NKX6-3	GSE92220	.	.	.	.
REG00014	ZNF385B	GSE92220	.	.	.	.
REG00014	FAM81B	GSE92220	.	.	.	.
REG00014	CYP2J2	GSE92220	.	.	.	.
REG00014	BTBD16	GSE92220	.	.	.	.
REG00014	ANKAR	GSE92220	.	.	.	.
REG00014	TTC23L	GSE92220	.	.	.	.
REG00014	CCDC13	GSE92220	.	.	.	.
REG00014	POU5F2	GSE92220	.	.	.	.
REG00014	FMR1NB	GSE92220	.	.	.	.

REG00014	CYP3A5	GSE92220	.	.	.	.
REG00014	PAQR4	GSE92220	.	.	.	.
REG00014	TTC9B	GSE92220	.	.	.	.
REG00014	AIFM3	GSE92220	.	.	.	.
REG00014	MARVELD2	GSE92220	.	.	.	.
REG00014	NADK2	GSE92220	.	.	.	.
REG00014	TTC30B	GSE92220	.	.	.	.
REG00014	FAAP20	GSE92220	.	.	.	.
REG00014	IKBIP	GSE92220	.	.	.	.
REG00014	C3orf33	GSE92220	.	.	.	.
REG00014	TSNARE1	GSE92220	.	.	.	.
REG00014	NUDT16	GSE92220	.	.	.	.
REG00014	KCTD18	GSE92220	.	.	.	.
REG00014	CYP4F2	GSE92220	.	.	.	.
REG00014	MANEAL	GSE92220	.	.	.	.
REG00014	CYP4F3	GSE92220	.	.	.	.
REG00014	SPATA33	GSE92220	.	.	.	.
REG00014	YTHDF3	GSE92220	.	.	.	.
REG00014	CCDC43	GSE92220	.	.	.	.
REG00014	VWCE	GSE92220	.	.	.	.
REG00014	KNCN	GSE92220	.	.	.	.
REG00014	CYP51A1	GSE92220	.	.	.	.
REG00014	ZNF513	GSE92220	.	.	.	.
REG00014	PUS10	GSE92220	.	.	.	.
REG00014	PPM1M	GSE92220	.	.	.	.
REG00014	CWF19L2	GSE92220	.	.	.	.
REG00014	FAM109A	GSE92220	.	.	.	.
REG00014	TMEM68	GSE92220	.	.	.	.
REG00014	HSPB6	GSE92220	.	.	.	.
REG00014	NSMCE2	GSE92220	.	.	.	.
REG00014	COQ10A	GSE92220	.	.	.	.
REG00014	CYB5D1	GSE92220	.	.	.	.
REG00014	C12orf66	GSE92220	.	.	.	.
REG00014	HGSNAT	GSE92220	.	.	.	.
REG00014	CYP8B1	GSE92220	.	.	.	.
REG00014	CFAP53	GSE92220	.	.	.	.
REG00014	CCDC138	GSE92220	.	.	.	.
REG00014	DNHD1	GSE92220	.	.	.	.
REG00014	FAM71A	GSE92220	.	.	.	.
REG00014	HEATR9	GSE92220	.	.	.	.
REG00014	ZFYVE27	GSE92220	.	.	.	.
REG00014	AXDND1	GSE92220	.	.	.	.
REG00014	LYSMD4	GSE92220	.	.	.	.
REG00014	CCDC17	GSE92220	.	.	.	.
REG00014	PRRT3	GSE92220	.	.	.	.
REG00014	KLHL35	GSE92220	.	.	.	.
REG00014	CCDC117	GSE92220	.	.	.	.
REG00014	WDR81	GSE92220	.	.	.	.
REG00014	HECTD4	GSE92220	.	.	.	.
REG00014	PDDC1	GSE92220	.	.	.	.

REG00014	KDF1	GSE92220	.	.	.	.
REG00014	ZNF554	GSE92220	.	.	.	.
REG00014	XXYLT1	GSE92220	.	.	.	.
REG00014	DAD1	GSE92220	.	.	.	.
REG00014	ENDOV	GSE92220	.	.	.	.
REG00014	SDE2	GSE92220	.	.	.	.
REG00014	CD55	GSE92220	.	.	.	.
REG00014	TRMT44	GSE92220	.	.	.	.
REG00014	RNF168	GSE92220	.	.	.	.
REG00014	EML3	GSE92220	.	.	.	.
REG00014	TERB1	GSE92220	.	.	.	.
REG00014	L3MBTL4	GSE92220	.	.	.	.
REG00014	MIER3	GSE92220	.	.	.	.
REG00014	MYOM3	GSE92220	.	.	.	.
REG00014	WDSUB1	GSE92220	.	.	.	.
REG00014	SYNE4	GSE92220	.	.	.	.
REG00014	SCAI	GSE92220	.	.	.	.
REG00014	C19orf25	GSE92220	.	.	.	.
REG00014	PROX2	GSE92220	.	.	.	.
REG00014	LRRC57	GSE92220	.	.	.	.
REG00014	DAP	GSE92220	.	.	.	.
REG00014	PATL1	GSE92220	.	.	.	.
REG00014	C19orf47	GSE92220	.	.	.	.
REG00014	ANKS6	GSE92220	.	.	.	.
REG00014	ZNF565	GSE92220	.	.	.	.
REG00014	ERFE	GSE92220	.	.	.	.
REG00014	DAP3	GSE92220	.	.	.	.
REG00014	DAPK1	GSE92220	.	.	.	.
REG00014	FBLN7	GSE92220	.	.	.	.
REG00014	C5orf24	GSE92220	.	.	.	.
REG00014	TYW5	GSE92220	.	.	.	.
REG00014	LCLAT1	GSE92220	.	.	.	.
REG00014	CNEP1R1	GSE92220	.	.	.	.
REG00014	DAPK3	GSE92220	.	.	.	.
REG00014	GPATCH11	GSE92220	.	.	.	.
REG00014	FAM98B	GSE92220	.	.	.	.
REG00014	TMEM192	GSE92220	.	.	.	.
REG00014	PAPD4	GSE92220	.	.	.	.
REG00014	DARS	GSE92220	.	.	.	.
REG00014	LSMEM2	GSE92220	.	.	.	.
REG00014	ZFP41	GSE92220	.	.	.	.
REG00014	C2orf69	GSE92220	.	.	.	.
REG00014	DIDO1	GSE92220	.	.	.	.
REG00014	COX18	GSE92220	.	.	.	.
REG00014	CNIH3	GSE92220	.	.	.	.
REG00014	CYB561D1	GSE92220	.	.	.	.
REG00014	DAXX	GSE92220	.	.	.	.
REG00014	EGFLAM	GSE92220	.	.	.	.
REG00014	ANKLE1	GSE92220	.	.	.	.
REG00014	RILPL1	GSE92220	.	.	.	.

REG00014	CCDC141	GSE92220	.	.	.	.
REG00014	MPV17L	GSE92220	.	.	.	.
REG00014	DAZAP1	GSE92220	.	.	.	.
REG00014	AMER1	GSE92220	.	.	.	.
REG00014	DAZAP2	GSE92220	.	.	.	.
REG00014	C12orf40	GSE92220	.	.	.	.
REG00014	C2orf61	GSE92220	.	.	.	.
REG00014	HSF5	GSE92220	.	.	.	.
REG00014	CCNYL1	GSE92220	.	.	.	.
REG00014	PARP15	GSE92220	.	.	.	.
REG00014	CKAP2L	GSE92220	.	.	.	.
REG00014	GEN1	GSE92220	.	.	.	.
REG00014	TAPT1	GSE92220	.	.	.	.
REG00014	FAM43A	GSE92220	.	.	.	.
REG00014	TPRN	GSE92220	.	.	.	.
REG00014	DBI	GSE92220	.	.	.	.
REG00014	CCDC96	GSE92220	.	.	.	.
REG00014	RELL2	GSE92220	.	.	.	.
REG00014	LVRN	GSE92220	.	.	.	.
REG00014	INO80E	GSE92220	.	.	.	.
REG00014	SLC38A9	GSE92220	.	.	.	.
REG00014	HTRA4	GSE92220	.	.	.	.
REG00014	SPC24	GSE92220	.	.	.	.
REG00014	N4BP2L2	GSE92220	.	.	.	.
REG00014	NCLN	GSE92220	.	.	.	.
REG00014	JAGN1	GSE92220	.	.	.	.
REG00014	FOXRED1	GSE92220	.	.	.	.
REG00014	CDV3	GSE92220	.	.	.	.
REG00014	C22orf31	GSE92220	.	.	.	.
REG00014	TMA7	GSE92220	.	.	.	.
REG00014	SPOUT1	GSE92220	.	.	.	.
REG00014	NOP16	GSE92220	.	.	.	.
REG00014	CTDSPL2	GSE92220	.	.	.	.
REG00014	TRMT112	GSE92220	.	.	.	.
REG00014	CXXC5	GSE92220	.	.	.	.
REG00014	FMC1	GSE92220	.	.	.	.
REG00014	HYI	GSE92220	.	.	.	.
REG00014	DBN1	GSE92220	.	.	.	.
REG00014	IZUMO4	GSE92220	.	.	.	.
REG00014	TDRP	GSE92220	.	.	.	.
REG00014	KIAA1109	GSE92220	.	.	.	.
REG00014	ZBTB47	GSE92220	.	.	.	.
REG00014	INO80	GSE92220	.	.	.	.
REG00014	GPCPD1	GSE92220	.	.	.	.
REG00014	MROH1	GSE92220	.	.	.	.
REG00014	WDR97	GSE92220	.	.	.	.
REG00014	DBNL	GSE92220	.	.	.	.
REG00014	WDR90	GSE92220	.	.	.	.
REG00014	RNF169	GSE92220	.	.	.	.
REG00014	PTPMT1	GSE92220	.	.	.	.

REG00014	LRRC58	GSE92220	.	.	.	.
REG00014	COX20	GSE92220	.	.	.	.
REG00014	ABHD15	GSE92220	.	.	.	.
REG00014	PDZD8	GSE92220	.	.	.	.
REG00014	ZNF720	GSE92220	.	.	.	.
REG00014	METTL23	GSE92220	.	.	.	.
REG00014	KRBA2	GSE92220	.	.	.	.
REG00014	DDX3Y	GSE92220	.	.	.	.
REG00014	C17orf64	GSE92220	.	.	.	.
REG00014	ZSWIM7	GSE92220	.	.	.	.
REG00014	ZNF787	GSE92220	.	.	.	.
REG00014	PARP1	GSE92220	.	.	.	.
REG00014	MISP	GSE92220	.	.	.	.
REG00014	SHE	GSE92220	.	.	.	.
REG00014	IFFO2	GSE92220	.	.	.	.
REG00014	TPRG1L	GSE92220	.	.	.	.
REG00014	C22orf39	GSE92220	.	.	.	.
REG00014	TRABD2A	GSE92220	.	.	.	.
REG00014	FAM168B	GSE92220	.	.	.	.
REG00014	C5orf47	GSE92220	.	.	.	.
REG00014	ECI1	GSE92220	.	.	.	.
REG00014	DNAJC21	GSE92220	.	.	.	.
REG00014	TMEM171	GSE92220	.	.	.	.
REG00014	SVOPL	GSE92220	.	.	.	.
REG00014	UBXN2B	GSE92220	.	.	.	.
REG00014	DCK	GSE92220	.	.	.	.
REG00014	SPIN4	GSE92220	.	.	.	.
REG00014	LDLRAD3	GSE92220	.	.	.	.
REG00014	PLEKHA7	GSE92220	.	.	.	.
REG00014	ETFRF1	GSE92220	.	.	.	.
REG00014	PGPEP1L	GSE92220	.	.	.	.
REG00014	C16orf52	GSE92220	.	.	.	.
REG00014	VWA3A	GSE92220	.	.	.	.
REG00014	CARMIL2	GSE92220	.	.	.	.
REG00014	DCT	GSE92220	.	.	.	.
REG00014	SLC22A31	GSE92220	.	.	.	.
REG00014	PARP4	GSE92220	.	.	.	.
REG00014	DCTD	GSE92220	.	.	.	.
REG00014	NOTUM	GSE92220	.	.	.	.
REG00014	DCTN1	GSE92220	.	.	.	.
REG00014	VSIG10L	GSE92220	.	.	.	.
REG00014	FAM98C	GSE92220	.	.	.	.
REG00014	DCTN2	GSE92220	.	.	.	.
REG00014	DPY19L3	GSE92220	.	.	.	.
REG00014	SHISA4	GSE92220	.	.	.	.
REG00014	BTBD19	GSE92220	.	.	.	.
REG00014	RNF187	GSE92220	.	.	.	.
REG00014	DDAH1	GSE92220	.	.	.	.
REG00014	YDJC	GSE92220	.	.	.	.
REG00014	DDAH2	GSE92220	.	.	.	.

REG00014	DDB1	GSE92220	.	.	.	.
REG00014	GAREM2	GSE92220	.	.	.	.
REG00014	DDB2	GSE92220	.	.	.	.
REG00014	DDC	GSE92220	.	.	.	.
REG00014	ZNF827	GSE92220	.	.	.	.
REG00014	PARP2	GSE92220	.	.	.	.
REG00014	ASAP1	GSE92220	.	.	.	.
REG00014	ASAP2	GSE92220	.	.	.	.
REG00014	RBM33	GSE92220	.	.	.	.
REG00014	ERICH1	GSE92220	.	.	.	.
REG00014	ITPRIPL2	GSE92220	.	.	.	.
REG00014	FAM134C	GSE92220	.	.	.	.
REG00014	KANK4	GSE92220	.	.	.	.
REG00014	ZNF800	GSE92220	.	.	.	.
REG00014	ZCCHC12	GSE92220	.	.	.	.
REG00014	MPZL3	GSE92220	.	.	.	.
REG00014	DDOST	GSE92220	.	.	.	.
REG00014	GRAMD2	GSE92220	.	.	.	.
REG00014	ACSF3	GSE92220	.	.	.	.
REG00014	EME2	GSE92220	.	.	.	.
REG00014	ADM5	GSE92220	.	.	.	.
REG00014	PARP3	GSE92220	.	.	.	.
REG00014	CRTC2	GSE92220	.	.	.	.
REG00014	IBA57	GSE92220	.	.	.	.
REG00014	DHFR2	GSE92220	.	.	.	.
REG00014	DDR2	GSE92220	.	.	.	.
REG00014	FLCN	GSE92220	.	.	.	.
REG00014	SAMD14	GSE92220	.	.	.	.
REG00014	TRIM65	GSE92220	.	.	.	.
REG00014	ZNF584	GSE92220	.	.	.	.
REG00014	DDT	GSE92220	.	.	.	.
REG00014	DDX1	GSE92220	.	.	.	.
REG00014	DDX10	GSE92220	.	.	.	.
REG00014	EEF1AKMT1	GSE92220	.	.	.	.
REG00014	DHX15	GSE92220	.	.	.	.
REG00014	ASPHD1	GSE92220	.	.	.	.
REG00014	ZDHHC24	GSE92220	.	.	.	.
REG00014	DHX16	GSE92220	.	.	.	.
REG00014	PLA2G4F	GSE92220	.	.	.	.
REG00014	DDX17	GSE92220	.	.	.	.
REG00014	INAFM1	GSE92220	.	.	.	.
REG00014	DDX18	GSE92220	.	.	.	.
REG00014	SYCE2	GSE92220	.	.	.	.
REG00014	C2orf72	GSE92220	.	.	.	.
REG00014	DDX19B	GSE92220	.	.	.	.
REG00014	DDX21	GSE92220	.	.	.	.
REG00014	DDX3X	GSE92220	.	.	.	.
REG00014	TMEM80	GSE92220	.	.	.	.
REG00014	RCOR2	GSE92220	.	.	.	.
REG00014	DDX5	GSE92220	.	.	.	.

REG00014	ZNF740	GSE92220	.	.	.	.
REG00014	SPRYD4	GSE92220	.	.	.	.
REG00014	DDX6	GSE92220	.	.	.	.
REG00014	GAS2L3	GSE92220	.	.	.	.
REG00014	GXYLT1	GSE92220	.	.	.	.
REG00014	DHX8	GSE92220	.	.	.	.
REG00014	DHX9	GSE92220	.	.	.	.
REG00014	KLHL23	GSE92220	.	.	.	.
REG00014	PIAS1	GSE92220	.	.	.	.
REG00014	DECR1	GSE92220	.	.	.	.
REG00014	FBXL22	GSE92220	.	.	.	.
REG00014	DECR2	GSE92220	.	.	.	.
REG00014	TSEN54	GSE92220	.	.	.	.
REG00014	TMEM235	GSE92220	.	.	.	.
REG00014	CCDC57	GSE92220	.	.	.	.
REG00014	C17orf58	GSE92220	.	.	.	.
REG00014	CISD3	GSE92220	.	.	.	.
REG00014	SIGLEC15	GSE92220	.	.	.	.
REG00014	ZNF780A	GSE92220	.	.	.	.
REG00014	ZNF575	GSE92220	.	.	.	.
REG00014	PPP1R37	GSE92220	.	.	.	.
REG00014	RPL22L1	GSE92220	.	.	.	.
REG00014	ZNF763	GSE92220	.	.	.	.
REG00014	SLC25A34	GSE92220	.	.	.	.
REG00014	LINC00176	GSE92220	.	.	.	.
REG00014	DEK	GSE92220	.	.	.	.
REG00014	FAM150B	GSE92220	.	.	.	.
REG00014	DENR	GSE92220	.	.	.	.
REG00014	IAH1	GSE92220	.	.	.	.
REG00014	CFD	GSE92220	.	.	.	.
REG00014	DPH3	GSE92220	.	.	.	.
REG00014	DFFA	GSE92220	.	.	.	.
REG00014	DCAF4L1	GSE92220	.	.	.	.
REG00014	MROH6	GSE92220	.	.	.	.
REG00014	TRIQK	GSE92220	.	.	.	.
REG00014	C9orf142	GSE92220	.	.	.	.
REG00014	ANKRD13D	GSE92220	.	.	.	.
REG00014	C17orf67	GSE92220	.	.	.	.
REG00014	OXLD1	GSE92220	.	.	.	.
REG00014	ARL16	GSE92220	.	.	.	.
REG00014	MSL1	GSE92220	.	.	.	.
REG00014	ADAMTSL5	GSE92220	.	.	.	.
REG00014	SPOPL	GSE92220	.	.	.	.
REG00014	ESPNL	GSE92220	.	.	.	.
REG00014	FAM170A	GSE92220	.	.	.	.
REG00014	MYLK4	GSE92220	.	.	.	.
REG00014	AGBL3	GSE92220	.	.	.	.
REG00014	MCRIP1	GSE92220	.	.	.	.
REG00014	LNP1	GSE92220	.	.	.	.
REG00014	TXNDC11	GSE92220	.	.	.	.

REG00014	DRICH1	GSE92220	.	.	.	.
REG00014	EMC4	GSE92220	.	.	.	.
REG00014	PDZD11	GSE92220	.	.	.	.
REG00014	TMEM69	GSE92220	.	.	.	.
REG00014	RNF181	GSE92220	.	.	.	.
REG00014	MARCHF2	GSE92220	.	.	.	.
REG00014	KRCC1	GSE92220	.	.	.	.
REG00014	MEX3C	GSE92220	.	.	.	.
REG00014	AMZ2	GSE92220	.	.	.	.
REG00014	GSAP	GSE92220	.	.	.	.
REG00014	HIGD1C	GSE92220	.	.	.	.
REG00014	FAM212B	GSE92220	.	.	.	.
REG00014	CIAPIN1	GSE92220	.	.	.	.
REG00014	CCDC181	GSE92220	.	.	.	.
REG00014	SAPCD2	GSE92220	.	.	.	.
REG00014	FER1L6	GSE92220	.	.	.	.
REG00014	ZNF799	GSE92220	.	.	.	.
REG00014	ZFAND2A	GSE92220	.	.	.	.
REG00014	COX19	GSE92220	.	.	.	.
REG00014	ZNF598	GSE92220	.	.	.	.
REG00014	UAP1L1	GSE92220	.	.	.	.
REG00014	NDUFAF2	GSE92220	.	.	.	.
REG00014	BICDL1	GSE92220	.	.	.	.
REG00014	DFNA5	GSE92220	.	.	.	.
REG00014	MMGT1	GSE92220	.	.	.	.
REG00014	TMEM97	GSE92220	.	.	.	.
REG00014	PPCDC	GSE92220	.	.	.	.
REG00014	ALDH16A1	GSE92220	.	.	.	.
REG00014	RBM42	GSE92220	.	.	.	.
REG00014	PRRC2B	GSE92220	.	.	.	.
REG00014	VPS25	GSE92220	.	.	.	.
REG00014	PRR7	GSE92220	.	.	.	.
REG00014	TCHP	GSE92220	.	.	.	.
REG00014	FBXW9	GSE92220	.	.	.	.
REG00014	SPX	GSE92220	.	.	.	.
REG00014	LIMD2	GSE92220	.	.	.	.
REG00014	TMEM177	GSE92220	.	.	.	.
REG00014	YIPF4	GSE92220	.	.	.	.
REG00014	EIF1AD	GSE92220	.	.	.	.
REG00014	CARD19	GSE92220	.	.	.	.
REG00014	CENPO	GSE92220	.	.	.	.
REG00014	C19orf57	GSE92220	.	.	.	.
REG00014	NUDT16L1	GSE92220	.	.	.	.
REG00014	MFSD5	GSE92220	.	.	.	.
REG00014	MFSD9	GSE92220	.	.	.	.
REG00014	GFOD2	GSE92220	.	.	.	.
REG00014	ZXDC	GSE92220	.	.	.	.
REG00014	CCDC28B	GSE92220	.	.	.	.
REG00014	PRRC1	GSE92220	.	.	.	.
REG00014	EFCAB2	GSE92220	.	.	.	.

REG00014	HDHD3	GSE92220	.	.	.	.
REG00014	C18orf25	GSE92220	.	.	.	.
REG00014	CCDC115	GSE92220	.	.	.	.
REG00014	ORAI3	GSE92220	.	.	.	.
REG00014	NUDT22	GSE92220	.	.	.	.
REG00014	PDCD2L	GSE92220	.	.	.	.
REG00014	BUD13	GSE92220	.	.	.	.
REG00014	ZNF764	GSE92220	.	.	.	.
REG00014	RHNO1	GSE92220	.	.	.	.
REG00014	MON1A	GSE92220	.	.	.	.
REG00014	PERM1	GSE92220	.	.	.	.
REG00014	TMEM141	GSE92220	.	.	.	.
REG00014	NAA38	GSE92220	.	.	.	.
REG00014	COX14	GSE92220	.	.	.	.
REG00014	TXNDC17	GSE92220	.	.	.	.
REG00014	UBL7	GSE92220	.	.	.	.
REG00014	LRRC39	GSE92220	.	.	.	.
REG00014	LLPH	GSE92220	.	.	.	.
REG00014	MIEN1	GSE92220	.	.	.	.
REG00014	ANKRD40	GSE92220	.	.	.	.
REG00014	ABHD14B	GSE92220	.	.	.	.
REG00014	CCDC183	GSE92220	.	.	.	.
REG00014	SLC38A10	GSE92220	.	.	.	.
REG00014	PHYKPL	GSE92220	.	.	.	.
REG00014	TBCK	GSE92220	.	.	.	.
REG00014	PCGF5	GSE92220	.	.	.	.
REG00014	FAM86B1	GSE92220	.	.	.	.
REG00014	R3HDM4	GSE92220	.	.	.	.
REG00014	METTL7B	GSE92220	.	.	.	.
REG00014	TNFAIP8L1	GSE92220	.	.	.	.
REG00014	TMEM263	GSE92220	.	.	.	.
REG00014	GSG1L	GSE92220	.	.	.	.
REG00014	TMEM106A	GSE92220	.	.	.	.
REG00014	CCDC97	GSE92220	.	.	.	.
REG00014	C15orf57	GSE92220	.	.	.	.
REG00014	WDR34	GSE92220	.	.	.	.
REG00014	MFSD12	GSE92220	.	.	.	.
REG00014	ADRA2C	GSE92220	.	.	.	.
REG00014	DOK6	GSE92220	.	.	.	.
REG00014	LRRC45	GSE92220	.	.	.	.
REG00014	CCDC151	GSE92220	.	.	.	.
REG00014	YIPF6	GSE92220	.	.	.	.
REG00014	FAM131A	GSE92220	.	.	.	.
REG00014	BRICD5	GSE92220	.	.	.	.
REG00014	HIGD2A	GSE92220	.	.	.	.
REG00014	TET3	GSE92220	.	.	.	.
REG00014	PHOSPHO2	GSE92220	.	.	.	.
REG00014	PPP1R35	GSE92220	.	.	.	.
REG00014	ZNF524	GSE92220	.	.	.	.
REG00014	ZBTB9	GSE92220	.	.	.	.

REG00014	DHRS13	GSE92220	.	.	.	.
REG00014	TMED6	GSE92220	.	.	.	.
REG00014	CCDC12	GSE92220	.	.	.	.
REG00014	DENND5B	GSE92220	.	.	.	.
REG00014	C1orf64	GSE92220	.	.	.	.
REG00014	FAM210A	GSE92220	.	.	.	.
REG00014	RMI2	GSE92220	.	.	.	.
REG00014	ZNF747	GSE92220	.	.	.	.
REG00014	LBHD1	GSE92220	.	.	.	.
REG00014	TBC1D16	GSE92220	.	.	.	.
REG00014	D2HGDH	GSE92220	.	.	.	.
REG00014	DDA1	GSE92220	.	.	.	.
REG00014	AUNIP	GSE92220	.	.	.	.
REG00014	METTL22	GSE92220	.	.	.	.
REG00014	THOC6	GSE92220	.	.	.	.
REG00014	LRFN3	GSE92220	.	.	.	.
REG00014	SLC25A42	GSE92220	.	.	.	.
REG00014	FAM213B	GSE92220	.	.	.	.
REG00014	PP2D1	GSE92220	.	.	.	.
REG00014	SENP5	GSE92220	.	.	.	.
REG00014	DCUN1D5	GSE92220	.	.	.	.
REG00014	RBM18	GSE92220	.	.	.	.
REG00014	SMIM7	GSE92220	.	.	.	.
REG00014	KXD1	GSE92220	.	.	.	.
REG00014	STAC3	GSE92220	.	.	.	.
REG00014	C19orf43	GSE92220	.	.	.	.
REG00014	AKT1S1	GSE92220	.	.	.	.
REG00014	PHF23	GSE92220	.	.	.	.
REG00014	DGAT1	GSE92220	.	.	.	.
REG00014	EMC6	GSE92220	.	.	.	.
REG00014	C9orf116	GSE92220	.	.	.	.
REG00014	C2orf15	GSE92220	.	.	.	.
REG00014	RPUSD3	GSE92220	.	.	.	.
REG00014	UBALD2	GSE92220	.	.	.	.
REG00014	CHMP7	GSE92220	.	.	.	.
REG00014	DIEXF	GSE92220	.	.	.	.
REG00014	C15orf40	GSE92220	.	.	.	.
REG00014	TMEM42	GSE92220	.	.	.	.
REG00014	ZNF511	GSE92220	.	.	.	.
REG00014	TMEM86B	GSE92220	.	.	.	.
REG00014	C11orf95	GSE92220	.	.	.	.
REG00014	FAM134A	GSE92220	.	.	.	.
REG00014	DERL1	GSE92220	.	.	.	.
REG00014	DBNDD1	GSE92220	.	.	.	.
REG00014	LRFN4	GSE92220	.	.	.	.
REG00014	PRR14	GSE92220	.	.	.	.
REG00014	TMUB2	GSE92220	.	.	.	.
REG00014	C17orf53	GSE92220	.	.	.	.
REG00014	NOC4L	GSE92220	.	.	.	.
REG00014	TMEM38A	GSE92220	.	.	.	.

REG00014	TMEM223	GSE92220	.	.	.	.
REG00014	FAAP24	GSE92220	.	.	.	.
REG00014	SNX33	GSE92220	.	.	.	.
REG00014	MRI1	GSE92220	.	.	.	.
REG00014	DGCR8	GSE92220	.	.	.	.
REG00014	CYB5D2	GSE92220	.	.	.	.
REG00014	TMEM43	GSE92220	.	.	.	.
REG00014	NOA1	GSE92220	.	.	.	.
REG00014	CHID1	GSE92220	.	.	.	.
REG00014	YIPF2	GSE92220	.	.	.	.
REG00014	TMEM161B	GSE92220	.	.	.	.
REG00014	C3orf58	GSE92220	.	.	.	.
REG00014	C10orf82	GSE92220	.	.	.	.
REG00014	PQLC3	GSE92220	.	.	.	.
REG00014	SETD9	GSE92220	.	.	.	.
REG00014	DGKD	GSE92220	.	.	.	.
REG00014	KIAA2013	GSE92220	.	.	.	.
REG00014	C11orf65	GSE92220	.	.	.	.
REG00014	DGKE	GSE92220	.	.	.	.
REG00014	EOGT	GSE92220	.	.	.	.
REG00014	UGT2A3	GSE92220	.	.	.	.
REG00014	IL34	GSE92220	.	.	.	.
REG00014	TYSND1	GSE92220	.	.	.	.
REG00014	KIAA0825	GSE92220	.	.	.	.
REG00014	SLC25A41	GSE92220	.	.	.	.
REG00014	C3orf22	GSE92220	.	.	.	.
REG00014	CCDC83	GSE92220	.	.	.	.
REG00014	C11orf42	GSE92220	.	.	.	.
REG00014	DGKI	GSE92220	.	.	.	.
REG00014	IQCK	GSE92220	.	.	.	.
REG00014	DGKQ	GSE92220	.	.	.	.
REG00014	LRRC43	GSE92220	.	.	.	.
REG00014	DGKZ	GSE92220	.	.	.	.
REG00014	PRR18	GSE92220	.	.	.	.
REG00014	PSTK	GSE92220	.	.	.	.
REG00014	DGUOK	GSE92220	.	.	.	.
REG00014	NOL12	GSE92220	.	.	.	.
REG00014	CXorf38	GSE92220	.	.	.	.
REG00014	DHCR24	GSE92220	.	.	.	.
REG00014	HEXIM2	GSE92220	.	.	.	.
REG00014	B3GALNT2	GSE92220	.	.	.	.
REG00014	DHCR7	GSE92220	.	.	.	.
REG00014	PROCA1	GSE92220	.	.	.	.
REG00014	ADIG	GSE92220	.	.	.	.
REG00014	DHFR	GSE92220	.	.	.	.
REG00014	RICTOR	GSE92220	.	.	.	.
REG00014	CBARP	GSE92220	.	.	.	.
REG00014	TMEM256	GSE92220	.	.	.	.
REG00014	FBXL14	GSE92220	.	.	.	.
REG00014	TCP11L2	GSE92220	.	.	.	.

REG00014	C12orf45	GSE92220	.	.	.	.
REG00014	FAM133B	GSE92220	.	.	.	.
REG00014	ZNF557	GSE92220	.	.	.	.
REG00014	ARRDC1	GSE92220	.	.	.	.
REG00014	GK5	GSE92220	.	.	.	.
REG00014	B9D2	GSE92220	.	.	.	.
REG00014	LRCH3	GSE92220	.	.	.	.
REG00014	GDPD3	GSE92220	.	.	.	.
REG00014	DHRS11	GSE92220	.	.	.	.
REG00014	ANKRD39	GSE92220	.	.	.	.
REG00014	RPAIN	GSE92220	.	.	.	.
REG00014	DIS3L2	GSE92220	.	.	.	.
REG00014	DHH	GSE92220	.	.	.	.
REG00014	FAM213A	GSE92220	.	.	.	.
REG00014	TMEM101	GSE92220	.	.	.	.
REG00014	ZDHHC23	GSE92220	.	.	.	.
REG00014	AMMECR1L	GSE92220	.	.	.	.
REG00014	ISCA1	GSE92220	.	.	.	.
REG00014	DOHH	GSE92220	.	.	.	.
REG00014	C17orf62	GSE92220	.	.	.	.
REG00014	CHAC1	GSE92220	.	.	.	.
REG00014	ZNF561	GSE92220	.	.	.	.
REG00014	CCDC24	GSE92220	.	.	.	.
REG00014	DHPS	GSE92220	.	.	.	.
REG00014	ELOF1	GSE92220	.	.	.	.
REG00014	ZADH2	GSE92220	.	.	.	.
REG00014	RAB42	GSE92220	.	.	.	.
REG00014	CRACR2B	GSE92220	.	.	.	.
REG00014	RFLNB	GSE92220	.	.	.	.
REG00014	PAGR1	GSE92220	.	.	.	.
REG00014	TMEM175	GSE92220	.	.	.	.
REG00014	B3GNT9	GSE92220	.	.	.	.
REG00014	RNF183	GSE92220	.	.	.	.
REG00014	CYB5R3	GSE92220	.	.	.	.
REG00014	C1QTNF9	GSE92220	.	.	.	.
REG00014	LCN12	GSE92220	.	.	.	.
REG00014	C17orf49	GSE92220	.	.	.	.
REG00014	PRR14L	GSE92220	.	.	.	.
REG00014	NQO1	GSE92220	.	.	.	.
REG00014	PDZD9	GSE92220	.	.	.	.
REG00014	TSPAN33	GSE92220	.	.	.	.
REG00014	CNIH2	GSE92220	.	.	.	.
REG00014	OAF	GSE92220	.	.	.	.
REG00014	ABHD17A	GSE92220	.	.	.	.
REG00014	TAF1D	GSE92220	.	.	.	.
REG00014	DIAPH1	GSE92220	.	.	.	.
REG00014	MED10	GSE92220	.	.	.	.
REG00014	PGAM5	GSE92220	.	.	.	.
REG00014	FNDC11	GSE92220	.	.	.	.
REG00014	SFT2D3	GSE92220	.	.	.	.

REG00014	C2orf49	GSE92220	.	.	.	.
REG00014	TMEM106C	GSE92220	.	.	.	.
REG00014	DCTPP1	GSE92220	.	.	.	.
REG00014	MDP1	GSE92220	.	.	.	.
REG00014	SARAF	GSE92220	.	.	.	.
REG00014	LRRC42	GSE92220	.	.	.	.
REG00014	MRFAP1L1	GSE92220	.	.	.	.
REG00014	TMEM184A	GSE92220	.	.	.	.
REG00014	C11orf68	GSE92220	.	.	.	.
REG00014	C18orf21	GSE92220	.	.	.	.
REG00014	TRABD	GSE92220	.	.	.	.
REG00014	AP1AR	GSE92220	.	.	.	.
REG00014	NDUFAF7	GSE92220	.	.	.	.
REG00014	LRRC59	GSE92220	.	.	.	.
REG00014	SH2D5	GSE92220	.	.	.	.
REG00014	WDR82	GSE92220	.	.	.	.
REG00014	DIO1	GSE92220	.	.	.	.
REG00014	OLA1	GSE92220	.	.	.	.
REG00014	IRGC	GSE92220	.	.	.	.
REG00014	MTFR1L	GSE92220	.	.	.	.
REG00014	TMEM234	GSE92220	.	.	.	.
REG00014	RBM4B	GSE92220	.	.	.	.
REG00014	GPBP1L1	GSE92220	.	.	.	.
REG00014	MED25	GSE92220	.	.	.	.
REG00014	TSKU	GSE92220	.	.	.	.
REG00014	RNF166	GSE92220	.	.	.	.
REG00014	P4HTM	GSE92220	.	.	.	.
REG00014	NIPBL	GSE92220	.	.	.	.
REG00014	IGF2BP1	GSE92220	.	.	.	.
REG00014	IGF2BP2	GSE92220	.	.	.	.
REG00014	IER2	GSE92220	.	.	.	.
REG00014	DISC1	GSE92220	.	.	.	.
REG00014	PATJ	GSE92220	.	.	.	.
REG00014	LEMD3	GSE92220	.	.	.	.
REG00014	TMED3	GSE92220	.	.	.	.
REG00014	SYNC	GSE92220	.	.	.	.
REG00014	ADM2	GSE92220	.	.	.	.
REG00014	GRK2	GSE92220	.	.	.	.
REG00014	YRDC	GSE92220	.	.	.	.
REG00014	DKK1	GSE92220	.	.	.	.
REG00014	PRAF2	GSE92220	.	.	.	.
REG00014	WDR45	GSE92220	.	.	.	.
REG00014	HSCB	GSE92220	.	.	.	.
REG00014	JMY	GSE92220	.	.	.	.
REG00014	NAT14	GSE92220	.	.	.	.
REG00014	CEP170	GSE92220	.	.	.	.
REG00014	DAGLB	GSE92220	.	.	.	.
REG00014	DKK3	GSE92220	.	.	.	.
REG00014	KRTCAP2	GSE92220	.	.	.	.
REG00014	KRTCAP3	GSE92220	.	.	.	.

REG00014	WDR43	GSE92220	.	.	.	.
REG00014	LRRC24	GSE92220	.	.	.	.
REG00014	NCAPD3	GSE92220	.	.	.	.
REG00014	GPD1L	GSE92220	.	.	.	.
REG00014	EMC1	GSE92220	.	.	.	.
REG00014	CKAP5	GSE92220	.	.	.	.
REG00014	DLAT	GSE92220	.	.	.	.
REG00014	KIAA0100	GSE92220	.	.	.	.
REG00014	SPCS2	GSE92220	.	.	.	.
REG00014	RBM34	GSE92220	.	.	.	.
REG00014	VGLL4	GSE92220	.	.	.	.
REG00014	URB2	GSE92220	.	.	.	.
REG00014	KIAA0141	GSE92220	.	.	.	.
REG00014	DLC1	GSE92220	.	.	.	.
REG00014	ZC3H3	GSE92220	.	.	.	.
REG00014	MLEC	GSE92220	.	.	.	.
REG00014	TTLL12	GSE92220	.	.	.	.
REG00014	FAM175B	GSE92220	.	.	.	.
REG00014	TTLL4	GSE92220	.	.	.	.
REG00014	IST1	GSE92220	.	.	.	.
REG00014	GSE1	GSE92220	.	.	.	.
REG00014	DLD	GSE92220	.	.	.	.
REG00014	GINS1	GSE92220	.	.	.	.
REG00014	HMGXB3	GSE92220	.	.	.	.
REG00014	TMEM94	GSE92220	.	.	.	.
REG00014	LPGAT1	GSE92220	.	.	.	.
REG00014	LARP4B	GSE92220	.	.	.	.
REG00014	TATDN2	GSE92220	.	.	.	.
REG00014	NPIPB3	GSE92220	.	.	.	.
REG00014	ZNF516	GSE92220	.	.	.	.
REG00014	KIAA0232	GSE92220	.	.	.	.
REG00014	PIEZO1	GSE92220	.	.	.	.
REG00014	AVL9	GSE92220	.	.	.	.
REG00014	PDXDC1	GSE92220	.	.	.	.
REG00014	DCUN1D4	GSE92220	.	.	.	.
REG00014	FAM168A	GSE92220	.	.	.	.
REG00014	ABCA1	GSE92220	.	.	.	.
REG00014	DLG1	GSE92220	.	.	.	.
REG00014	C2CD2L	GSE92220	.	.	.	.
REG00014	ZNF609	GSE92220	.	.	.	.
REG00014	ZNF646	GSE92220	.	.	.	.
REG00014	SEC16A	GSE92220	.	.	.	.
REG00014	ZNF629	GSE92220	.	.	.	.
REG00014	ZNF518A	GSE92220	.	.	.	.
REG00014	SETD1A	GSE92220	.	.	.	.
REG00014	KDM6B	GSE92220	.	.	.	.
REG00014	CLEC16A	GSE92220	.	.	.	.
REG00014	ZBTB39	GSE92220	.	.	.	.
REG00014	KIAA0355	GSE92220	.	.	.	.
REG00014	KIAA0368	GSE92220	.	.	.	.

REG00014	SPECC1L	GSE92220	.	.	.	.
REG00014	ANKRD28	GSE92220	.	.	.	.
REG00014	SGSM2	GSE92220	.	.	.	.
REG00014	ATG2A	GSE92220	.	.	.	.
REG00014	TTI1	GSE92220	.	.	.	.
REG00014	DLG4	GSE92220	.	.	.	.
REG00014	ATMIN	GSE92220	.	.	.	.
REG00014	RPRD2	GSE92220	.	.	.	.
REG00014	DLG5	GSE92220	.	.	.	.
REG00014	KLHL21	GSE92220	.	.	.	.
REG00014	DENND4B	GSE92220	.	.	.	.
REG00014	ZBTB40	GSE92220	.	.	.	.
REG00014	C2CD5	GSE92220	.	.	.	.
REG00014	GPATCH8	GSE92220	.	.	.	.
REG00014	DLK1	GSE92220	.	.	.	.
REG00014	LRRC37B	GSE92220	.	.	.	.
REG00014	VWA8	GSE92220	.	.	.	.
REG00014	CEP68	GSE92220	.	.	.	.
REG00014	IFT140	GSE92220	.	.	.	.
REG00014	KDM1A	GSE92220	.	.	.	.
REG00014	ZNF623	GSE92220	.	.	.	.
REG00014	PDS5A	GSE92220	.	.	.	.
REG00014	PPP1R26	GSE92220	.	.	.	.
REG00014	DLL3	GSE92220	.	.	.	.
REG00014	SMCHD1	GSE92220	.	.	.	.
REG00014	ATG13	GSE92220	.	.	.	.
REG00014	OBSL1	GSE92220	.	.	.	.
REG00014	ZC3H11A	GSE92220	.	.	.	.
REG00014	CLUH	GSE92220	.	.	.	.
REG00014	TSC22D2	GSE92220	.	.	.	.
REG00014	TBC1D9B	GSE92220	.	.	.	.
REG00014	RBM19	GSE92220	.	.	.	.
REG00014	TELO2	GSE92220	.	.	.	.
REG00014	ADSL	GSE92220	.	.	.	.
REG00014	RRP12	GSE92220	.	.	.	.
REG00014	ANKLE2	GSE92220	.	.	.	.
REG00014	PLEKHG5	GSE92220	.	.	.	.
REG00014	DDHD2	GSE92220	.	.	.	.
REG00014	DLST	GSE92220	.	.	.	.
REG00014	IQSEC1	GSE92220	.	.	.	.
REG00014	GRAMD4	GSE92220	.	.	.	.
REG00014	FCHSD2	GSE92220	.	.	.	.
REG00014	TMCC1	GSE92220	.	.	.	.
REG00014	TMEM63A	GSE92220	.	.	.	.
REG00014	FRYL	GSE92220	.	.	.	.
REG00014	HAUS5	GSE92220	.	.	.	.
REG00014	KDM4B	GSE92220	.	.	.	.
REG00014	FAM169A	GSE92220	.	.	.	.
REG00014	MAU2	GSE92220	.	.	.	.
REG00014	EHBP1	GSE92220	.	.	.	.

REG00014	KIAA0907	GSE92220	.	.	.	.
REG00014	ZNF652	GSE92220	.	.	.	.
REG00014	PHLPP2	GSE92220	.	.	.	.
REG00014	DIP2C	GSE92220	.	.	.	.
REG00014	DTX4	GSE92220	.	.	.	.
REG00014	BAHD1	GSE92220	.	.	.	.
REG00014	ICE1	GSE92220	.	.	.	.
REG00014	SBNO2	GSE92220	.	.	.	.
REG00014	ZNF510	GSE92220	.	.	.	.
REG00014	NINL	GSE92220	.	.	.	.
REG00014	SIK3	GSE92220	.	.	.	.
REG00014	TRAPPC8	GSE92220	.	.	.	.
REG00014	FAN1	GSE92220	.	.	.	.
REG00014	KAZN	GSE92220	.	.	.	.
REG00014	WASHC4	GSE92220	.	.	.	.
REG00014	WDTC1	GSE92220	.	.	.	.
REG00014	FOXJ3	GSE92220	.	.	.	.
REG00014	TTC28	GSE92220	.	.	.	.
REG00014	TCF25	GSE92220	.	.	.	.
REG00014	CEP164	GSE92220	.	.	.	.
REG00014	TBC1D2B	GSE92220	.	.	.	.
REG00014	ANKRD26	GSE92220	.	.	.	.
REG00014	SETD1B	GSE92220	.	.	.	.
REG00014	TNRC6B	GSE92220	.	.	.	.
REG00014	PPP6R1	GSE92220	.	.	.	.
REG00014	CCSER2	GSE92220	.	.	.	.
REG00014	KIAA1143	GSE92220	.	.	.	.
REG00014	ADSS	GSE92220	.	.	.	.
REG00014	ISY1	GSE92220	.	.	.	.
REG00014	ERGIC1	GSE92220	.	.	.	.
REG00014	LRRC47	GSE92220	.	.	.	.
REG00014	KIAA1191	GSE92220	.	.	.	.
REG00014	MIER2	GSE92220	.	.	.	.
REG00014	ZNF512B	GSE92220	.	.	.	.
REG00014	CEMIP	GSE92220	.	.	.	.
REG00014	PRR12	GSE92220	.	.	.	.
REG00014	ZNF644	GSE92220	.	.	.	.
REG00014	HEG1	GSE92220	.	.	.	.
REG00014	ATAD2B	GSE92220	.	.	.	.
REG00014	PARP14	GSE92220	.	.	.	.
REG00014	MAVS	GSE92220	.	.	.	.
REG00014	WWC3	GSE92220	.	.	.	.
REG00014	INTS2	GSE92220	.	.	.	.
REG00014	RBM27	GSE92220	.	.	.	.
REG00014	TBC1D14	GSE92220	.	.	.	.
REG00014	KIAA1328	GSE92220	.	.	.	.
REG00014	SOBP	GSE92220	.	.	.	.
REG00014	TAOK1	GSE92220	.	.	.	.
REG00014	NCEH1	GSE92220	.	.	.	.
REG00014	KLHL14	GSE92220	.	.	.	.

REG00014	PPP4R3B	GSE92220	.	.	.	.
REG00014	CHPF2	GSE92220	.	.	.	.
REG00014	HEATR5B	GSE92220	.	.	.	.
REG00014	CFAP97	GSE92220	.	.	.	.
REG00014	ZNF687	GSE92220	.	.	.	.
REG00014	BAHCC1	GSE92220	.	.	.	.
REG00014	DMD	GSE92220	.	.	.	.
REG00014	FNIP2	GSE92220	.	.	.	.
REG00014	SLAIN2	GSE92220	.	.	.	.
REG00014	DIP2B	GSE92220	.	.	.	.
REG00014	ARHGAP23	GSE92220	.	.	.	.
REG00014	ZSWIM5	GSE92220	.	.	.	.
REG00014	KIAA1522	GSE92220	.	.	.	.
REG00014	UVSSA	GSE92220	.	.	.	.
REG00014	GRAMD1A	GSE92220	.	.	.	.
REG00014	CALCOCO1	GSE92220	.	.	.	.
REG00014	CAMSAP3	GSE92220	.	.	.	.
REG00014	FBRSL1	GSE92220	.	.	.	.
REG00014	TNRC6C	GSE92220	.	.	.	.
REG00014	SHTN1	GSE92220	.	.	.	.
REG00014	FAM160B1	GSE92220	.	.	.	.
REG00014	NCKAP5L	GSE92220	.	.	.	.
REG00014	DENND1A	GSE92220	.	.	.	.
REG00014	TLDC1	GSE92220	.	.	.	.
REG00014	KIAA1614	GSE92220	.	.	.	.
REG00014	ANO8	GSE92220	.	.	.	.
REG00014	DMPK	GSE92220	.	.	.	.
REG00014	METTL14	GSE92220	.	.	.	.
REG00014	ZC3H12C	GSE92220	.	.	.	.
REG00014	TSPYL5	GSE92220	.	.	.	.
REG00014	UNK	GSE92220	.	.	.	.
REG00014	ITPRIPL1	GSE92220	.	.	.	.
REG00014	PHYHIPL	GSE92220	.	.	.	.
REG00014	LZTS2	GSE92220	.	.	.	.
REG00014	MSANTD4	GSE92220	.	.	.	.
REG00014	ZNF527	GSE92220	.	.	.	.
REG00014	CC2D1B	GSE92220	.	.	.	.
REG00014	KIAA1841	GSE92220	.	.	.	.
REG00014	PLIN4	GSE92220	.	.	.	.
REG00014	DNAH1	GSE92220	.	.	.	.
REG00014	MYSM1	GSE92220	.	.	.	.
REG00014	PWWP2A	GSE92220	.	.	.	.
REG00014	FBXO41	GSE92220	.	.	.	.
REG00014	DNAH10	GSE92220	.	.	.	.
REG00014	FAM171B	GSE92220	.	.	.	.
REG00014	PPP1R18	GSE92220	.	.	.	.
REG00014	ZNF526	GSE92220	.	.	.	.
REG00014	C2CD4C	GSE92220	.	.	.	.
REG00014	FNIP1	GSE92220	.	.	.	.
REG00014	RSPRY1	GSE92220	.	.	.	.

REG00014	ANKS3	GSE92220	.	.	.	.
REG00014	PEAK1	GSE92220	.	.	.	.
REG00014	RC3H1	GSE92220	.	.	.	.
REG00014	WWC1	GSE92220	.	.	.	.
REG00014	GLMP	GSE92220	.	.	.	.
REG00014	DNAH14	GSE92220	.	.	.	.
REG00014	GLIS2	GSE92220	.	.	.	.
REG00014	FGFBP2	GSE92220	.	.	.	.
REG00014	TOR1AIP1	GSE92220	.	.	.	.
REG00014	KIAA1147	GSE92220	.	.	.	.
REG00014	ZNF580	GSE92220	.	.	.	.
REG00014	LEPROT	GSE92220	.	.	.	.
REG00014	ROGDI	GSE92220	.	.	.	.
REG00014	LRG1	GSE92220	.	.	.	.
REG00014	TET1	GSE92220	.	.	.	.
REG00014	KMT5A	GSE92220	.	.	.	.
REG00014	FUK	GSE92220	.	.	.	.
REG00014	LCOR	GSE92220	.	.	.	.
REG00014	ZACN	GSE92220	.	.	.	.
REG00014	SRCIN1	GSE92220	.	.	.	.
REG00014	APH1A	GSE92220	.	.	.	.
REG00014	MTFR1	GSE92220	.	.	.	.
REG00014	CEP131	GSE92220	.	.	.	.
REG00014	AQR	GSE92220	.	.	.	.
REG00014	PLEKHG2	GSE92220	.	.	.	.
REG00014	DHX58	GSE92220	.	.	.	.
REG00014	C12orf57	GSE92220	.	.	.	.
REG00014	SPSB2	GSE92220	.	.	.	.
REG00014	CFAP20	GSE92220	.	.	.	.
REG00014	GLIS1	GSE92220	.	.	.	.
REG00014	HIGD1A	GSE92220	.	.	.	.
REG00014	ZC3H15	GSE92220	.	.	.	.
REG00014	TBL1XR1	GSE92220	.	.	.	.
REG00014	KIFC2	GSE92220	.	.	.	.
REG00014	LARP1	GSE92220	.	.	.	.
REG00014	NAA50	GSE92220	.	.	.	.
REG00014	ESYT1	GSE92220	.	.	.	.
REG00014	MAPKBP1	GSE92220	.	.	.	.
REG00014	NOB1	GSE92220	.	.	.	.
REG00014	SUDS3	GSE92220	.	.	.	.
REG00014	SH3YL1	GSE92220	.	.	.	.
REG00014	SHCBP1	GSE92220	.	.	.	.
REG00014	RIC8A	GSE92220	.	.	.	.
REG00014	TBRG1	GSE92220	.	.	.	.
REG00014	UBE2O	GSE92220	.	.	.	.
REG00014	EFHD1	GSE92220	.	.	.	.
REG00014	NEXN	GSE92220	.	.	.	.
REG00014	VMP1	GSE92220	.	.	.	.
REG00014	DNASE1	GSE92220	.	.	.	.
REG00014	THYN1	GSE92220	.	.	.	.

REG00014	KIAA0430	GSE92220	.	.	.	.
REG00014	CCDC92	GSE92220	.	.	.	.
REG00014	C16orf70	GSE92220	.	.	.	.
REG00014	DOLPP1	GSE92220	.	.	.	.
REG00014	PLPPR2	GSE92220	.	.	.	.
REG00014	LHFPL4	GSE92220	.	.	.	.
REG00014	DNASE1L1	GSE92220	.	.	.	.
REG00014	LEAP2	GSE92220	.	.	.	.
REG00014	UBALD1	GSE92220	.	.	.	.
REG00014	TMEM170A	GSE92220	.	.	.	.
REG00014	SPATA18	GSE92220	.	.	.	.
REG00014	DNASE1L2	GSE92220	.	.	.	.
REG00014	MACROD1	GSE92220	.	.	.	.
REG00014	MED19	GSE92220	.	.	.	.
REG00014	C17orf80	GSE92220	.	.	.	.
REG00014	HSFX1	GSE92220	.	.	.	.
REG00014	SH2B3	GSE92220	.	.	.	.
REG00014	MTDH	GSE92220	.	.	.	.
REG00014	ACP6	GSE92220	.	.	.	.
REG00014	DYNC1H1	GSE92220	.	.	.	.
REG00014	CNTROB	GSE92220	.	.	.	.
REG00014	MAN2B2	GSE92220	.	.	.	.
REG00014	BUD31	GSE92220	.	.	.	.
REG00014	TMEM205	GSE92220	.	.	.	.
REG00014	MAGEF1	GSE92220	.	.	.	.
REG00014	DYNC1I2	GSE92220	.	.	.	.
REG00014	MUM1	GSE92220	.	.	.	.
REG00014	GDE1	GSE92220	.	.	.	.
REG00014	PAQR5	GSE92220	.	.	.	.
REG00014	FAM57A	GSE92220	.	.	.	.
REG00014	MAGI3	GSE92220	.	.	.	.
REG00014	WDR77	GSE92220	.	.	.	.
REG00014	ZNF771	GSE92220	.	.	.	.
REG00014	MESP2	GSE92220	.	.	.	.
REG00014	DYNC1LI2	GSE92220	.	.	.	.
REG00014	MSMP	GSE92220	.	.	.	.
REG00014	GNPTAB	GSE92220	.	.	.	.
REG00014	MICALL2	GSE92220	.	.	.	.
REG00014	CLCC1	GSE92220	.	.	.	.
REG00014	PUM3	GSE92220	.	.	.	.
REG00014	PAM16	GSE92220	.	.	.	.
REG00014	SLC25A33	GSE92220	.	.	.	.
REG00014	GFM2	GSE92220	.	.	.	.
REG00014	IARS2	GSE92220	.	.	.	.
REG00014	DNM1L	GSE92220	.	.	.	.
REG00014	DNM2	GSE92220	.	.	.	.
REG00014	DNMT1	GSE92220	.	.	.	.
REG00014	DNMT3A	GSE92220	.	.	.	.
REG00014	SLC25A37	GSE92220	.	.	.	.
REG00014	TOP1MT	GSE92220	.	.	.	.

REG00014	MTUS1	GSE92220	.	.	.	.
REG00014	MOB3C	GSE92220	.	.	.	.
REG00014	MICALL1	GSE92220	.	.	.	.
REG00014	NFKBIZ	GSE92220	.	.	.	.
REG00014	DNPEP	GSE92220	.	.	.	.
REG00014	MPHOSPH8	GSE92220	.	.	.	.
REG00014	NRN1L	GSE92220	.	.	.	.
REG00014	SAP130	GSE92220	.	.	.	.
REG00014	MUS81	GSE92220	.	.	.	.
REG00014	ISYNA1	GSE92220	.	.	.	.
REG00014	MYL6B	GSE92220	.	.	.	.
REG00014	MYL12B	GSE92220	.	.	.	.
REG00014	CDC42BPG	GSE92220	.	.	.	.
REG00014	NAT10	GSE92220	.	.	.	.
REG00014	NADK	GSE92220	.	.	.	.
REG00014	NSMF	GSE92220	.	.	.	.
REG00014	SOCS7	GSE92220	.	.	.	.
REG00014	DOC2A	GSE92220	.	.	.	.
REG00014	N4BP1	GSE92220	.	.	.	.
REG00014	N4BP2	GSE92220	.	.	.	.
REG00014	FAM192A	GSE92220	.	.	.	.
REG00014	AKAP8L	GSE92220	.	.	.	.
REG00014	NLK	GSE92220	.	.	.	.
REG00014	CHMP3	GSE92220	.	.	.	.
REG00014	SHC2	GSE92220	.	.	.	.
REG00014	DOCK1	GSE92220	.	.	.	.
REG00014	THOC7	GSE92220	.	.	.	.
REG00014	C1orf43	GSE92220	.	.	.	.
REG00014	UBAP2L	GSE92220	.	.	.	.
REG00014	NIT2	GSE92220	.	.	.	.
REG00014	ISCU	GSE92220	.	.	.	.
REG00014	UBASH3B	GSE92220	.	.	.	.
REG00014	CSDE1	GSE92220	.	.	.	.
REG00014	NTAN1	GSE92220	.	.	.	.
REG00014	CALCOCO2	GSE92220	.	.	.	.
REG00014	NUP210L	GSE92220	.	.	.	.
REG00014	NARF	GSE92220	.	.	.	.
REG00014	NDUFAF3	GSE92220	.	.	.	.
REG00014	DXO	GSE92220	.	.	.	.
REG00014	NUCKS1	GSE92220	.	.	.	.
REG00014	NOP58	GSE92220	.	.	.	.
REG00014	DONSON	GSE92220	.	.	.	.
REG00014	COASY	GSE92220	.	.	.	.
REG00014	NLRC5	GSE92220	.	.	.	.
REG00014	CACTIN	GSE92220	.	.	.	.
REG00014	GATAD1	GSE92220	.	.	.	.
REG00014	TRAK1	GSE92220	.	.	.	.
REG00014	DPAGT1	GSE92220	.	.	.	.
REG00014	OIT3	GSE92220	.	.	.	.
REG00014	SLC51A	GSE92220	.	.	.	.

REG00014	TTC33	GSE92220	.	.	.	.
REG00014	OSCP1	GSE92220	.	.	.	.
REG00014	QRFP	GSE92220	.	.	.	.
REG00014	ZMAT3	GSE92220	.	.	.	.
REG00014	TP53AIP1	GSE92220	.	.	.	.
REG00014	GATAD2A	GSE92220	.	.	.	.
REG00014	NUPR1	GSE92220	.	.	.	.
REG00014	PAN3	GSE92220	.	.	.	.
REG00014	A3GALT2	GSE92220	.	.	.	.
REG00014	PEF1	GSE92220	.	.	.	.
REG00014	PDF	GSE92220	.	.	.	.
REG00014	DPEP1	GSE92220	.	.	.	.
REG00014	HELZ2	GSE92220	.	.	.	.
REG00014	PCNP	GSE92220	.	.	.	.
REG00014	PEX5L	GSE92220	.	.	.	.
REG00014	PPRC1	GSE92220	.	.	.	.
REG00014	PLEKHO2	GSE92220	.	.	.	.
REG00014	JADE1	GSE92220	.	.	.	.
REG00014	PGS1	GSE92220	.	.	.	.
REG00014	PACS1	GSE92220	.	.	.	.
REG00014	PHPT1	GSE92220	.	.	.	.
REG00014	PLEKHA5	GSE92220	.	.	.	.
REG00014	PLEKHA8	GSE92220	.	.	.	.
REG00014	DPH2	GSE92220	.	.	.	.
REG00014	SMG1	GSE92220	.	.	.	.
REG00014	DPM1	GSE92220	.	.	.	.
REG00014	NUP210	GSE92220	.	.	.	.
REG00014	GPR171	GSE92220	.	.	.	.
REG00014	LPCAT4	GSE92220	.	.	.	.
REG00014	DPM2	GSE92220	.	.	.	.
REG00014	NOMO1	GSE92220	.	.	.	.
REG00014	PBRM1	GSE92220	.	.	.	.
REG00014	KIAA0319L	GSE92220	.	.	.	.
REG00014	POLR3A	GSE92220	.	.	.	.
REG00014	POLR3C	GSE92220	.	.	.	.
REG00014	REEP5	GSE92220	.	.	.	.
REG00014	REEP6	GSE92220	.	.	.	.
REG00014	DUS1L	GSE92220	.	.	.	.
REG00014	DPP4	GSE92220	.	.	.	.
REG00014	PRAM1	GSE92220	.	.	.	.
REG00014	NAMPT	GSE92220	.	.	.	.
REG00014	OSGIN1	GSE92220	.	.	.	.
REG00014	SF3B6	GSE92220	.	.	.	.
REG00014	PCF11	GSE92220	.	.	.	.
REG00014	CPSF7	GSE92220	.	.	.	.
REG00014	NPB	GSE92220	.	.	.	.
REG00014	PSENEN	GSE92220	.	.	.	.
REG00014	C16orf72	GSE92220	.	.	.	.
REG00014	ATAD2	GSE92220	.	.	.	.
REG00014	PAQR3	GSE92220	.	.	.	.

REG00014	PAQR9	GSE92220	.	.	.	.
REG00014	PAQR6	GSE92220	.	.	.	.
REG00014	DPYSL2	GSE92220	.	.	.	.
REG00014	TBKBP1	GSE92220	.	.	.	.
REG00014	ALKBH3	GSE92220	.	.	.	.
REG00014	GDF15	GSE92220	.	.	.	.
REG00014	PRMT3	GSE92220	.	.	.	.
REG00014	ZFAND6	GSE92220	.	.	.	.
REG00014	PDIA4	GSE92220	.	.	.	.
REG00014	PDIA6	GSE92220	.	.	.	.
REG00014	DR1	GSE92220	.	.	.	.
REG00014	STRADA	GSE92220	.	.	.	.
REG00014	SAC3D1	GSE92220	.	.	.	.
REG00014	CNFN	GSE92220	.	.	.	.
REG00014	TMEM167B	GSE92220	.	.	.	.
REG00014	GTPBP6	GSE92220	.	.	.	.
REG00014	DRAP1	GSE92220	.	.	.	.
REG00014	WDR83OS	GSE92220	.	.	.	.
REG00014	C11orf54	GSE92220	.	.	.	.
REG00014	AAMDC	GSE92220	.	.	.	.
REG00014	RNFT1	GSE92220	.	.	.	.
REG00014	ERGIC2	GSE92220	.	.	.	.
REG00014	ATP13A2	GSE92220	.	.	.	.
REG00014	CHMP2A	GSE92220	.	.	.	.
REG00014	DIMT1	GSE92220	.	.	.	.
REG00014	RFT1	GSE92220	.	.	.	.
REG00014	UBAC1	GSE92220	.	.	.	.
REG00014	SLC52A2	GSE92220	.	.	.	.
REG00014	SPPL2A	GSE92220	.	.	.	.
REG00014	PREPL	GSE92220	.	.	.	.
REG00014	SZRD1	GSE92220	.	.	.	.
REG00014	CYSTM1	GSE92220	.	.	.	.
REG00014	TEX264	GSE92220	.	.	.	.
REG00014	SMIM3	GSE92220	.	.	.	.
REG00014	QSOX2	GSE92220	.	.	.	.
REG00014	DRD4	GSE92220	.	.	.	.
REG00014	BAMBI	GSE92220	.	.	.	.
REG00014	PRELID1	GSE92220	.	.	.	.
REG00014	PYGO1	GSE92220	.	.	.	.
REG00014	PYGO2	GSE92220	.	.	.	.
REG00014	PYM1	GSE92220	.	.	.	.
REG00014	PDXP	GSE92220	.	.	.	.
REG00014	DRD5	GSE92220	.	.	.	.
REG00014	PNPO	GSE92220	.	.	.	.
REG00014	PYCR2	GSE92220	.	.	.	.
REG00014	PDP2	GSE92220	.	.	.	.
REG00014	RILP	GSE92220	.	.	.	.
REG00014	RAB11FIP4	GSE92220	.	.	.	.
REG00014	RALGPS2	GSE92220	.	.	.	.
REG00014	RGL3	GSE92220	.	.	.	.

REG00014	RUNDC3B	GSE92220	.	.	.	.
REG00014	RPTOR	GSE92220	.	.	.	.
REG00014	DTL	GSE92220	.	.	.	.
REG00014	DRG1	GSE92220	.	.	.	.
REG00014	RASL10B	GSE92220	.	.	.	.
REG00014	UIMC1	GSE92220	.	.	.	.
REG00014	LRIF1	GSE92220	.	.	.	.
REG00014	DRG2	GSE92220	.	.	.	.
REG00014	PHB2	GSE92220	.	.	.	.
REG00014	UBXN8	GSE92220	.	.	.	.
REG00014	RGMA	GSE92220	.	.	.	.
REG00014	RER1	GSE92220	.	.	.	.
REG00014	ARID3A	GSE92220	.	.	.	.
REG00014	UBR4	GSE92220	.	.	.	.
REG00014	FAM208A	GSE92220	.	.	.	.
REG00014	ARHGEF28	GSE92220	.	.	.	.
REG00014	RPP14	GSE92220	.	.	.	.
REG00014	ATN1	GSE92220	.	.	.	.
REG00014	DNAJC13	GSE92220	.	.	.	.
REG00014	POLR3E	GSE92220	.	.	.	.
REG00014	POLR3B	GSE92220	.	.	.	.
REG00014	POLR3H	GSE92220	.	.	.	.
REG00014	SBSPON	GSE92220	.	.	.	.
REG00014	ENOSF1	GSE92220	.	.	.	.
REG00014	DSC3	GSE92220	.	.	.	.
REG00014	DNMBP	GSE92220	.	.	.	.
REG00014	SHISA5	GSE92220	.	.	.	.
REG00014	SCRIB	GSE92220	.	.	.	.
REG00014	EXOC3	GSE92220	.	.	.	.
REG00014	SEH1L	GSE92220	.	.	.	.
REG00014	VMO1	GSE92220	.	.	.	.
REG00014	CLEC18A	GSE92220	.	.	.	.
REG00014	IFT172	GSE92220	.	.	.	.
REG00014	SELENOO	GSE92220	.	.	.	.
REG00014	SELENOS	GSE92220	.	.	.	.
REG00014	LEO1	GSE92220	.	.	.	.
REG00014	SENP7	GSE92220	.	.	.	.
REG00014	SCAF1	GSE92220	.	.	.	.
REG00014	HTRA3	GSE92220	.	.	.	.
REG00014	TSSK6	GSE92220	.	.	.	.
REG00014	TPRA1	GSE92220	.	.	.	.
REG00014	TMEM147	GSE92220	.	.	.	.
REG00014	SH2B1	GSE92220	.	.	.	.
REG00014	SH3D19	GSE92220	.	.	.	.
REG00014	HSD11B1L	GSE92220	.	.	.	.
REG00014	RCAN3	GSE92220	.	.	.	.
REG00014	CACYBP	GSE92220	.	.	.	.
REG00014	SPPL3	GSE92220	.	.	.	.
REG00014	PSMG1	GSE92220	.	.	.	.
REG00014	STAP2	GSE92220	.	.	.	.

REG00014	MYBPHL	GSE92220	.	.	.	.
REG00014	PLD6	GSE92220	.	.	.	.
REG00014	PTAR1	GSE92220	.	.	.	.
REG00014	NAPRT	GSE92220	.	.	.	.
REG00014	RCCD1	GSE92220	.	.	.	.
REG00014	UBR3	GSE92220	.	.	.	.
REG00014	METTL21A	GSE92220	.	.	.	.
REG00014	FAM171A2	GSE92220	.	.	.	.
REG00014	NXNL2	GSE92220	.	.	.	.
REG00014	PCMTD1	GSE92220	.	.	.	.
REG00014	DSG2	GSE92220	.	.	.	.
REG00014	FAM122B	GSE92220	.	.	.	.
REG00014	USF3	GSE92220	.	.	.	.
REG00014	KIF7	GSE92220	.	.	.	.
REG00014	PHLDB3	GSE92220	.	.	.	.
REG00014	PRRT2	GSE92220	.	.	.	.
REG00014	DSP	GSE92220	.	.	.	.
REG00014	SLC46A1	GSE92220	.	.	.	.
REG00014	MARVELD3	GSE92220	.	.	.	.
REG00014	DNAJC19	GSE92220	.	.	.	.
REG00014	NUDCD2	GSE92220	.	.	.	.
REG00014	CFAP36	GSE92220	.	.	.	.
REG00014	TMEM41A	GSE92220	.	.	.	.
REG00014	CDKN2AIPNL	GSE92220	.	.	.	.
REG00014	FDX1L	GSE92220	.	.	.	.
REG00014	BTF3L4	GSE92220	.	.	.	.
REG00014	MARCHF6	GSE92220	.	.	.	.
REG00014	TXNL4A	GSE92220	.	.	.	.
REG00014	RSRC2	GSE92220	.	.	.	.
REG00014	PARS2	GSE92220	.	.	.	.
REG00014	UBFD1	GSE92220	.	.	.	.
REG00014	DTNA	GSE92220	.	.	.	.
REG00014	ZNF627	GSE92220	.	.	.	.
REG00014	SIGIRR	GSE92220	.	.	.	.
REG00014	ADI1	GSE92220	.	.	.	.
REG00014	MIB2	GSE92220	.	.	.	.
REG00014	SSH1	GSE92220	.	.	.	.
REG00014	DTNB	GSE92220	.	.	.	.
REG00014	SSH3	GSE92220	.	.	.	.
REG00014	HSPB9	GSE92220	.	.	.	.
REG00014	TMEM50A	GSE92220	.	.	.	.
REG00014	UNC45A	GSE92220	.	.	.	.
REG00014	PPP1R21	GSE92220	.	.	.	.
REG00014	PIGZ	GSE92220	.	.	.	.
REG00014	DTYMK	GSE92220	.	.	.	.
REG00014	STT3B	GSE92220	.	.	.	.
REG00014	SPDYA	GSE92220	.	.	.	.
REG00014	ELP5	GSE92220	.	.	.	.
REG00014	SPNS1	GSE92220	.	.	.	.
REG00014	SPIRE2	GSE92220	.	.	.	.

REG00014	YTHDC1	GSE92220	.	.	.	.
REG00014	SPPL2B	GSE92220	.	.	.	.
REG00014	SPSB1	GSE92220	.	.	.	.
REG00014	SPSB3	GSE92220	.	.	.	.
REG00014	SCAP	GSE92220	.	.	.	.
REG00014	TSACC	GSE92220	.	.	.	.
REG00014	DUSP1	GSE92220	.	.	.	.
REG00014	SND1	GSE92220	.	.	.	.
REG00014	STRA8	GSE92220	.	.	.	.
REG00014	SLC50A1	GSE92220	.	.	.	.
REG00014	SAE1	GSE92220	.	.	.	.
REG00014	UBA2	GSE92220	.	.	.	.
REG00014	VHLL	GSE92220	.	.	.	.
REG00014	SUSD2	GSE92220	.	.	.	.
REG00014	SNAP47	GSE92220	.	.	.	.
REG00014	SYNPO	GSE92220	.	.	.	.
REG00014	TAB3	GSE92220	.	.	.	.
REG00014	TAPBPL	GSE92220	.	.	.	.
REG00014	TAX1BP3	GSE92220	.	.	.	.
REG00014	TXLNA	GSE92220	.	.	.	.
REG00014	CAND1	GSE92220	.	.	.	.
REG00014	DUSP3	GSE92220	.	.	.	.
REG00014	SP9	GSE92220	.	.	.	.
REG00014	LRRC8B	GSE92220	.	.	.	.
REG00014	WDR36	GSE92220	.	.	.	.
REG00014	ITFG1	GSE92220	.	.	.	.
REG00014	AES	GSE92220	.	.	.	.
REG00014	FAM155B	GSE92220	.	.	.	.
REG00014	FAM60A	GSE92220	.	.	.	.
REG00014	FAM71F1	GSE92220	.	.	.	.
REG00014	TEX261	GSE92220	.	.	.	.
REG00014	ADAD1	GSE92220	.	.	.	.
REG00014	DUSP6	GSE92220	.	.	.	.
REG00014	CCDC62	GSE92220	.	.	.	.
REG00014	CFAP70	GSE92220	.	.	.	.
REG00014	GTF2A1L	GSE92220	.	.	.	.
REG00014	DUSP7	GSE92220	.	.	.	.
REG00014	TARS2	GSE92220	.	.	.	.
REG00014	COPS2	GSE92220	.	.	.	.
REG00014	PAPD5	GSE92220	.	.	.	.
REG00014	DUSP9	GSE92220	.	.	.	.
REG00014	TMEM165	GSE92220	.	.	.	.
REG00014	TTC21A	GSE92220	.	.	.	.
REG00014	NR2C2AP	GSE92220	.	.	.	.
REG00014	TNIK	GSE92220	.	.	.	.
REG00014	KNSTRN	GSE92220	.	.	.	.
REG00014	NATD1	GSE92220	.	.	.	.
REG00014	TFAP2E	GSE92220	.	.	.	.
REG00014	CDCA7L	GSE92220	.	.	.	.
REG00014	GATAD2B	GSE92220	.	.	.	.

REG00014	MTERF2	GSE92220	.	.	.	.
REG00014	NAA15	GSE92220	.	.	.	.
REG00014	SERTAD2	GSE92220	.	.	.	.
REG00014	UBIAD1	GSE92220	.	.	.	.
REG00014	EIF5B	GSE92220	.	.	.	.
REG00014	CEP57	GSE92220	.	.	.	.
REG00014	STRAP	GSE92220	.	.	.	.
REG00014	TM9SF4	GSE92220	.	.	.	.
REG00014	PNPLA2	GSE92220	.	.	.	.
REG00014	ZBED5	GSE92220	.	.	.	.
REG00014	ZBED8	GSE92220	.	.	.	.
REG00014	TRNAU1AP	GSE92220	.	.	.	.
REG00014	MRNIP	GSE92220	.	.	.	.
REG00014	NT5DC3	GSE92220	.	.	.	.
REG00014	TDRD7	GSE92220	.	.	.	.
REG00014	TRAPPC9	GSE92220	.	.	.	.
REG00014	CDK2AP2	GSE92220	.	.	.	.
REG00014	POC1B	GSE92220	.	.	.	.
REG00014	DVL1	GSE92220	.	.	.	.
REG00014	CD302	GSE92220	.	.	.	.
REG00014	SEZ6L2	GSE92220	.	.	.	.
REG00014	SNRNP35	GSE92220	.	.	.	.
REG00014	U2SURP	GSE92220	.	.	.	.
REG00014	EFTUD2	GSE92220	.	.	.	.
REG00014	SNRNP200	GSE92220	.	.	.	.
REG00014	DVL2	GSE92220	.	.	.	.
REG00014	LSM11	GSE92220	.	.	.	.
REG00014	UQCR11	GSE92220	.	.	.	.
REG00014	UQCR10	GSE92220	.	.	.	.
REG00014	ZC3H7B	GSE92220	.	.	.	.
REG00014	DVL3	GSE92220	.	.	.	.
REG00014	ITFG2	GSE92220	.	.	.	.
REG00014	USE1	GSE92220	.	.	.	.
REG00014	TEX2	GSE92220	.	.	.	.
REG00014	TRAPPC2L	GSE92220	.	.	.	.
REG00014	HUWE1	GSE92220	.	.	.	.
REG00014	VCPIP1	GSE92220	.	.	.	.
REG00014	AVPI1	GSE92220	.	.	.	.
REG00014	ZNF655	GSE92220	.	.	.	.
REG00014	USO1	GSE92220	.	.	.	.
REG00014	VILL	GSE92220	.	.	.	.
REG00014	SORBS3	GSE92220	.	.	.	.
REG00014	DYRK1A	GSE92220	.	.	.	.
REG00014	VWA1	GSE92220	.	.	.	.
REG00014	DCAF1	GSE92220	.	.	.	.
REG00014	WIZ	GSE92220	.	.	.	.
REG00014	DYRK1B	GSE92220	.	.	.	.
REG00014	DYRK2	GSE92220	.	.	.	.
REG00014	YY1AP1	GSE92220	.	.	.	.
REG00014	ZFHX4	GSE92220	.	.	.	.

REG00014	ZC3H8	GSE92220	.	.	.	.
REG00014	ZNF593	GSE92220	.	.	.	.
REG00014	ZNF600	GSE92220	.	.	.	.
REG00014	ZBTB10	GSE92220	.	.	.	.
REG00014	ZNF587	GSE92220	.	.	.	.
REG00014	ZC3H7A	GSE92220	.	.	.	.
REG00014	DYSF	GSE92220	.	.	.	.
REG00014	SECISBP2	GSE92220	.	.	.	.
REG00014	TOR1A	GSE92220	.	.	.	.
REG00014	IFT20	GSE92220	.	.	.	.
REG00014	C9orf69	GSE92220	.	.	.	.
REG00014	SLC16A13	GSE92220	.	.	.	.
REG00014	SLC41A3	GSE92220	.	.	.	.
REG00014	TMEM132A	GSE92220	.	.	.	.
REG00014	MYO18A	GSE92220	.	.	.	.
REG00014	ZNF564	GSE92220	.	.	.	.
REG00014	CTXN1	GSE92220	.	.	.	.
REG00014	E2F1	GSE92220	.	.	.	.
REG00014	CCDC58	GSE92220	.	.	.	.
REG00014	E2F3	GSE92220	.	.	.	.
REG00014	E2F4	GSE92220	.	.	.	.
REG00014	E2F5	GSE92220	.	.	.	.
REG00014	E2F6	GSE92220	.	.	.	.
REG00014	E4F1	GSE92220	.	.	.	.
REG00014	OR6C76	GSE92220	.	.	.	.
REG00014	ATXN2L	GSE92220	.	.	.	.
REG00014	EBP	GSE92220	.	.	.	.
REG00014	RAB12	GSE92220	.	.	.	.
REG00014	IDNK	GSE92220	.	.	.	.
REG00014	TOMM5	GSE92220	.	.	.	.
REG00014	GPR179	GSE92220	.	.	.	.
REG00014	SWI5	GSE92220	.	.	.	.
REG00014	C9orf131	GSE92220	.	.	.	.
REG00014	FAM102A	GSE92220	.	.	.	.
REG00014	TOPORS-AS1	GSE92220	.	.	.	.
REG00014	ZBTB34	GSE92220	.	.	.	.
REG00014	LURAP1L	GSE92220	.	.	.	.
REG00014	C9orf152	GSE92220	.	.	.	.
REG00014	ECE1	GSE92220	.	.	.	.
REG00014	ECH1	GSE92220	.	.	.	.
REG00014	AFG3L2	GSE92220	.	.	.	.
REG00014	ECT2	GSE92220	.	.	.	.
REG00014	EDA	GSE92220	.	.	.	.
REG00014	EDF1	GSE92220	.	.	.	.
REG00014	ACSM6	GSE92220	.	.	.	.
REG00014	YTHDF2	GSE92220	.	.	.	.
REG00014	ANO7	GSE92220	.	.	.	.
REG00014	PRR5	GSE92220	.	.	.	.
REG00014	YY2	GSE92220	.	.	.	.
REG00014	MAP3K15	GSE92220	.	.	.	.

REG00014	RNF165	GSE92220	.	.	.	.
REG00014	AFP	GSE92220	.	.	.	.
REG00014	TMEM241	GSE92220	.	.	.	.
REG00014	PLAC8L1	GSE92220	.	.	.	.
REG00014	EDN1	GSE92220	.	.	.	.
REG00014	BOD1L1	GSE92220	.	.	.	.
REG00014	FAM45A	GSE92220	.	.	.	.
REG00014	NSUN4	GSE92220	.	.	.	.
REG00014	ARHGAP27	GSE92220	.	.	.	.
REG00014	PHC1	GSE92220	.	.	.	.
REG00014	PHC2	GSE92220	.	.	.	.
REG00014	MT1HL1	GSE92220	.	.	.	.
REG00014	EEF1A1	GSE92220	.	.	.	.
REG00014	RGL4	GSE92220	.	.	.	.
REG00014	ROPN1B	GSE92220	.	.	.	.
REG00014	CEACAM16	GSE92220	.	.	.	.
REG00014	CEACAM19	GSE92220	.	.	.	.
REG00014	GOLGA8A	GSE92220	.	.	.	.
REG00014	GOLGA8B	GSE92220	.	.	.	.
REG00014	AGER	GSE92220	.	.	.	.
REG00014	TMEM120B	GSE92220	.	.	.	.
REG00014	LDLRAD1	GSE92220	.	.	.	.
REG00014	LDLRAD2	GSE92220	.	.	.	.
REG00014	EEF1B2	GSE92220	.	.	.	.
REG00014	EEF1D	GSE92220	.	.	.	.
REG00014	EEF1E1	GSE92220	.	.	.	.
REG00014	EEF1G	GSE92220	.	.	.	.
REG00014	EEF2	GSE92220	.	.	.	.
REG00014	MTG1	GSE92220	.	.	.	.
REG00014	PVRIG	GSE92220	.	.	.	.
REG00014	LIN28B	GSE92220	.	.	.	.
REG00014	EFNA1	GSE92220	.	.	.	.
REG00014	EFNA2	GSE92220	.	.	.	.
REG00014	EEF2KMT	GSE92220	.	.	.	.
REG00014	WIPI2	GSE92220	.	.	.	.
REG00014	C8orf59	GSE92220	.	.	.	.
REG00014	EFNA4	GSE92220	.	.	.	.
REG00014	EFNB1	GSE92220	.	.	.	.
REG00014	EFNB2	GSE92220	.	.	.	.
REG00014	EGF	GSE92220	.	.	.	.
REG00014	ZNF704	GSE92220	.	.	.	.
REG00014	C1orf189	GSE92220	.	.	.	.
REG00014	FAM132A	GSE92220	.	.	.	.
REG00014	CELSR2	GSE92220	.	.	.	.
REG00014	RBM12B	GSE92220	.	.	.	.
REG00014	SCX	GSE92220	.	.	.	.
REG00014	CFAP126	GSE92220	.	.	.	.
REG00014	MEGF8	GSE92220	.	.	.	.
REG00014	MEGF9	GSE92220	.	.	.	.
REG00014	TMEM81	GSE92220	.	.	.	.

REG00014	CHAC2	GSE92220	.	.	.	.
REG00014	TMEM88	GSE92220	.	.	.	.
REG00014	TMEM89	GSE92220	.	.	.	.
REG00014	EGR1	GSE92220	.	.	.	.
REG00014	TMEM91	GSE92220	.	.	.	.
REG00014	SMTNL1	GSE92220	.	.	.	.
REG00014	DGKK	GSE92220	.	.	.	.
REG00014	SCART1	GSE92220	.	.	.	.
REG00014	EHD1	GSE92220	.	.	.	.
REG00014	RPRML	GSE92220	.	.	.	.
REG00014	SPEM1	GSE92220	.	.	.	.
REG00014	EHD2	GSE92220	.	.	.	.
REG00014	SLC38A8	GSE92220	.	.	.	.
REG00014	LRTM2	GSE92220	.	.	.	.
REG00014	OST4	GSE92220	.	.	.	.
REG00014	ALKBH2	GSE92220	.	.	.	.
REG00014	EIF1	GSE92220	.	.	.	.
REG00014	EIF1AX	GSE92220	.	.	.	.
REG00014	ARSI	GSE92220	.	.	.	.
REG00014	ANKDD1B	GSE92220	.	.	.	.
REG00014	PRCD	GSE92220	.	.	.	.
REG00014	EIF2A	GSE92220	.	.	.	.
REG00014	EIF2AK3	GSE92220	.	.	.	.
REG00014	CEMP1	GSE92220	.	.	.	.
REG00014	FAM83G	GSE92220	.	.	.	.
REG00014	DCDC2B	GSE92220	.	.	.	.
REG00014	EIF2B2	GSE92220	.	.	.	.
REG00014	AGPAT3	GSE92220	.	.	.	.
REG00014	EIF2B4	GSE92220	.	.	.	.
REG00014	EIF2B5	GSE92220	.	.	.	.
REG00014	AGO1	GSE92220	.	.	.	.
REG00014	AGO2	GSE92220	.	.	.	.
REG00014	EIF2S1	GSE92220	.	.	.	.
REG00014	EIF2S2	GSE92220	.	.	.	.
REG00014	TRIM71	GSE92220	.	.	.	.
REG00014	EIF2S3	GSE92220	.	.	.	.
REG00014	WDR83	GSE92220	.	.	.	.
REG00014	MED11	GSE92220	.	.	.	.
REG00014	AGPS	GSE92220	.	.	.	.
REG00014	EIF3J	GSE92220	.	.	.	.
REG00014	EIF3A	GSE92220	.	.	.	.
REG00014	EIF3H	GSE92220	.	.	.	.
REG00014	EIF3G	GSE92220	.	.	.	.
REG00014	EIF3F	GSE92220	.	.	.	.
REG00014	EIF3E	GSE92220	.	.	.	.
REG00014	EIF3D	GSE92220	.	.	.	.
REG00014	PNO1	GSE92220	.	.	.	.
REG00014	AGR2	GSE92220	.	.	.	.
REG00014	EIF3B	GSE92220	.	.	.	.
REG00014	EIF4A1	GSE92220	.	.	.	.

REG00014	EIF4A2	GSE92220	.	.	.	.
REG00014	EIF4B	GSE92220	.	.	.	.
REG00014	EIF4E	GSE92220	.	.	.	.
REG00014	EIF4EBP1	GSE92220	.	.	.	.
REG00014	EIF4EBP2	GSE92220	.	.	.	.
REG00014	AGRN	GSE92220	.	.	.	.
REG00014	EIF4EBP3	GSE92220	.	.	.	.
REG00014	EIF4E2	GSE92220	.	.	.	.
REG00014	CENPP	GSE92220	.	.	.	.
REG00014	SEBOX	GSE92220	.	.	.	.
REG00014	RNF207	GSE92220	.	.	.	.
REG00014	SMPD4	GSE92220	.	.	.	.
REG00014	EIF4G1	GSE92220	.	.	.	.
REG00014	EID3	GSE92220	.	.	.	.
REG00014	EIF4G2	GSE92220	.	.	.	.
REG00014	HAUS7	GSE92220	.	.	.	.
REG00014	EIF4G3	GSE92220	.	.	.	.
REG00014	EIF5	GSE92220	.	.	.	.
REG00014	EIF5A	GSE92220	.	.	.	.
REG00014	EIF5A2	GSE92220	.	.	.	.
REG00014	ZNF780B	GSE92220	.	.	.	.
REG00014	SERPINB1	GSE92220	.	.	.	.
REG00014	ELAVL1	GSE92220	.	.	.	.
REG00014	LRRC75B	GSE92220	.	.	.	.
REG00014	ELF1	GSE92220	.	.	.	.
REG00014	ELF2	GSE92220	.	.	.	.
REG00014	DBX1	GSE92220	.	.	.	.
REG00014	MZT2A	GSE92220	.	.	.	.
REG00014	ELF4	GSE92220	.	.	.	.
REG00014	PLIN5	GSE92220	.	.	.	.
REG00014	ELK1	GSE92220	.	.	.	.
REG00014	DUSP28	GSE92220	.	.	.	.
REG00014	ELK3	GSE92220	.	.	.	.
REG00014	ZNF814	GSE92220	.	.	.	.
REG00014	ELK4	GSE92220	.	.	.	.
REG00014	ZBED6	GSE92220	.	.	.	.
REG00014	ATXN1L	GSE92220	.	.	.	.
REG00014	AGT	GSE92220	.	.	.	.
REG00014	EMD	GSE92220	.	.	.	.
REG00014	MARK2	GSE92220	.	.	.	.
REG00014	EMP2	GSE92220	.	.	.	.
REG00014	CTTN	GSE92220	.	.	.	.
REG00014	EMX1	GSE92220	.	.	.	.
REG00014	RNF215	GSE92220	.	.	.	.
REG00014	CCDC137	GSE92220	.	.	.	.
REG00014	ENDOG	GSE92220	.	.	.	.
REG00014	EXOG	GSE92220	.	.	.	.
REG00014	MEX3A	GSE92220	.	.	.	.
REG00014	C15orf52	GSE92220	.	.	.	.
REG00014	CT83	GSE92220	.	.	.	.

REG00014	ENO1	GSE92220	.	.	.	.
REG00014	ENO2	GSE92220	.	.	.	.
REG00014	ENO3	GSE92220	.	.	.	.
REG00014	FNDC9	GSE92220	.	.	.	.
REG00014	NDUFAF8	GSE92220	.	.	.	.
REG00014	ENPP1	GSE92220	.	.	.	.
REG00014	ENPP2	GSE92220	.	.	.	.
REG00014	ENPP3	GSE92220	.	.	.	.
REG00014	ENSA	GSE92220	.	.	.	.
REG00014	TMEM191B	GSE92220	.	.	.	.
REG00014	TMEM191C	GSE92220	.	.	.	.
REG00014	CPSF4L	GSE92220	.	.	.	.
REG00014	ENTPD2	GSE92220	.	.	.	.
REG00014	ENTPD5	GSE92220	.	.	.	.
REG00014	ENTPD6	GSE92220	.	.	.	.
REG00014	TTC36	GSE92220	.	.	.	.
REG00014	CDPF1	GSE92220	.	.	.	.
REG00014	CXorf65	GSE92220	.	.	.	.
REG00014	FITM1	GSE92220	.	.	.	.
REG00014	METTL21C	GSE92220	.	.	.	.
REG00014	TMEM201	GSE92220	.	.	.	.
REG00014	PRR19	GSE92220	.	.	.	.
REG00014	EP300	GSE92220	.	.	.	.
REG00014	C3orf70	GSE92220	.	.	.	.
REG00014	NCCRP1	GSE92220	.	.	.	.
REG00014	EPAS1	GSE92220	.	.	.	.
REG00014	MSANTD1	GSE92220	.	.	.	.
REG00014	REP15	GSE92220	.	.	.	.
REG00014	NHLRC3	GSE92220	.	.	.	.
REG00014	TOMM20L	GSE92220	.	.	.	.
REG00014	C16orf86	GSE92220	.	.	.	.
REG00014	C2orf82	GSE92220	.	.	.	.
REG00014	EPB41	GSE92220	.	.	.	.
REG00014	LAMTOR4	GSE92220	.	.	.	.
REG00014	FAM150A	GSE92220	.	.	.	.
REG00014	EPB41L1	GSE92220	.	.	.	.
REG00014	PTRHD1	GSE92220	.	.	.	.
REG00014	TMEM200B	GSE92220	.	.	.	.
REG00014	EPB41L2	GSE92220	.	.	.	.
REG00014	C12orf76	GSE92220	.	.	.	.
REG00014	C17orf97	GSE92220	.	.	.	.
REG00014	C2orf66	GSE92220	.	.	.	.
REG00014	DMTN	GSE92220	.	.	.	.
REG00014	UFSP1	GSE92220	.	.	.	.
REG00014	ERVFRD-1	GSE92220	.	.	.	.
REG00014	C8orf82	GSE92220	.	.	.	.
REG00014	STOM	GSE92220	.	.	.	.
REG00014	COA5	GSE92220	.	.	.	.
REG00014	CLEC18B	GSE92220	.	.	.	.
REG00014	CCDC157	GSE92220	.	.	.	.

REG00014	FAM196A	GSE92220	.	.	.	.
REG00014	TMPPE	GSE92220	.	.	.	.
REG00014	CCNI2	GSE92220	.	.	.	.
REG00014	DNLZ	GSE92220	.	.	.	.
REG00014	POTEE	GSE92220	.	.	.	.
REG00014	POTEF	GSE92220	.	.	.	.
REG00014	EPHA8	GSE92220	.	.	.	.
REG00014	RNASEK	GSE92220	.	.	.	.
REG00014	EPHB1	GSE92220	.	.	.	.
REG00014	NPS	GSE92220	.	.	.	.
REG00014	SLC35E2B	GSE92220	.	.	.	.
REG00014	EPHB4	GSE92220	.	.	.	.
REG00014	PPIAL4C	GSE92220	.	.	.	.
REG00014	ABCA4	GSE92220	.	.	.	.
REG00014	POM121C	GSE92220	.	.	.	.
REG00014	PLEKHM3	GSE92220	.	.	.	.
REG00014	EPHX1	GSE92220	.	.	.	.
REG00014	EPHX2	GSE92220	.	.	.	.
REG00014	STX2	GSE92220	.	.	.	.
REG00014	TMEM210	GSE92220	.	.	.	.
REG00014	AGXT	GSE92220	.	.	.	.
REG00014	EPOR	GSE92220	.	.	.	.
REG00014	EPRS	GSE92220	.	.	.	.
REG00014	EPS15	GSE92220	.	.	.	.
REG00014	EPS8	GSE92220	.	.	.	.
REG00014	FAM159B	GSE92220	.	.	.	.
REG00014	TMEM170B	GSE92220	.	.	.	.
REG00014	AHCY	GSE92220	.	.	.	.
REG00014	ERBB2	GSE92220	.	.	.	.
REG00014	ERBB3	GSE92220	.	.	.	.
REG00014	ERCC2	GSE92220	.	.	.	.
REG00014	TRNP1	GSE92220	.	.	.	.
REG00014	ERCC3	GSE92220	.	.	.	.
REG00014	C1orf226	GSE92220	.	.	.	.
REG00014	C2orf68	GSE92220	.	.	.	.
REG00014	C19orf67	GSE92220	.	.	.	.
REG00014	ERCC4	GSE92220	.	.	.	.
REG00014	FAM177B	GSE92220	.	.	.	.
REG00014	AHCYL1	GSE92220	.	.	.	.
REG00014	HEPN1	GSE92220	.	.	.	.
REG00014	HYKK	GSE92220	.	.	.	.
REG00014	PRSS53	GSE92220	.	.	.	.
REG00014	NEURL4	GSE92220	.	.	.	.
REG00014	FAM228A	GSE92220	.	.	.	.
REG00014	C6orf226	GSE92220	.	.	.	.
REG00014	ERF	GSE92220	.	.	.	.
REG00014	SMIM23	GSE92220	.	.	.	.
REG00014	C11orf91	GSE92220	.	.	.	.
REG00014	C12orf73	GSE92220	.	.	.	.
REG00014	FAM180B	GSE92220	.	.	.	.

REG00014	C15orf61	GSE92220	.	.	.	.
REG00014	CCDC154	GSE92220	.	.	.	.
REG00014	ERH	GSE92220	.	.	.	.
REG00014	C15orf62	GSE92220	.	.	.	.
REG00014	ERN1	GSE92220	.	.	.	.
REG00014	EPOP	GSE92220	.	.	.	.
REG00014	C19orf68	GSE92220	.	.	.	.
REG00014	C19orf71	GSE92220	.	.	.	.
REG00014	ZNF860	GSE92220	.	.	.	.
REG00014	VOPP1	GSE92220	.	.	.	.
REG00014	TOMM6	GSE92220	.	.	.	.
REG00014	EFCAB9	GSE92220	.	.	.	.
REG00014	EFCAB10	GSE92220	.	.	.	.
REG00014	ERV3-1	GSE92220	.	.	.	.
REG00014	MYADML2	GSE92220	.	.	.	.
REG00014	AHRR	GSE92220	.	.	.	.
REG00014	ESD	GSE92220	.	.	.	.
REG00014	AHNAK	GSE92220	.	.	.	.
REG00014	ESRRA	GSE92220	.	.	.	.
REG00014	ETF1	GSE92220	.	.	.	.
REG00014	AHR	GSE92220	.	.	.	.
REG00014	ETFA	GSE92220	.	.	.	.
REG00014	ETFB	GSE92220	.	.	.	.
REG00014	ETS2	GSE92220	.	.	.	.
REG00014	AHSG	GSE92220	.	.	.	.
REG00014	ETV1	GSE92220	.	.	.	.
REG00014	ETV4	GSE92220	.	.	.	.
REG00014	ETV5	GSE92220	.	.	.	.
REG00014	EVC	GSE92220	.	.	.	.
REG00014	EVI2A	GSE92220	.	.	.	.
REG00014	ABCA5	GSE92220	.	.	.	.
REG00014	EVI5	GSE92220	.	.	.	.
REG00014	EVPL	GSE92220	.	.	.	.
REG00014	EXT1	GSE92220	.	.	.	.
REG00014	EXTL3	GSE92220	.	.	.	.
REG00014	EZH2	GSE92220	.	.	.	.
REG00014	F10	GSE92220	.	.	.	.
REG00014	F11	GSE92220	.	.	.	.
REG00014	F2	GSE92220	.	.	.	.
REG00014	F2RL1	GSE92220	.	.	.	.
REG00014	F2RL3	GSE92220	.	.	.	.
REG00014	F3	GSE92220	.	.	.	.
REG00014	TSTD1	GSE92220	.	.	.	.
REG00014	EML6	GSE92220	.	.	.	.
REG00014	F5	GSE92220	.	.	.	.
REG00014	F7	GSE92220	.	.	.	.
REG00014	CCDC85C	GSE92220	.	.	.	.
REG00014	FAAH	GSE92220	.	.	.	.
REG00014	HSD17B8	GSE92220	.	.	.	.
REG00014	FABP1	GSE92220	.	.	.	.

REG00014	FABP3	GSE92220	.	.	.	.
REG00014	AIM1	GSE92220	.	.	.	.
REG00014	ACSL1	GSE92220	.	.	.	.
REG00014	ACSL3	GSE92220	.	.	.	.
REG00014	ACSL4	GSE92220	.	.	.	.
REG00014	FADD	GSE92220	.	.	.	.
REG00014	FADS2	GSE92220	.	.	.	.
REG00014	FADS3	GSE92220	.	.	.	.
REG00014	FAF1	GSE92220	.	.	.	.
REG00014	FAH	GSE92220	.	.	.	.
REG00014	BPTF	GSE92220	.	.	.	.
REG00014	FANCA	GSE92220	.	.	.	.
REG00014	FANCE	GSE92220	.	.	.	.
REG00014	FARP1	GSE92220	.	.	.	.
REG00014	FARSA	GSE92220	.	.	.	.
REG00014	FASN	GSE92220	.	.	.	.
REG00014	FAT1	GSE92220	.	.	.	.
REG00014	FBL	GSE92220	.	.	.	.
REG00014	FBLN1	GSE92220	.	.	.	.
REG00014	FBLN5	GSE92220	.	.	.	.
REG00014	AK1	GSE92220	.	.	.	.
REG00014	FCER1G	GSE92220	.	.	.	.
REG00014	AK2	GSE92220	.	.	.	.
REG00014	FCGRT	GSE92220	.	.	.	.
REG00014	FDFT1	GSE92220	.	.	.	.
REG00014	FDPS	GSE92220	.	.	.	.
REG00014	FDX1	GSE92220	.	.	.	.
REG00014	FDXR	GSE92220	.	.	.	.
REG00014	FECH	GSE92220	.	.	.	.
REG00014	FEM1B	GSE92220	.	.	.	.
REG00014	FEN1	GSE92220	.	.	.	.
REG00014	FES	GSE92220	.	.	.	.
REG00014	FEZ2	GSE92220	.	.	.	.
REG00014	FGA	GSE92220	.	.	.	.
REG00014	FGB	GSE92220	.	.	.	.
REG00014	AKAP1	GSE92220	.	.	.	.
REG00014	FGF18	GSE92220	.	.	.	.
REG00014	FGF22	GSE92220	.	.	.	.
REG00014	AKAP10	GSE92220	.	.	.	.
REG00014	FGF8	GSE92220	.	.	.	.
REG00014	FGFR1	GSE92220	.	.	.	.
REG00014	FGFR2	GSE92220	.	.	.	.
REG00014	AKAP11	GSE92220	.	.	.	.
REG00014	FGFR3	GSE92220	.	.	.	.
REG00014	FGFR4	GSE92220	.	.	.	.
REG00014	FGFRL1	GSE92220	.	.	.	.
REG00014	FGG	GSE92220	.	.	.	.
REG00014	FGL1	GSE92220	.	.	.	.
REG00014	ABCA7	GSE92220	.	.	.	.
REG00014	AKAP12	GSE92220	.	.	.	.

REG00014	FH	GSE92220	.	.	.	.
REG00014	FHIT	GSE92220	.	.	.	.
REG00014	FIBP	GSE92220	.	.	.	.
REG00014	GATSL2	GSE92220	.	.	.	.
REG00014	TMEM88B	GSE92220	.	.	.	.
REG00014	AKAP13	GSE92220	.	.	.	.
REG00014	ZAR1L	GSE92220	.	.	.	.
REG00014	FKBP1B	GSE92220	.	.	.	.
REG00014	FKBP3	GSE92220	.	.	.	.
REG00014	FKBP4	GSE92220	.	.	.	.
REG00014	MFSD2B	GSE92220	.	.	.	.
REG00014	C11orf94	GSE92220	.	.	.	.
REG00014	C17orf107	GSE92220	.	.	.	.
REG00014	FKBP8	GSE92220	.	.	.	.
REG00014	HSBP1L1	GSE92220	.	.	.	.
REG00014	SMIM24	GSE92220	.	.	.	.
REG00014	ZGLP1	GSE92220	.	.	.	.
REG00014	SRRM5	GSE92220	.	.	.	.
REG00014	FKBP9	GSE92220	.	.	.	.
REG00014	SMIM4	GSE92220	.	.	.	.
REG00014	SMIM20	GSE92220	.	.	.	.
REG00014	C4orf51	GSE92220	.	.	.	.
REG00014	LRRC14B	GSE92220	.	.	.	.
REG00014	CTAGE9	GSE92220	.	.	.	.
REG00014	C9orf172	GSE92220	.	.	.	.
REG00014	STPG3	GSE92220	.	.	.	.
REG00014	AKAP5	GSE92220	.	.	.	.
REG00014	FLII	GSE92220	.	.	.	.
REG00014	FLNA	GSE92220	.	.	.	.
REG00014	FLNB	GSE92220	.	.	.	.
REG00014	FLOT1	GSE92220	.	.	.	.
REG00014	FLOT2	GSE92220	.	.	.	.
REG00014	FLRT1	GSE92220	.	.	.	.
REG00014	FMO5	GSE92220	.	.	.	.
REG00014	FMR1	GSE92220	.	.	.	.
REG00014	FN1	GSE92220	.	.	.	.
REG00014	AKAP8	GSE92220	.	.	.	.
REG00014	FNTA	GSE92220	.	.	.	.
REG00014	ATXN7L3B	GSE92220	.	.	.	.
REG00014	FOSB	GSE92220	.	.	.	.
REG00014	FOSL2	GSE92220	.	.	.	.
REG00014	AKR1A1	GSE92220	.	.	.	.
REG00014	FOXC1	GSE92220	.	.	.	.
REG00014	FOXE3	GSE92220	.	.	.	.
REG00014	AKR1B1	GSE92220	.	.	.	.
REG00014	FOXO1	GSE92220	.	.	.	.
REG00014	AKR1B10	GSE92220	.	.	.	.
REG00014	FOXO3	GSE92220	.	.	.	.
REG00014	FOXP1	GSE92220	.	.	.	.
REG00014	AKR1C1	GSE92220	.	.	.	.

REG00014	AKR1C2	GSE92220	.	.	.	.
REG00014	AKR1C3	GSE92220	.	.	.	.
REG00014	ZNF865	GSE92220	.	.	.	.
REG00014	AKT1	GSE92220	.	.	.	.
REG00014	AKT2	GSE92220	.	.	.	.
REG00014	MTOR	GSE92220	.	.	.	.
REG00014	ALAD	GSE92220	.	.	.	.
REG00014	FXN	GSE92220	.	.	.	.
REG00014	NCS1	GSE92220	.	.	.	.
REG00014	FRZB	GSE92220	.	.	.	.
REG00014	ALAS1	GSE92220	.	.	.	.
REG00014	FSCN2	GSE92220	.	.	.	.
REG00014	FSCN3	GSE92220	.	.	.	.
REG00014	FST	GSE92220	.	.	.	.
REG00014	FTCD	GSE92220	.	.	.	.
REG00014	FTH1	GSE92220	.	.	.	.
REG00014	FTHL17	GSE92220	.	.	.	.
REG00014	ALB	GSE92220	.	.	.	.
REG00014	FTL	GSE92220	.	.	.	.
REG00014	ALCAM	GSE92220	.	.	.	.
REG00014	RNF223	GSE92220	.	.	.	.
REG00014	IFITM10	GSE92220	.	.	.	.
REG00014	MANSC4	GSE92220	.	.	.	.
REG00014	TEX22	GSE92220	.	.	.	.
REG00014	SMIM5	GSE92220	.	.	.	.
REG00014	SMIM6	GSE92220	.	.	.	.
REG00014	C16orf95	GSE92220	.	.	.	.
REG00014	FUBP1	GSE92220	.	.	.	.
REG00014	FUBP3	GSE92220	.	.	.	.
REG00014	AATK-AS1	GSE92220	.	.	.	.
REG00014	FUCA2	GSE92220	.	.	.	.
REG00014	FUS	GSE92220	.	.	.	.
REG00014	MED14OS	GSE92220	.	.	.	.
REG00014	FUT6	GSE92220	.	.	.	.
REG00014	ALDH1A1	GSE92220	.	.	.	.
REG00014	FXR1	GSE92220	.	.	.	.
REG00014	FXR2	GSE92220	.	.	.	.
REG00014	FXYD2	GSE92220	.	.	.	.
REG00014	ALDH3A2	GSE92220	.	.	.	.
REG00014	ALDH2	GSE92220	.	.	.	.
REG00014	FZD4	GSE92220	.	.	.	.
REG00014	FZD5	GSE92220	.	.	.	.
REG00014	FZD9	GSE92220	.	.	.	.
REG00014	XRCC6	GSE92220	.	.	.	.
REG00014	G6PC	GSE92220	.	.	.	.
REG00014	G6PD	GSE92220	.	.	.	.
REG00014	ALDH4A1	GSE92220	.	.	.	.
REG00014	SLC37A4	GSE92220	.	.	.	.
REG00014	GAA	GSE92220	.	.	.	.
REG00014	GAB1	GSE92220	.	.	.	.

REG00014	GABARAP	GSE92220	.	.	.	.
REG00014	GABARAPL1	GSE92220	.	.	.	.
REG00014	ALDH1B1	GSE92220	.	.	.	.
REG00014	GABPA	GSE92220	.	.	.	.
REG00014	GABPB1	GSE92220	.	.	.	.
REG00014	ALDH5A1	GSE92220	.	.	.	.
REG00014	GABRE	GSE92220	.	.	.	.
REG00014	GABRR2	GSE92220	.	.	.	.
REG00014	GADD45A	GSE92220	.	.	.	.
REG00014	ABCB10	GSE92220	.	.	.	.
REG00014	GAK	GSE92220	.	.	.	.
REG00014	GAL	GSE92220	.	.	.	.
REG00014	GALE	GSE92220	.	.	.	.
REG00014	GALK1	GSE92220	.	.	.	.
REG00014	GALK2	GSE92220	.	.	.	.
REG00014	GALNT1	GSE92220	.	.	.	.
REG00014	GALNT2	GSE92220	.	.	.	.
REG00014	GAMT	GSE92220	.	.	.	.
REG00014	GAN	GSE92220	.	.	.	.
REG00014	GANAB	GSE92220	.	.	.	.
REG00014	ALDOA	GSE92220	.	.	.	.
REG00014	GAPDH	GSE92220	.	.	.	.
REG00014	GARS	GSE92220	.	.	.	.
REG00014	GART	GSE92220	.	.	.	.
REG00014	GAS8	GSE92220	.	.	.	.
REG00014	GAS2	GSE92220	.	.	.	.
REG00014	GATA2	GSE92220	.	.	.	.
REG00014	GATA4	GSE92220	.	.	.	.
REG00014	GATA6	GSE92220	.	.	.	.
REG00014	GBA	GSE92220	.	.	.	.
REG00014	GBAS	GSE92220	.	.	.	.
REG00014	GBE1	GSE92220	.	.	.	.
REG00014	GBX2	GSE92220	.	.	.	.
REG00014	GCAT	GSE92220	.	.	.	.
REG00014	GCDH	GSE92220	.	.	.	.
REG00014	PROB1	GSE92220	.	.	.	.
REG00014	SAP25	GSE92220	.	.	.	.
REG00014	C7orf73	GSE92220	.	.	.	.
REG00014	GCKR	GSE92220	.	.	.	.
REG00014	NPIPA8	GSE92220	.	.	.	.
REG00014	NPIPB4	GSE92220	.	.	.	.
REG00014	GCN1	GSE92220	.	.	.	.
REG00014	KAT2A	GSE92220	.	.	.	.
REG00014	GCNT2	GSE92220	.	.	.	.
REG00014	GCSH	GSE92220	.	.	.	.
REG00014	GDF1	GSE92220	.	.	.	.
REG00014	GDF7	GSE92220	.	.	.	.
REG00014	GDI1	GSE92220	.	.	.	.
REG00014	GDI2	GSE92220	.	.	.	.
REG00014	GDNF	GSE92220	.	.	.	.

REG00014	GFER	GSE92220	.	.	.	.
REG00014	GFI1	GSE92220	.	.	.	.
REG00014	NCR3LG1	GSE92220	.	.	.	.
REG00014	GFPT1	GSE92220	.	.	.	.
REG00014	GGCX	GSE92220	.	.	.	.
REG00014	GGH	GSE92220	.	.	.	.
REG00014	GGPS1	GSE92220	.	.	.	.
REG00014	GGT1	GSE92220	.	.	.	.
REG00014	GGT5	GSE92220	.	.	.	.
REG00014	GHRH	GSE92220	.	.	.	.
REG00014	GHSR	GSE92220	.	.	.	.
REG00014	GIT1	GSE92220	.	.	.	.
REG00014	GIT2	GSE92220	.	.	.	.
REG00014	GJB1	GSE92220	.	.	.	.
REG00014	GK	GSE92220	.	.	.	.
REG00014	ALOX12	GSE92220	.	.	.	.
REG00014	ANKRD65	GSE92220	.	.	.	.
REG00014	FNDC10	GSE92220	.	.	.	.
REG00014	SMIM18	GSE92220	.	.	.	.
REG00014	TAP1	GSE92220	.	.	.	.
REG00014	ALOX12B	GSE92220	.	.	.	.
REG00014	GCLC	GSE92220	.	.	.	.
REG00014	GCLM	GSE92220	.	.	.	.
REG00014	GLDC	GSE92220	.	.	.	.
REG00014	GLE1	GSE92220	.	.	.	.
REG00014	GLG1	GSE92220	.	.	.	.
REG00014	GLI1	GSE92220	.	.	.	.
REG00014	GLI4	GSE92220	.	.	.	.
REG00014	GLO1	GSE92220	.	.	.	.
REG00014	ALOX15	GSE92220	.	.	.	.
REG00014	GLRX	GSE92220	.	.	.	.
REG00014	GLS	GSE92220	.	.	.	.
REG00014	GLTSCR1	GSE92220	.	.	.	.
REG00014	GLTSCR2	GSE92220	.	.	.	.
REG00014	GLUD1	GSE92220	.	.	.	.
REG00014	GLUL	GSE92220	.	.	.	.
REG00014	LINC00493	GSE92220	.	.	.	.
REG00014	GM2A	GSE92220	.	.	.	.
REG00014	GMDS	GSE92220	.	.	.	.
REG00014	GMFB	GSE92220	.	.	.	.
REG00014	GMPR	GSE92220	.	.	.	.
REG00014	GMPR2	GSE92220	.	.	.	.
REG00014	GMPS	GSE92220	.	.	.	.
REG00014	GNA11	GSE92220	.	.	.	.
REG00014	GNA12	GSE92220	.	.	.	.
REG00014	GNA13	GSE92220	.	.	.	.
REG00014	GNA14	GSE92220	.	.	.	.
REG00014	GNA15	GSE92220	.	.	.	.
REG00014	GNAI2	GSE92220	.	.	.	.
REG00014	ALPP	GSE92220	.	.	.	.

REG00014	GNAQ	GSE92220	.	.	.	.
REG00014	GNAS	GSE92220	.	.	.	.
REG00014	GNAT1	GSE92220	.	.	.	.
REG00014	GNAZ	GSE92220	.	.	.	.
REG00014	GNB1	GSE92220	.	.	.	.
REG00014	GNB1L	GSE92220	.	.	.	.
REG00014	GNB2	GSE92220	.	.	.	.
REG00014	RACK1	GSE92220	.	.	.	.
REG00014	TAP2	GSE92220	.	.	.	.
REG00014	GNB3	GSE92220	.	.	.	.
REG00014	GNB5	GSE92220	.	.	.	.
REG00014	GNG10	GSE92220	.	.	.	.
REG00014	GNG3	GSE92220	.	.	.	.
REG00014	GNG4	GSE92220	.	.	.	.
REG00014	SPTY2D1-AS1	GSE92220	.	.	.	.
REG00014	GNL1	GSE92220	.	.	.	.
REG00014	TGFBR3L	GSE92220	.	.	.	.
REG00014	TMEM249	GSE92220	.	.	.	.
REG00014	TPBGL	GSE92220	.	.	.	.
REG00014	GNPDA1	GSE92220	.	.	.	.
REG00014	GNRH1	GSE92220	.	.	.	.
REG00014	GNRH2	GSE92220	.	.	.	.
REG00014	ARL14EPL	GSE92220	.	.	.	.
REG00014	SMIM1	GSE92220	.	.	.	.
REG00014	MPC1L	GSE92220	.	.	.	.
REG00014	PINLYP	GSE92220	.	.	.	.
REG00014	GNS	GSE92220	.	.	.	.
REG00014	GOLGA3	GSE92220	.	.	.	.
REG00014	GOLGA4	GSE92220	.	.	.	.
REG00014	GOLGA5	GSE92220	.	.	.	.
REG00014	GOLGB1	GSE92220	.	.	.	.
REG00014	ALS2	GSE92220	.	.	.	.
REG00014	GOSR1	GSE92220	.	.	.	.
REG00014	GOSR2	GSE92220	.	.	.	.
REG00014	PYURF	GSE92220	.	.	.	.
REG00014	GOT1	GSE92220	.	.	.	.
REG00014	GOT2	GSE92220	.	.	.	.
REG00014	MMP24-AS1	GSE92220	.	.	.	.
REG00014	GP5	GSE92220	.	.	.	.
REG00014	GP9	GSE92220	.	.	.	.
REG00014	GPAA1	GSE92220	.	.	.	.
REG00014	GPC1	GSE92220	.	.	.	.
REG00014	SETX	GSE92220	.	.	.	.
REG00014	GPC3	GSE92220	.	.	.	.
REG00014	GPC4	GSE92220	.	.	.	.
REG00014	GPC6	GSE92220	.	.	.	.
REG00014	GPD1	GSE92220	.	.	.	.
REG00014	GPI	GSE92220	.	.	.	.
REG00014	FAM229A	GSE92220	.	.	.	.
REG00014	CEP295NL	GSE92220	.	.	.	.

REG00014	GPR12	GSE92220	.	.	.	.
REG00014	CCER2	GSE92220	.	.	.	.
REG00014	SMLR1	GSE92220	.	.	.	.
REG00014	UTS2R	GSE92220	.	.	.	.
REG00014	ZBED1	GSE92220	.	.	.	.
REG00014	GPR18	GSE92220	.	.	.	.
REG00014	GPR21	GSE92220	.	.	.	.
REG00014	GPR25	GSE92220	.	.	.	.
REG00014	GPR3	GSE92220	.	.	.	.
REG00014	GPER1	GSE92220	.	.	.	.
REG00014	ALX3	GSE92220	.	.	.	.
REG00014	GPR35	GSE92220	.	.	.	.
REG00014	MLNR	GSE92220	.	.	.	.
REG00014	ABCB4	GSE92220	.	.	.	.
REG00014	ALX4	GSE92220	.	.	.	.
REG00014	PTGDR2	GSE92220	.	.	.	.
REG00014	GPR45	GSE92220	.	.	.	.
REG00014	LGR5	GSE92220	.	.	.	.
REG00014	GABBR2	GSE92220	.	.	.	.
REG00014	ADGRG1	GSE92220	.	.	.	.
REG00014	GPR68	GSE92220	.	.	.	.
REG00014	GPR75	GSE92220	.	.	.	.
REG00014	AMBP	GSE92220	.	.	.	.
REG00014	GPR84	GSE92220	.	.	.	.
REG00014	GRK4	GSE92220	.	.	.	.
REG00014	GRK5	GSE92220	.	.	.	.
REG00014	GRK6	GSE92220	.	.	.	.
REG00014	GPS1	GSE92220	.	.	.	.
REG00014	GPS2	GSE92220	.	.	.	.
REG00014	TECR	GSE92220	.	.	.	.
REG00014	GPT	GSE92220	.	.	.	.
REG00014	GPX1	GSE92220	.	.	.	.
REG00014	GPX2	GSE92220	.	.	.	.
REG00014	GPX3	GSE92220	.	.	.	.
REG00014	GPX4	GSE92220	.	.	.	.
REG00014	GPX7	GSE92220	.	.	.	.
REG00014	GRB10	GSE92220	.	.	.	.
REG00014	GRB2	GSE92220	.	.	.	.
REG00014	GRB7	GSE92220	.	.	.	.
REG00014	RAPGEF1	GSE92220	.	.	.	.
REG00014	AMD1	GSE92220	.	.	.	.
REG00014	GRHPR	GSE92220	.	.	.	.
REG00014	GRID1	GSE92220	.	.	.	.
REG00014	GRIK4	GSE92220	.	.	.	.
REG00014	GRIN2C	GSE92220	.	.	.	.
REG00014	GRINA	GSE92220	.	.	.	.
REG00014	ARHGAP35	GSE92220	.	.	.	.
REG00014	GRM2	GSE92220	.	.	.	.
REG00014	GRN	GSE92220	.	.	.	.
REG00014	PDIA3	GSE92220	.	.	.	.

REG00014	GRSF1	GSE92220	.	.	.	.
REG00014	GSK3A	GSE92220	.	.	.	.
REG00014	GSK3B	GSE92220	.	.	.	.
REG00014	GSN	GSE92220	.	.	.	.
REG00014	GSPT1	GSE92220	.	.	.	.
REG00014	GSR	GSE92220	.	.	.	.
REG00014	GSS	GSE92220	.	.	.	.
REG00014	GSTA1	GSE92220	.	.	.	.
REG00014	AMFR	GSE92220	.	.	.	.
REG00014	GSTM3	GSE92220	.	.	.	.
REG00014	AMH	GSE92220	.	.	.	.
REG00014	GSTZ1	GSE92220	.	.	.	.
REG00014	GTF2A1	GSE92220	.	.	.	.
REG00014	GTF2A2	GSE92220	.	.	.	.
REG00014	GTF2B	GSE92220	.	.	.	.
REG00014	AMHR2	GSE92220	.	.	.	.
REG00014	GTF2E1	GSE92220	.	.	.	.
REG00014	GTF2E2	GSE92220	.	.	.	.
REG00014	GTF2F2	GSE92220	.	.	.	.
REG00014	GTF2H3	GSE92220	.	.	.	.
REG00014	GTF2H4	GSE92220	.	.	.	.
REG00014	GTF2I	GSE92220	.	.	.	.
REG00014	GTF2IRD1	GSE92220	.	.	.	.
REG00014	GTF3A	GSE92220	.	.	.	.
REG00014	GTF3C1	GSE92220	.	.	.	.
REG00014	GTF3C2	GSE92220	.	.	.	.
REG00014	GTF3C3	GSE92220	.	.	.	.
REG00014	GTPBP1	GSE92220	.	.	.	.
REG00014	GTPBP2	GSE92220	.	.	.	.
REG00014	AMPD1	GSE92220	.	.	.	.
REG00014	GUCA2A	GSE92220	.	.	.	.
REG00014	AMPD2	GSE92220	.	.	.	.
REG00014	GUK1	GSE92220	.	.	.	.
REG00014	GUSB	GSE92220	.	.	.	.
REG00014	GYG1	GSE92220	.	.	.	.
REG00014	ABCB6	GSE92220	.	.	.	.
REG00014	GYG2	GSE92220	.	.	.	.
REG00014	GYS1	GSE92220	.	.	.	.
REG00014	GZMB	GSE92220	.	.	.	.
REG00014	GZMM	GSE92220	.	.	.	.
REG00014	H1F0	GSE92220	.	.	.	.
REG00014	HIST1H1C	GSE92220	.	.	.	.
REG00014	HIST1H1E	GSE92220	.	.	.	.
REG00014	HIST1H2AI	GSE92220	.	.	.	.
REG00014	HIST1H2AJ	GSE92220	.	.	.	.
REG00014	AMT	GSE92220	.	.	.	.
REG00014	HIST1H2AC	GSE92220	.	.	.	.
REG00014	HIST1H2AM	GSE92220	.	.	.	.
REG00014	HIST1H2AG	GSE92220	.	.	.	.
REG00014	H2AFX	GSE92220	.	.	.	.

REG00014	H2AFY	GSE92220	.	.	.	.
REG00014	H2AFZ	GSE92220	.	.	.	.
REG00014	HIST1H2BG	GSE92220	.	.	.	.
REG00014	HIST1H2BD	GSE92220	.	.	.	.
REG00014	HIST1H2BN	GSE92220	.	.	.	.
REG00014	HIST1H2BF	GSE92220	.	.	.	.
REG00014	HIST1H2BC	GSE92220	.	.	.	.
REG00014	HIST2H2BE	GSE92220	.	.	.	.
REG00014	H3F3B	GSE92220	.	.	.	.
REG00014	HIST1H3D	GSE92220	.	.	.	.
REG00014	HIST1H3H	GSE92220	.	.	.	.
REG00014	HIST1H3B	GSE92220	.	.	.	.
REG00014	HIST1H4J	GSE92220	.	.	.	.
REG00014	HIST1H4B	GSE92220	.	.	.	.
REG00014	HIST1H4E	GSE92220	.	.	.	.
REG00014	H6PD	GSE92220	.	.	.	.
REG00014	HAAO	GSE92220	.	.	.	.
REG00014	HSD17B10	GSE92220	.	.	.	.
REG00014	HADHA	GSE92220	.	.	.	.
REG00014	HADHB	GSE92220	.	.	.	.
REG00014	HAGH	GSE92220	.	.	.	.
REG00014	HAL	GSE92220	.	.	.	.
REG00014	HAO1	GSE92220	.	.	.	.
REG00014	HAP1	GSE92220	.	.	.	.
REG00014	HARS2	GSE92220	.	.	.	.
REG00014	HAS3	GSE92220	.	.	.	.
REG00014	SMIM22	GSE92220	.	.	.	.
REG00014	HBQ1	GSE92220	.	.	.	.
REG00014	SERPIND1	GSE92220	.	.	.	.
REG00014	HCLS1	GSE92220	.	.	.	.
REG00014	HCN2	GSE92220	.	.	.	.
REG00014	HCRT	GSE92220	.	.	.	.
REG00014	HCRTR1	GSE92220	.	.	.	.
REG00014	HTT	GSE92220	.	.	.	.
REG00014	HDAC2	GSE92220	.	.	.	.
REG00014	HDAC3	GSE92220	.	.	.	.
REG00014	HDLBP	GSE92220	.	.	.	.
REG00014	HELLS	GSE92220	.	.	.	.
REG00014	MRLN	GSE92220	.	.	.	.
REG00014	HERC2	GSE92220	.	.	.	.
REG00014	HEXB	GSE92220	.	.	.	.
REG00014	ANGPTL1	GSE92220	.	.	.	.
REG00014	HGD	GSE92220	.	.	.	.
REG00014	HGFAC	GSE92220	.	.	.	.
REG00014	HGS	GSE92220	.	.	.	.
REG00014	HHEX	GSE92220	.	.	.	.
REG00014	HIC1	GSE92220	.	.	.	.
REG00014	ANGPTL3	GSE92220	.	.	.	.
REG00014	HINT1	GSE92220	.	.	.	.
REG00014	UBE2K	GSE92220	.	.	.	.

REG00014	HIPK3	GSE92220	.	.	.	.
REG00014	AK6	GSE92220	.	.	.	.
REG00014	HIRA	GSE92220	.	.	.	.
REG00014	HIRIP3	GSE92220	.	.	.	.
REG00014	ANK1	GSE92220	.	.	.	.
REG00014	HIVEP1	GSE92220	.	.	.	.
REG00014	HIVEP2	GSE92220	.	.	.	.
REG00014	HK1	GSE92220	.	.	.	.
REG00014	HK3	GSE92220	.	.	.	.
REG00014	PFDN6	GSE92220	.	.	.	.
REG00014	HKR1	GSE92220	.	.	.	.
REG00014	CEBPZOS	GSE92220	.	.	.	.
REG00014	ZBTB48	GSE92220	.	.	.	.
REG00014	HLA-A	GSE92220	.	.	.	.
REG00014	HLA-B	GSE92220	.	.	.	.
REG00014	HLA-C	GSE92220	.	.	.	.
REG00014	HLA-DOB	GSE92220	.	.	.	.
REG00014	TMEM262	GSE92220	.	.	.	.
REG00014	CCDC182	GSE92220	.	.	.	.
REG00014	KANTR	GSE92220	.	.	.	.
REG00014	FAM83H-AS1	GSE92220	.	.	.	.
REG00014	HLA-E	GSE92220	.	.	.	.
REG00014	HLA-F	GSE92220	.	.	.	.
REG00014	HLA-G	GSE92220	.	.	.	.
REG00014	MR1	GSE92220	.	.	.	.
REG00014	HLF	GSE92220	.	.	.	.
REG00014	MNX1	GSE92220	.	.	.	.
REG00014	HMBS	GSE92220	.	.	.	.
REG00014	HMGB1	GSE92220	.	.	.	.
REG00014	HMGN1	GSE92220	.	.	.	.
REG00014	SMIM10L1	GSE92220	.	.	.	.
REG00014	HMGN2	GSE92220	.	.	.	.
REG00014	HMGN4	GSE92220	.	.	.	.
REG00014	A1BG	GSE92220	.	.	.	.
REG00014	ANPEP	GSE92220	.	.	.	.
REG00014	HMGB2	GSE92220	.	.	.	.
REG00014	HMG20B	GSE92220	.	.	.	.
REG00014	HMGXB4	GSE92220	.	.	.	.
REG00014	HMGCL	GSE92220	.	.	.	.
REG00014	HMGCR	GSE92220	.	.	.	.
REG00014	HMGCS1	GSE92220	.	.	.	.
REG00014	HMGCS2	GSE92220	.	.	.	.
REG00014	HMGA2	GSE92220	.	.	.	.
REG00014	HMOX1	GSE92220	.	.	.	.
REG00014	HMOX2	GSE92220	.	.	.	.
REG00014	FOXA1	GSE92220	.	.	.	.
REG00014	FOXA2	GSE92220	.	.	.	.
REG00014	FOXA3	GSE92220	.	.	.	.
REG00014	HNF4A	GSE92220	.	.	.	.
REG00014	HNF4G	GSE92220	.	.	.	.

REG00014	HNMT	GSE92220	.	.	.	.
REG00014	HNRNPA0	GSE92220	.	.	.	.
REG00014	HNRNPA1	GSE92220	.	.	.	.
REG00014	HNRNPA2B1	GSE92220	.	.	.	.
REG00014	HNRNPAB	GSE92220	.	.	.	.
REG00014	HNRNPC	GSE92220	.	.	.	.
REG00014	HNRNPD	GSE92220	.	.	.	.
REG00014	HNRNPDL	GSE92220	.	.	.	.
REG00014	HNRNPF	GSE92220	.	.	.	.
REG00014	HNRNPH1	GSE92220	.	.	.	.
REG00014	HNRNPH3	GSE92220	.	.	.	.
REG00014	HNRNPK	GSE92220	.	.	.	.
REG00014	HNRNPL	GSE92220	.	.	.	.
REG00014	HNRNPM	GSE92220	.	.	.	.
REG00014	HNRNPR	GSE92220	.	.	.	.
REG00014	HNRNPU	GSE92220	.	.	.	.
REG00014	TLX2	GSE92220	.	.	.	.
REG00014	ABCC1	GSE92220	.	.	.	.
REG00014	HOXA2	GSE92220	.	.	.	.
REG00014	HOXA3	GSE92220	.	.	.	.
REG00014	C11orf98	GSE92220	.	.	.	.
REG00014	TMEM265	GSE92220	.	.	.	.
REG00014	RNF225	GSE92220	.	.	.	.
REG00014	HP	GSE92220	.	.	.	.
REG00014	HPCAL1	GSE92220	.	.	.	.
REG00014	HPD	GSE92220	.	.	.	.
REG00014	HPN	GSE92220	.	.	.	.
REG00014	HPR	GSE92220	.	.	.	.
REG00014	HPRT1	GSE92220	.	.	.	.
REG00014	HPX	GSE92220	.	.	.	.
REG00014	UQCRHL	GSE92220	.	.	.	.
REG00014	HRAS	GSE92220	.	.	.	.
REG00014	ERAS	GSE92220	.	.	.	.
REG00014	AGFG1	GSE92220	.	.	.	.
REG00014	KRR1	GSE92220	.	.	.	.
REG00014	AGFG2	GSE92220	.	.	.	.
REG00014	HRC	GSE92220	.	.	.	.
REG00014	PRMT2	GSE92220	.	.	.	.
REG00014	PRMT1	GSE92220	.	.	.	.
REG00014	PRMT8	GSE92220	.	.	.	.
REG00014	CCDC188	GSE92220	.	.	.	.
REG00014	HES1	GSE92220	.	.	.	.
REG00014	HS2ST1	GSE92220	.	.	.	.
REG00014	HS3ST3B1	GSE92220	.	.	.	.
REG00014	HS6ST1	GSE92220	.	.	.	.
REG00014	HSBP1	GSE92220	.	.	.	.
REG00014	HSD11B2	GSE92220	.	.	.	.
REG00014	HSD17B1	GSE92220	.	.	.	.
REG00014	HSD17B2	GSE92220	.	.	.	.
REG00014	HSD17B3	GSE92220	.	.	.	.

REG00014	HSD17B4	GSE92220	.	.	.	.
REG00014	HSF2	GSE92220	.	.	.	.
REG00014	HSF4	GSE92220	.	.	.	.
REG00014	DNAJB2	GSE92220	.	.	.	.
REG00014	DNAJA1	GSE92220	.	.	.	.
REG00014	HSPA1A	GSE92220	.	.	.	.
REG00014	HSPA1B	GSE92220	.	.	.	.
REG00014	HSPA4	GSE92220	.	.	.	.
REG00014	HSPA5	GSE92220	.	.	.	.
REG00014	LOC100130705	GSE92220	.	.	.	.
REG00014	HSPA8	GSE92220	.	.	.	.
REG00014	HSPA9	GSE92220	.	.	.	.
REG00014	HSPB1	GSE92220	.	.	.	.
REG00014	HSP90AA1	GSE92220	.	.	.	.
REG00014	HSP90AB1	GSE92220	.	.	.	.
REG00014	HSPD1	GSE92220	.	.	.	.
REG00014	DNAJB1	GSE92220	.	.	.	.
REG00014	DNAJC4	GSE92220	.	.	.	.
REG00014	HSPG2	GSE92220	.	.	.	.
REG00014	FOXN2	GSE92220	.	.	.	.
REG00014	HTR2B	GSE92220	.	.	.	.
REG00014	ABCC2	GSE92220	.	.	.	.
REG00014	HUS1	GSE92220	.	.	.	.
REG00014	HYAL1	GSE92220	.	.	.	.
REG00014	HYAL2	GSE92220	.	.	.	.
REG00014	HYAL3	GSE92220	.	.	.	.
REG00014	IARS	GSE92220	.	.	.	.
REG00014	ICAM1	GSE92220	.	.	.	.
REG00014	ICAM2	GSE92220	.	.	.	.
REG00014	ICAM3	GSE92220	.	.	.	.
REG00014	ICAM4	GSE92220	.	.	.	.
REG00014	ICAM5	GSE92220	.	.	.	.
REG00014	ANXA11	GSE92220	.	.	.	.
REG00014	ICMT	GSE92220	.	.	.	.
REG00014	MRPL58	GSE92220	.	.	.	.
REG00014	ID1	GSE92220	.	.	.	.
REG00014	ID2	GSE92220	.	.	.	.
REG00014	ID3	GSE92220	.	.	.	.
REG00014	ID4	GSE92220	.	.	.	.
REG00014	LOC389332	GSE92220	.	.	.	.
REG00014	ANXA2	GSE92220	.	.	.	.
REG00014	IDE	GSE92220	.	.	.	.
REG00014	IDH1	GSE92220	.	.	.	.
REG00014	IDH2	GSE92220	.	.	.	.
REG00014	IDH3A	GSE92220	.	.	.	.
REG00014	IDH3B	GSE92220	.	.	.	.
REG00014	IDI1	GSE92220	.	.	.	.
REG00014	IDS	GSE92220	.	.	.	.
REG00014	IDUA	GSE92220	.	.	.	.
REG00014	IER3	GSE92220	.	.	.	.

REG00014	CFI	GSE92220	.	.	.	.
REG00014	IFI30	GSE92220	.	.	.	.
REG00014	IFI35	GSE92220	.	.	.	.
REG00014	ABCC3	GSE92220	.	.	.	.
REG00014	SP110	GSE92220	.	.	.	.
REG00014	ANXA3	GSE92220	.	.	.	.
REG00014	IFITM2	GSE92220	.	.	.	.
REG00014	IFITM3	GSE92220	.	.	.	.
REG00014	ANXA4	GSE92220	.	.	.	.
REG00014	ANXA5	GSE92220	.	.	.	.
REG00014	IFNAR1	GSE92220	.	.	.	.
REG00014	IFNAR2	GSE92220	.	.	.	.
REG00014	IFNGR1	GSE92220	.	.	.	.
REG00014	ANXA6	GSE92220	.	.	.	.
REG00014	IFNGR2	GSE92220	.	.	.	.
REG00014	IFRD1	GSE92220	.	.	.	.
REG00014	IFRD2	GSE92220	.	.	.	.
REG00014	IGF1R	GSE92220	.	.	.	.
REG00014	IGF2	GSE92220	.	.	.	.
REG00014	IGF2R	GSE92220	.	.	.	.
REG00014	IGFALS	GSE92220	.	.	.	.
REG00014	IGFBP1	GSE92220	.	.	.	.
REG00014	ANXA9	GSE92220	.	.	.	.
REG00014	IGFBP3	GSE92220	.	.	.	.
REG00014	IGFBP4	GSE92220	.	.	.	.
REG00014	AOC2	GSE92220	.	.	.	.
REG00014	AOC3	GSE92220	.	.	.	.
REG00014	AOX1	GSE92220	.	.	.	.
REG00014	IGHMBP2	GSE92220	.	.	.	.
REG00014	AP1G1	GSE92220	.	.	.	.
REG00014	AP1G2	GSE92220	.	.	.	.
REG00014	SYNRG	GSE92220	.	.	.	.
REG00014	ABCC5	GSE92220	.	.	.	.
REG00014	AP2A1	GSE92220	.	.	.	.
REG00014	AP2A2	GSE92220	.	.	.	.
REG00014	AP2B1	GSE92220	.	.	.	.
REG00014	AP2M1	GSE92220	.	.	.	.
REG00014	AP2S1	GSE92220	.	.	.	.
REG00014	AP3B1	GSE92220	.	.	.	.
REG00014	AP3D1	GSE92220	.	.	.	.
REG00014	AP3M1	GSE92220	.	.	.	.
REG00014	ABCC6	GSE92220	.	.	.	.
REG00014	RBPJ	GSE92220	.	.	.	.
REG00014	AP4S1	GSE92220	.	.	.	.
REG00014	APBA1	GSE92220	.	.	.	.
REG00014	APBB2	GSE92220	.	.	.	.
REG00014	APEH	GSE92220	.	.	.	.
REG00014	APEX1	GSE92220	.	.	.	.
REG00014	ATG12	GSE92220	.	.	.	.
REG00014	BIRC2	GSE92220	.	.	.	.

REG00014	BIRC3	GSE92220	.	.	.	.
REG00014	XIAP	GSE92220	.	.	.	.
REG00014	BIRC5	GSE92220	.	.	.	.
REG00014	API5	GSE92220	.	.	.	.
REG00014	IGSF1	GSE92220	.	.	.	.
REG00014	CD101	GSE92220	.	.	.	.
REG00014	CADM1	GSE92220	.	.	.	.
REG00014	IGSF6	GSE92220	.	.	.	.
REG00014	IKBKAP	GSE92220	.	.	.	.
REG00014	IKBKB	GSE92220	.	.	.	.
REG00014	IKBKG	GSE92220	.	.	.	.
REG00014	IL10	GSE92220	.	.	.	.
REG00014	IL10RB	GSE92220	.	.	.	.
REG00014	IL12RB1	GSE92220	.	.	.	.
REG00014	IL15RA	GSE92220	.	.	.	.
REG00014	APLP2	GSE92220	.	.	.	.
REG00014	IL17C	GSE92220	.	.	.	.
REG00014	IL18BP	GSE92220	.	.	.	.
REG00014	IL1R1	GSE92220	.	.	.	.
REG00014	IL1RAP	GSE92220	.	.	.	.
REG00014	IL1RL2	GSE92220	.	.	.	.
REG00014	APOA1	GSE92220	.	.	.	.
REG00014	IL1RN	GSE92220	.	.	.	.
REG00014	IL2RB	GSE92220	.	.	.	.
REG00014	APOA2	GSE92220	.	.	.	.
REG00014	IL4R	GSE92220	.	.	.	.
REG00014	IL6ST	GSE92220	.	.	.	.
REG00014	APOB	GSE92220	.	.	.	.
REG00014	FOXK2	GSE92220	.	.	.	.
REG00014	ILF2	GSE92220	.	.	.	.
REG00014	ILF3	GSE92220	.	.	.	.
REG00014	ILK	GSE92220	.	.	.	.
REG00014	ILVBL	GSE92220	.	.	.	.
REG00014	IMMT	GSE92220	.	.	.	.
REG00014	IMPA1	GSE92220	.	.	.	.
REG00014	IMPA2	GSE92220	.	.	.	.
REG00014	IMPDH1	GSE92220	.	.	.	.
REG00014	IMPDH2	GSE92220	.	.	.	.
REG00014	IMPG1	GSE92220	.	.	.	.
REG00014	ING1	GSE92220	.	.	.	.
REG00014	APOC1	GSE92220	.	.	.	.
REG00014	INPP1	GSE92220	.	.	.	.
REG00014	INPP5A	GSE92220	.	.	.	.
REG00014	INPP5B	GSE92220	.	.	.	.
REG00014	INPP5D	GSE92220	.	.	.	.
REG00014	INPPL1	GSE92220	.	.	.	.
REG00014	INSL3	GSE92220	.	.	.	.
REG00014	INSM1	GSE92220	.	.	.	.
REG00014	INSR	GSE92220	.	.	.	.
REG00014	ABCD1	GSE92220	.	.	.	.

REG00014	APOC3	GSE92220	.	.	.	.
REG00014	IQGAP1	GSE92220	.	.	.	.
REG00014	IQGAP2	GSE92220	.	.	.	.
REG00014	IRAK1	GSE92220	.	.	.	.
REG00014	IREB2	GSE92220	.	.	.	.
REG00014	IRF2	GSE92220	.	.	.	.
REG00014	IRF4	GSE92220	.	.	.	.
REG00014	IRF5	GSE92220	.	.	.	.
REG00014	IRF7	GSE92220	.	.	.	.
REG00014	IRS1	GSE92220	.	.	.	.
REG00014	IRS4	GSE92220	.	.	.	.
REG00014	APOE	GSE92220	.	.	.	.
REG00014	ISG20	GSE92220	.	.	.	.
REG00014	IRF9	GSE92220	.	.	.	.
REG00014	ITGA1	GSE92220	.	.	.	.
REG00014	ITGA2	GSE92220	.	.	.	.
REG00014	ITGA2B	GSE92220	.	.	.	.
REG00014	ITGA6	GSE92220	.	.	.	.
REG00014	ITGAE	GSE92220	.	.	.	.
REG00014	ITGAV	GSE92220	.	.	.	.
REG00014	ITGB1	GSE92220	.	.	.	.
REG00014	ITGB1BP2	GSE92220	.	.	.	.
REG00014	ITGB4	GSE92220	.	.	.	.
REG00014	EIF6	GSE92220	.	.	.	.
REG00014	APOH	GSE92220	.	.	.	.
REG00014	ITGB5	GSE92220	.	.	.	.
REG00014	ITIH2	GSE92220	.	.	.	.
REG00014	ITIH3	GSE92220	.	.	.	.
REG00014	STT3A	GSE92220	.	.	.	.
REG00014	ITM2B	GSE92220	.	.	.	.
REG00014	ITM2C	GSE92220	.	.	.	.
REG00014	ITPA	GSE92220	.	.	.	.
REG00014	ITPKA	GSE92220	.	.	.	.
REG00014	APOL1	GSE92220	.	.	.	.
REG00014	ITPR2	GSE92220	.	.	.	.
REG00014	ITPR3	GSE92220	.	.	.	.
REG00014	ITSN1	GSE92220	.	.	.	.
REG00014	ITSN2	GSE92220	.	.	.	.
REG00014	IVD	GSE92220	.	.	.	.
REG00014	JAG1	GSE92220	.	.	.	.
REG00014	APOL2	GSE92220	.	.	.	.
REG00014	JAK2	GSE92220	.	.	.	.
REG00014	JAK3	GSE92220	.	.	.	.
REG00014	JARID2	GSE92220	.	.	.	.
REG00014	JRK	GSE92220	.	.	.	.
REG00014	APP	GSE92220	.	.	.	.
REG00014	JTB	GSE92220	.	.	.	.
REG00014	JUN	GSE92220	.	.	.	.
REG00014	JUNB	GSE92220	.	.	.	.
REG00014	JUND	GSE92220	.	.	.	.

REG00014	KARS	GSE92220	.	.	.	.
REG00014	KATNA1	GSE92220	.	.	.	.
REG00014	APPBP2	GSE92220	.	.	.	.
REG00014	KCNAB2	GSE92220	.	.	.	.
REG00014	KCNAB3	GSE92220	.	.	.	.
REG00014	KCNC3	GSE92220	.	.	.	.
REG00014	KCND1	GSE92220	.	.	.	.
REG00014	KCNE1	GSE92220	.	.	.	.
REG00014	KCNE5	GSE92220	.	.	.	.
REG00014	KCNG2	GSE92220	.	.	.	.
REG00014	KCNH3	GSE92220	.	.	.	.
REG00014	KCNJ11	GSE92220	.	.	.	.
REG00014	KCNJ13	GSE92220	.	.	.	.
REG00014	APRT	GSE92220	.	.	.	.
REG00014	KCNK4	GSE92220	.	.	.	.
REG00014	KCNK7	GSE92220	.	.	.	.
REG00014	KCNK9	GSE92220	.	.	.	.
REG00014	KCNMB3	GSE92220	.	.	.	.
REG00014	KCNMB4	GSE92220	.	.	.	.
REG00014	KCNQ4	GSE92220	.	.	.	.
REG00014	KCNS1	GSE92220	.	.	.	.
REG00014	KDELR1	GSE92220	.	.	.	.
REG00014	KDELR2	GSE92220	.	.	.	.
REG00014	KDELR3	GSE92220	.	.	.	.
REG00014	KHK	GSE92220	.	.	.	.
REG00014	KIF1C	GSE92220	.	.	.	.
REG00014	KIF2A	GSE92220	.	.	.	.
REG00014	KIF3B	GSE92220	.	.	.	.
REG00014	KIF3C	GSE92220	.	.	.	.
REG00014	KIF5B	GSE92220	.	.	.	.
REG00014	KIF5C	GSE92220	.	.	.	.
REG00014	KIFC3	GSE92220	.	.	.	.
REG00014	KIN	GSE92220	.	.	.	.
REG00014	KISS1	GSE92220	.	.	.	.
REG00014	KLF2	GSE92220	.	.	.	.
REG00014	KLHL5	GSE92220	.	.	.	.
REG00014	KLC1	GSE92220	.	.	.	.
REG00014	KPNA1	GSE92220	.	.	.	.
REG00014	KPNA2	GSE92220	.	.	.	.
REG00014	KPNA3	GSE92220	.	.	.	.
REG00014	KPNA4	GSE92220	.	.	.	.
REG00014	KPNA5	GSE92220	.	.	.	.
REG00014	KPNA6	GSE92220	.	.	.	.
REG00014	AQP7	GSE92220	.	.	.	.
REG00014	KPNB1	GSE92220	.	.	.	.
REG00014	TNPO1	GSE92220	.	.	.	.
REG00014	IPO5	GSE92220	.	.	.	.
REG00014	KPTN	GSE92220	.	.	.	.
REG00014	KRT10	GSE92220	.	.	.	.
REG00014	KRT18	GSE92220	.	.	.	.

REG00014	KRT19	GSE92220	.	.	.	.
REG00014	KRT8	GSE92220	.	.	.	.
REG00014	ARAF	GSE92220	.	.	.	.
REG00014	KSR1	GSE92220	.	.	.	.
REG00014	KTN1	GSE92220	.	.	.	.
REG00014	KYNU	GSE92220	.	.	.	.
REG00014	LAG3	GSE92220	.	.	.	.
REG00014	LAMA5	GSE92220	.	.	.	.
REG00014	LAMB1	GSE92220	.	.	.	.
REG00014	LAMB2	GSE92220	.	.	.	.
REG00014	LAMB4	GSE92220	.	.	.	.
REG00014	LAMC1	GSE92220	.	.	.	.
REG00014	LAMC2	GSE92220	.	.	.	.
REG00014	LAMP1	GSE92220	.	.	.	.
REG00014	LAMP2	GSE92220	.	.	.	.
REG00014	RPSA	GSE92220	.	.	.	.
REG00014	LANCL1	GSE92220	.	.	.	.
REG00014	LANCL2	GSE92220	.	.	.	.
REG00014	STMN1	GSE92220	.	.	.	.
REG00014	LARGE1	GSE92220	.	.	.	.
REG00014	LARS	GSE92220	.	.	.	.
REG00014	LASP1	GSE92220	.	.	.	.
REG00014	LATS1	GSE92220	.	.	.	.
REG00014	LATS2	GSE92220	.	.	.	.
REG00014	LBR	GSE92220	.	.	.	.
REG00014	LCAT	GSE92220	.	.	.	.
REG00014	LCT	GSE92220	.	.	.	.
REG00014	LDB1	GSE92220	.	.	.	.
REG00014	LDHA	GSE92220	.	.	.	.
REG00014	ARF3	GSE92220	.	.	.	.
REG00014	LDHC	GSE92220	.	.	.	.
REG00014	COG1	GSE92220	.	.	.	.
REG00014	LDLR	GSE92220	.	.	.	.
REG00014	ARF4	GSE92220	.	.	.	.
REG00014	LEPROTL1	GSE92220	.	.	.	.
REG00014	LETM1	GSE92220	.	.	.	.
REG00014	ARL4D	GSE92220	.	.	.	.
REG00014	LGALS1	GSE92220	.	.	.	.
REG00014	LGALS3BP	GSE92220	.	.	.	.
REG00014	LGALS4	GSE92220	.	.	.	.
REG00014	LGALS9	GSE92220	.	.	.	.
REG00014	EIF2D	GSE92220	.	.	.	.
REG00014	LHB	GSE92220	.	.	.	.
REG00014	LHFPL2	GSE92220	.	.	.	.
REG00014	ARF6	GSE92220	.	.	.	.
REG00014	LHX3	GSE92220	.	.	.	.
REG00014	LIF	GSE92220	.	.	.	.
REG00014	LIFR	GSE92220	.	.	.	.
REG00014	LIG1	GSE92220	.	.	.	.
REG00014	LIG3	GSE92220	.	.	.	.

REG00014	LIG4	GSE92220	.	.	.	.
REG00014	ARFGAP3	GSE92220	.	.	.	.
REG00014	LIMD1	GSE92220	.	.	.	.
REG00014	LIMK1	GSE92220	.	.	.	.
REG00014	LIMK2	GSE92220	.	.	.	.
REG00014	LIMS1	GSE92220	.	.	.	.
REG00014	LLGL1	GSE92220	.	.	.	.
REG00014	LLGL2	GSE92220	.	.	.	.
REG00014	LMAN1	GSE92220	.	.	.	.
REG00014	LMAN1L	GSE92220	.	.	.	.
REG00014	LMNA	GSE92220	.	.	.	.
REG00014	LMNB1	GSE92220	.	.	.	.
REG00014	LMNB2	GSE92220	.	.	.	.
REG00014	LMO4	GSE92220	.	.	.	.
REG00014	LMO7	GSE92220	.	.	.	.
REG00014	LMOD2	GSE92220	.	.	.	.
REG00014	LMOD3	GSE92220	.	.	.	.
REG00014	LNPEP	GSE92220	.	.	.	.
REG00014	LOX	GSE92220	.	.	.	.
REG00014	LOXL2	GSE92220	.	.	.	.
REG00014	LPP	GSE92220	.	.	.	.
REG00014	RHOB	GSE92220	.	.	.	.
REG00014	RHOC	GSE92220	.	.	.	.
REG00014	LRCH4	GSE92220	.	.	.	.
REG00014	LRP1	GSE92220	.	.	.	.
REG00014	LRP3	GSE92220	.	.	.	.
REG00014	LRP5	GSE92220	.	.	.	.
REG00014	ABCD3	GSE92220	.	.	.	.
REG00014	LRP8	GSE92220	.	.	.	.
REG00014	LRPAP1	GSE92220	.	.	.	.
REG00014	LRRFIP1	GSE92220	.	.	.	.
REG00014	LSS	GSE92220	.	.	.	.
REG00014	LTA4H	GSE92220	.	.	.	.
REG00014	LTB4R	GSE92220	.	.	.	.
REG00014	LTBP2	GSE92220	.	.	.	.
REG00014	LTBP3	GSE92220	.	.	.	.
REG00014	LTBR	GSE92220	.	.	.	.
REG00014	LTC4S	GSE92220	.	.	.	.
REG00014	RHOG	GSE92220	.	.	.	.
REG00014	LTK	GSE92220	.	.	.	.
REG00014	BCAM	GSE92220	.	.	.	.
REG00014	LUC7L	GSE92220	.	.	.	.
REG00014	CD180	GSE92220	.	.	.	.
REG00014	LY6E	GSE92220	.	.	.	.
REG00014	ARHGAP1	GSE92220	.	.	.	.
REG00014	LYL1	GSE92220	.	.	.	.
REG00014	LYPLA1	GSE92220	.	.	.	.
REG00014	LYPLA2	GSE92220	.	.	.	.
REG00014	ARHGAP4	GSE92220	.	.	.	.
REG00014	LYZ	GSE92220	.	.	.	.

REG00014	LZTFL1	GSE92220	.	.	.	.
REG00014	LZTR1	GSE92220	.	.	.	.
REG00014	CAPRIN1	GSE92220	.	.	.	.
REG00014	NBR1	GSE92220	.	.	.	.
REG00014	ARHGAP5	GSE92220	.	.	.	.
REG00014	M6PR	GSE92220	.	.	.	.
REG00014	MAB21L2	GSE92220	.	.	.	.
REG00014	MARCKS	GSE92220	.	.	.	.
REG00014	MXD1	GSE92220	.	.	.	.
REG00014	MAD1L1	GSE92220	.	.	.	.
REG00014	MAD2L2	GSE92220	.	.	.	.
REG00014	MADD	GSE92220	.	.	.	.
REG00014	SMAD1	GSE92220	.	.	.	.
REG00014	SMAD2	GSE92220	.	.	.	.
REG00014	SMAD3	GSE92220	.	.	.	.
REG00014	ARHGAP8	GSE92220	.	.	.	.
REG00014	SMAD4	GSE92220	.	.	.	.
REG00014	SMAD5	GSE92220	.	.	.	.
REG00014	SMAD6	GSE92220	.	.	.	.
REG00014	SMAD7	GSE92220	.	.	.	.
REG00014	ARHGDIA	GSE92220	.	.	.	.
REG00014	MAFG	GSE92220	.	.	.	.
REG00014	MAFK	GSE92220	.	.	.	.
REG00014	ABCD4	GSE92220	.	.	.	.
REG00014	ARHGEF1	GSE92220	.	.	.	.
REG00014	MAGED1	GSE92220	.	.	.	.
REG00014	MAGOH	GSE92220	.	.	.	.
REG00014	ARHGEF2	GSE92220	.	.	.	.
REG00014	MAN1A1	GSE92220	.	.	.	.
REG00014	MAN1A2	GSE92220	.	.	.	.
REG00014	MAN2A1	GSE92220	.	.	.	.
REG00014	MAN2A2	GSE92220	.	.	.	.
REG00014	MAN2B1	GSE92220	.	.	.	.
REG00014	MAN2C1	GSE92220	.	.	.	.
REG00014	ARHGEF3	GSE92220	.	.	.	.
REG00014	MANBA	GSE92220	.	.	.	.
REG00014	MAOA	GSE92220	.	.	.	.
REG00014	MAP1B	GSE92220	.	.	.	.
REG00014	MAP1LC3A	GSE92220	.	.	.	.
REG00014	MAP2K1	GSE92220	.	.	.	.
REG00014	MAP2K2	GSE92220	.	.	.	.
REG00014	MAP2K3	GSE92220	.	.	.	.
REG00014	MAP2K5	GSE92220	.	.	.	.
REG00014	MAP2K7	GSE92220	.	.	.	.
REG00014	MAP3K1	GSE92220	.	.	.	.
REG00014	MAP3K10	GSE92220	.	.	.	.
REG00014	MAP3K11	GSE92220	.	.	.	.
REG00014	MAP3K13	GSE92220	.	.	.	.
REG00014	MAP3K2	GSE92220	.	.	.	.
REG00014	MAP3K3	GSE92220	.	.	.	.

REG00014	MAP3K4	GSE92220	.	.	.	.
REG00014	MAP3K7	GSE92220	.	.	.	.
REG00014	RHOH	GSE92220	.	.	.	.
REG00014	MAP3K8	GSE92220	.	.	.	.
REG00014	MAP4	GSE92220	.	.	.	.
REG00014	MAP4K2	GSE92220	.	.	.	.
REG00014	MAP4K3	GSE92220	.	.	.	.
REG00014	MAP4K4	GSE92220	.	.	.	.
REG00014	MAP4K5	GSE92220	.	.	.	.
REG00014	MAPK1	GSE92220	.	.	.	.
REG00014	MAPK11	GSE92220	.	.	.	.
REG00014	MAPK12	GSE92220	.	.	.	.
REG00014	MAPK13	GSE92220	.	.	.	.
REG00014	MAPK14	GSE92220	.	.	.	.
REG00014	MAPK6	GSE92220	.	.	.	.
REG00014	MAPK8	GSE92220	.	.	.	.
REG00014	MAPK8IP1	GSE92220	.	.	.	.
REG00014	MAPK8IP2	GSE92220	.	.	.	.
REG00014	MAPK8IP3	GSE92220	.	.	.	.
REG00014	MAPK9	GSE92220	.	.	.	.
REG00014	MAPKAPK2	GSE92220	.	.	.	.
REG00014	MAPKAPK5	GSE92220	.	.	.	.
REG00014	ARIH1	GSE92220	.	.	.	.
REG00014	MAPRE1	GSE92220	.	.	.	.
REG00014	MAPRE2	GSE92220	.	.	.	.
REG00014	MARK3	GSE92220	.	.	.	.
REG00014	MARS	GSE92220	.	.	.	.
REG00014	ARIH2	GSE92220	.	.	.	.
REG00014	MASP1	GSE92220	.	.	.	.
REG00014	MASP2	GSE92220	.	.	.	.
REG00014	MAT1A	GSE92220	.	.	.	.
REG00014	MAT2A	GSE92220	.	.	.	.
REG00014	MATN2	GSE92220	.	.	.	.
REG00014	MATN3	GSE92220	.	.	.	.
REG00014	PHOX2A	GSE92220	.	.	.	.
REG00014	MATN4	GSE92220	.	.	.	.
REG00014	MATR3	GSE92220	.	.	.	.
REG00014	MAZ	GSE92220	.	.	.	.
REG00014	MBD1	GSE92220	.	.	.	.
REG00014	MBD2	GSE92220	.	.	.	.
REG00014	MBD3	GSE92220	.	.	.	.
REG00014	ARL1	GSE92220	.	.	.	.
REG00014	MBL2	GSE92220	.	.	.	.
REG00014	MBNL1	GSE92220	.	.	.	.
REG00014	LAPTM4A	GSE92220	.	.	.	.
REG00014	MBP	GSE92220	.	.	.	.
REG00014	MC1R	GSE92220	.	.	.	.
REG00014	MCCC2	GSE92220	.	.	.	.
REG00014	MCL1	GSE92220	.	.	.	.
REG00014	MCM2	GSE92220	.	.	.	.

REG00014	MCM3	GSE92220	.	.	.	.
REG00014	MCM3AP	GSE92220	.	.	.	.
REG00014	MCM4	GSE92220	.	.	.	.
REG00014	MCM5	GSE92220	.	.	.	.
REG00014	MCM6	GSE92220	.	.	.	.
REG00014	MCM7	GSE92220	.	.	.	.
REG00014	CD46	GSE92220	.	.	.	.
REG00014	ARL5A	GSE92220	.	.	.	.
REG00014	MCRS1	GSE92220	.	.	.	.
REG00014	MDFI	GSE92220	.	.	.	.
REG00014	ARL6IP1	GSE92220	.	.	.	.
REG00014	MDH1	GSE92220	.	.	.	.
REG00014	MDH2	GSE92220	.	.	.	.
REG00014	MDK	GSE92220	.	.	.	.
REG00014	MDM2	GSE92220	.	.	.	.
REG00014	MDM4	GSE92220	.	.	.	.
REG00014	MEA1	GSE92220	.	.	.	.
REG00014	MECP2	GSE92220	.	.	.	.
REG00014	MEF2A	GSE92220	.	.	.	.
REG00014	MEF2B	GSE92220	.	.	.	.
REG00014	MEF2D	GSE92220	.	.	.	.
REG00014	A2M	GSE92220	.	.	.	.
REG00014	ABCF1	GSE92220	.	.	.	.
REG00014	MEIS1	GSE92220	.	.	.	.
REG00014	RAB8A	GSE92220	.	.	.	.
REG00014	MEN1	GSE92220	.	.	.	.
REG00014	MERTK	GSE92220	.	.	.	.
REG00014	MET	GSE92220	.	.	.	.
REG00014	ARPC1A	GSE92220	.	.	.	.
REG00014	MFAP1	GSE92220	.	.	.	.
REG00014	MFAP2	GSE92220	.	.	.	.
REG00014	MFAP3	GSE92220	.	.	.	.
REG00014	MELTF	GSE92220	.	.	.	.
REG00014	MFNG	GSE92220	.	.	.	.
REG00014	ARPC1B	GSE92220	.	.	.	.
REG00014	MGAT1	GSE92220	.	.	.	.
REG00014	MGAT2	GSE92220	.	.	.	.
REG00014	MGAT4A	GSE92220	.	.	.	.
REG00014	MGAT4B	GSE92220	.	.	.	.
REG00014	MGAT5	GSE92220	.	.	.	.
REG00014	ARPC2	GSE92220	.	.	.	.
REG00014	MGEA5	GSE92220	.	.	.	.
REG00014	MGMT	GSE92220	.	.	.	.
REG00014	ARPC3	GSE92220	.	.	.	.
REG00014	MGST1	GSE92220	.	.	.	.
REG00014	MGST2	GSE92220	.	.	.	.
REG00014	ARPC4	GSE92220	.	.	.	.
REG00014	MIA	GSE92220	.	.	.	.
REG00014	ARPC5	GSE92220	.	.	.	.
REG00014	CD99	GSE92220	.	.	.	.

REG00014	MICA	GSE92220	.	.	.	.
REG00014	MIF	GSE92220	.	.	.	.
REG00014	ABCF2	GSE92220	.	.	.	.
REG00014	MINPP1	GSE92220	.	.	.	.
REG00014	MKI67	GSE92220	.	.	.	.
REG00014	MKKS	GSE92220	.	.	.	.
REG00014	MKLN1	GSE92220	.	.	.	.
REG00014	ARRB1	GSE92220	.	.	.	.
REG00014	MKNK2	GSE92220	.	.	.	.
REG00014	MKRN1	GSE92220	.	.	.	.
REG00014	MKRN2	GSE92220	.	.	.	.
REG00014	ARRB2	GSE92220	.	.	.	.
REG00014	MLANA	GSE92220	.	.	.	.
REG00014	MLF2	GSE92220	.	.	.	.
REG00014	MLH1	GSE92220	.	.	.	.
REG00014	ARSA	GSE92220	.	.	.	.
REG00014	KMT2A	GSE92220	.	.	.	.
REG00014	KMT2D	GSE92220	.	.	.	.
REG00014	MLLT1	GSE92220	.	.	.	.
REG00014	AFF1	GSE92220	.	.	.	.
REG00014	AFDN	GSE92220	.	.	.	.
REG00014	MLLT6	GSE92220	.	.	.	.
REG00014	FOXO4	GSE92220	.	.	.	.
REG00014	ARSB	GSE92220	.	.	.	.
REG00014	GSDMC	GSE92220	.	.	.	.
REG00014	MMD	GSE92220	.	.	.	.
REG00014	MMP11	GSE92220	.	.	.	.
REG00014	MMP15	GSE92220	.	.	.	.
REG00014	MMP17	GSE92220	.	.	.	.
REG00014	ARSD	GSE92220	.	.	.	.
REG00014	MMP23B	GSE92220	.	.	.	.
REG00014	ALDH6A1	GSE92220	.	.	.	.
REG00014	MN1	GSE92220	.	.	.	.
REG00014	MNAT1	GSE92220	.	.	.	.
REG00014	MNT	GSE92220	.	.	.	.
REG00014	ARSE	GSE92220	.	.	.	.
REG00014	MOCS2	GSE92220	.	.	.	.
REG00014	ABCF3	GSE92220	.	.	.	.
REG00014	MOV10	GSE92220	.	.	.	.
REG00014	MPG	GSE92220	.	.	.	.
REG00014	MPHOSPH10	GSE92220	.	.	.	.
REG00014	MPHOSPH9	GSE92220	.	.	.	.
REG00014	MPI	GSE92220	.	.	.	.
REG00014	MPST	GSE92220	.	.	.	.
REG00014	MPV17	GSE92220	.	.	.	.
REG00014	MPZ	GSE92220	.	.	.	.
REG00014	MPZL1	GSE92220	.	.	.	.
REG00014	ARTN	GSE92220	.	.	.	.
REG00014	ARVCF	GSE92220	.	.	.	.
REG00014	SEPTIN9	GSE92220	.	.	.	.

REG00014	MSH4	GSE92220	.	.	.	.
REG00014	MSH5	GSE92220	.	.	.	.
REG00014	MSH6	GSE92220	.	.	.	.
REG00014	ASAH1	GSE92220	.	.	.	.
REG00014	MST1	GSE92220	.	.	.	.
REG00014	MSX1	GSE92220	.	.	.	.
REG00014	MT1E	GSE92220	.	.	.	.
REG00014	ASCL3	GSE92220	.	.	.	.
REG00014	MT2A	GSE92220	.	.	.	.
REG00014	MTA1	GSE92220	.	.	.	.
REG00014	MTA2	GSE92220	.	.	.	.
REG00014	MTAP	GSE92220	.	.	.	.
REG00014	ASGR1	GSE92220	.	.	.	.
REG00014	ASGR2	GSE92220	.	.	.	.
REG00014	MTHFD1	GSE92220	.	.	.	.
REG00014	MTHFD2	GSE92220	.	.	.	.
REG00014	MTHFR	GSE92220	.	.	.	.
REG00014	MTHFS	GSE92220	.	.	.	.
REG00014	ASH2L	GSE92220	.	.	.	.
REG00014	TESMIN	GSE92220	.	.	.	.
REG00014	MTMR1	GSE92220	.	.	.	.
REG00014	ASIP	GSE92220	.	.	.	.
REG00014	MTMR2	GSE92220	.	.	.	.
REG00014	MTMR3	GSE92220	.	.	.	.
REG00014	MTMR4	GSE92220	.	.	.	.
REG00014	ASL	GSE92220	.	.	.	.
REG00014	MTNR1A	GSE92220	.	.	.	.
REG00014	MTTP	GSE92220	.	.	.	.
REG00014	MTR	GSE92220	.	.	.	.
REG00014	MTRF1	GSE92220	.	.	.	.
REG00014	MTRR	GSE92220	.	.	.	.
REG00014	ASMT	GSE92220	.	.	.	.
REG00014	MTX2	GSE92220	.	.	.	.
REG00014	ASMTL	GSE92220	.	.	.	.
REG00014	MUC13	GSE92220	.	.	.	.
REG00014	MUC6	GSE92220	.	.	.	.
REG00014	ASNA1	GSE92220	.	.	.	.
REG00014	TRIM37	GSE92220	.	.	.	.
REG00014	MVD	GSE92220	.	.	.	.
REG00014	ASNS	GSE92220	.	.	.	.
REG00014	MVK	GSE92220	.	.	.	.
REG00014	MVP	GSE92220	.	.	.	.
REG00014	MX1	GSE92220	.	.	.	.
REG00014	MX2	GSE92220	.	.	.	.
REG00014	MXI1	GSE92220	.	.	.	.
REG00014	MXRA7	GSE92220	.	.	.	.
REG00014	MXRA8	GSE92220	.	.	.	.
REG00014	MYADM	GSE92220	.	.	.	.
REG00014	MYBBP1A	GSE92220	.	.	.	.
REG00014	MYBL2	GSE92220	.	.	.	.

REG00014	MYBPC3	GSE92220	.	.	.	.
REG00014	MYD88	GSE92220	.	.	.	.
REG00014	MYH11	GSE92220	.	.	.	.
REG00014	ASPH	GSE92220	.	.	.	.
REG00014	MYH3	GSE92220	.	.	.	.
REG00014	MYH9	GSE92220	.	.	.	.
REG00014	ASS1	GSE92220	.	.	.	.
REG00014	MYL5	GSE92220	.	.	.	.
REG00014	MYL6	GSE92220	.	.	.	.
REG00014	MYLK	GSE92220	.	.	.	.
REG00014	MYO15A	GSE92220	.	.	.	.
REG00014	MYO1A	GSE92220	.	.	.	.
REG00014	MYO1B	GSE92220	.	.	.	.
REG00014	MYO1C	GSE92220	.	.	.	.
REG00014	ABL1	GSE92220	.	.	.	.
REG00014	MYO5A	GSE92220	.	.	.	.
REG00014	MYO5C	GSE92220	.	.	.	.
REG00014	MYO6	GSE92220	.	.	.	.
REG00014	MYO7A	GSE92220	.	.	.	.
REG00014	MYOC	GSE92220	.	.	.	.
REG00014	PPP1R12A	GSE92220	.	.	.	.
REG00014	PPP1R12B	GSE92220	.	.	.	.
REG00014	NAB1	GSE92220	.	.	.	.
REG00014	NAB2	GSE92220	.	.	.	.
REG00014	NACA	GSE92220	.	.	.	.
REG00014	NAGA	GSE92220	.	.	.	.
REG00014	NAGLU	GSE92220	.	.	.	.
REG00014	NAP1L1	GSE92220	.	.	.	.
REG00014	NAP1L4	GSE92220	.	.	.	.
REG00014	NAPG	GSE92220	.	.	.	.
REG00014	NARS	GSE92220	.	.	.	.
REG00014	NASP	GSE92220	.	.	.	.
REG00014	NAT2	GSE92220	.	.	.	.
REG00014	NBEA	GSE92220	.	.	.	.
REG00014	NCBP1	GSE92220	.	.	.	.
REG00014	NCBP2	GSE92220	.	.	.	.
REG00014	NCF1	GSE92220	.	.	.	.
REG00014	NCF2	GSE92220	.	.	.	.
REG00014	NCK1	GSE92220	.	.	.	.
REG00014	NCK2	GSE92220	.	.	.	.
REG00014	NCKAP1	GSE92220	.	.	.	.
REG00014	NCL	GSE92220	.	.	.	.
REG00014	NCOA2	GSE92220	.	.	.	.
REG00014	NCOA3	GSE92220	.	.	.	.
REG00014	NCOA4	GSE92220	.	.	.	.
REG00014	NCOR1	GSE92220	.	.	.	.
REG00014	NCOR2	GSE92220	.	.	.	.
REG00014	NDRG1	GSE92220	.	.	.	.
REG00014	NDST1	GSE92220	.	.	.	.
REG00014	NDST2	GSE92220	.	.	.	.

REG00014	NDUFA10	GSE92220	.	.	.	.
REG00014	NDUFA2	GSE92220	.	.	.	.
REG00014	NDUFA3	GSE92220	.	.	.	.
REG00014	NDUFA4	GSE92220	.	.	.	.
REG00014	NDUFA8	GSE92220	.	.	.	.
REG00014	NDUFA9	GSE92220	.	.	.	.
REG00014	NDUFAB1	GSE92220	.	.	.	.
REG00014	NDUFB4	GSE92220	.	.	.	.
REG00014	ABL2	GSE92220	.	.	.	.
REG00014	NDUFB5	GSE92220	.	.	.	.
REG00014	NDUFB7	GSE92220	.	.	.	.
REG00014	NDUFB8	GSE92220	.	.	.	.
REG00014	NDUFB9	GSE92220	.	.	.	.
REG00014	NDUFC1	GSE92220	.	.	.	.
REG00014	NDUFC2	GSE92220	.	.	.	.
REG00014	NDUFS1	GSE92220	.	.	.	.
REG00014	NDUFS2	GSE92220	.	.	.	.
REG00014	NDUFS4	GSE92220	.	.	.	.
REG00014	NDUFS6	GSE92220	.	.	.	.
REG00014	NDUFS7	GSE92220	.	.	.	.
REG00014	NDUFV2	GSE92220	.	.	.	.
REG00014	NDUFV3	GSE92220	.	.	.	.
REG00014	NEDD4	GSE92220	.	.	.	.
REG00014	NEDD4L	GSE92220	.	.	.	.
REG00014	SEPTIN2	GSE92220	.	.	.	.
REG00014	NEDD8	GSE92220	.	.	.	.
REG00014	NEDD9	GSE92220	.	.	.	.
REG00014	NEFH	GSE92220	.	.	.	.
REG00014	NEK2	GSE92220	.	.	.	.
REG00014	NEK3	GSE92220	.	.	.	.
REG00014	NEK5	GSE92220	.	.	.	.
REG00014	NEK6	GSE92220	.	.	.	.
REG00014	SERPINC1	GSE92220	.	.	.	.
REG00014	NES	GSE92220	.	.	.	.
REG00014	NEU1	GSE92220	.	.	.	.
REG00014	NEU3	GSE92220	.	.	.	.
REG00014	NF1	GSE92220	.	.	.	.
REG00014	ZFHX3	GSE92220	.	.	.	.
REG00014	NF2	GSE92220	.	.	.	.
REG00014	NFAT5	GSE92220	.	.	.	.
REG00014	NFATC1	GSE92220	.	.	.	.
REG00014	NFATC2	GSE92220	.	.	.	.
REG00014	NFATC3	GSE92220	.	.	.	.
REG00014	NFE2L1	GSE92220	.	.	.	.
REG00014	NFE2L2	GSE92220	.	.	.	.
REG00014	NFIA	GSE92220	.	.	.	.
REG00014	NFIC	GSE92220	.	.	.	.
REG00014	NFIL3	GSE92220	.	.	.	.
REG00014	NFIX	GSE92220	.	.	.	.
REG00014	NFKB2	GSE92220	.	.	.	.

REG00014	NFKBIA	GSE92220	.	.	.	.
REG00014	ABLIM1	GSE92220	.	.	.	.
REG00014	NFKBIL1	GSE92220	.	.	.	.
REG00014	NFRKB	GSE92220	.	.	.	.
REG00014	NFX1	GSE92220	.	.	.	.
REG00014	NFYC	GSE92220	.	.	.	.
REG00014	NGEF	GSE92220	.	.	.	.
REG00014	NHLH1	GSE92220	.	.	.	.
REG00014	SNU13	GSE92220	.	.	.	.
REG00014	ATE1	GSE92220	.	.	.	.
REG00014	NID1	GSE92220	.	.	.	.
REG00014	NINJ1	GSE92220	.	.	.	.
REG00014	ATF1	GSE92220	.	.	.	.
REG00014	NKTR	GSE92220	.	.	.	.
REG00014	NKX2-4	GSE92220	.	.	.	.
REG00014	NKX3-1	GSE92220	.	.	.	.
REG00014	ATF2	GSE92220	.	.	.	.
REG00014	NMB	GSE92220	.	.	.	.
REG00014	NME1	GSE92220	.	.	.	.
REG00014	ATF3	GSE92220	.	.	.	.
REG00014	NME2	GSE92220	.	.	.	.
REG00014	NME3	GSE92220	.	.	.	.
REG00014	NME4	GSE92220	.	.	.	.
REG00014	NME5	GSE92220	.	.	.	.
REG00014	NQO2	GSE92220	.	.	.	.
REG00014	NMT1	GSE92220	.	.	.	.
REG00014	NMT2	GSE92220	.	.	.	.
REG00014	ATF4	GSE92220	.	.	.	.
REG00014	NNAT	GSE92220	.	.	.	.
REG00014	EMC8	GSE92220	.	.	.	.
REG00014	NODAL	GSE92220	.	.	.	.
REG00014	NOP2	GSE92220	.	.	.	.
REG00014	NOL3	GSE92220	.	.	.	.
REG00014	NONO	GSE92220	.	.	.	.
REG00014	NOS3	GSE92220	.	.	.	.
REG00014	CNOT1	GSE92220	.	.	.	.
REG00014	CNOT3	GSE92220	.	.	.	.
REG00014	CNOT4	GSE92220	.	.	.	.
REG00014	PNP	GSE92220	.	.	.	.
REG00014	NPAS1	GSE92220	.	.	.	.
REG00014	NPAS2	GSE92220	.	.	.	.
REG00014	NPC1	GSE92220	.	.	.	.
REG00014	NPC1L1	GSE92220	.	.	.	.
REG00014	NPDC1	GSE92220	.	.	.	.
REG00014	ATF5	GSE92220	.	.	.	.
REG00014	NPEPPS	GSE92220	.	.	.	.
REG00014	NPHP1	GSE92220	.	.	.	.
REG00014	NPIPA1	GSE92220	.	.	.	.
REG00014	ATF6	GSE92220	.	.	.	.
REG00014	NPM1	GSE92220	.	.	.	.

REG00014	ATF7	GSE92220	.	.	.	.
REG00014	NPM2	GSE92220	.	.	.	.
REG00014	NPM3	GSE92220	.	.	.	.
REG00014	ATIC	GSE92220	.	.	.	.
REG00014	ATM	GSE92220	.	.	.	.
REG00014	NPTXR	GSE92220	.	.	.	.
REG00014	NR0B2	GSE92220	.	.	.	.
REG00014	NR1D1	GSE92220	.	.	.	.
REG00014	NR1D2	GSE92220	.	.	.	.
REG00014	NR1H2	GSE92220	.	.	.	.
REG00014	NR1H3	GSE92220	.	.	.	.
REG00014	NR1H4	GSE92220	.	.	.	.
REG00014	NR1I2	GSE92220	.	.	.	.
REG00014	NR2C1	GSE92220	.	.	.	.
REG00014	NR2C2	GSE92220	.	.	.	.
REG00014	NR2E1	GSE92220	.	.	.	.
REG00014	NR2F1	GSE92220	.	.	.	.
REG00014	NR2F2	GSE92220	.	.	.	.
REG00014	NR2F6	GSE92220	.	.	.	.
REG00014	NR3C1	GSE92220	.	.	.	.
REG00014	ATOX1	GSE92220	.	.	.	.
REG00014	NR4A1	GSE92220	.	.	.	.
REG00014	NR5A1	GSE92220	.	.	.	.
REG00014	NRAS	GSE92220	.	.	.	.
REG00014	ATP1A1	GSE92220	.	.	.	.
REG00014	NRBP1	GSE92220	.	.	.	.
REG00014	NRDC	GSE92220	.	.	.	.
REG00014	NRF1	GSE92220	.	.	.	.
REG00014	NRIP1	GSE92220	.	.	.	.
REG00014	NRP1	GSE92220	.	.	.	.
REG00014	NRTN	GSE92220	.	.	.	.
REG00014	CNTNAP1	GSE92220	.	.	.	.
REG00014	NSMAF	GSE92220	.	.	.	.
REG00014	NT5E	GSE92220	.	.	.	.
REG00014	NT5C2	GSE92220	.	.	.	.
REG00014	NTRK1	GSE92220	.	.	.	.
REG00014	ATP1B1	GSE92220	.	.	.	.
REG00014	NTSR2	GSE92220	.	.	.	.
REG00014	NUBP1	GSE92220	.	.	.	.
REG00014	NUDC	GSE92220	.	.	.	.
REG00014	NUDT3	GSE92220	.	.	.	.
REG00014	NUDT4	GSE92220	.	.	.	.
REG00014	NUDT5	GSE92220	.	.	.	.
REG00014	ATP1B3	GSE92220	.	.	.	.
REG00014	NUMB	GSE92220	.	.	.	.
REG00014	NUMBL	GSE92220	.	.	.	.
REG00014	NUP153	GSE92220	.	.	.	.
REG00014	NUP155	GSE92220	.	.	.	.
REG00014	NUP214	GSE92220	.	.	.	.
REG00014	NXF1	GSE92220	.	.	.	.

REG00014	NXPH3	GSE92220	.	.	.	.
REG00014	NXPH4	GSE92220	.	.	.	.
REG00014	NYX	GSE92220	.	.	.	.
REG00014	OAS2	GSE92220	.	.	.	.
REG00014	OAZ1	GSE92220	.	.	.	.
REG00014	OAZ2	GSE92220	.	.	.	.
REG00014	OAZ3	GSE92220	.	.	.	.
REG00014	ABR	GSE92220	.	.	.	.
REG00014	OC90	GSE92220	.	.	.	.
REG00014	OCA2	GSE92220	.	.	.	.
REG00014	OCLN	GSE92220	.	.	.	.
REG00014	OCRL	GSE92220	.	.	.	.
REG00014	ODC1	GSE92220	.	.	.	.
REG00014	ATP2A1	GSE92220	.	.	.	.
REG00014	ODF2	GSE92220	.	.	.	.
REG00014	ATP2A2	GSE92220	.	.	.	.
REG00014	OGG1	GSE92220	.	.	.	.
REG00014	OGT	GSE92220	.	.	.	.
REG00014	ATP2A3	GSE92220	.	.	.	.
REG00014	OMG	GSE92220	.	.	.	.
REG00014	ONECUT1	GSE92220	.	.	.	.
REG00014	ONECUT2	GSE92220	.	.	.	.
REG00014	ATP2B1	GSE92220	.	.	.	.
REG00014	OPA3	GSE92220	.	.	.	.
REG00014	SIGMAR1	GSE92220	.	.	.	.
REG00014	OR1D5	GSE92220	.	.	.	.
REG00014	ACAA1	GSE92220	.	.	.	.
REG00014	ATP4B	GSE92220	.	.	.	.
REG00014	ATP5A1	GSE92220	.	.	.	.
REG00014	OR2K2	GSE92220	.	.	.	.
REG00014	ATP5B	GSE92220	.	.	.	.
REG00014	ATP5C1	GSE92220	.	.	.	.
REG00014	ATP5D	GSE92220	.	.	.	.
REG00014	OR7D2	GSE92220	.	.	.	.
REG00014	ATP5E	GSE92220	.	.	.	.
REG00014	ATP5F1	GSE92220	.	.	.	.
REG00014	ATP5G1	GSE92220	.	.	.	.
REG00014	ATP5G2	GSE92220	.	.	.	.
REG00014	ATP5G3	GSE92220	.	.	.	.
REG00014	ATP5H	GSE92220	.	.	.	.
REG00014	ATP5J	GSE92220	.	.	.	.
REG00014	ATP5J2	GSE92220	.	.	.	.
REG00014	ORC2	GSE92220	.	.	.	.
REG00014	ORC4	GSE92220	.	.	.	.
REG00014	ORC5	GSE92220	.	.	.	.
REG00014	SLC22A13	GSE92220	.	.	.	.
REG00014	ORM1	GSE92220	.	.	.	.
REG00014	ORM2	GSE92220	.	.	.	.
REG00014	ACACB	GSE92220	.	.	.	.
REG00014	ATP5O	GSE92220	.	.	.	.

REG00014	OSBP	GSE92220	.	.	.	.
REG00014	OSMR	GSE92220	.	.	.	.
REG00014	OXSR1	GSE92220	.	.	.	.
REG00014	ATP6V1A	GSE92220	.	.	.	.
REG00014	OTX1	GSE92220	.	.	.	.
REG00014	OXA1L	GSE92220	.	.	.	.
REG00014	P2RX4	GSE92220	.	.	.	.
REG00014	ATP6V1B2	GSE92220	.	.	.	.
REG00014	P2RY11	GSE92220	.	.	.	.
REG00014	P4HA1	GSE92220	.	.	.	.
REG00014	P4HB	GSE92220	.	.	.	.
REG00014	BLOC1S6	GSE92220	.	.	.	.
REG00014	ATP6V0C	GSE92220	.	.	.	.
REG00014	PA2G4	GSE92220	.	.	.	.
REG00014	PABPC1	GSE92220	.	.	.	.
REG00014	PABPC3	GSE92220	.	.	.	.
REG00014	PABPC4	GSE92220	.	.	.	.
REG00014	ATP6V1C1	GSE92220	.	.	.	.
REG00014	PABPN1	GSE92220	.	.	.	.
REG00014	PCSK6	GSE92220	.	.	.	.
REG00014	PACSIN2	GSE92220	.	.	.	.
REG00014	PACSIN3	GSE92220	.	.	.	.
REG00014	PAFAH1B1	GSE92220	.	.	.	.
REG00014	PAFAH1B2	GSE92220	.	.	.	.
REG00014	PAH	GSE92220	.	.	.	.
REG00014	SERPINE1	GSE92220	.	.	.	.
REG00014	PAK2	GSE92220	.	.	.	.
REG00014	PALM	GSE92220	.	.	.	.
REG00014	PANK1	GSE92220	.	.	.	.
REG00014	PANX2	GSE92220	.	.	.	.
REG00014	PAPSS1	GSE92220	.	.	.	.
REG00014	PARG	GSE92220	.	.	.	.
REG00014	PARN	GSE92220	.	.	.	.
REG00014	ATP6V0B	GSE92220	.	.	.	.
REG00014	PAWR	GSE92220	.	.	.	.
REG00014	PAXIP1	GSE92220	.	.	.	.
REG00014	ATP6V0E1	GSE92220	.	.	.	.
REG00014	PEBP1	GSE92220	.	.	.	.
REG00014	PBX2	GSE92220	.	.	.	.
REG00014	PBX3	GSE92220	.	.	.	.
REG00014	ATP6V1G1	GSE92220	.	.	.	.
REG00014	SLC45A3	GSE92220	.	.	.	.
REG00014	PCBD1	GSE92220	.	.	.	.
REG00014	PCBP1	GSE92220	.	.	.	.
REG00014	PCBP2	GSE92220	.	.	.	.
REG00014	PCBP4	GSE92220	.	.	.	.
REG00014	PCCA	GSE92220	.	.	.	.
REG00014	PCCB	GSE92220	.	.	.	.
REG00014	ATP6V0A4	GSE92220	.	.	.	.
REG00014	ATP6AP1	GSE92220	.	.	.	.

REG00014	ACAD8	GSE92220	.	.	.	.
REG00014	ATP7B	GSE92220	.	.	.	.
REG00014	ATPIF1	GSE92220	.	.	.	.
REG00014	SERPINA5	GSE92220	.	.	.	.
REG00014	PCK1	GSE92220	.	.	.	.
REG00014	PCK2	GSE92220	.	.	.	.
REG00014	PCM1	GSE92220	.	.	.	.
REG00014	PCMT1	GSE92220	.	.	.	.
REG00014	PCNA	GSE92220	.	.	.	.
REG00014	NUP85	GSE92220	.	.	.	.
REG00014	PCOLCE	GSE92220	.	.	.	.
REG00014	PCSK7	GSE92220	.	.	.	.
REG00014	CDK16	GSE92220	.	.	.	.
REG00014	CDK17	GSE92220	.	.	.	.
REG00014	CDK18	GSE92220	.	.	.	.
REG00014	PCTP	GSE92220	.	.	.	.
REG00014	PCYT1A	GSE92220	.	.	.	.
REG00014	PCYT2	GSE92220	.	.	.	.
REG00014	PDCD2	GSE92220	.	.	.	.
REG00014	PDCD4	GSE92220	.	.	.	.
REG00014	PDCD5	GSE92220	.	.	.	.
REG00014	PDCD6	GSE92220	.	.	.	.
REG00014	PDCD6IP	GSE92220	.	.	.	.
REG00014	PDCD7	GSE92220	.	.	.	.
REG00014	MRPS30	GSE92220	.	.	.	.
REG00014	ALDH7A1	GSE92220	.	.	.	.
REG00014	PDCL	GSE92220	.	.	.	.
REG00014	PDE2A	GSE92220	.	.	.	.
REG00014	PDE3A	GSE92220	.	.	.	.
REG00014	PDE3B	GSE92220	.	.	.	.
REG00014	PDE4A	GSE92220	.	.	.	.
REG00014	PDE4C	GSE92220	.	.	.	.
REG00014	PDE4D	GSE92220	.	.	.	.
REG00014	PDE6D	GSE92220	.	.	.	.
REG00014	PDE7A	GSE92220	.	.	.	.
REG00014	PDE8A	GSE92220	.	.	.	.
REG00014	PDE9A	GSE92220	.	.	.	.
REG00014	PDGFA	GSE92220	.	.	.	.
REG00014	PDGFRL	GSE92220	.	.	.	.
REG00014	PDHA1	GSE92220	.	.	.	.
REG00014	PDHB	GSE92220	.	.	.	.
REG00014	PDK1	GSE92220	.	.	.	.
REG00014	PDK2	GSE92220	.	.	.	.
REG00014	PDK3	GSE92220	.	.	.	.
REG00014	PDPK1	GSE92220	.	.	.	.
REG00014	SLC26A4	GSE92220	.	.	.	.
REG00014	PDXK	GSE92220	.	.	.	.
REG00014	SERPINF1	GSE92220	.	.	.	.
REG00014	PEG3	GSE92220	.	.	.	.
REG00014	PELI1	GSE92220	.	.	.	.

REG00014	PEMT	GSE92220	.	.	.	.
REG00014	PEPD	GSE92220	.	.	.	.
REG00014	PER1	GSE92220	.	.	.	.
REG00014	PER2	GSE92220	.	.	.	.
REG00014	PER3	GSE92220	.	.	.	.
REG00014	GATB	GSE92220	.	.	.	.
REG00014	ATRN	GSE92220	.	.	.	.
REG00014	PEX10	GSE92220	.	.	.	.
REG00014	PEX13	GSE92220	.	.	.	.
REG00014	PEX14	GSE92220	.	.	.	.
REG00014	PEX6	GSE92220	.	.	.	.
REG00014	ATRX	GSE92220	.	.	.	.
REG00014	PFAS	GSE92220	.	.	.	.
REG00014	CFP	GSE92220	.	.	.	.
REG00014	PFDN1	GSE92220	.	.	.	.
REG00014	PFDN5	GSE92220	.	.	.	.
REG00014	PFKL	GSE92220	.	.	.	.
REG00014	PFKM	GSE92220	.	.	.	.
REG00014	PFN1	GSE92220	.	.	.	.
REG00014	PFN2	GSE92220	.	.	.	.
REG00014	PGA5	GSE92220	.	.	.	.
REG00014	PGAM1	GSE92220	.	.	.	.
REG00014	PGAM2	GSE92220	.	.	.	.
REG00014	PGC	GSE92220	.	.	.	.
REG00014	PGD	GSE92220	.	.	.	.
REG00014	PGF	GSE92220	.	.	.	.
REG00014	PGK1	GSE92220	.	.	.	.
REG00014	AUH	GSE92220	.	.	.	.
REG00014	PGLS	GSE92220	.	.	.	.
REG00014	PGM1	GSE92220	.	.	.	.
REG00014	PGM3	GSE92220	.	.	.	.
REG00014	PGP	GSE92220	.	.	.	.
REG00014	AUP1	GSE92220	.	.	.	.
REG00014	PHB	GSE92220	.	.	.	.
REG00014	PHF1	GSE92220	.	.	.	.
REG00014	PHF2	GSE92220	.	.	.	.
REG00014	PHF3	GSE92220	.	.	.	.
REG00014	PHGDH	GSE92220	.	.	.	.
REG00014	PHKA2	GSE92220	.	.	.	.
REG00014	PHKB	GSE92220	.	.	.	.
REG00014	PHKG1	GSE92220	.	.	.	.
REG00014	PHKG2	GSE92220	.	.	.	.
REG00014	PHLDA1	GSE92220	.	.	.	.
REG00014	PHLDA3	GSE92220	.	.	.	.
REG00014	PHTF1	GSE92220	.	.	.	.
REG00014	PHYH	GSE92220	.	.	.	.
REG00014	SERPINA1	GSE92220	.	.	.	.
REG00014	SERPINA4	GSE92220	.	.	.	.
REG00014	SERPINB6	GSE92220	.	.	.	.
REG00014	PIGC	GSE92220	.	.	.	.

REG00014	PIGF	GSE92220	.	.	.	.
REG00014	PIGL	GSE92220	.	.	.	.
REG00014	PIGN	GSE92220	.	.	.	.
REG00014	PIK3C2A	GSE92220	.	.	.	.
REG00014	PIK3CA	GSE92220	.	.	.	.
REG00014	PIK3CB	GSE92220	.	.	.	.
REG00014	PIK3R1	GSE92220	.	.	.	.
REG00014	PIK3R2	GSE92220	.	.	.	.
REG00014	PIK3R3	GSE92220	.	.	.	.
REG00014	PI4KB	GSE92220	.	.	.	.
REG00014	PIM1	GSE92220	.	.	.	.
REG00014	PIM2	GSE92220	.	.	.	.
REG00014	PIN1	GSE92220	.	.	.	.
REG00014	PIP5K1A	GSE92220	.	.	.	.
REG00014	PIP5K1B	GSE92220	.	.	.	.
REG00014	PIP5K1C	GSE92220	.	.	.	.
REG00014	PIP4K2A	GSE92220	.	.	.	.
REG00014	PIP4K2B	GSE92220	.	.	.	.
REG00014	PISD	GSE92220	.	.	.	.
REG00014	ACADS	GSE92220	.	.	.	.
REG00014	PITPNA	GSE92220	.	.	.	.
REG00014	PITPNB	GSE92220	.	.	.	.
REG00014	PITPNM1	GSE92220	.	.	.	.
REG00014	PKD2	GSE92220	.	.	.	.
REG00014	PKD2L2	GSE92220	.	.	.	.
REG00014	PKIG	GSE92220	.	.	.	.
REG00014	PKLR	GSE92220	.	.	.	.
REG00014	PKM	GSE92220	.	.	.	.
REG00014	PKNOX1	GSE92220	.	.	.	.
REG00014	PKP2	GSE92220	.	.	.	.
REG00014	PKP3	GSE92220	.	.	.	.
REG00014	PKP4	GSE92220	.	.	.	.
REG00014	PLA2G10	GSE92220	.	.	.	.
REG00014	AXIN1	GSE92220	.	.	.	.
REG00014	PLA2G2A	GSE92220	.	.	.	.
REG00014	PLA2G4B	GSE92220	.	.	.	.
REG00014	PLA2G4C	GSE92220	.	.	.	.
REG00014	AXIN2	GSE92220	.	.	.	.
REG00014	PLAG1	GSE92220	.	.	.	.
REG00014	AXL	GSE92220	.	.	.	.
REG00014	PLCB3	GSE92220	.	.	.	.
REG00014	PPP1R14B	GSE92220	.	.	.	.
REG00014	PLCD4	GSE92220	.	.	.	.
REG00014	PLCG1	GSE92220	.	.	.	.
REG00014	PLD1	GSE92220	.	.	.	.
REG00014	PLEC	GSE92220	.	.	.	.
REG00014	PLGLB2	GSE92220	.	.	.	.
REG00014	SERPINF2	GSE92220	.	.	.	.
REG00014	PLK1	GSE92220	.	.	.	.
REG00014	PLOD1	GSE92220	.	.	.	.

REG00014	PLOD2	GSE92220	.	.	.	.
REG00014	PLOD3	GSE92220	.	.	.	.
REG00014	PLRG1	GSE92220	.	.	.	.
REG00014	PLS3	GSE92220	.	.	.	.
REG00014	PLSCR1	GSE92220	.	.	.	.
REG00014	PLTP	GSE92220	.	.	.	.
REG00014	PLXNA1	GSE92220	.	.	.	.
REG00014	ACADSB	GSE92220	.	.	.	.
REG00014	AZGP1	GSE92220	.	.	.	.
REG00014	PLXNA2	GSE92220	.	.	.	.
REG00014	PLXNA3	GSE92220	.	.	.	.
REG00014	PLXNB1	GSE92220	.	.	.	.
REG00014	PLXNB2	GSE92220	.	.	.	.
REG00014	PLXNB3	GSE92220	.	.	.	.
REG00014	PLXNC1	GSE92220	.	.	.	.
REG00014	PLXND1	GSE92220	.	.	.	.
REG00014	PMF1	GSE92220	.	.	.	.
REG00014	PML	GSE92220	.	.	.	.
REG00014	PMM1	GSE92220	.	.	.	.
REG00014	PMM2	GSE92220	.	.	.	.
REG00014	PMPCB	GSE92220	.	.	.	.
REG00014	PMS1	GSE92220	.	.	.	.
REG00014	PMS2	GSE92220	.	.	.	.
REG00014	GIGYF1	GSE92220	.	.	.	.
REG00014	B2M	GSE92220	.	.	.	.
REG00014	PMVK	GSE92220	.	.	.	.
REG00014	PNKD	GSE92220	.	.	.	.
REG00014	PNKP	GSE92220	.	.	.	.
REG00014	PNMA1	GSE92220	.	.	.	.
REG00014	PNMT	GSE92220	.	.	.	.
REG00014	PNN	GSE92220	.	.	.	.
REG00014	PNOC	GSE92220	.	.	.	.
REG00014	B3GALT2	GSE92220	.	.	.	.
REG00014	PODXL	GSE92220	.	.	.	.
REG00014	POLB	GSE92220	.	.	.	.
REG00014	POLD1	GSE92220	.	.	.	.
REG00014	POLE	GSE92220	.	.	.	.
REG00014	POLG	GSE92220	.	.	.	.
REG00014	POLG2	GSE92220	.	.	.	.
REG00014	POLL	GSE92220	.	.	.	.
REG00014	POLR2A	GSE92220	.	.	.	.
REG00014	POLR2B	GSE92220	.	.	.	.
REG00014	POLR2C	GSE92220	.	.	.	.
REG00014	B3GALT4	GSE92220	.	.	.	.
REG00014	POLR2D	GSE92220	.	.	.	.
REG00014	POLR2E	GSE92220	.	.	.	.
REG00014	POLR2H	GSE92220	.	.	.	.
REG00014	POLR2L	GSE92220	.	.	.	.
REG00014	POLRMT	GSE92220	.	.	.	.
REG00014	POMT1	GSE92220	.	.	.	.

REG00014	POMZP3	GSE92220	.	.	.	.
REG00014	PON1	GSE92220	.	.	.	.
REG00014	PON2	GSE92220	.	.	.	.
REG00014	PON3	GSE92220	.	.	.	.
REG00014	CNOT8	GSE92220	.	.	.	.
REG00014	POU2F1	GSE92220	.	.	.	.
REG00014	POU3F2	GSE92220	.	.	.	.
REG00014	POU4F1	GSE92220	.	.	.	.
REG00014	B3GAT2	GSE92220	.	.	.	.
REG00014	SLC43A1	GSE92220	.	.	.	.
REG00014	PPA1	GSE92220	.	.	.	.
REG00014	PLPP3	GSE92220	.	.	.	.
REG00014	B3GAT3	GSE92220	.	.	.	.
REG00014	PLPP2	GSE92220	.	.	.	.
REG00014	PPARD	GSE92220	.	.	.	.
REG00014	PPARG	GSE92220	.	.	.	.
REG00014	PPARGC1A	GSE92220	.	.	.	.
REG00014	PPEF2	GSE92220	.	.	.	.
REG00014	PPFIA1	GSE92220	.	.	.	.
REG00014	PPFIBP1	GSE92220	.	.	.	.
REG00014	B4GALT2	GSE92220	.	.	.	.
REG00014	CTSA	GSE92220	.	.	.	.
REG00014	PPIA	GSE92220	.	.	.	.
REG00014	PPIB	GSE92220	.	.	.	.
REG00014	PPID	GSE92220	.	.	.	.
REG00014	PPIF	GSE92220	.	.	.	.
REG00014	B4GALT3	GSE92220	.	.	.	.
REG00014	PPIL2	GSE92220	.	.	.	.
REG00014	PPIL3	GSE92220	.	.	.	.
REG00014	PPM1A	GSE92220	.	.	.	.
REG00014	PPM1D	GSE92220	.	.	.	.
REG00014	PPM1G	GSE92220	.	.	.	.
REG00014	B4GALT5	GSE92220	.	.	.	.
REG00014	PPOX	GSE92220	.	.	.	.
REG00014	PPP1CA	GSE92220	.	.	.	.
REG00014	PPP1CB	GSE92220	.	.	.	.
REG00014	PPP1CC	GSE92220	.	.	.	.
REG00014	PPP1R10	GSE92220	.	.	.	.
REG00014	PPP1R11	GSE92220	.	.	.	.
REG00014	PPP1R2	GSE92220	.	.	.	.
REG00014	PPP1R8	GSE92220	.	.	.	.
REG00014	PPP2CA	GSE92220	.	.	.	.
REG00014	ACAT1	GSE92220	.	.	.	.
REG00014	B4GALT7	GSE92220	.	.	.	.
REG00014	PPP2CB	GSE92220	.	.	.	.
REG00014	PPP2R1A	GSE92220	.	.	.	.
REG00014	PPP2R1B	GSE92220	.	.	.	.
REG00014	PPP2R2A	GSE92220	.	.	.	.
REG00014	PTPA	GSE92220	.	.	.	.
REG00014	PPP2R5A	GSE92220	.	.	.	.

REG00014	PPP2R5C	GSE92220	.	.	.	.
REG00014	PPP2R5E	GSE92220	.	.	.	.
REG00014	PPP3CA	GSE92220	.	.	.	.
REG00014	PPP3CB	GSE92220	.	.	.	.
REG00014	PPP3CC	GSE92220	.	.	.	.
REG00014	PPP3R1	GSE92220	.	.	.	.
REG00014	PPP4C	GSE92220	.	.	.	.
REG00014	BAAT	GSE92220	.	.	.	.
REG00014	PPP4R1	GSE92220	.	.	.	.
REG00014	PPP5C	GSE92220	.	.	.	.
REG00014	PPT1	GSE92220	.	.	.	.
REG00014	PQBP1	GSE92220	.	.	.	.
REG00014	PRC1	GSE92220	.	.	.	.
REG00014	PRCC	GSE92220	.	.	.	.
REG00014	PRCP	GSE92220	.	.	.	.
REG00014	PRDM2	GSE92220	.	.	.	.
REG00014	PRDM4	GSE92220	.	.	.	.
REG00014	BACH1	GSE92220	.	.	.	.
REG00014	PRDX1	GSE92220	.	.	.	.
REG00014	PRDX2	GSE92220	.	.	.	.
REG00014	PRDX3	GSE92220	.	.	.	.
REG00014	PRDX5	GSE92220	.	.	.	.
REG00014	PREB	GSE92220	.	.	.	.
REG00014	PREP	GSE92220	.	.	.	.
REG00014	SLC26A5	GSE92220	.	.	.	.
REG00014	PRG4	GSE92220	.	.	.	.
REG00014	PRH1	GSE92220	.	.	.	.
REG00014	BAG1	GSE92220	.	.	.	.
REG00014	PRKAA1	GSE92220	.	.	.	.
REG00014	PRKAB1	GSE92220	.	.	.	.
REG00014	PRKAB2	GSE92220	.	.	.	.
REG00014	BAG2	GSE92220	.	.	.	.
REG00014	PRKACA	GSE92220	.	.	.	.
REG00014	PRKAG1	GSE92220	.	.	.	.
REG00014	PRKAR1A	GSE92220	.	.	.	.
REG00014	BAG3	GSE92220	.	.	.	.
REG00014	PRKAR1B	GSE92220	.	.	.	.
REG00014	ZMYND8	GSE92220	.	.	.	.
REG00014	PRKCD	GSE92220	.	.	.	.
REG00014	ACAT2	GSE92220	.	.	.	.
REG00014	BAG4	GSE92220	.	.	.	.
REG00014	PRKCG	GSE92220	.	.	.	.
REG00014	PKN1	GSE92220	.	.	.	.
REG00014	PKN2	GSE92220	.	.	.	.
REG00014	PRKD3	GSE92220	.	.	.	.
REG00014	BAG5	GSE92220	.	.	.	.
REG00014	PRKCZ	GSE92220	.	.	.	.
REG00014	PRKDC	GSE92220	.	.	.	.
REG00014	EIF2AK2	GSE92220	.	.	.	.
REG00014	PRKRA	GSE92220	.	.	.	.

REG00014	DNAJC3	GSE92220	.	.	.	.
REG00014	THAP12	GSE92220	.	.	.	.
REG00014	PRNP	GSE92220	.	.	.	.
REG00014	PROC	GSE92220	.	.	.	.
REG00014	PROS1	GSE92220	.	.	.	.
REG00014	PROSC	GSE92220	.	.	.	.
REG00014	PROX1	GSE92220	.	.	.	.
REG00014	MAGI1	GSE92220	.	.	.	.
REG00014	PROZ	GSE92220	.	.	.	.
REG00014	PRPS1	GSE92220	.	.	.	.
REG00014	PRPS2	GSE92220	.	.	.	.
REG00014	PRPSAP1	GSE92220	.	.	.	.
REG00014	PRPSAP2	GSE92220	.	.	.	.
REG00014	PRRG1	GSE92220	.	.	.	.
REG00014	BAIAP2	GSE92220	.	.	.	.
REG00014	LGMN	GSE92220	.	.	.	.
REG00014	LONP1	GSE92220	.	.	.	.
REG00014	BAIAP3	GSE92220	.	.	.	.
REG00014	BAK1	GSE92220	.	.	.	.
REG00014	PRSS8	GSE92220	.	.	.	.
REG00014	PSAP	GSE92220	.	.	.	.
REG00014	SLC33A1	GSE92220	.	.	.	.
REG00014	BAP1	GSE92220	.	.	.	.
REG00014	CYTH1	GSE92220	.	.	.	.
REG00014	CYTH2	GSE92220	.	.	.	.
REG00014	PSD	GSE92220	.	.	.	.
REG00014	PSEN1	GSE92220	.	.	.	.
REG00014	PSKH1	GSE92220	.	.	.	.
REG00014	PSMA1	GSE92220	.	.	.	.
REG00014	PSMA2	GSE92220	.	.	.	.
REG00014	PSMA3	GSE92220	.	.	.	.
REG00014	PSMA4	GSE92220	.	.	.	.
REG00014	PSMA5	GSE92220	.	.	.	.
REG00014	PSMA6	GSE92220	.	.	.	.
REG00014	PSMA7	GSE92220	.	.	.	.
REG00014	PSMB1	GSE92220	.	.	.	.
REG00014	PSMB2	GSE92220	.	.	.	.
REG00014	PSMB3	GSE92220	.	.	.	.
REG00014	PSMB4	GSE92220	.	.	.	.
REG00014	PSMB5	GSE92220	.	.	.	.
REG00014	PSMB7	GSE92220	.	.	.	.
REG00014	PSMB8	GSE92220	.	.	.	.
REG00014	PSMC2	GSE92220	.	.	.	.
REG00014	PSMC3	GSE92220	.	.	.	.
REG00014	PSMC4	GSE92220	.	.	.	.
REG00014	PSMC5	GSE92220	.	.	.	.
REG00014	PSMC6	GSE92220	.	.	.	.
REG00014	PSMD1	GSE92220	.	.	.	.
REG00014	PSMD10	GSE92220	.	.	.	.
REG00014	PSMD11	GSE92220	.	.	.	.

REG00014	PSMD12	GSE92220	.	.	.	.
REG00014	PSMD13	GSE92220	.	.	.	.
REG00014	PSMD2	GSE92220	.	.	.	.
REG00014	PSMD3	GSE92220	.	.	.	.
REG00014	PSMD4	GSE92220	.	.	.	.
REG00014	PSMD6	GSE92220	.	.	.	.
REG00014	PSMD7	GSE92220	.	.	.	.
REG00014	PSMD8	GSE92220	.	.	.	.
REG00014	PSME1	GSE92220	.	.	.	.
REG00014	PSME2	GSE92220	.	.	.	.
REG00014	PSME3	GSE92220	.	.	.	.
REG00014	PSMF1	GSE92220	.	.	.	.
REG00014	PSPH	GSE92220	.	.	.	.
REG00014	PSPN	GSE92220	.	.	.	.
REG00014	PSTPIP2	GSE92220	.	.	.	.
REG00014	PTAFR	GSE92220	.	.	.	.
REG00014	PTCH1	GSE92220	.	.	.	.
REG00014	PTDSS1	GSE92220	.	.	.	.
REG00014	PTEN	GSE92220	.	.	.	.
REG00014	BAX	GSE92220	.	.	.	.
REG00014	PTGER1	GSE92220	.	.	.	.
REG00014	BAZ1A	GSE92220	.	.	.	.
REG00014	PTGIS	GSE92220	.	.	.	.
REG00014	PTGS1	GSE92220	.	.	.	.
REG00014	PTH2R	GSE92220	.	.	.	.
REG00014	BAZ1B	GSE92220	.	.	.	.
REG00014	PTK2	GSE92220	.	.	.	.
REG00014	PTK7	GSE92220	.	.	.	.
REG00014	BAZ2A	GSE92220	.	.	.	.
REG00014	PTMA	GSE92220	.	.	.	.
REG00014	PTOV1	GSE92220	.	.	.	.
REG00014	PTP4A1	GSE92220	.	.	.	.
REG00014	PTP4A2	GSE92220	.	.	.	.
REG00014	PTP4A3	GSE92220	.	.	.	.
REG00014	HACD2	GSE92220	.	.	.	.
REG00014	PTPN1	GSE92220	.	.	.	.
REG00014	PTPN11	GSE92220	.	.	.	.
REG00014	PTPN12	GSE92220	.	.	.	.
REG00014	PTPN14	GSE92220	.	.	.	.
REG00014	PTPN18	GSE92220	.	.	.	.
REG00014	PTPN3	GSE92220	.	.	.	.
REG00014	PTPN4	GSE92220	.	.	.	.
REG00014	BBS1	GSE92220	.	.	.	.
REG00014	PTPN9	GSE92220	.	.	.	.
REG00014	PTPRA	GSE92220	.	.	.	.
REG00014	PTPRCAP	GSE92220	.	.	.	.
REG00014	PTPRF	GSE92220	.	.	.	.
REG00014	PTPRG	GSE92220	.	.	.	.
REG00014	PTPRH	GSE92220	.	.	.	.
REG00014	PTPRK	GSE92220	.	.	.	.

REG00014	PTPRM	GSE92220	.	.	.	.
REG00014	PTPRN2	GSE92220	.	.	.	.
REG00014	PTPRO	GSE92220	.	.	.	.
REG00014	PTPRS	GSE92220	.	.	.	.
REG00014	PTPRU	GSE92220	.	.	.	.
REG00014	PURA	GSE92220	.	.	.	.
REG00014	PVR	GSE92220	.	.	.	.
REG00014	NECTIN1	GSE92220	.	.	.	.
REG00014	NECTIN2	GSE92220	.	.	.	.
REG00014	BCAR1	GSE92220	.	.	.	.
REG00014	PWP2	GSE92220	.	.	.	.
REG00014	PEX19	GSE92220	.	.	.	.
REG00014	PEX5	GSE92220	.	.	.	.
REG00014	PYCR1	GSE92220	.	.	.	.
REG00014	ALDH18A1	GSE92220	.	.	.	.
REG00014	PYGB	GSE92220	.	.	.	.
REG00014	PYGL	GSE92220	.	.	.	.
REG00014	PYGM	GSE92220	.	.	.	.
REG00014	BCAR3	GSE92220	.	.	.	.
REG00014	PZP	GSE92220	.	.	.	.
REG00014	QARS	GSE92220	.	.	.	.
REG00014	QDPR	GSE92220	.	.	.	.
REG00014	QPRT	GSE92220	.	.	.	.
REG00014	QSOX1	GSE92220	.	.	.	.
REG00014	R3HDM1	GSE92220	.	.	.	.
REG00014	RAB1A	GSE92220	.	.	.	.
REG00014	RAB10	GSE92220	.	.	.	.
REG00014	BCAT1	GSE92220	.	.	.	.
REG00014	RAB11A	GSE92220	.	.	.	.
REG00014	RAB13	GSE92220	.	.	.	.
REG00014	RAB2A	GSE92220	.	.	.	.
REG00014	RAB22A	GSE92220	.	.	.	.
REG00014	RAB24	GSE92220	.	.	.	.
REG00014	RAB27A	GSE92220	.	.	.	.
REG00014	RAB28	GSE92220	.	.	.	.
REG00014	RGL2	GSE92220	.	.	.	.
REG00014	RAB35	GSE92220	.	.	.	.
REG00014	RAB36	GSE92220	.	.	.	.
REG00014	RAB3D	GSE92220	.	.	.	.
REG00014	BCCIP	GSE92220	.	.	.	.
REG00014	RAB4A	GSE92220	.	.	.	.
REG00014	RAB5A	GSE92220	.	.	.	.
REG00014	RAB5B	GSE92220	.	.	.	.
REG00014	RAB6A	GSE92220	.	.	.	.
REG00014	KIF20A	GSE92220	.	.	.	.
REG00014	RAB29	GSE92220	.	.	.	.
REG00014	RABAC1	GSE92220	.	.	.	.
REG00014	RABGGTA	GSE92220	.	.	.	.
REG00014	RABGGTB	GSE92220	.	.	.	.
REG00014	RABIF	GSE92220	.	.	.	.

REG00014	RAC1	GSE92220	.	.	.	.
REG00014	RAC3	GSE92220	.	.	.	.
REG00014	RAD1	GSE92220	.	.	.	.
REG00014	RAD17	GSE92220	.	.	.	.
REG00014	RAD23A	GSE92220	.	.	.	.
REG00014	RAD23B	GSE92220	.	.	.	.
REG00014	RAD50	GSE92220	.	.	.	.
REG00014	RAD52	GSE92220	.	.	.	.
REG00014	RAE1	GSE92220	.	.	.	.
REG00014	RAF1	GSE92220	.	.	.	.
REG00014	MOK	GSE92220	.	.	.	.
REG00014	RAI1	GSE92220	.	.	.	.
REG00014	RALA	GSE92220	.	.	.	.
REG00014	RALB	GSE92220	.	.	.	.
REG00014	RALBP1	GSE92220	.	.	.	.
REG00014	RALGDS	GSE92220	.	.	.	.
REG00014	RAN	GSE92220	.	.	.	.
REG00014	RANBP1	GSE92220	.	.	.	.
REG00014	RANBP2	GSE92220	.	.	.	.
REG00014	RANBP3	GSE92220	.	.	.	.
REG00014	IPO7	GSE92220	.	.	.	.
REG00014	IPO8	GSE92220	.	.	.	.
REG00014	RANGAP1	GSE92220	.	.	.	.
REG00014	RAP1A	GSE92220	.	.	.	.
REG00014	RAP1B	GSE92220	.	.	.	.
REG00014	RAP1GAP	GSE92220	.	.	.	.
REG00014	RAP1GDS1	GSE92220	.	.	.	.
REG00014	RAP2A	GSE92220	.	.	.	.
REG00014	RAP2B	GSE92220	.	.	.	.
REG00014	RAPSN	GSE92220	.	.	.	.
REG00014	RARA	GSE92220	.	.	.	.
REG00014	RARRES2	GSE92220	.	.	.	.
REG00014	RARS	GSE92220	.	.	.	.
REG00014	RASA1	GSE92220	.	.	.	.
REG00014	RASA2	GSE92220	.	.	.	.
REG00014	RASSF2	GSE92220	.	.	.	.
REG00014	ARID4A	GSE92220	.	.	.	.
REG00014	RBBP4	GSE92220	.	.	.	.
REG00014	RBBP6	GSE92220	.	.	.	.
REG00014	BCL10	GSE92220	.	.	.	.
REG00014	RBBP7	GSE92220	.	.	.	.
REG00014	RBBP9	GSE92220	.	.	.	.
REG00014	RBL1	GSE92220	.	.	.	.
REG00014	RBL2	GSE92220	.	.	.	.
REG00014	RBM3	GSE92220	.	.	.	.
REG00014	RBM4	GSE92220	.	.	.	.
REG00014	RBM5	GSE92220	.	.	.	.
REG00014	RBM6	GSE92220	.	.	.	.
REG00014	RBM7	GSE92220	.	.	.	.
REG00014	RBM8A	GSE92220	.	.	.	.

REG00014	RBFOX2	GSE92220	.	.	.	.
REG00014	RBMS1	GSE92220	.	.	.	.
REG00014	RBMX	GSE92220	.	.	.	.
REG00014	BCL2L1	GSE92220	.	.	.	.
REG00014	RBP4	GSE92220	.	.	.	.
REG00014	RBX1	GSE92220	.	.	.	.
REG00014	BCL2L10	GSE92220	.	.	.	.
REG00014	RCN1	GSE92220	.	.	.	.
REG00014	RCN2	GSE92220	.	.	.	.
REG00014	BCL2L11	GSE92220	.	.	.	.
REG00014	PRPH2	GSE92220	.	.	.	.
REG00014	RDX	GSE92220	.	.	.	.
REG00014	RECQL4	GSE92220	.	.	.	.
REG00014	BCL2L2	GSE92220	.	.	.	.
REG00014	RECQL5	GSE92220	.	.	.	.
REG00014	REL	GSE92220	.	.	.	.
REG00014	RELA	GSE92220	.	.	.	.
REG00014	RELB	GSE92220	.	.	.	.
REG00014	RELN	GSE92220	.	.	.	.
REG00014	RENBP	GSE92220	.	.	.	.
REG00014	UPF1	GSE92220	.	.	.	.
REG00014	RERE	GSE92220	.	.	.	.
REG00014	REST	GSE92220	.	.	.	.
REG00014	REV3L	GSE92220	.	.	.	.
REG00014	RFC1	GSE92220	.	.	.	.
REG00014	RFC3	GSE92220	.	.	.	.
REG00014	RFC5	GSE92220	.	.	.	.
REG00014	RFNG	GSE92220	.	.	.	.
REG00014	TRIM27	GSE92220	.	.	.	.
REG00014	TRIM13	GSE92220	.	.	.	.
REG00014	RFXAP	GSE92220	.	.	.	.
REG00014	RGN	GSE92220	.	.	.	.
REG00014	RGS11	GSE92220	.	.	.	.
REG00014	RGS12	GSE92220	.	.	.	.
REG00014	RGS3	GSE92220	.	.	.	.
REG00014	RSC1A1	GSE90639	Inf	.	.	.
REG00014	TREX1	GSE90639	Inf	.	.	.
REG00014	ZNF22	GSE90639	Inf	.	.	.
REG00014	DEXI	GSE90639	Inf	.	.	.
REG00014	HRASLS	GSE90639	Inf	.	.	.
REG00014	RRAS2	GSE90639	Inf	.	.	.
REG00014	CDK3	GSE90639	Inf	.	.	.
REG00014	NUDT11	GSE90639	Inf	.	.	.
REG00014	P2RY12	GSE90639	Inf	.	.	.
REG00014	FOXD4L1	GSE90639	Inf	.	.	.
REG00014	C22orf23	GSE90639	Inf	.	.	.
REG00014	TAS2R46	GSE90639	Inf	.	.	.
REG00014	GJD3	GSE90639	Inf	.	.	.
REG00014	CMTM1	GSE90639	Inf	.	.	.
REG00014	PGBD3	GSE90639	Inf	.	.	.

REG00014	CKS2	GSE90639	Inf	.	.	.
REG00014	CENPU	GSE90639	Inf	.	.	.
REG00014	FSIP1	GSE90639	Inf	.	.	.
REG00014	COX8A	GSE90639	Inf	.	.	.
REG00014	PLGRKT	GSE90639	Inf	.	.	.
REG00014	CTAG1A	GSE90639	Inf	.	.	.
REG00014	TEX30	GSE90639	Inf	.	.	.
REG00014	UFSP2	GSE90639	Inf	.	.	.
REG00014	C10orf95	GSE90639	Inf	.	.	.
REG00014	PIGY	GSE90639	Inf	.	.	.
REG00014	TMED6	GSE90639	Inf	.	.	.
REG00014	CCDC107	GSE90639	Inf	.	.	.
REG00014	TMEM27	GSE90639	Inf	.	.	.
REG00014	HIST2H2AA4	GSE90639	Inf	.	.	.
REG00014	SP9	GSE90639	Inf	.	.	.
REG00014	FOXD4L6	GSE90639	Inf	.	.	.
REG00014	OST4	GSE90639	Inf	.	.	.
REG00014	SDHAF1	GSE90639	Inf	.	.	.
REG00014	TOMM6	GSE90639	Inf	.	.	.
REG00014	CMC4	GSE90639	Inf	.	.	.
REG00014	ZMYM6NB	GSE90639	Inf	.	.	.
REG00014	NPIPA2	GSE90639	Inf	.	.	.
REG00014	GDF1	GSE90639	Inf	.	.	.
REG00014	FSBP	GSE90639	Inf	.	.	.
REG00014	GNG10	GSE90639	Inf	.	.	.
REG00014	PYURF	GSE90639	Inf	.	.	.
REG00014	GOLGA8T	GSE90639	Inf	.	.	.
REG00014	GPR52	GSE90639	Inf	.	.	.
REG00014	HIST1H1C	GSE90639	Inf	.	.	.
REG00014	HIST1H2AK	GSE90639	Inf	.	.	.
REG00014	HIST1H2AD	GSE90639	Inf	.	.	.
REG00014	HIST1H2AB	GSE90639	Inf	.	.	.
REG00014	HIST1H2BL	GSE90639	Inf	.	.	.
REG00014	HIST1H3A	GSE90639	Inf	.	.	.
REG00014	HIST1H3J	GSE90639	Inf	.	.	.
REG00014	HIST1H4A	GSE90639	Inf	.	.	.
REG00014	HIST1H4K	GSE90639	Inf	.	.	.
REG00014	HIST1H4C	GSE90639	Inf	.	.	.
REG00014	HNRNPA1P48	GSE90639	Inf	.	.	.
REG00014	MGAT2	GSE90639	Inf	.	.	.
REG00014	MPHOSPH6	GSE90639	Inf	.	.	.
REG00014	NDUFC1	GSE90639	Inf	.	.	.
REG00014	PRH1	GSE90639	Inf	.	.	.
